# Gastroesophageal disease risk and inhalational exposure a systematic review and meta-analysis

**DOI:** 10.1038/s41598-025-06620-7

**Published:** 2025-07-02

**Authors:** Daniel Hyun Kim, Sanjiti Podury, Aida Fallah Zadeh, Tara Mahmoodi, Sophia Kwon, Gabriele Grunig, Mengling Liu, Anna Nolan

**Affiliations:** 1 Division of Pulmonary, Critical Care and Sleep Medicine, Department of Medicine, New York University Grossman School of Medicine (NYUGSoM), New Bellevue, 16N Room 20 (Lab), 462 1st Avenue, New York, NY 10016 USA; 2 Division of Environmental Medicine, Department of Medicine, NYUGSoM, New York, NY USA; 3 Division of Biostatistics, Department of Population Health, NYUGSoM, New York, NY USA

**Keywords:** Systematic review, Environmental exposure, Smoking, Particulate matter, Aerodigestive disease, Meta-analysis, Occupational health, Epidemiology, Quality of life, Risk factors, Oesophageal diseases, Stomach diseases

## Abstract

Environmental exposure-associated diseases, particularly in the context of rising air pollution and inhalant use, are an active area of research. Our group is dedicated to the study of exposure-related inflammation and its downstream adverse health effects. While many studies have focused on the impact of environmental exposures on respiratory sequelae, there is growing evidence of the involvement of other systems including gastrointestinal. This systematic review provides updates on the associations between inhalation exposures and the risk of upper gastrointestinal disease. Primary search identified *N* = 764 PubMed and *N* = 1,036 Web of Science studies, of which *N* = 111 met eligibility criteria. Our systematic review and meta-analysis showed significant associations between inhalational exposures (cigarette smoking, waterpipe smoking, and particulate matter) and upper gastrointestinal diseases. The pooled estimate of esophagitis was 1.32 (95% confidence interval [CI], 1.06–1.65; I^2^:86%), gastroesophageal reflux disease was 1.71 (1.14–2.55; I²:94%), peptic ulcer disease was 1.21 (1.03–1.43; I^2^:93%), esophageal cancer was 1.83 (1.54–2.18; I^2^:73%), and gastric cancer was 1.71 (1.39–2.10; I^2^:73%). However, the pooled estimate for Barrett’s esophagus was 0.93 (0.65–1.34; I^2^:76%), indicating no significant association. Sensitivity analyses confirmed these findings. Risk of bias assessment showed most studies were of good quality. Our findings emphasize the impact of inhalational exposures on gastrointestinal disease risk, highlighting the need for further research to better understand this interaction and targeted public health interventions.

**Supplementary Information:**

The online version contains supplementary material available at 10.1038/s41598-025-06620-7.

## Introduction

Exposome-associated morbidity and mortality is a global health concern. Environmental exposures that individuals encounter over their lifetime include air pollution, water pollution, diet, and radiation. The exposome has been linked to heterogeneous negative health effects, and mechanisms remain elusive in many disease states. Studying the exposome provides valuable insights into the interplay between environmental factors and human health^1^.

Of the various environmental exposures, inhalational exposure has been of great interest, especially in the context of rising levels of global air pollution due to global warming, wildfires, wars, and population growth. Studies investigating the link between environmental exposures and disease have the potential to impact millions globally. Air pollution is associated with 7 million premature deaths annually, and levels have steadily risen over the past few decades^2,3^. Over half of the world’s population is exposed to levels of air pollution that are substantially above the World Health Organization (WHO) air quality guidelines^4^. A greater proportion of non-communicable diseases are attributable to environmental exposure in developing countries that utilize industrial production factories, without the most modern emission safeguards and therefore are primary contributors to emissions^5^.

Particulate matter (PM) exposure, is a global cause of significant aerodigestive morbidity and mortality^6,7^. Globally, gastroesophageal reflux disease (GERD) prevalence is 10–25%^8,9^. GERD is the most prevalent gastrointestinal disorder affecting at least 20% of the United States of America (USA) population, and leading to substantial morbidity^8,10–12^. Aerodigestive complications also include Barrett’s Esophagus (BE), and malignancy such as esophageal adenocarcinoma (EAC)^13–15^. The aerodigestive disease can also induce or worsen airway hyperreactivity (AHR) and other forms of obstructive airway disease (OAD). This may be explained by the clearing mechanism of the respiratory system and its proximity to the digestive system at the pharynx leading to gastric reflux being transported into the lungs. However, this is an area of active investigation^16^. Prior systematic reviews have only focused on single inhalational exposures and/or single diseases/outcomes^17–22^.

Our group has focused on the adverse health effects secondary to the destruction of the World Trade Center (WTC) on September 11, 2001 (9/11). This intense PM exposure of first responders and inhabitants of New York City (NYC) led to heterogeneous end-organ involvement^23,24^. WTC-PM exposure is associated with OAD and gastroesophageal diseases including GERD and BE^25–27^. Approximately 44% of WTC rescue/recovery workers had developed GERD symptoms by 2005^28^. There is also evidence of comorbid GERD and OAD, as WTC-exposed firefighters with OAD had a 3-fold higher risk of developing GERD^27,29^. Therefore, due to our interest in a more diverse exposure profile we have also designed our systematic review to focus on heterogeneous gastroesophageal diseases. Specifically, we investigated the associations between the exposures (PM and smoking) with diseases of the gastrointestinal tract (GERD, BE, and malignancy).

## Methods

Search Strategy & Identification. Our systematic review adhered to the Preferred Reporting Items for Systematic Reviews and Meta-Analysis (PRISMA) guidelines^30,31^. Our Population, Exposure, Outcome (PEO) question was to investigate among adult populations (P), whether there is an association between inhalational exposure (e.g., air pollution, cigarette/tobacco smoke, marijuana smoke, vape/e-cigarette aerosols) (E) and esophageal or gastric disorders/disease (O).

The protocol of our systematic review was registered on PROSPERO, April 29, 2024, and can be accessed at Prospero ID 536,834. A comprehensive search was conducted in PubMed (May 1, 2024) and Web of Science (WoS, August 23, 2024) using predefined MeSH terms related to inhalational exposures (e.g., PM, tobacco smoke, vaping, marijuana) and upper gastrointestinal diseases (e.g., GERD, BE, peptic ulcer disease (PUD), esophagitis, esophageal cancer (ECa), gastric cancer (GCa)). Our search strategy included automatic database filters (full-text, human subjects, English language, publication within the last 10 years) and manual reference-list screening to identify relevant studies.

The following MeSH Terms were searched for using the MeSH Database:


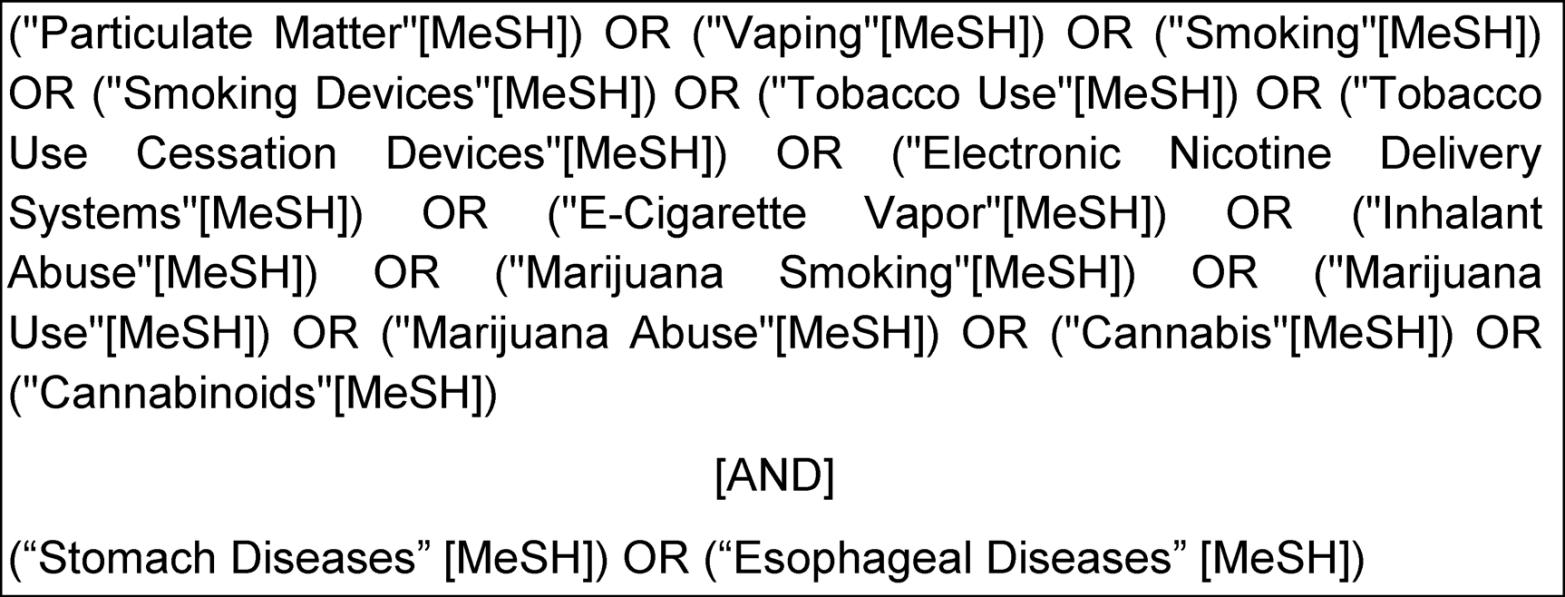


The complete listing of associated terms that were searched for with each of the above MeSH terms can be found in the MeSH Database. When searching for “stomach diseases” under its associated MeSH term, associated terms Reflux, Gastritis, Peptic Ulcer, Stomach Neoplasms, Zollinger-Ellison syndrome, etc. were included in search results. The reference-list screening was also used.

For this review, we have defined environmental exposure to include PM_2.5_, PM_10_, tobacco/cigarette smoke, vape/e-cigarette aerosols, and marijuana/cannabinoid inhalation. We have defined esophageal and gastric disease to include the following: GERD, BE, PUD, esophagitis, gastritis, ECa, and GCa.

### Study criteria

Studies were included if they (1) discussed the environmental/occupational exposure to inhalants, specifically, PM_2.5_, PM_10_, tobacco/cigarette smoke, marijuana smoke, and/or vape/e-cigarette vapor, (2) evaluated effects of exposures on esophageal or gastric diseases, (3) performed on adult human subjects, (5) were written in English, and (6) were published within the last 10 years. Studies were excluded if they (1) were not original research, (2) consisted of translational research, (3) were case reports or series, (4) were conference abstracts, or (5) were conducted on a pediatric population.

### Data extraction

Articles were reviewed and data regarding study design, patient characteristics, sample size, exposures, and outcomes were extracted. Results from database searches were filtered for full-text articles, human subjects, English language, and publication date and imported into Endnote X9. Original research papers were reviewed (title, abstract, and full text) to ascertain eligibility. We examined references cited in the relevant articles. All results were screened by Daniel Hyun Kim, Aida Fallah Zadeh, Tara Mahmoodi, and Sanjiti Podury and further independently evaluated by Anna Nolan. Disagreements were resolved by consensus.

### Risk of bias (RoB) assessment

Systematic review inherent biases (selection, detection, performance, and reporting) were addressed through the study design/search algorithm. Selection bias was addressed by having pre-determined inclusion criteria, exclusion criteria, and distinct definitions. Detection and performance bias were addressed by having at least two rounds of screening individually performed by Daniel Hyun Kim and Sanjiti Podury. Reporting bias was minimized by using PubMed and WoS search filters for peer-reviewed published articles of human subjects written in English and removing duplicates.

The Newcastle-Ottawa Scale (NOS), a domain-based approach was used to assess the degree of bias^32,33^. Scales adapted for case-control and cross-sectional studies were used. Total scores obtained by the scale were converted to Agency for Healthcare Research and Quality (AHRQ) standards or as done in previous studies to reflect the quality of each paper: low-risk studies were concordant in all domains (green); studies with at least one unclear or high-risk domain were considered as unclear or high risk of bias studies (yellow or red), respectively^34–37^. Briefly, cohort studies were assessed for three key domains of interest: (1) Assessment of Outcomes. (2) Comparability and (3) Selection. Case Control Studies included were assessed for three key domains of interest (1) Selection (2) Comparability and (3) Exposure. Finally, Cross-Sectional Studies were assessed for the key domains of (1) Selection (2) Comparability and (3) Outcome, Supplemental Table 7 A-C (details for each of these criteria may be found in the footnote of each table).

### Meta-analysis

Meta-analysis was performed (MetaAnalysisOnline.com). The platform supports various models for data analysis, including fixed-effects and random-effects models, depending on the heterogeneity of the included studies. For each study, adjusted odds ratio (aOR) and 95% confidence intervals (CI) were extracted. If a study had no aORs, it was excluded from the meta-analysis.

Heterogeneity across studies was assessed using the I² statistic to determine the appropriate model for analysis and the random-effects model was applied to account for variability between studies. A forest plot was generated for each outcome, to visually represent the individual study effect sizes and the overall pooled effect. Sensitivity analysis was also conducted to assess the strength of the findings by evaluating the impact of each study on the overall effect size. The effect of studies with high weight (%) and with a large effect size were studied.

### Ethics approval

This study does not require ethics approval as it involves a review of publicly available research and utilized anonymized original data.

## Results

### Literature search

**Fig. 1:**
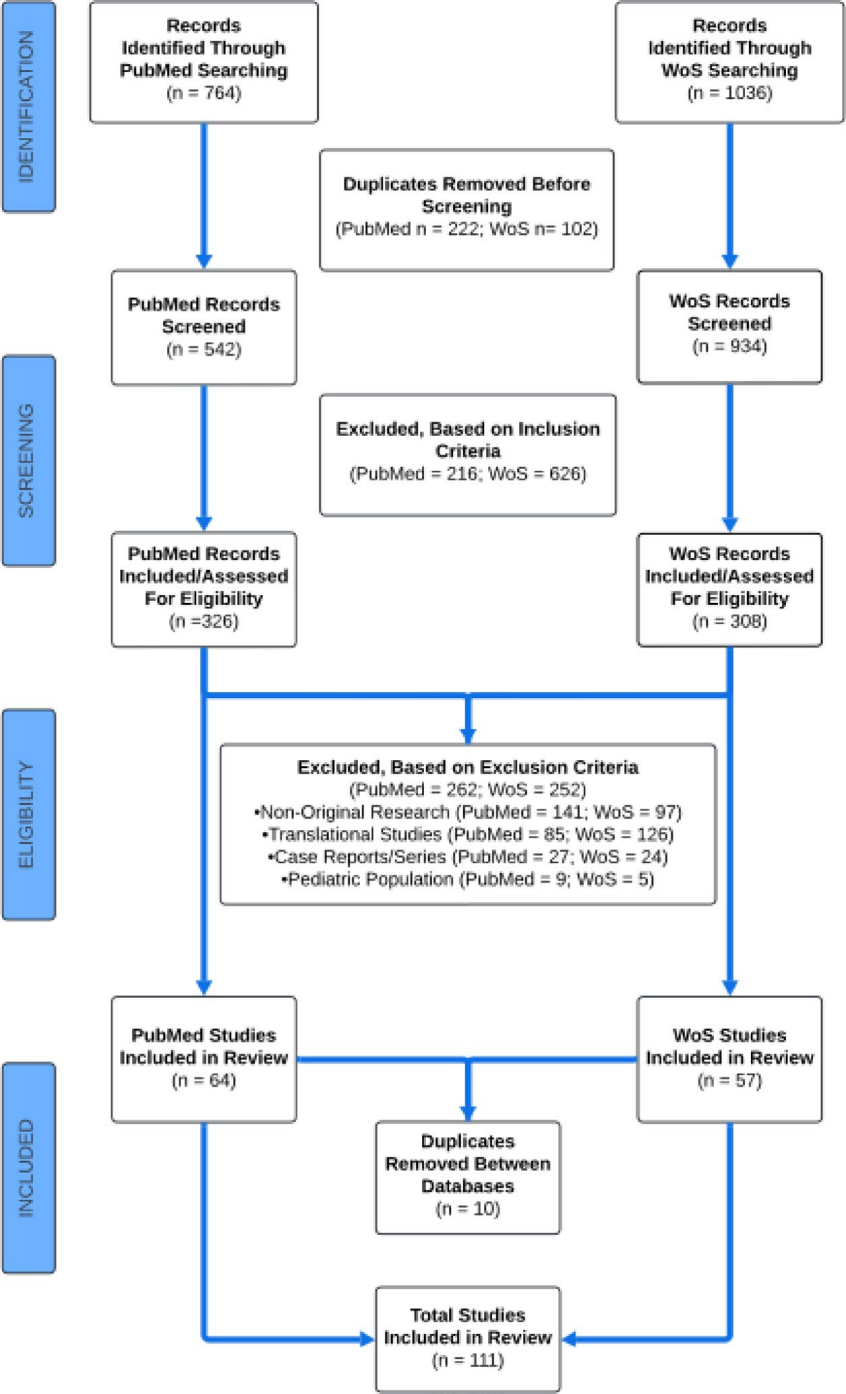
Study design per preferred reporting items for systematic reviews and meta-analyses (PRISMA) guidelines. PRISMA is an evidence-based minimum set of items for reporting in systematic reviews and meta-analyses^30,31^.

Our PubMed and WoS searches identified *N* = 764 and *N* = 1,036 studies, respectively, Fig. 1. After the removal of 222 and 102 duplicates from our PubMed and WoS searches, respectively, 542 PubMed articles and 934 WoS articles were screened. Following the application of inclusion criteria, 216 articles from PubMed and 626 articles from WoS were excluded and 326 PubMed articles and 308 WoS articles were assessed for eligibility based on exclusion criteria. Application of exclusion criteria involved the removal of 238 (141 from PubMed, 97 from WoS) non-original research articles, 211 (85 from PubMed, 126 from WoS) translational studies, 51 (27 from PubMed, 24 from WoS) case reports/series, and 14 (9 from PubMed, 5 from WoS) pediatric studies for a total of 514 (262 from PubMed, 252 from WoS) articles. 64 original PubMed and 57 original WoS research articles were considered eligible. After the removal of 10 duplicates between the two database searches, *N* = 111 studies were included in this review, Table 1. Data from screening and extraction are available, Supplemental Tables 1–6.

RoB using NOS was assessed in cohort (*N* = 39), case-control (*N* = 39), and cross-sectional studies (*N* = 29), Supplemental Table 7. Two case-crossover studies, an ecological study, and a time-series study were unable to be assessed for RoB as the NOS and our adaptations did not cover these types of studies. Scores obtained from the NOS were adapted as in previously published studies to reflect the quality of each paper^34^. Cutoffs for each risk of bias assessment depending on article type can be found within the footnote of Supplemental Table 7. Among cohort studies, *N* = 33 articles were of good quality, *N* = 1 of fair quality, and *N* = 5 of poor quality. Among case-control studies, *N* = 22 were of good.

**Table 1:** Study characteristics (*N* = 111).

Study		Country	Exposure/ design	Study size/ time period	Outcome of interest	Relevant findings
PubMed (*N* = 64)						
Smoking exposures						
1	Almadi^38^	Saudi Arabia	Smoking cohort study	4 shopping centers, Riyadh / *N* = 1265 Dec. 2012–Jan. 2013	GERD	Higher prevalence of GERD in smokers (51.63% vs. 44.41%), but not significant (*p* = 0.09) No significant association between GERD and smoking (OR: 1.34; 95% CI 0.95–1.87)
2*	Baroudi^39^	Tunisia	Smoking case-control study	Salah Azaiez Insititue of Oncology *N* = 348 2009–2010	GCa	Smoking more than 10 cigarettes a day is significantly associated with an increased risk in gastric cancer (OR: 3.66; 95% CI 1.82–7.78).
3	Begovic^40^	North Macedonia	Smoking cohort study	2 University Clinics / *N* = 67 2014	PUD	Smoking is an important risk factor, and more than half of ulcer patients were smokers (65.75%). Percent difference in relation to those who are non-smokers is statistically significant (*p* = 0.0000).
4*	Chuang^41^	Taiwan	Smoking cross-sectional study	4 hospitals in SW Taiwan / *N* = 8135 Apr. 2008–Dec. 2013	E / BE / ECa / PUD / GCa	Current tobacco use was a significant risk factor for RE (aOR: 1.26; 95% CI 1.09–1.46), BE (aOR: 1.47; 95% CI 1.08-2.00) and PUD (1.79; 95% CI 1.52–2.10), but nonsignificant for ESCC (aOR: 1.40; 95% CI 0.57–3.43) and GC (aOR: 1.24; 95% CI 0.53–2.91). Higher cumulative amounts of tobacco use were at higher risk for PUD (aOR: 1.92; 95% CI 1.60–2.31)
5	Crews^42^	USA	Smoking cohort study	Olmsted County, MN *N* = 205 Apr. 2011–Oct. 2013	E / BE	In a univariate analysis, ever tobacco use was not a significant risk factor for EE/BE (OR: 0.9; 95% CI 0.5–1.9)
6*	Dore^43^	Italy	Smoking cohort study	Sassari, Sardinia / *N* = 5156 Jan. 2002–Dec. 2013	BE / GERD	Adjusted ORs of BE and GERD for current smokers were 0.447 (95% CI; 0.199–1.002) and 1.392 (95% CI 1.085–1.787), respectively.
7	Filiberti^44^	Italy	Smoking case-control study	12 endoscopic units *N* = 1420 Mar. 2009–Oct. 2012	RE/BE	Associations shown between smoking and BE that was independent of intensity, age at initiation, GERD and dependent of duration and years since cessation Among current smokers who smoke > 18 cigarettes/day, ORs for RE and BE were 1.31 (95% CI 0.80–2.17) and 1.86 (95% CI 0.98–3.16), respectively. Risk of BE significantly increased for those who had smoked for > 32 years (OR: 2.44; 95% CI; 1.33–4.45) and those whom < 9 years have passed since quitting (OR: 2.11; 95% CI 1.19–3.72)
8	Ghoshal^45^	India	Smoking cross-sectional study	Uttar Pradesh, Jaunpur District / *N* = 2876	GERD	On univariate analysis, tobacco smoking (105 [35.2%] vs. 672 [27.1%]) was associated with GERD. On multivariate analysis, tobacco smoking (OR: 1.36; 95% CI 0.99–1.88) was associated with GERD
9	Jayalekshmi^46^	India	Smoking cohort study	Karunagappally Cohort, Kerala / *N* = 65,553 men 1990–2009	GCa	Bidi smoking was significantly associated with GCa risk (RR: 1.6; 95% CI 1.0-2.5; *P* = 0.042), but cigarette smoking was not (RR: 0.8; 95% CI 0.5–1.2) Bidi smoking increased risk of GCa among never cigarette smokers (RR: 2.2, 95% CI 1.3-4.0) GCa risk increased with the number of bidis smoked daily (*P* = 0.012) and with duration of bidi smoking (*P* = 0.036)
10	Jayalekshmi^47^	India	Smoking Cohort Study	Karunagappally Cohort, Kerala / *N* = 65,528 men Jan. 1990–Dec. 2013	ECa	RRs for current bidi and cigarette smokers were 1.4 (95% CI 0.98–2.12) and 1.3 (95% CI 0.9–1.8), respectively. Higher risks for ESCC observed for current bidi smokers (RR: 2.2; 95% CI 1.3–3.8) and cigarette smokers (RR: 1.6; 95% CI 1.0-2.5)
11	Kayamba^48^	Zambia	Smoking case-control study	University Teaching Hospital, Lusaka / *N* = 100 Oct. 2013–May 2014	ECa (ESCC)	Ever smokers showed greater risk of developing ESCC (OR: 8.0; 95% CI 2.8–22.7) Much greater proportion of cases than controls (38% vs. 0%) were current smokers (*p* < 0.000)
12	Kim^49^	South Korea	Smoking cohort study	Kangbuk Samsung Hospital, Seoul / *N* = 199,235 Jan. 2011–Dec. 2017	GCa	For current smokers, the multivariable-adjusted HR for men and women were 1.51 (95% CI 1.41–1.61) and 0.94 (0.73–1.22), respectively.
13	Kim^50^	South Korea	Smoking cohort study	*N* = 2368 Mar. 2013–Dec. 2015	GERD	Former smokers showed a significantly greater risk of GERD (OR: 1.93; 95% CI 1.12–3.35). Current smokers showed a non-significantly greater risk of GERD (OR: 2.31; 95% CI 0.94–5.66).
14*	Kim^51^	South Korea	Smoking cross-sectional study	Ewha Womans University Mokdong Hospital / *N* = 10,158 Jan. 2015–Dec. 2016	RE / GERD	Among men, smokers yielded ORs for RE and GERD of 1.67 (95% CI 1.30–2.16) and 1.48 (95% CI 0.85–2.57), respectively. Among women, smokers yielded ORs for RE and GERD of 3.47 (95% CI 1.61–7.48) and 1.35 (95% CI 0.68–2.67), respectively.
15	Koca^52^	Turkey	Smoking case-control study	Erzurum Regional Training and Research Hospital, Anatolia / *N* = 408 Jan. 2008–Mar. 2014	ECa	Smoking (X^2^ = 7.629; *p* = 0.022) was significantly higher in the patient group than the control group.
16	Koutlas^53^	USA	Smoking case-control study	University of North Carolina / *N* = 340 2011–2015	EE	EE cases were less likely to have ever smoked cigarettes compared to endoscopy-based non-EE controls (23% vs. 47%, *P* < 0.001). aOR for ever-smoking was 0.36 (95% CI 0.17–0.76).
17	Lee^54^	South Korea	Smoking case-control study	Konkuk University Medical Center / *N* = 2961 Jan. 2010–Jun. 2014	EE (Asymptomatic)	Current smoking was an independent predisposing factor for asymptomatic EE (OR 1.366; 95% CI 1.068–1.748)
18	Levenstein^55^	Denmark	Smoking cohort study	Copenhagen County / *N* = 3365 1982	PUD	Age-, gender-, and socioeconomic status-adjusted associations were significant for smoking (HR: 1.8; 95% CI 1.1–2.8).
19	Lin^56^	China	Smoking case-control study	Xianyou County, Fujian Province / *N* = 1244 Mar. 2013–Jan. 2017	GCa	Current cigarette smoking status was attributed to 83% increased risk of GCa (OR: 1.83, 95% CI 1.19–2.80) Smokers with longer duration of smoking (≥ 20 years) or started at later age (≥ 20 years) had nearly twofold increased risk of GCa vs. nonsmoker (OR: 1.97; 95% CI 1.28–3.04, OR: 2.02; 95% CI 1.30–3.14, respectively).
20	Martinucci^57^	Italy	Smoking cross-sectional study	University of Pisa / *N* = 3012 Oct. 2016–May 2017	GERD	In the set of students with GERD, percentage of smokers was higher. In a multivariate analysis, smoking status showed an increased risk of GERD (OR: 1.6; 95% CI 1.25–2.05)
21	Matsuzaki^58^	Japan	Smoking case-control study	Keio University Hospital / *N* = 2608 Oct. 2012–Nov. 2013	RE / BE	Current smoking showed risks for RE and BE of OR: 1.79 (95% CI 1.23–2.60) and OR:1.37 (0.83–2.26), respectively.
22	Miftahussurur^59^	Indonesia	Smoking case-control study	Surabaya / *N* = 104 Oct. 2014-Nov. 2015	GERD	Smokers had a significantly higher risk of GERD compared to non-smokers (OR: 3.60; 95% CI 1.298–9.955)
23	Minami^60^	Japan	Smoking cohort study	Miyagi Cancer Center Hospital / *N* = 1576 Jan. 1997–Dec. 2010	GCa	Current smokers had increased risk of stomach cancer death in a multivariate adjusted model (HR: 1.25; 95% CI 0.92–1.69).
24	Mlombe^61^	Malawi	Smoking case–control study	2 tertiary teaching hospitals / *N* = 276 Jan. 2011–Feb. 2013	ECa	In unadjusted analysis, odds of developing ESCC was 11.2 times higher among smokers than non-smokers, and in adjusted analysis it was 5.4 times higher. OR: 11.2 (95% CI 5.2–24.0) and aOR: 5.4 (2.0-15.2)
25	Moses^62^	Malawi	Smoking cohort study	Kamuzu Central Hospital, Lilongwe / *N* = 172 Jun. 2009–Sept. 2012	ECa	Esophageal cancer was among the commonest cancers in the cohort (*n* = 172; 34.5%). Patients with esophageal cancer were more likely to be smokers (OR: 2.02).
26	Navab^63^	USA	Smoking cross-sectional study	Tertiary care center, PA *N* = 158 1999–2008	BE	Correlation coefficients for current and prior tobacco use were 0.73 (95% CI 0.50–1.06) and 0.92 (0.64–1.31), respectively.
27	Nguyen^64^	Vietnam	Smoking / waterpipe smoking case-control study	Bach Mai Hospital / *N* = 226 Jan. 2018–Dec. 2018	GCa	Compared to never tobacco smokers, risk of GCa significantly increased among tobacco smokers (OR: 2.95; 95% CI 1.26–6.90, *p* = 0.013) For types of tobacco, increased risk was observed in exclusively cigarette smokers (OR: 3.26; 95% CI 1.24–8.55, *p* = 0.017) and WPT smokers (OR: 2.90; 95% CI 1.05–7.97, *p* = 0.039).
28	Okamoto^65^	Japan	Smoking cross-sectional study	Ebina Medical Center, Ebina / *N* = 965 Jan. 2015-Jun. 2015	RE	Compared to never smokers, former and current smokers showed increased risk of RE (OR: 1.5; 95% CI 0.9–2.4, *p* = 0.08) and (OR: 2.4; 95% CI 1.5–3.9, *p* = 0.01), respectively.
29	Okello^66^	Uganda	Smoking case-control study	Mbarara Regional Referral Hospital / *N* = 209 Jan. 2003–Dec. 2014	ECa	In multivariate analysis, smoking was not statistically associated with ESCC. According to univariate analysis, smoking was significantly associated with ESCC (OR: 2.93; 95% CI 1.43–5.71, *p* = 0.003). On multivariate analysis OR was 1.38 (95% CI 0.41–4.67, *p* = 0.600).
30*	Pan^67^	China	Smoking cross-sectional study	Huai’an, Jiangsu Province / *N* = 11,518 Jan. 2011–Dec. 2017	ECa	Excessive smoking was associated with an increased risk of esophageal precancerous lesions (EPL) Consuming > 30 cigarettes/day was significantly associated with EPL (OR: 1.75; 95% CI 1.09–2.80). Having 40 or more pack-years of cumulative amount of smoking was also significantly associated with EPL (OR: 1.40; 95% CI; 1.03–1.89).
31	Park^68^	South Korea	Smoking cohort study	Korea National Health Insurance Database / *N* = 43,380 2002–2013	PUD	Compared to the never-never group, all other groups had significantly adjusted HRs for gastroduodenal ulcer. HR for current-current smokers was 1.379 (95% CI 1.256–1.513). Heavy smokers had the highest risk, followed by moderate and light smokers.
32	Park^69^	South Korea	Smoking cohort study	Korea National Health Insurance Database / *N* = 97,700 2003–2014	GCa	Compared to the never-never group, current smokers had higher HRs for gastric Ca. HR for current-current smokers was 1.589 (95% CI 1.355–1.864). Risk for gastric cancer was highest in heavy smokers, followed by moderate smokers.
33*	Rafiq^70^	India	Smoking/second-hand smoking case-control study	Kashmir / *N* = 2367 Sept. 2008–Jan. 2012	ECa	Among never-tobacco users, the association between ever exposure to SHS and ECa risk were (OR: 1.32; 95% CI 0.43–4.02) Non-smokers exposed to SHS had OR of 1.25 (95% CI 0.66–2.38), whereas active smokers not exposed to SHS had OR of 1.49 (95% CI 1.08–2.04).
34	Ramos^71^	Brazil	Smoking case-control study	Sao Paolo / *N* = 739 2001–2007	GCa	Former and current smokers had ORs of 2.25 (95% CI 1.53–3.31) and 2.67 (95% CI 1.72–4.13), respectively. Smoking habit was associated with increased risk in all quartiles of consumption analyzed.
35*	Sadafi^72^	Iran	Smoking cross-sectional study	Ravansar / *N* = 9631 2014–2023	GERD	The odds of GERD among current smokers were 23% higher than non-smokers (OR: 1.23; 95% CI 1.02–1.55)
36	Schmidt^73^	Germany	Smoking Case-Control Study	Southern Germany and Augsburg / *N* = 587 and 1976 2013–2017	BE	BE cases were statistically significantly more likely to smoke (32.3% vs. 46.1% nonsmokers). Male patients with BE were significantly more likely to smoke (28.2% vs. 38.3% non-smokers) 67.7% of BE cases were ever-smokers.
37	Sewram^74^	South Africa	Smoking case-control study	3 major public referral hospitals, East Cape Province / *N* = 1858 Nov. 2001–Feb. 2003	ECa	For males, ever smokers had 4-fold increased odds compared to never smokers (OR: 4.11; 95% CI 2.55–6.65) For females, ever smokers had 3.5-fold increase odd compared to non-smokers (OR: 3.45; 95% CI 2.47–4.82).
38	Simba^75^	Kenya, Tanzania, Malawi	Smoking case-control study	Eldoret, Kenya; Moshi, Tanzania; Blantyre, Malawi / *N* = 623, 1131, 870 Aug. 2013–May 2020	ECa	Ever-tobacco use was associated with increased ESCC risk in all countries: Tanzania (OR: 3.09; 95% CI 1.83–5.23), Malawi (OR: 2.45; 95% CI 1.80–3.33), and lesser in Kenya (OR: 1.37; 95% CI 0.94-2.00). Combined OR: 2.15 (95% CI 1.72–2.68) ESCC risk increased in with tobacco intensity and smoking duration. In all three countries, smoking tobacco showed increased risk of ESCC (OR: 2.28; 95% CI 1.80–2.89).
39	Song^76^	South Korea	Smoking cross-sectional study	Seoul National University Bundang Hospital / *N* = 14,598 May 2003–Feb. 2020	GCa	In the univariate analysis smoking was significantly associated with single GCa and SGMCa in all patients (OR: 0.971; 95% CI 0.694–1.359) and in EGCa and AGCa patients (OR: 1.200; 95% CI 0.899–1.602 and OR: 0.468; 95% CI 0.231–0.949, respectively) Multivariate analysis, smoking was significantly associated with single GCa and SGMCa in AGCa patients.
40	Spreafico^77^	USA / Canada	Smoking cohort study	Boston, MA and Toronto, Ontario / *N* = 564 (235; 329) 1999–2004 & 2006–2011	ECa	Smoking conferred worse overall survival in the combined Boston-Toronto Cohort with aHR of 1.22 (95% CI 1.15–1.43) for each 20 pack-year increase.
41*	Thrift^78^	USA	Smoking case-control study	Houston, TX / *N* = 1,962	GCa	Compared to never smokers, current smokers had 2-fold increased risk for gastric intestinal metaplasia (OR: 2.05; 95% CI 1.47–2.85). Among ever smokers, increasing duration and total dose were significantly associated with increased risk (*p* = 0.004 and 0.01, respectively).
42	Wang^79^	India	Smoking cross-sectional study	Trivandrum District / *N* = 1,072 2010–2011	GERD	No association between cigarette smoking and risk of GERD. For the association of ever-smokers and risk of GERD, a mutually adjusted analysis yielded OR of 0.7 (95% CI 0.4–1.2).
43*	Wang^80^	USA	Smoking cohort study	NIH-AARP Cohort / *N* = 490,605 1995–2011	ECa/GCa	For esophageal cancers, current smoking yielded HRs of 5.75 (95% CI 3.90–8.49) for ESCC and 3.16 (95% CI 2.54–3.92) for EADC. For gastric cancers, current smoking yielded HRs of 3.16 (95% CI 2.42–4.13) for GADC and 1.61 (95% CI 1.27–2.05) for GNCA.
44	Wei^81^	China	Smoking Case-Control Study	Feicheng, Shandong / *N* = 464 Jul. 2013-Apr. 2014	ECa	Ever smoking was associated with 3.11-fold increase in ESCC risk (OR: 3.11; 95% CI 1.63–6.05) For each cigarette-years increase in smoking index, ESCC risk increased by 56% (OR: 1.56; 95% CI 1.18–2.13).
45	Yang^82^	China	Smoking case-control study	Fujian Province / *N* = 423 Jan. 2010-Dec. 2016	ECa	Tobacco smoking was related to ESCC risk, but no significant difference in magnitude of its association with respect to macroscopic type of cancer. Tobacco smoking showed increased risk for ulcerative type ESCC (OR: 2.24; 95% CI 1.20–4.19) and medullary type ESCC (OR: 2.56; 95% CI 1.29–5.06).
46*	Yang ^83^	China	Smoking case-control study	Taixing / *N* = 3314 Oct. 2010–Sept. 2013	ECa	In a fully adjusted analysis, current smokers had OR of 1.12 (0.88–1.44) but not significant. Male heavy smokers (i.e., smoked more than 20 cigarettes/day or 40 pack-years or started smoking early) showed a moderately increased risk for ESCC.
47	Yates^84^	UK	Smoking cohort study	EPIC-Norfolk Cohort *N* = 24,068 1993–1997	BE/ECa	Hazard ratios for current and former smokers for BE were 1.57 (95% CI 0.83–2.96) and 1.38 (95% CI 0.88–2.16), respectively. Hazard ratios for current and formers smokers for EAC were 1.82 (95% CI 0.81–4.09) and 1.27 (95% CI 0.71–2.27), respectively. Current and former smoking were not significantly associated with BE and EAC.
48	Zacharakis^85^	Saudi Arabia	Smoking cohort study	Al-Kharj, Riyadh / *N* = 1080 Jan. 2017-May 2023	GCa	Current and former smoking yielded ORs of 4.00 (95% CI 2.05–7.81) and 0.79 (95%. CI 0.28–2.24), respectively. Only current smoking was a significant risk factor for GCa (*P* = 0.002)
49*	Zhao^86^	China	Smoking case-control study	4 counties Jiangsu Province / *N* = 18,093 Jan. 2003–Dec. 2010	ECa/GCa	Tobacco smoking was associated positively with both esophageal (aOR: 1.68; 95% CI 1.50–1.87) and stomach cancer (aOR: 1.61; 95% CI 1.43–1.81). There was a significant does-response relationship between pack-years of smoking and risks of esophageal (*P* < 0.001) and stomach cancer (*P* < 0.001).
Smoking and waterpipe exposures						
50	Etemadi^87^	Iran	Smoking /waterpipe smoking cohort study	Valashahr, Fars / *N* = 9264 2012–2017	GERD	Strongest associations of waterpipe smoking were with ‘severe and frequent reflux’ (OR: 1.30; 95% CI 1.08–1.56) Former use had a stronger association with ‘severe reflux’ and (OR: 1.29; 95% CI 1.06–1.56) and current use with ‘frequent reflux’ (OR: 1.18; 95% CI 1.03–1.36). Current cigarette use was a significant risk factor for “any reflux” among men (OR: 1.20; 95% CI 1.02–1.40) Increases in reflux prevalence associated with waterpipe use duration and intensity.
51	Lai^88^	Vietnam	Smoking /waterpipe smoking case-control study	3 major hospitals, Hanoi / *N* = 1082 Feb. 2003–Apr. 2011	GCa	WPT smoking was positively associated with GCa risk. Significantly high GCa risk in current WPT smokers (OR: 1.8; 95% CI 1.3–2.4) Current cigarette smoking was not a significant risk factor for GCa (OR: 1.1; 95% CI 0.8–1.4) No significant interaction between effects of WPT and cigarette smoking on GCa risk.
52	Le^89^	Vietnam	Smoking / waterpipe smoking cohort study	3 Northern Vietnam Provinces / *N* = 25,619 2008–2019	GCa	Significantly higher GCa mortality among ever-smokers than never-smokers (aHR: 2.43; 95% CI 1.35–4.36) Exclusive WPT smokers showed the highest risk (HR: 3.22; 95% CI 1.67–6.21), followed by smokers of both WPT and cigarette (HR: 1.99; 95% CI 0.89–4.63), then exclusive cigarette smokers (HR: 1.90; 95% CI 0.88–4.07).
Smoking and PM exposures						
53*	Sun^90^	China	Smoking / PM_2.5_ cohort study	China Kadoorie Biobank / *N* = 510,125 2005–2017	ECa	A linear concentration-response relationship between long-term PM_2.5_ exposure and ECa. Each 10-µg/m^3^ increase in PM_2.5_, the HR for ECa was 1.16 (95% CI1.04–1.30) Using lowest group of PM_2.5_ exposure as reference, HRs for other quartile groups, from low to high, were 1.09 (95% CI 0.86–1.37), 1.28 (95% CI 0.98–1.66), and 1.32 (95% CI 1.01–1.72). Subgroup analyses showed ever smoking had an HR of 1.18 (95% CI 1.04–1.35).
54	Wong^91^	China	Smoking / PM_2.5_ cross-sectional study	Hong Kong / *N* = 66,820 Jul. 1998–Dec. 2001	PUD	Adjusted HR for PUD hospitalization per 10 µg/m^3^ of PM_2.5_ was 1.18 (95% CI 1.02–1.36). Associations with PM_2.5_ were significant for gastric ulcers (HR: 1.29; 95% CI 1.09–1.53) but not for duodenal ulcers (HR: 0.98; 95% CI 0.78–1.22) For other variables, current smokers were to have significantly increased risk for hospitalization of PUD (HR: 1.59; 95% CI 1.37–1.84).
PM exposures						
55	Li^92^	China	PM_2.5_ Cross-sectional population study	388 cancer registry institutes, Mainland China 2007–2015	ECa	Significantly positive association between PM_2.5_ and EC incidence. Lag effect of 4 years showed the greatest risk of 1.32% (95% CI 1.20–1.45%) and 2.70% (95% CI 2.49–2.92%), respectively.
56	Li^93^	China	PM_2.5_ cross-sectional population study	213 Prefectural Level Cities, Mainland China 2000–2015	ECa	Stronger association between PM_2.5_ and incidence observed in low urbanization groups, and association was stronger for females than males.
57	Li^94^	China	PM_2.5_ cross-sectional study	Jiangsu Province / *N* = 524,019 2015–2020	ECa/GCa	Long-term exposure to black carbon, organic carbon, nitrate, and ammonium was significantly associated with esophageal and stomach cancer. Sulfate exposure was significantly associated with stomach cancer.
58	Lin*^95^	Taiwan	PM_2.5_ cross-sectional population study	Entire population of Taiwan / *N* = 23.57 million 2010–2017	ECa	Due to linear regression analysis, the average number of deaths from esophagus cancer decreases 0.17 (95% CI -0.22, -0.12) per 100,000 people with increasing average PM_2.5_ concentration.
59	Quan^96^	Canada	PM_2.5_, PM_10_ case-crossover study	Calgary (Discovery) and Edmonton (Replication) / *N* = 1374 and 1159 2004–2010	PUD	When air pollution exposures were assessed as 3-, 5-, and 7- day averages, pollutants were inversely associated with UGIB in the discovery cohort. 5-day averages of PM_2.5_ and PM_10_ had ORs of 0.75 (95% CI 0.61–0.90) and 0.87 (95% CI 0.75-1.00), respectively.
60	Rao^97^	China	PM_2.5_ cross-sectional study	Fujian Province / *N* = 5479 Jan. 2016-Dec. 2016	ECa	Spatial distribution of hospitalization rate of ECa in 2016 was not consistent with that of concentration of PM_2.5_ in same year. Concentration of PM_2.5_ in 2003 and 2004 had strongest correlation with hospitalization rate of ECa in 2016, with Pearson correlation coefficient r value of -0.365.
61	Seo^98^	South Korea	PM_2.5_, PM_10_ cross-sectional study	Korea National Health Insurance Database / *N* = 200,000 2002–2017	GERD	The final model of the study significantly predicted GERD-related medical utilization. PM_2.5_ and CO were identified as risk factors for GERD.
62	Tsai^99^	Taiwan	PM_2.5_, PM_10_ case-crossover study	Taipei / *N* = 23,205 2009–2013	PUD	Increases in both PM_2.5_ (OR: 1.14; 95% CI 1.09–1.18) and PM_10_ (OR: 1.05; 95% CI 1.01–1.08) were significantly associated with increased risk of hospital admissions on warm days. On cool days, only increases in PM_10_ were found to be significantly associated with increased risk of hospital admission (OR: 1.04; 95% CI 1.02–1.07).
63	Wu^100^	China	PM_2.5_ / PM_10_ ecological study	Yinzhou District, Ningbo City, Zhejiang Province / *N* = 204,257 Jan. 2017-Dec. 2019	PUD	Cumulative risk ratios for PM_2.5_ and PM_10_ showed nearly linear adverse effect and gently grew to maximums of 2.40 (95% CI 1.36–4.24) and 1.65 (95% CI 0.98–2.76), respectively. Significant associations for both men and women were only observed for PM_2.5_.
64	Yu^101^	China	PM_2.5_ cross-sectional study	Zhejiang Province / *N* = 647,092 Jan. 2014-Dec. 2018	PUD	A potential dose-response relationship was observed between quartile concentrations of PM_2.5_ 1 month before gastroscopy and detection of PUD. Subjects in the highest quartile of PM_2.5_ exposure displayed significantly higher risk (OR: 1.178; 95% CI 1.118–1.242). The overall estimated OR for the detection of PUDs associated with a 10 µg/m^3^ increase in PM_2.5_ was 1.050 (95% CI 1.038–1.063)
Web of science (*N* = 47 unique manuscripts and *N* = 13 overlap with PubMed*)						
Smoking exposures						
65	Ahmed^102^	Pakistan	Smoking cross-sectional study	Darul Sehat Hospital, Zubaida Medical Center, Liaquat National Hospital/ *N* = 2000 Jan 2018–Oct 2018	GERD	Results assessing association of various lifestyle factors with GERD showed that those with habits of smoking were at high risk of GERD with aOR of 6.25 (95% CI 4.40–8.91).
66	Al-Towairqi^103^	Saudi Arabia	Smoking cross-sectional study	Taif University, Taif City/ *N* = 240 Jan 2019–Apr 2020	GERD	Results showed that smoking was an insignificant risk factor for GERD (*p* = 0.398) with a prevalence of 35.71% among smokers.
67	Alcala^104^	Iran	Smoking cohort study	Golestan Province/ *N* = 50,045 2004–2008	ECa	Opium and cigarette smoking yielded a population attributable fraction of 13% among the general population and 15% among males for the risk of esophageal cancer.•
68	Alrashed^105^	Saudi Arabia	Smoking cross-sectional study	Shaqra University, Shaqra City/ *N* = 435 2018–2019	GERD	Smoking showed statistical significance and association (*p* < 0.05) with symptomatic GERD. Among those who were current smokers, 37.3% had GERD.
69	Arroyo-Martinez^106^	Spain	Smoking cohort study	Unnamed Spanish Health District/ *N* = 430 1996–2011	BE/ECa	Cigarette smoking showed no significant association with any forms of BE progression and EAC. Cigarette smoking showed the highest association in the progression of BE w/o dysplasia to EAC (*p* = 0.170)
70	Asombang^107^	Zambia	Smoking case-control study	University Teach Hospital, Lusaka/ *N* = 72 Nov 2010–Jan 2012	ECa	Smoking was found to be significantly associated with ECa with an aOR of 11.24 (95% CI 1.37–92.40; *p* = 0.024)
71	Chen^108^	China	Smoking cohort study	Jiangsu, Anhui, Shandong, Henan Provinces/ *N* = 86,745 2007–2015	ECa	Smoking less than 30 pack-years showed an aOR of 1.58 (95% CI 1.14–2.18), whereas smoking greater than or equal to 30 pack-years showed an aOR of 2.08 (95% CI 1.48–2.92).
72	Chen^109^	China	Smoking cross-sectional study	Wuwei Municipality/ *N* = 9326	GCa	The present study did not find significant association between CAG and smoking. Smoking did show to significantly increase the risk of progression from CAG to IM with an aOR of 1.26 (95% CI 1.07–1.43)
73	Chen^110^	China	Smoking cohort study	China Kadoorie Biobank/ *N* = 512,891 2004–2008	ECa/GCa	In men, smoking was found to have a significant association with ECa and GCa with aRRs of 1.47 (95% CI 1.25–1.73) and 1.34 (95% CI 1.16–1.55), respectively. In women, there was no significant association with aRRs for ECa and GCa of 1.24 (95% CI 0.71–2.17) and 1.19 (95% CI 0.81–1.75), respectively.
74	Dighe^111^	USA	Smoking cohort study	Roswell Park Comprehensive Cancer Center, Buffalo, NY/ *N* = 371 Jan 2003–Sept 2019	ECa	Smoking history was found to be significantly associated with survival of esophageal cancer of stages I, II, and III with current smoking having an HR of 2.54 (95% CI 1.42–4.53; *p* = 0.002)
75	Etemadi^112^	Iran	Smoking cohort study	Golestan Province/ *N* = 50,045 Jan 2004–June 2008	ECa	Among tobacco users, metabolites of styrene and xylene were associated with ESCC. In addition, among tobacco users, 2 tobacco-specific nitrosamines (NNN and N’-Nitrosoanatabine) were also associated with ESCC.
76	Fang^113^	China	Smoking case-control study	Affiliated Drum Tower Hospital, Nanjing/ *N* = 3176 Jan 2005–Dec 2012	GCa	In a univariate analysis, tobacco abuse was found to be significantly associated with distal gastric carcinoma (OR: 1.47; 95% CI 1.01–2.14) but not proximal gastric carcinoma (OR: 1.42; 95% CI 0.89–2.26). In a multivariate analysis, tobacco abuse was found to be significantly associated with neither distal gastric carcinoma (OR: 1.33; 95% CI 0.74–2.40) nor proximal gastric carcinoma (OR: 0.80; 95% CI 0.37–1.75).
77	Flores-Luna^114^	Mexico, Paraguay, Columbia	Smoking case-control study	Mexico (*N* = 559), Colombia (*N* = 461), Paraguay (*N* = 202)/ *N* = 1222 Oct 1999–Jul 2002	GCa	Ever smoking was found to be not significantly associated with preneoplastic lesions nor gastric cancer with aORs of 1.3 (95% CI 1.0-1.8) and 1.3 (95% CI 0.9-2.0), respectively.
78	Gado^115^	Egypt	Smoking case-control study	Bolak Eldakror Hospital, Giza/ *N* = 433 Jan 2000–Jan 2013	RE	On a univariate analysis, current smoking was significantly associated with RE with an OR of 1.99 (95% CI 1.3–3.1) On a multivariate analysis, current smoking was not significantly associated with RE with an OR of 1.49 (95% CI 0.8–2.7).
79	Ghanadi^116^	Iran	Smoking case-control study	Khorramabad City/ *N* = 60 2015	PUD	On a multivariate analysis, regular smoking history was significantly associated with PUD with an aOR of 4.75 (95% CI 1.61–8.2).
80	Ghosh^117^	India	Smoking case-control study	Chittaranjan National Cancer Institute, West Bengal/ *N* = 751 2014–2018	GCa	Tobacco intake in the form of smoking was found as an important risk factor in gastric cancer development with risk ratio and odds ratio of 1.18 and 3.14, respectively.
81	Guo^118^	China	Smoking cross-sectional study	Henan Province/ *N* = 43423Form Oct 2013–Oct 2017	ECa/GCa	Current smoking was not significantly associated with any esophageal and gastric neoplasms with aORs of 1.35 (95% CI 0.92–1.98) and 0.55 (95% CI 0.23–1.34), respectively. Former smoking was significantly associated with any esophageal neoplasms and not significantly associated with any gastric neoplasms with aORs of 2.11 (95% CI 1.33–3.35) and 0.74 (95% CI 0.24–2.30), respectively.
82	Hazarika^119^	India	Smoking case-control study	Adichunchanagiri Hospital, Mandya/ *N* = 50	GCa	Tobacco smoking in the form of cigarette and bidi smoking was found to be a risk factor for gastric carcinoma as it was seen in 22 (44%) patients all being males.
83	Jideh^120^	Australia	Smoking case-control study	Nepean Hospital, Sydney/ *N* = 6962 Jun 2010–Mar 2015	ECa	Among patients that had esophageal squamous papillomas (ESPs), 44% of them had a history of cigarette smoking.
84	Kaimila^121^	Malawi	Smoking case-control study	Kamuzu Central Hospital, Lilongwe/ *N* = 300 Aug 2017–Apr 2020	ECa	Differential mortality by ESCC was not noted among smokers and nonsmokers yielding a crude HR of 1.06 (95% CI 0.81–1.38).
85	Kang^122^	Korea	Smoking cohort study	Samsung Medical Center, Korea/ *N* = 5765 Jan 2006–Dec 2008	EE	Univariate analysis revealed smoking was significantly associated with an increased risk of developing RE, with former smokers showing a aRR of 1.95 (95% CI; 1.64–2.31) and current smoking showing a higher aRR of 2.70 (95% CI 2.26–3.23) compared to never smokers.
86	Kim^123^	South Korea	Smoking cohort study	Asan Medical Center/ *N* = 308 May 2010–Apr 2012	ECa	Multivariate analysis showed that smoking (aOR: 8.317; 95% CI 0.940-73.583) increased the risk of developing synchronous esophageal squamous cell neoplasm.
87	Kim^124^	South Korea	Smoking cohort study	Ewha Woman University Mokdong Hospital/ *N* = 253 Sep 2006– Apr 2010	RE	Multiple logistic regression analyses revealed that smoking pack-years (aOR: 1.015: 95% CI 1.004–1.025) was independent factor associated with RE in COPD.
88	Kumar^125^	USA	Smoking cohort study	Veterans Health Administration, USA/ *N* = 371,813 Jan 1994-Dec 2018	GCa	Smoking was significantly associated with increased risk of GCa (aSHR: 1.38, 95% CI 1.25–1.52).
89	Kunzmann^126^	UK	Smoking cohort study	UK Biobank/ *N* = 355,034 2006–2010	ECa	Former smokers were associated with an increased risk of Eca with aOR of 2.03 (95% CI 1.47–2.80) Current smokers demonstrated an even higher risk of ECa with aOR of3.83 (95% CI 2.59–5.66)
90	Laaksonen^127^	Australia	Smoking cohort study	Australian Cancer-PAF Cohort Consortium/ *N* = 365,052	ECa/GCa	Former and current smoking were found to be significantly linked to all esophageal cancers avoidable by change in exposure to behavioral risk factors with aHRs of 1.75 (95% CI 1.16–2.64) and 3.27 (95% CI 1.84–5.80), respectively. Former and current smoking were found to be significantly linked to stomach cardia cancers avoidable by change in exposure to behavioral risk factors with aHRs of 1.73 (95% CI 1.19–2.50) and 1.96 (95% CI 1.07–3.59), respectively.
91	Li^128^	China	Smoking cohort study	Qibao, Minhang District, Shanghai/ *N* = 23,415 2008–2011	GCa	Environmental tobacco smoke (ETS) was significantly associated with an increased risk of GCa in all participants (aHR: 1.86, 95% CI 1.21–2.85). The risk was further elevated among individuals exposed to both active smoking and ETS, revealing a stronger joint effect (aHR: 2.07,95% CI 1.14–3.74).
92	Lim^129^	South Korea	Smoking cohort study	Gangnam Center, eoul National university Hospital, South Korea/ *N* = 297 Jan 2004- May 2016	GCa	After adjustment, smoking did not independently contribute to risk of GCa with an aOR of 0.66 (95% CI 0.25–1.72).
93	Liu^130^	China	Smoking case-control study	You’an Hospital, Beijing/ case: 420 control: 409 Jan. 2011- Aug 2021	RE	In multivariate analysis smoking was a risk factor for RE among liver cirrhosis patients (aOR: 2.41:95% CI 1.43–4.06).
94	Lu^131^	China	Smoking cross-sectional study	People’s Hospital of Feicheng/ *N* = 5476	ECa	Ever smoking yielded aORs of 1.26 (95% CI 1.00-1.578) Smoking > 20 pack-years was found to be significantly associated with ESCC with aOR of 1.48 (1.11–1.98)
95	Meyers^132^	USA	Smoking Population-bBased case-control study	Los Angeles county, California, USA/ Case: Lung Cancer: 611- UADT Cancer: 601- Control: 1040 1999–2004	ECa	Cumulative tar exposure was significantly associated with Eadenocarcinoma, particularly in the second tertile (aOR: 2.52; 95% CI 1.21–5.25). Broader associations with UADT cancers, including ECa, were observed, with each 1 IQR increase in tar exposure linked to an aOR of 1.46 (95% CI 1.24–1.73).
96	Ness-Jensen^133^	Norway	Smoking cross-sectional study	Tromsø municipality, Norway/ *N* = 21,083 1974–2016	GERD	The risk of GRD over time was consistently higher with overweight and current daily tobacco smoking with an aOR of 1.14 (95% CI 1.01–2.29).
97	Ohashi^134^	Japan	Smoking cross-sectional study	Kyoto University Hospital/ *N* = 433	RE	The Brinkman Index, a measure of cumulative smoking exposure, was significantly associated with an increased risk of the condition with an aOR: 1.94 (95% CI 1.56–2.42).
98	Pan^135^	China	Smoking case-control study	Huai; an District Jingsu Province, China/ Case: 200 Control: 200 2007–2017	ECa	Smoking was significantly associated with an increased risk of ECa with an aOR of 3.11 (95% CI 1-9.63).
99	Poosari^136^	Thailand	Smoking case-control study	Srinagarind Hospital, Khon Kaen, Thailand/ case: 105 control: 105 2007–2017	ECa	Smoking was significantly associated with an increased risk of ECa with an aOR of3.5 (95% CI 1.28–9.56).
100	Pournaghi^137^	Iran	Smoking case-control study	North Khorasan, Iran/ case: 96 control: 187 2013–2015	ESCC	There was no significant association between cigarette smoking and the risk of ESCC with OR of 1.1 (95% CI0.6–2.04) However, a significant association was observed for quitting smoking for more than 10 years with OR of 9.8 (95% CI 1.1–85.4).
101	Rabiee^138^	Iran	Smoking cohort study	Mazandaran province, Iran/ *N* = 933 2014–2015	GERD	Smoking was a potential risk factor for GERD. multivariate logistic regression, a significant association was seen between frequent GERD and smoking with aOR of 3.53 (95% CI 2.17–5.74), however, there was no significant association between non-frequent GERD and smoking.
102	Sheikh^139^	Iran	Smoking Cohort study	Golestan Province, Iran/ *N* = 50,038 2004–2008	ESCC	There was a significant association between opium smoking, exposure to indoor air pollution with an increased risk of ESCC. However, smoking cigarettes was not associated with it with aHR: 1.35 (95% CI 0.09–2.02).
103	Soroush^140^	Iran	Smoking Cohort study	Rural regions of the Golestan province, Iran/ *N* = 49,559 2004–2008	GERD	There was a significant inverse association between GERD symptoms and ESCC in tobacco smokers (aHR 0.26, 95% CI 0.08–0.83) but not in non-smokers (aHR 1.09, 95% CI 0.78–1.53).
104	Wang^141^	China	Smoking cross-sectional study	Hua County of Anyang, China/ *N* = 2844 Jul 2013- Mar 2014	RE	Multivariate analysis revealed smoking and male gender as significant risk factors for RE, with smoking having an OR of 1.41 (95% CI 1.01–1.98) and male gender associated with a significantly higher risk, with an OR of 4.18 (95% CI 3.09–5.65.
105	Wang^142^	Australia	Smoking cohort study	Australia/ *N* = 20,975 1990–1994	GERD/BE	Current and former smokers had earlier onset than never smokers. There was a significant association between current smokers and daily symptom frequency of GERD with aOR of 1.13 (95% CI 0.93–1.38). former smokers, but not current smokers at baseline, had higher BE risk compared with never smokers.
106	Wang^143^	Taiwan	Smoking case-control study	Case: National Cheng Kung Hospital in Tainin, Taiwan Control: Cancer screening Cohort, Tainin, Taiwan/ case: 41 control: 123 Case: 2002–2019 Control: 2008–2013	Esophageal neuroendocrine neoplasms	Cigarette smoking was significantly associated with an increased risk of Esophageal neuroendocrine neoplasms, with an aOR of4.7 (95% CI 1.6–13.5).
107	Zhang^144^	China	Smoking nested case- control study	Urban areas of China/ Case: 215 Control;645 Sep 2012- Dec 2019	GCa	Smoking was significantly associated with increased GCa risk with an aOR of 3.06 (95% CI 1.7–5.54).
PM exposures						
108	Ethan^145^	China	PM_2.5_ time series study	Xi’an Jan 2014–Dec 2016	GCa	As a single pollutant, PM2.5 was significantly associated with stomach cancer mortality with a RR of 1.0003 (95% CI 1.0001–1.002). On a multi-pollutant analysis, PM2.5 combinations with NO2 were significantly associated with stomach cancer mortality with an RR of 1.0103 (95% CI 1.009–1.021).
109	Fan^146^	China	PM_2.5_ cross-sectional study	Jiangsu Province1.028/ *N* = 947,337 2015–2020	GCa	Each 1 µg/m3 increment in PM2.5 exposure was significantly associated with a 2.7% increase in the risk of all-site cancer mortality. Specifically, PM2.5-mortality for gastric cancer had an aRR of 1.028 (95% CI 1.011, 1.046)
110	Huang^147^	China	PM_10_ cross-sectional study	Shandong Province/ *N* = 1255 2010–2014	ECa	In correlation analyses, PM_10_ (CC: 0.51; *p* = 0.046) and NO_2_ (CC: 0.53; *p* = 0.03) both had significant linear correlations with esophageal cancer mortality rates.
111	Li^148^	China	PM_2.5_ cohort study	Jiangsu Province/ *N* = 524,019 2015–2020	ECa/GCa	In PM_2.5_-adjusted models and constituent-residual models, sulfates, ammonium, and chloride were found to be significantly associated with esophageal and gastric cancer mortality.

1–64 PubMed; 65–111 WoS; * Found in both PubMed and WoS Search

GERD, gastroesophageal reflux disease; GCa, gastric cancer; PUD, peptic ulcer disease; RE, reflux esophagitis; BE, barrett’s esophagitis; ECa, esophageal cancer; ESCC, esophageal squamous cell carcinoma; EE, erosive esophagitis; aOR, adjusted Odds ratio; aHR, adjusted Hazard ratio; aRR, adjusted relative risk; GERD, gastroesophageal reflux disease; OR, odds ratio; CI, confidence interval; RR, relative risk; HR, hazard ratio; EPL, esophageal precancerous lesions; WPT, waterpipe tobacco; SHS, second-hand smoke; EGCa, esophageal gastric cancer; SGMCa, squamous gastric cancer; GADC, gastric adenocarcinoma; GNCA, gastric non-cardia adenocarcinoma; EADC, esophageal adenocarcinoma; PM_2.5_, particulate matter less than 2.5 micrometers; PM_10_, particulate matter less than 10 micrometers; NO_2_, nitrogen dioxide; UGIB, upper gastrointestinal bleeding; ETS, environmental tobacco smoke; aSHR, adjusted sub-distribution hazard ratio; IQR, interquartile range.

Quality, *N* = 8 of fair quality, and *N* = 9 of poor quality. Among cross-sectional studies, *N* = 23 were of good quality, *N* = 4 of satisfactory quality, and *N* = 2 of unsatisfactory quality.

### Study characteristics

**Fig. 2:**
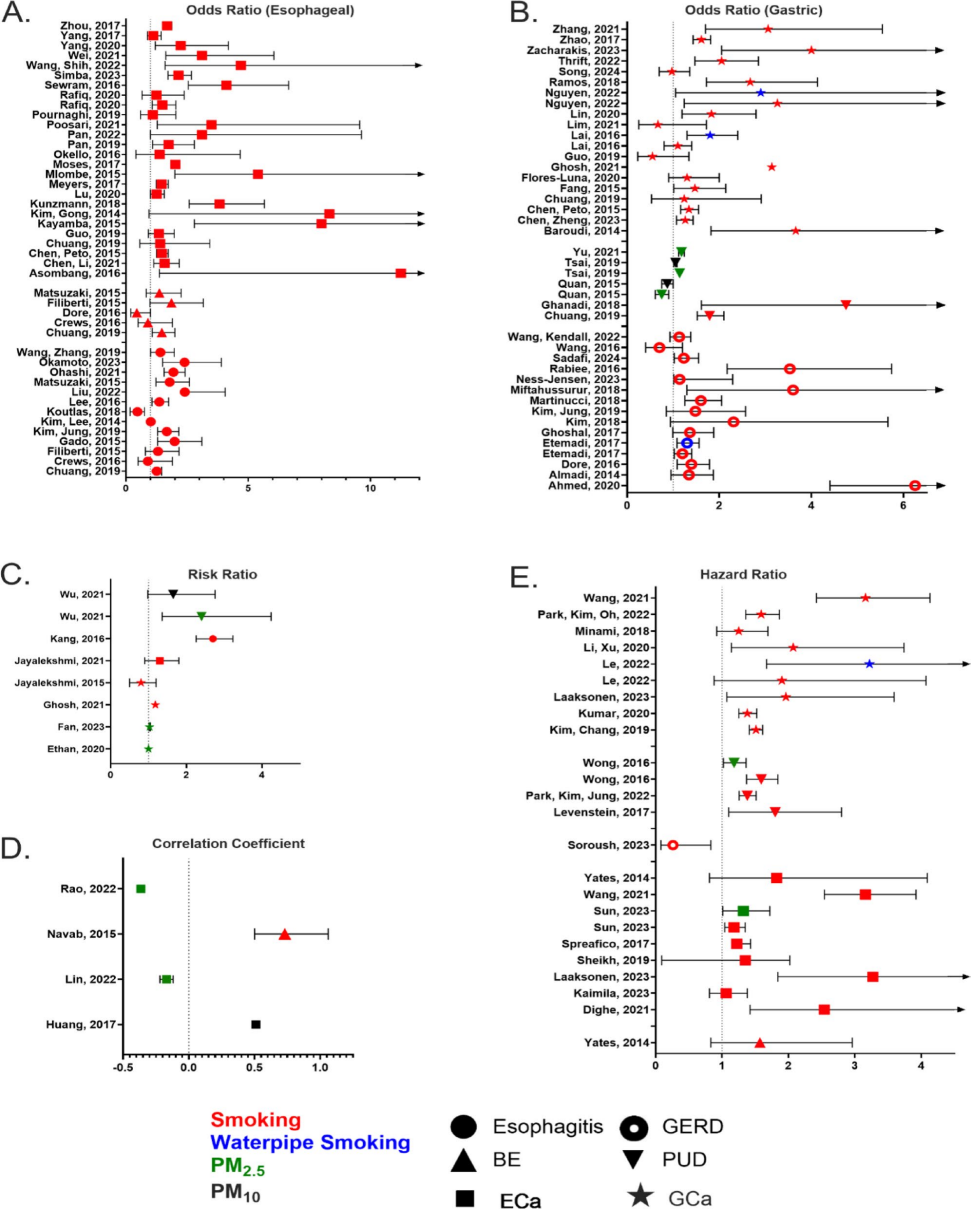
Overview of data synthesis: (**A**) Summary of odds ratios for esophageal diseases (esophagitis, BE, ECa), (**B**) Summary of odds ratios for gastric diseases (GERD, PUD, GCa), (**C**) Summary of risk ratios, (**D**) Summary of correlation coefficients, (**E**) Summary of hazard ratios.

The populations of patients with esophageal or gastric disease included those afflicted with esophagitis (*n* = 8), BE (*n* = 8), ECa (*n* = 24), GERD (*n* = 11), PUD (*n* = 9), and GCa (*n* = 16). Studies that focused on any other outcomes did not meet exclusion/inclusion criteria. The investigated exposures were smoking, waterpipe smoking, and PM_2.5_/PM_10_ exposure. While there were no studies that focused on marijuana smoking or vaping/e-cigarettes that met our inclusion/exclusion criteria we know from the literature that the use of cannabinoids and vaping are linked to the development of gastrointestinal disorders^149^. One study investigated the role of exposure to second-hand smoke, in addition to direct cigarette smoke exposure^70^. Most studies produced an odds ratio (OR), risk ratio (RR), correlation coefficient (CC), or hazard ratio (HR) to measure each of the risks associated with their respective exposures for a particular outcome, which are summarized in Fig. 2 (see raw data in Supplemental Table 8 A–E). Among those studies, some reported using adjusted models in their analyses. Additionally, other studies focused on the percent presentation of risk factors;^40,53^ risk by measuring the increase in incidence of the respective disease;^73,92–94^ the differences in mortality with respect to magnitude of exposure^95^and utilized a novel predictive model to identify risk factors, Table 1^98^.

### Esophagitis

Current tobacco use was identified as a significant risk factor for reflux esophagitis (RE)^41,44,58,65,115,122,134^. Some studies focused on specific groups of patients and found that smoking was associated with RE among COPD patients^124^ and liver cirrhosis patients^130^. When studying gender-specific differences between smoking and risk of RE, Kim et al. found that smoking led to greater risks of RE among women compared to men^51^, whereas Wang et al. found that smoking led to greater risks of RE among men^141^. Lee et al. identified smoking as a significant risk factor for asymptomatic erosive esophagitis (EE)^54^. Associations between smoking and eosinophilic esophagitis (EOE) were also investigated. One study found that those with EOE were significantly less likely to have ever smoked cigarettes compared to non-EOE controls, but smoking was not significantly associated with an increased risk of EOE^53^.

**Fig. 3:**
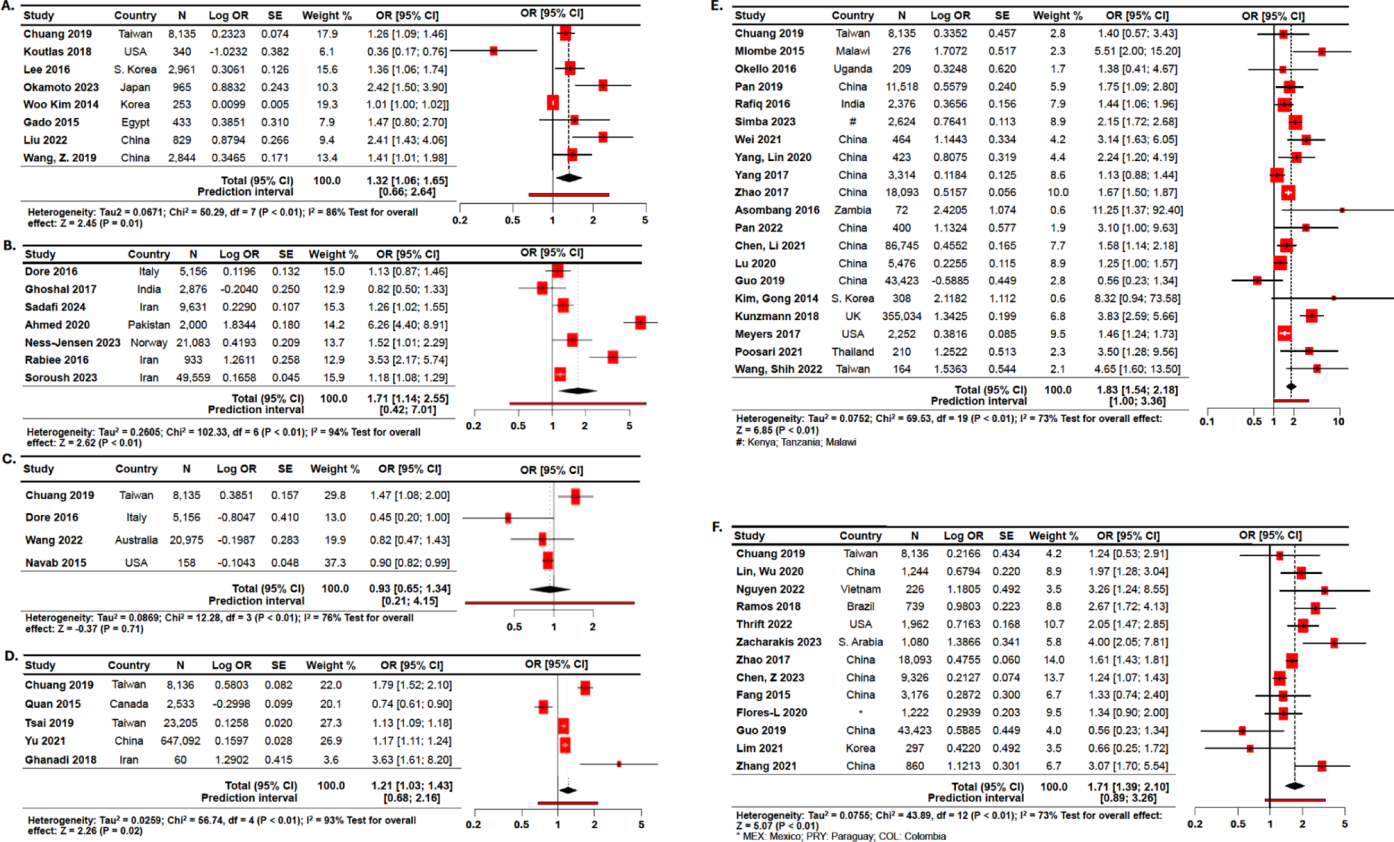
Meta-analysis of the association between inhalational exposures and upper gastrointestinal diseases: (**A**) esophagitis, (**B**) GERD, (**C**) BE, (**D**) PUD, (**E**) ECa, (**F**) GCa.

Our meta-analysis of 8 studies^41,53,54,65,115,124,130,141^ revealed that inhalational exposures were significantly associated with an increased risk of esophagitis with a pooled estimate of 1.32 (95% CI 1.06–1.65; I^2^ = 86%), Fig. 3A. In our sensitivity analysis, we excluded one study^53^ that had high heterogeneity; however, the analysis results revealed no significant differences with a pooled estimate of 1.43 (95% CI 1.15–1.78; I^2^ = 86%), as shown in Supplementary Fig. 1 (1.3A vs 1.3A’).

### Gastroesophageal reflux disease

Multiple studies identified smoking as a risk factor for GERD^43,45,57,59,72,102,105^. Kim et al. interestingly found that former smoking was significantly associated with risk of GERD, while current smoking was not significantly associated^50^. When investigating gender-specific differences in the effects of smoking on the risk of GERD, Kim et al. found that smoking increased risks in both men and women^51^. One study investigated the effects of waterpipe smoking in addition to traditional cigarette smoking on the risk of GERD. Etemadi et al. found that waterpipe smoking was most strongly associated with “severe and frequent” reflux, and the prevalence of the disease was associated with waterpipe use and duration. In addition, they found that cigarette smoking was a significant risk factor for any form of reflux among men^87^. Additionally, Wang et al. identified smoking and male gender as significant risk factors for reflux esophagitis, a subtype of GERD^141^. Similarly, a significant association between smoking and increased risk of RE has been reported in multiple studies^122,124,130,134^While other researches has found no significant association between GERD and smoking^103^. Almadi et al. observed a higher prevalence of GERD among smokers than non-smokers, but found no significant difference^38^. Wang et al. also did not find any association between cigarette smoking and risk of GERD^79^. Seo et al. developed a prediction model that was significantly able to predict GERD-related medical utilization in the South Korean population and identified PM_2.5_ as a risk factor for GERD^98^.

Our meta-analysis of 7 studies^43,45,72,102,133,138,140^ revealed that inhalational exposures were significantly associated with increased risk of GERD with a pooled estimate of 1.71 (95% CI 1.14–2.55; I^2^ = 94%), Fig. 3B In our sensitivity analysis, we excluded one study^102^ that had high heterogeneity; the analysis results revealed a reduction of I^2^ after removing Ahmed 2020 showing differences with a pooled estimate of 1.34 (95% CI 1.06 to 1.68; I^2^ = 78%), as shown in Supplementary Fig. 1 (1.3B vs 1. 3B′).

### Barrett’s esophagus

Smoking was identified as a risk factor for BE^41,58,142^. Schmidt et al. found that BE cases were significantly more likely to smoke^73^. Navab et al. found a positive correlation between current and prior tobacco use and BE^63^. Etemadi et al. found associations between smoking and BE that were independent of intensity, age at initiation, and GERD, but dependent on duration and years since cessation^87^. Other studies, however, produced conflicting results: some studies found that current and former smoking were not significantly associated with BE^43,84^. Additionally, Arroyo-Martínez et al. found that cigarette smoking showed no significant association with any form of the progression of BE^106^.

Our meta-analysis of 4 studies revealed that inhalational exposures were not significantly associated with risk of BE with a pooled estimate of 0.93 (95% CI 0.65–1.34; I^2^ = 76%), Fig. 3C. Sensitivity analysis was not performed since study weight and effect size were not concerning.

### Peptic ulcer disease

Ghanadi et al. found that smoking history was significantly associated with PUD^116^. Similarly, Chuang et al. also identified current tobacco use as a significant risk factor for PUD and that higher cumulative amounts of tobacco use were at higher risk for PUD^41^. Further, Begovic et al. found that more than half of ulcer patients enrolled in their study were smokers, and this difference was significant when compared to those who were non-smokers^40^. Levenstein et al. observed that age-, gender-, and socioeconomic status-adjusted associations were significant for smoking^55^. Park et al. investigated the role of changes in smoking status in the risk of gastroduodenal ulcer^68^. They observed that changes in smoking status, particularly from never smoker or former smoker to current smoker, had relatively higher HRs than other groups. When comparing smoking amount levels, they found that smokers who smoked > 20 pack-years had a significantly higher risk of PUD.

The role of PM exposure as a risk of PUD has been studied. Tsai et al. found that increases in both PM_2.5_ and PM_10_ were significantly associated with increased risk of PUD hospitalizations on warm days, but only PM_10_ was significantly associated with cold days^99^. Similarly, Wong et al. found that PUD hospitalization was associated with 10 ug/m^3^ increases in PM_2.5_. When investigating different types of ulcers, they found that associations with PM_2.5_ were significant for gastric ulcers, but not for duodenal ulcers^91^. Wu et al. observed that cumulative RRs for PM_2.5_ and PM_10_ showed nearly linear adverse effects^100^. When looking at gender-adjusted differences, significant associations for men and women were only observed for PM_2.5_. Quan et al. found that when air pollution exposures were assessed over 3-, 5-, and 7-day averages, pollutants were inversely associated with upper gastrointestinal bleeding (UGIB)^96^. Yu et al. observed a potential dose-response relationship between quartile concentrations of PM_2.5_ one month before detection of PUD. Subjects in the highest quartile of PM_2.5_ exposure displayed significantly higher risk, and the detection of PUD was associated with a 10 ug/m^3^ in PM_2.5_^101^. Our meta-analysis of 5 studies^41,96,99,101,116^ revealed that inhalational exposures were significantly associated with increased risk of PUD with a pooled estimate of 1.21 (95% CI 1.03–1.43; I^2^ = 93%), Fig. 3D. Sensitivity analysis was not performed since the study with a different effect size had a low weight.

### Esophageal cancer

Smoking as a risk factor for ECa was the focus of several studies^62,74,86,104,107,108,120,123,126,127,131,132,136^. Other studies focused on esophageal squamous cell carcinoma and also identified smoking as a risk factor and this risk increased with tobacco intensity and smoking duration^131^but there was no significant difference with respect to macroscopic type of cancer, as smoking showed similarly increased risks for both ulcerative type and medullary type eosinophilic squamous cell carcinoma (ESCC)^48,52,61,75,81,82^. Jayalekshmi et al. observed higher risks of ESCC for current bidi and cigarette smokers^47^. In gender association studies, Chen et al. found that there was a significant association between smoking and ECa in men, but not in women^110^. Etemadi A et al. studied how among tobacco users, metabolites of styrene and xylene were associated with ESCC, showing how specific components of tobacco smoke could contribute to disease^112^.

Conversely, some studies observed a non-significant relationship between inhalational exposures and ECa^41,66,83,137^. Arroyo-Martínez et al. found that cigarette smoking showed no significant association with any forms of progression of EAC, but in all forms of progression, smoking showed the highest association in the progression of BE without dysplasia to EAC^106^. Interestingly, Guo et al. found that current smoking was not significantly associated with any esophageal neoplasms while former smoking was^106,118^. However, Sheikh M et al. demonstrated that smoking cigarettes was not associated with the risk of ESCC^139^. Pournaghi et al. found a significant association between hookah smoking and an elevated risk of ESCC. Additionally, the same study identified that individuals who had quit smoking more than 10 times had a higher risk of ESCC^137^. Furthermore, a study on cumulative tar exposure revealed that individuals in the second tertile had an increased risk of ESCC, although this association became non-significant after adjusting for pack-years due to the high correlation between cumulative tar and pack-years^132^. Some studies looked at how smoking affected survival for those afflicted with ECa. Spreafico et al. found that smoking conferred worse overall survival in the combined Boston-Toronto Cohort for each 20 pack-year increase^77^. Others observed how current and former smoking contributed to decreased survival with respect to subtypes, specifically ESCC and EAC^80,84^. Dighe et al. found that current smoking was significantly associated with mortality of stage I-III ECa^111^. There were studies, however, that undermined this relationship^121^.

One study in particular, Rafiq R et al. evaluated both smoking and second-hand smoke as a risk factor for ECa, with increased risks associated with either exposure^70^. Pan et al. conducted two studies on the association between smoking and esophageal precancerous lesions (EPL), finding that smoking more than 20 cigarettes a day or having 40 or more pack-years of cumulative smoking was significantly associated with an elevated risk of EPL^67,135^. Cigarette smoking was also associated with the development of esophageal neuroendocrine neoplasms (NEN) and the risk increased to 4.7 times for all cases and to 4.0 for pure NEN cases^143^.

The relationship between PM_2.5_ exposure and ECa was also assessed. Li et al. observed a significantly positive association between PM_2.5_ and ECa incidence. When investigating the corresponding lag effects on ECa incidence, they found that a lag effect of 4 years showed the greatest risk for the overall population^92^. Li et al. examined the modifying effects of urbanization and socioeconomic factors and found a stronger association between PM_2.5_ and incidence for low urbanization groups, and this association was stronger for females than males^93^. Some studies identified long-term exposure to various components of PM_2.5_ such as black carbon, organic carbon, nitrate, sulfates, chlorides, and ammonium to be significantly associated with ECa^94,148^. Rao et al. found that although spatial distributions of the hospitalization rate of ECa in 2016 were not consistent with that of PM_2.5_ concentration in the same year, concentrations of PM_2.5_ in 2003 and 2004 had the strongest correlations with the hospitalization rate in 2016^97^. Sun D et al. observed a linear concentration-response relationship between long-term PM_2.5_ and ECa^90^. Huang et al. found that PM_10_ and NO_2_ had significant linear correlations with ECa mortality rates^147^. Our meta-analysis of 20 studies^41,61,66,67,70,75,81–83,86,107,108,118,123,126,131,132,135,136,143^ revealed that inhalational exposures were significantly associated with increased risk of ECa; pooled estimate of 1.83(95% CI1.54–2.18; I^2^ = 73%), Fig. 3E.

### Gastric cancer

As with the previous outcomes, most studies identified smoking as a risk factor for GCa^113,117,119,125,127,144^. Increased risk of GCa was associated with current cigarette smoking status, longer durations of smoking (at least 20 years) or later starting ages of smoking^56^. Current smoking was also found to have an increased risk of mortality from stomach cancer^60^. When assessing changes in smoking status, one study found that those who changed their current status to “smoking” showed an increased risk of GCa, and this risk was the highest in heavier smokers^69^. One study found that smoking was only significantly associated with single GCa and synchronous multiple gastric cancer in advanced gastric cancer patients^76^.

Current smoking also showed increased risk for gastric adenocarcinoma and gastric non-cardia adenocarcinoma^80^. Additionally, another study highlighted that smoking history was significantly associated with an increased risk of gastric adenocarcinoma^125^. Interestingly, Jayalekshmi et al. found that bidi smoking was significantly associated with GCa risk, but cigarette smoking was not. This risk increased with the number of bidis smoked daily and with the duration of bidi smoking^46^. Conversely, Chuang YS et al. found that tobacco use was a non-significant risk factor for GCa^41^. Other studies found that current smoking increased risks of intestinal metaplasia for both men and women. Further, this risk increased with increasing duration and total dose^49,78^. Chen et al. found that smoking significantly increased the risk of progression from chronic atrophic gastritis to intestinal metaplasia^109^.

In gender association studies, Chen ZM et al. found that there was a significant association between smoking and esophageal cancer in men, but not in women^110^.

Some studies investigated the role of waterpipe smoking in GCa risk. Several studies in Vietnam showed that waterpipe smoking was positively associated with GCa risk, but there was no significant interaction between the effects of water pipes and cigarette smoking on GCa risk^64,88,89^. Additionally, Environmental Tobacco Smoke (ETS), commonly known as secondhand smoke, has also been identified as a potential risk factor for GCa, suggesting that passive exposure to tobacco smoke may contribute to increased risk^128^.

Still, other studies found no such significant relationship between smoking and GCa^114,129^. Interestingly, Guo et al. found that current smoking was not significantly associated with any gastric neoplasms while former smoking was^118^.

Other studies investigated the relationship between PM and GCa. Some studies identified long-term exposure to various components of PM_2.5_ such as black carbon, organic carbon, nitrate, sulfates, chlorides, and ammonium to be significantly associated with GCa^94,148^. Ethan et al. found that as a single pollutant and as a multipollutant in combination with NO_2_, PM_2.5_ was significantly associated with stomach cancer mortality^145^. Fan et al. found that each 1ug/m^3^ increment in PM_2.5_ exposure was significantly associated with an increase in the risk of GCa^146^. Our meta-analysis of 13 studies^41,56,64,71,78,85,86,109,113,114,118,129,144^ revealed that inhalational exposures were significantly associated with increased risk of GCa with a pooled estimate of 1.71 (95% CI 1.39–2.10; I^2^ = 94%), Fig. 3F. Sensitivity analysis was not performed for ECa and GCa there were a relatively large number of studies and therefore the their moderate heterogeneity was to be expected.

## Discussion

In this systematic review, we investigated the associations between environmental exposures and diseases of the upper gastrointestinal tract. Through a comprehensive review of the available literature, we identified complex relationships between environmental exposures and upper gastrointestinal diseases. Most of the studies showed that exposures including PM, smoking, and waterpipe use were significantly associated with a higher risk of aerodigestive diseases. Based on the meta-analysis results, inhalational exposures were significantly associated with an increased risk of GERD, PUD, GCa, ECa, and esophagitis.

PM exposure is a global cause of significant pulmonary morbidity and mortality^7,13,150–157^. Our review supports existing evidence suggesting that exposure to PM may also increase the risk of diseases affecting the upper gastrointestinal tract. Studies included in this review demonstrated links between PM exposure and an increased risk of ECa and PUD, although the underlying mechanisms remain to be fully explained. These findings highlight the importance of considering environmental factors, such as air pollution, in the context of upper gastrointestinal health. PM consists of various harmful compounds that can trigger inflammatory responses, oxidative stress, and DNA damage that contribute to the development of cancer and ulceration. Moreover, studies showed that PM may disrupt the gut microbiota, leading to an increased risk of gastrointestinal inflammation and cancers^158^.

Cigarette smoking has been recognized as a major risk factor for various cancers, including those of the gastrointestinal tract. Consistent with previous research, our review highlights the detrimental effects of smoking on the upper gastrointestinal tract, with a notable association observed between smoking and an elevated risk of BE, GCa, ECa, and PUD. The carcinogenic effect of smoking is attributable to mutations in critical genes caused by tobacco metabolites and chemicals. Smoking is also associated with progression, aggressiveness, and reduced survival rates of existing gastrointestinal cancers. Smoking may be associated with exacerbation of GERD symptoms due to reducing esophageal sphincter tone and increasing gastric acid production^159^.

Waterpipe smoking has increased worldwide due to a perception that it is less harmful than cigarette smoking. However, waterpipe smoke contains tobacco and several toxicants that may increase the risk of developing aerodigestive disease, as identified in our review. Numerous carcinogens have been identified in waterpipe smoke including polycyclic aromatic hydrocarbons, volatile aldehydes, and heavy metals, which can cause DNA damage and develop cancer over time^160^. Moreover, emerging evidence suggests that vaping and marijuana use may also impact gastrointestinal health, although further investigation is warranted to better understand the nature of these associations.

Although this review was unable to identify papers identifying risks of gastrointestinal disease from vaping, e-cigarette/ vaping use has been associated with nonspecific symptoms including nausea, vomiting, diarrhea, and abdominal pain^161,162^. One case report identified non-respiratory complaints including nausea, vomiting, and fever as associated symptoms of E-cigarette or Vaping Product Use–Associated Lung Injury (EVALI)^163^. Nausea was also more frequently associated with cannabinoid-based vaping, but was not affected by concurrent smoking and vaping^164^.

Nitrosamines are potent carcinogens from tobacco products that contribute to esophageal and stomach cancer from smoking^165,166^. E-cigarettes also contain the same nitrosamines that directly cause DNA damage at relatively lower concentrations, but have been shown to be sufficient in inducing lung cancer and bladder hyperplasia in in vitro and murine translational models^167^. However the lag time of at least two decades for smokers to develop cancer makes it difficult to ascertain the full carcinogenic threat of vaping in humans.

Furthermore, the nicotinic acetylcholine receptor (nAChR), a genetic variant of which is consistently linked to lung cancer in large genetic studies, might mediate carcinogenesis through directly binding nicotine (and nitrosamines) in airway epithelium. This mechanism could provide direct carcinogenesis of nicotine and nicotine metabolites to all cells that express the nAChR, particularly in carriers of the variants that are associated with tobacco smoking and cancer^168–171^. Following the idea that inhaled nicotine could produce carcinogenic molecules in human users, an untargeted metabolomics analysis of urine demonstrated a trend of increased carcinogen biomarkers in the samples of a relatively small cohort of vapers (*n* = 34 vs. *n* = 45 non-users)^172^.

### Limitations

While this systematic review provides valuable insights into the associations between environmental exposures and upper gastrointestinal diseases, the included studies vary in design, methodology, and population characteristics. Some studies used adjusted models when calculating ORs or HRs (aOR; aHR), which varied in complexity and contributed to high heterogeneity. To address this problem, we used a random-effects model to account for variability between studies. However, the considerable heterogeneity, evident from a high I^2^may limit the generalizability of the pooled effect sizes. Factors such as differences in study designs, populations, interventions, and outcome measures may have contributed to this variability.

Additionally, many studies are observational thus, limiting causal inference and necessitating further research, including prospective cohort studies and mechanistic investigations. Furthermore, studies were required to be in English, which limited access to data from articles in languages in other languages. While this study included both PubMed and WoS databases for the identification of potentially eligible studies we understand that there are several databases that while complementary may have additional studies. Our risk of bias assessment (NOS) was able to evaluate the majority, but not all studies assessed in this review.

Other limitations revolved around how we defined environmental exposure and aerodigestive disease. Our study defined environmental exposures as air pollution in the form of PM, cigarette/tobacco smoke, marijuana smoke, and vape/e-cigarette aerosols. Due to this, it was not possible to completely cover the entire scope of environmental exposures that afflict society. In addition, our definition of aerodigestive disease focused on diseases of the upper gastrointestinal tract, which comprised esophagitis, GERD, BE, PUD, and esophageal/GCa based on the articles we found. There are likely other aerodigestive diseases that interact strongly with environmental exposures that were not covered by this paper. Due to these definitions and our inclusion/exclusion criteria, we also found no eligible articles that investigated the interactions of marijuana smoke and vape/e-cigarette aerosols with aerodigestive disease.

### Future research

Our search was unable to identify human studies that clearly define the carcinogenic potential of non-burning nicotine delivery products such as e-cigarettes. However, this could be due to the very extensive lag time between carcinogen exposure and clinical cancer diagnosis in humans. Future studies could expand our definitions to account for interactions that have not been included in this review, such as those of marijuana smoking, vaping, and those of the lower intestinal tract. Additionally, future studies could include occupational exposures as this study focused on the general population. Such additional exposures include asbestos, synthetic fiber dust, chrysotile dust, nephrite, and potentially harmful elements which are all commonly present in mining or textile industries and developing societies. Such investigations could yield valuable insights for those whose occupation or geographic location puts them at risk for such diseases, as aerodigestive disease is often underrecognized in those populations. In addition, this could identify how specific exposures incite disease in various cohorts.

## Conclusion

The implications of these findings are significant from both a public health and clinical perspective. Efforts to reduce exposure to environmental pollutants, such as PM, could potentially mitigate the burden of upper gastrointestinal diseases in affected populations. Similarly, targeted interventions aimed at reducing smoking behavior and promoting smoking cessation may help reduce the incidence of gastrointestinal disease and malignancy. Furthermore, continued research into the potential health effects of emerging trends, such as vaping and marijuana use, is crucial for informing preventive strategies and improving patient outcomes.

This review provides support for the connection between environmental exposures and digestive health, which is especially important considering that those who have been exposed to environmental/occupational inhalants are generally not screened for gastrointestinal disease as part of their exposure. We hope that this review will promote further recognition of the treatment of digestive disease with inhalational exposure.

In conclusion, this systematic review contributes to our understanding of the interplay between exposure to inhalational exposures and diseases of the upper gastrointestinal tract. By analyzing existing evidence and identifying knowledge gaps, this study highlights the need for approaches to address environmental risk factors and promote gastrointestinal health.

## Electronic supplementary material

Below is the link to the electronic supplementary material.

Supplementary Material 1.

Supplementary Material 2.

Supplementary Material 3.

Supplementary Material 4.

Supplementary Material 5.

Supplementary Material 6.

Supplementary Material 7.

Supplementary Material 8.

Supplementary Material 9.

## Data Availability

All data generated or analyzed during this study are included in this published article and its supplementary information files.

## Abbreviations

ACG: American College of Gastroenterology
AGCa: Advanced gastric cancer
AHR: Airway hyperreactivity
AHRQ: Agency for healthcare research and quality
AJCC: American Joint Committee on Cancer
aOR: Adjusted odds ratio
BE: Barrett’s esophagus
Ca: Cancer
CC: Correlation coefficient
CI: Confidence interval
EAC: Esophageal adenocarcinoma
ECa: Esophageal cancer
EE: Erosive esophagitis
EOE: Eosinophilic esophagitis
EPL: Esophageal precancerous lesions
ESCC: Esophageal squamous cell carcinoma
ESGE: European society of gastrointestinal endoscopy
EVALI: E-cigarette or vaping product use–associated lung injury
GAC: Gastric adenocarcinoma
GERD: Gastroesophageal reflux disease
GCa: Gastric cancer
GNCA: Gastric non-cardia adenocarcinoma
HR: Hazard ratio
LA: Los Angeles
nAChR: Nicotinic acetylcholine receptor
NEN: Neuroendocrine neoplasms
NYC: New York city
NOS: New-Castle Ottawa scale
OAD: Obstructive airway disease
OR: Odds ratio
PAH: Polycyclic aromatic hydrocarbons
PEO: Population, exposure, outcome
PM: Particulate matter
PRISMA: Preferred reporting items for systematic reviews and meta-analyses
PUD: Peptic ulcer disease
RE: Reflux esophagitis
RoB: Risk of bias
RR: Risk ratio
SGMCa: Synchronous multiple gastric cancer
SHS: Second-hand smoke
UGIB: Upper gastrointestinal bleeding
USA: United States of America
WHO: World Health Organization
WPT: Waterpipe tobacco
WTC: World trade center
9/11: September 11, 2001

## References

[1] National Institute of Environmental Health Sciences. *Exposure Science* https://www.niehs.nih.gov/health/topics/science/exposure.

[2] Orru, H., Ebi, K. L. & Forsberg, B. The interplay of climate change and air pollution on health. *Curr. Environ. Health Rep.* **4**, 504–513. 10.1007/s40572-017-0168-6 (2017). 29080073 10.1007/s40572-017-0168-6 PMC5676805

[3] World Health Organization. *Air Pollution*, (2024). https://www.who.int/health-topics/air-pollution#tab=tab_1.

[4] Shaddick, G., Thomas, M. L., Mudu, P., Ruggeri, G. & Gumy, S. Half the world’s population are exposed to increasing air pollution. *Npj Clim. Atmospheric Sci.* **3**, 23. 10.1038/s41612-020-0124-2 (2020).

[5] Landrigan, P. J. et al. Health consequences of environmental exposures: Changing global patterns of exposure and disease. *Ann. Glob Health*. **82**, 10–19. 10.1016/j.aogh.2016.01.005 (2016). 27325064 10.1016/j.aogh.2016.01.005

[6] Peters, A. et al. Exposure to traffic and the onset of myocardial infarction. *N. Engl. J. Med.* **351**, 1721–1730 (2004). 15496621 10.1056/NEJMoa040203

[7] Wellenius, G. A. et al. Effects of ambient air pollution on functional status in patients with chronic congestive heart failure: A repeated-measures study. *Environ. Health*. **6**, 26 (2007). 17845720 10.1186/1476-069X-6-26 PMC2014745

[8] Richter, J. E. & Rubenstein, J. H. Presentation and epidemiology of gastroesophageal reflux disease. *Gastroenterology* **154**, 267–276. 10.1053/j.gastro.2017.07.045 (2018). 28780072 10.1053/j.gastro.2017.07.045 PMC5797499

[9] Jang, S. H., Ryu, H. S., Choi, S. C. & Lee, S. Y. Psychological factors influence the gastroesophageal reflux disease (GERD) and their effect on quality of life among firefighters in South Korea. *Int. J. Occup. Environ. Health*. **22**, 315–320. 10.1080/10773525.2016.1235675 (2016). 27691373 10.1080/10773525.2016.1235675 PMC5137555

[10] Dent, J., El-Serag, H. B., Wallander, M. A. & Johansson, S. Epidemiology of gastro-oesophageal reflux disease: A systematic review. *Gut* **54**, 710–717. 10.1136/gut.2004.051821 (2005). 15831922 10.1136/gut.2004.051821 PMC1774487

[11] Savarino, E. et al. Advances in the physiological assessment and diagnosis of GERD. *Nat. Reviews Gastroenterol. Hepatol.* **14**, 665. 10.1038/nrgastro.2017.130 (2017). 10.1038/nrgastro.2017.130 28951582

[12] Shaheen, N. J. et al. The burden of gastrointestinal and liver diseases, 2006. *Am. J. Gastroenterol.* **101**, 2128–2138. 10.1111/j.1572-0241.2006.00723.x (2006). 16848807 10.1111/j.1572-0241.2006.00723.x

[13] Haider, S. H. et al. Predictive biomarkers of gastroesophageal reflux disease and barrett’s esophagus in world trade center exposed firefighters: A 15 year longitudinal study. *Sci. Rep.* **8**, 3106. 10.1038/s41598-018-21334-9 (2018). 29449669 10.1038/s41598-018-21334-9 PMC5814524

[14] Coppeta, L., Pietroiusti, A., Magrini, A., Somma, G. & Bergamaschi, A. Prevalence and characteristics of functional dyspepsia among workers exposed to cement dust. *Scand. J. Work Env Health* **34**, 396–402. 10.5271/sjweh.1275 (2008). 18853070 10.5271/sjweh.1275

[15] Joo, Y. H., Lee, S. S., Han, K. D. & Park, K. H. Association between Chronic Laryngitis and Particulate Matter Based on the Korea National Health and Nutrition Examination Survey 2008–2012. *Plos One* **10**10.1371/journal.pone.0133180 (2015). 10.1371/journal.pone.0133180 PMC4503512 26177353

[16] Havemann, B. D., Henderson, C. A. & El-Serag, H. B. The association between gastro-oesophageal reflux disease and asthma: A systematic review. *Gut* **56**, 1654–1664. 10.1136/gut.2007.122465 (2007). 17682001 10.1136/gut.2007.122465 PMC2095717

[17] Butt, J. et al. Helicobacter Pylori serology, and gastric cancer risk in prospective studies from china, japan, and Korea. *Cancer Prev. Res. (Phila)* **12**, 667–674. 10.1158/1940-6207.Capr-19-0238 (2019). 10.1158/1940-6207.CAPR-19-0238 PMC6854526 31350279

[18] Dong, J. & Thrift, A. P. Alcohol, smoking and risk of oesophago-gastric cancer. *Best Pract. Res. Clin. Gastroenterol.* **31**, 509–517. 10.1016/j.bpg.2017.09.002 (2017). 29195670 10.1016/j.bpg.2017.09.002

[19] Ferro, A. et al. Tobacco smoking and gastric cancer: Meta-analyses of published data versus pooled analyses of individual participant data (StoP Project). *Eur. J. Cancer Prev.* **27**, 197–204. 10.1097/cej.0000000000000401 (2018). 29595756 10.1097/CEJ.0000000000000401

[20] Kayamba, V., Heimburger, D. C., Morgan, D. R., Atadzhanov, M. & Kelly, P. Exposure to biomass smoke as a risk factor for oesophageal and gastric cancer in low-income populations: A systematic review. *Malawi Med. J.* **29**, 212–217. 10.4314/mmj.v29i2.25 (2017). 28955435 10.4314/mmj.v29i2.25 PMC5610298

[21] Li, L. F. et al. Cigarette smoking and gastrointestinal diseases: The causal relationship and underlying molecular mechanisms (review). *Int. J. Mol. Med.* **34**, 372–380. 10.3892/ijmm.2014.1786 (2014). 24859303 10.3892/ijmm.2014.1786

[22] Rota, M. et al. Dose-response association between cigarette smoking and gastric cancer risk: A systematic review and meta-analysis. *Gastric Cancer* **27**, 197–209. 10.1007/s10120-023-01459-1 (2024). 38231449 10.1007/s10120-023-01459-1

[23] Veerappan, A. et al. World trade center-cardiorespiratory and vascular dysfunction: Assessing the phenotype and metabolome of a murine particulate matter exposure model. *Sci. Rep.* **10**, 3130. 10.1038/s41598-020-58717-w (2020). 32081898 10.1038/s41598-020-58717-w PMC7035300

[24] Haider, S. H. et al. Multiomics of world trade center particulate matter-induced persistent airway hyperreactivity. Role of receptor for advanced glycation end products. *Am. J. Respir Cell. Mol. Biol.* **63**, 219–233. 10.1165/rcmb.2019-0064OC (2020). 32315541 10.1165/rcmb.2019-0064OC PMC7397767

[25] Prezant, D. J. et al. Cough and bronchial responsiveness in firefighters at the world trade center site. *N. Engl. J. Med.* **347**, 806–815. 10.1056/NEJMoa021300 (2002). 12226151 10.1056/NEJMoa021300

[26] de la Hoz, R. E. et al. Reflux symptoms and disorders and pulmonary disease in former world trade center rescue and recovery workers and volunteers. *J. Occup. Environ. Med.* **50**, 1351–1354. 10.1097/JOM.0b013e3181845f9b (2008). 19092489 10.1097/JOM.0b013e3181845f9b

[27] Li, J. et al. Gastroesophageal reflux symptoms and comorbid asthma and posttraumatic stress disorder following the 9/11 terrorist attacks on world trade center in new York City. *Am. J. Gastroenterol.* **106**, 1933–1941. 10.1038/ajg.2011.300 (2011). 21894225 10.1038/ajg.2011.300

[28] Webber, M. P. et al. Trends in respiratory symptoms of firefighters exposed to the world trade center disaster: 2001–2005. *Environ. Health Perspect.* **117**, 975–980. 10.1289/ehp.0800291 (2009). 19590693 10.1289/ehp.0800291 PMC2702416

[29] Liu, X. et al. The effect of world trade center exposure on the timing of diagnoses of obstructive airway disease, chronic rhinosinusitis, and gastroesophageal reflux disease. *Front. Public. Health*. **5**, 2. 10.3389/fpubh.2017.00002 (2017). 28229067 10.3389/fpubh.2017.00002 PMC5296346

[30] Shamseer, L. et al. Preferred reporting items for systematic review and meta-analysis protocols (PRISMA-P) 2015: Elaboration and explanation. *Bmj* **350**, g7647. 10.1136/bmj.g7647 (2015). 25555855 10.1136/bmj.g7647

[31] Liberati, A. et al. The PRISMA statement for reporting systematic reviews and meta-analyses of studies that evaluate health care interventions: Explanation and elaboration. *J. Clin. Epidemiol.* **62**, e1–34. 10.1016/j.jclinepi.2009.06.006 (2009). 19631507 10.1016/j.jclinepi.2009.06.006

[32] Wells, G. A. S. et al. The Newcastle-Ottawa Scale (NOS) for assessing the quality of nonrandomized studies in meta-analysis. (2011). http://www.ohri.ca/programs/clinical_epidemiology/oxford.asp.

[33] Li, H., Boakye, D., Chen, X., Hoffmeister, M. & Brenner, H. Association of body mass index with risk of Early-Onset colorectal cancer: systematic review and Meta-Analysis. *Official J. Am. Coll. Gastroenterol. ACG* **116** (2021). 10.14309/ajg.0000000000001393 PMC8560162 34309586

[34] Shamsrizi, P. et al. Variation of effect estimates in the analysis of mortality and length of hospital stay in patients with infections caused by bacteria-producing extended-spectrum beta-lactamases: A systematic review and meta-analysis. *BMJ Open.* **10**, e030266. 10.1136/bmjopen-2019-030266 (2020). 31964661 10.1136/bmjopen-2019-030266 PMC7044956

[35] Farooqi, M. S. et al. Noninvasive, multiomic, and multicompartmental biomarkers of reflux disease: A systematic review. *Gastro Hep Adv.* **2**, 608–620. 10.1016/j.gastha.2023.01.014 (2023). 38009162 10.1016/j.gastha.2023.01.014 PMC10673619

[36] Podury, S. et al. Severe acute respiratory syndrome and particulate matter exposure: A systematic review. *Life (Basel)* **13**. 10.3390/life13020538 (2023). 10.3390/life13020538 PMC9962044 36836898

[37] Viswanathan, M. et al. Assessing the risk of bias in individual studies in systematic review of health care interventions. Agency for Healthcare Research and Quality methods guide for comparative effectiveness reviews. 22479713

[38] Almadi, M. A. et al. Prevalence of symptoms of gastroesopahgeal reflux in a cohort of Saudi Arabians: A Study of 1265 subjects. *Saudi J. Gastroenterol.* **20**, 248–254. 10.4103/1319-3767.136982 (2014). 25038211 10.4103/1319-3767.136982 PMC4131308

[39] Baroudi, O. et al. Impact of lifestyle factors and nutrients intake on occurrence of gastrointestinal cancer in Tunisian population. *Tumour Biol.* **35**, 5815–5822. 10.1007/s13277-014-1771-x (2014). 24615521 10.1007/s13277-014-1771-x

[40] Begovic, G. & Selmani, R. Etiological factors in urgent gastroduodenal ulcer. *Pril (Makedon Akad. Nauk. Umet Odd Med. Nauki)* **36**, 203–210. 10.1515/prilozi-2015-0068 (2015). 27442386 10.1515/prilozi-2015-0068

[41] Chuang, Y. S. et al. Risks of substance uses, alcohol flush response, Helicobacter pylori infection and upper digestive tract diseases-An endoscopy cross-sectional study. *Kaohsiung J. Med. Sci.* **35**, 341–349. 10.1002/kjm2.12071 (2019). 31001924 10.1002/kjm2.12071 PMC11900762

[42] Crews, N. R. et al. Prevalence and predictors of gastroesophageal reflux complications in community subjects. *Dig. Dis. Sci.* **61**, 3221–3228. 10.1007/s10620-016-4266-3 (2016). 27510751 10.1007/s10620-016-4266-3 PMC5069175

[43] Dore, M. P. et al. Risk factors for erosive and non-erosive gastroesophageal reflux disease and barrett’s esophagus in nothern Sardinia. *Scand. J. Gastroenterol.* **51**, 1281–1287. 10.1080/00365521.2016.1200137 (2016). 27381266 10.1080/00365521.2016.1200137

[44] Filiberti, R. et al. Smoking as an independent determinant of barrett’s esophagus and, to a lesser degree, of reflux esophagitis. *Cancer Causes Control*. **26**, 419–429. 10.1007/s10552-014-0518-8 (2015). 25555994 10.1007/s10552-014-0518-8

[45] Ghoshal, U. C., Singh, R. & Rai, S. Prevalence and risk factors of gastroesophageal reflux disease in a rural Indian population. *Indian J. Gastroenterol.* **40**, 56–64. 10.1007/s12664-020-01135-7 (2021). 33443640 10.1007/s12664-020-01135-7

[46] Jayalekshmi, P. A. et al. Gastric cancer risk in relation to tobacco use and alcohol drinking in kerala, India–Karunagappally cohort study. *World J. Gastroenterol.* **21**, 12676–12685. 10.3748/wjg.v21.i44.12676 (2015). 26640345 10.3748/wjg.v21.i44.12676 PMC4658623

[47] Jayalekshmi, P. A., Nandakumar, A., Nair, R. A., Akiba, S. & Koriyama, C. Esophageal cancer in relation to alcohol drinking and tobacco use among men in kerala, India - Karunagappally cohort. *Cancer Epidemiol.* **74**, 102018. 10.1016/j.canep.2021.102018 (2021). 34507085 10.1016/j.canep.2021.102018

[48] Kayamba, V. et al. HIV infection and domestic smoke exposure, but not human papillomavirus, are risk factors for esophageal squamous cell carcinoma in zambia: A case-control study. *Cancer Med.* **4**, 588–595. 10.1002/cam4.434 (2015). 25641622 10.1002/cam4.434 PMC4402073

[49] Kim, K. et al. Smoking and urinary cotinine levels are predictors of increased risk for gastric intestinal metaplasia. *Cancer Res.* **79**, 676–684. 10.1158/0008-5472.Can-18-2268 (2019). 30563886 10.1158/0008-5472.CAN-18-2268

[50] Kim, O. et al. Gastroesophageal reflux disease and its related factors among women of reproductive age: Korea nurses’ health study. *BMC Public. Health* **18**, 1133. 10.1186/s12889-018-6031-3 (2018). 30241473 10.1186/s12889-018-6031-3 PMC6150961

[51] Kim, S. Y. et al. Gender specific differences in prevalence and risk factors for gastro-esophageal reflux disease. *J. Korean Med. Sci.* **34**, e158. 10.3346/jkms.2019.34.e158 (2019). 31144481 10.3346/jkms.2019.34.e158 PMC6543060

[52] Koca, T. et al. Dietary and demographical risk factors for oesophageal squamous cell carcinoma in the Eastern Anatolian region of Turkey where upper gastrointestinal cancers are endemic. *Asian Pac. J. Cancer Prev.* **16**, 1913–1917. 10.7314/apjcp.2015.16.5.1913 (2015). 25773844 10.7314/apjcp.2015.16.5.1913

[53] Koutlas, N. T. et al. Impact of smoking, alcohol consumption, and NSAID use on risk for and phenotypes of eosinophilic esophagitis. *Dis. Esophagus*. **31**, 1–7. 10.1093/dote/dox111 (2018). 29025076 10.1093/dote/dox111 PMC6036648

[54] Lee, S. P. et al. The clinical features and predisposing factors of asymptomatic erosive esophagitis. *Dig. Dis. Sci.* **61**, 3522–3529. 10.1007/s10620-016-4341-9 (2016). 27796766 10.1007/s10620-016-4341-9

[55] Levenstein, S., Jacobsen, R. K., Rosenstock, S. & Jørgensen, T. Mental vulnerability, *Helicobacter pylori*, and incidence of hospital-diagnosed peptic ulcer over 28 years in a population-based cohort. *Scand. J. Gastroenterol.* **52**, 954–961. 10.1080/00365521.2017.1324897 (2017). 28503971 10.1080/00365521.2017.1324897

[56] Lin, Y. et al. Sociodemographic and lifestyle factors in relation to gastric cancer in a high-risk region of china: A matched case-control study. *Nutr. Cancer*. **72**, 421–430. 10.1080/01635581.2019.1638425 (2020). 31306032 10.1080/01635581.2019.1638425

[57] Martinucci, I. et al. Gastroesophageal reflux symptoms among Italian university students: Epidemiology and dietary correlates using automatically recorded transactions. *BMC Gastroenterol.* **18**, 116. 10.1186/s12876-018-0832-9 (2018). 30016938 10.1186/s12876-018-0832-9 PMC6050672

[58] Matsuzaki, J. et al. Association of visceral fat area, smoking, and alcohol consumption with reflux esophagitis and barrett’s esophagus in Japan. *PLoS One*. **10**, e0133865. 10.1371/journal.pone.0133865 (2015). 26225858 10.1371/journal.pone.0133865 PMC4520496

[59] Miftahussurur, M. et al. Gastroesophageal reflux disease in an area with low *Helicobacter pylori*infection prevalence. *PLoS One*. **13**, e0205644. 10.1371/journal.pone.0205644 (2018). 30427843 10.1371/journal.pone.0205644 PMC6241118

[60] 60 Minami, Y. et al. Associations of cigarette smoking and alcohol drinking with stomach cancer survival: A prospective patient cohort study in Japan. *Int. J. Cancer*. **143**, 1072–1085. 10.1002/ijc.31408 (2018). 29603213 10.1002/ijc.31408

[61] Mlombe, Y. B. et al. Environmental risk factors for oesophageal cancer in malawi: A case-control study. *Malawi Med. J.* **27**, 88–92. 10.4314/mmj.v27i3.3 (2015). 26715952 10.4314/mmj.v27i3.3 PMC4688868

[62] Moses, A. et al. Risk factors for common cancers among patients at Kamuzu central hospital in lilongwe, malawi: A retrospective cohort study. *Malawi Med. J.* **29**, 136–141. 10.4314/mmj.v29i2.11 (2017). 28955421 10.4314/mmj.v29i2.11 PMC5610284

[63] Navab, F., Nathanson, B. H. & Desilets, D. J. The impact of lifestyle on barrett’s esophagus: A precursor to esophageal adenocarcinoma. *Cancer Epidemiol.* **39**, 885–891. 10.1016/j.canep.2015.10.013 (2015). 26519660 10.1016/j.canep.2015.10.013

[64] Nguyen, C. L. et al. Waterpipe tobacco smoking and risk of stomach cancer: A Case-Control study in Vietnamese men. *Asian Pac. J. Cancer Prev.* **23**, 1587–1593. 10.31557/apjcp.2022.23.5.1587 (2022). 35633542 10.31557/APJCP.2022.23.5.1587 PMC9587894

[65] Okamoto, T. & Ito, A. The association between smoking exposure and reflux esophagitis: A cross-sectional study among men conducted as a part of health screening. *Intern. Med.* **62**, 3571–3577. 10.2169/internalmedicine.0451-22 (2023). 37164660 10.2169/internalmedicine.0451-22 PMC10781557

[66] Okello, S. et al. Population attributable fraction of esophageal squamous cell carcinoma due to smoking and alcohol in Uganda. *BMC Cancer*. **16**, 446. 10.1186/s12885-016-2492-x (2016). 27400987 10.1186/s12885-016-2492-x PMC4940693

[67] Pan, D. et al. A distinct epidemiologic pattern of precancerous lesions of esophageal squamous cell carcinoma in a high-risk area of Huai’an, Jiangsu province, China. *Cancer Prev. Res. (Phila)*. **12**, 449–462. 10.1158/1940-6207.Capr-18-0462 (2019). 31040152 10.1158/1940-6207.CAPR-18-0462

[68] Park, S. K. et al. Change in smoking status and its relation to the risk of gastroduodenal ulcer in Korean men. *J. Gastroenterol. Hepatol.* **37**, 2091–2097. 10.1111/jgh.15979 (2022). 35940868 10.1111/jgh.15979

[69] Park, S. K. et al. The risk of gastric cancer according to changes in smoking status among Korean men. *Epidemiol. Health*. **44**, e2022086. 10.4178/epih.e2022086 (2022). 36228669 10.4178/epih.e2022086 PMC10089702

[70] Rafiq, R. et al. Secondhand smoking and the risk of esophageal squamous cell carcinoma in a high incidence region, Kashmir, India: A case-control-observational study. *Medicine (Baltimore)* **95**, e2340. 10.1097/md.0000000000002340 (2016). 10.1097/MD.0000000000002340 PMC4706255 26735535

[71] Ramos, M. et al. Risk factors associated with the development of gastric cancer - case-control study. *Rev. Assoc. Med .Bras* **64**, 611–619. 10.1590/1806-9282.64.07.611 (2018). 10.1590/1806-9282.64.07.611 30365663

[72] Sadafi, S., Azizi, A., Pasdar, Y., Shakiba, E. & Darbandi, M. Risk factors for gastroesophageal reflux disease: A population-based study. *BMC Gastroenterol.* **24**, 64. 10.1186/s12876-024-03143-9 (2024). 38317085 10.1186/s12876-024-03143-9 PMC10840240

[73] Schmidt, M. et al. Epidemiologic risk factors in a comparison of a barrett esophagus registry (BarrettNET) and a case-control population in Germany. *Cancer Prev. Res. (Phila)* **13**, 377–384. 10.1158/1940-6207.Capr-19-0474 (2020). 32066580 10.1158/1940-6207.CAPR-19-0474

[74] Sewram, V., Sitas, F., O’Connell, D. & Myers, J. Tobacco and alcohol as risk factors for oesophageal cancer in a high incidence area in South Africa. *Cancer Epidemiol.* **41**, 113–121. 10.1016/j.canep.2016.02.001 (2016). 26900781 10.1016/j.canep.2016.02.001

[75] Simba, H. et al. The contribution of smoking and smokeless tobacco to oesophageal squamous cell carcinoma risk in the African oesophageal cancer corridor: results from the ESCCAPE multicentre case-control studies. *Int. J. Cancer*. **152**, 2269–2282. 10.1002/ijc.34458 (2023). 36733225 10.1002/ijc.34458

[76] Song, D. H. et al. Analysis of characteristics and risk factors of patients with single gastric cancer and synchronous multiple gastric cancer among 14,603 patients. *Gut Liver* **18**, 231–244. 10.5009/gnl220491 (2024). 36987384 10.5009/gnl220491 PMC10938156

[77] Spreafico, A. et al. Early adulthood body mass index, cumulative smoking, and esophageal adenocarcinoma survival. *Cancer Epidemiol.* **47**, 28–34. 10.1016/j.canep.2016.11.009 (2017). 28088657 10.1016/j.canep.2016.11.009

[78] Thrift, A. P., Jove, A. G., Liu, Y., Tan, M. C. & El-Serag, H. B. Associations of duration, intensity, and quantity of smoking with risk of gastric intestinal metaplasia. *J. Clin. Gastroenterol.* **56**, e71–e76. 10.1097/mcg.0000000000001479 (2022). 33337636 10.1097/MCG.0000000000001479 PMC8875544

[79] Wang, H. Y. et al. Prevalence of gastro-esophageal reflux disease and its risk factors in a community-based population in Southern India. *BMC Gastroenterol.* **16**, 36. 10.1186/s12876-016-0452-1 (2016). 26979399 10.1186/s12876-016-0452-1 PMC4791779

[80] 80 Wang, S. M. et al. Population attributable risks of subtypes of esophageal and gastric cancers in the united States. *Am. J. Gastroenterol.* **116**, 1844–1852. 10.14309/ajg.0000000000001355 (2021). 34240714 10.14309/ajg.0000000000001355 PMC8410651

[81] Wei, M. et al. The mediation effect of serum metabolites on the relationship between long-term smoking exposure and esophageal squamous cell carcinoma. *BMC Cancer*. **21**, 415. 10.1186/s12885-021-08151-6 (2021). 33858379 10.1186/s12885-021-08151-6 PMC8050928

[82] Yang, H. et al. Risk factors of esophageal squamous cell cancer specific for different macroscopic types. *Nutr. Cancer* **72**, 1336–1344. 10.1080/01635581.2020.1733623 (2020). 32156160 10.1080/01635581.2020.1733623

[83] 83 Yang, X. et al. Smoking and alcohol drinking in relation to the risk of esophageal squamous cell carcinoma: A population-based case-control study in China. *Sci. Rep.* **7**, 17249. 10.1038/s41598-017-17617-2 (2017). 29222520 10.1038/s41598-017-17617-2 PMC5722909

[84] Yates, M. et al. Body mass index, smoking, and alcohol and risks of barrett’s esophagus and esophageal adenocarcinoma: A UK prospective cohort study. *Dig. Dis. Sci.* **59**, 1552–1559. 10.1007/s10620-013-3024-z (2014). 24500448 10.1007/s10620-013-3024-z PMC4067535

[85] Zacharakis, G. et al. Risk factors for gastric cancer and surveillance of premalignant gastric lesions: A prospective cohort study of central Saudi Arabia. *Curr. Oncol.* **30**, 8338–8351. 10.3390/curroncol30090605 (2023). 37754520 10.3390/curroncol30090605 PMC10528333

[86] Zhao, J. K. et al. Jiangsu four cancers study: A large case-control study of lung, liver, stomach, and esophageal cancers in Jiangsu province, China. *Eur. J. Cancer Prev.* **26**, 357–364. 10.1097/cej.0000000000000262 (2017). 27275735 10.1097/CEJ.0000000000000262 PMC6057468

[87] Etemadi, A. et al. The association between waterpipe smoking and gastroesophageal reflux disease. *Int. J. Epidemiol.* **46**, 1968–1977. 10.1093/ije/dyx158 (2017). 29025018 10.1093/ije/dyx158 PMC5837680

[88] Lai, H. T. et al. Waterpipe tobacco smoking and gastric Cancer risk among Vietnamese men. *PLoS One*. **11**, e0165587. 10.1371/journal.pone.0165587 (2016). 27802311 10.1371/journal.pone.0165587 PMC5089735

[89] Le, H. X. et al. A prospective cohort study on the association between waterpipe tobacco smoking and gastric cancer mortality in Northern Vietnam. *BMC Cancer* **22**, 803. 10.1186/s12885-022-09894-6 (2022). 35864477 10.1186/s12885-022-09894-6 PMC9306202

[90] 90 Sun, D. et al. Long-Term exposure to fine particulate matter and incidence of esophageal cancer: A prospective study of 0.5 million Chinese adults. *Gastroenterology* **165**, 61–70e65. 10.1053/j.gastro.2023.03.233 (2023). 37059339 10.1053/j.gastro.2023.03.233 PMC7615725

[91] Wong, C. M. et al. STROBE-long-term exposure to ambient fine particulate air pollution and hospitalization due to peptic ulcers. *Medicine (Baltimore)* **95**, e3543. 10.1097/md.0000000000003543 (2016). 10.1097/MD.0000000000003543 PMC4863781 27149464

[92] Li, P. et al. The lag effect of exposure to PM(2.5) on esophageal cancer in urban-rural areas across China. *Environ. Sci. Pollut Res. Int.* **29**, 4390–4400. 10.1007/s11356-021-15942-8 (2022). 34406566 10.1007/s11356-021-15942-8

[93] 93 Li, P. et al. The associations of air pollution and socioeconomic factors with esophageal cancer in China based on a spatiotemporal analysis. *Environ. Res.* **196**, 110415. 10.1016/j.envres.2020.110415 (2021). 33159927 10.1016/j.envres.2020.110415

[94] Li, Y. et al. Long-term exposure to ambient fine particulate matter constituents and mortality from total and site-specific gastrointestinal cancer. *Environ. Res.* **244**, 117927. 10.1016/j.envres.2023.117927 (2024). 38103778 10.1016/j.envres.2023.117927

[95] Lin, Y. C., Shih, H. S. & Lai, C. Y. Long-term nonlinear relationship between PM(2.5) and ten leading causes of death. *Environ. Geochem. Health*. **44**, 3967–3990. 10.1007/s10653-021-01136-1 (2022). 34773532 10.1007/s10653-021-01136-1

[96] Quan, S. et al. Upper Gastrointestinal bleeding due to peptic ulcer disease is not associated with air pollution: A case-crossover study. *BMC Gastroenterol.* **15**, 131. 10.1186/s12876-015-0363-6 (2015). 26467538 10.1186/s12876-015-0363-6 PMC4604641

[97] Rao, Z. et al. The Spatiotemporal correlation of PM(2.5) concentration on esophageal cancer hospitalization rate in Fujian province of China. *Environ. Sci. Pollut Res. Int.* **29**, 67325–67335. 10.1007/s11356-022-20587-2 (2022). 35524092 10.1007/s11356-022-20587-2

[98] Seo, H. S., Hong, J. & Jung, J. Relationship of meteorological factors and air pollutants with medical care utilization for gastroesophageal reflux disease in urban area. *World J. Gastroenterol.* **26**, 6074–6086. 10.3748/wjg.v26.i39.6074 (2020). 33132656 10.3748/wjg.v26.i39.6074 PMC7584054

[99] Tsai, S. S., Chiu, H. F. & Yang, C. Y. Ambient air pollution and hospital admissions for peptic ulcers in taipei: A time-stratified case-crossover study. *Int. J. Environ. Res. Public. Health*. **16**10.3390/ijerph16111916 (2019). 10.3390/ijerph16111916 PMC6603676 31151209

[100] 100 Wu, M. et al. Ambient air pollution and hospital visits for peptic ulcer disease in china: A three-year analysis. *Environ. Res.* **196**, 110347. 10.1016/j.envres.2020.110347 (2021). 33130162 10.1016/j.envres.2020.110347

[101] Yu, Z. et al. Association between past exposure to fine particulate matter (PM(2.5)) and peptic ulcer: A cross-sectional study in Eastern China. *Chemosphere* **265**, 128706. 10.1016/j.chemosphere.2020.128706 (2021). 33139052 10.1016/j.chemosphere.2020.128706

[102] Ahmed, S., Jamil, S., Shaikh, H. & Abbasi, M. Effects of life style factors on the symptoms of gastro esophageal reflux disease: A cross sectional study in a Pakistani population. *Pakistan J. Med. Sci.* **36**, 115–120. 10.12669/pjms.36.2.1371 (2020). 10.12669/pjms.36.2.1371 PMC6994865 32063943

[103] 103 et al. Prevalence and risk factors of gastroesophageal reflux disease among female medical students at Taif university, Saudi Arabia. *World Family Med.* **18**, 77–81. 10.5742/mewfm.2020.93912 (2020).

[104] Alcala, K. et al. Incident cancers attributable to using opium and smoking cigarettes in the Golestan cohort study. *Eclinicalmedicine* **64**10.1016/j.eclinm.2023.102229 (2023). 10.1016/j.eclinm.2023.102229 PMC10541463 37781157

[105] Alrashed, A. A. et al. Prevalence and risk factors of gastroesophageal reflux disease among Shaqra university students, Saudi Arabia. *J. Family Med. Prim. Care*. **8**, 462–467. 10.4103/jfmpc.jfmpc_443_18 (2019). 30984655 10.4103/jfmpc.jfmpc_443_18 PMC6436310

[106] 106 Arroyo-Martínez. Epidemiology of barrett’s esophagus and esophageal adenocarcinoma in spain. A unicentric study. *Rev. Esp. Enferm. Dig.* **108**, 609–616. 10.17235/reed.2016.4229/2016 (2016). 27616661 10.17235/reed.2016.4229/2016

[107] 107 Asombang, A. W. et al. Esophageal squamous cell cancer in a highly endemic region. *World J. Gastroenterol.* **22**, 2811–2817. 10.3748/wjg.v22.i9.2811 (2016). 26973419 10.3748/wjg.v22.i9.2811 PMC4778003

[108] Chen, W. Q. et al. Selection of high-risk individuals for esophageal cancer screening: A prediction model of esophageal squamous cell carcinoma based on a multicenter screening cohort in rural China. *Int. J. Cancer*. **148**, 329–339. 10.1002/ijc.33208 (2021). 32663318 10.1002/ijc.33208

[109] Chen, Z. F. et al. Risk factors in the development of gastric adenocarcinoma in the general population: A cross-sectional study of the Wuwei cohort. *Front. Microbiol.* **13**10.3389/fmicb.2022.1024155 (2023). 10.3389/fmicb.2022.1024155 PMC9878447 36713177

[110] Chen, Z. M. et al. Emerging tobacco-related cancer risks in china: A nationwide, prospective study of 0.5 million adults. *Cancer* **121**, 3097–3106. 10.1002/cncr.29560 (2015). 26331816 10.1002/cncr.29560 PMC4584499

[111] Dighe, S. G. et al. Clinical and Lifestyle-Related prognostic indicators among esophageal adenocarcinoma patients receiving treatment at a comprehensive Cancer center. *Cancers* **13**10.3390/cancers13184653 (2021). 10.3390/cancers13184653 PMC8465866 34572881

[112] Etemadi, A. et al. Exposure to polycyclic aromatic hydrocarbons, volatile organic compounds, and tobacco-specific nitrosamines and incidence of esophageal cancer. *Jnci-Journal Natl. Cancer Inst.* **116**, 379–388. 10.1093/jnci/djad218 (2024). 10.1093/jnci/djad218 PMC10919344 37856326

[113] Fang, C. et al. Risk factors of early proximal gastric carcinoma in Chinese diagnosed using WHO criteria. *J. Dig. Dis.* **16**, 327–336. 10.1111/1751-2980.12240 (2015). 25754397 10.1111/1751-2980.12240

[114] Flores-Luna, L. et al. Risk factors for gastric precancerous and cancers lesions in Latin American counties with difference gastric cancer risk. *Cancer Epidemiol.* **64**10.1016/j.canep.2019.101630 (2020). 10.1016/j.canep.2019.101630 PMC6983355 31756677

[115] Gado, A., Ebeid, B., Abdelmohsen, A. & Axon, A. Prevalence of reflux esophagitis among patients undergoing endoscopy in a secondary referral hospital in giza, Egypt. *Alexandria J. Med.* **51**, 89–94. 10.1016/j.ajme.2013.09.002 (2015).

[116] Ghanadi, K. & Anbari, K. Risk factors of peptic ulcer disease in Khorramabad city, Southwest of iran: A case control study. *World Family Med.* **16**, 133–138. 10.5742/mewfm.2018.93215 (2018).

[117] Ghosh, P., Mandal, S., Mustafi, S. M. & Murmu, N. Clinicopathological characteristics and incidence of gastric cancer in Eastern india: A retrospective study. *J. Gastrointest. Cancer*. **52**, 863–871. 10.1007/s12029-020-00478-w (2021). 32809138 10.1007/s12029-020-00478-w

[118] Guo, L. W. et al. Determinants of participation and detection rate of upper Gastrointestinal cancer from population-based screening program in China. *Cancer Med.* **8**, 7098–7107. 10.1002/cam4.2578 (2019). 31560836 10.1002/cam4.2578 PMC6853828

[119] 1 Hazarika, A., Bora, P. P. & Kumar, K. S. A clinical study on incidence, pathological pattern and management of gastric carcinoma in rural setup (Adichunchanagiri Institute of medical Sciences), Mandya. *J. Evol. Med. Dent. Sciences-Jemds*. **5**, 2114–2122. 10.14260/jemds/2016/496 (2016).

[120] Jideh, B., Weltman, M., Wu, Y. & Chan, C. H. Y. Esophageal squamous papilloma lacks clear clinicopathological associations. *World J. Clin. Cases*. **5**, 134–139. 10.12998/wjcc.v5.i4.134 (2017). 28470005 10.12998/wjcc.v5.i4.134 PMC5395981

[121] Kaimila, B. et al. Survival after diagnosis of esophageal squamous cell carcinoma in Malawi. *Jco Global Oncol.* **9**10.1200/go.23.00173 (2023). 10.1200/GO.23.00173 PMC10645405 37944090

[122] Kang, S. H. et al. A model for predicting the future risk of incident erosive esophagitis in an asymptomatic population undergoing regular check-ups. *Med. (Baltim).* **95**, e2591. 10.1097/md.0000000000002591 (2016). 10.1097/MD.0000000000002591 PMC5291576 26825906

[123] Kim, D. H. et al. Clinical significance of intensive endoscopic screening for synchronous esophageal neoplasm in patients with head and neck squamous cell carcinoma. *Scand. J. Gastroenterol.* **49**, 1486–1492. 10.3109/00365521.2013.832369 (2014). 25372595 10.3109/00365521.2013.832369

[124] Kim, S. W., Lee, J. H., Sim, Y. S., Ryu, Y. J. & Chang, J. H. Prevalence and risk factors for reflux esophagitis in patients with chronic obstructive pulmonary disease. *Korean J. Intern. Med.* **29**, 466–473. 10.3904/kjim.2014.29.4.466 (2014). 25045294 10.3904/kjim.2014.29.4.466 PMC4101593

[125] 125 Kumar, S., Metz, D. C., Ellenberg, S., Kaplan, D. E. & Goldberg, D. S. Risk factors and incidence of gastric cancer after detection of *Helicobacter pylori* infection: A large cohort study. *Gastroenterology* **158**, 527–536e527. 10.1053/j.gastro.2019.10.019 (2020). 31654635 10.1053/j.gastro.2019.10.019 PMC7010558

[126] Kunzmann, A. T. et al. Model for identifying individuals at risk for esophageal adenocarcinoma. *Clin. Gastroenterol. Hepatol.* **16**, 1229–1236e1224. 10.1016/j.cgh.2018.03.014 (2018). 29559360 10.1016/j.cgh.2018.03.014

[127] Laaksonen, M. A. et al. The future burden of oesophageal and stomach cancers attributable to modifiable behaviours in australia: A pooled cohort study. *Br. J. Cancer*. **128**, 1052–1069. 10.1038/s41416-022-02104-x (2023). 36564563 10.1038/s41416-022-02104-x PMC10006078

[128] Li, J. et al. Environmental tobacco smoke and cancer risk, a prospective cohort study in a Chinese population. *Environ. Res.* **191**, 110015. 10.1016/j.envres.2020.110015 (2020). 32818497 10.1016/j.envres.2020.110015

[129] Lim, J. H., Song, J. H., Chung, S. J., Chung, G. E. & Kim, J. S. Characteristics of interval gastric neoplasms detected within two years after negative screening endoscopy among Koreans. *BMC Cancer* **21**, 218. 10.1186/s12885-021-07929-y (2021). 33653298 10.1186/s12885-021-07929-y PMC7923316

[130] Liu, Z., Wei, L. & Ding, H. Clinical characteristics of reflux esophagitis among patients with liver cirrhosis: A case-control study. *Scand. J. Gastroenterol.* **57**, 384–391. 10.1080/00365521.2021.2018489 (2022). 34965186 10.1080/00365521.2021.2018489

[131] Lu, P., Gu, J., Zhang, N., Sun, Y. & Wang, J. Risk factors for precancerous lesions of esophageal squamous cell carcinoma in high-risk areas of rural china: A population-based screening study. *Med. (Baltim).* **99**, e21426. 10.1097/md.0000000000021426 (2020). 10.1097/MD.0000000000021426 PMC7402764 32756148

[132] Meyers, T. J. et al. Case-control study of cumulative cigarette tar exposure and lung and upper aerodigestive tract cancers. *Int. J. Cancer* **140**, 2040–2050. 10.1002/ijc.30632 (2017). 28164274 10.1002/ijc.30632 PMC5552057

[133] Ness-Jensen, E., Hammer, A. & Hopstock, L. A. Trends in gastro-oesophageal reflux in a Norwegian general population: The Tromsø study 1979–2016. *Scand. J. Gastroenterol.* **58**, 840–843. 10.1080/00365521.2023.2183733 (2023). 36847288 10.1080/00365521.2023.2183733

[134] Ohashi, S. et al. Visceral fat obesity is the key risk factor for the development of reflux erosive esophagitis in 40–69-years subjects. *Esophagus* **18**, 889–899. 10.1007/s10388-021-00859-5 (2021). 34117973 10.1007/s10388-021-00859-5 PMC8387261

[135] Pan, D. et al. Inverse relations between *Helicobacter pylori* infection and risk of esophageal precancerous lesions in drinkers and peanut consumption. *World J. Gastrointest. Oncol.* **14**, 1689–1698. 10.4251/wjgo.v14.i9.1689 (2022). 36187387 10.4251/wjgo.v14.i9.1689 PMC9516658

[136] Poosari, A. et al. Association between infection with *Campylobacter* species, poor oral health and environmental risk factors on esophageal cancer: A hospital-based case-control study in Thailand. *Eur. J. Med. Res.* **26**, 82. 10.1186/s40001-021-00561-3 (2021). 34332608 10.1186/s40001-021-00561-3 PMC8325836

[137] Pournaghi, S. J. et al. Tobacco consumption, opium use, alcohol drinking and the risk of esophageal cancer in North khorasan, Iran. *J. Subst. Use*. **24**, 105–109. 10.1080/14659891.2018.1523962 (2019).

[138] Rabiee, B. et al. Gastro esophageal reflux disease (GERD) prevalence and related risk factors in North of Iran. *Esophagus* **13**, 330–336. 10.1007/s10388-016-0536-6 (2016).

[139] Sheikh, M. et al. Individual and combined effects of environmental risk factors for esophageal cancer based on results from the golestan cohort study. *Gastroenterology* **156**, 1416–1427. 10.1053/j.gastro.2018.12.024 (2019). 30611753 10.1053/j.gastro.2018.12.024 PMC7507680

[140] Soroush, A. et al. Sex and smoking differences in the association between gastroesophageal reflux and risk of esophageal squamous cell carcinoma in a high-incidence area: Golestan cohort study. *Int. J. Cancer*. **152**, 1137–1149. 10.1002/ijc.34313 (2023). 36214797 10.1002/ijc.34313 PMC9851948

[141] Wang, K. et al. A population-based survey of gastroesophageal reflux disease in a region with high prevalence of esophageal cancer in China. *Chin. Med. J. (Engl)* **132**, 1516–1523. 10.1097/cm9.0000000000000275 (2019). 31045906 10.1097/CM9.0000000000000275 PMC6616241

[142] Wang, S. E. et al. Demographic and lifestyle risk factors for gastroesophageal reflux disease and Barrett’s esophagus in Australia. *Dis. Esophagus*. **35**10.1093/dote/doab058 (2022). 10.1093/dote/doab058 34409990

[143] Wang, Y. K. et al. Substance use and esophageal neuroendocrine neoplasm: A case-control study. *Kaohsiung J. Med. Sci.* **38**, 1224–1229. 10.1002/kjm2.12592 (2022). 36156405 10.1002/kjm2.12592 PMC11896237

[144] 144 Zhang, R. et al. Risk factors for gastric cancer: a large-scale, population-based case-control study. *Chin. Med. J. (Engl)* **134**, 1952–1958. 10.1097/cm9.0000000000001652 (2021). 34310399 10.1097/CM9.0000000000001652 PMC8382323

[145] Ethan, C. J. et al. Association between pm _2.5_ and mortality of stomach and colorectal cancer in xi’an: a time-series study. *Environ. Sci. Pollut. Res.* **27**, 22353–22363. 10.1007/s11356-020-08628-0 (2020). 10.1007/s11356-020-08628-0 32314282

[146] Fan, Z. Y. et al. Long-term exposure to fine particulate matter and site-specific cancer mortality: A difference-in-differences analysis in Jiangsu province, China. *Environ. Res.* **222**10.1016/j.envres.2023.115405 (2023). 10.1016/j.envres.2023.115405 36736553

[147] Huang, X. C. et al. The effects of air pollution on mortality and clinicopathological features of esophageal cancer. *Oncotarget* **8**, 58563–58576. 10.18632/oncotarget.17266 (2017). 28938579 10.18632/oncotarget.17266 PMC5601675

[148] 148 Li, Y. X. et al. Long-term exposure to ambient fine particulate matter constituents and mortality from total and site-specific gastrointestinal cancer. *Environ. Res.* **244**10.1016/j.envres.2023.117927 (2024). 10.1016/j.envres.2023.117927 38103778

[149] Adenusi, A. O., Magacha, H. M., Nwaneki, C. M., Asifat, O. A. & Annor, E. N. Cannabis use and associated gastrointestinal disorders: A literature review. *Cureus* **15**, e41825. 10.7759/cureus.41825 (2023). 37575784 10.7759/cureus.41825 PMC10423018

[150] Peters, A., Dockery, D. W., Muller, J. E. & Mittleman, M. A. Increased particulate air pollution and the triggering of myocardial infarction. *Circulation* **103**, 2810–2815 (2001). 11401937 10.1161/01.cir.103.23.2810

[151] Wellenius, G. A., Schwartz, J. & Mittleman, M. A. Particulate air pollution and hospital admissions for congestive heart failure in seven United States cities. *Am. J. Cardiol.* **97**, 404–408 (2006). 16442405 10.1016/j.amjcard.2005.08.061

[152] Dominici, F. et al. Fine particulate air pollution and hospital admission for cardiovascular and respiratory diseases. *JAMA: J. Am. Med. Assoc.* **295**, 1127–1134. 10.1001/jama.295.10.1127 (2006). 10.1001/jama.295.10.1127 PMC3543154 16522832

[153] Simkhovich, B. Z., Kleinman, M. T. & Kloner, R. A. Particulate air pollution and coronary heart disease. *Curr. Opin. Cardiol.* **24**, 604–609. 10.1097/HCO.0b013e32833161e5 (2009). 19696664 10.1097/HCO.0b013e32833161e5

[154] Organization, W. H. O out of 10 people worldwide breathe polluted air, but more countries are taking action(2018).

[155] Long, N. P. et al. High-Throughput omics and statistical learning integration for the discovery and validation of novel diagnostic signatures in colorectal cancer. *Int. J. Mol. Sci.* **20**10.3390/ijms20020296 (2019). 10.3390/ijms20020296 PMC6358915 30642095

[156] 156 Kwon, S. et al. Metabolic syndrome biomarkers of world trade center airway hyperreactivity: A 16-year prospective cohort study. *Int. J. Environ. Res.* **16**, 1486 10.3390/ijerph16091486 (2019). 10.3390/ijerph16091486 PMC6539892 31035527

[157] Haider, S. H. et al. Receptor for advanced glycation end-products and environmental exposure related obstructive airways disease: A systematic review. *Eur. Respir Rev.* **28**10.1183/16000617.0096-2018 (2019). 10.1183/16000617.0096-2018 PMC7006869 30918021

[158] Gupta, N. et al. Deleterious effect of air pollution on human microbial community and bacterial flora: A short review. *Int. J. Environ. Res. Public. Health* **19**10.3390/ijerph192315494 (2022). 10.3390/ijerph192315494 PMC9738139 36497569

[159] Kahrilas, P. J. & Gupta, R. R. Mechanisms of acid reflux associated with cigarette smoking. *Gut* **31**, 4–10. 10.1136/gut.31.1.4 (1990). 2318431 10.1136/gut.31.1.4 PMC1378332

[160] Mamtani, R. et al. Cancer risk in waterpipe smokers: A meta-analysis. *Int. J. Public. Health*. **62**, 73–83. 10.1007/s00038-016-0856-2 (2017). 27421466 10.1007/s00038-016-0856-2 PMC5288449

[161] Ali, M. et al. A Case Series of Vaping-Induced Lung Injury in a Community Hospital Setting. *Case Rep. Pulmonol.* 9631916. 10.1155/2020/9631916 (2020). 10.1155/2020/9631916 PMC7013298 32082682

[162] Vaithilingam, S., Venkata, A. N. & Meena, N. K. A 41-year-old man presenting with shortness of breath, nausea, vomiting, and diarrhea. *Chest* **159**, e87–e91. 10.1016/j.chest.2020.09.096 (2021). 33563460 10.1016/j.chest.2020.09.096

[163] Matta, P., Hamati, J. N., Unno, H. L. & Fox, M. D. E-cigarette or vaping product use-associated lung injury (EVALI) without respiratory symptoms. *Pediatrics* **145**10.1542/peds.2019-3408 (2020). 10.1542/peds.2019-3408 32317307

[164] Sund, L. J., Dargan, P. I., Archer, J. R. H., Blundell, M. S. & Wood, D. M. The emerging cloud: A survey of vapers, their health and utilization of healthcare within the UK. *QJM* **116**, 993–1001. 10.1093/qjmed/hcad210 (2023). 37738584 10.1093/qjmed/hcad210 PMC10753409

[165] 165 Lee, H. W. et al. E-cigarette smoke damages DNA and reduces repair activity in mouse lung, heart, and bladder as well as in human lung and bladder cells. *Proc. Natl. Acad. Sci. U S A* **115**, E1560–E1569. 10.1073/pnas.1718185115 (2018). 29378943 10.1073/pnas.1718185115 PMC5816191

[166] Tang, M. S. et al. Electronic-cigarette smoke induces lung adenocarcinoma and bladder urothelial hyperplasia in mice. *Proc. Natl. Acad. Sci. U S A* **116**, 21727–21731. 10.1073/pnas.1911321116 (2019). 31591243 10.1073/pnas.1911321116 PMC6815158

[167] Tang, M. S. & Tang, Y. L. Can electronic-cigarette vaping cause cancer? *J. Cancer Biol.* **2**, 68–70. 10.46439/cancerbiology.2.027 (2021). 35759322 10.46439/cancerbiology.2.027 PMC9222281

[168] Young, R. P. et al. Lung cancer gene associated with COPD: triple whammy or possible confounding effect? *Eur. Respir J.* **32**, 1158–1164. 10.1183/09031936.00093908 (2008). 18978134 10.1183/09031936.00093908

[169] Young, R. P. & Scott, R. J. Inhaled nicotine and lung cancer: Potential role of the nicotinic acetylcholine receptor. *Proc. Natl. Acad. Sci. U S A*. **117**, 4460–4461. 10.1073/pnas.1921567117 (2020). 32047047 10.1073/pnas.1921567117 PMC7060687

[170] Tang, M. S. Reply to young and scott: nicotine and nicotinic acetylcholine receptor mutations in electronic-cigarette smoke lung carcinogenicity. *Proc. Natl. Acad. Sci. U S A*. **117**, 4462–4463. 10.1073/pnas.1922490117 (2020). 32047046 10.1073/pnas.1922490117 PMC7060684

[171] Tang, M. S. et al. DNA damage, DNA repair and carcinogenicity: Tobacco smoke versus electronic cigarette aerosol. *Mutat. Res. Rev. Mutat. Res.* **789**, 108409. 10.1016/j.mrrev.2021.108409 (2022). 35690412 10.1016/j.mrrev.2021.108409 PMC9208310

[172] Hsiao, Y. C. et al. Untargeted metabolomics to characterize the urinary chemical landscape of E-cigarette users. *Chem. Res. Toxicol.* **36**, 630–642. 10.1021/acs.chemrestox.2c00346 (2023). 36912507 10.1021/acs.chemrestox.2c00346 PMC10371198

