# Supplementary material for: Gastroesophageal disease risk and inhalational exposure a systematic review and meta-analysis
Source: Sci Rep. 2025 Jul 2;15:22581. doi: 10.1038/s41598-025-06620-7 (PMC12218983; doi:10.1038/s41598-025-06620-7)
Supplement: Supplementary file 1 — Supplementary Material 1. [file 41598_2025_6620_MOESM1_ESM.docx]

| **Supplemental Table 1. Unique Manuscripts Identified after Removal of Duplicates (N = 1,476)** | | | | | |
| --- | --- | --- | --- | --- | --- |
|  | **Author** | **Year** | **Title** | **Journal** | **DOI** |
|  | **PubMed (N = 542)** | | | | |
| **1** | Abbes, L., Perrod, G., Rahmi, G. and Cellier, C. | 2017 | Esophageal intramural pseudodiverticulosis, a rare cause of stenosis | Clin Res Hepatol Gastroenterol | 10.1016/j.clinre.2017.04.001 |
| **2** | Abiko, S., Shimizu, Y., Miyamoto, S., Ishikawa, M., Matsuda, K., Tsuda, M., Mizushima, T., Yamamoto, K., Ono, S., Kudo, T., Ono, K. and Sakamoto, N. | 2018 | Risk assessment of metachronous squamous cell carcinoma after endoscopic resection for esophageal carcinoma based on the genetic polymorphisms of alcoholdehydrogense-1B aldehyde dehydrogenase-2: temperance reduces the risk | J Gastroenterol | 10.1007/s00535-018-1441-7 |
| **3** | Abioye, A. I., Odesanya, M. O., Abioye, A. I. and Ibrahim, N. A. | 2015 | Physical activity and risk of gastric cancer: a meta-analysis of observational studies | Br J Sports Med | 10.1136/bjsports-2013-092778 |
| **4** | Abnet, C. C., Arnold, M. and Wei, W. Q. | 2018 | Epidemiology of Esophageal Squamous Cell Carcinoma | Gastroenterology | 10.1053/j.gastro.2017.08.023 |
| **5** | Aceves, S. S. | 2019 | Local Antigen Deposition in Eosinophilic Esophagitis: Implications for Immune Activation | Gastroenterology | 10.1053/j.gastro.2019.05.044 |
| **6** | Achilleos, A. | 2016 | Evidence-based Evaluation and Management of Chronic Cough | Med Clin North Am | 10.1016/j.mcna.2016.04.008 |
| **7** | Adami, H. O. and Nyrén, O. | 2016 | Enigmas, priorities and opportunities in cancer epidemiology | Eur J Epidemiol | 10.1007/s10654-016-0218-7 |
| **8** | Adejumo, A. C., Li, J., Akanbi, O., Adejumo, K. L. and Bukong, T. N. | 2019 | Reduced Prevalence of Alcoholic Gastritis in Hospitalized Individuals Who Consume Cannabis | Alcohol Clin Exp Res | 10.1111/acer.13930 |
| **9** | Akizue, N., Okimoto, K., Arai, M., Hirotsu, Y., Amemiya, K., Oura, H., Kaneko, T., Tokunaga, M., Ishikawa, K., Ohta, Y., Taida, T., Saito, K., Maruoka, D., Matsumura, T., Nakagawa, T., Nishimura, M., Chiba, T., Matsushita, K., Mochizuki, H., Yokosuka, O., Omata, M. and Kato, N. | 2021 | Comprehensive mutational analysis of background mucosa in patients with Lugol-voiding lesions | Cancer Med | 10.1002/cam4.3905 |
| **10** | Aksamit, T. R., O'Donnell, A. E., Barker, A., Olivier, K. N., Winthrop, K. L., Daniels, M. L. A., Johnson, M., Eden, E., Griffith, D., Knowles, M., Metersky, M., Salathe, M., Thomashow, B., Tino, G., Turino, G., Carretta, B. and Daley, C. L. | 2017 | Adult Patients With Bronchiectasis: A First Look at the US Bronchiectasis Research Registry | Chest | 10.1016/j.chest.2016.10.055 |
| **11** | Al-Qadasi, F. A., Shah, S. A. and Ghazi, H. F. | 2017 | Tobacco chewing and risk of gastric cancer: a case-control study in Yemen | East Mediterr Health J | 10.26719/2016.22.10.719 |
| **12** | Albert, R. K., Smith, B., Perlman, C. E. and Schwartz, D. A. | 2019 | Is Progression of Pulmonary Fibrosis due to Ventilation-induced Lung Injury? | Am J Respir Crit Care Med | 10.1164/rccm.201903-0497PP |
| **13** | Almadi, M. A., Almousa, M. A., Althwainy, A. F., Altamimi, A. M., Alamoudi, H. O., Alshamrani, H. S., Alharbi, O. R., Azzam, N. A., Sadaf, N. and Aljebreen, A. M. | 2014 | Prevalence of symptoms of gastroesopahgeal reflux in a cohort of Saudi Arabians: a study of 1265 subjects | Saudi J Gastroenterol | 10.4103/1319-3767.136982 |
| **14** | Alwan, H., La Rosa, S., Andreas Kopp, P., Germann, S., Maspoli-Conconi, M., Sempoux, C. and Bulliard, J. L. | 2020 | Incidence trends of lung and gastroenteropancreatic neuroendocrine neoplasms in Switzerland | Cancer Med | 10.1002/cam4.3524 |
| **15** | American College of Physicians | 2018 | Hot Tea Consumption and the Risk for Esophageal Cancer | Ann Intern Med | 10.7326/p17-9054 |
| **16** | Ami, R., Hatta, W., Iijima, K., Koike, T., Ohkata, H., Kondo, Y., Ara, N., Asanuma, K., Asano, N., Imatani, A. and Shimosegawa, T. | 2017 | Factors Associated With Metachronous Gastric Cancer Development After Endoscopic Submucosal Dissection for Early Gastric Cancer | J Clin Gastroenterol | 10.1097/mcg.0000000000000620 |
| **17** | Andresen, N. S., Lee, D. J., Kowalski, C. E. and Bayon, R. | 2018 | Fall With e-Cigarette in Mouth Resulting in Pharyngeal and Esophageal Burns | JAMA Otolaryngol Head Neck Surg | 10.1001/jamaoto.2017.3265 |
| **18** | Ansari, A. Z., Bhatia, N. Y., Gharat, S. A., Godad, A. P. and Doshi, G. M. | 2023 | Exploring Cytokines as Potential Target in Peptic Ulcer Disease: A Systematic Update | Endocr Metab Immune Disord Drug Targets | 10.2174/1871530322666220829142124 |
| **19** | Arora, Z., Garber, A. and Thota, P. N. | 2016 | Risk factors for Barrett's esophagus | J Dig Dis | 10.1111/1751-2980.12332 |
| **20** | Arul, P., Vinoth, B., Alexander, T., Phansalkar, M. and Padhi, S. | 2015 | Correlation of narrow band imaging endoscopy and histopathology in the diagnosis of nonerosive reflux disease | Saudi J Gastroenterol | 10.4103/1319-3767.164205 |
| **21** | Asanuma, K., Iijima, K. and Shimosegawa, T. | 2016 | Gender difference in gastro-esophageal reflux diseases | World J Gastroenterol | 10.3748/wjg.v22.i5.1800 |
| **22** | Asombang, A. W., Chishinga, N., Nkhoma, A., Chipaila, J., Nsokolo, B., Manda-Mapalo, M., Montiero, J. F. G., Banda, L. and Dua, K. S. | 2019 | Systematic review and meta-analysis of esophageal cancer in Africa: Epidemiology, risk factors, management and outcomes | World J Gastroenterol | 10.3748/wjg.v25.i31.4512 |
| **23** | Asombang, A. W., Kayamba, V., Lisulo, M. M., Trinkaus, K., Mudenda, V., Sinkala, E., Mwanamakondo, S., Banda, T., Soko, R. and Kelly, P. | 2016 | Esophageal squamous cell cancer in a highly endemic region | World J Gastroenterol | 10.3748/wjg.v22.i9.2811 |
| **24** | Austin, S., Bailey, D., Chandu, A., Dastaran, M. and Judge, R. | 2015 | Analysis of commonly reported medical conditions amongst patients receiving dental implant therapy in private practice | Aust Dent J | 10.1111/adj.12237 |
| **25** | Baba, Y., Yoshida, N., Kinoshita, K., Iwatsuki, M., Yamashita, Y. I., Chikamoto, A., Watanabe, M. and Baba, H. | 2018 | Clinical and Prognostic Features of Patients With Esophageal Cancer and Multiple Primary Cancers: A Retrospective Single-institution Study | Ann Surg | 10.1097/sla.0000000000002118 |
| **26** | Badulak, J. H., Schurr, M., Sauaia, A., Ivashchenko, A. and Peltz, E. | 2018 | Defining the criteria for intubation of the patient with thermal burns | Burns | 10.1016/j.burns.2018.02.016 |
| **27** | Bai, F. and Xiao, K. | 2020 | Prediction of gastric cancer risk: association between ZBTB20 genetic variance and gastric cancer risk in Chinese Han population | Biosci Rep | 10.1042/bsr20202102 |
| **28** | Baldwin-Hunter, B. L., Knotts, R. M., Leeds, S. D., Rubenstein, J. H., Lightdale, C. J. and Abrams, J. A. | 2019 | Use of the Electronic Health Record to Target Patients for Non-endoscopic Barrett's Esophagus Screening | Dig Dis Sci | 10.1007/s10620-019-05707-2 |
| **29** | Banday, A. R., Papenberg, B. W. and Prokunina-Olsson, L. | 2020 | When the Smoke Clears m(6)A from a Y Chromosome-Linked lncRNA, Men Get an Increased Risk of Cancer | Cancer Res | 10.1158/0008-5472.Can-20-0961 |
| **30** | Bang, C. S., Baik, G. H., Kim, J. H., Kim, J. B., Suk, K. T., Yoon, J. H., Kim, Y. S. and Kim, D. J. | 2014 | Peptic ulcer disease in liver cirrhosis and chronic hepatitis: impact of portal hypertension | Scand J Gastroenterol | 10.3109/00365521.2014.923501 |
| **31** | Baroudi, O., Chaaben, A. B., Mezlini, A., Moussa, A., Omrane, I., Jilson, I., Benammar-Elgaaied, A. and Chabchoub, S. | 2014 | Impact of lifestyle factors and nutrients intake on occurrence of gastrointestinal cancer in Tunisian population | Tumour Biol | 10.1007/s13277-014-1771-x |
| **32** | Barrea, L., Muscogiuri, G., Modica, R., Altieri, B., Pugliese, G., Minotta, R., Faggiano, A., Colao, A. and Savastano, S. | 2021 | Cardio-Metabolic Indices and Metabolic Syndrome as Predictors of Clinical Severity of Gastroenteropancreatic Neuroendocrine Tumors | Front Endocrinol (Lausanne) | 10.3389/fendo.2021.649496 |
| **33** | Bawankule, R., Singh, A., Kumar, K. and Pedgaonkar, S. | 2019 | Oral problems and associated risk indicators in adults in the Russian Federation, India, and China | BMC Oral Health | 10.1186/s12903-019-0811-8 |
| **34** | Becskeházi, E., Korsós, M. M., Gál, E., Tiszlavicz, L., Hoyk, Z., Deli, M. A., Köhler, Z. M., Keller-Pintér, A., Horváth, A., Csekő, K., Helyes, Z., Hegyi, P. and Venglovecz, V. | 2021 | Inhibition of NHE-1 Increases Smoke-Induced Proliferative Activity of Barrett's Esophageal Cell Line | Int J Mol Sci | 10.3390/ijms221910581 |
| **35** | Bede-Ojimadu, O. and Orisakwe, O. E. | 2020 | Exposure to Wood Smoke and Associated Health Effects in Sub-Saharan Africa: A Systematic Review | Ann Glob Health | 10.5334/aogh.2725 |
| **36** | Begovic, G. and Selmani, R. | 2015 | Etiological Factors in Urgent Gastroduodenal Ulcer | Pril (Makedon Akad Nauk Umet Odd Med Nauki) | 10.1515/prilozi-2015-0068 |
| **37** | Benli Yavuz, B., Koç, M., Kozacıoğlu, S., Kanyılmaz, G. and Aktan, M. | 2019 | Prognostic importance of PTEN, EGFR, HER-2, and IGF-1R in gastric cancer patients treated with postoperative chemoradiation | Turk J Med Sci | 10.3906/sag-1802-34 |
| **38** | Bhandari, S. and Venkatesan, T. | 2017 | Clinical Characteristics, Comorbidities and Hospital Outcomes in Hospitalizations with Cyclic Vomiting Syndrome: A Nationwide Analysis | Dig Dis Sci | 10.1007/s10620-016-4432-7 |
| **39** | Bhat, G. A., Bhat, A. B., Lone, M. M. and Dar, N. A. | 2017 | Association of Genetic Variants of CYP2C19 and CYP2D6 with Esophageal Squamous Cell Carcinoma Risk in Northern India, Kashmir | Nutr Cancer | 10.1080/01635581.2017.1299874 |
| **40** | Biagioni, M., Olmos, J. I., Antelo, P., Waldbaum, C., Wonaga, A. and Sordá, J. | 2018 | Dysphagia caused by intramural oesophageal pseudodiverticulosis: An unusual endoscopic finding | Gastroenterol Hepatol | 10.1016/j.gastrohep.2017.08.011 |
| **41** | Bikov, A., Lazar, Z., Gyulai, N., Szentkereszty, M., Losonczy, G., Horvath, I. and Galffy, G. | 2015 | Exhaled Breath Condensate pH in Lung Cancer, the Impact of Clinical Factors | Lung | 10.1007/s00408-015-9778-7 |
| **42** | Botteri, E., Gallus, S. and Lugo, A. | 2021 | Response to Hoffmeister et al | Am J Gastroenterol | 10.14309/ajg.0000000000001152 |
| **43** | Bozzella, M. J., Magyar, M., DeBiasi, R. L. and Ferrer, K. | 2020 | Epiglottitis Associated With Intermittent E-cigarette Use: The Vagaries of Vaping Toxicity | Pediatrics | 10.1542/peds.2019-2399 |
| **44** | Brewczyński, A., Jabłońska, B., Mrowiec, S., Składowski, K. and Rutkowski, T. | 2020 | Nutritional Support in Head and Neck Radiotherapy Patients Considering HPV Status | Nutrients | 10.3390/nu13010057 |
| **45** | Brewczyński, A., Jabłońska, B. and Pawlicki, K. | 2017 | Associations Between Nutritional Parameters and Clinicopathologic Factors in Patients with Gastric Cancer: A Comprehensive Study | Nutr Cancer | 10.1080/01635581.2017.1324993 |
| **46** | Brodin, N. P., Kabarriti, R., Pankuch, M., Schechter, C. B., Gondi, V., Kalnicki, S., Guha, C., Garg, M. K. and Tomé, W. A. | 2019 | A Quantitative Clinical Decision-Support Strategy Identifying Which Patients With Oropharyngeal Head and Neck Cancer May Benefit the Most From Proton Radiation Therapy | Int J Radiat Oncol Biol Phys | 10.1016/j.ijrobp.2018.11.039 |
| **47** | Brouwer, A. F., Eisenberg, M. C. and Meza, R. | 2018 | Case Studies of Gastric, Lung, and Oral Cancer Connect Etiologic Agent Prevalence to Cancer Incidence | Cancer Res | 10.1158/0008-5472.Can-17-3467 |
| **48** | Bruley des Varannes, S., Cestari, R., Usova, L., Triantafyllou, K., Alvarez Sanchez, A., Keim, S., Bergmans, P., Marelli, S., Grahl, E. and Ducrotté, P. | 2014 | Classification of adults suffering from typical gastroesophageal reflux disease symptoms: contribution of latent class analysis in a European observational study | BMC Gastroenterol | 10.1186/1471-230x-14-112 |
| **49** | Buas, M. F., Onstad, L., Levine, D. M., Risch, H. A., Chow, W. H., Liu, G., Fitzgerald, R. C., Bernstein, L., Ye, W., Bird, N. C., Romero, Y., Casson, A. G., Corley, D. A., Shaheen, N. J., Wu, A. H., Gammon, M. D., Reid, B. J., Hardie, L. J., Peters, U., Whiteman, D. C. and Vaughan, T. L. | 2015 | MiRNA-Related SNPs and Risk of Esophageal Adenocarcinoma and Barrett's Esophagus: Post Genome-Wide Association Analysis in the BEACON Consortium | PLoS One | 10.1371/journal.pone.0128617 |
| **50** | Budukh, A., Shah, S., Kulkarni, S., Pimple, S., Patil, S., Chaukar, D. and Pramesh, C. S. | 2022 | Tobacco and cancer awareness program among school children in rural areas of Ratnagiri district of Maharashtra state in India | Indian J Cancer | 10.4103/ijc.IJC_629_19 |
| **51** | Bugter, O., van Iwaarden, D. L. P., Dronkers, E. A. C., de Herdt, M. J., Wieringa, M. H., Verduijn, G. M., Mureau, M. A. M., Ten Hove, I., van Meerten, E., Hardillo, J. A. and Baatenburg de Jong, R. J. | 2019 | Survival of patients with head and neck cancer with metachronous multiple primary tumors is surprisingly favorable | Head Neck | 10.1002/hed.25595 |
| **52** | Busch, E. L., Zevallos, J. P. and Olshan, A. F. | 2016 | Gastroesophageal reflux disease and odds of head and neck squamous cell carcinoma in North Carolina | Laryngoscope | 10.1002/lary.25716 |
| **53** | Butt, J., Varga, M. G., Wang, T., Tsugane, S., Shimazu, T., Zheng, W., Abnet, C. C., Yoo, K. Y., Park, S. K., Kim, J., Jee, S. H., Qiao, Y. L., Shu, X. O., Waterboer, T., Pawlita, M. and Epplein, M. | 2019 | Smoking, Helicobacter Pylori Serology, and Gastric Cancer Risk in Prospective Studies from China, Japan, and Korea | Cancer Prev Res (Phila) | 10.1158/1940-6207.Capr-19-0238 |
| **54** | Cai, M., Dai, S., Chen, W., Xia, C., Lu, L., Dai, S., Qi, J., Wang, M., Wang, M., Zhou, L., Lei, F., Zuo, T., Zeng, H. and Zhao, X. | 2017 | Environmental factors, seven GWAS-identified susceptibility loci, and risk of gastric cancer and its precursors in a Chinese population | Cancer Med | 10.1002/cam4.1038 |
| **55** | Camargo, M. C., Song, M., Ito, H., Oze, I., Koyanagi, Y. N., Kasugai, Y., Rabkin, C. S. and Matsuo, K. | 2021 | Associations of circulating mediators of inflammation, cell regulation and immune response with esophageal squamous cell carcinoma | J Cancer Res Clin Oncol | 10.1007/s00432-021-03687-3 |
| **56** | Camilleri, M. and Zheng, T. | 2023 | Cannabinoids and the Gastrointestinal Tract | Clin Gastroenterol Hepatol | 10.1016/j.cgh.2023.07.031 |
| **57** | Cammarano, C. A. and Villaluz, J. E. | 2021 | A Reason to Rethink Fasting Guidelines? Marijuana-Induced Gastroparesis and the Implications for Aspiration Risk in the Nil Per Os (NPO) Patient: A Case Report | Am J Case Rep | 10.12659/ajcr.934187 |
| **58** | Canseco-Ávila, L. M., Zamudio-Castellanos, F. Y., Sánchez-González, R. A., Trujillo-Vizuet, M. G., Domínguez-Arrevillaga, S. and López-López, C. A. | 2019 | Gastric cancer epidemiology in tertiary healthcare in Chiapas | Rev Gastroenterol Mex (Engl Ed) | 10.1016/j.rgmx.2018.06.006 |
| **59** | Cao, J., Chen, Z., Tian, C., Yu, J., Zhang, H., Yang, J. and Yang, W. | 2020 | A Shared Susceptibility Locus in the p53 Gene for both Gastric and Esophageal Cancers in a Northwestern Chinese Population | Genet Test Mol Biomarkers | 10.1089/gtmb.2020.0192 |
| **60** | Castro, C., Peleteiro, B. and Lunet, N. | 2018 | Modifiable factors and esophageal cancer: a systematic review of published meta-analyses | J Gastroenterol | 10.1007/s00535-017-1375-5 |
| **61** | Cavatorta, O., Scida, S., Miraglia, C., Barchi, A., Nouvenne, A., Leandro, G., Meschi, T., De' Angelis, G. L. and Di Mario, F. | 2018 | Epidemiology of gastric cancer and risk factors | Acta Biomed | 10.23750/abm.v89i8-S.7966 |
| **62** | Chan, P. K., To, K. F., Tsang, S. H., Lau, C. H., Kwong, W. H. and Chan, Y. H. | 2017 | Human papillomavirus infection and squamous cell carcinoma in Hong Kong: a case-control study | Hong Kong Med J |  |
| **63** | Chang, C. C., Chung, Y. H., Liou, C. B., Lee, Y. C., Weng, W. L., Yu, Y. C., Yen, T. H. and Wu, J. M. | 2015 | Influence of residential environment and lifestyle on multiple primary malignancies in Taiwan | Asian Pac J Cancer Prev | 10.7314/apjcp.2015.16.8.3533 |
| **64** | Chang, W. L., Lin, M. Y., Kuo, H. Y., Yang, H. B., Cheng, H. C., Lu, C. C. and Sheu, B. S. | 2017 | Osteopontin polymorphism increases gastric precancerous intestinal metaplasia susceptibility in Helicobacter pylori infected male | Future Oncol | 10.2217/fon-2017-0006 |
| **65** | Changela, K. and Reddy, M. | 2017 | Smoker's melanosis: Isolated pigmented lesion in the laryngopharynx and esophagus | Turk J Gastroenterol | 10.5152/tjg.2017.17186 |
| **66** | Chatila, A. T., Nguyen, M. T. T., Krill, T., Roark, R., Bilal, M. and Reep, G. | 2020 | Natural history, pathophysiology and evaluation of gastroesophageal reflux disease | Dis Mon | 10.1016/j.disamonth.2019.02.001 |
| **67** | Chatzopoulos, G. S., Cisneros, A., Sanchez, M. and Wolff, L. F. | 2018 | Systemic medical conditions and periodontal status in older individuals | Spec Care Dentist | 10.1111/scd.12319 |
| **68** | Chen, W., Chen, S., Zhao, L., Zhang, M., Geng, H., Dong, C. and Li, R. | 2022 | Effects of real-ambient PM(2.5) exposure plus lipopolysaccharide on multiple organ damage in mice | Hum Exp Toxicol | 10.1177/09603271211061505 |
| **69** | Chen, W. C., Bye, H., Matejcic, M., Amar, A., Govender, D., Khew, Y. W., Beynon, V., Kerr, R., Singh, E., Prescott, N. J., Lewis, C. M., Babb de Villiers, C., Parker, M. I. and Mathew, C. G. | 2019 | Association of genetic variants in CHEK2 with oesophageal squamous cell carcinoma in the South African Black population | Carcinogenesis | 10.1093/carcin/bgz026 |
| **70** | Chetwood, J. D., Garg, P., Finch, P. and Gordon, M. | 2019 | Systematic review: the etiology of esophageal squamous cell carcinoma in low-income settings | Expert Rev Gastroenterol Hepatol | 10.1080/17474124.2019.1543024 |
| **71** | Choi, J. H., Lee, J., Choi, I. J., Kim, Y. W., Ryu, K. W. and Kim, J. | 2016 | Genetic Variation in the TAS2R38 Bitter Taste Receptor and Gastric Cancer Risk in Koreans | Sci Rep | 10.1038/srep26904 |
| **72** | Choi, Y. J., Lee, D. H., Han, K. D., Yoon, H., Shin, C. M., Park, Y. S. and Kim, N. | 2017 | Elevated serum gamma-glutamyltransferase is associated with an increased risk of oesophageal carcinoma in a cohort of 8,388,256 Korean subjects | PLoS One | 10.1371/journal.pone.0177053 |
| **73** | Chuang, Y. S., Wu, M. C., Wang, Y. K., Chen, Y. H., Kuo, C. H., Wu, D. C., Wu, M. T. and Wu, I. C. | 2019 | Risks of substance uses, alcohol flush response, Helicobacter pylori infection and upper digestive tract diseases-An endoscopy cross-sectional study | Kaohsiung J Med Sci | 10.1002/kjm2.12071 |
| **74** | Chubachi, S., Sato, M., Kameyama, N., Tsutsumi, A., Sasaki, M., Tateno, H., Nakamura, H., Asano, K. and Betsuyaku, T. | 2016 | Identification of five clusters of comorbidities in a longitudinal Japanese chronic obstructive pulmonary disease cohort | Respir Med | 10.1016/j.rmed.2016.07.002 |
| **75** | Chung, H. H., Kim, K. O., Lee, S. H., Jang, B. I. and Kim, T. N. | 2017 | Frequency and risk factors of colorectal adenoma in patients with early gastric cancer | Intern Med J | 10.1111/imj.13542 |
| **76** | Çolak, Y., Nordestgaard, B. G., Laursen, L. C., Afzal, S., Lange, P. and Dahl, M. | 2017 | Risk Factors for Chronic Cough Among 14,669 Individuals From the General Population | Chest | 10.1016/j.chest.2017.05.038 |
| **77** | Coleman, H. G., Xie, S. H. and Lagergren, J. | 2018 | The Epidemiology of Esophageal Adenocarcinoma | Gastroenterology | 10.1053/j.gastro.2017.07.046 |
| **78** | Collatuzzo, G., Pelucchi, C., Negri, E., López-Carrillo, L., Tsugane, S., Hidaka, A., Shigueaki Hamada, G., Hernández-Ramírez, R. U., López-Cervantes, M., Malekzadeh, R., Pourfarzi, F., Mu, L., Zhang, Z. F., Lunet, N., La Vecchia, C. and Boffetta, P. | 2021 | Exploring the interactions between Helicobacter pylori (Hp) infection and other risk factors of gastric cancer: A pooled analysis in the Stomach cancer Pooling (StoP) Project | Int J Cancer | 10.1002/ijc.33678 |
| **79** | Cook, M. B., Barnett, M. J., Bock, C. H., Cross, A. J., Goodman, P. J., Goodman, G. E., Haiman, C. A., Khaw, K. T., McCullough, M. L., Newton, C. C., Boutron-Ruault, M. C., Lund, E., Rutegård, M., Thornquist, M. D., Spriggs, M., Giffen, C., Freedman, N. D., Kemp, T., Kroenke, C. H., Le Marchand, L., Park, J. Y., Simon, M., Wilkens, L. R., Pinto, L., Hildesheim, A. and Campbell, P. T. | 2019 | Prediagnostic circulating markers of inflammation and risk of oesophageal adenocarcinoma: a study within the National Cancer Institute Cohort Consortium | Gut | 10.1136/gutjnl-2018-316678 |
| **80** | Cook, M. B., Corley, D. A., Murray, L. J., Liao, L. M., Kamangar, F., Ye, W., Gammon, M. D., Risch, H. A., Casson, A. G., Freedman, N. D., Chow, W. H., Wu, A. H., Bernstein, L., Nyrén, O., Pandeya, N., Whiteman, D. C. and Vaughan, T. L. | 2014 | Gastroesophageal reflux in relation to adenocarcinomas of the esophagus: a pooled analysis from the Barrett's and Esophageal Adenocarcinoma Consortium (BEACON) | PLoS One | 10.1371/journal.pone.0103508 |
| **81** | Crews, N. R., Johnson, M. L., Schleck, C. D., Enders, F. T., Wongkeesong, L. M., Wang, K. K., Katzka, D. A. and Iyer, P. G. | 2016 | Prevalence and Predictors of Gastroesophageal Reflux Complications in Community Subjects | Dig Dis Sci | 10.1007/s10620-016-4266-3 |
| **82** | Dąbrowska, M., Grabczak, E. M., Arcimowicz, M., Domeracka-Kołodziej, A., Domagała-Kulawik, J., Krenke, R., Maskey-Warzęchowska, M., Tarchalska, B. and Chazan, R. | 2015 | Causes of Chronic Cough in Non-smoking Patients | Adv Exp Med Biol | 10.1007/5584_2015_153 |
| **83** | Dahiya, D. S., Kichloo, A., Shaka, H., Singh, J., Edigin, E., Solanki, D., Eseaton, P. O. and Wani, F. | 2021 | Gastroparesis with Cannabis Use: A Retrospective Study from the Nationwide Inpatient Sample | Postgrad Med | 10.1080/00325481.2021.1940219 |
| **84** | Dai, J. Y., Tapsoba Jde, D., Buas, M. F., Risch, H. A. and Vaughan, T. L. | 2016 | Constrained Score Statistics Identify Genetic Variants Interacting with Multiple Risk Factors in Barrett's Esophagus | Am J Hum Genet | 10.1016/j.ajhg.2016.06.018 |
| **85** | Dai, Q., Cantwell, M. M., Murray, L. J., Zheng, W., Anderson, L. A. and Coleman, H. G. | 2016 | Dietary magnesium, calcium:magnesium ratio and risk of reflux oesophagitis, Barrett's oesophagus and oesophageal adenocarcinoma: a population-based case-control study | Br J Nutr | 10.1017/s0007114515004444 |
| **86** | Datta, K. K., Patil, S., Patel, K., Babu, N., Raja, R., Nanjappa, V., Mangalaparthi, K. K., Dhaka, B., Rajagopalan, P., Deolankar, S. C., Kannan, R., Kumar, P., Prasad, T. S. K., Mathur, P. P., Kumari, A., Manoharan, M., Coral, K., Murugan, S., Sidransky, D., Gupta, R., Gupta, R., Khanna-Gupta, A., Chatterjee, A. and Gowda, H. | 2019 | Chronic Exposure to Chewing Tobacco Induces Metabolic Reprogramming and Cancer Stem Cell-Like Properties in Esophageal Epithelial Cells | Cells | 10.3390/cells8090949 |
| **87** | de-Tomás, J. and Monturiol, J. M. | 2016 | Is there any relationship between drug addiction and the development of a signet ring cell carcinoma of the stomach? | Rev Esp Enferm Dig | 10.17235/reed.2016.4070/2015 |
| **88** | DelRosso, L. M. | 2015 | A 3-month-old infant with recurrent apparent life-threatening events in a car seat | Chest | 10.1378/chest.14-1595 |
| **89** | Deng, J., Zhang, J., Wang, Ch, Wei, Q., Zhou, D. and Zhao, K. | 2016 | Methylation and expression of PTPN22 in esophageal squamous cell carcinoma | Oncotarget | 10.18632/oncotarget.11581 |
| **90** | Deng, N., Liu, J. W., Sun, L. P., Xu, Q., Duan, Z. P., Dong, N. N. and Yuan, Y. | 2014 | Expression of XPG protein in the development, progression and prognosis of gastric cancer | PLoS One | 10.1371/journal.pone.0108704 |
| **91** | Ding, H., Duan, Z., Yang, D., Zhang, Z., Wang, L., Sun, X., Yao, Y., Lin, X., Yang, H., Wang, S. and Chen, J. D. Z. | 2017 | High-resolution manometry in patients with and without globus pharyngeus and/or symptoms of laryngopharyngeal reflux | BMC Gastroenterol | 10.1186/s12876-017-0666-x |
| **92** | DiSiena, M., Perelman, A., Birk, J. and Rezaizadeh, H. | 2021 | Esophageal Cancer: An Updated Review | South Med J | 10.14423/smj.0000000000001226 |
| **93** | Dittrich, L., Schwenninger, M. V., Dittrich, K., Pratschke, J., Aigner, F. and Raakow, J. | 2020 | Marginal ulcers after laparoscopic Roux-en-Y gastric bypass: analysis of the amount of daily and lifetime smoking on postoperative risk | Surg Obes Relat Dis | 10.1016/j.soard.2019.11.022 |
| **94** | Djeddi, D., Stephan-Blanchard, E., Léké, A., Ammari, M., Delanaud, S., Lemaire-Hurtel, A. S., Bach, V. and Telliez, F. | 2018 | Effects of Smoking Exposure in Infants on Gastroesophageal Reflux as a Function of the Sleep-Wakefulness State | J Pediatr | 10.1016/j.jpeds.2018.05.057 |
| **95** | Dong, J. and Thrift, A. P. | 2017 | Alcohol, smoking and risk of oesophago-gastric cancer | Best Pract Res Clin Gastroenterol | 10.1016/j.bpg.2017.09.002 |
| **96** | Dong, Y., Chen, J., Chen, Z., Tian, C., Lu, H., Ruan, J. and Yang, W. | 2015 | Evaluating the Association of Eight Polymorphisms with Cancer Susceptibility in a Han Chinese Population | PLoS One | 10.1371/journal.pone.0132797 |
| **97** | Dore, M. P., Pes, G. M., Bassotti, G., Farina, M. A., Marras, G. and Graham, D. Y. | 2016 | Risk factors for erosive and non-erosive gastroesophageal reflux disease and Barrett's esophagus in Nothern Sardinia | Scand J Gastroenterol | 10.1080/00365521.2016.1200137 |
| **98** | Drahos, J., Xiao, Q., Risch, H. A., Freedman, N. D., Abnet, C. C., Anderson, L. A., Bernstein, L., Brown, L., Chow, W. H., Gammon, M. D., Kamangar, F., Liao, L. M., Murray, L. J., Ward, M. H., Ye, W., Wu, A. H., Vaughan, T. L., Whiteman, D. C. and Cook, M. B. | 2016 | Age-specific risk factor profiles of adenocarcinomas of the esophagus: A pooled analysis from the international BEACON consortium | Int J Cancer | 10.1002/ijc.29688 |
| **99** | Dugué, P. A., Bassett, J. K., Wong, E. M., Joo, J. E., Li, S., Yu, C., Schmidt, D. F., Makalic, E., Doo, N. W., Buchanan, D. D., Hodge, A. M., English, D. R., Hopper, J. L., Giles, G. G., Southey, M. C. and Milne, R. L. | 2021 | Biological Aging Measures Based on Blood DNA Methylation and Risk of Cancer: A Prospective Study | JNCI Cancer Spectr | 10.1093/jncics/pkaa109 |
| **100** | Duvvuri, P. D., Bhardwaj, C. and Wattanakit, K. | 2021 | A 50-Year-Old Woman With Shortness of Breath | Chest | 10.1016/j.chest.2020.10.098 |
| **101** | Dzahini, O., Singh, N., Taylor, D. and Haddad, P. M. | 2018 | Antipsychotic drug use and pneumonia: Systematic review and meta-analysis | J Psychopharmacol | 10.1177/0269881118795333 |
| **102** | Eisenberg, J. D., Rosato, E. L., Lavu, H., Yeo, C. J. and Winter, J. M. | 2015 | Delayed Gastric Emptying After Pancreaticoduodenectomy: an Analysis of Risk Factors and Cost | J Gastrointest Surg | 10.1007/s11605-015-2865-5 |
| **103** | El Khadir, M., Boukhris Alaoui, S., Benajah, D. A., Ibrahimi, S. A., Chbani, L., El Abkari, M. and Bennani, B. | 2020 | VacA genotypes and cagA-EPIYA-C motifs of Helicobacter pylori and gastric histopathological lesions | Int J Cancer | 10.1002/ijc.33158 |
| **104** | Elsamadicy, A. A., Adogwa, O., Sergesketter, A., Vuong, V. D., Lydon, E., Behrens, S., Cheng, J., Bagley, C. A. and Karikari, I. O. | 2017 | Reduced Impact of Smoking Status on 30-Day Complication and Readmission Rates After Elective Spinal Fusion (≥3 Levels) for Adult Spine Deformity: A Single Institutional Study of 839 Patients | World Neurosurg | 10.1016/j.wneu.2017.07.174 |
| **105** | Enderes, J., Teschke, J., von Websky, M., Manekeller, S., Kalff, J. C. and Glowka, T. R. | 2021 | Active smokers show ameliorated delayed gastric emptying after pancreatoduodenectomy | BMC Surg | 10.1186/s12893-021-01311-2 |
| **106** | Endo, K., Nakada, H., Kadota, Y., Mizutani, Y., Shinkawa, N., Onoe, K., Yoshinaga, N., Azuma, M. and Hirai, T. | 2018 | Risk factors for atrophic gastritis in the Japanese young and middle-aged: a study using double-contrast upper gastrointestinal barium X-ray radiography | Jpn J Radiol | 10.1007/s11604-018-0782-8 |
| **107** | Esteban Ronda, V., Franco Serrano, J. and Briones Urtiaga, M. L. | 2016 | Pulmonary Strongyloides stercoralis infection | Arch Bronconeumol | 10.1016/j.arbres.2016.01.010 |
| **108** | Etemadi, A., Gandomkar, A., Freedman, N. D., Moghadami, M., Fattahi, M. R., Poustchi, H., Islami, F., Boffetta, P., Dawsey, S. M., Abnet, C. C. and Malekzadeh, R. | 2017 | The association between waterpipe smoking and gastroesophageal reflux disease | Int J Epidemiol | 10.1093/ije/dyx158 |
| **109** | Eusebi, L. H., Ratnakumaran, R., Yuan, Y., Solaymani-Dodaran, M., Bazzoli, F. and Ford, A. C. | 2018 | Global prevalence of, and risk factors for, gastro-oesophageal reflux symptoms: a meta-analysis | Gut | 10.1136/gutjnl-2016-313589 |
| **110** | Eusebi, L. H., Telese, A., Marasco, G., Bazzoli, F. and Zagari, R. M. | 2020 | Gastric cancer prevention strategies: A global perspective | J Gastroenterol Hepatol | 10.1111/jgh.15037 |
| **111** | Fahey, P. P., Mallitt, K. A., Astell-Burt, T., Stone, G. and Whiteman, D. C. | 2015 | Impact of pre-diagnosis behavior on risk of death from esophageal cancer: a systematic review and meta-analysis | Cancer Causes Control | 10.1007/s10552-015-0635-z |
| **112** | Fahey, P. P., Page, A., Stone, G. and Astell-Burt, T. | 2020 | Augmenting cancer registry data with health survey data with no cases in common: the relationship between pre-diagnosis health behaviour and post-diagnosis survival in oesophageal cancer | BMC Cancer | 10.1186/s12885-020-06990-3 |
| **113** | Ferro, A., Morais, S., Pelucchi, C., Aragonés, N., Kogevinas, M., López-Carrillo, L., Malekzadeh, R., Tsugane, S., Hamada, G. S., Hidaka, A., Hernández-Ramírez, R. U., López-Cervantes, M., Zaridze, D., Maximovitch, D., Pourfarzi, F., Zhang, Z. F., Yu, G. P., Pakseresht, M., Ye, W., Plymoth, A., Leja, M., Gasenko, E., Derakhshan, M. H., Negri, E., La Vecchia, C., Peleteiro, B. and Lunet, N. | 2019 | Smoking and Helicobacter pylori infection: an individual participant pooled analysis (Stomach Cancer Pooling- StoP Project) | Eur J Cancer Prev | 10.1097/cej.0000000000000471 |
| **114** | Ferro, A., Morais, S., Rota, M., Pelucchi, C., Bertuccio, P., Bonzi, R., Galeone, C., Zhang, Z. F., Matsuo, K., Ito, H., Hu, J., Johnson, K. C., Yu, G. P., Palli, D., Ferraroni, M., Muscat, J., Malekzadeh, R., Ye, W., Song, H., Zaridze, D., Maximovitch, D., Aragonés, N., Castaño-Vinyals, G., Vioque, J., Navarrete-Muñoz, E. M., Pakseresht, M., Pourfarzi, F., Wolk, A., Orsini, N., Bellavia, A., Håkansson, N., Mu, L., Pastorino, R., Kurtz, R. C., Derakhshan, M. H., Lagiou, A., Lagiou, P., Boffetta, P., Boccia, S., Negri, E., La Vecchia, C., Peleteiro, B. and Lunet, N. | 2018 | Tobacco smoking and gastric cancer: meta-analyses of published data versus pooled analyses of individual participant data (StoP Project) | Eur J Cancer Prev | 10.1097/cej.0000000000000401 |
| **115** | Filiberti, R., Fontana, V., De Ceglie, A., Blanchi, S., Grossi, E., Della Casa, D., Lacchin, T., De Matthaeis, M., Ignomirelli, O., Cappiello, R., Foti, M., Laterza, F., Annese, V., Iaquinto, G. and Conio, M. | 2015 | Smoking as an independent determinant of Barrett's esophagus and, to a lesser degree, of reflux esophagitis | Cancer Causes Control | 10.1007/s10552-014-0518-8 |
| **116** | Floud, S., Hermon, C., Simpson, R. F. and Reeves, G. K. | 2023 | Alcohol consumption and cancer incidence in women: interaction with smoking, body mass index and menopausal hormone therapy | BMC Cancer | 10.1186/s12885-023-11184-8 |
| **117** | Fu, C. K., Mong, M. C., Tzeng, H. E., Yang, M. D., Chen, J. C., Hsia, T. C., Hsia, N. Y., Tsai, C. W., Chang, W. S., Chen, C. P. and Bau, D. T. | 2024 | The Significant Contribution of Interleukin-16 Genotypes, Smoking, Alcohol Drinking, and Helicobacter Pylori Infection to Gastric Cancer | In Vivo | 10.21873/invivo.13414 |
| **118** | Fukai, K., Kojimahara, N., Hoshi, K., Toyota, A. and Tatemichi, M. | 2020 | Combined effects of occupational exposure to hazardous operations and lifestyle-related factors on cancer incidence | Cancer Sci | 10.1111/cas.14663 |
| **119** | Gaetti-Jardim, E., Jr., Jardim, E. C. G., Schweitzer, C. M., da Silva, J. C. L., Oliveira, M. M., Masocatto, D. C. and Dos Santos, C. M. | 2018 | Supragingival and subgingival microbiota from patients with poor oral hygiene submitted to radiotherapy for head and neck cancer treatment | Arch Oral Biol | 10.1016/j.archoralbio.2018.01.003 |
| **120** | Gallagher, L. G., Li, W., Ray, R. M., Romano, M. E., Wernli, K. J., Gao, D. L., Thomas, D. B. and Checkoway, H. | 2015 | Occupational exposures and risk of stomach and esophageal cancers: update of a cohort of female textile workers in Shanghai, China | Am J Ind Med | 10.1002/ajim.22412 |
| **121** | Gallegos-Arreola, M. P., Zúñiga-González, G. M., Sánchez-López, J. Y., Cruz, A. Y. N., Peralta-Leal, V., Figuera, L. E., Puebla-Pérez, A. M., Ronquillo-Carreón, C. A. and Puebla-Mora, A. G. | 2018 | TYMS 2R3R polymorphism and DPYD [IVS]14+1G>A gene mutation in Mexican colorectal cancer patients | Acta Biochim Pol | 10.18388/abp.2017_2338 |
| **122** | Gallo, O., Locatello, L. G., Larotonda, G., Napoleone, V. and Cannavicci, A. | 2018 | Nomograms for prediction of postoperative complications in open partial laryngeal surgery | J Surg Oncol | 10.1002/jso.25232 |
| **123** | Galway, N. C. and Shields, M. D. | 2019 | The child with an incessant dry cough | Paediatr Respir Rev | 10.1016/j.prrv.2018.08.002 |
| **124** | Gao, S., Li, J., Feng, X., Shi, S. and He, J. | 2016 | Characteristics and Surgical Outcomes for Primary Malignant Melanoma of the Esophagus | Sci Rep | 10.1038/srep23804 |
| **125** | Gasenko, E., Isajevs, S., Camargo, M. C., Offerhaus, G. J. A., Polaka, I., Gulley, M. L., Skapars, R., Sivins, A., Kojalo, I., Kirsners, A., Santare, D., Pavlova, J., Sjomina, O., Liepina, E., Tzivian, L., Rabkin, C. S. and Leja, M. | 2019 | Clinicopathological characteristics of Epstein-Barr virus-positive gastric cancer in Latvia | Eur J Gastroenterol Hepatol | 10.1097/meg.0000000000001521 |
| **126** | Gasmelseed, N., Abudris, D., Elhaj, A., Eltayeb, E. A., Elmadani, A., Elhassan, M. M., Mohammed, K., Elgaili, E. M., Elbalal, M., Schuz, J. and Leon, M. E. | 2015 | Patterns of Esophageal Cancer in the National Cancer Institute at the University of Gezira, in Gezira State, Sudan, in 1999-2012 | Asian Pac J Cancer Prev | 10.7314/apjcp.2015.16.15.6481 |
| **127** | Ghosh, P., Alam, N., Mandal, S., Mustafi, S. M. and Murmu, N. | 2020 | Association of mTOR pathway with risk of gastric cancer in male smoker with potential prognostic significance | Mol Biol Rep | 10.1007/s11033-020-05808-6 |
| **128** | Ghoshal, U. C., Singh, R. and Rai, S. | 2021 | Prevalence and risk factors of gastroesophageal reflux disease in a rural Indian population | Indian J Gastroenterol | 10.1007/s12664-020-01135-7 |
| **129** | Gil, G. F., Anderson, J. A., Aravkin, A., Bhangdia, K., Carr, S., Dai, X., Flor, L. S., Hay, S. I., Malloy, M. J., McLaughlin, S. A., Mullany, E. C., Murray, C. J. L., O'Connell, E. M., Okereke, C., Sorensen, R. J. D., Whisnant, J., Zheng, P. and Gakidou, E. | 2024 | Health effects associated with chewing tobacco: a Burden of Proof study | Nat Commun | 10.1038/s41467-024-45074-9 |
| **130** | Gill, S.S. and Thakkar, S. | 2013 | Images of the month: Arytenoid swelling causing dysphagia secondary to ingestion of a hot object | Am J Gastroenterol | 10.1038/ajg.2013.29 |
| **131** | Giraldi, L., Stojanovic, J., Arzani, D., Persiani, R., Hu, J., Johnson, K. C., Zhang, Z. F., Ferraroni, M., Palli, D., Yu, G. P., La Vecchia, C., Pelucchi, C., Lunet, N., Ferro, A., Malekzadeh, R., Muscat, J., Zaridze, D., Maximovich, D., Aragones, N., Martin, V., Vioque, J., Navarrete-Munoz, E. M., Pakseresht, M., Negri, E., Rota, M., Pourfarzi, F., Mu, L., Kurtz, R. C., Lagiou, A., Lagiou, P., Pastorino, R. and Boccia, S. | 2023 | Adult height and risk of gastric cancer: a pooled analysis within the Stomach cancer Pooling Project | Eur J Cancer Prev | 10.1097/cej.0000000000000613 |
| **132** | Global Burden of Disease (GBD) 2017 Oesophageal Cancer Collaborators | 2020 | The global, regional, and national burden of oesophageal cancer and its attributable risk factors in 195 countries and territories, 1990-2017: a systematic analysis for the Global Burden of Disease Study 2017 | Lancet Gastroenterol Hepatol | 10.1016/s2468-1253(20)30007-8 |
| **133** | Golpe, R., Martín-Robles, I., Sanjuán-López, P., Cano-Jiménez, E., Castro-Añon, O., Mengual-Macenlle, N. and Pérez-de-Llano, L. | 2017 | Prevalence of Major Comorbidities in Chronic Obstructive Pulmonary Disease Caused by Biomass Smoke or Tobacco | Respiration | 10.1159/000472718 |
| **134** | Gong, E. J., Kim, D. H., Jung, H. Y., Lim, H., Ahn, J. Y., Choi, K. S., Lee, J. H., Choi, K. D., Song, H. J., Lee, G. H., Kim, J. H. and Baek, S. | 2014 | Pneumonia after endoscopic resection for gastric neoplasm | Dig Dis Sci | 10.1007/s10620-014-3223-2 |
| **135** | Gong, J., Chu, Y., Xu, M., Huo, J. and Lv, L. | 2016 | Esophageal squamous cell carcinoma cell proliferation induced by exposure to low concentration of cigarette smoke extract is mediated via targeting miR-101-3p/COX-2 pathway | Oncol Rep | 10.3892/or.2015.4379 |
| **136** | Goto, H., Oshikiri, T., Kato, T., Sawada, R., Harada, H., Urakawa, N., Hasegawa, H., Kanaji, S., Yamashita, K., Matsuda, T. and Kakeji, Y. | 2023 | The Influence of Preoperative Smoking Status on Postoperative Complications and Long-Term Outcome Following Thoracoscopic Esophagectomy in Prone Position for Esophageal Carcinoma | Ann Surg Oncol | 10.1245/s10434-022-12898-y |
| **137** | Grossman, K., Beasley, M. B. and Braman, S. S. | 2016 | Hepatoid adenocarcinoma of the lung: Review of a rare form of lung cancer | Respir Med | 10.1016/j.rmed.2016.09.003 |
| **138** | Grøtting, M. S., Løberg, E. M., Johannessen, H. O. and Johnson, E. | 2016 | Resection for oesophageal cancer - complications and survival | Tidsskr Nor Laegeforen | 10.4045/tidsskr.15.1136 |
| **139** | Haider, S. H., Kwon, S., Lam, R., Lee, A. K., Caraher, E. J., Crowley, G., Zhang, L., Schwartz, T. M., Zeig-Owens, R., Liu, M., Prezant, D. J. and Nolan, A. | 2018 | Predictive Biomarkers of Gastroesophageal Reflux Disease and Barrett's Esophagus in World Trade Center Exposed Firefighters: a 15 Year Longitudinal Study | Sci Rep | 10.1038/s41598-018-21334-9 |
| **140** | Hammad, T. A., Thrift, A. P., El-Serag, H. B. and Husain, N. S. | 2019 | Missed Opportunities for Screening and Surveillance of Barrett's Esophagus in Veterans with Esophageal Adenocarcinoma | Dig Dis Sci | 10.1007/s10620-018-5336-5 |
| **141** | Hamzaoui, L., Bouassida, M., Ben Mansour, I., Medhioub, M., Ezzine, H., Touinsi, H. and Azouz, M. M. | 2015 | Balloon dilatation in patients with gastric outlet obstruction related to peptic ulcer disease | Arab J Gastroenterol | 10.1016/j.ajg.2015.07.004 |
| **142** | Han, R., Chen, G., Li, M., Peng, Z. M. and Xu, L. | 2021 | Screening and clinical significance of lymph node metastasis-related genes within esophagogastric junction adenocarcinoma | Cancer Med | 10.1002/cam4.4065 |
| **143** | Hanu, C., Timotin, E., Wong, R., Sur, R. K., Hayward, J. E., Seymour, C. B. and Mothersill, C. E. | 2016 | The influence of smoking on radiation-induced bystander signal production in esophageal cancer patients | Environ Res | 10.1016/j.envres.2015.12.030 |
| **144** | Hashibe, M., Morgenstern, H., Cui, Y., Tashkin, D. P., Zhang, Z. F., Cozen, W., Mack, T. M. and Greenland, S. | 2006 | Marijuana use and the risk of lung and upper aerodigestive tract cancers: results of a population-based case-control study | Cancer Epidemiol Biomarkers Prev | 10.1158/1055-9965.Epi-06-0330 |
| **145** | Hatta, W., Koike, T., Asonuma, S., Okata, H., Uno, K., Oikawa, T., Iwai, W., Yonechi, M., Fukushi, D., Kayaba, S., Kikuchi, R., Ohyauchi, M., Fushiya, J., Maejima, R., Abe, Y., Kawamura, M., Honda, J., Kondo, Y., Dairaku, N., Norita, K., Watanabe, K., Takahashi, K., Echigo, H., Abe, Y., Endo, H., Okata, T., Hoshi, T., Nakamura, T., Nakaya, N., Iijima, K. and Masamune, A. | 2023 | Smoking history and severe atrophic gastritis assessed by pepsinogen are risk factors for the prevalence of synchronous gastric cancers in patients with gastric endoscopic submucosal dissection: a multicenter prospective cohort study | J Gastroenterol | 10.1007/s00535-023-01967-y |
| **146** | He, Y. T., Christos, P. J. and Reisacher, W. R. | 2018 | Airborne and food sensitization patterns in children and adults with eosinophilic esophagitis | Int Forum Allergy Rhinol | 10.1002/alr.22095 |
| **147** | Hekking, P. P., Amelink, M., Wener, R. R., Bouvy, M. L. and Bel, E. H. | 2018 | Comorbidities in Difficult-to-Control Asthma | J Allergy Clin Immunol Pract | 10.1016/j.jaip.2017.06.008 |
| **148** | Henry, M. A., Lerco, M. M., Ribeiro, P. W. and Rodrigues, M. A. | 2014 | Epidemiological features of esophageal cancer. Squamous cell carcinoma versus adenocarcinoma | Acta Cir Bras | 10.1590/s0102-86502014000600007 |
| **149** | Herout, K. T., Durant, E. J. and Fong, J. | 2021 | Dysphagia as the Predominant Symptom in Posterior Circulation Stroke: A Case Report | Am J Case Rep | 10.12659/ajcr.930502 |
| **150** | Hill, D. A. and Spergel, J. M. | 2018 | Is eosinophilic esophagitis a member of the atopic march? | Ann Allergy Asthma Immunol | 10.1016/j.anai.2017.10.003 |
| **151** | Hirabayashi, M., Inoue, M., Sawada, N., Saito, E., Abe, S. K., Hidaka, A., Iwasaki, M., Yamaji, T., Shimazu, T. and Tsugane, S. | 2019 | Helicobacter pylori infection, atrophic gastritis, and risk of pancreatic cancer: A population-based cohort study in a large Japanese population: the JPHC Study | Sci Rep | 10.1038/s41598-019-42365-w |
| **152** | Ho, T. W., Tsai, Y. J., Huang, C. T., Lien, A. S. and Lai, F. | 2020 | Impact of tobacco-related chronic obstructive pulmonary disease on developmental trajectories of comorbidities in the Taiwan population | Sci Rep | 10.1038/s41598-020-78325-y |
| **153** | Horiuchi, Y., Fujisaki, J., Ishizuka, N., Omae, M., Ishiyama, A., Yoshio, T., Hirasawa, T., Yamamoto, Y., Nagahama, M., Takahashi, H. and Tsuchida, T. | 2017 | Study on Clinical Factors Involved in Helicobacter pylori-Uninfected, Undifferentiated-Type Early Gastric Cancer | Digestion | 10.1159/000481817 |
| **154** | Houghton, L. A., Lee, A. S., Badri, H., DeVault, K. R. and Smith, J. A. | 2016 | Respiratory disease and the oesophagus: reflux, reflexes and microaspiration | Nat Rev Gastroenterol Hepatol | 10.1038/nrgastro.2016.91 |
| **155** | Hsu, C. C., Hsu, Y. C., Chang, K. H., Lee, C. Y., Chong, L. W., Lin, C. L., Shang, C. S., Sung, F. C. and Kao, C. H. | 2015 | Depression and the Risk of Peptic Ulcer Disease: A Nationwide Population-Based Study | Medicine (Baltimore) | 10.1097/md.0000000000002333 |
| **156** | Huang, I. H., Schol, J., Khatun, R., Carbone, F., Van den Houte, K., Colomier, E., Balsiger, L. M., Törnblom, H., Vanuytsel, T., Sundelin, E., Simrén, M., Palsson, O. S., Bangdiwala, S. I., Sperber, A. D. and Tack, J. | 2022 | Worldwide prevalence and burden of gastroparesis-like symptoms as defined by the United European Gastroenterology (UEG) and European Society for Neurogastroenterology and Motility (ESNM) consensus on gastroparesis | United European Gastroenterol J | 10.1002/ueg2.12289 |
| **157** | Huang, Z. S., Chen, W. L., Huang, Z. Q. and Yang, Z. H. | 2016 | Dysphagia in Tongue Cancer Patients Before and After Surgery | J Oral Maxillofac Surg | 10.1016/j.joms.2016.03.031 |
| **158** | Hudler, P. | 2015 | Challenges of deciphering gastric cancer heterogeneity | World J Gastroenterol | 10.3748/wjg.v21.i37.10510 |
| **159** | Ireland, C. J., Thompson, S. K., Laws, T. A. and Esterman, A. | 2016 | Risk factors for Barrett's esophagus: a scoping review | Cancer Causes Control | 10.1007/s10552-015-0710-5 |
| **160** | Ishioka, K., Masaoka, H., Ito, H., Oze, I., Ito, S., Tajika, M., Shimizu, Y., Niwa, Y., Nakamura, S. and Matsuo, K. | 2018 | Association between ALDH2 and ADH1B polymorphisms, alcohol drinking and gastric cancer: a replication and mediation analysis | Gastric Cancer | 10.1007/s10120-018-0823-0 |
| **161** | Janson, C., Johannessen, A., Franklin, K., Svanes, C., Schiöler, L., Malinovschi, A., Gislason, T., Benediktsdottir, B., Schlünssen, V., Jõgi, R., Jarvis, D. and Lindberg, E. | 2018 | Change in the prevalence asthma, rhinitis and respiratory symptom over a 20 year period: associations to year of birth, life style and sleep related symptoms | BMC Pulm Med | 10.1186/s12890-018-0690-9 |
| **162** | Jayalekshmi, P. A., Hassani, S., Nandakumar, A., Koriyama, C., Sebastian, P. and Akiba, S. | 2015 | Gastric cancer risk in relation to tobacco use and alcohol drinking in Kerala, India--Karunagappally cohort study | World J Gastroenterol | 10.3748/wjg.v21.i44.12676 |
| **163** | Jayalekshmi, P. A., Nandakumar, A., Nair, R. A., Akiba, S. and Koriyama, C. | 2021 | Esophageal cancer in relation to alcohol drinking and tobacco use among men in Kerala, India - Karunagappally cohort | Cancer Epidemiol | 10.1016/j.canep.2021.102018 |
| **164** | Jayasekara, H., MacInnis, R. J., Lujan-Barroso, L., Mayen-Chacon, A. L., Cross, A. J., Wallner, B., Palli, D., Ricceri, F., Pala, V., Panico, S., Tumino, R., Kühn, T., Kaaks, R., Tsilidis, K., Sánchez, M. J., Amiano, P., Ardanaz, E., Chirlaque López, M. D., Merino, S., Rothwell, J. A., Boutron-Ruault, M. C., Severi, G., Sternby, H., Sonestedt, E., Bueno-de-Mesquita, B., Boeing, H., Travis, R., Sandanger, T. M., Trichopoulou, A., Karakatsani, A., Peppa, E., Tjønneland, A., Yang, Y., Hodge, A. M., Mitchell, H., Haydon, A., Room, R., Hopper, J. L., Weiderpass, E., Gunter, M. J., Riboli, E., Giles, G. G., Milne, R. L., Agudo, A., English, D. R. and Ferrari, P. | 2021 | Lifetime alcohol intake, drinking patterns over time and risk of stomach cancer: A pooled analysis of data from two prospective cohort studies | Int J Cancer | 10.1002/ijc.33504 |
| **165** | Jee, Y. H., Shin, A., Lee, J. K. and Oh, C. M. | 2016 | Decreases in Smoking-Related Cancer Mortality Rates Are Associated with Birth Cohort Effects in Korean Men | Int J Environ Res Public Health | 10.3390/ijerph13121208 |
| **166** | Jehangir, A. and Parkman, H. P. | 2019 | Cannabinoid Use in Patients With Gastroparesis and Related Disorders: Prevalence and Benefit | Am J Gastroenterol | 10.14309/ajg.0000000000000181 |
| **167** | Jeong, S., Jo, M. J., Yun, H. K., Kim, D. Y., Kim, B. R., Kim, J. L., Park, S. H., Na, Y. J., Jeong, Y. A., Kim, B. G., Ashktorab, H., Smoot, D. T., Heo, J. Y., Han, J., Il Lee, S., Do Kim, H., Kim, D. H., Oh, S. C. and Lee, D. H. | 2019 | Cannabidiol promotes apoptosis via regulation of XIAP/Smac in gastric cancer | Cell Death Dis | 10.1038/s41419-019-2001-7 |
| **168** | Jetté, M. E., Dill-McFarland, K. A., Hanshew, A. S., Suen, G. and Thibeault, S. L. | 2016 | The human laryngeal microbiome: effects of cigarette smoke and reflux | Sci Rep | 10.1038/srep35882 |
| **169** | Jetté, M. E., Seroogy, C. M. and Thibeault, S. L. | 2017 | Laryngeal T regulatory cells in the setting of smoking and reflux | Laryngoscope | 10.1002/lary.26223 |
| **170** | Ji, W., Zheng, W., Li, B., Cao, C. and Mao, W. | 2016 | Influence of body mass index on the long-term outcomes of patients with esophageal squamous cell carcinoma who underwent esophagectomy as a primary treatment: A 10-year medical experience | Medicine (Baltimore) | 10.1097/md.0000000000004204 |
| **171** | Jia, X., Sheng, C., Han, X., Li, M. and Wang, K. | 2024 | Global burden of stomach cancer attributable to smoking from 1990 to 2019 and predictions to 2044 | Public Health | 10.1016/j.puhe.2023.11.019 |
| **172** | Jiang, D., Song, Q., Zhang, F., Xu, C., Li, X., Zeng, H., Su, J., Huang, J., Xu, Y., Lu, S. and Hou, Y. | 2023 | Prognostic significance of CCND1 amplification/overexpression in smoking patients with esophageal squamous cell carcinoma | Cancer Genet | 10.1016/j.cancergen.2023.07.004 |
| **173** | Jin, Z. Y., Wallar, G., Zhou, J. Y., Yang, J., Han, R. Q., Wang, P. H., Liu, A. M., Gu, X. P., Zhang, X. F., Wang, X. S., Su, M., Hu, X., Sun, Z., Li, G., Mu, L. N., Lu, Q. Y., Liu, X., Li, L. M., He, N., Wu, M., Zhao, J. K. and Zhang, Z. F. | 2019 | Consumption of garlic and its interactions with tobacco smoking and alcohol drinking on esophageal cancer in a Chinese population | Eur J Cancer Prev | 10.1097/cej.0000000000000456 |
| **174** | Jing, J. J., Sun, L. P., Xu, Q. and Yuan, Y. | 2015 | Effect of ERCC8 tagSNPs and their association with H. pylori infection, smoking, and alcohol consumption on gastric cancer and atrophic gastritis risk | Tumour Biol | 10.1007/s13277-015-3703-9 |
| **175** | Jo, S., Kim, T. J., Lee, H., Min, Y. W., Min, B. H., Lee, J. H., Son, H. J., Rhee, P. L., Baek, S. Y., Kim, S. W. and Kim, J. J. | 2018 | Associations between Atopic Dermatitis and Risk of Gastric Cancer: A Nationwide Population-based Study | Korean J Gastroenterol | 10.4166/kjg.2018.71.1.38 |
| **176** | Johnson, J. A. and Landreneau, R. J. | 1991 | Esophageal obstruction and mediastinitis: a hard pill to swallow for drug smugglers | Am Surg |  |
| **177** | Jung, K. H., Kim, S. M., Choi, M. G., Lee, J. H., Noh, J. H., Sohn, T. S., Bae, J. M. and Kim, S. | 2015 | Preoperative smoking cessation can reduce postoperative complications in gastric cancer surgery | Gastric Cancer | 10.1007/s10120-014-0415-6 |
| **178** | Kakaje, A., Alhalabi, M. M., Alyousbashi, A., Hamid, A. and Mahmoud, Y. | 2020 | Laryngopharyngeal reflux in war-torn Syria and its association with smoking and other risks: an online cross-sectional population study | BMJ Open | 10.1136/bmjopen-2020-041183 |
| **179** | Kamangar, F. and Freedman, N. D. | 2018 | Hot Tea and Esophageal Cancer | Ann Intern Med | 10.7326/m17-3370 |
| **180** | Kamarajah, S. K., Madhavan, A., Chmelo, J., Navidi, M., Wahed, S., Immanuel, A., Hayes, N., Griffin, S. M. and Phillips, A. W. | 2021 | Impact of Smoking Status on Perioperative Morbidity, Mortality, and Long-Term Survival Following Transthoracic Esophagectomy for Esophageal Cancer | Ann Surg Oncol | 10.1245/s10434-021-09720-6 |
| **181** | Kamarajah, S. K. and Phillips, A. W. | 2021 | ASO Author Reflections: Smoking Status Impact on Perioperative Morbidity and Long-Term Survival of Patients Undergoing Esophagectomy for Cancer | Ann Surg Oncol | 10.1245/s10434-021-09765-7 |
| **182** | Kamboj, A. K., Gibbens, Y. Y., Hagen, C. E., Wang, K. K., Iyer, P. G. and Katzka, D. A. | 2021 | Esophageal Epidermoid Metaplasia: Clinical Characteristics and Risk of Esophageal Squamous Neoplasia | Am J Gastroenterol | 10.14309/ajg.0000000000001225 |
| **183** | Kang, H. H., Seo, M., Lee, J., Ha, S. Y., Oh, J. H. and Lee, S. H. | 2021 | Reflux esophagitis in patients with chronic obstructive pulmonary disease | Medicine (Baltimore) | 10.1097/md.0000000000027091 |
| **184** | Kang, M. Y., Jung, J., Koo, J. W., Kim, I., Kim, H. R. and Myong, J. P. | 2021 | Increased risk of gastric cancer in workers with occupational dust exposure | Korean J Intern Med | 10.3904/kjim.2019.421 |
| **185** | Kang, S. H., Lim, Y., Lee, H., Kim, J., Chi, S., Min, Y. W., Min, B. H., Lee, J. H., Son, H. J., Ryu, S., Rhee, P. L. and Kim, J. J. | 2016 | A Model for Predicting the Future Risk of Incident Erosive Esophagitis in an Asymptomatic Population Undergoing Regular Check-ups | Medicine (Baltimore) | 10.1097/md.0000000000002591 |
| **186** | Kawano, H., Saeki, H., Kitao, H., Tsuda, Y., Otsu, H., Ando, K., Ito, S., Egashira, A., Oki, E., Morita, M., Oda, Y. and Maehara, Y. | 2014 | Chromosomal instability associated with global DNA hypomethylation is associated with the initiation and progression of esophageal squamous cell carcinoma | Ann Surg Oncol | 10.1245/s10434-014-3818-z |
| **187** | Kayalı Dinc, A. S., Cayonu, M., Sengezer, T. and Sahin, M. M. | 2020 | Smoking Cessation Improves the Symptoms and the Findings of Laryngeal Irritation | Ear Nose Throat J | 10.1177/0145561319881559 |
| **188** | Kayamba, V., Bateman, A. C., Asombang, A. W., Shibemba, A., Zyambo, K., Banda, T., Soko, R. and Kelly, P. | 2015 | HIV infection and domestic smoke exposure, but not human papillomavirus, are risk factors for esophageal squamous cell carcinoma in Zambia: a case-control study | Cancer Med | 10.1002/cam4.434 |
| **189** | Kayamba, V., Heimburger, D. C., Morgan, D. R., Atadzhanov, M. and Kelly, P. | 2017 | Exposure to biomass smoke as a risk factor for oesophageal and gastric cancer in low-income populations: A systematic review | Malawi Med J | 10.4314/mmj.v29i2.25 |
| **190** | Kayamba, V., Zyambo, K., Mulenga, C., Mwakamui, S., Tembo, M. J., Shibemba, A., Heimburger, D. C., Atadzhanov, M. and Kelly, P. | 2020 | Biomass Smoke Exposure Is Associated With Gastric Cancer and Probably Mediated Via Oxidative Stress and DNA Damage: A Case-Control Study | JCO Glob Oncol | 10.1200/go.20.00002 |
| **191** | Kaz, A. M., Wong, C. J., Varadan, V., Willis, J. E., Chak, A. and Grady, W. M. | 2016 | Global DNA methylation patterns in Barrett's esophagus, dysplastic Barrett's, and esophageal adenocarcinoma are associated with BMI, gender, and tobacco use | Clin Epigenetics | 10.1186/s13148-016-0273-7 |
| **192** | Keene, J. D., Jacobson, S., Kechris, K., Kinney, G. L., Foreman, M. G., Doerschuk, C. M., Make, B. J., Curtis, J. L., Rennard, S. I., Barr, R. G., Bleecker, E. R., Kanner, R. E., Kleerup, E. C., Hansel, N. N., Woodruff, P. G., Han, M. K., Paine, R., 3rd, Martinez, F. J., Bowler, R. P. and O'Neal, W. K. | 2017 | Biomarkers Predictive of Exacerbations in the SPIROMICS and COPDGene Cohorts | Am J Respir Crit Care Med | 10.1164/rccm.201607-1330OC |
| **193** | Khalid, S. I., Eldridge, C., Singh, R., Shanker, R. M., MacDonald, A. M., Chilakapati, S., Smith, J., Mehta, A. I. and Adogwa, O. | 2022 | The impact of smoking and smoking cessation interventions on outcomes following single-level anterior cervical discectomy and fusion procedures | Clin Neurol Neurosurg | 10.1016/j.clineuro.2022.107319 |
| **194** | Khan, T., Relitti, N., Brindisi, M., Magnano, S., Zisterer, D., Gemma, S., Butini, S. and Campiani, G. | 2020 | Autophagy modulators for the treatment of oral and esophageal squamous cell carcinomas | Med Res Rev | 10.1002/med.21646 |
| **195** | Kilbane, K. S., Girgla, N., Zhao, L., Barnett, S. L., Berezovsky, A., Lagisetty, K., Lin, J. and Reddy, R. M. | 2021 | Adaptive and Maladaptive Coping Mechanisms Used by Patients With Esophageal Cancer After Esophagectomy | J Surg Res | 10.1016/j.jss.2020.07.043 |
| **196** | Kim, D. H., Park, J. Y., Karm, M. H., Bae, H. Y., Lee, J. Y., Soo Ahn, H., Lee, K. and Leem, J. G. | 2017 | Smoking May Increase Postoperative Opioid Consumption in Patients Who Underwent Distal Gastrectomy With Gastroduodenostomy for Early Stomach Cancer: A Retrospective Analysis | Clin J Pain | 10.1097/ajp.0000000000000472 |
| **197** | Kim, H. J., Kim, D. K., Sohn, T. S., Lee, J. H. and Lee, G. H. | 2015 | A laparoscopic gastrectomy approach decreases the incidence and severity of emergence agitation after sevoflurane anesthesia | J Anesth | 10.1007/s00540-014-1905-8 |
| **198** | Kim, H. S., Lee, S. E., Bae, Y. S., Kim, D. J., Lee, C. G., Hur, J., Chung, H., Park, J. C., Jung, D. H., Shin, S. K., Lee, S. K., Lee, Y. C., Kim, H. R., Moon, Y. W., Kim, J. H., Shim, Y. M., Jewell, S. S., Kim, H., Choi, Y. L. and Cho, B. C. | 2015 | Fibroblast growth factor receptor 1 gene amplification is associated with poor survival in patients with resected esophageal squamous cell carcinoma | Oncotarget | 10.18632/oncotarget.2944 |
| **199** | Kim, K., Chang, Y., Ahn, J., Yang, H. J., Jung, J. Y., Kim, S., Sohn, C. I. and Ryu, S. | 2019 | Smoking and Urinary Cotinine Levels Are Predictors of Increased Risk for Gastric Intestinal Metaplasia | Cancer Res | 10.1158/0008-5472.Can-18-2268 |
| **200** | Kim, M., Choi, K. S., Suh, M., Jun, J. K., Chuck, K. W. and Park, B. | 2018 | Risky Lifestyle Behaviors among Gastric Cancer Survivors Compared with Matched Non-cancer Controls: Results from Baseline Result of Community Based Cohort Study | Cancer Res Treat | 10.4143/crt.2017.129 |
| **201** | Kim, O., Jang, H. J., Kim, S., Lee, H. Y., Cho, E., Lee, J. E., Jung, H. and Kim, J. | 2018 | Gastroesophageal reflux disease and its related factors among women of reproductive age: Korea Nurses' Health Study | BMC Public Health | 10.1186/s12889-018-6031-3 |
| **202** | Kim, S. A., Choi, B. Y., Song, K. S., Park, C. H., Eun, C. S., Han, D. S., Kim, Y. S. and Kim, H. J. | 2019 | Prediagnostic Smoking and Alcohol Drinking and Gastric Cancer Survival: A Korean Prospective Cohort Study | Korean J Gastroenterol | 10.4166/kjg.2019.73.3.141 |
| **203** | Kim, S. A., Kwak, J. H., Eun, C. S., Han, D. S., Kim, Y. S., Song, K. S., Choi, B. Y. and Kim, H. J. | 2023 | Association of Dietary Antioxidant Vitamin Intake and Gastric Cancer Risk According to Smoking Status and Histological Subtypes of Gastric Cancer: A Case-Control Study in Korea | Nutr Cancer | 10.1080/01635581.2022.2147274 |
| **204** | Kim, S. H., Yun, J. M., Chang, C. B., Piao, H., Yu, S. J. and Shin, D. W. | 2016 | Prevalence of upper gastrointestinal bleeding risk factors among the general population and osteoarthritis patients | World J Gastroenterol | 10.3748/wjg.v22.i48.10643 |
| **205** | Kim, S. W., Lee, J. H., Sim, Y. S., Ryu, Y. J. and Chang, J. H. | 2014 | Prevalence and risk factors for reflux esophagitis in patients with chronic obstructive pulmonary disease | Korean J Intern Med | 10.3904/kjim.2014.29.4.466 |
| **206** | Kim, S. Y., Jung, H. K., Lim, J., Kim, T. O., Choe, A. R., Tae, C. H., Shim, K. N., Moon, C. M., Kim, S. E. and Jung, S. A. | 2019 | Gender Specific Differences in Prevalence and Risk Factors for Gastro-Esophageal Reflux Disease | J Korean Med Sci | 10.3346/jkms.2019.34.e158 |
| **207** | Kirenga, B., Chakaya, J., Yimer, G., Nyale, G., Haile, T., Muttamba, W., Mugenyi, L., Katagira, W., Worodria, W., Aanyu-Tukamuhebwa, H., Lugogo, N., Joloba, M., Bekele, A., Makumbi, F., Green, C., de Jong, C., Kamya, M. and van der Molen, T. | 2020 | Phenotypic characteristics and asthma severity in an East African cohort of adults and adolescents with asthma: findings from the African severe asthma project | BMJ Open Respir Res | 10.1136/bmjresp-2019-000484 |
| **208** | Kissiedu, J., Thota, P. N., Gohel, T., Lopez, R. and Gordon, I. O. | 2016 | Post-ablation lymphocytic esophagitis in Barrett esophagus with high grade dysplasia or intramucosal carcinoma | Mod Pathol | 10.1038/modpathol.2016.50 |
| **209** | Ko, E. H. and Rubin, A. D. | 2014 | Dysphagia due to inclusion body myositis: case presentation and review of the literature | Ann Otol Rhinol Laryngol | 10.1177/0003489414525588 |
| **210** | Ko, K. P., Shin, A., Cho, S., Park, S. K. and Yoo, K. Y. | 2018 | Environmental contributions to gastrointestinal and liver cancer in the Asia-Pacific region | J Gastroenterol Hepatol | 10.1111/jgh.14005 |
| **211** | Koca, T., Arslan, D., Basaran, H., Cerkesli, A. K., Tastekin, D., Sezen, D., Koca, O., Binici, D. N., Bassorgun, C. I. and Ozdogan, M. | 2015 | Dietary and demographical risk factors for oesophageal squamous cell carcinoma in the Eastern Anatolian region of Turkey where upper gastrointestinal cancers are endemic | Asian Pac J Cancer Prev | 10.7314/apjcp.2015.16.5.1913 |
| **212** | Kosaka, M., Yamazaki, Y., Maruno, T., Sakaguchi, K. and Sawaki, S. | 2021 | Corticosteroids as adjunctive therapy in the treatment of coronavirus disease 2019: A report of two cases and literature review | J Infect Chemother | 10.1016/j.jiac.2020.09.007 |
| **213** | Koutlas, N. T., Eluri, S., Rusin, S., Perjar, I., Hollyfield, J., Woosley, J. T., Shaheen, N. J. and Dellon, E. S. | 2018 | Impact of smoking, alcohol consumption, and NSAID use on risk for and phenotypes of eosinophilic esophagitis | Dis Esophagus | 10.1093/dote/dox111 |
| **214** | Krishnamoorthi, R., Singh, S., Ragunathan, K., Visrodia, K., Wang, K. K., Katzka, D. A. and Iyer, P. G. | 2018 | Factors Associated With Progression of Barrett's Esophagus: A Systematic Review and Meta-analysis | Clin Gastroenterol Hepatol | 10.1016/j.cgh.2017.11.044 |
| **215** | Kubo, A., Block, G., Quesenberry, C. P., Jr., Buffler, P. and Corley, D. A. | 2014 | Dietary guideline adherence for gastroesophageal reflux disease | BMC Gastroenterol | 10.1186/1471-230x-14-144 |
| **216** | Kumar, A., Kim, M. and Lukin, D. J. | 2018 | Helicobacter pylori is associated with increased risk of serrated colonic polyps: Analysis of serrated polyp risk factors | Indian J Gastroenterol | 10.1007/s12664-018-0855-8 |
| **217** | Kumar, S., Kumari, N., Mittal, R. D. and Ghoshal, U. C. | 2016 | Pepsinogen-II 100 bp ins/del gene polymorphism and its elevated circulating levels are associated with gastric cancer, particularly with Helicobacter pylori infection and intestinal metaplasia | Gastric Cancer | 10.1007/s10120-015-0550-8 |
| **218** | Kumar, S., Metz, D. C., Ellenberg, S., Kaplan, D. E. and Goldberg, D. S. | 2020 | Risk Factors and Incidence of Gastric Cancer After Detection of Helicobacter pylori Infection: A Large Cohort Study | Gastroenterology | 10.1053/j.gastro.2019.10.019 |
| **219** | Kuo, W. H., Huang, C. Y., Fu, C. K., Hsieh, Y. H., Liao, C. H., Hsu, C. M., Huang, Y. K., Tsai, C. W., Chang, W. S. and Bau, D. T. | 2014 | Effects of interleukin-10 polymorphisms and smoking on the risk of gastric cancer in Taiwan | In Vivo |  |
| **220** | Kurotschka, P. K., Serafini, A. and Ebell, M. H. | 2024 | [Top 4 Research Studies of the month for Italian Primary Care Physicians: December 2023.] | Recenti Prog Med | 10.1701/4169.41641 |
| **221** | La Vecchia, C., Bosetti, C., Bertuccio, P., Castro, C., Pelucchi, C. and Negri, E. | 2014 | Trends in alcohol consumption in Europe and their impact on major alcohol-related cancers | Eur J Cancer Prev | 10.1097/CEJ.0b013e32836562f1 |
| **222** | Lachmann, G., von Haefen, C., Kurth, J., Yuerek, F., Wernecke, K. D. and Spies, C. | 2017 | Smoking, Gender, and Overweight Are Important Influencing Factors on Monocytic HLA-DR before and after Major Cancer Surgery | Biomed Res Int | 10.1155/2017/5216562 |
| **223** | Lai, H. T., Koriyama, C., Tokudome, S., Tran, H. H., Tran, L. T., Nandakumar, A., Akiba, S. and Le, N. T. | 2016 | Waterpipe Tobacco Smoking and Gastric Cancer Risk among Vietnamese Men | PLoS One | 10.1371/journal.pone.0165587 |
| **224** | Lai, K. and Long, L. | 2020 | Current Status and Future Directions of Chronic Cough in China | Lung | 10.1007/s00408-019-00319-z |
| **225** | Lam, A. K. | 2020 | Introduction: Esophageal Squamous Cell Carcinoma-Current Status and Future Advances | Methods Mol Biol | 10.1007/978-1-0716-0377-2_1 |
| **226** | Le, H. X., Truong, D. T. T., Tran, L. B., Le, P. H., Pham, B. U. D., Wada, K., Ikeda, S., Garidkhuu, A., Van Phan, C. and Le, N. T. | 2022 | A prospective cohort study on the association between waterpipe tobacco smoking and gastric cancer mortality in Northern Vietnam | BMC Cancer | 10.1186/s12885-022-09894-6 |
| **227** | Leceaga Gaztambide, E., Aguilar Cayuelas, A. and Fuster Martí, D. | 2018 | Empyema necessitatis 17 years after a pneumonectomy | Med Clin (Barc) | 10.1016/j.medcli.2017.11.006 |
| **228** | Lee, E., Liu, L., Zhang, J., Stern, M. C., Barzi, A., Hwang, A., Kim, A. E., Hamilton, A. S., Wu, A. H. and Deapen, D. | 2017 | Stomach Cancer Disparity among Korean Americans by Tumor Characteristics: Comparison with Non-Hispanic Whites, Japanese Americans, South Koreans, and Japanese | Cancer Epidemiol Biomarkers Prev | 10.1158/1055-9965.Epi-16-0573 |
| **229** | Lee, S. P., Lee, S. Y., Kim, J. H., Sung, I. K., Park, H. S. and Shim, C. S. | 2017 | Factors Related to Upper Gastrointestinal Symptom Generation in 2275 Helicobacter pylori Seroprevalent Adults | Dig Dis Sci | 10.1007/s10620-017-4529-7 |
| **230** | Lee, S. P., Sung, I. K., Kim, J. H., Lee, S. Y., Park, H. S. and Shim, C. S. | 2016 | The Clinical Features and Predisposing Factors of Asymptomatic Erosive Esophagitis | Dig Dis Sci | 10.1007/s10620-016-4341-9 |
| **231** | Lee, Y. B., Yu, J., Choi, H. H., Jeon, B. S., Kim, H. K., Kim, S. W., Kim, S. S., Park, Y. G. and Chae, H. S. | 2017 | The association between peptic ulcer diseases and mental health problems: A population-based study: a STROBE compliant article | Medicine (Baltimore) | 10.1097/md.0000000000007828 |
| **232** | Lee, Y. J., Redd, M., Bayman, L., Frederickson, N., Valestin, J. and Schey, R. | 2015 | Comparison of clinical features in patients with eosinophilic esophagitis living in an urban and rural environment | Dis Esophagus | 10.1111/dote.12164 |
| **233** | Levenstein, S., Jacobsen, R. K., Rosenstock, S. and Jørgensen, T. | 2017 | Mental vulnerability, Helicobacter pylori, and incidence of hospital-diagnosed peptic ulcer over 28 years in a population-based cohort | Scand J Gastroenterol | 10.1080/00365521.2017.1324897 |
| **234** | Li, G., Wulan, H., Song, Z., Paik, P. A., Tsao, M. L., Goodman, G. M., MacEachern, P. T., Downey, R. S., Jankowska, A. J., Rabinowitz, Y. M., Learch, T. B., Song, D. Z., Yuan, J. J., Zheng, S. and Zheng, Z. | 2015 | Regulatory B Cell Function Is Suppressed by Smoking and Obesity in H. pylori-Infected Subjects and Is Correlated with Elevated Risk of Gastric Cancer | PLoS One | 10.1371/journal.pone.0134591 |
| **235** | Li, L. F., Chan, R. L., Lu, L., Shen, J., Zhang, L., Wu, W. K., Wang, L., Hu, T., Li, M. X. and Cho, C. H. | 2014 | Cigarette smoking and gastrointestinal diseases: the causal relationship and underlying molecular mechanisms (review) | Int J Mol Med | 10.3892/ijmm.2014.1786 |
| **236** | Li, N., Wu, P., Shen, Y., Yang, C., Zhang, L., Chen, Y., Wang, Z. and Jiang, J. | 2021 | Predictions of mortality related to four major cancers in China, 2020 to 2030 | Cancer Commun (Lond) | 10.1002/cac2.12143 |
| **237** | Li, P., Guo, X., Jing, J., Hu, W., Wei, W. Q., Qi, X. and Zhuang, G. | 2022 | The lag effect of exposure to PM(2.5) on esophageal cancer in urban-rural areas across China | Environ Sci Pollut Res Int | 10.1007/s11356-021-15942-8 |
| **238** | Li, P., Jing, J., Guo, W., Guo, X., Hu, W., Qi, X., Wei, W. Q. and Zhuang, G. | 2021 | The associations of air pollution and socioeconomic factors with esophageal cancer in China based on a spatiotemporal analysis | Environ Res | 10.1016/j.envres.2020.110415 |
| **239** | Li, P., Jing, J., Liu, W., Wang, J., Qi, X. and Zhang, G. | 2023 | Spatiotemporal Patterns of Esophageal Cancer Burden Attributable to Behavioral, Metabolic, and Dietary Risk Factors From 1990 to 2019: Longitudinal Observational Study | JMIR Public Health Surveill | 10.2196/46051 |
| **240** | Li, Q., Zhu, L., Wei, T., Zang, Z., Zhang, X., Wang, Y., Gao, R., Zhang, Y., Zheng, X. and Liu, F. | 2023 | Secular trends and attributable risk factors of esophageal cancer deaths among non-elderly adults based on Global Burden of Disease Study | J Cancer Res Clin Oncol | 10.1007/s00432-023-05380-z |
| **241** | Li, S., Chen, H., Man, J., Zhang, T., Yin, X., He, Q., Yang, X. and Lu, M. | 2021 | Changing trends in the disease burden of esophageal cancer in China from 1990 to 2017 and its predicted level in 25 years | Cancer Med | 10.1002/cam4.3775 |
| **242** | Li, W. and Wang, W. | 2024 | Causal effects of exposure to ambient air pollution on cancer risk: Insights from genetic evidence | Sci Total Environ | 10.1016/j.scitotenv.2023.168843 |
| **243** | Li, W. Y., Han, Y., Xu, H. M., Wang, Z. N., Xu, Y. Y., Song, Y. X., Xu, H., Yin, S. C., Liu, X. Y. and Miao, Z. F. | 2019 | Smoking status and subsequent gastric cancer risk in men compared with women: a meta-analysis of prospective observational studies | BMC Cancer | 10.1186/s12885-019-5601-9 |
| **244** | Li, X., Yu, C., Guo, Y., Bian, Z., Shen, Z., Yang, L., Chen, Y., Wei, Y., Zhang, H., Qiu, Z., Chen, J., Chen, F., Chen, Z., Lv, J. and Li, L. | 2019 | Association between tea consumption and risk of cancer: a prospective cohort study of 0.5 million Chinese adults | Eur J Epidemiol | 10.1007/s10654-019-00530-5 |
| **245** | Li, Y., He, Z., Wei, J., Xu, R., Liu, T., Zhong, Z., Liu, L., Liang, S., Zheng, Y., Chen, G., Lv, Z., Huang, S., Chen, X., Sun, H. and Liu, Y. | 2024 | Long-term exposure to ambient fine particulate matter constituents and mortality from total and site-specific gastrointestinal cancer | Environ Res | 10.1016/j.envres.2023.117927 |
| **246** | Liang, Z. F., Zhang, Y., Guo, W., Chen, B., Fang, S. and Qian, H. | 2022 | Gastric cancer stem cell-derived exosomes promoted tobacco smoke-triggered development of gastric cancer by inducing the expression of circ670 | Med Oncol | 10.1007/s12032-022-01906-6 |
| **247** | Lie, T. M., Bomme, M., Hveem, K., Hansen, J. M. and Ness-Jensen, E. | 2017 | Snus and risk of gastroesophageal reflux. A population-based case-control study: the HUNT study | Scand J Gastroenterol | 10.1080/00365521.2016.1245775 |
| **248** | Lim, J. H., Lee, D. H., Lee, S. H., Kim, J. S., Jung, H. C. and Cho, S. H. | 2019 | Asthma under control is inversely related with erosive esophagitis among healthy adults | PLoS One | 10.1371/journal.pone.0210490 |
| **249** | Lin, R. A., Calvert, G. M. and Udasin, I. G. | 2023 | World Trade Center Health Program best practices for the diagnosis and treatment of gastroesophageal reflux disease | Arch Environ Occup Health | 10.1080/19338244.2023.2171958 |
| **250** | Lin, S., Wang, X., Huang, C., Liu, X., Zhao, J., Yu, I. T. and Christiani, D. C. | 2015 | Consumption of salted meat and its interactions with alcohol drinking and tobacco smoking on esophageal squamous-cell carcinoma | Int J Cancer | 10.1002/ijc.29406 |
| **251** | Lin, S., Wang, X., Yano, E., Yu, I., Lan, Y., Courtice, M. N. and Christiani, D. C. | 2014 | Exposure to chrysotile mining dust and digestive cancer mortality in a Chinese miner/miller cohort | Occup Environ Med | 10.1136/oemed-2013-101360 |
| **252** | Lin, W. C., Ding, Y. F., Hsu, H. L., Chang, J. H., Yuan, K. S., Wu, A. T. H., Chow, J. M., Chang, C. L., Chen, S. U. and Wu, S. Y. | 2017 | Value and application of trimodality therapy or definitive concurrent chemoradiotherapy in thoracic esophageal squamous cell carcinoma | Cancer | 10.1002/cncr.30823 |
| **253** | Lin, Y., Wu, C., Yan, W., Guo, S., Lin, S. and Liu, B. | 2020 | Sociodemographic and Lifestyle Factors in Relation to Gastric Cancer in a High-Risk Region of China: A Matched Case-Control Study | Nutr Cancer | 10.1080/01635581.2019.1638425 |
| **254** | Lin, Y., Zheng, Y., Wang, H. L. and Wu, J. | 2021 | Global Patterns and Trends in Gastric Cancer Incidence Rates (1988-2012) and Predictions to 2030 | Gastroenterology | 10.1053/j.gastro.2021.03.023 |
| **255** | Lin, Y. C., Shih, H. S. and Lai, C. Y. | 2022 | Long-term nonlinear relationship between PM(2.5) and ten leading causes of death | Environ Geochem Health | 10.1007/s10653-021-01136-1 |
| **256** | Lipka, S., Kumar, A. and Richter, J. E. | 2016 | Impact of Diagnostic Delay and Other Risk Factors on Eosinophilic Esophagitis Phenotype and Esophageal Diameter | J Clin Gastroenterol | 10.1097/mcg.0000000000000297 |
| **257** | Liu, X., Wang, X., Lin, S., Lao, X., Zhao, J., Song, Q., Su, X. and Tak-Sun Yu, I. | 2017 | Dietary patterns and the risk of esophageal squamous cell carcinoma: A population-based case-control study in a rural population | Clin Nutr | 10.1016/j.clnu.2015.11.009 |
| **258** | Liu, X. L., Wang, R. C., Liu, Y. Y., Chen, H., Qi, C., Hu, L. W., Yi, J. and Wang, W. | 2021 | Risk prediction nomogram for major morbidity related to primary resection for esophageal squamous cancer | Medicine (Baltimore) | 10.1097/md.0000000000026189 |
| **259** | Lleo, A. and Colapietro, F. | 2018 | Changes in the Epidemiology of Primary Biliary Cholangitis | Clin Liver Dis | 10.1016/j.cld.2018.03.001 |
| **260** | Löhler, J., Gerstner, A. O., Bootz, F. and Walther, L. E. | 2014 | Incidence and localization of abnormal mucosa findings in patients consulting ENT outpatient clinics and data analysis of a cancer registry | Eur Arch Otorhinolaryngol | 10.1007/s00405-013-2738-z |
| **261** | Long, L. and Lai, K. | 2019 | Characteristics of Chinese chronic cough patients | Pulm Pharmacol Ther | 10.1016/j.pupt.2019.101811 |
| **262** | Lopes, A. B., Metzdorf, M., Metzdorf, L., Sousa, M. P. R., Kavalco, C., Etemadi, A., Pritchett, N. R., Murphy, G., Calafat, A. M., Abnet, C. C., Dawsey, S. M. and Fagundes, R. B. | 2018 | Urinary Concentrations of Polycyclic Aromatic Hydrocarbon Metabolites in Maté Drinkers in Rio Grande do Sul, Brazil | Cancer Epidemiol Biomarkers Prev | 10.1158/1055-9965.Epi-17-0773 |
| **263** | Lu, L., Chen, J., Li, M., Tang, L., Wu, R., Jin, L. and Liang, Z. | 2018 | β‑carotene reverses tobacco smoke‑induced gastric EMT via Notch pathway in vivo | Oncol Rep | 10.3892/or.2018.6246 |
| **264** | Lund, I. and Scheffels, J. | 2014 | Perceptions of relative risk of disease and addiction from cigarettes and snus | Psychol Addict Behav | 10.1037/a0032657 |
| **265** | Luo, M. X., Long, B. B., Li, F., Zhang, C., Pan, M. T., Huang, Y. Q. and Chen, B. | 2019 | Roles of Cyclooxygenase-2 gene -765G > C (rs20417) and -1195G > A (rs689466) polymorphisms in gastric cancer: A systematic review and meta-analysis | Gene | 10.1016/j.gene.2018.10.077 |
| **266** | Luquiens, A., Lourenco, N., Benyamina, A. and Aubin, H. J. | 2015 | Self-medication of achalasia with cannabis, complicated by a cannabis use disorder | World J Gastroenterol | 10.3748/wjg.v21.i20.6381 |
| **267** | Lv, Z., Sun, L., Xu, Q., Gong, Y., Jing, J., Dong, N., Xing, C. and Yuan, Y. | 2018 | SNP interactions of PGC with its neighbor lncRNAs enhance the susceptibility to gastric cancer/atrophic gastritis and influence the expression of involved molecules | Cancer Med | 10.1002/cam4.1743 |
| **268** | Lyons, K., Le, L. C., Pham, Y. T., Borron, C., Park, J. Y., Tran, C. T. D., Tran, T. V., Tran, H. T., Vu, K. T., Do, C. D., Pelucchi, C., La Vecchia, C., Zgibor, J., Boffetta, P. and Luu, H. N. | 2019 | Gastric cancer: epidemiology, biology, and prevention: a mini review | Eur J Cancer Prev | 10.1097/cej.0000000000000480 |
| **269** | Ma, G., Zhang, X., Ma, Q., Rong, T., Long, H., Lin, P., Fu, J. and Zhang, L. | 2015 | A novel multivariate scoring system for determining the prognosis of lymph node-negative esophageal squamous cell carcinoma following surgical therapy: an observational study | Eur J Surg Oncol | 10.1016/j.ejso.2015.01.013 |
| **270** | Mahale, P., Sturgis, E. M., Tweardy, D. J., Ariza-Heredia, E. J. and Torres, H. A. | 2016 | Association Between Hepatitis C Virus and Head and Neck Cancers | J Natl Cancer Inst | 10.1093/jnci/djw035 |
| **271** | Malik, Z., Bayman, L., Valestin, J., Rizvi-Toner, A., Hashmi, S. and Schey, R. | 2017 | Dronabinol increases pain threshold in patients with functional chest pain: a pilot double-blind placebo-controlled trial | Dis Esophagus | 10.1111/dote.12455 |
| **272** | Mantziari, S., Allemann, P., Winiker, M., Demartines, N. and Schäfer, M. | 2018 | Locoregional Tumor Extension and Preoperative Smoking are Significant Risk Factors for Early Recurrence After Esophagectomy for Cancer | World J Surg | 10.1007/s00268-017-4422-8 |
| **273** | Mantziari, S., Hübner, M., Demartines, N. and Schäfer, M. | 2014 | Impact of preoperative risk factors on morbidity after esophagectomy: is there room for improvement? | World J Surg | 10.1007/s00268-014-2686-9 |
| **274** | Marqués-Lespier, J. M., González-Pons, M. and Cruz-Correa, M. | 2016 | Current Perspectives on Gastric Cancer | Gastroenterol Clin North Am | 10.1016/j.gtc.2016.04.002 |
| **275** | Martinucci, I., Natilli, M., Lorenzoni, V., Pappalardo, L., Monreale, A., Turchetti, G., Pedreschi, D., Marchi, S., Barale, R. and de Bortoli, N. | 2018 | Gastroesophageal reflux symptoms among Italian university students: epidemiology and dietary correlates using automatically recorded transactions | BMC Gastroenterol | 10.1186/s12876-018-0832-9 |
| **276** | Matejcic, M., Gunter, M. J. and Ferrari, P. | 2017 | Alcohol metabolism and oesophageal cancer: a systematic review of the evidence | Carcinogenesis | 10.1093/carcin/bgx067 |
| **277** | Matejcic, M., Vogelsang, M., Wang, Y. and Iqbal Parker, M. | 2015 | NAT1 and NAT2 genetic polymorphisms and environmental exposure as risk factors for oesophageal squamous cell carcinoma: a case-control study | BMC Cancer | 10.1186/s12885-015-1105-4 |
| **278** | Matsueda, K., Manabe, N., Toshikuni, N., Sato, Y., Watanabe, T., Yamamoto, H. and Haruma, K. | 2017 | Clinical characteristics and associated factors of Japanese patients with adenocarcinoma of the esophagogastric junction: a multicenter clinicoepidemiological study | Dis Esophagus | 10.1093/dote/dox007 |
| **279** | Matsuzaki, J., Suzuki, H., Kobayakawa, M., Inadomi, J. M., Takayama, M., Makino, K., Iwao, Y., Sugino, Y. and Kanai, T. | 2015 | Association of Visceral Fat Area, Smoking, and Alcohol Consumption with Reflux Esophagitis and Barrett's Esophagus in Japan | PLoS One | 10.1371/journal.pone.0133865 |
| **280** | May Maestas, M., Perry, K. D., Smith, K., Firszt, R., Allen-Brady, K., Robson, J., Joy, E. and Peterson, K. | 2019 | Food impactions in Eosinophilic esophagitis and acute exposures to fine particulate pollution | Allergy | 10.1111/all.13932 |
| **281** | McCain, R. S., McManus, D. T., McQuaid, S., James, J. A., Salto-Tellez, M., Reid, N. B., Craig, S., Chisambo, C., Bingham, V., McCarron, E., Parkes, E., Turkington, R. C. and Coleman, H. G. | 2020 | Alcohol intake, tobacco smoking, and esophageal adenocarcinoma survival: a molecular pathology epidemiology cohort study | Cancer Causes Control | 10.1007/s10552-019-01247-2 |
| **282** | McCallum, R. W. and Bashashati, M. | 2019 | Cannabis for Gastroparesis: Hype or Hope? | Am J Gastroenterol | 10.14309/ajg.0000000000000277 |
| **283** | McCarty, T. R., Chouairi, F., Hathorn, K. E., Chan, W. W. and Thompson, C. C. | 2022 | Trends and Socioeconomic Health Outcomes of Cannabis Use Among Patients With Gastroparesis: A United States Nationwide Inpatient Sample Analysis | J Clin Gastroenterol | 10.1097/mcg.0000000000001526 |
| **284** | Mehrtash, H., Duncan, K., Parascandola, M., David, A., Gritz, E. R., Gupta, P. C., Mehrotra, R., Amer Nordin, A. S., Pearlman, P. C., Warnakulasuriya, S., Wen, C. P., Zain, R. B. and Trimble, E. L. | 2017 | Defining a global research and policy agenda for betel quid and areca nut | Lancet Oncol | 10.1016/s1470-2045(17)30460-6 |
| **285** | Miftahussurur, M., Doohan, D., Nusi, I. A., Adi, P., Rezkitha, Y. A. A., Waskito, L. A., Fauzia, K. A., Bramantoro, T., Maimunah, U., Thamrin, H., Masithah, S. I., Sukadiono, S., Uchida, T., Lusida, M. I. and Yamaoka, Y. | 2018 | Gastroesophageal reflux disease in an area with low Helicobacter pylori infection prevalence | PLoS One | 10.1371/journal.pone.0205644 |
| **286** | Milliet, F., Bozec, A., Schiappa, R., Viotti, J., Modesto, A., Dassonville, O., Poissonnet, G., Guelfucci, B., Bizeau, A., Vergez, S., Dupret-Bories, A., Garrel, R., Fakhry, N., Santini, L., Lallemant, B., Chambon, G., Sudaka, A., Peyrade, F., Saada-Bouzid, E., Benezery, K., Jourdan-Soulier, F., Chapel, F., Sophie Ramay, A., Roger, P., Galissier, T., Coste, V., Ben Lakdar, A., Guerlain, J., Temam, S., Mirghani, H., Gorphe, P., Chamorey, E. and Culié, D. | 2021 | Synchronous primary neoplasia in patients with oropharyngeal cancer: Impact of tumor HPV status. A GETTEC multicentric study | Oral Oncol | 10.1016/j.oraloncology.2020.105041 |
| **287** | Mills, R. and Hathorn, I. | 2016 | Aetiology and pathology of otitis media with effusion in adult life | J Laryngol Otol | 10.1017/s0022215116000943 |
| **288** | Minakari, M., Badihian, S., Jalalpour, P. and Sebghatollahi, V. | 2017 | Etiology and outcome in patients with upper gastrointestinal bleeding: Study on 4747 patients in the central region of Iran | J Gastroenterol Hepatol | 10.1111/jgh.13617 |
| **289** | Minami, Y., Kanemura, S., Oikawa, T., Suzuki, S., Hasegawa, Y., Miura, K., Nishino, Y., Kakugawa, Y. and Fujiya, T. | 2018 | Associations of cigarette smoking and alcohol drinking with stomach cancer survival: A prospective patient cohort study in Japan | Int J Cancer | 10.1002/ijc.31408 |
| **290** | Minowa, H. | 2016 | Respiratory inhibition after crying or gastroesophageal reflux and feeding hypoxemia in infants | J Matern Fetal Neonatal Med | 10.3109/14767058.2015.1085011 |
| **291** | Miranti, E. H., Freedman, N. D., Weinstein, S. J., Abnet, C. C., Selhub, J., Murphy, G., Diaw, L., Männistö, S., Taylor, P. R., Albanes, D. and Stolzenberg-Solomon, R. Z. | 2016 | Prospective study of serum cysteine and cysteinylglycine and cancer of the head and neck, esophagus, and stomach in a cohort of male smokers | Am J Clin Nutr | 10.3945/ajcn.115.125799 |
| **292** | Mlombe, Y. B., Rosenberg, N. E., Wolf, L. L., Dzamalala, C. P., Chalulu, K., Chisi, J., Shaheen, N. J., Hosseinipour, M. C. and Shores, C. G. | 2015 | Environmental risk factors for oesophageal cancer in Malawi: A case-control study | Malawi Med J | 10.4314/mmj.v27i3.3 |
| **293** | Moberg, L., Nilsson, P. M., Samsioe, G., Sallsten, G., Barregard, L., Engström, G. and Borgfeldt, C. | 2017 | Increased blood cadmium levels were not associated with increased fracture risk but with increased total mortality in women: the Malmö Diet and Cancer Study | Osteoporos Int | 10.1007/s00198-017-4047-7 |
| **294** | Mochizuki, N., Fujita, T., Kobayashi, M., Yamazaki, Y., Terao, S., Sanuki, T., Okada, A., Adachi, M., Murakami, M., Arisaka, Y., Uno, K., Masuda, A., Yoshida, M., Umegaki, E., Kutsumi, H. and Azuma, T. | 2018 | Factors associated with the presentation of erosive esophagitis symptoms in health checkup subjects: A prospective, multicenter cohort study | PLoS One | 10.1371/journal.pone.0196848 |
| **295** | Moeller, M., Pink, C., Endlich, N., Endlich, K., Grabe, H. J., Völzke, H., Dörr, M., Nauck, M., Lerch, M. M., Köhling, R., Holtfreter, B., Kocher, T. and Fuellen, G. | 2017 | Mortality is associated with inflammation, anemia, specific diseases and treatments, and molecular markers | PLoS One | 10.1371/journal.pone.0175909 |
| **296** | Mohammadi, N., Alimohammadian, M., Feizesani, A., Poustchi, H., Alizadeh, A., Yaseri, M., Mansournia, M. A. and Sadjadi, A. | 2021 | The marginal causal effect of opium consumption on the upper gastrointestinal cancer death using parametric g-formula: An analysis of 49,946 cases in the Golestan Cohort Study, Iran | PLoS One | 10.1371/journal.pone.0246004 |
| **297** | Mohy-Ud-Din, N., Krill, T. S., Shah, A. R., Chatila, A. T., Singh, S., Bilal, M. and Parupudi, S. | 2020 | Barrett's esophagus: What do we need to know? | Dis Mon | 10.1016/j.disamonth.2019.02.003 |
| **298** | Montazeri, Z., Nyiraneza, C., El-Katerji, H. and Little, J. | 2017 | Waterpipe smoking and cancer: systematic review and meta-analysis | Tob Control | 10.1136/tobaccocontrol-2015-052758 |
| **299** | Montiel-Jarquín Á, J., Lara-Cisneros, L. G. V., López-Colombo, A., Solís-Mendoza, H. A., Palmer-Márquez, M. L. and Romero-Figueroa, M. S. | 2019 | Expression of metalloproteinase-9 in patients with mild and severe forms of gastroesophageal reflux disease | Cir Cir | 10.24875/ciru.18000691 |
| **300** | Morais, S., Antunes, L., Bento, M. J. and Lunet, N. | 2019 | Risk and survival of third primary cancers in a population-based cohort of gastric cancer patients | Dig Liver Dis | 10.1016/j.dld.2018.12.003 |
| **301** | Morais, S., Rodrigues, S., Amorim, L., Peleteiro, B. and Lunet, N. | 2014 | Tobacco smoking and intestinal metaplasia: Systematic review and meta-analysis | Dig Liver Dis | 10.1016/j.dld.2014.08.034 |
| **302** | Morimoto, C., Matsumoto, H., Nagasaki, T., Kanemitsu, Y., Ishiyama, Y., Sunadome, H., Oguma, T., Ito, I., Murase, K., Kawaguchi, T., Tabara, Y., Niimi, A., Muro, S., Matsuda, F., Chin, K. and Hirai, T. | 2021 | Gastroesophageal reflux disease is a risk factor for sputum production in the general population: the Nagahama study | Respir Res | 10.1186/s12931-020-01601-y |
| **303** | Moses, A., Mwafongo, A., Chikasema, M., Kafantenganji, L., Stanely, C., Chimzukira, E., Kampani, C., Krysiak, R., Gopal, S., Rosenberg, N. E., Shores, C. G. and Hosseinipour, M. C. | 2017 | Risk factors for common cancers among patients at Kamuzu Central Hospital in Lilongwe, Malawi: A retrospective cohort study | Malawi Med J | 10.4314/mmj.v29i2.11 |
| **304** | Mozzanica, F., Ginocchio, D., Barillari, R., Barozzi, S., Maruzzi, P., Ottaviani, F. and Schindler, A. | 2016 | Prevalence and Voice Characteristics of Laryngeal Pathology in an Italian Voice Therapy-seeking Population | J Voice | 10.1016/j.jvoice.2015.11.018 |
| **305** | Muhsen, K., Sinnreich, R., Merom, D., Beer-Davidson, G., Nassar, H., Cohen, D. and Kark, J. D. | 2019 | Prevalence and determinants of serological evidence of atrophic gastritis among Arab and Jewish residents of Jerusalem: a cross-sectional study | BMJ Open | 10.1136/bmjopen-2018-024689 |
| **306** | Mukherjee, D., Devi, K. R., Deka, M., Malakar, M., Kaur, T., Barua, D., Mahanta, J. and Narain, K. | 2016 | Association of toll-like receptor 2 ∆22 and risk for gastric cancer considering main effects and interactions with smoking: a matched case-control study from Mizoram, India | Tumour Biol | 10.1007/s13277-016-4982-5 |
| **307** | Naclerio, R. M. and Baroody, F. M. | 2016 | Other Phenotypes and Treatment of Chronic Rhinosinusitis | J Allergy Clin Immunol Pract | 10.1016/j.jaip.2016.03.016 |
| **308** | Najafi, F. | 2019 | Tobacco Smoking and Alcohol Drinking: Two Clinically Significant Risk Factors for Esophageal Squamous Cell Carcinoma | Gastroenterology | 10.1053/j.gastro.2019.04.054 |
| **309** | Nana Sede Mbakop, R., Kesiena, O., Greene, T. E. and Amakye, D. | 2023 | Cannabinoid Hyperemesis Syndrome in a 23-Year-Old Woman with Uncontrolled Type 1 Diabetes Mellitus | Am J Case Rep | 10.12659/ajcr.938418 |
| **310** | Navab, F., Nathanson, B. H. and Desilets, D. J. | 2015 | The impact of lifestyle on Barrett's Esophagus: A precursor to esophageal adenocarcinoma | Cancer Epidemiol | 10.1016/j.canep.2015.10.013 |
| **311** | Negovan, A., Iancu, M., Moldovan, V., Mocan, S. and Banescu, C. | 2017 | The Interaction between GSTT1, GSTM1, and GSTP1 Ile105Val Gene Polymorphisms and Environmental Risk Factors in Premalignant Gastric Lesions Risk | Biomed Res Int | 10.1155/2017/7365080 |
| **312** | Negovan, A., Iancu, M., Tripon, F., Crauciuc, A., Mocan, S. and Bănescu, C. | 2018 | The CAT-262 C>T, MnSOD Ala16Val, GPX1 Pro198Leu Polymorphisms Related to Oxidative Stress and the Presence of Gastric Lesions | J Gastrointestin Liver Dis | 10.15403/jgld.2014.1121.274.cat |
| **313** | Ness-Jensen, E., Hveem, K., El-Serag, H. and Lagergren, J. | 2016 | Lifestyle Intervention in Gastroesophageal Reflux Disease | Clin Gastroenterol Hepatol | 10.1016/j.cgh.2015.04.176 |
| **314** | Ness-Jensen, E. and Lagergren, J. | 2017 | Tobacco smoking, alcohol consumption and gastro-oesophageal reflux disease | Best Pract Res Clin Gastroenterol | 10.1016/j.bpg.2017.09.004 |
| **315** | Nguyen, C. L., Sengngam, K., Hoc, T. H., Le, P. H., Hang, L. T. M., Dao, H. V. and Tran Ngoan, L. | 2022 | Waterpipe Tobacco Smoking and Risk of Stomach Cancer: A Case-Control Study in Vietnamese Men | Asian Pac J Cancer Prev | 10.31557/apjcp.2022.23.5.1587 |
| **316** | Nguyen, T., Tang, Z., Younes, M., Alsarraj, A., Ramsey, D., Fitzgerald, S., Kramer, J. R. and El-Serag, H. B. | 2015 | Esophageal COX-2 expression is increased in Barrett's esophagus, obesity, and smoking | Dig Dis Sci | 10.1007/s10620-014-3333-x |
| **317** | Nguyen, T. H., Thrift, A. P., Ramsey, D., Green, L., Shaib, Y. H., Graham, D. Y. and El-Serag, H. B. | 2014 | Risk factors for Barrett's esophagus compared between African Americans and non-Hispanic Whites | Am J Gastroenterol | 10.1038/ajg.2014.351 |
| **318** | Nicolini, A., Barbagelata, E., Tagliabue, E., Colombo, D., Monacelli, F. and Braido, F. | 2018 | Gender differences in chronic obstructive pulmonary diseases: a narrative review | Panminerva Med | 10.23736/s0031-0808.18.03463-8 |
| **319** | Nobel, T. B., Livschitz, J., Xing, X. X., Barbetta, A., Hsu, M., Tan, K. S., Sihag, S., Jones, D. R. and Molena, D. | 2019 | Surveillance Implications of Recurrence Patterns in Early Node-Negative Esophageal Adenocarcinoma | Ann Thorac Surg | 10.1016/j.athoracsur.2019.05.066 |
| **320** | Nolen, L. D., Bruden, D., Miernyk, K., McMahon, B. J., Sacco, F., Varner, W., Mezzetti, T., Hurlburt, D., Tiesinga, J. and Bruce, M. G. | 2018 | H. pylori-associated pathologic findings among Alaska native patients | Int J Circumpolar Health | 10.1080/22423982.2018.1510715 |
| **321** | Norii, T. and Plate, A. | 2017 | Electronic Cigarette Explosion Resulting in a C1 and C2 Fracture: A Case Report | J Emerg Med | 10.1016/j.jemermed.2016.08.010 |
| **322** | Oh, T. K., Jeon, J. H., Lee, J. M., Kim, M. S., Kim, J. H., Lee, S. J. and Eom, W. | 2018 | Chronic Smoking is Not Associated with Increased Postoperative Opioid Use in Patients with Lung Cancer or Esophageal Cancer | Pain Physician |  |
| **323** | Ohashi, S., Miyamoto, S., Kikuchi, O., Goto, T., Amanuma, Y. and Muto, M. | 2015 | Recent Advances From Basic and Clinical Studies of Esophageal Squamous Cell Carcinoma | Gastroenterology | 10.1053/j.gastro.2015.08.054 |
| **324** | Okada, E., Nakamura, K., Ukawa, S., Sakata, K., Date, C., Iso, H. and Tamakoshi, A. | 2016 | Dietary Patterns and Risk of Esophageal Cancer Mortality: The Japan Collaborative Cohort Study | Nutr Cancer | 10.1080/01635581.2016.1192202 |
| **325** | Okamoto, T. and Ito, A. | 2023 | The Association between Smoking Exposure and Reflux Esophagitis: A Cross-sectional Study among Men Conducted as a Part of Health Screening | Intern Med | 10.2169/internalmedicine.0451-22 |
| **326** | Okello, S., Churchill, C., Owori, R., Nasasira, B., Tumuhimbise, C., Abonga, C. L., Mutiibwa, D., Christiani, D. C. and Corey, K. E. | 2016 | Population attributable fraction of Esophageal squamous cell carcinoma due to smoking and alcohol in Uganda | BMC Cancer | 10.1186/s12885-016-2492-x |
| **327** | Olsson, E. C., Jobson, M. and Lim, M. R. | 2015 | Risk factors for persistent dysphagia after anterior cervical spine surgery | Orthopedics | 10.3928/01477447-20150402-61 |
| **328** | Ong, J. S., Gharahkhani, P., Vaughan, T. L., Whiteman, D., Kendall, B. J. and MacGregor, S. | 2022 | Assessing the genetic relationship between gastro-esophageal reflux disease and risk of COVID-19 infection | Hum Mol Genet | 10.1093/hmg/ddab253 |
| **329** | Ovnat Tamir, S., Gershnabel Milk, D., Roth, Y., Cinamon, U., Winder, A., Brenner, R., Katz, A. and Marom, T. | 2016 | Laryngeal Side Effects of Tyrosine Kinase Inhibitors | J Voice | 10.1016/j.jvoice.2015.07.006 |
| **330** | Özden Mat, D., Firat, S., Aksu, K., Aksu, F. and Duyar, SŞ | 2021 | Obstructive sleep apnea is a determinant of asthma control independent of smoking, reflux, and rhinitis | Allergy Asthma Proc | 10.2500/aap.2021.42.200098 |
| **331** | Oze, I., Charvat, H., Matsuo, K., Ito, H., Tamakoshi, A., Nagata, C., Wada, K., Sugawara, Y., Sawada, N., Yamaji, T., Naito, M., Tanaka, K., Shimazu, T., Mizoue, T., Tsugane, S. and Inoue, M. | 2019 | Revisit of an unanswered question by pooled analysis of eight cohort studies in Japan: Does cigarette smoking and alcohol drinking have interaction for the risk of esophageal cancer? | Cancer Med | 10.1002/cam4.2514 |
| **332** | Pan, D., Su, M., Zhang, T., Miao, C., Fu, L., Yang, L., Song, G., Raine, P. J., Wang, S. and Sun, G. | 2019 | A Distinct Epidemiologic Pattern of Precancerous Lesions of Esophageal Squamous Cell Carcinoma in a High-risk Area of Huai'an, Jiangsu Province, China | Cancer Prev Res (Phila) | 10.1158/1940-6207.Capr-18-0462 |
| **333** | Pan, W., Du, J., Shi, M., Jin, G. and Yang, M. | 2017 | Short leukocyte telomere length, alone and in combination with smoking, contributes to increased risk of gastric cancer or esophageal squamous cell carcinoma | Carcinogenesis | 10.1093/carcin/bgw111 |
| **334** | Pan, W., Yang, J., Wei, J., Chen, H., Ge, Y., Zhang, J., Wang, Z., Zhou, C., Yuan, Q., Zhou, L. and Yang, M. | 2015 | Functional BCL-2 regulatory genetic variants contribute to susceptibility of esophageal squamous cell carcinoma | Sci Rep | 10.1038/srep11833 |
| **335** | Pandey, A., Tripathi, S. C., Mahata, S., Vishnoi, K., Shukla, S., Misra, S. P., Misra, V., Hedau, S., Mehrotra, R., Dwivedi, M. and Bharti, A. C. | 2014 | Carcinogenic Helicobacter pylori in gastric pre-cancer and cancer lesions: association with tobacco-chewing | World J Gastroenterol | 10.3748/wjg.v20.i22.6860 |
| **336** | Pandey, A., Tripathi, S. C., Shukla, S., Mahata, S., Vishnoi, K., Misra, S. P., Misra, V., Mitra, S., Dwivedi, M. and Bharti, A. C. | 2018 | Differentially localized survivin and STAT3 as markers of gastric cancer progression: Association with Helicobacter pylori | Cancer Rep (Hoboken) | 10.1002/cnr2.1004 |
| **337** | Parasa, S., Vennalaganti, S., Gaddam, S., Vennalaganti, P., Young, P., Gupta, N., Thota, P., Cash, B., Mathur, S., Sampliner, R., Moawad, F., Lieberman, D., Bansal, A., Kennedy, K. F., Vargo, J., Falk, G., Spaander, M., Bruno, M. and Sharma, P. | 2018 | Development and Validation of a Model to Determine Risk of Progression of Barrett's Esophagus to Neoplasia | Gastroenterology | 10.1053/j.gastro.2017.12.009 |
| **338** | Park, S. K., Kim, M. H., Jung, J. Y., Oh, C. M., Ha, E., Yang, E. H., Lee, H. C., Hwang, W. Y., You, A. H. and Ryoo, J. H. | 2022 | Change in smoking status and its relation to the risk of gastroduodenal ulcer in Korean men | J Gastroenterol Hepatol | 10.1111/jgh.15979 |
| **339** | Park, S. K., Kim, M. H., Oh, C. M., Ha, E., Yang, E. H., Hwang, W. Y., You, A. H. and Ryoo, J. H. | 2022 | The risk of gastric cancer according to changes in smoking status among Korean men | Epidemiol Health | 10.4178/epih.e2022086 |
| **340** | Parkman, H. P., Sharkey, E. P., Nguyen, L. A., Yates, K. P., Abell, T. L., Hasler, W. L., Snape, W., Clarke, J., Schey, R., Koch, K. L., Kuo, B., McCallum, R. W., Sarosiek, I., Grover, M., Farrugia, G., Tonascia, J. and Pasricha, P. J. | 2020 | Marijuana Use in Patients with Symptoms of Gastroparesis: Prevalence, Patient Characteristics, and Perceived Benefit | Dig Dis Sci | 10.1007/s10620-019-05963-2 |
| **341** | Parmar, G. S., Das, S. and Ingledew, P. A. | 2023 | Quality of Online Information for Esophageal Cancer | J Cancer Educ | 10.1007/s13187-022-02198-0 |
| **342** | Parsel, S. M., Iarocci, A. L., Gastañaduy, M., Winters, R. D., Marino, J. P. and McCoul, E. D. | 2020 | Reflux Disease and Laryngeal Neoplasia in Nonsmokers and Nondrinkers | Otolaryngol Head Neck Surg | 10.1177/0194599820917669 |
| **343** | Parsel, S. M., Wu, E. L., Riley, C. A. and McCoul, E. D. | 2019 | Gastroesophageal and Laryngopharyngeal Reflux Associated With Laryngeal Malignancy: A Systematic Review and Meta-analysis | Clin Gastroenterol Hepatol | 10.1016/j.cgh.2018.10.028 |
| **344** | Pasricha, S., Gupta, A., Reed, C. C., Speck, O., Woosley, J. T. and Dellon, E. S. | 2016 | Lymphocytic Esophagitis: An Emerging Clinicopathologic Disease Associated with Dysphagia | Dig Dis Sci | 10.1007/s10620-016-4230-2 |
| **345** | Pasricha, T. S. and Kochar, B. | 2021 | Vaping-associated esophagitis | BMC Gastroenterol | 10.1186/s12876-021-01695-8 |
| **346** | Patterson, K. A., Roberts-Thomson, P. J., Lester, S., Tan, J. A., Hakendorf, P., Rischmueller, M., Zochling, J., Sahhar, J., Nash, P., Roddy, J., Hill, C., Nikpour, M., Stevens, W., Proudman, S. M. and Walker, J. G. | 2015 | Interpretation of an Extended Autoantibody Profile in a Well-Characterized Australian Systemic Sclerosis (Scleroderma) Cohort Using Principal Components Analysis | Arthritis Rheumatol | 10.1002/art.39316 |
| **347** | Paul, G., Bohle, W. and Zoller, W. | 2019 | Risk Factors for the Development of Esophagorespiratory Fistula in Esophageal Cancer | J Gastrointestin Liver Dis | 10.15403/jgld-271 |
| **348** | Pavlidou, E., Papadopoulou, S. K., Tolia, M., Mentzelou, M., Tsoukalas, N., Alexatou, O., Tsiouda, T., Tsourouflis, G., Psara, E., Bikos, V., Kavantzas, N., Kotta-Loizou, I., Dakanalis, A., Vorvolakos, T. and Giaginis, C. | 2023 | Association of Mediterranean Diet Adherence with Disease Progression Characteristics, Lifestyle Factors and Overall Survival in Gastric Cancer Patients | Med Sci (Basel) | 10.3390/medsci11040074 |
| **349** | Pedersen, S. B., Nielsen, J. C., Bøtker, H. E., Farkas, D. K., Schmidt, M. and Sørensen, H. T. | 2015 | Implantable cardioverter-defibrillators and subsequent cancer risk: a nationwide population-based cohort study | Europace | 10.1093/europace/euv076 |
| **350** | Peleteiro, B., Castro, C., Morais, S., Ferro, A. and Lunet, N. | 2015 | Worldwide Burden of Gastric Cancer Attributable to Tobacco Smoking in 2012 and Predictions for 2020 | Dig Dis Sci | 10.1007/s10620-015-3624-x |
| **351** | Peng, L. C., Hui, X., Cheng, Z., Bowers, M. R., Moore, J., Cecil, E., Choflet, A., Thompson, A., Muse, M., Kiess, A. P., Page, B. R., Gourin, C. G., Fakhry, C., Szczesniak, M., Maclean, J., Wu, P., Cook, I., McNutt, T. R. and Quon, H. | 2018 | Prospective evaluation of patient reported swallow function with the Functional Assessment of Cancer Therapy (FACT), MD Anderson Dysphagia Inventory (MDADI) and the Sydney Swallow Questionnaire (SSQ) in head and neck cancer patients | Oral Oncol | 10.1016/j.oraloncology.2018.05.012 |
| **352** | Pesek, R. D., Rettiganti, M., O'Brien, E., Beckwith, S., Daniel, C., Luo, C., Scurlock, A. M., Chandler, P., Levy, R. A., Perry, T. T., Kennedy, J. L., Chervinskiy, S., Vonlanthen, M., Casteel, H., Fiedorek, S. C., Gibbons, T. and Jones, S. M. | 2017 | Effects of allergen sensitization on response to therapy in children with eosinophilic esophagitis | Ann Allergy Asthma Immunol | 10.1016/j.anai.2017.06.006 |
| **353** | Phuoc, L. H., Sengngam, K., Ogawa, T., Ngatu, N. R., Ikeda, S., Hoc, T. H., Phu, P. V., Minh, D. T. and Ngoan, L. T. | 2020 | Fruit and Vegetable Intake and Stomach Cancer among Male Adults: A Case-Control Study in Northern Viet Nam | Asian Pac J Cancer Prev | 10.31557/apjcp.2020.21.7.2109 |
| **354** | Pilakasiri, A. and Mahakit, P. | 2018 | Prospective study of the prevalence and co-morbidities of obstructive sleep apnea in active-duty army personnel in the three southernmost provinces of Thailand using questionnaire screening | Mil Med Res | 10.1186/s40779-018-0186-1 |
| **355** | Piloiu, C. and Dumitrascu, D. L. | 2020 | Barrett's Esophagus in Romania: what do we know? | Rom J Intern Med | 10.2478/rjim-2020-0007 |
| **356** | Plumejeaud, S., Reis, A. P., Tassistro, V., Patinha, C., Noack, Y. and Orsière, T. | 2018 | Potentially harmful elements in house dust from Estarreja, Portugal: characterization and genotoxicity of the bioaccessible fraction | Environ Geochem Health | 10.1007/s10653-016-9888-z |
| **357** | Poosari, A., Nutravong, T., Sa-Ngiamwibool, P., Namwat, W., Chatrchaiwiwatana, S. and Ungareewittaya, P. | 2021 | Association between infection with Campylobacter species, poor oral health and environmental risk factors on esophageal cancer: a hospital-based case-control study in Thailand | Eur J Med Res | 10.1186/s40001-021-00561-3 |
| **358** | Popa, S. L., Chiarioni, G., David, L., Golea, G. I. and Dumitrascu, D. L. | 2019 | Functional Emesis | J Gastrointestin Liver Dis | 10.15403/jgld-236 |
| **359** | Prabhu, A., Obi, K., Lieberman, D. and Rubenstein, J. H. | 2016 | The Race-Specific Incidence of Esophageal Squamous Cell Carcinoma in Individuals With Exposure to Tobacco and Alcohol | Am J Gastroenterol | 10.1038/ajg.2016.346 |
| **360** | Prabhu, A., Obi, K. O. and Rubenstein, J. H. | 2014 | The synergistic effects of alcohol and tobacco consumption on the risk of esophageal squamous cell carcinoma: a meta-analysis | Am J Gastroenterol | 10.1038/ajg.2014.71 |
| **361** | Praud, D., Bertuccio, P., Bosetti, C., Turati, F., Ferraroni, M. and La Vecchia, C. | 2014 | Adherence to the Mediterranean diet and gastric cancer risk in Italy | Int J Cancer | 10.1002/ijc.28620 |
| **362** | Praud, D., Rota, M., Pelucchi, C., Bertuccio, P., Rosso, T., Galeone, C., Zhang, Z. F., Matsuo, K., Ito, H., Hu, J., Johnson, K. C., Yu, G. P., Palli, D., Ferraroni, M., Muscat, J., Lunet, N., Peleteiro, B., Malekzadeh, R., Ye, W., Song, H., Zaridze, D., Maximovitch, D., Aragonés, N., Castaño-Vinyals, G., Vioque, J., Navarrete-Muñoz, E. M., Pakseresht, M., Pourfarzi, F., Wolk, A., Orsini, N., Bellavia, A., Håkansson, N., Mu, L., Pastorino, R., Kurtz, R. C., Derakhshan, M. H., Lagiou, A., Lagiou, P., Boffetta, P., Boccia, S., Negri, E. and La Vecchia, C. | 2018 | Cigarette smoking and gastric cancer in the Stomach Cancer Pooling (StoP) Project | Eur J Cancer Prev | 10.1097/cej.0000000000000290 |
| **363** | Qin, Y., Tong, X., Fan, J., Liu, Z., Zhao, R., Zhang, T., Suo, C., Chen, X. and Zhao, G. | 2021 | Global Burden and Trends in Incidence, Mortality, and Disability of Stomach Cancer From 1990 to 2017 | Clin Transl Gastroenterol | 10.14309/ctg.0000000000000406 |
| **364** | Quan, H., Ouyang, L., Zhou, H., Ouyang, Y. and Xiao, H. | 2019 | The effect of preoperative smoking cessation and smoking dose on postoperative complications following radical gastrectomy for gastric cancer: a retrospective study of 2469 patients | World J Surg Oncol | 10.1186/s12957-019-1607-7 |
| **365** | Quan, S., Yang, H., Tanyingoh, D., Villeneuve, P. J., Stieb, D. M., Johnson, M., Hilsden, R., Madsen, K., van Zanten, S. V., Novak, K., Lang, E., Ghosh, S. and Kaplan, G. G. | 2015 | Upper gastrointestinal bleeding due to peptic ulcer disease is not associated with air pollution: a case-crossover study | BMC Gastroenterol | 10.1186/s12876-015-0363-6 |
| **366** | Rafiq, R., Bhat, G. A., Lone, M. M., Masood, A. and Dar, N. A. | 2016 | Potential risk of esophageal squamous cell carcinoma due to nucleotide excision repair XPA and XPC gene variants and their interaction among themselves and with environmental factors | Tumour Biol | 10.1007/s13277-016-4895-3 |
| **367** | Rafiq, R., Shah, I. A., Bhat, G. A., Lone, M. M., Islami, F., Boffetta, P. and Dar, N. A. | 2016 | Secondhand Smoking and the Risk of Esophageal Squamous Cell Carcinoma in a High Incidence Region, Kashmir, India: A Case-control-observational Study | Medicine (Baltimore) | 10.1097/md.0000000000002340 |
| **368** | Raghu, G., Morrow, E., Collins, B. F., Ho, L. A., Hinojosa, M. W., Hayes, J. M., Spada, C. A., Oelschlager, B., Li, C., Yow, E., Anstrom, K. J., Mart, D., Xiao, K. and Pellegrini, C. A. | 2016 | Laparoscopic anti-reflux surgery for idiopathic pulmonary fibrosis at a single centre | Eur Respir J | 10.1183/13993003.00488-2016 |
| **369** | Rai, S., Kulkarni, A. and Ghoshal, U. C. | 2021 | Prevalence and risk factors for gastroesophageal reflux disease in the Indian population: A meta-analysis and meta-regression study | Indian J Gastroenterol | 10.1007/s12664-020-01104-0 |
| **370** | Rameez, M. H. and Mayberry, J. F. | 2015 | Epidemiology and risk factors for Barrett's oesophagus | Br J Hosp Med (Lond) | 10.12968/hmed.2015.76.3.138 |
| **371** | Ramôa, C. P., Eissenberg, T. and Sahingur, S. E. | 2017 | Increasing popularity of waterpipe tobacco smoking and electronic cigarette use: Implications for oral healthcare | J Periodontal Res | 10.1111/jre.12458 |
| **372** | Ramos, Mfkp, Ribeiro Júnior, U., Viscondi, J. K. Y., Zilberstein, B., Cecconello, I. and Eluf-Neto, J. | 2018 | Risk factors associated with the development of gastric cancer - case-control study | Rev Assoc Med Bras (1992) | 10.1590/1806-9282.64.07.611 |
| **373** | Ranaldo, N., Losurdo, G., Iannone, A., Principi, M., Barone, M., De Carne, M., Ierardi, E. and Di Leo, A. | 2017 | Tailored therapy guided by multichannel intraluminal impedance pH monitoring for refractory non-erosive reflux disease | Cell Death Dis | 10.1038/cddis.2017.436 |
| **374** | Rantanen, T., Oksala, N. and Sand, J. | 2016 | Adenocarcinoma of the Oesophagus and Oesophagogastric Junction: Analysis of Incidence and Risk Factors | Anticancer Res |  |
| **375** | Rao, S. J., Kirse, D. J. and Shetty, A. K. | 2021 | Cannabis induced thermal epiglottitis in a pediatric patient | Am J Emerg Med | 10.1016/j.ajem.2021.05.024 |
| **376** | Rao, Z., Xie, X., Tang, X., Peng, H., Zheng, Z., Hu, Z. and Peng, X. | 2022 | The spatiotemporal correlation of PM(2.5) concentration on esophageal cancer hospitalization rate in Fujian province of China | Environ Sci Pollut Res Int | 10.1007/s11356-022-20587-2 |
| **377** | Refaat, T., Choi, M., Thomas, T. O., Bacchus, I., Agulnik, M., Pelzer, H. J., Mellott, A. L., Rademaker, A. W., Liu, D., Sathiaseelan, V. and Mittal, B. B. | 2015 | Whole-Field Sequential Intensity-Modulated Radiotherapy for Local-Regional Advanced Head-and-Neck Squamous Cell Carcinoma | Am J Clin Oncol | 10.1097/coc.0000000000000001 |
| **378** | Roa Colomo, A., García Robles, A. and Ruiz Escolano, E. | 2019 | Black esophagus, is it as bad as it seems? | Gastroenterol Hepatol | 10.1016/j.gastrohep.2019.06.004 |
| **379** | Rodríguez-Lago, I., Calderón, Á, Cazallas, J., Camino, M. E., Barredo, I. and Cabriada, J. L. | 2017 | First case report of oesophageal actinomycosis in a patient with active eosinophilic oesophagitis | Gastroenterol Hepatol | 10.1016/j.gastrohep.2016.04.012 |
| **380** | Rosenthal, D. I., Mohamed, A. S., Weber, R. S., Garden, A. S., Sevak, P. R., Kies, M. S., Morrison, W. H., Lewin, J. S., El-Naggar, A. K., Ginsberg, L. E., Kocak-Uzel, E., Ang, K. K. and Fuller, C. D. | 2015 | Long-term outcomes after surgical or nonsurgical initial therapy for patients with T4 squamous cell carcinoma of the larynx: A 3-decade survey | Cancer | 10.1002/cncr.29241 |
| **381** | Rota, M., Possenti, I., Valsassina, V., Santucci, C., Bagnardi, V., Corrao, G., Bosetti, C., Specchia, C., Gallus, S. and Lugo, A. | 2024 | Dose-response association between cigarette smoking and gastric cancer risk: a systematic review and meta-analysis | Gastric Cancer | 10.1007/s10120-023-01459-1 |
| **382** | Rubenstein, J. H., Morgenstern, H. and Longstreth, K. | 2019 | Clustering of esophageal cancer among white men in the United States | Dis Esophagus | 10.1093/dote/doy081 |
| **383** | Ruiz-Tovar, J., Díaz, G., Alias, D., Jiménez-Fuertes, M. and Durán, M. | 2016 | Hemoperitoneum secondary to an spontaneous rupture of the spleen mimmicking a duodenal perforated ulcera: A case report | Rev Esp Enferm Dig |  |
| **384** | Runge, T. M., Abrams, J. A. and Shaheen, N. J. | 2015 | Epidemiology of Barrett's Esophagus and Esophageal Adenocarcinoma | Gastroenterol Clin North Am | 10.1016/j.gtc.2015.02.001 |
| **385** | Sabău, D., Dumitra, A., Sabău, A., Maniu, D., Mitachescu, A., Ilie, S., Hulpus, R. and Smarandache, G. | 2015 | Esotracheal Fistula in Esophageal Stenoses of Malignant Origin - Case Report | Chirurgia (Bucur) |  |
| **386** | Sack, C. and Raghu, G. | 2019 | Idiopathic pulmonary fibrosis: unmasking cryptogenic environmental factors | Eur Respir J | 10.1183/13993003.01699-2018 |
| **387** | Sadafi, S., Azizi, A., Pasdar, Y., Shakiba, E. and Darbandi, M. | 2024 | Risk factors for gastroesophageal reflux disease: a population-based study | BMC Gastroenterol | 10.1186/s12876-024-03143-9 |
| **388** | Saikia, S., Rehman, A. U., Barooah, P., Sarmah, P., Bhattacharyya, M., Deka, M., Deka, M., Goswami, B., Husain, S. A. and Medhi, S. | 2017 | Alteration in the expression of MGMT and RUNX3 due to non-CpG promoter methylation and their correlation with different risk factors in esophageal cancer patients | Tumour Biol | 10.1177/1010428317701630 |
| **389** | Sakthivel, P., Samy, K., Panda, S. and Amit Singh, C. | 2021 | 14 "S" in head and neck cancers | Oral Oncol | 10.1016/j.oraloncology.2020.105070 |
| **390** | Saleem, S., Tarar, Z. I., Aziz, M., Alsamman, M. A., Tansel, A. and Abell, T. L. | 2023 | Cannabis Use in Patients with Gastroparesis | Cannabis Cannabinoid Res | 10.1089/can.2022.0189 |
| **391** | Şanlı, A., Bekmez, E., Yıldız, G., Erdoğan, B. A., Yılmaz, H. B. and Altın, G. | 2016 | Relationship between smoking and otorhinolaryngological symptoms | Kulak Burun Bogaz Ihtis Derg | 10.5606/kbbihtisas.2016.87059 |
| **392** | Sardana, R. K., Chhikara, N., Tanwar, B. and Panghal, A. | 2018 | Dietary impact on esophageal cancer in humans: a review | Food Funct | 10.1039/c7fo01908d |
| **393** | Schiöler, L., Ruth, M., Jõgi, R., Gislason, T., Storaas, T., Janson, C., Forsberg, B., Sigsgaard, T., Torén, K. and Hellgren, J. | 2015 | Nocturnal GERD - a risk factor for rhinitis/rhinosinusitis: the RHINE study | Allergy | 10.1111/all.12615 |
| **394** | Schmidt, M., Ankerst, D. P., Chen, Y., Wiethaler, M., Slotta-Huspenina, J., Becker, K. F., Horstmann, J., Kohlmayer, F., Lehmann, A., Linkohr, B., Strauch, K., Schmid, R. M., Quante, A. S. and Quante, M. | 2020 | Epidemiologic Risk Factors in a Comparison of a Barrett Esophagus Registry (BarrettNET) and a Case-Control Population in Germany | Cancer Prev Res (Phila) | 10.1158/1940-6207.Capr-19-0474 |
| **395** | Schuman, A. D., Birkeland, A. C., Farlow, J. L., Lyden, T., Blakely, A., Spector, M. E. and Rosko, A. J. | 2021 | Predictors of Stricture and Swallowing Function Following Salvage Laryngectomy | Laryngoscope | 10.1002/lary.29215 |
| **396** | Scida, S., Russo, M., Miraglia, C., Leandro, G., Franzoni, L., Meschi, T., De' Angelis, G. L. and Di Mario, F. | 2018 | Relationship between Helicobacter pylori infection and GERD | Acta Biomed | 10.23750/abm.v89i8-S.7918 |
| **397** | Sengngam, K., Hoc, T. H., Phuoc, L. H., Hang, D. V. and Ngoan, L. T. | 2022 | Interaction of Helicobacter pylori Infection with Waterpipe Tobacco Smoking in the Development of Stomach Cancer in Vietnamese Men | Asian Pac J Cancer Prev | 10.31557/apjcp.2022.23.4.1199 |
| **398** | Seo, H. S., Hong, J. and Jung, J. | 2020 | Relationship of meteorological factors and air pollutants with medical care utilization for gastroesophageal reflux disease in urban area | World J Gastroenterol | 10.3748/wjg.v26.i39.6074 |
| **399** | Sethi, S. and Richter, J. E. | 2017 | Diet and gastroesophageal reflux disease: role in pathogenesis and management | Curr Opin Gastroenterol | 10.1097/mog.0000000000000337 |
| **400** | Sevinc, M. M., Kinaci, E., Bayrak, S., Yardimci, A. H., Cakar, E. and Bektaş, H. | 2015 | Extraordinary cause of acute gastric dilatation and hepatic portal venous gas: Chronic use of synthetic cannabinoid | World J Gastroenterol | 10.3748/wjg.v21.i37.10704 |
| **401** | Sewram, V., Sitas, F., O'Connell, D. and Myers, J. | 2016 | Tobacco and alcohol as risk factors for oesophageal cancer in a high incidence area in South Africa | Cancer Epidemiol | 10.1016/j.canep.2016.02.001 |
| **402** | Shah, I. A., Bhat, G. A., Mehta, P., Lone, M. M. and Dar, N. A. | 2016 | Genotypes of CYP1A1, SULT1A1 and SULT1A2 and risk of squamous cell carcinoma of esophagus: outcome of a case-control study from Kashmir, India | Dis Esophagus | 10.1111/dote.12427 |
| **403** | Shah, S. C., Nunez, H., Chiu, S., Hazan, A., Chen, S., Wang, S., Itzkowitz, S. and Jandorf, L. | 2020 | Low baseline awareness of gastric cancer risk factors amongst at-risk multiracial/ethnic populations in New York City: results of a targeted, culturally sensitive pilot gastric cancer community outreach program | Ethn Health | 10.1080/13557858.2017.1398317 |
| **404** | Shao, Y., Guo, X., Zhao, L., Shen, Y., Niu, C., Wei, W. and Liu, F. | 2020 | A Functional Variant of the miR-15 Family Is Associated with a Decreased Risk of Esophageal Squamous Cell Carcinoma | DNA Cell Biol | 10.1089/dna.2020.5606 |
| **405** | Shaw, D. E., Sousa, A. R., Fowler, S. J., Fleming, L. J., Roberts, G., Corfield, J., Pandis, I., Bansal, A. T., Bel, E. H., Auffray, C., Compton, C. H., Bisgaard, H., Bucchioni, E., Caruso, M., Chanez, P., Dahlén, B., Dahlen, S. E., Dyson, K., Frey, U., Geiser, T., Gerhardsson de Verdier, M., Gibeon, D., Guo, Y. K., Hashimoto, S., Hedlin, G., Jeyasingham, E., Hekking, P. P., Higenbottam, T., Horváth, I., Knox, A. J., Krug, N., Erpenbeck, V. J., Larsson, L. X., Lazarinis, N., Matthews, J. G., Middelveld, R., Montuschi, P., Musial, J., Myles, D., Pahus, L., Sandström, T., Seibold, W., Singer, F., Strandberg, K., Vestbo, J., Vissing, N., von Garnier, C., Adcock, I. M., Wagers, S., Rowe, A., Howarth, P., Wagener, A. H., Djukanovic, R., Sterk, P. J. and Chung, K. F. | 2015 | Clinical and inflammatory characteristics of the European U-BIOPRED adult severe asthma cohort | Eur Respir J | 10.1183/13993003.00779-2015 |
| **406** | Shephard, E. A., Parkinson, M. A. and Hamilton, W. T. | 2019 | Recognising laryngeal cancer in primary care: a large case-control study using electronic records | Br J Gen Pract | 10.3399/bjgp19X700997 |
| **407** | Shivappa, N., Hébert, J. R. and Rashidkhani, B. | 2015 | Dietary Inflammatory Index and Risk of Esophageal Squamous Cell Cancer in a Case-Control Study from Iran | Nutr Cancer | 10.1080/01635581.2015.1082108 |
| **408** | Short, M. W., Burgers, K. G. and Fry, V. T. | 2017 | Esophageal Cancer | Am Fam Physician |  |
| **409** | Siddiqi, K., Shah, S., Abbas, S. M., Vidyasagaran, A., Jawad, M., Dogar, O. and Sheikh, A. | 2015 | Global burden of disease due to smokeless tobacco consumption in adults: analysis of data from 113 countries | BMC Med | 10.1186/s12916-015-0424-2 |
| **410** | Simba, H., Menya, D., Mmbaga, B. T., Dzamalala, C., Finch, P., Mlombe, Y., Mremi, A., Narh, C. T., Schüz, J. and McCormack, V. | 2023 | The contribution of smoking and smokeless tobacco to oesophageal squamous cell carcinoma risk in the African oesophageal cancer corridor: Results from the ESCCAPE multicentre case-control studies | Int J Cancer | 10.1002/ijc.34458 |
| **411** | Singhi, A. D., Seethala, R. R., Nason, K., Foxwell, T. J., Roche, R. L., McGrath, K. M., Levy, R. M., Luketich, J. D. and Davison, J. M. | 2015 | Undifferentiated carcinoma of the esophagus: a clinicopathological study of 16 cases | Hum Pathol | 10.1016/j.humpath.2014.11.021 |
| **412** | Slae, M., Persad, R., Leung, A. J., Gabr, R., Brocks, D. and Huynh, H. Q. | 2015 | Role of Environmental Factors in the Development of Pediatric Eosinophilic Esophagitis | Dig Dis Sci | 10.1007/s10620-015-3740-7 |
| **413** | Soldatova, L., Hrelec, C. and Matrka, L. | 2016 | Can PFTS Differentiate PVFMD From Subglottic Stenosis? | Ann Otol Rhinol Laryngol | 10.1177/0003489416665195 |
| **414** | Song, D. H., Kim, N., Jo, H. H., Kim, S., Choi, Y., Oh, H. J., Lee, H. S., Yoon, H., Shin, C. M., Park, Y. S., Lee, D. H., Kang, S. H., Park, Y. S., Ahn, S. H., Suh, Y. S., Park, D. J., Kim, H. H., Kim, J. W., Kim, J. W., Lee, K. W., Chang, W., Park, J. H., Lee, Y. J., Lee, K. H., Kim, Y. H., Ahn, S. and Surh, Y. J. | 2024 | Analysis of Characteristics and Risk Factors of Patients with Single Gastric Cancer and Synchronous Multiple Gastric Cancer among 14,603 Patients | Gut Liver | 10.5009/gnl220491 |
| **415** | Song, J. H., Yang, S. Y., Lim, J. H., Choi, J. M. and Kim, S. G. | 2017 | The Effect of Helicobacter pylori Eradication on the Metachronous Neoplasm after Endoscopic Resection for Gastric Dysplasia | Korean J Gastroenterol | 10.4166/kjg.2017.70.1.27 |
| **416** | Song, M., Choi, J. Y., Yang, J. J., Sung, H., Lee, Y., Lee, H. W., Kong, S. H., Lee, H. J., Kim, H. H., Kim, S. G., Yang, H. K. and Kang, D. | 2015 | Obesity at adolescence and gastric cancer risk | Cancer Causes Control | 10.1007/s10552-014-0506-z |
| **417** | Song, Q., Zhang, Z., Liu, Y., Han, S. and Zhang, X. | 2015 | The tag SNP rs10746463 in decay-accelerating factor is associated with the susceptibility to gastric cancer | Mol Immunol | 10.1016/j.molimm.2014.10.006 |
| **418** | Sonnenberg, W. R. | 2017 | Gastrointestinal Malignancies | Prim Care | 10.1016/j.pop.2017.07.013 |
| **419** | Soroush, A., Malekzadeh, R., Roshandel, G., Khoshnia, M., Poustchi, H., Kamangar, F., Brennan, P., Boffetta, P., Dawsey, S. M., Abnet, C. C., Abrams, J. A. and Etemadi, A. | 2023 | Sex and smoking differences in the association between gastroesophageal reflux and risk of esophageal squamous cell carcinoma in a high-incidence area: Golestan Cohort Study | Int J Cancer | 10.1002/ijc.34313 |
| **420** | Spaniolas, K., Yang, J., Crowley, S., Yin, D., Docimo, S., Bates, A. T. and Pryor, A. D. | 2018 | Association of Long-term Anastomotic Ulceration After Roux-en-Y Gastric Bypass With Tobacco Smoking | JAMA Surg | 10.1001/jamasurg.2018.1616 |
| **421** | Spantideas, N., Drosou, E., Karatsis, A. and Assimakopoulos, D. | 2015 | Voice disorders in the general Greek population and in patients with laryngopharyngeal reflux. Prevalence and risk factors | J Voice | 10.1016/j.jvoice.2014.08.006 |
| **422** | Splittgerber, M. and Velanovich, V. | 2015 | Barrett esophagus | Surg Clin North Am | 10.1016/j.suc.2015.02.011 |
| **423** | Spreafico, A., Coate, L., Zhai, R., Xu, W., Chen, Z. F., Chen, Z., Patel, D., Tse, B., Brown, M. C., Heist, R. S., Dodbiba, L., Teichman, J., Kulke, M., Su, L., Eng, L., Knox, J., Wong, R., Darling, G. E., Christiani, D. C. and Liu, G. | 2017 | Early adulthood body mass index, cumulative smoking, and esophageal adenocarcinoma survival | Cancer Epidemiol | 10.1016/j.canep.2016.11.009 |
| **424** | Sun, D., Liu, C., Zhu, Y., Yu, C., Guo, Y., Sun, D., Pang, Y., Pei, P., Du, H., Yang, L., Chen, Y., Meng, X., Liu, Y., Zhang, J., Schmidt, D., Avery, D., Chen, J., Chen, Z., Lv, J., Kan, H. and Li, L. | 2023 | Long-Term Exposure to Fine Particulate Matter and Incidence of Esophageal Cancer: A Prospective Study of 0.5 Million Chinese Adults | Gastroenterology | 10.1053/j.gastro.2023.03.233 |
| **425** | Sun, D. and Ye, Q. | 2023 | Mendelian randomization analysis suggests no causal influence of gastroesophageal reflux disease on the susceptibility and prognosis of idiopathic pulmonary fibrosis | BMC Pulm Med | 10.1186/s12890-023-02788-8 |
| **426** | Sun, L., Tu, H., Liu, J., Gong, Y., Xu, Q., Jing, J., Dong, N. and Yuan, Y. | 2014 | A comprehensive evaluation of fasting serum gastrin-17 as a predictor of diseased stomach in Chinese population | Scand J Gastroenterol | 10.3109/00365521.2014.950693 |
| **427** | Suwanwongse, K. and Shabarek, N. | 2020 | Epidemiology, clinical features, and outcomes of hospitalized infants with COVID-19 in the Bronx, New York | Arch Pediatr | 10.1016/j.arcped.2020.07.009 |
| **428** | Svenningsson, A., Svensson, T., Akre, O. and Nordenskjöld, A. | 2014 | Maternal and pregnancy characteristics and risk of infantile hypertrophic pyloric stenosis | J Pediatr Surg | 10.1016/j.jpedsurg.2014.01.053 |
| **429** | Taha, F., Lipsitz, J. D., Galea, S., Demmer, R. T., Talley, N. J. and Goodwin, R. D. | 2014 | Anxiety disorders and risk of self-reported ulcer: a 10-year longitudinal study among US adults | Gen Hosp Psychiatry | 10.1016/j.genhosppsych.2014.07.005 |
| **430** | Takeuchi, T., Oota, K., Harada, S., Edogawa, S., Kojima, Y., Sanomura, M., Sakaguchi, M., Hayashi, K., Hongoh, Y., Itabashi, T., Kitae, H., Hoshimoto, M., Takeuchi, N. and Higuchi, K. | 2015 | Characteristics of refractory gastroesophageal reflux disease (GERD) symptoms -is switching proton pump inhibitors based on the patient's CYP2C19 genotype an effective management strategy? | Intern Med | 10.2169/internalmedicine.54.3412 |
| **431** | Talagala, I. A., Nawarathne, M. and Arambepola, C. | 2018 | Novel risk factors for primary prevention of oesophageal carcinoma: a case-control study from Sri Lanka | BMC Cancer | 10.1186/s12885-018-4975-4 |
| **432** | Tasneem, S., Sarwar, M. T., Bashir, M. R., Hussain, H., Ahmed, J. and Pervez, S. | 2018 | Expression analysis of cyclooxygenase-2 in patients suffering from esophageal squamous cell carcinoma | PLoS One | 10.1371/journal.pone.0205508 |
| **433** | Tavaluc, R. and Tan-Geller, M. | 2019 | Reinke's Edema | Otolaryngol Clin North Am | 10.1016/j.otc.2019.03.006 |
| **434** | Tedla, M., Chakrabarti, S., Suchankova, M. and Weickert, M. O. | 2016 | Voice outcomes after thyroidectomy without superior and recurrent laryngeal nerve injury: VoiSS questionnaire and GRBAS tool assessment | Eur Arch Otorhinolaryngol | 10.1007/s00405-016-4163-6 |
| **435** | Teixeira, L., Manso, M. C. and Manarte-Monteiro, P. | 2018 | Oral Health-Related Quality of Life Among a Portuguese Sample of Institutionalised Alcoholic Patients under Rehabilitation Therapy | Oral Health Prev Dent | 10.3290/j.ohpd.a40719 |
| **436** | Thrift, A. P. | 2016 | Determination of risk for Barrett's esophagus and esophageal adenocarcinoma | Curr Opin Gastroenterol | 10.1097/mog.0000000000000274 |
| **437** | Thrift, A. P. | 2016 | The epidemic of oesophageal carcinoma: Where are we now? | Cancer Epidemiol | 10.1016/j.canep.2016.01.013 |
| **438** | Thrift, A. P., Jove, A. G., Liu, Y., Tan, M. C. and El-Serag, H. B. | 2022 | Associations of Duration, Intensity, and Quantity of Smoking With Risk of Gastric Intestinal Metaplasia | J Clin Gastroenterol | 10.1097/mcg.0000000000001479 |
| **439** | Thrift, A. P., Kramer, J. R., Hartman, C. M., Royse, K., Richardson, P., Dong, Y., Raychaudhury, S., Desiderio, R., Sanchez, D., Anandasabapathy, S., White, D. L. and Chiao, E. Y. | 2019 | Risk and Predictors of Esophageal and Stomach Cancers in HIV-Infected Veterans: A Matched Cohort Study | J Acquir Immune Defic Syndr | 10.1097/qai.0000000000002038 |
| **440** | Thrumurthy, S. G., Chaudry, M. A., Thrumurthy, S. S. D. and Mughal, M. | 2019 | Oesophageal cancer: risks, prevention, and diagnosis | Bmj | 10.1136/bmj.l4373 |
| **441** | Torre, L. A., Bray, F., Siegel, R. L., Ferlay, J., Lortet-Tieulent, J. and Jemal, A. | 2015 | Global cancer statistics, 2012 | CA Cancer J Clin | 10.3322/caac.21262 |
| **442** | Torres-Román, A. L., Rodríguez-Flores, K. L., Hernández-Mora, V. M., Ruiz-García, E., Prospero-García, O., Guijosa, A., Molina, A., Morales-Mulia, M., Aschner, M., Santamaría, A. and Ortega-Gómez, A. | 2023 | Examining the Role of Histaminergic, Orexinergic, and Cannabinergic Systems in Redox Regulation in Gastric Adenocarcinoma | Mini Rev Med Chem | 10.2174/1389557523666230221104504 |
| **443** | Trigueros, J. A., Plaza, V., Domínguez-Ortega, J., Serrano, J., Cisneros, C., Padilla, A., Antón Gironés, M., Mosteiro, M., Martínez Moragón, E., Olaguíbel Rivera, J. M., Delgado, J., García Rivero, J. L., Martínez Rivera, C., Garrido, J. J. and Quirce, S. | 2020 | Asthma, Comorbidities, and Aggravating Circumstances: The GEMA-FORUM II Task Force | J Investig Allergol Clin Immunol | 10.18176/jiaci.0460 |
| **444** | Tsai, S. S., Chiu, H. F. and Yang, C. Y. | 2019 | Ambient Air Pollution and Hospital Admissions for Peptic Ulcers in Taipei: A Time-Stratified Case-Crossover Study | Int J Environ Res Public Health | 10.3390/ijerph16111916 |
| **445** | Uchihara, T., Yoshida, N., Baba, Y., Yagi, T., Toihata, T., Oda, E., Kuroda, D., Eto, T., Ohuchi, M., Nakamura, K., Sawayama, H., Kinoshita, K., Iwatsuki, M., Ishimoto, T., Sakamoto, Y. and Baba, H. | 2018 | Risk factors for pulmonary morbidities after minimally invasive esophagectomy for esophageal cancer | Surg Endosc | 10.1007/s00464-017-5993-z |
| **446** | Udasin, I. G., Sunderram, J. and Calvert, G. | 2023 | The World Trade Center Health Program: Obstructive sleep apnea best practices | Arch Environ Occup Health | 10.1080/19338244.2023.2195604 |
| **447** | Ueda, K., Ohishi, W., Cullings, H., Fujiwara, S., Suzuki, G., Hayashi, T., Mitsui, F., Hida, A., Ozasa, K., Ito, M., Chayama, K. and Tahara, E. | 2020 | Modifying Effect of Chronic Atrophic Gastritis on Radiation Risk for Noncardia Gastric Cancer According to Histological Type | Radiat Res | 10.1667/rr15482.1 |
| **448** | Ugwuegbu, O., Shibli, F., Kim, Y., Rangan, V., Kurin, M., Ayoub, F., Ganocy, S., Kavitt, R. and Fass, R. | 2024 | The Impact of Chronic Cannabis Use on Esophageal Motility in Patients Referred for Esophageal Manometry | J Clin Gastroenterol | 10.1097/mcg.0000000000001887 |
| **449** | Underner, M. | 2008 | [Underrated effects of tobacco and marijuana smoking on the thyroid, the esophagus, the kidney, the skeletal system and the mouth] | Rev Mal Respir | 10.1016/s0761-8425(08)75108-8 |
| **450** | Usai-Satta, P., Bellini, M., Morelli, O., Geri, F., Lai, M. and Bassotti, G. | 2020 | Gastroparesis: New insights into an old disease | World J Gastroenterol | 10.3748/wjg.v26.i19.2333 |
| **451** | Valdez-Solis, E. M., Ramírez-Rentería, C., Ferreira-Hermosillo, A., Molina-Ayala, M., Mendoza-Zubieta, V. and Rodríguez-Pérez, V. | 2017 | Gastroesophageal reflux disease in patients with long standing type 1 diabetes mellitus: utility of two self-report questionnaires in a multifactorial disease | Colomb Med (Cali) | 10.25100/cm.v48i3.2801 |
| **452** | van Boven, J. F., Román-Rodríguez, M., Palmer, J. F., Toledo-Pons, N., Cosío, B. G. and Soriano, J. B. | 2016 | Comorbidome, Pattern, and Impact of Asthma-COPD Overlap Syndrome in Real Life | Chest | 10.1016/j.chest.2015.12.002 |
| **453** | Vogelsang, M., Paccez, J. D., Schäfer, G., Dzobo, K., Zerbini, L. F. and Parker, M. I. | 2014 | Aberrant methylation of the MSH3 promoter and distal enhancer in esophageal cancer patients exposed to first-hand tobacco smoke | J Cancer Res Clin Oncol | 10.1007/s00432-014-1736-x |
| **454** | Vogtmann, E., Flores, R., Yu, G., Freedman, N. D., Shi, J., Gail, M. H., Dye, B. A., Wang, G. Q., Klepac-Ceraj, V., Paster, B. J., Wei, W. Q., Guo, H. Q., Dawsey, S. M., Qiao, Y. L. and Abnet, C. C. | 2015 | Association between tobacco use and the upper gastrointestinal microbiome among Chinese men | Cancer Causes Control | 10.1007/s10552-015-0535-2 |
| **455** | Vyas, M. V., Laupacis, A., Austin, P. C., Fang, J., Silver, F. L. and Kapral, M. K. | 2020 | Association Between Immigration Status and Acute Stroke Care: A Retrospective Study | Stroke | 10.1161/strokeaha.119.027791 |
| **456** | Waki, K., Ishihara, R., Maekawa, A., Inoue, T., Shoji, A., Matsueda, K., Miyake, M., Fukuda, H., Shichijo, S., Kanesaka, T., Takeuchi, Y., Higashino, K., Uedo, N. and Michida, T. | 2021 | Endoscopic findings in the soft palatal mucosa are associated with the risk of esophageal squamous cell carcinoma | J Gastroenterol Hepatol | 10.1111/jgh.15291 |
| **457** | Wang, C., Guan, S., Chen, X., Liu, B., Liu, F., Han, L., Un Nesa, E., Song, Q., Bao, C., Wang, X. and Cheng, Y. | 2015 | Clinical potential of miR-3651 as a novel prognostic biomarker for esophageal squamous cell cancer | Biochem Biophys Res Commun | 10.1016/j.bbrc.2015.07.109 |
| **458** | Wang, G., Ye, M., Zheng, S., Wu, K., Geng, H. and Liu, C. | 2020 | Cigarette Smoke Extract induces H19 in Esophageal Squamous Cell Carcinoma in Smoking Patients: Based on A Chronic Exposed Cell Model | Toxicol Lett | 10.1016/j.toxlet.2020.07.030 |
| **459** | Wang, H. Y., Leena, K. B., Plymoth, A., Hergens, M. P., Yin, L., Shenoy, K. T. and Ye, W. | 2016 | Prevalence of gastro-esophageal reflux disease and its risk factors in a community-based population in southern India | BMC Gastroenterol | 10.1186/s12876-016-0452-1 |
| **460** | Wang, J., Qiu, M., Xu, Y., Li, M., Dong, G., Mao, Q., Yin, R. and Xu, L. | 2015 | Long noncoding RNA CCAT2 correlates with smoking in esophageal squamous cell carcinoma | Tumour Biol | 10.1007/s13277-015-3220-x |
| **461** | Wang, J., Shen, H., Fu, G., Zhao, D. and Wang, W. | 2017 | Nuclear overexpression of the overexpressed in lung cancer 1 predicts worse prognosis in gastric adenocarcinoma | Oncotarget | 10.18632/oncotarget.14217 |
| **462** | Wang, L., Du, L., Xiong, X., Lin, Y., Zhu, J., Yao, Z., Wang, S., Guo, Y., Chen, Y., Geary, K., Pan, Y., Zhou, F., Gao, S., Zhang, D., Yeung, S. J. and Zhang, H. | 2021 | Repurposing dextromethorphan and metformin for treating nicotine-induced cancer by directly targeting CHRNA7 to inhibit JAK2/STAT3/SOX2 signaling | Oncogene | 10.1038/s41388-021-01682-z |
| **463** | Wang, L., Xiao, S., Zheng, Y. and Gao, Z. | 2022 | Interaction Between Vascular Endothelial Growth Factor Gene Polymorphism and Smoking on Gastric Cancer Risk in Chinese Han Population | Pathol Oncol Res | 10.3389/pore.2022.1610495 |
| **464** | Wang, N., Yang, J., Lu, J., Qiao, Q., Bao, G., Wu, T. and He, X. | 2014 | IL-17 gene polymorphism is associated with susceptibility to gastric cancer | Tumour Biol | 10.1007/s13277-014-2255-8 |
| **465** | Wang, Q. L., Lagergren, J. and Xie, S. H. | 2019 | Prediction of individuals at high absolute risk of esophageal squamous cell carcinoma | Gastrointest Endosc | 10.1016/j.gie.2018.10.025 |
| **466** | Wang, S. M., Katki, H. A., Graubard, B. I., Kahle, L. L., Chaturvedi, A., Matthews, C. E., Freedman, N. D. and Abnet, C. C. | 2021 | Population Attributable Risks of Subtypes of Esophageal and Gastric Cancers in the United States | Am J Gastroenterol | 10.14309/ajg.0000000000001355 |
| **467** | Wang, T., Ma, L., Yang, D. L., Wang, H., Bai, Z. L., Zhang, L. J. and Ding, W. Y. | 2017 | Factors predicting dysphagia after anterior cervical surgery: A multicenter retrospective study for 2 years of follow-up | Medicine (Baltimore) | 10.1097/md.0000000000007916 |
| **468** | Wang, V. L., Jalilvand, A. D., Gupta, A., Chen, J., Vadlamudi, C. and Perry, K. A. | 2021 | Tobacco use is not associated with increased risk of recurrent reflux 5 years after laparoscopic anti-reflux surgery | Surg Endosc | 10.1007/s00464-020-07956-z |
| **469** | Wang, W. L., Chang, I. W., Chen, C. C., Chang, C. Y., Mo, L. R., Lin, J. T., Wang, H. P. and Lee, C. T. | 2015 | Radiofrequency Ablation Versus Endoscopic Submucosal Dissection in Treating Large Early Esophageal Squamous Cell Neoplasia | Medicine (Baltimore) | 10.1097/md.0000000000002240 |
| **470** | Wang, Y., Jiang, Y. and Zhu, Z. | 2021 | Question on Databases Used to Examine the Association Between Gastric Cancer and Heavy Alcohol Use | Am J Gastroenterol | 10.14309/ajg.0000000000001392 |
| **471** | Wang, Y., Shen, C., Ge, J. and Duan, H. | 2015 | Regular aspirin use and stomach cancer risk in China | Eur J Surg Oncol | 10.1016/j.ejso.2015.02.006 |
| **472** | Wang, Z., Koh, W. P., Jin, A., Wang, R. and Yuan, J. M. | 2017 | Composite protective lifestyle factors and risk of developing gastric adenocarcinoma: the Singapore Chinese Health Study | Br J Cancer | 10.1038/bjc.2017.7 |
| **473** | Wang, Z., Koh, W. P., Jin, A., Wang, R. and Yuan, J. M. | 2018 | Telomere length and risk of developing gastric adenocarcinoma: The Singapore Chinese Health Study | Gastric Cancer | 10.1007/s10120-017-0783-9 |
| **474** | Watanabe, K. | 2017 | Black hairy tongue | Acta Otorrinolaringol Esp (Engl Ed) | 10.1016/j.otorri.2016.10.002 |
| **475** | Wei, M., Zhao, L., Lv, J., Li, X., Zhou, G., Fan, B., Shen, X., Zhao, D., Xue, F., Wang, J. and Zhang, T. | 2021 | The mediation effect of serum metabolites on the relationship between long-term smoking exposure and esophageal squamous cell carcinoma | BMC Cancer | 10.1186/s12885-021-08151-6 |
| **476** | Weinmayr, G., Chen, J., Jaensch, A., Skodda, L., Rodopoulou, S., Strak, M., de Hoogh, K., Andersen, Z. J., Bellander, T., Brandt, J., Fecht, D., Forastiere, F., Gulliver, J., Hertel, O., Hoffmann, B., Hvidtfeldt, U. A., Katsouyanni, K., Ketzel, M., Leander, K., Magnusson, P. K. E., Pershagen, G., Rizzuto, D., Samoli, E., Severi, G., Stafoggia, M., Tjønneland, A., Vermeulen, R., Wolf, K., Zitt, E., Brunekreef, B., Thurston, G., Hoek, G., Raaschou-Nielsen, O. and Nagel, G. | 2024 | Long-term exposure to several constituents and sources of PM(2.5) is associated with incidence of upper aerodigestive tract cancers but not gastric cancer: Results from the large pooled European cohort of the ELAPSE project | Sci Total Environ | 10.1016/j.scitotenv.2023.168789 |
| **477** | Weinmayr, G., Pedersen, M., Stafoggia, M., Andersen, Z. J., Galassi, C., Munkenast, J., Jaensch, A., Oftedal, B., Krog, N. H., Aamodt, G., Pyko, A., Pershagen, G., Korek, M., De Faire, U., Pedersen, N. L., Östenson, C. G., Rizzuto, D., Sørensen, M., Tjønneland, A., Bueno-de-Mesquita, B., Vermeulen, R., Eeftens, M., Concin, H., Lang, A., Wang, M., Tsai, M. Y., Ricceri, F., Sacerdote, C., Ranzi, A., Cesaroni, G., Forastiere, F., de Hoogh, K., Beelen, R., Vineis, P., Kooter, I., Sokhi, R., Brunekreef, B., Hoek, G., Raaschou-Nielsen, O. and Nagel, G. | 2018 | Particulate matter air pollution components and incidence of cancers of the stomach and the upper aerodigestive tract in the European Study of Cohorts of Air Pollution Effects (ESCAPE) | Environ Int | 10.1016/j.envint.2018.07.030 |
| **478** | Weinreb, S. F., Piersiala, K., Hillel, A. T., Akst, L. M. and Best, S. R. | 2021 | Dysphonia and dysphagia as early manifestations of autoimmune inflammatory myopathy | Am J Otolaryngol | 10.1016/j.amjoto.2020.102747 |
| **479** | Wen, J., Pang, Y., Zhou, T., Qi, X., Zhao, M., Xuan, B., Meng, X., Guo, Y., Liu, Q., Liang, H., Li, Y., Dong, H. and Wang, Y. | 2016 | Essential role of Na+/Ca2+ exchanger 1 in smoking-induced growth and migration of esophageal squamous cell carcinoma | Oncotarget | 10.18632/oncotarget.11695 |
| **480** | Wen, Q., Mao, X., Shi, X., Wang, Y. and Wang, J. | 2023 | Impacts of heavy smoking on non-coding RNA expression for patients with esophageal carcinoma | BMC Med Genomics | 10.1186/s12920-023-01574-z |
| **481** | Westra, W. M., Lutzke, L. S., Mostafavi, N. S., Roes, A. L., Calpe, S., Wang, K. K. and Krishnadath, K. K. | 2018 | Smokeless Tobacco and Cigar and/or Pipe Are Risk Factors for Barrett Esophagus in Male Patients With Gastroesophageal Reflux Disease | Mayo Clin Proc | 10.1016/j.mayocp.2018.04.022 |
| **482** | Wienecke, A., Barnes, B., Neuhauser, H. and Kraywinkel, K. | 2015 | Incident cancers attributable to alcohol consumption in Germany, 2010 | Cancer Causes Control | 10.1007/s10552-015-0566-8 |
| **483** | Wong, C. M., Tsang, H., Lai, H. K., Thach, T. Q., Thomas, G. N., Chan, K. P., Lee, S. Y., Ayres, J. G., Lam, T. H. and Leung, W. K. | 2016 | STROBE-Long-Term Exposure to Ambient Fine Particulate Air Pollution and Hospitalization Due to Peptic Ulcers | Medicine (Baltimore) | 10.1097/md.0000000000003543 |
| **484** | Wu, G., Wu, Q., Xu, J., Gao, G., Chen, T. and Chen, G. | 2024 | Mortality burden and future projections of major risk factors for esophageal cancer in China from 1990 to 2019 | Gen Thorac Cardiovasc Surg | 10.1007/s11748-023-01987-8 |
| **485** | Wu, H. and Chen, H. L. | 2021 | The Association Between Heavy Alcohol Use and Gastric Cancer | Am J Gastroenterol | 10.14309/ajg.0000000000001325 |
| **486** | Wu, M., Lu, J., Yang, Z., Wei, F., Shen, P., Yu, Z., Tang, M., Jin, M., Lin, H., Chen, K. and Wang, J. | 2021 | Ambient air pollution and hospital visits for peptic ulcer disease in China: A three-year analysis | Environ Res | 10.1016/j.envres.2020.110347 |
| **487** | Wu, S., Zhang, L., Deng, J., Guo, B., Li, F., Wang, Y., Wu, R., Zhang, S., Lu, J. and Zhou, Y. | 2020 | A Novel Micropeptide Encoded by Y-Linked LINC00278 Links Cigarette Smoking and AR Signaling in Male Esophageal Squamous Cell Carcinoma | Cancer Res | 10.1158/0008-5472.Can-19-3440 |
| **488** | Wu, W., Li, L., Qu, C., Wang, M., Liang, S., Gao, X., Bao, X., Wang, L., Liu, H., Han, H., Xu, B., Zhou, Y., Li, B., Zhang, Y., Wang, G. and Zhong, C. | 2019 | Reflux finding score is associated with gastroesophageal flap valve status in patients with laryngopharyngeal reflux disease: a retrospective study | Sci Rep | 10.1038/s41598-019-52349-5 |
| **489** | Wu, X. C., Zheng, Y. F., Tang, M., Li, X. F., Zeng, R. and Zhang, J. R. | 2015 | Association Between Smoking and p53 Mutation in Oesophageal Squamous Cell Carcinoma: A Meta-analysis | Clin Oncol (R Coll Radiol) | 10.1016/j.clon.2015.02.007 |
| **490** | Xi, S., Inchauste, S., Guo, H., Shan, J., Xiao, Z., Xu, H., Miettenen, M., Zhang, M. R., Hong, J. A., Raiji, M. T., Altorki, N. K., Casson, A. G., Beer, D. G., Robles, A. I., Bowman, E. D., Harris, C. C., Steinberg, S. M. and Schrump, D. S. | 2015 | Cigarette smoke mediates epigenetic repression of miR-217 during esophageal adenocarcinogenesis | Oncogene | 10.1038/onc.2015.10 |
| **491** | Xie, F., Wang, D., Huang, Z. and Guo, Y. | 2014 | Coffee consumption and risk of gastric cancer: a large updated meta-analysis of prospective studies | Nutrients | 10.3390/nu6093734 |
| **492** | Xie, Y., Huang, S. and Su, Y. | 2016 | Dietary Flavonols Intake and Risk of Esophageal and Gastric Cancer: A Meta-Analysis of Epidemiological Studies | Nutrients | 10.3390/nu8020091 |
| **493** | Xiong, G. L., Atkin, A., Moquin, K., Candido, M., Beilenson, P., Kasirye, O., Wasserman, M., Blum, P. and Hilty, D. | 2020 | COVID-19 Transmission in a Psychiatric Long-Term Care Rehabilitation Facility: An Observational Study | Prim Care Companion CNS Disord | 10.4088/PCC.20m02765 |
| **494** | Xu, X., Mao, B., Wu, L., Liu, L., Rui, J. and Chen, G. | 2017 | A118G Polymorphism in μ-Opioid Receptor Gene and Interactions with Smoking and Drinking on Risk of Oesophageal Squamous Cell Carcinoma | J Clin Lab Anal | 10.1002/jcla.22018 |
| **495** | Xu, Y., Wang, J., He, Z., Rao, Z., Zhang, Z., Zhou, J., Zhou, T. and Wang, H. | 2024 | A review on the effect of COX-2-mediated mechanisms on development and progression of gastric cancer induced by nicotine | Biochem Pharmacol | 10.1016/j.bcp.2023.115980 |
| **496** | Yang, H., Lin, Z., Lin, Y., He, F., Liu, S., Zhang, Z., Wang, J., Xie, Q., Chen, Y., Chen, W. and Hu, Z. | 2020 | Risk Factors of Esophageal Squamous Cell Cancer Specific for Different Macroscopic Types | Nutr Cancer | 10.1080/01635581.2020.1733623 |
| **497** | Yang, H. Y., Huang, S. H., Shie, R. H. and Chen, P. C. | 2016 | Cancer mortality in a population exposed to nephrite processing | Occup Environ Med | 10.1136/oemed-2016-103586 |
| **498** | Yang, J., Wu, H., Wei, S., Xiong, H., Fu, X., Qi, Z., Jiang, Q., Li, W., Hu, G., Yuan, X. and Liao, Z. | 2014 | HPV seropositivity joints with susceptibility loci identified in GWASs at apoptosis associated genes to increase the risk of Esophageal Squamous Cell Carcinoma (ESCC) | BMC Cancer | 10.1186/1471-2407-14-501 |
| **499** | Yang, S., Lin, S., Li, N., Deng, Y., Wang, M., Xiang, D., Xiang, G., Wang, S., Ye, X., Zheng, Y., Yao, J., Zhai, Z., Wu, Y., Hu, J., Kang, H. and Dai, Z. | 2020 | Burden, trends, and risk factors of esophageal cancer in China from 1990 to 2017: an up-to-date overview and comparison with those in Japan and South Korea | J Hematol Oncol | 10.1186/s13045-020-00981-4 |
| **500** | Yang, X., Chen, X., Zhuang, M., Yuan, Z., Nie, S., Lu, M., Jin, L. and Ye, W. | 2017 | Smoking and alcohol drinking in relation to the risk of esophageal squamous cell carcinoma: A population-based case-control study in China | Sci Rep | 10.1038/s41598-017-17617-2 |
| **501** | Yao, B., Guan, S., Huang, X., Su, P., Song, Q. and Cheng, Y. | 2015 | A collision tumor of esophagus | Int J Clin Exp Pathol |  |
| **502** | Yao, W., Qin, X., Qi, B., Lu, J., Guo, L., Liu, F., Liu, S. and Zhao, B. | 2014 | Association of p53 expression with prognosis in patients with esophageal squamous cell carcinoma | Int J Clin Exp Pathol |  |
| **503** | Yaqoob, Z., Al-Kindi, S. G. and Zein, J. | 2016 | Association Between Celiac Disease and Asthma | Dig Dis Sci | 10.1007/s10620-016-4321-0 |
| **504** | Yates, M., Cheong, E., Luben, R., Igali, L., Fitzgerald, R., Khaw, K. T. and Hart, A. | 2014 | Body mass index, smoking, and alcohol and risks of Barrett's esophagus and esophageal adenocarcinoma: a UK prospective cohort study | Dig Dis Sci | 10.1007/s10620-013-3024-z |
| **505** | Yeh, J. M., Hur, C., Ward, Z., Schrag, D. and Goldie, S. J. | 2016 | Gastric adenocarcinoma screening and prevention in the era of new biomarker and endoscopic technologies: a cost-effectiveness analysis | Gut | 10.1136/gutjnl-2014-308588 |
| **506** | Yen, Y. C., Chang, J. H., Lin, W. C., Chiou, J. F., Chang, Y. C., Chang, C. L., Hsu, H. L., Chow, J. M., Yuan, K. S., Wu, A. T. H. and Wu, S. Y. | 2017 | Effectiveness of esophagectomy in patients with thoracic esophageal squamous cell carcinoma receiving definitive radiotherapy or concurrent chemoradiotherapy through intensity-modulated radiation therapy techniques | Cancer | 10.1002/cncr.30565 |
| **507** | Yim, M. H., Kim, K. H. and Lee, B. J. | 2021 | The number of household members as a risk factor for peptic ulcer disease | Sci Rep | 10.1038/s41598-021-84892-5 |
| **508** | Yin, J., Wang, X., Wei, J., Wang, L., Shi, Y., Zheng, L., Tang, W., Ding, G., Liu, C., Liu, R., Chen, S., Xu, Z. and Gu, H. | 2015 | Interleukin 12B rs3212227 T > G polymorphism was associated with an increased risk of gastric cardiac adenocarcinoma in a Chinese population | Dis Esophagus | 10.1111/dote.12189 |
| **509** | Yokota, T., Serizawa, M., Hosokawa, A., Kusafuka, K., Mori, K., Sugiyama, T., Tsubosa, Y. and Koh, Y. | 2018 | PIK3CA mutation is a favorable prognostic factor in esophageal cancer: molecular profile by next-generation sequencing using surgically resected formalin-fixed, paraffin-embedded tissue | BMC Cancer | 10.1186/s12885-018-4733-7 |
| **510** | Yokoyama, A., Kakiuchi, N., Yoshizato, T., Nannya, Y., Suzuki, H., Takeuchi, Y., Shiozawa, Y., Sato, Y., Aoki, K., Kim, S. K., Fujii, Y., Yoshida, K., Kataoka, K., Nakagawa, M. M., Inoue, Y., Hirano, T., Shiraishi, Y., Chiba, K., Tanaka, H., Sanada, M., Nishikawa, Y., Amanuma, Y., Ohashi, S., Aoyama, I., Horimatsu, T., Miyamoto, S., Tsunoda, S., Sakai, Y., Narahara, M., Brown, J. B., Sato, Y., Sawada, G., Mimori, K., Minamiguchi, S., Haga, H., Seno, H., Miyano, S., Makishima, H., Muto, M. and Ogawa, S. | 2019 | Age-related remodelling of oesophageal epithelia by mutated cancer drivers | Nature | 10.1038/s41586-018-0811-x |
| **511** | Yoshida, N., Baba, Y., Hiyoshi, Y., Shigaki, H., Kurashige, J., Sakamoto, Y., Miyamoto, Y., Iwatsuki, M., Ishimoto, T., Kosumi, K., Sugihara, H., Harada, K., Tokunaga, R., Izumi, D., Watanabe, M. and Baba, H. | 2016 | Duration of Smoking Cessation and Postoperative Morbidity After Esophagectomy for Esophageal Cancer: How Long Should Patients Stop Smoking Before Surgery? | World J Surg | 10.1007/s00268-015-3236-9 |
| **512** | Yoshida, N., Baba, Y., Kuroda, D., Miyamoto, Y., Iwatsuki, M., Hiyoshi, Y., Ishimoto, T., Sawayama, H., Imamura, Y., Watanabe, M. and Baba, H. | 2018 | Clinical utility of exhaled carbon monoxide in assessing preoperative smoking status and risks of postoperative morbidity after esophagectomy | Dis Esophagus | 10.1093/dote/doy024 |
| **513** | Yoshida, N., Nakamura, K., Kuroda, D., Baba, Y., Miyamoto, Y., Iwatsuki, M., Hiyoshi, Y., Ishimoto, T., Imamura, Y., Watanabe, M. and Baba, H. | 2018 | Preoperative Smoking Cessation is Integral to the Prevention of Postoperative Morbidities in Minimally Invasive Esophagectomy | World J Surg | 10.1007/s00268-018-4572-3 |
| **514** | Yu, C., Tang, H., Guo, Y., Bian, Z., Yang, L., Chen, Y., Tang, A., Zhou, X., Yang, X., Chen, J., Chen, Z., Lv, J. and Li, L. | 2018 | Hot Tea Consumption and Its Interactions With Alcohol and Tobacco Use on the Risk for Esophageal Cancer: A Population-Based Cohort Study | Ann Intern Med | 10.7326/m17-2000 |
| **515** | Yu, J., Deng, Y. and Chen, J. P. | 2014 | N-acetyltransferase 2 status and gastric cancer risk: a meta-analysis | Tumour Biol | 10.1007/s13277-014-1847-7 |
| **516** | Yu, J., Yang, P., Qin, X., Li, C., Lv, Y. and Wang, X. | 2022 | Impact of smoking on the eradication of Helicobacter pylori | Helicobacter | 10.1111/hel.12860 |
| **517** | Yu, Z., Mao, X., Tang, M., Chen, Y., Wu, M., Jin, M., Wang, J., Xu, L., Ye, G., Ding, J., Ye, B., Chen, D., Chen, Y., Chen, X., Sheng, X., Li, H., Chen, Q., Teng, X., Ding, Q., Yang, H., Shen, Z., Chen, K. and Yu, C. | 2021 | Association between past exposure to fine particulate matter (PM(2.5)) and peptic ulcer: A cross-sectional study in eastern China | Chemosphere | 10.1016/j.chemosphere.2020.128706 |
| **518** | Yuan, S., Chen, J., Ruan, X., Sun, Y., Zhang, K., Wang, X., Li, X., Gill, D., Burgess, S., Giovannucci, E. and Larsson, S. C. | 2023 | Smoking, alcohol consumption, and 24 gastrointestinal diseases: Mendelian randomization analysis | Elife | 10.7554/eLife.84051 |
| **519** | Zacharakis, G., Almasoud, A., Arahmane, O., Alzahrani, J. and Al-Ghamdi, S. | 2023 | Epidemiology, Risk Factors for Gastric Cancer and Surveillance of Premalignant Gastric Lesions: A Prospective Cohort Study of Central Saudi Arabia | Curr Oncol | 10.3390/curroncol30090605 |
| **520** | Zakko, L., Lutzke, L. and Wang, K. K. | 2017 | Screening for Barrett's esophagus | Minerva Med | 10.23736/s0026-4806.16.04864-3 |
| **521** | Zamora-Ros, R., Luján-Barroso, L., Bueno-de-Mesquita, H. B., Dik, V. K., Boeing, H., Steffen, A., Tjønneland, A., Olsen, A., Bech, B. H., Overvad, K., Boutron-Ruault, M. C., Racine, A., Fagherazzi, G., Kuhn, T., Katzke, V., Trichopoulou, A., Lagiou, P., Trichopoulos, D., Tumino, R., Panico, S., Vineis, P., Grioni, S., Palli, D., Weiderpass, E., Skeie, G., Huerta, J. M., Sánchez, M. J., Argüelles, M., Amiano, P., Ardanaz, E., Nilsson, L., Wallner, B., Lindkvist, B., Wallström, P., Peeters, P. H., Key, T. J., Khaw, K. T., Wareham, N. J., Freisling, H., Stepien, M., Ferrari, P., Gunter, M. J., Murphy, N., Riboli, E. and González, C. A. | 2014 | Tea and coffee consumption and risk of esophageal cancer: the European prospective investigation into cancer and nutrition study | Int J Cancer | 10.1002/ijc.28789 |
| **522** | Zhang, C., Ding, Z., Lv, G., Li, J., Zhang, J. F. and Zhou, P. | 2015 | CD226 rs727088A>G polymorphism increases the susceptibility to gastric cancer in Chinese populations | Gene | 10.1016/j.gene.2014.12.022 |
| **523** | Zhang, H., Liang, H., Gao, Y., Shang, X., Gong, L., Ma, Z., Sun, K., Tang, P. and Yu, Z. | 2016 | Metastatic lymph node ratio demonstrates better prognostic stratification than pN staging in patients with esophageal squamous cell carcinoma after esophagectomy | Sci Rep | 10.1038/srep38804 |
| **524** | Zhang, J., Huang, X., Xiao, J., Yang, Y., Zhou, Y., Wang, X., Liu, Q., Yang, J., Wang, M., Qiu, L., Zheng, Y., Zhang, P., Li, J., Wang, Y., Wei, Q., Jin, L., Wang, J. and Wang, M. | 2014 | Pri-miR-124 rs531564 and pri-miR-34b/c rs4938723 polymorphisms are associated with decreased risk of esophageal squamous cell carcinoma in Chinese populations | PLoS One | 10.1371/journal.pone.0100055 |
| **525** | Zhang, J., Ju, H., Gao, J. R., Jiao, X. L. and Lu, Y. | 2017 | Polymorphisms in human telomerase reverse transcriptase (hTERT) gene, gene- gene and gene-smoking interaction with susceptibility to gastric cancer in Chinese Han population | Oncotarget | 10.18632/oncotarget.15664 |
| **526** | Zhang, L., Jiang, Y., Wu, Q., Li, Q., Chen, D., Xu, L., Zhang, C., Zhang, M. and Ye, L. | 2014 | Gene-environment interactions on the risk of esophageal cancer among Asian populations with the G48A polymorphism in the alcohol dehydrogenase-2 gene: a meta-analysis | Tumour Biol | 10.1007/s13277-014-1616-7 |
| **527** | Zhang, W. B., Gu, H. Y., Shi, Y. J., Shao, A. Z., Chen, S. C., Yin, J. and Jiang, P. C. | 2015 | RANK rs1805034 T>C Polymorphism Is Associated with Susceptibility to Gastric Cardia Adenocarcinoma in a Chinese Population | Oncol Res Treat | 10.1159/000440855 |
| **528** | Zhang, X., Qin, Y., Pan, Z., Li, M., Liu, X., Chen, X., Qu, G., Zhou, L., Xu, M., Zheng, Q. and Li, D. | 2019 | Cannabidiol Induces Cell Cycle Arrest and Cell Apoptosis in Human Gastric Cancer SGC-7901 Cells | Biomolecules | 10.3390/biom9080302 |
| **529** | Zhang, Y. and Tong, T. | 2018 | Clinical Significance of O-6-Methylguanine-DNA-Methyltransferase Promoter Methylation in Patients with Esophageal Carcinoma: A Systematic Meta-Analysis | Dig Dis | 10.1159/000481342 |
| **530** | Zhao, J. K., Wu, M., Kim, C. H., Jin, Z. Y., Zhou, J. Y., Han, R. Q., Yang, J., Zhang, X. F., Wang, X. S., Liu, A. M., Gu, X., Su, M., Hu, X., Sun, Z., Li, G., Li, L., Mu, L. and Zhang, Z. F. | 2017 | Jiangsu Four Cancers Study: a large case-control study of lung, liver, stomach, and esophageal cancers in Jiangsu Province, China | Eur J Cancer Prev | 10.1097/cej.0000000000000262 |
| **531** | Zhao, L. L., Huang, H., Wang, Y., Wang, T. B., Zhou, H., Ma, F. H., Ren, H., Niu, P. H., Zhao, D. B. and Chen, Y. T. | 2020 | Lifestyle factors and long-term survival of gastric cancer patients: A large bidirectional cohort study from China | World J Gastroenterol | 10.3748/wjg.v26.i14.1613 |
| **532** | Zhao, X. and Lim, F. | 2020 | Lifestyle Risk Factors in Esophageal Cancer: An Integrative Review | Crit Care Nurs Q | 10.1097/cnq.0000000000000295 |
| **533** | Zhao, Z., Yin, Z. and Zhang, C. | 2021 | Lifestyle interventions can reduce the risk of Barrett's esophagus: a systematic review and meta-analysis of 62 studies involving 250,157 participants | Cancer Med | 10.1002/cam4.4061 |
| **534** | Zheng, L. M., Zhang, Z. W., Wang, W., Li, Y. and Wen, F. | 2022 | Relationship between smoking and postoperative complications of cervical spine surgery: a systematic review and meta-analysis | Sci Rep | 10.1038/s41598-022-13198-x |
| **535** | Zheng, T., BouSaba, J., Taylor, A., Dilmaghani, S., Busciglio, I., Carlson, P., Torres, M., Ryks, M., Burton, D., Harmsen, W. S. and Camilleri, M. | 2023 | A Randomized, Controlled Trial of Efficacy and Safety of Cannabidiol in Idiopathic and Diabetic Gastroparesis | Clin Gastroenterol Hepatol | 10.1016/j.cgh.2023.07.008 |
| **536** | Zheng, Y., Cao, X., Wen, J., Yang, H., Luo, K., Liu, Q., Huang, Q., Chen, J. and Fu, J. | 2015 | Smoking affects treatment outcome in patients with resected esophageal squamous cell carcinoma who received chemotherapy | PLoS One | 10.1371/journal.pone.0123246 |
| **537** | Zhou, H., Sun, H., Liu, X., Chen, J., Zhang, L., Lin, S., Han, X., Nie, C., Liu, Y., Tian, W. and Zhao, Y. | 2019 | Combined effect between WT1 methylation and Helicobacter pylori infection, smoking, and alcohol consumption on the risk of gastric cancer | Helicobacter | 10.1111/hel.12650 |
| **538** | Zhou, R., Li, Y., Wang, N., Niu, C., Huang, X., Cao, S. and Huo, X. | 2021 | PARP1 rs1136410 C/C genotype associated with an increased risk of esophageal cancer in smokers | Mol Biol Rep | 10.1007/s11033-021-06169-4 |
| **539** | Zhou, Y., Yang, S., Lin, Q., He, Q. and Cui, Y. | 2023 | Frequent presence of major dust mite allergens in human digestive tissues of children with gastritis | Allergy | 10.1111/all.15587 |
| **540** | Zhu, J. F., Feng, X. Y., Zhang, X. W., Wen, Y. S., Lin, P., Cai, L. and Zhang, L. J. | 2015 | Time distribution of recurrence risk of oesophageal squamous cell carcinoma with complete resection (R0) in a Chinese population | Eur J Cardiothorac Surg | 10.1093/ejcts/ezv147 |
| **541** | Zhu, M. L., He, J., Wang, M., Sun, M. H., Jin, L., Wang, X., Yang, Y. J., Wang, J. C., Zheng, L., Xiang, J. Q. and Wei, Q. Y. | 2014 | Potentially functional polymorphisms in the ERCC2 gene and risk of esophageal squamous cell carcinoma in Chinese populations | Sci Rep | 10.1038/srep06281 |
| **542** | Zwink, N., Choinitzki, V., Baudisch, F., Hölscher, A., Boemers, T. M., Turial, S., Kurz, R., Heydweiller, A., Keppler, K., Müller, A., Bagci, S., Pauly, M., Brokmeier, U., Leutner, A., Degenhardt, P., Schmiedeke, E., Märzheuser, S., Grasshoff-Derr, S., Holland-Cunz, S., Palta, M., Schäfer, M., Ure, B. M., Lacher, M., Nöthen, M. M., Schumacher, J., Jenetzky, E. and Reutter, H. | 2016 | Comparison of environmental risk factors for esophageal atresia, anorectal malformations, and the combined phenotype in 263 German families | Dis Esophagus | 10.1111/dote.12431 |
|  | **Web of Science (N = 934)** | | | | |
| **1** | A. F. Abbas, A. G. M. Al-Saadi and S. A. Fazaa | 2017 | Investigation of IgG and IgM seroprevalence of <i>Helicobacter pylori</i> infections and their relation toIL-6 and some risk factors among dyspeptic patients in Al-Qasim city of Babylon province | Research Journal of Pharmaceutical Biological and Chemical Sciences |  |
| **2** | A. I. A. Abd Alrheam, M. M. M. Makhlouf, H. F. Gomaa and A. I. Abd Elneam | 2018 | Biochemical and Histological Studies on the Effect of Nicotine on the Mucosa of Albino Rat Stomach | Research Journal of Pharmaceutical Biological and Chemical Sciences |  |
| **3** | M. M. Abd El-Mawgod, N. A. H. Alanazi, M. S. F. Alenezi, M. A. M. Almesned and A. F. K. Alenezi | 2022 | Awareness of esophageal cancer among the adult population in Arar city, Saudi Arabia | Medical Science | 10.54905/disssi/v26i129/ms475e2555 |
| **4** | A. H. M. Abdelraheem, A. Z. A. Alharthi, F. B. H. Alziyadi, A. M. A. Sharahili, A. S. M. Alsgoor, M. Alshehri, A. A. M. Alyala and A. M. Albusaamara | 2019 | PEPTIC ULCER DISEASE AMONG ADULT MALE AND FEMALE PATIENTS AT KING KHALID HOSPITAL FROM 4-7/1434 | Indo American Journal of Pharmaceutical Sciences | 10.5281/zenodo.2556195 |
| **5** | O. Abdihamid, H. Abdourahman, A. Ibrahim, T. Kareu, A. Hadi, A. Omar and M. Mutebi | 2024 | Landscape of esophageal cancer in Northern Kenya: experience from Garissa Regional Cancer Center | Ecancermedicalscience | 10.3332/ecancer.2024.1694 |
| **6** | V. B. Abhilash, M. K. Behera, S. C. U. Patne, S. K. Shukla and V. K. Dixit | 2023 | Clinicopathological Significance and Prognostic Role of Her2neu Protein Expression in Patients with Carcinoma Stomach: A Prospective Study from Northern India | South Asian Journal of Cancer | 10.1055/s-0042-1759601 |
| **7** | A. C. Adejumo, J. J. Li, O. Akanbi, K. L. Adejumo and T. N. Bukong | 2019 | Reduced Prevalence of Alcoholic Gastritis in Hospitalized Individuals Who Consume Cannabis | Alcoholism-Clinical and Experimental Research | 10.1111/acer.13930 |
| **8** | K. K. Adkison, J. J. Gan, L. Elko-Simms, S. Gardner, E. Dumont, L. S. Jones, J. Saunders, T. Marbury, W. Smith, J. Berg, C. Galloway and P. J. Stump | 2015 | Pharmacokinetics of hepatitis C virus NS5A inhibitor JNJ-56914845 (GSK2336805) in subjects with hepatic impairment | Journal of Clinical Pharmacology | 10.1002/jcph.512 |
| **9** | A. Afzal, M. A. Qayyum and M. H. Shah | 2021 | Comparative Assessment of Trace Elements in the Blood of Gastric Cancer Patients and Healthy Subjects | Biointerface Research in Applied Chemistry | 10.33263/briac113.1082410843 |
| **10** | I. Agalliu, Z. G. Chen, T. Wang, R. B. Hayes, N. D. Freedman, S. M. Gapstur and R. D. Burk | 2018 | Oral Alpha, Beta, and Gamma HPV Types and Risk of Incident Esophageal Cancer | Cancer Epidemiology Biomarkers & Prevention | 10.1158/1055-9965.Epi-18-0287 |
| **11** | B. Ahmadi, M. Alimohammadian, M. Yaseri, A. Majidi, M. Boreiri, F. Islami, H. Poustchi, M. H. Derakhshan, A. Feizesani, A. Pourshams, C. C. Abnet, P. Brennan, S. M. Dawsey, F. Kamangar, P. Boffetta, A. Sadjadi and R. Malekzadeh | 2016 | Multimorbidity: Epidemiology and Risk Factors in the Golestan Cohort Study, Iran: A Cross-Sectional Analysis | Medicine | 10.1097/md.0000000000002756 |
| **12** | S. Ahmed, S. Jamil, H. Shaikh and M. Abbasi | 2020 | Effects of Life style factors on the symptoms of gastro esophageal reflux disease: A cross sectional study in a Pakistani population | Pakistan Journal of Medical Sciences | 10.12669/pjms.36.2.1371 |
| **13** | S. Akiyama, H. Saeki, Y. Nakashima, M. Iimori, H. Kitao, E. Oki, Y. Oda, Y. Nakabeppu, Y. Kakeji and Y. Maehara | 2017 | Prognostic impact of MutT homolog-1 expression on esophageal squamous cell carcinoma | Cancer Medicine | 10.1002/cam4.979 |
| **14** | M. F. Akl, M. A. Ibrahem, A. Khater, E. El-zahaf, K. Farag and H. Abdallah | 2018 | Etiologic and Clinicopathological Correlates of Gastric Carcinoma in the Egyptian Delta | Indian Journal of Surgical Oncology | 10.1007/s13193-018-0754-6 |
| **15** | F. Al Gharaibeh and L. Gibson | 2022 | The impact of COVID-19 quarantine measures on the mental health of families | Journal of Social Work | 10.1177/14680173211011705 |
| **16** | B. M. R. Al-Aajem and N. H. Majeed | 2019 | Molecular Study of CagA gene in <i>Helicobacter pylori</i> Isolated from Gastritis | Pakistan Journal of Medical & Health Sciences |  |
| **17** | M. Al-Azri, J. Al-Kindi, T. Al-Harthi, M. Al-Dahri, S. M. Panchatcharam and A. Al-Maniri | 2019 | Awareness of Stomach and Colorectal Cancer Risk Factors, Symptoms and Time Taken to Seek Medical Help Among Public Attending Primary Care Setting in Muscat Governorate, Oman | Journal of Cancer Education | 10.1007/s13187-017-1266-8 |
| **18** | M. A. Al-Ghamdi, M. A. Murad, R. M. Alshiakh, H. J. Abousada, M. B. Alharbi, R. A. Almehdhar, T. S. Aljuhani, A. M. H. Alsobyei, A. H. Alharbi, G. A. Al Ghanem, S. A. Alharbi, A. S. Alonezi, R. S. A. Alghamdi, R. T. Almowllad and F. M. Alfaqih | 2022 | Prevalence and Complications of Inflammatory Bowel Disease among Saudi Population: A Cross-Sectional Study | Journal of Research in Medical and Dental Science |  |
| **19** | A. Al-Kaabi, N. S. Baranov, R. S. van der Post, E. J. Schoon, C. Rosman, H. W. M. van Laarhoven, M. Verheij, R. H. A. Verhoeven and P. D. Siersema | 2022 | Age-specific incidence, treatment, and survival trends in esophageal cancer: a Dutch population-based cohort study | Acta Oncologica | 10.1080/0284186x.2021.2024878 |
| **20** | R. T. Al-Kasasbeh, N. Korenevskiy, M. S. Alshamasin, F. Ionescu, E. Boitcova and E. Ai-Kasasbeh | 2019 | Fuzzy prediction and early detection of stomach diseases by means of combined iteration fuzzy models | International Journal of Biomedical Engineering and Technology |  |
| **21** | J. Al-Kassmy, M. Alsalmi, W. J. Kang and P. Huot | 2024 | Anticonvulsant Agents for Treatment of Restless Legs Syndrome | Neurologist | 10.1097/nrl.0000000000000552 |
| **22** | S. A. Al-Towairqi, W. J. Alharthi, A. S. Almalki, M. M. Dabi and R. A. Althobaiti | 2020 | Prevalence and risk factors of gastroesophageal reflux disease among female Medical students at Taif University, Saudi Arabia | World Family Medicine | 10.5742/mewfm.2020.93912 |
| **23** | K. Alcala, H. Poustchi, V. Viallon, F. Islami, A. Pourshams, A. Sadjadi, S. Nemati, M. Khoshnia, A. Gharavi, G. Roshandel, M. Hashemian, S. M. Dawsey, C. C. Abnet, P. Brennan, P. Boffetta, K. Zendehdel, F. Kamangar, R. Malekzadeh and M. Sheikh | 2023 | Incident cancers attributable to using opium and smoking cigarettes in the Golestan cohort study | Eclinicalmedicine | 10.1016/j.eclinm.2023.102229 |
| **24** | A. Alcaraz, J. Caporale, A. Bardach, F. Augustovski and A. Pichon-Riviere | 2016 | Burden of disease attributable to tobacco use in Argentina and potential impact of price increases through taxes | Revista Panamericana De Salud Publica-Pan American Journal of Public Health |  |
| **25** | A. G. Alghamdi, A. M. Alshareef, A. T. Alzahrani, Z. S. Alharthi, S. S. Alghamdi, A. M. Alghamdi, F. A. Alzahrani and R. A. Alzahrani | 2023 | Knowledge and Awareness About Gastric Cancer Among the General Population in Al-Baha City, Saudi Arabia | Cureus Journal of Medical Science | 10.7759/cureus.39589 |
| **26** | M. M. Alhalabi, S. A. Alsayd and M. E. Albattah | 2022 | Advanced diffuse gastric adenocarcinoma in young Syrian woman. A case report | Annals of Medicine and Surgery | 10.1016/j.amsu.2022.103728 |
| **27** | A. B. Ali, N. A. Khan, D. T. Nguyen, R. Chihara, E. Y. Chan, E. A. Graviss, B. J. Dunkin and M. P. Kim | 2020 | Robotic and per-oral endoscopic myotomy have fewer technical complications compared to laparoscopic Heller myotomy | Surgical Endoscopy and Other Interventional Techniques | 10.1007/s00464-019-07093-2 |
| **28** | I. Ali, Q. Abdo, S. M. Al-Hihi and A. Shawabkeh | 2022 | Association between ulcerative colitis and <i>Helicobacter pylori</i> infection: A case-control study | Heliyon | 10.1016/j.heliyon.2022.e08930 |
| **29** | G. Alicandro, P. Bertuccio, G. Collatuzzo, C. Pelucchi, R. Bonzi, L. M. Liao, C. S. Rabkin, R. Sinha, E. Negri, M. Dalmartello, D. Zaridze, D. Maximovich, J. Vioque, M. G. de la Hera, S. Tsugane, A. Hidaka, G. S. Hamada, L. López-Carrillo, R. U. Hernández-Ramírez, R. Malekzadeh, F. Pourfarzi, Z. F. Zhang, R. C. Kurtz, M. C. Camargo, M. P. Curado, N. Lunet, P. Boffetta and C. La Vecchia | 2022 | The mediating role of combined lifestyle factors on the relationship between education and gastric cancer in the Stomach cancer Pooling (StoP) Project | British Journal of Cancer | 10.1038/s41416-022-01857-9 |
| **30** | M. Alimohammadian, A. Majidi, M. Yaseri, B. Ahmadi, F. Islami, M. Derakhshan, A. Delavari, M. Amani, A. Feyz-Sani, H. Poustchi, A. Pourshams, A. M. Sadjadi, M. Khoshnia, S. Qaravi, C. C. Abnet, S. Dawsey, P. Brennan, F. Kamangar, P. Boffetta, A. Sadjadi and R. Malekzadeh | 2017 | Multimorbidity as an important issue among women: results of a gender difference investigation in a large population-based cross-sectional study in West Asia | Bmj Open | 10.1136/bmjopen-2016-013548 |
| **31** | A. G. R. Alkushi and N. A. M. Elsawy | 2017 | Quercetin attenuates, indomethacin-induced acute gastric ulcer in rats | Folia Morphologica | 10.5603/FM.a2016.0067 |
| **32** | E. M. Allen, B. H. Alexander, R. F. MacLehose, H. H. Nelson, G. Ramachandran and J. H. Mandel | 2015 | Cancer incidence among Minnesota taconite mining industry workers | Annals of Epidemiology | 10.1016/j.annepidem.2015.08.003 |
| **33** | J. E. Allen, M. Desai, C. A. M. Roumans, S. Vennalaganti, P. Vennalaganti, A. Bansal, G. Falk, D. Lieberman, R. Sampliner, P. Thota, J. Vargo, N. Gupta, F. Moawad, M. Bruno, K. F. Kennedy, S. Gaddam, P. Young, S. Mathur, B. Cash, M. Spaander and P. Sharma | 2021 | Low Risk of Progression of Barrett's Esophagus to Neoplasia in Women | Journal of Clinical Gastroenterology | 10.1097/mcg.0000000000001362 |
| **34** | A. E. Almazar, J. D. Penfield, Y. A. Saito and N. J. Talley | 2021 | Survival Times of Patients With Menetrier's Disease and Risk of Gastric Cancer | Clinical Gastroenterology and Hepatology | 10.1016/j.cgh.2020.03.017 |
| **35** | M. K. Almutairi, A. K. Alkharji, W. A. Alhelal, S. A. Alqahtani and M. M. Altalha | 2019 | ETIOLOGY OF APPARENT LIFE-THREATENING EVENT IN INFANTS AT NATIONAL GUARD HEALTH AFFAIRS IN RIYADH, SAUDI ARABIA | Indo American Journal of Pharmaceutical Sciences | 10.5281/zenodo.2648626 |
| **36** | A. Alotaibi, V. P. Gadekar, P. S. Gundla, S. Mandarthi, N. Jayendra, A. Tungekar, B. V. Lavanya, A. K. Bhagavath, M. A. W. Cordero, J. Pitkaniemi, S. K. Niazi, R. Upadhya, A. Bepari and P. Hebbar | 2023 | Global comparative transcriptomes uncover novel and population-specific gene expression in esophageal squamous cell carcinoma | Infectious Agents and Cancer | 10.1186/s13027-023-00525-8 |
| **37** | A. A. Alrashed, K. I. Aljammaz, A. Pathan, A. A. Mandili, S. A. Almatrafi, M. H. Almotire and S. M. Bahkali | 2019 | Prevalence and risk factors of gastroesophageal reflux disease among Shaqra University students, Saudi Arabia | Journal of Family Medicine and Primary Care | 10.4103/jfmpc.jfmpc_443_18 |
| **38** | A. M. Alsaihati, B. E. Almasoud, S. T. Al Omran, A. H. A. Ali, A. S. Alsulaim, G. S. Almarzoqi, M. S. AlAbbad, B. I. AlKhalifah, M. F. Al Hemaid, M. S. M. Alhagbanim, M. N. A. Al Hajjar and M. A. Almakhayitah | 2018 | PUBLIC AWARENESS TOWARDS GERD AMONG SAUDI POPULATION IN AL-DAMMAM CITY, SAUDI ARABIA | Indo American Journal of Pharmaceutical Sciences | 10.5281/zenodo.1480868 |
| **39** | F. M. AlTassan, S. S. Al-Khowaiter, H. E. Alsubki, W. A. Alhamoud, A. K. Niazi and B. M. AlJarallah | 2020 | Prevalence of gastro-esophageal reflux in diabetic patients at a tertiary hospital in Central Saudi Arabia | Saudi Medical Journal | 10.15537/smj.2020.2.24844 |
| **40** | A. Amani, A. A. A. Kamrani, R. Fadayevatan, B. Eshrati and M. Rafiee | 2023 | Burden of Important Risk Factors for Common Cancers Among Older Adults in Markazi Province, Iran in 2016 | Salmand-Iranian Journal of Ageing | 10.32598/sija.2023.3517.1 |
| **41** | K. Amenu, B. Wieland, B. Szonyi and D. Grace | 2019 | Milk handling practices and consumption behavior among Borana pastoralists in southern Ethiopia | Journal of Health Population and Nutrition | 10.1186/s41043-019-0163-7 |
| **42** | F. H. Amin, M. Ghaemi, S. M. Mostafavi, L. Goshayeshi, K. Rezaei, M. Vahed and B. Kiani | 2021 | A Geospatial database of gastric cancer patients and associated potential risk factors including lifestyle and air pollution | Bmc Research Notes | 10.1186/s13104-021-05506-x |
| **43** | T. Anuk, S. Kahramanca and O. Kaya | 2018 | Predictive Parameters for Barrett's Esophagus: Percent Body Fat (PBF) and Visceral Fat Area (VFA) are more Valuable than Body Mass Index (BMI) | Kuwait Medical Journal |  |
| **44** | K. Arakawa, K. Hata, Y. Yamamoto, T. Nishikawa, T. Tanaka, T. Kiyomatsu, K. Kawai, H. Nozawa, M. Yoshida, H. Fukuhara, M. Fujishiro, T. Morikawa, T. Yamasoba, K. Koike, M. Fukayama and T. Watanabe | 2018 | Nine primary malignant neoplas-msinvolving the esophagus, stomach, colon, rectum, prostate, and external ear canal-without microsatellite instability: a case report | Bmc Cancer | 10.1186/s12885-017-3973-2 |
| **45** | B. L. A. Arias, J. P. D. Ríos and J. D. O. Olarte | 2023 | Association between gastroesophageal reflux and lifestyle: which non-pharmacological interventions improve the management of the disease? | Archivos De Medicina | 10.30554/archmed.23.2.4967.2023 |
| **46** | E. Armand, D. Boulate, A. Fourdrain, N. A. T. Nguyen, N. Resseguier, G. Brioude, D. Trousse, C. Doddoli, X. B. D'Journo and P. A. Thomas | 2022 | Benignant and malignant epidemiology among surgical resections for suspicious solitary lung cancer without preoperative tissue diagnosis | European Journal of Cardio-Thoracic Surgery | 10.1093/ejcts/ezac590 |
| **47** | Z. Arora, A. Garber and P. N. Thota | 2016 | Risk factors for Barrett's esophagus | Journal of Digestive Diseases | 10.1111/1751-2980.12332 |
| **48** | Q. Arroyo-Martínez, M. Rodríguez-Téllez, A. García-Escudero, J. Brugal-Medina, R. González-Cámpora and A. Caunedo-Alvarez | 2016 | Epidemiology of Barrett's esophagus and esophageal adenocarcinoma in Spain. A unicentric study | Revista Espanola De Enfermedades Digestivas | 10.17235/reed.2016.4229/2016 |
| **49** | I. Arshad, S. Zeb, S. H. Keerio, K. Almani, S. A. Raza and M. M. Naeem | 2021 | Risk Factors Associated with Oral Manifestations and Oral Health Impact of Gastro-Oesophageal Reflux Disease | Pakistan Journal of Medical & Health Sciences | 10.53350/pjmhs211582202 |
| **50** | A. Aryzbekova, K. T. Juszkiewicz, D. E. Burgess, A. Polski and E. Poleszak | 2015 | A brief analysis of patients suffering from stomach or duodenal ulcers in Almaty hospital <i>No</i> 1 | Current Issues in Pharmacy and Medical Sciences | 10.1515/cipms-2015-0079 |
| **51** | M. Asadollahi, O. Firuzi, F. H. Jamebozorgi, M. Alizadeh and A. R. Jassbi | 2019 | Ethnopharmacological studies, chemical composition, antibacterial and cytotoxic activities of essential oils of eleven <i>Salvia</i> in Iran | Journal of Herbal Medicine | 10.1016/j.hermed.2018.11.006 |
| **52** | S. Asghar, S. Asghar, S. Shahid, H. Sajjad, J. A. Nasir and M. Usman | 2023 | Gastroparesis-Related Symptoms in Patients With Type 2 Diabetes Mellitus: Early Detection, Risk Factors, and Prevalence | Cureus Journal of Medical Science | 10.7759/cureus.35787 |
| **53** | M. F. Ashraf, S. Richter, S. H. Arker and N. Parsa | 2021 | A Rare Case of Esophageal Leukoplakia: A Potential Precursor to Esophageal Malignancy | Cureus Journal of Medical Science | 10.7759/cureus.17205 |
| **54** | A. W. Asombang, V. Kayamba, M. M. Lisulo, K. Trinkaus, V. Mudenda, E. Sinkala, S. Mwanamakondo, T. Banda, R. Soko and P. Kelly | 2016 | Esophageal squamous cell cancer in a highly endemic region | World Journal of Gastroenterology | 10.3748/wjg.v22.i9.2811 |
| **55** | M. O. Avinçsal, H. Shinomiya, M. Teshima, M. Kubo, N. Otsuki, N. Kyota, R. Sasaki, Y. Zen and K. Nibu | 2018 | Impact of alcohol dehydrogenase-aldehyde dehydrogenase polymorphism on clinical outcome in patients with hypopharyngeal cancer | Head and Neck-Journal for the Sciences and Specialties of the Head and Neck | 10.1002/hed.25050 |
| **56** | A. Awaya and Y. Kuroiwa | 2020 | The Relationship between Annual Airborne Pollen Levels and Occurrence of All Cancers, and Lung, Stomach, Colorectal, Pancreatic and Breast Cancers: A Retrospective Study from the National Registry Database of Cancer Incidence in Japan, 1975-2015 | International Journal of Environmental Research and Public Health | 10.3390/ijerph17113950 |
| **57** | V. Babaei, Y. Saghaei, H. Z. Gohardani, F. Vali and S. Teimourian | 2017 | Effects of Different Environmental Factors and Virulence Factors, <i>dupA</i> and <i>iceA</i> Genes, of <i>Helicobacter pylori</i> on Peptic Ulcer | Jundishapur Journal of Microbiology | 10.5812/jjm.40161 |
| **58** | N. Babhadiashar, M. Sotoudeh, E. Azizi, J. Bashiri, R. Didevar, R. Malekzadeh and M. H. Ghahremani | 2014 | Correlation between Cigarette Smoking and Urine Cotinine Level in Gastric Cancer Patients | Iranian Journal of Pharmaceutical Research |  |
| **59** | B. Babic, R. R. Datta, W. Schröder, L. M. Schiffmann, T. Schmidt, C. J. Bruns and H. F. Fuchs | 2021 | Impact of COVID-19 on oncological surgery of the upper gastrointestinal tract | Chirurg | 10.1007/s00104-021-01489-4 |
| **60** | X. P. Bai, E. Ihara, Y. Otsuka, S. Tsuruta, K. Hirano, Y. Tanaka, H. Ogino, M. Hirano, T. Chinen, H. Akiho, K. Nakamura, Y. Oda and Y. Ogawa | 2019 | Involvement of different receptor subtypes in prostaglandin E2-induced contraction and relaxation in the lower esophageal sphincter and esophageal body | European Journal of Pharmacology | 10.1016/j.ejphar.2019.172405 |
| **61** | D. Baik, J. Sheng, K. Schlaffer, F. K. Friedenberg, M. S. Smith and A. C. Ehrlich | 2017 | Abdominal diameter index is a stronger predictor of prevalent Barrett's esophagus than BMI or waist-to-hip ratio | Diseases of the Esophagus | 10.1093/dote/dox056 |
| **62** | M. E. Bailey, L. F. Borges, H. J. Goldberg, K. E. Hathorn, S. Gavini, W. K. Lo and W. W. Chan | 2023 | Abnormal bolus reflux on impedance-pH testing independently predicts 3-year pulmonary outcome and mortality in pulmonary fibrosis | Journal of Gastroenterology and Hepatology | 10.1111/jgh.16325 |
| **63** | D. C. Baird, D. J. Harker and A. S. Karmes | 2015 | Diagnosis and Treatment of Gastroesophageal Reflux in Infants and Children | American Family Physician |  |
| **64** | O. Bakr, W. Zhao and D. Corley | 2018 | Gastroesophageal Reflux Frequency, Severity, Age of Onset, Family History and Acid Suppressive Therapy Predict Barrett Esophagus in a Large Population | Journal of Clinical Gastroenterology | 10.1097/mcg.0000000000000983 |
| **65** | S. Banerjee | 2020 | Risk Associated with NAD (P) H: Quinone Oxidoreductase 1 (NQO1) C609T Polymorphism for Cigarette Smoke Induced Cardiovascular Disease (CVD): A Study on Male Current Smokers from Eastern India | International Journal of Life Science and Pharma Research | 10.22376/ijpbs/lpr.2020.10.4.L29-35 |
| **66** | N. Baras, S. Dahm, J. Haberland, M. Janz, K. Emrich, K. Kraywinkel and A. Salama | 2017 | Subsequent malignancies among long-term survivors of Hodgkin lymphoma and non-Hodgkin lymphoma: a pooled analysis of German cancer registry data (1990-2012) | British Journal of Haematology | 10.1111/bjh.14530 |
| **67** | G. D. Batty, C. M. Calvin, C. E. Brett, I. Cukic and I. J. Deary | 2015 | Childhood Body Weight in Relation to Morbidity From Cardiovascular Disease and Cancer in Older Adulthood: 67-Year Follow-up of Participants in the 1947 Scottish Mental Survey | American Journal of Epidemiology | 10.1093/aje/kwv154 |
| **68** | C. Bazin, A. Benezech, M. Alessandrini, J. C. Grimaud and V. Vitton | 2018 | Esophageal Motor Disorders Are a Strong and Independant Associated Factor of Barrett's Esophagus | Journal of Neurogastroenterology and Motility | 10.5056/jnm17090 |
| **69** | E. Becskeházi, M. M. Korsós, E. Gál, L. Tiszlavicz, Z. Hoyk, M. A. Deli, Z. M. Köhler, A. Keller-Pintér, A. Horváth, K. Cseko, Z. Helyes, P. Hegyi and V. Venglovecz | 2021 | Inhibition of NHE-1 Increases Smoke-Induced Proliferative Activity of Barrett's Esophageal Cell Line | International Journal of Molecular Sciences | 10.3390/ijms221910581 |
| **70** | Y. Benakli, A. Khenchouche, S. Rabea, A. A. Mansour, M. M. Salem-Bekhit, E. I. Taha, M. M. Salem, S. Singh, K. O. Ouali, Y. Benguerba and K. Houali | 2023 | Characterizing EBV-associated Gastric Carcinoma (EBVaGC): A deep dive into LMP1 expression patterns | Cellular and Molecular Biology | 10.14715/cmb/2023.69.13.33 |
| **71** | J. Berro, M. Akel, S. Hallit and S. Obeid | 2021 | Relationships between inappropriate eating habits and problematic alcohol use, cigarette and waterpipe dependence among male adolescents in Lebanon | Bmc Public Health | 10.1186/s12889-021-10184-2 |
| **72** | P. Bertuccio, G. Alicandro, M. Rota, C. Pelucchi, R. Bonzi, C. Galeone, F. Bravi, K. C. Johnson, J. Hu, D. Palli, M. Ferraroni, L. López-Carrillo, N. Lunet, A. Ferro, R. Malekzadeh, D. Zaridze, D. Maximovitch, J. Vioque, E. M. Navarrete-Munoz, M. Pakseresht, R. U. Hernández-Ramírez, M. López-Cervantes, M. Ward, F. Pourfarzi, S. Tsugane, A. Hidaka, Z. F. Zhang, R. C. Kurtz, P. Lagiou, A. Lagiou, P. Boffetta, S. Boccia, E. Negri and C. La Vecchia | 2019 | Citrus fruit intake and gastric cancer: The stomach cancer pooling (StoP) project consortium | International Journal of Cancer | 10.1002/ijc.32046 |
| **73** | P. P. Bessonov and N. G. Bessonova | 2019 | CONCOMITANT DISEASES AND RISK FACTORS OF GASTRODUODENAL EROSION IN THE CONDITIONS OF YAKUTIA | Yakut Medical Journal | 10.25789/ymj.2019.68.20 |
| **74** | D. Bhandari, Y. Y. Zhu, C. Zhang, W. Z. Zhu, A. Alexandridis, A. Etemadi, N. D. Freedman, C. Y. Chang, C. C. Abnet, S. M. Dawsey, M. Inoue-Choi, H. Poustchi, A. Pourshams, P. Boffetta, R. Malekzadeh and B. Blount | 2023 | Smoke exposure associated with higher urinary benzene biomarker muconic acid (MUCA) in Golestan Cohort Study participants | Biomarkers | 10.1080/1354750x.2023.2276030 |
| **75** | J. H. Bi, H. Y. Yuan, Y. Jiang, Y. Zhang, W. W. Zheng, L. Zhang, Z. Y. Li, H. L. Li, Y. T. Tan, W. S. Zhao and Y. B. Xiang | 2022 | Incidence, Mortality Features and Lifetime Risk Estimation of Digestive Tract Cancers in an Urban District of Shanghai, China | Journal of Epidemiology and Global Health | 10.1007/s44197-022-00047-3 |
| **76** | Y. H. Bi, J. J. Pei, C. F. Hao, W. Yao and H. X. Wang | 2021 | The relationship between chronic diseases and depression in middle-aged and older adults: A 4-year follow-up study from the China Health and Retirement Longitudinal Study | Journal of Affective Disorders | 10.1016/j.jad.2021.04.032 |
| **77** | Z. Bilgi and S. J. Swanson | 2019 | Current indications and outcomes for thoracoscopic segmentectomy for early stage lung cancer | Journal of Thoracic Disease | 10.21037/jtd.2019.07.06 |
| **78** | C. G. Birngruber, F. Veit, J. Lang and M. A. Verhoff | 2017 | Inhaled cyanide poisoning as a vital sign in a room fire victim | Forensic Science International | 10.1016/j.forsciint.2017.10.037 |
| **79** | P. J. C. Biselli, J. P. Kirkness, L. Grote, K. Fricke, A. R. Schwartz, P. Smith and H. Schneider | 2017 | Nasal high-flow therapy reduces work of breathing compared with oxygen during sleep in COPD and smoking controls: a prospective observational study | Journal of Applied Physiology | 10.1152/japplphysiol.00279.2016 |
| **80** | F. Böhme, K. Racz, C. Sebesta and C. Sebesta | 2023 | Esophageal Cancer | Wiener Medizinische Wochenschrift | 10.1007/s10354-022-00972-9 |
| **81** | J. D. Boice, B. Quinn, I. Al-Nabulsi, A. Ansari, P. K. Blake, S. R. Blattnig, E. A. Caffrey, S. S. Cohen, A. P. Golden, K. D. Held, D. W. Jokisch, R. W. Leggett, M. T. Mumma, C. Samuels, J. E. Till, S. Y. Tolmachev, R. C. Yoder, J. Y. Zhou and L. T. Dauer | 2022 | A million persons, a million dreams: a vision for a national center of radiation epidemiology and biology | International Journal of Radiation Biology | 10.1080/09553002.2021.1988183 |
| **82** | L. F. Borges, V. Jagadeesan, H. Goldberg, S. Gavini, W. K. Lo, R. Burakoff, N. Feldman and W. W. Chan | 2018 | Abnormal Bolus Reflux Is Associated With Poor Pulmonary Outcome in Patients With Idiopathic Pulmonary Fibrosis | Journal of Neurogastroenterology and Motility | 10.5056/jnm18023 |
| **83** | L. F. Borges, S. Salgado, K. E. Hathorn, N. Feldman, T. L. Carroll and W. W. Chan | 2022 | Failed Swallows on High-Resolution Manometry Independently Correlates With Severity of LPR Symptoms | Journal of Voice | 10.1016/j.jvoice.2020.09.003 |
| **84** | C. Bosetti, E. Traini, T. Alam, C. A. Allen, G. Carreras, K. Compton, C. Fitzmaurice, L. M. Force, S. Gallus, G. Gorini, J. D. Harvey, J. M. Kocarnik, C. La Vecchia, A. Lugo, M. Naghavi, A. Pennini, C. Piccinelli, L. Ronfani, R. X. Xu and L. Monasta | 2020 | National burden of cancer in Italy, 1990-2017: a systematic analysis for the global burden of disease study 2017 | Scientific Reports | 10.1038/s41598-020-79176-3 |
| **85** | E. Botteri, G. Peveri, P. Berstad, V. Bagnardi, G. Hoff, A. K. Heath, A. J. Cross, P. Vineis, L. Dossus, M. Johansson, H. Freisling, K. Matta, I. Huybrechts, S. L. F. Chen, K. B. Borch, T. M. Sandanger, T. H. Nost, C. C. Dahm, C. S. Antoniussen, S. T. Tin, A. Fournier, C. Marques, F. Artaud, M. J. Sanchez, M. Guevara, C. Santiuste, A. Agudo, R. Bajracharya, V. Katzke, F. Ricceri, C. Agnoli, M. M. Bergmann, M. B. Schulze, S. Panico, G. Masala, A. Tjonneland, A. Olsen, T. Stocks, J. Manjer, A. Aizpurua-Atxega, E. Weiderpass, E. Riboli, M. J. Gunter and P. Ferrari | 2024 | Lifestyle changes in middle age and risk of cancer: evidence from the European Prospective Investigation into Cancer and Nutrition | European Journal of Epidemiology | 10.1007/s10654-023-01059-4 |
| **86** | E. Bouchard, R. Sharma, N. Bachand, A. A. Gajadhar and E. J. Jenkins | 2017 | Pathology, clinical signs, and tissue distribution of <i>Toxoplasma gondii</i> in experimentally infected reindeer (<i>Rangifer tarandus</i>) | International Journal for Parasitology-Parasites and Wildlife | 10.1016/j.ijppaw.2017.08.004 |
| **87** | J. M. Brandenburg, A. C. Jenke, A. Stern, M. T. J. Daum, A. Schulze, R. Younis, P. Petrynowski, T. Davitashvili, V. Vanat, N. Bhasker, S. Schneider, L. Mündermann, A. Reinke, F. R. Kolbinger, V. Jörns, F. Fritz-Kebede, M. Dugas, L. Maier-Hein, R. Klotz, M. Distler, J. Weitz, B. P. Müller-Stich, S. Speidel, S. Bodenstedt and M. Wagner | 2023 | Active learning for extracting surgomic features in robot-assisted minimally invasive esophagectomy: a prospective annotation study | Surgical Endoscopy and Other Interventional Techniques | 10.1007/s00464-023-10447-6 |
| **88** | E. Brosens, M. Ploeg, Y. van Bever, A. E. Koopmans, H. Ijsselstijn, R. J. Rottier, R. Wijnen, D. Tibboel and A. de Klein | 2014 | Clinical and etiological heterogeneity in patients with tracheo-esophageal malformations and associated anomalies | European Journal of Medical Genetics | 10.1016/j.ejmg.2014.05.009 |
| **89** | C. S. Brown, B. Lapin, C. Wang, J. L. Goldstein, J. G. Linn, W. Denham, S. P. Haggerty, M. S. Talamonti, J. A. Howington, J. Carbray and M. B. Ujiki | 2015 | Reflux control is important in the management of Barrett's Esophagus: results from a retrospective 1,830 patient cohort | Surgical Endoscopy and Other Interventional Techniques | 10.1007/s00464-015-4103-3 |
| **90** | O. Bruserud, D. E. Costea, S. Laakso, B. Z. Garty, E. Mathisen, A. Mäkitie, O. Mäkitie and E. S. Husebye | 2018 | Oral Tongue Malignancies in Autoimmune Polyendocrine Syndrome Type 1 | Frontiers in Endocrinology | 10.3389/fendo.2018.00463 |
| **91** | N. Brusselaers, J. Maret-Ouda, P. Konings, H. B. El-Serag and J. Lagergren | 2017 | Menopausal hormone therapy and the risk of esophageal and gastric cancer | International Journal of Cancer | 10.1002/ijc.30588 |
| **92** | C. Bryce, M. Bucaj and R. Gazda | 2022 | Barrett Esophagus: Rapid Evidence Review | American Family Physician |  |
| **93** | M. F. Buas, H. W. Gu, D. Djukovic, J. J. Zhu, L. Onstad, B. J. Reid, D. Raftery and T. L. Vaughan | 2017 | Candidate serum metabolite biomarkers for differentiating gastroesophageal reflux disease, Barrett's esophagus, and high-grade dysplasia/esophageal adenocarcinoma | Metabolomics | 10.1007/s11306-016-1154-y |
| **94** | M. F. Buas, Q. C. He, L. G. Johnson, L. Onstad, D. M. Levine, A. P. Thrift, P. Gharahkhani, C. Palles, J. Lagergren, R. C. Fitzgerald, W. M. Ye, C. Caldas, N. C. Bird, N. J. Shaheen, L. Bernstein, M. D. Gammon, A. H. Wu, L. J. Hardie, P. D. Pharoah, G. Liu, P. Iyer, D. A. Corley, H. A. Risch, W. H. Chow, H. Prenen, L. Chegwidden, S. Love, S. Attwood, P. Moayyedi, D. MacDonald, R. Harrison, P. Watson, H. Barr, J. deCaestecker, I. Tomlinson, J. Jankowski, D. C. Whiteman, S. MacGregor, T. L. Vaughan and M. M. Madeleine | 2017 | Germline variation in inflammation-related pathways and risk of Barrett's oesophagus and oesophageal adenocarcinoma | Gut | 10.1136/gutjnl-2016-311622 |
| **95** | M. F. Buas, D. M. Levine, K. W. Makar, H. Utsugi, L. Onstad, X. H. Li, P. C. Galipeau, N. J. Shaheen, L. J. Hardie, Y. Romero, L. Bernstein, M. D. Gammon, A. G. Casson, N. C. Bird, H. A. Risch, W. M. Ye, G. Liu, D. A. Corley, P. L. Blount, R. C. Fitzgerald, D. C. Whiteman, A. H. Wu, B. J. Reid and T. L. Vaughan | 2014 | Integrative post-genome-wide association analysis of <i>CDKN2A</i> and <i>TP53</i> SNPs and risk of esophageal adenocarcinoma | Carcinogenesis | 10.1093/carcin/bgu207 |
| **96** | M. F. Buas, L. Onstad, D. M. Levine, H. A. Risch, W. H. Chow, G. Liu, R. C. Fitzgerald, L. Bernstein, W. M. Ye, N. C. Bird, Y. Romero, A. G. Casson, D. A. Corley, N. J. Shaheen, A. H. Wu, M. D. Gammon, B. J. Reid, L. J. Hardie, U. Peters, D. C. Whiteman and T. L. Vaughan | 2015 | MiRNA-Related SNPs and Risk of Esophageal Adenocarcinoma and Barrett's Esophagus: Post Genome-Wide Association Analysis in the BEACON Consortium | Plos One | 10.1371/journal.pone.0128617 |
| **97** | A. Budukh, S. H. Shah, S. Kulkarni, S. Pimple, S. Patil, D. Chaukar and C. Pramesh | 2022 | Tobacco and cancer awareness program among school children in rural areas of Ratnagiri district of Maharashtra state in India | Indian Journal of Cancer | 10.4103/ijc.IJC_629_19 |
| **98** | J. Budzynski, M. Ziólkowski, M. Klopocka and D. Czarnecki | 2016 | Blood glucose and lipid concentrations after overload are not associated with the risk of alcohol relapse | Drug and Alcohol Dependence | 10.1016/j.drugalcdep.2016.02.029 |
| **99** | Y. Q. Cai, J. X. Lin, W. B. Wei, P. X. Chen and K. T. Yao | 2022 | Burden of esophageal cancer and its attributable risk factors in 204 countries and territories from 1990 to 2019 | Frontiers in Public Health | 10.3389/fpubh.2022.952087 |
| **100** | L. Calderón-Garcidueñas, R. Reynoso-Robles, B. Pérez-Guillé, P. S. Mukherjee and A. Gónzalez-Maciel | 2017 | Combustion-derived nanoparticles, the neuroenteric system, cervical vagus, hyperphosphorylated alpha synuclein and tau in young Mexico City residents | Environmental Research | 10.1016/j.envres.2017.08.008 |
| **101** | S. J. Callahan, M. Xia, S. Murray and K. R. Flaherty | 2016 | Clinical characteristics in patients with asymmetric idiopathic pulmonary fibrosis | Respiratory Medicine | 10.1016/j.rmed.2016.08.028 |
| **102** | C. M. Calvin, G. D. Batty, G. Der, C. E. Brett, A. Taylor, A. Pattie, I. Cukic and I. J. Deary | 2017 | Childhood intelligence in relation to major causes of death in 68 year follow-up: prospective population study | Bmj-British Medical Journal | 10.1136/bmj.j2708 |
| **103** | J. Cao, Z. Q. Chen, C. Y. Tian, J. Yu, H. F. Zhang, J. W. Yang and W. J. Yang | 2020 | A Shared Susceptibility Locus in the <i>p53</i> Gene for both Gastric and Esophageal Cancers in a Northwestern Chinese Population | Genetic Testing and Molecular Biomarkers | 10.1089/gtmb.2020.0192 |
| **104** | J. J. Cao, H. X. Xu, W. Li, Z. Q. Guo, Y. Lin, Y. Y. Shi, W. Hu, Y. Ba, S. Y. Li, Z. N. Li, K. H. Wang, J. Wu, Y. He, J. J. Yang, C. H. Xie, F. X. Zhou, X. X. Song, G. Y. Chen, W. J. Ma, S. X. Luo, Z. H. Chen, M. H. Cong, H. Ma, C. L. Zhou, W. Wang, Q. Luo, Y. M. Shi, Y. M. Qi, H. P. Jiang, W. X. Guan, J. Q. Chen, J. X. Chen, Y. Fang, L. Zhou, Y. D. Feng, R. S. Tan, J. W. Ou, Q. C. Zhao, J. X. Wu, X. Lin, L. Q. Yang, Z. M. Fu, C. Wang, L. Deng, T. Li, C. H. Song, H. P. Shi, O. Invest Nutr Status Clinical and O. Chinese Soc Nutr | 2021 | Nutritional assessment and risk factors associated to malnutrition in patients with esophageal cancer | Current Problems in Cancer | 10.1016/j.currproblcancer.2020.100638 |
| **105** | G. Capurso and E. Lahner | 2017 | The interaction between smoking, alcohol and the gut microbiome | Best Practice & Research Clinical Gastroenterology | 10.1016/j.bpg.2017.10.006 |
| **106** | G. Caruana, R. Cachia, S. Micallef, M. Sammut and J. Psaila | 2024 | Metastatic mediastinal hepatoid adenocarcinoma | Bmj Case Reports | 10.1136/bcr-2022-253747 |
| **107** | A. M. Carvalho, A. M. Miranda, F. A. Santos, A. P. M. Loureiro, R. M. Fisberg and D. M. Marchioni | 2015 | High intake of heterocyclic amines from meat is associated with oxidative stress | British Journal of Nutrition | 10.1017/s0007114515000628 |
| **108** | L. Cattelan, F. M. Ghazawi, M. Le, F. Lagacé, E. Savin, A. Zubarev, J. Gantchev, M. Tomaszewski, D. Sasseville, K. Waschke and I. V. Litvinov | 2020 | Epidemiologic trends and geographic distribution of esophageal cancer in Canada: A national population-based study | Cancer Medicine | 10.1002/cam4.2700 |
| **109** | M. K. A. Chaar, A. Godin, W. S. Harmsen, C. Wzientek, S. A. Saddoughi, C. L. Hallemeier, S. D. Cassivi, F. C. Nichols, J. S. Reisenauer, K. R. Shen, L. F. Tapias, D. A. Wigle and S. H. Blackmon | 2023 | Determinants of Long-term Survival Decades After Esophagectomy for Esophageal Cancer | Annals of Thoracic Surgery | 10.1016/j.athoracsur.2023.05.033 |
| **110** | W. C. Chan, I. Y. Millwood, C. Kartsonaki, H. D. Du, Y. Guo, Y. P. Chen, Z. Bian, R. G. Walters, J. Lv, P. He, C. Hu, L. M. Li, L. Yang, Z. M. Chen and C. K. B. C. Grp | 2021 | Spicy food consumption and risk of gastrointestinal-tract cancers: findings from the China Kadoorie Biobank | International Journal of Epidemiology | 10.1093/ije/dyaa275 |
| **111** | P. Y. Chang, W. Y. Huang, C. L. Lin, T. C. Huang, Y. Y. Wu, J. H. Chen and C. H. Kao | 2015 | Propranolol Reduces Cancer Risk A Population-Based Cohort Study | Medicine | 10.1097/md.0000000000001097 |
| **112** | Y. K. Chao, H. Y. Ku, C. Y. Chen and T. W. Liu | 2017 | Induction therapy before surgery improves survival in patients with clinical T3N0 esophageal cancer: a nationwide study in Taiwan | Diseases of the Esophagus | 10.1093/dote/dox103 |
| **113** | B. F. Chen, Y. W. Jiao, F. K. Yaolong, T. S. Li, Y. X. Liu, M. Q. Wang, X. L. Gu and X. H. Feng | 2019 | The <i>POLR2E</i> rs3787016 polymorphism is strongly associated with the risk of female breast and cervical cancer | Pathology Research and Practice | 10.1016/j.prp.2019.02.015 |
| **114** | C. Chen, X. Q. Cheng, S. Y. Li, H. H. Chen, M. J. Cui, L. L. Bian and H. Jin | 2021 | A Novel Signature for Predicting Prognosis of Smoking-Related Squamous Cell Carcinoma | Frontiers in Genetics | 10.3389/fgene.2021.666371 |
| **115** | C. L. Chen, W. C. Chang, C. H. Yi, J. S. Hung, T. T. Liu, W. Y. Lei and C. S. Hsu | 2019 | Association of coffee consumption and liver fibrosis progression in patients with HBeAg-negative chronic hepatitis B: A 5-year population-based cohort study | Journal of the Formosan Medical Association | 10.1016/j.jfma.2018.08.002 |
| **116** | C. X. Chen, T. M. Wen and Q. Y. Zhao | 2019 | The Change of Laboratory Tests Could Be Predictive Factors for Infection after McKeown Esophagogastrectomy | Biomed Research International | 10.1155/2019/9718705 |
| **117** | D. N. Chen, N. B. Fan, J. X. Mo, W. D. Wang, R. Q. Wang, Y. F. Chen, J. Hu and Z. S. Wen | 2019 | Multiple primary malignancies for squamous cell carcinoma and adenocarcinoma of the esophagus | Journal of Thoracic Disease | 10.21037/jtd.2019.08.51 |
| **118** | J. H. Chen, Z. T. Wu, H. Gao, L. Li, Y. L. Wang, J. J. Han, C. Zhang, P. P. Ding and J. Wu | 2023 | Association between air temperature and emergency admission for esophagogastric variceal bleeding: a case-crossover study in Beijing, China | Bmc Gastroenterology | 10.1186/s12876-023-02683-w |
| **119** | L. Chen, G. Y. Zhu, L. She, Y. N. Ding, C. Q. Yang and F. S. Zhu | 2021 | Analysis of Risk Factors and Establishment of a Prediction Model for Endoscopic Primary Bile Reflux: A Single-Center Retrospective Study | Frontiers in Medicine | 10.3389/fmed.2021.758771 |
| **120** | W. C. Chen, E. Singh, M. Muchengeti, D. Bradshaw, C. G. Mathew, C. B. de Villiers, C. M. Lewis, T. Waterboer, R. Newton and F. Sitas | 2020 | Johannesburg Cancer Study (JCS): contribution to knowledge and opportunities arising from 20 years of data collection in an African setting | Cancer Epidemiology | 10.1016/j.canep.2020.101701 |
| **121** | W. Q. Chen, H. Li, J. S. Ren, R. S. Zheng, J. F. Shi, J. Li, M. M. Cao, D. Q. Sun, S. Y. He, X. B. Sun, X. Q. Cao, S. X. Feng, J. Y. Zhou, P. F. Luo, Z. Q. Zha, S. C. Jia, J. L. Wang, H. M. Ma, H. M. Zeng, K. Canfell and J. He | 2021 | Selection of high-risk individuals for esophageal cancer screening: A prediction model of esophageal squamous cell carcinoma based on a multicenter screening cohort in rural China | International Journal of Cancer | 10.1002/ijc.33208 |
| **122** | X. D. Chen, Z. Y. Yuan, M. Lu, Y. C. Zhang, L. Jin and W. M. Ye | 2017 | Poor oral health is associated with an increased risk of esophageal squamous cell carcinoma - a population-based case-control study in China | International Journal of Cancer | 10.1002/ijc.30484 |
| **123** | X. Y. Chen, Y. J. Zhang, X. J. Zhou, M. Wang, F. F. Na, L. Zhou, Y. Xu, B. W. Zou, J. X. Xue, Y. M. Liu and Y. L. Gong | 2023 | Involved-field irradiation or elective-nodal irradiation in neoadjuvant chemo-radiotherapy for locally-advanced esophageal cancer: comprehensive analysis for dosimetry, treatment-related complications, impact on lymphocyte, patterns of failure and survival | Frontiers in Oncology | 10.3389/fonc.2023.1274924 |
| **124** | Y. Chen, Z. Zhang, G. L. Jiang and K. L. Zhao | 2016 | Gross tumor volume is the prognostic factor for squamous cell esophageal cancer patients treated with definitive radiotherapy | Journal of Thoracic Disease | 10.21037/jtd.2016.04.08 |
| **125** | Y. F. Chen, Q. Li, D. T. Chen, J. H. Pan, Y. H. Chen, Z. S. Wen and W. A. Zeng | 2016 | Prognostic value of pre-operative serum uric acid levels in esophageal squamous cell carcinoma patients who undergo R0 esophagectomy | Cancer Biomarkers | 10.3233/cbm-160621 |
| **126** | Y. H. Chen, T. M. Jao, Y. L. Shiue, I. J. Feng and P. I. Hsu | 2022 | Prevalence and risk factors for <i>Candida </i>esophagitis among human immunodeficiency virus-negative individuals | World Journal of Clinical Cases | 10.12998/wjcc.v10.i30.10896 |
| **127** | Y. L. Chen, T. H. Chen and J. Y. Fang | 2023 | Burden of gastrointestinal cancers in China from 1990 to 2019 and projection through 2029 | Cancer Letters | 10.1016/j.canlet.2023.216127 |
| **128** | Z. F. Chen, Y. Zheng, P. Fan, M. Li, W. Liu, H. Yuan, X. Liu, Z. Y. Zhang, Z. Q. Wu, Y. P. Wang, R. Ji, Q. H. Guo, Y. W. Ye, J. H. Zhang, X. H. Li, F. An, L. Z. Lu, Y. P. Li, X. Wang, J. Zhang, Q. L. Guan, Q. Li, M. Liu, Q. Ren, X. B. Hu, H. Lu, H. L. Zhang, Y. Zhao, X. Gou, X. C. Shu, J. Wang, Z. A. Hu, S. Q. Xue, J. K. Liu and Y. N. Zhou | 2023 | Risk factors in the development of gastric adenocarcinoma in the general population: A cross-sectional study of the Wuwei Cohort | Frontiers in Microbiology | 10.3389/fmicb.2022.1024155 |
| **129** | Z. M. Chen, R. Peto, A. Iona, Y. Guo, Y. P. Chen, Z. Bian, L. Yang, W. Y. Zhang, F. Lu, J. S. Chen, R. Collins, L. M. Li and B. China Kadoorie | 2015 | Emerging tobacco-related cancer risks in China: A nationwide, prospective study of 0.5 million adults | Cancer | 10.1002/cncr.29560 |
| **130** | S. M. Cheng, K. L. Hung, Y. J. Wang, S. P. Ng and H. F. Chiang | 2021 | Influence of gastric morphology on gastroesophageal reflux in adults An observational study | Medicine | 10.1097/md.0000000000027241 |
| **131** | Y. L. Cheng, M. H. Yu, Q. Yao, T. He, R. F. Zhang and Z. Q. Long | 2023 | The impact of indirect notification of a cancer diagnosis and a risk model based on it to predict the prognosis of postoperative stage T3 esophageal cancer patients | Medicine | 10.1097/md.0000000000035895 |
| **132** | R. Chia, S. Saez-Atienzar, N. Murphy, A. Chiò, C. Blauwendraat, R. H. Roda, P. J. Tienari, H. J. Kaminski, R. Ricciardi, M. Guida, A. De Rosa, L. Petrucci, A. Evoli, C. Provenzano, D. B. Drachman, B. J. Traynor and C. Int Myasthenia Gravis Genomics | 2022 | Identification of genetic risk loci and prioritization of genes and pathways for myasthenia gravis: a genome-wide association study | Proceedings of the National Academy of Sciences of the United States of America | 10.1073/pnas.2108672119 |
| **133** | C. L. Chiang, Y. W. Hu, C. H. Wu, Y. T. Chen, C. J. Liu, Y. H. Luo, Y. M. Chen, T. J. Chen, K. C. Su and K. T. Chou | 2016 | Spectrum of cancer risk among Taiwanese with chronic obstructive pulmonary disease | International Journal of Clinical Oncology | 10.1007/s10147-016-0983-z |
| **134** | S. H. Chien, C. J. Liu, Y. C. Hong, C. J. Teng, Y. W. Hu, F. C. Ku, C. M. Yeh, T. J. Chiou, J. P. Gau and C. H. Tzeng | 2015 | Development of second primary malignancy in patients with non-Hodgkin lymphoma: a nationwide population-based study | Journal of Cancer Research and Clinical Oncology | 10.1007/s00432-015-1979-1 |
| **135** | S. S. Chisholm, J. E. Khoury, M. M. Jamal, C. Palacio, S. Pudhota and K. J. Vega | 2017 | The frequency of histologically confirmed Barrett's esophagus varies by the combination of ethnicity and gender | Journal of Gastrointestinal Oncology | 10.21037/jgo.2016.12.07 |
| **136** | B. Chitti, A. Pham, S. Marcott, X. Wang, L. Potters, A. G. Wernicke and B. Parashar | 2018 | Temporal Changes in Esophageal Cancer Mortality by Geographic Region: A Population-based Analysis | Cureus Journal of Medical Science | 10.7759/cureus.3596 |
| **137** | S. W. Cho, W. G. Jeong, J. E. Lee, I. J. Oh, S. Y. Song, H. M. Park, H. J. Lee and Y. H. Kim | 2022 | Clinical implication of interstitial lung abnormality in elderly patients with early-stage non-small cell lung cancer | Thoracic Cancer | 10.1111/1759-7714.14341 |
| **138** | B. H. Choi, J. Church, J. Sonett and R. P. Kiran | 2023 | Colonic interposition in esophagectomy: an ACS-NSQIP study | Surgical Endoscopy and Other Interventional Techniques | 10.1007/s00464-023-10420-3 |
| **139** | B. J. Choi, S. Lee, I. J. Lee, S. W. Park and S. Lee | 2020 | Gastric and rectal cancers in workers exposed to asbestos: a case series | Annals of Occupational and Environmental Medicine | 10.35371/aoem.2020.32.e4 |
| **140** | R. Chowdhury, S. E. Sarnat, L. Darrow, W. McClellan and K. Steenland | 2014 | Mortality among participants in a lead surveillance program | Environmental Research | 10.1016/j.envres.2014.03.008 |
| **141** | S. Christakoudi, A. Kakourou, G. Markozannes, I. Tzoulaki, E. Weiderpass, P. Brennan, M. Gunter, C. C. Dahm, K. Overvad, A. Olsen, A. Tjonneland, M. C. Boutron-Ruault, A. L. Madika, G. Severi, V. Katzke, T. Kühn, M. M. Bergmann, H. Boeing, A. Karakatsani, G. Martimianaki, P. Thriskos, G. Masala, S. Sieri, S. Panico, R. Tumino, F. Ricceri, A. Agudo, D. Redondo-Sánchez, S. M. Colorado-Yohar, O. Mokoroa, O. Melander, T. Stocks, C. Häggström, S. Harlid, B. Bueno-de-Mesquita, C. H. van Gils, R. C. Vermeulen, K. T. Khaw, N. J. Wareham, T. Y. N. Tong, H. Freisling, M. Johansson, H. Lennon, D. Aune, E. Ribolil, D. Trichopoulos, A. Trichopoulou and K. K. Tsilidis | 2020 | Blood pressure and risk of cancer in the European Prospective Investigation into Cancer and Nutrition | International Journal of Cancer | 10.1002/ijc.32576 |
| **142** | S. Christie, R. O'Reilly, H. Li, G. A. Wittert and A. J. Page | 2020 | Biphasic effects of methanandamide on murine gastric vagal afferent mechanosensitivity | Journal of Physiology-London | 10.1113/jp278696 |
| **143** | A. Christopoulou, A. Ardavanis, C. Papandreou, G. Koumakis, G. Papatsimpas, P. Papakotoulas, N. Tsoukalas, C. Andreadis, G. Samelis, P. Papakostas, G. Aravantinos, N. Ziras, M. Souggleri, C. Kalofonos, E. Samantas, P. Makrantonakis, G. Pentheroudakis, A. Athanasiadis, H. Stergiou, A. Bokas, A. Grivas, E. S. Tripodaki, I. Varthalitis, E. Timotheadou and I. Boukovinas | 2022 | Prophylaxis of cancer-associated venous thromboembolism with low-molecular-weight heparin-tinzaparin: Real world evidence | Oncology Letters | 10.3892/ol.2022.13235 |
| **144** | Y. S. Chuan, M. C. Wu, Y. K. Wang, Y. H. Chen, C. H. Kuo, D. C. Wu, M. T. Wu and I. C. Wu | 2019 | Risks of substance uses, alcohol flush response, <i>Helicobacter pylori</i> infection and upper digestive tract diseases-An endoscopy cross-sectional study | Kaohsiung Journal of Medical Sciences | 10.1002/kjm2.12071 |
| **145** | Y. S. Chuang, M. C. Wu, F. J. Yu, Y. K. Wang, C. Y. Lu, D. C. Wu, C. T. Kuo, M. T. Wu and I. C. Wu | 2017 | Effects of alcohol consumption, cigarette smoking, and betel quid chewing on upper digestive diseases: a large cross-sectional study and meta-analysis | Oncotarget | 10.18632/oncotarget.20831 |
| **146** | T. Cleverley, I. Meredith, D. Sika-Paotonu and J. Gurney | 2023 | Cancer incidence, mortality and survival for Pacific Peoples in Aotearoa New Zealand | New Zealand Medical Journal |  |
| **147** | H. Çolak, F. E. Günes, Y. Alahdab and B. Karakoyun | 2022 | Investigation of Eating Habits in Patients with Functional Dyspepsia | Turkish Journal of Gastroenterology | 10.5152/tjg.2022.21502 |
| **148** | H. G. Coleman, R. T. Gray, K. W. Lau, C. McCaughey, P. V. Coyle, L. J. Murray and B. T. Johnston | 2016 | Socio-economic status and lifestyle factors are associated with achalasia risk: A population-based case-control study | World Journal of Gastroenterology | 10.3748/wjg.v22.i15.4002 |
| **149** | H. G. Coleman, S. H. Xie and J. Lagergren | 2018 | The Epidemiology of Esophageal Adenocarcinoma | Gastroenterology | 10.1053/j.gastro.2017.07.046 |
| **150** | G. Collatuzzo, G. Alicandro, P. Bertuccio, C. Pelucchi, R. Bonzi, D. Palli, M. Ferraroni, W. M. Ye, A. Plymoth, D. Zaridze, D. Maximovich, N. Aragones, G. Castaño-Vinyals, J. Vioque, M. G. de la Hera, Z. F. Zhang, J. F. Hu, L. Lopez-Carrillo, M. López-Cervantes, M. Dalmartello, L. N. Mu, M. H. Ward, C. Rabkin, G. P. Yu, M. C. Camargo, M. P. Curado, N. Lunet, E. Negri, C. La Vecchia and P. Boffetta | 2022 | Peptic ulcer as mediator of the association between risk of gastric cancer and socioeconomic status, tobacco smoking, alcohol drinking and salt intake | Journal of Epidemiology and Community Health | 10.1136/jech-2022-219074 |
| **151** | G. Collatuzzo, J. C. Lainez, C. Pelucchi, E. Negri, R. Bonzi, D. Palli, M. Ferraroni, Z. F. Zhang, G. P. Yu, N. Lunet, S. Morais, L. Lopez-Carrillo, D. Zaridze, D. Maximovitch, M. Guevara, V. Santos-Sanchez, J. Vioque, M. G. de la Hera, M. H. Ward, R. Malekzadeh, M. Pakseresht, R. U. Hernández-Ramirez, F. Turati, C. S. Rabkin, L. M. Liao, R. Sinha, M. López-Cervantes, S. Tsugane, A. Hidaka, M. C. Camargo, M. P. Curado, N. Zubair, D. Kristjansson, S. Shah, C. La Vecchia and P. Boffetta | 2024 | The association between dietary fiber intake and gastric cancer: a pooled analysis of 11 case-control studies | European Journal of Nutrition | 10.1007/s00394-024-03388-w |
| **152** | G. Collatuzzo, C. Pelucchi, E. Negri, M. Kogevinas, J. M. Huerta, J. Vioque, M. G. de la Hera, S. Tsugane, G. S. Hamada, A. Hidaka, Z. F. Zhang, M. C. Camargo, M. P. Curado, N. Lunet, C. La Vecchia and P. Boffetta | 2023 | Sleep Duration and Stress Level in the Risk of Gastric Cancer: A Pooled Analysis of Case-Control Studies in the Stomach Cancer Pooling (StoP) Project | Cancers | 10.3390/cancers15174319 |
| **153** | G. Collatuzzo, C. Pelucchi, E. Negri, L. Lopez-Carrillo, S. Tsugane, A. Hidaka, G. S. Hamada, R. U. Hernandez-Ramirez, M. Lopez-Cervantes, R. Malekzadeh, F. Pourfarzi, L. N. Mu, Z. F. Zhang, N. Lunet, C. La Vecchia and P. Boffetta | 2021 | Exploring the interactions between Helicobacter pylori (Hp) infection and other risk factors of gastric cancer: A pooled analysis in the Stomach cancer Pooling (StoP) Project | International Journal of Cancer | 10.1002/ijc.33678 |
| **154** | G. Collatuzzo, F. Teglia, C. Pelucchi, E. Negri, C. S. Rabkin, L. M. Liao, R. Sinha, L. López-Carrillo, N. Lunet, S. Morais, N. Aragonés, V. Moreno, J. Vioque, M. G. de la Hera, M. H. Ward, R. Malekzadeh, M. Pakseresht, R. U. Hernández-Ramírez, M. López-Cervantes, R. Bonzi, M. Dalmartello, S. Tsugane, A. Hidaka, M. C. Camargo, M. P. Curado, Z. F. Zhang, N. Zubair, C. La Vecchia, S. Shah and P. Boffetta | 2022 | Inverse Association between Dietary Iron Intake and Gastric Cancer: A Pooled Analysis of Case-Control Studies of the Stop Consortium | Nutrients | 10.3390/nu14122555 |
| **155** | M. B. Cook, D. A. Corley, L. J. Murray, L. M. Liao, F. Kamangar, W. M. Ye, M. D. Gammon, H. A. Risch, A. G. Casson, N. D. Freedman, W. H. Chow, A. H. Wu, L. Bernstein, O. Nyrén, N. Pandeya, D. C. Whiteman and T. L. Vaughan | 2014 | Gastroesophageal Reflux in Relation to Adenocarcinomas of the Esophagus: A Pooled Analysis from the Barrett's and Esophageal Adenocarcinoma Consortium (BEACON) | Plos One | 10.1371/journal.pone.0103508 |
| **156** | M. B. Cook, S. N. Wood, B. D. Cash, P. Young, R. D. Acosta, R. T. Falk, R. M. Pfeiffer, N. Hu, H. Su, L. M. Wang, C. Y. Wang, B. Gherman, C. Giffen, C. Dykes, V. Turcotte, P. Caron, C. Guillemette, S. M. Dawsey, C. C. Abnet, P. L. Hyland and P. R. Taylor | 2015 | Association Between Circulating Levels of Sex Steroid Hormones and Barrett's Esophagus in Men: A Case-Control Analysis | Clinical Gastroenterology and Hepatology | 10.1016/j.cgh.2014.08.027 |
| **157** | C. Correa, C. Mallarino, R. Peña, L. C. Rincón, G. Gracia and I. Zarante | 2014 | Congenital malformations of pediatric surgical interest: Prevalence, risk factors, and prenatal diagnosis between 2005 and 2012 in the capital city of a developing country. Bogota, Colombia | Journal of Pediatric Surgery | 10.1016/j.jpedsurg.2014.03.001 |
| **158** | C. Cotton, P. Alton, D. M. Hughes and S. S. Zhao | 2023 | Genetic liability to gastro-esophageal reflux disease, obesity, and risk of idiopathic pulmonary fibrosis | Respiratory Investigation | 10.1016/j.resinv.2023.02.005 |
| **159** | B. Dabo, C. Pelucchi, M. Rota, H. Jain, P. Bertuccio, R. Bonzi, D. Palli, M. Ferraroni, Z. F. Zhang, A. Sanchez-Anguiano, Y. T. H. Pham, C. T. D. Tran, A. G. Pham, G. P. Yu, T. C. Nguyen, J. Muscat, S. Tsugane, A. Hidaka, G. S. Hamada, D. Zaridze, D. Maximovitch, M. Kogevinas, N. F. de Larrea, S. Boccia, R. Pastorino, R. C. Kurtz, A. Lagiou, P. Lagiou, J. Vioque, M. C. Camargo, M. P. Curado, N. Lunet, P. Boffetta, E. Negri, C. La Vecchia and H. N. Luu | 2022 | The association between diabetes and gastric cancer: results from the Stomach Cancer Pooling Project Consortium | European Journal of Cancer Prevention | 10.1097/cej.0000000000000703 |
| **160** | D. S. Dahiya, A. Kichloo, H. Shaka, J. Singh, E. Edigin, D. Solanki, P. O. Eseaton and F. Wani | 2021 | Gastroparesis with Cannabis Use: A Retrospective Study from the Nationwide Inpatient Sample | Postgraduate Medicine | 10.1080/00325481.2021.1940219 |
| **161** | J. Y. Dai, J. D. Tapsoba, M. F. Buas, L. E. Onstad, D. M. Levine, H. A. Risch, W. H. Chow, L. Bernstein, W. M. Ye, J. Lagergren, N. C. Bird, D. A. Corley, N. J. Shaheen, A. H. Wu, B. J. Reid, L. J. Hardie, D. C. Whiteman and T. L. Vaughan | 2015 | A Newly Identified Susceptibility Locus near <i>FOXP1</i> Modifies the Association of Gastroesophageal Reflux with Barrett's Esophagus | Cancer Epidemiology Biomarkers & Prevention | 10.1158/1055-9965.Epi-15-0507 |
| **162** | J. Y. Dai, J. D. Tapsoba, M. F. Buas, H. A. Risch, T. L. Vaughan and B. Consortium | 2016 | Constrained Score Statistics Identify Genetic Variants Interacting with Multiple Risk Factors in Barrett's Esophagus | American Journal of Human Genetics | 10.1016/j.ajhg.2016.06.018 |
| **163** | G. E. Darling, F. Li, D. Patsios, C. Massey, A. G. Wallis, L. Coate, S. Keshavjee, A. Pierre, M. De Perrot, K. Yasufuku, M. Cypel and T. Waddell | 2015 | Neoadjuvant chemoradiation and surgery improves survival outcomes compared with definitive chemoradiation in the treatment of stage IIIA N2 non-small-cell lung cancer | European Journal of Cardio-Thoracic Surgery | 10.1093/ejcts/ezu504 |
| **164** | R. de Mutsert, Q. Sun, W. C. Willett, F. B. Hu and R. M. van Dam | 2014 | Overweight in Early Adulthood, Adult Weight Change, and Risk of Type 2 Diabetes, Cardiovascular Diseases, and Certain Cancers in Men: a Cohort Study | American Journal of Epidemiology | 10.1093/aje/kwu052 |
| **165** | D. De Rocchi, A. Zona, R. Turnino, V. Egidi and R. Pasetto | 2021 | Mortality temporal trends and cancer incidence profiles of residents in the petrochemical industrially contaminated town of Gela (Sicily, Italy) | Annali Dell Istituto Superiore Di Sanita | 10.4415/ann_21_02_10 |
| **166** | A. Del Monaco, C. Dimitriadis, S. Xie, G. Benke, M. R. Sim and K. Walker-Bone | 2023 | Workers in Australian prebake aluminium smelters: update on risk of mortality and cancer incidence in the Healthwise cohort | Occupational and Environmental Medicine | 10.1136/oemed-2022-108605 |
| **167** | J. Y. Deng, J. H. Zhang, C. Y. Wang, Q. Wei, D. Z. Zhou and K. L. Zhao | 2016 | Methylation and expression of PTPN22 in esophageal squamous cell carcinoma | Oncotarget | 10.18632/oncotarget.11581 |
| **168** | Y. X. Deng, T. Qiu, N. Patel, S. Zhou, T. Xue and H. J. Zhang | 2019 | Clinical Management of Risk of Radiation Pneumonia with Serum Markers During the Radiotherapy for Patients with Thoracic Malignant Tumors | Cancer Management and Research | 10.2147/cmar.S231995 |
| **169** | D. J. Desilets, B. H. Nathanson and F. Navab | 2014 | Barrett's Esophagus in Practice: Gender and Screening Issues | Journal of Mens Health | 10.1089/jomh.2014.0037 |
| **170** | L. Dhaliwal, D. C. Codipilly, P. Gandhi, M. L. Johnson, R. Lansing, K. N. K. Wang, C. L. Leggett, D. A. Katzka and P. G. Iyer | 2021 | Neoplasia Detection Rate in Barrett's Esophagus and Its Impact on Missed Dysplasia: Results from a Large Population-Based Database | Clinical Gastroenterology and Hepatology | 10.1016/j.cgh.2020.07.034 |
| **171** | P. K. Dhillon, P. Mathur, A. Nandakumar, C. Fitzmaurice, G. A. Kumar, R. Mehrotra, D. K. Shukla, G. K. Rath, P. C. Gupta, R. Swaminathan, J. S. Thakur, S. Dey, C. Allen, R. A. Badwe, R. Dikshit, R. S. Dhaliwal, T. Kaur, A. C. Kataki, R. N. Visvveswara, P. Gangadharan, E. Dutta, M. Furtado, C. M. Varghese, D. Bhardwaj, P. Muraleedharan, C. M. Odell, S. Glenn, M. S. Bal, P. P. Bapsy, J. Bennett, V. K. Bodal, J. K. Chakma, S. Chakravarty, M. Chaturvedi, P. Das, V. Deshmane, N. Gangane, J. Harvey, P. Jayalekshmi, K. Jerang, S. C. Johnson, P. K. Julka, D. Kaushik, V. Khamo, S. Koyande, M. Kutz, W. B. Langstieh, K. B. Lingegowda, R. C. Mahajan, J. Mahanta, G. Majumdar, N. Manoharan, A. Mathew, B. M. Nene, S. Pati, P. K. Pradhan, V. Raina, R. Rama, C. Ramesh, K. Sathishkumar, K. Schelonka, P. Sebastian, K. Shackelford, J. Shah, Shanta, J. D. Sharma, A. Shrivastava, S. Tawsik, B. B. Tyagi, K. Vaitheeswaran, E. Vallikad, Y. Verma, E. Zomawia, S. S. Lim, T. Vos, R. Dandona, K. S. Reddy, M. Naghavi, C. J. L. Murray, S. Swaminathan, L. Dandona and I. India State-Level Dis Burden | 2018 | The burden of cancers and their variations across the states of India: the Global Burden of Disease Study 1990-2016 | Lancet Oncology | 10.1016/s1470-2045(18)30447-9 |
| **172** | C. G. Dietrich, T. Kottmann, J. Labenz, K. Streetz and P. Hellebrandt | 2019 | The "Aachen sings" study ("Aachen choir engagement study into GERD symptoms"): moderate singing and breathing exercises in a choir reduce reflux symptoms - a cohort study in non-specialist choristers | Zeitschrift Fur Gastroenterologie | 10.1055/a-0855-4339 |
| **173** | S. G. Dighe, L. Yan, S. Mukherjee, C. S. McGillicuddy, K. L. Hulme, S. N. Hochwald, S. Yendamuri, A. J. Bain, K. T. Robillard, K. B. Moysich, C. B. Ambrosone, A. E. Millen and M. F. Buas | 2021 | Clinical and Lifestyle-Related Prognostic Indicators among Esophageal Adenocarcinoma Patients Receiving Treatment at a Comprehensive Cancer Center | Cancers | 10.3390/cancers13184653 |
| **174** | I. Dina, O. Ginghina, C. D. Toderescu, C. Balalau, B. Galateanu, C. Negrei and C. Iacobescu | 2017 | Zenker's diverticulum and squamous esophageal cancer: a case report | Journal of Mind and Medical Sciences | 10.22543/7674.42.P193197 |
| **175** | H. Ding, Y. M. Chen, H. Qiu, C. Liu, Y. F. Wang, M. Q. Kang and W. F. Tang | 2017 | PPARG c.1347C&gt;T polymorphism is associated with cancer susceptibility: from a case-control study to a meta-analysis | Oncotarget | 10.18632/oncotarget.20925 |
| **176** | H. Y. Ding, S. Fan, L. Zhang, Z. Y. Hao and C. Z. Liang | 2017 | Does prostatitis increase the risk of prostate cancer? A meta-analysis | International Journal of Clinical and Experimental Medicine |  |
| **177** | N. N. Ding, Y. S. Mao, J. He, S. G. Gao, Y. Zhao, D. Yang, K. L. Sun, G. Y. Cheng, J. W. Mu, Q. Xue, D. L. Wang, J. Zhao, Y. S. Gao, X. Y. Liu, D. K. Fang, J. Li, Y. G. Wang, J. F. Huang, B. Wang and L. Z. Zhang | 2017 | Experiences in the management of anastomotic leakages and analysis of the factors affecting leakage healing in patients with esophagogastric junction cancer | Journal of Thoracic Disease | 10.21037/jtd.2017.02.34 |
| **178** | P. Dobsch, A. Mehrl and A. Kandulski | 2021 | Gastrointestinal endoscopy in geriatric patients | Gastroenterologe | 10.1007/s11377-021-00550-2 |
| **179** | S. Docimo, A. Mathew, A. J. Shope, J. S. Winder, R. S. Haluck and E. M. Pauli | 2017 | Reduced postoperative pain scores and narcotic use favor per-oral endoscopic myotomy over laparoscopic Heller myotomy | Surgical Endoscopy and Other Interventional Techniques | 10.1007/s00464-016-5034-3 |
| **180** | J. Dong, M. F. Buas, P. Gharahkhani, B. J. Kendall, L. Onstad, S. S. Zhao, L. A. Anderson, A. H. Wu, W. M. Ye, N. C. Bird, L. Bernstein, W. H. Chow, M. D. Gammon, G. Liu, C. Caldas, P. D. Pharoah, H. A. Risch, P. G. Iyer, B. J. Reid, L. J. Hardie, J. Lagergren, N. J. Shaheen, D. A. Corley, R. C. Fitzgerald, D. C. Whiteman, T. L. Vaughan, A. P. Thrift, Stomach and S. Oesophageal Canc | 2018 | Determining Risk of Barrett's Esophagus and Esophageal Adenocarcinoma Based on Epidemiologic Factors and Genetic Variants | Gastroenterology | 10.1053/j.gastro.2017.12.003 |
| **181** | J. Dong, P. Gharahkhani, W. H. Chow, M. D. Gammon, G. Liu, C. Caldas, A. H. Wu, W. M. Ye, L. Onstad, L. A. Anderson, L. Bernstein, P. D. Pharoah, H. A. Risch, D. A. Corley, R. C. Fitzgerald, P. G. Iyer, B. J. Reid, J. Lagergren, N. J. Shaheen, T. L. Vaughan, S. MacGregor, S. Love, C. Palles, I. Tomlinson, I. Gockel, A. May, C. Gerges, M. Anders, A. C. Böhmer, J. Becker, N. Kreuser, R. Thieme, T. Noder, M. Venerito, L. Veits, T. Schmidt, C. Schmidt, J. R. Izbicki, A. H. Hölscher, H. K. Lang, D. Lorenz, B. Schumacher, R. Mayershofer, Y. Vashist, K. Ott, M. Vieth, J. Weismüller, M. M. Nöthen, S. Moebus, M. Knapp, W. H. M. Peters, H. Neuhaus, T. Rösch, C. Ell, J. Jankowski, J. Schumacher, R. E. Neale, D. C. Whiteman, A. P. Thrift and S. Stomach Esophageal Canc | 2019 | No Association Between Vitamin D Status and Risk of Barrett's Esophagus or Esophageal Adenocarcinoma: A Mendelian Randomization Study | Clinical Gastroenterology and Hepatology | 10.1016/j.cgh.2019.01.041 |
| **182** | J. Dong, D. M. Levine, M. F. Buas, R. Zhang, L. Onstad, R. C. Fitzgerald, D. A. Corley, N. J. Shaheen, J. Lagergren, L. J. Hardie, B. J. Reid, P. G. Iyer, H. A. Risch, C. Caldas, I. Caldas, P. D. Pharoah, G. Liu, M. D. Gammon, W. H. Chow, L. Bernstein, N. C. Bird, W. M. Ye, A. H. Wu, L. A. Anderson, S. MacGregor, D. C. Whiteman, T. L. Vaughan, A. P. Thrift and S. O. C. Stomach Oesophageal Canc Study | 2018 | Interactions Between Genetic Variants and Environmental Factors Affect Risk of Esophageal Adenocarcinoma and Barrett's Esophagus | Clinical Gastroenterology and Hepatology | 10.1016/j.cgh.2018.03.007 |
| **183** | N. M. Donin, L. Kwan, A. T. Lenis, A. Drakaki and K. Chamie | 2019 | Second primary lung cancer in United States Cancer Survivors, 1992-2008 | Cancer Causes & Control | 10.1007/s10552-019-01161-7 |
| **184** | M. P. Dore, G. M. Pes, G. Bassotti, M. A. Farina, G. Marras and D. Y. Graham | 2016 | Risk factors for erosive and non-erosive gastroesophageal reflux disease and Barrett's esophagus in Nothern Sardinia | Scandinavian Journal of Gastroenterology | 10.1080/00365521.2016.1200137 |
| **185** | S. Dorosti, S. J. Ghoushchi, E. Sobhrakhshankhah, M. Ahmadi and A. Sharifi | 2020 | Application of gene expression programming and sensitivity analyses in analyzing effective parameters in gastric cancer tumor size and location | Soft Computing | 10.1007/s00500-019-04507-0 |
| **186** | J. Drahos, Q. Xiao, H. A. Risch, N. D. Freedman, C. C. Abnet, L. A. Anderson, L. Bernstein, L. Brown, W. H. Chow, M. D. Gammon, F. Kamangar, L. M. Liao, L. J. Murray, M. H. Ward, W. M. Ye, A. H. Wu, T. L. Vaughan, D. C. Whiteman and M. B. Cook | 2016 | Age-specific risk factor profiles of adenocarcinomas of the esophagus: A pooled analysis from the international BEACON consortium | International Journal of Cancer | 10.1002/ijc.29688 |
| **187** | F. J. Duan, C. H. Song, J. C. Shi, P. Wang, H. Ye, L. P. Dai, J. Y. Zhang and K. J. Wang | 2021 | Identification and epidemiological evaluation of gastric cancer risk factors: based on a field synopsis and meta-analysis in Chinese population | Aging-Us |  |
| **188** | J. Dudley, T. Wieczorek, M. Selig, H. Cheung, J. Shen, R. Odze, V. Deshpande and L. Zukerberg | 2017 | Clinicopathological characteristics of invasive gastric <i>Helicobacter pylori</i> | Human Pathology | 10.1016/j.humpath.2016.09.029 |
| **189** | K. Dzobo, N. Hassen, D. A. Senthebane, N. E. Thomford, A. Rowe, H. Shipanga, A. Wonkam, M. I. Parker, S. Mowla and C. Dandara | 2018 | Chemoresistance to Cancer Treatment: Benzo-α-Pyrene as Friend or Foe? | Molecules | 10.3390/molecules23040930 |
| **190** | B. Ege, T. Dinç, B. D. Yildiz, Z. Balci and H. Bozkaya | 2015 | Utility of Endoscopy for Diagnosis of Barrett in a Non-Western Society: Endoscopic and Histopathologic Correlation | International Surgery | 10.9738/intsurg-d-14-00167.1 |
| **191** | M. Eisa, A. Sandhu, R. Prakash, S. J. Ganocy and R. Fass | 2020 | The Risk of Acute Myocardial Infarction in Patients With Gastroesophageal Reflux Disease | Journal of Neurogastroenterology and Motility | 10.5056/jnm19192 |
| **192** | J. A. Elliott, S. Casey, C. F. Murphy, N. G. Docherty, N. Ravi, P. Beddy, J. V. Reynolds and C. W. le Roux | 2019 | Risk factors for loss of bone mineral density after curative esophagectomy | Archives of Osteoporosis | 10.1007/s11657-018-0556-z |
| **193** | O. Eslami, M. Shahraki, A. Bahari and T. Shahraki | 2017 | Dietary habits and obesity indices in patients with gastro-esophageal reflux disease: a comparative cross-sectional study | Bmc Gastroenterology | 10.1186/s12876-017-0699-1 |
| **194** | A. Etemadi, A. Gandomkar, N. D. Freedman, M. Moghadami, M. R. Fattahi, H. Poustchi, F. Islami, P. Boffetta, S. M. Dawsey, C. C. Abnet and R. Malekzadeh | 2017 | The association between waterpipe smoking and gastroesophageal reflux disease | International Journal of Epidemiology | 10.1093/ije/dyx158 |
| **195** | A. Etemadi, S. Hariri, H. Hassanian-moghaddam, H. Poustchi, G. Roshandel, A. Shayanrad, F. Kamangar, P. Boffetta, P. Brennan, P. I. Dargan, S. M. Dawsey, R. L. Jones, N. D. Freedman, R. Malekzadeh and C. C. Abnet | 2022 | Lead poisoning among asymptomatic individuals with a long-term history of opiate use in Golestan Cohort Study | International Journal of Drug Policy | 10.1016/j.drugpo.2022.103695 |
| **196** | A. Etemadi, F. Kamangar, F. Islami, H. Poustchi, A. Pourshams, P. Brennan, P. Boffetta, R. Malekzadeh, S. M. Dawsey, C. C. Abnet and A. Emadi | 2015 | Mortality and cancer in relation to ABO blood group phenotypes in the Golestan Cohort Study | Bmc Medicine | 10.1186/s12916-014-0237-8 |
| **197** | A. Etemadi, H. Khademi, F. Kamangar, N. D. Freedman, C. C. Abnet, P. Brennan, R. Malekzadeh and T. Golestan Cohort Study | 2017 | Hazards of cigarettes, smokeless tobacco and waterpipe in a Middle Eastern Population: a Cohort Study of 50 000 individuals from Iran | Tobacco Control | 10.1136/tobaccocontrol-2016-053245 |
| **198** | A. Etemadi, H. Poustchi, A. M. Calafat, B. C. Blount, V. R. De Jesús, L. Q. Wang, A. Pourshams, R. Shakeri, M. Inoue-Choi, M. S. Shiels, G. Roshandel, G. Murphy, C. S. Sosnoff, D. Bhandari, J. Feng, B. Y. Xia, Y. S. Wang, L. Meng, F. Kamangar, P. Brennan, P. Boffetta, S. M. Dawsey, C. C. Abnet, R. Malekzadeh and N. D. Freedman | 2020 | Opiate and Tobacco Use and Exposure to Carcinogens and Toxicants in the Golestan Cohort Study | Cancer Epidemiology Biomarkers & Prevention | 10.1158/1055-9965.Epi-19-1212 |
| **199** | A. Etemadi, H. Poustchi, C. M. Chang, B. C. Blount, A. M. Calafat, L. Q. Wang, V. R. De Jesus, A. Pourshams, R. Shakeri, M. S. Shiels, M. Inoue-Choi, B. K. Ambrose, C. H. Christensen, B. G. Wang, G. Murphy, X. Y. Ye, D. Bhandari, J. Feng, B. Y. Xia, C. S. Sosnoff, F. Kamangar, P. Brennan, P. Boffetta, S. M. Dawsey, C. C. Abnet, R. Malekzadeh and N. D. Freedman | 2019 | Urinary Biomarkers of Carcinogenic Exposure among Cigarette, Waterpipe, and Smokeless Tobacco Users and Never Users of Tobacco in the Golestan Cohort Study | Cancer Epidemiology Biomarkers & Prevention | 10.1158/1055-9965.Epi-18-0743 |
| **200** | A. Etemadi, H. Poustchi, C. M. Chang, A. M. Calafat, B. C. Blount, D. Bhandari, L. Q. Wang, G. Roshandel, A. Alexandridis, J. C. Botelho, B. Y. Xia, Y. S. Wang, C. S. Sosnoff, J. Feng, M. Nalini, M. Khoshnia, A. Pourshams, M. Sotoudeh, M. H. Gail, S. M. Dawsey, F. Kamangar, P. Boffetta, P. Brennan, C. C. Abnet, R. Malekzadeh and N. D. Freedman | 2024 | Exposure to polycyclic aromatic hydrocarbons, volatile organic compounds, and tobacco-specific nitrosamines and incidence of esophageal cancer | Jnci-Journal of the National Cancer Institute | 10.1093/jnci/djad218 |
| **201** | A. Etemadi, S. Safiri, S. G. Sepanlou, K. Ikuta, C. Bisignano, R. Shakeri, M. Amani, C. Fitzmaurice, M. R. Nixon, N. Abbasi, H. Abolhassani, S. M. Advani, M. Afarideh, T. Akinyemiju, T. Alam, M. Alikhani, V. Alipour, C. A. Allen, A. Almasi-Hashiani, J. Arabloo, R. Assadi, S. Atique, A. Awasthi, A. Bakhtiari, M. Behzadifar, K. Berhe, N. Bhala, A. Bijani, M. S. Bin Sayeed, T. Bjorge, A. M. Borzì, D. Braithwaite, H. Brenner, G. Carreras, F. Carvalho, C. A. Castañeda-Orjuela, F. Castro, D. T. Chu, V. M. Costa, A. Daryani, D. V. Davitoiu, G. T. Demoz, A. B. Demis, E. Denova-Gutiérrez, S. Dey, M. D. Nasab, S. Djalalinia, M. H. Emamian, M. Farahmand, J. C. Fernandes, F. Fischer, M. Foroutan, M. M. Gad, S. Gallus, G. G. Gebremeskel, G. A. Gedefew, F. Ghaseni-Kebria, G. Gorini, N. Hafezi-Nejad, A. Haj-Mirzaian, J. M. Haro, J. D. Harvey, A. Hasanzadeh, M. Hashemian, H. Y. Hassen, S. I. Hay, H. D. Hidru, M. Hostiuc, M. Househ, O. S. Ilesanmi, M. D. Ilic, K. Innos, F. Islami, S. L. James, E. Jenabi, R. Kalhor, F. Kamangar, A. Kasaeian, A. P. Kengne, Y. S. Khader, R. Khalilov, E. A. Khan, G. Khan, M. Khayamzadeh, M. Khazaee-Pool, S. Khazaei, A. T. Khoja, F. Khosravi Shadmani, Y. J. Kim, J. M. Kocarnik, H. Komaki, A. Koyanagi, V. Kumar, C. La Vecchia, A. D. Lopez, R. Lunevicius, N. Manafi, A. L. Manda, B. Geta, H. Meheretu, G. Mengistu, B. Miazgowski, S. M. Mir, K. A. Mohammad, N. M. G. Mezerji, M. Mohammadian, A. Mohammadian-Hafshejani, R. Mohammadpourhodki, S. Mohammed, F. Mohebi, A. H. Mokdad, L. Monasta, M. Moosazadeh, M. Moossavi, G. Moradi, F. Moradpour, R. Moradzadeh, I. M. Velasquez, A. Mosapour, M. Naderi, G. Naik, F. Najafi, A. Nahvijou, I. Negoi, R. Nikbakhsh, M. Nojomi, A. T. Olagunju, T. O. Olagunju, E. Oren, H. Parsian, C. Piccinelli, A. Pourshams, H. Poustchi, N. Rabiee, A. Radfar, A. Rafiei, M. Rahimi, M. Rahmati, A. M. N. Renzaho, N. Rezaei, A. I. Ribeiro, G. Roshandel, A. M. Saad, S. Saadatagah, H. Salimzadeh, A. M. Samy, J. Sanabria, M. M. S. Milicevic, A. Sarveazad, M. Sawhney, F. Shaahmadi, M. Sekerija, M. A. Shaikh, A. Shamshirian, S. K. S. Malleshappa, J. A. Singh, C. G. Smarandache, M. Soofi, T. Tabuchi, D. B. B. Tadesse, L. Tapak, B. E. Tesfay, E. Traini, B. Tran, K. B. Tran, M. Vacante, A. Vahedian-Azimi, Y. Veisani, K. Vosoughi, I. S. Vujcic, R. Westerman, A. B. Wondmieneh, R. X. Xu, S. Yaya, V. Yazdi-Feyzabadi, Z. Yousefi, B. Yousefi, T. Z. Moghadam, L. Zaki, M. Zamani, M. Zamanian, H. Zandian, A. Zarghi, Z. J. Zhang, M. Naghavi, R. Malekzadeh and G. B. D. S. C. Collaborator | 2020 | The global, regional, and national burden of stomach cancer in 195 countries, 1990-2017: a systematic analysis for the Global Burden of Disease study 2017 | Lancet Gastroenterology & Hepatology | 10.1016/s2468-1253(19)30328-0 |
| **202** | C. J. Ethan, K. K. Mokoena, Y. Yu, K. Shale, Y. M. Fan, J. Rong and F. Liu | 2020 | Association between PM<sub>2.5</sub> and mortality of stomach and colorectal cancer in Xi'an: a time-series study | Environmental Science and Pollution Research | 10.1007/s11356-020-08628-0 |
| **203** | R. Everatt, I. Kuzmickiene, E. Davidaviciene and S. Cicenas | 2017 | Non-pulmonary cancer risk following tuberculosis: a nationwide retrospective cohort study in Lithuania | Infectious Agents and Cancer | 10.1186/s13027-017-0143-8 |
| **204** | C. Y. Fan, W. Y. Huang, C. S. Lin, Y. F. Su, C. H. Lo, C. C. Tsao, M. Y. Liu, C. L. Lin and C. H. Kao | 2017 | Risk of second primary malignancies among patients with prostate cancer: A population-based cohort study | Plos One | 10.1371/journal.pone.0175217 |
| **205** | J. H. Fan, W. Y. Sun, H. Yang, X. K. Wang, C. C. Abnet and Y. L. Qiao | 2023 | Short-term and long-term effect of nutrition intervention in the Linxian Dysplasia Nutrition Intervention Trial and the reason for disappearance of the intervention effect: A cohort study | Cancer | 10.1002/cncr.34761 |
| **206** | Y. G. Fan, Y. Jiang, L. Gong, Y. Wang, Z. Su, X. B. Li, H. Wu, H. L. Pan, J. Wang, Z. W. Meng, Q. H. Zhou and Y. L. Qiao | 2023 | Epidemiological and demographic drivers of lung cancer mortality from 1990 to 2019: results from the global burden of disease study 2019 | Frontiers in Public Health | 10.3389/fpubh.2023.1054200 |
| **207** | Z. Y. Fan, Y. X. Li, J. Wei, G. B. Chen, R. Wang, T. T. Liu, Z. Q. Lv, S. L. Huang, H. Sun and Y. W. Liu | 2023 | Long-term exposure to fine particulate matter and site-specific cancer mortality: A difference-in-differences analysis in Jiangsu province, China | Environmental Research | 10.1016/j.envres.2023.115405 |
| **208** | C. Fang, Q. Huang, L. Lu, J. Shi, Q. Sun, G. F. Xu, J. Gold, H. Mashimo and X. P. Zou | 2015 | Risk factors of early proximal gastric carcinoma in Chinese diagnosed using WHO criteria | Journal of Digestive Diseases | 10.1111/1751-2980.12240 |
| **209** | P. Fang, W. Jiang, R. Davuluri, C. Xu, S. Krishnan, R. Mohan, A. C. Koong, C. C. Hsu and S. H. Lin | 2018 | High lymphocyte count during neoadjuvant chemoradiotherapy is associated with improved pathologic complete response in esophageal cancer | Radiotherapy and Oncology | 10.1016/j.radonc.2018.02.025 |
| **210** | S. Faramarzi, B. Kiani, S. Faramarzi and N. Firouraghi | 2024 | Cancer patterns in Iran: a gender-specific spatial modelling of cancer incidence during 2014-2017 | Bmc Cancer | 10.1186/s12885-024-11940-4 |
| **211** | E. H. Farbu, M. Skandfer, C. Nielsen, T. Brenn, A. Stubhaug and A. C. Höper | 2019 | Working in a cold environment, feeling cold at work and chronic pain: a cross-sectional analysis of the Tromso Study | Bmj Open | 10.1136/bmjopen-2019-031248 |
| **212** | M. Fasullo, A. Sreenivasen, E. Holzwanger, C. Lavender, M. Patel, T. Shah, P. Mutha, R. F. Yacavone, K. Sultan, A. J. Trindade and G. Smallfield | 2021 | Co-existing inflammatory bowel disease and Barrett's esophagus is associated with esophageal dysplasia: a propensity score-matched cohort | Endoscopy International Open | 10.1055/a-1526-0507 |
| **213** | M. Fattouh, G. Y. Chang, T. J. Ow, K. Shifteh, G. Rosenblatt, V. M. Patel, R. V. Smith, M. B. Prystowsky and N. F. Schlecht | 2019 | Association between pretreatment obesity, sarcopenia, and survival in patients with head and neck cancer | Head and Neck-Journal for the Sciences and Specialties of the Head and Neck | 10.1002/hed.25420 |
| **214** | X. Feng, Z. L. Hua, Q. Zhou, A. W. Shi, T. Q. Song, D. F. Qian, R. Chen, G. Q. Wang, W. Q. Wei, J. Y. Zhou, J. J. Wang, G. Shao and X. Wang | 2021 | Prevalence and coprevalence of modifiable risk factors for upper digestive tract cancer among residents aged 40-69 years in Yangzhong city, China: a cross-sectional study | Bmj Open | 10.1136/bmjopen-2020-042006 |
| **215** | X. Feng, J. H. Zhu, Z. L. Hua, Q. P. Shi, J. Y. Zhou and P. F. Luo | 2023 | The prevalence and determinant of overweight and obesity among residents aged 40-69 years in high-risk regions for upper gastrointestinal cancer in southeast China | Scientific Reports | 10.1038/s41598-023-35477-x |
| **216** | A. Ferro, S. Morais, C. Pelucchi, N. Aragonés, M. Kogevinas, L. López-Carrillo, R. Malekzadeh, S. Tsugane, G. S. Hamada, A. Hidaka, R. U. Hernández-Ramírez, M. López-Cervantes, D. Zaridze, D. Maximovitch, F. Pourfarzi, Z. F. Zhang, G. P. Yu, M. Pakseresht, W. M. Ye, A. Plymoth, M. Leja, E. Gasenko, M. H. Derakhshan, E. Negri, C. La Vecchia, B. Peleteiro and N. Lunet | 2019 | Smoking and <i>Helicobacter pylori</i> infection: an individual participant pooled analysis (Stomach Cancer Pooling- StoP Project) | European Journal of Cancer Prevention | 10.1097/cej.0000000000000471 |
| **217** | A. Ferro, S. Morais, C. Pelucchi, T. Dierssen-Sotos, V. Martín, L. López-Carrillo, R. Malekzadeh, S. Tsugane, G. S. Hamada, A. Hidaka, R. U. Hernández-Ramírez, M. López-Cervantes, D. Zaridze, D. Maximovitch, F. Pourfarzi, Z. F. Zhang, G. P. Yu, M. Pakseresht, W. M. Ye, A. Plymoth, M. Leja, E. Gasenko, M. H. Derakhshan, E. Negri, C. La Vecchia, B. Peleteiro and N. Lunet | 2019 | Sex differences in the prevalence of Helicobacter pylori infection: an individual participant data pooled analysis (StoP Project) | European Journal of Gastroenterology & Hepatology | 10.1097/meg.0000000000001389 |
| **218** | A. Ferro, S. Morais, M. Rota, C. Pelucchi, P. Bertuccio, R. Bonzi, C. Galeone, Z. F. Zhang, K. Matsuo, H. Ito, J. F. Hu, K. C. Johnson, G. P. Yu, D. Palli, M. Ferraroni, J. Muscat, R. Malekzadeh, W. M. Ye, H. Song, D. Zaridze, D. Maximovitch, N. Aragonés, G. Castaño-Vinyals, J. Vioque, E. M. Navarrete-Muñoz, M. Pakseresht, F. Pourfarzi, A. Wolk, N. Orsini, A. Bellavia, N. Håkansson, L. N. Mu, R. Pastorino, R. C. Kurtz, M. H. Derakhshan, A. Lagiou, P. Lagiou, P. Boffetta, S. Boccia, E. Negri, C. La Vecchia, B. Peleteiro and N. Lunet | 2018 | Tobacco smoking and gastric cancer: meta-analyses of published data versus pooled analyses of individual participant data (StoP Project) | European Journal of Cancer Prevention | 10.1097/cej.0000000000000401 |
| **219** | A. Ferro, S. Morais, M. Rota, C. Pelucchi, P. Bertuccio, R. Bonzi, C. Galeone, Z. F. Zhang, K. Matsuo, H. Ito, J. F. Hu, K. C. Johnson, G. P. Yu, D. Palli, M. Ferraroni, J. Muscat, R. Malekzadeh, W. M. Ye, H. Song, D. Zaridze, D. Maximovitch, N. F. de Larrea, M. Kogevinas, J. Vioque, E. M. Navarrete-Muñoz, M. Pakseresht, F. Pourfarzi, A. Wolk, N. Orsini, A. Bellavia, N. Håkansson, L. N. Mu, R. Pastorino, R. C. Kurtz, M. H. Derakhshan, A. Lagiou, P. Lagiou, P. Boffetta, S. Boccia, E. Negri, C. La Vecchia, B. Peleteiro and N. Lunet | 2018 | Alcohol intake and gastric cancer: Meta-analyses of published data versus individual participant data pooled analyses (StoP Project) | Cancer Epidemiology | 10.1016/j.canep.2018.04.009 |
| **220** | A. Ferro, V. Rosato, M. Rota, A. R. Costa, S. Morais, C. Pelucchi, K. C. Johnson, J. F. Hu, D. Palli, M. Ferraroni, Z. F. Zhang, R. Bonzi, G. P. Yu, B. Peleteiro, L. López-Carrillo, S. Tsugane, G. S. Hamada, A. Hidaka, D. Zaridze, D. Maximovitch, J. Vioque, E. M. Navarrete-Munoz, N. Aragonés, V. Martín, R. U. Hernández-Ramírez, P. Bertuccio, M. H. Ward, R. Malekzadeh, F. Pourfarzi, L. N. Mu, M. López-Cervantes, R. Persiani, R. C. Kurtz, A. Lagiou, P. Lagiou, P. Boffetta, S. Boccia, E. Negri, M. C. Camargo, M. P. Curado, C. La Vecchia and N. Lunet | 2020 | Meat intake and risk of gastric cancer in the Stomach cancer Pooling (StoP) project | International Journal of Cancer | 10.1002/ijc.32707 |
| **221** | E. Finocchio, F. Locatelli, F. Sanna, R. Vesentini, P. Marchetti, G. Spiteri, L. Antonicelli, S. Battaglia, R. Bono, A. G. Corsico, M. Ferrari, N. Murgia, P. Pirina, M. Olivieri and G. Verlato | 2021 | Gastritis and gastroesophageal reflux disease are strongly associated with non-allergic nasal disorders | Bmc Pulmonary Medicine | 10.1186/s12890-020-01364-8 |
| **222** | C. Fitzmaurice, C. Allen, R. M. Barber, L. Barregard, Z. A. Bhutta, H. Brenner, D. J. Dicker, O. Chimed-Orchir, R. Dandona, L. Dandona, T. Fleming, M. H. Forouzanfar, J. Hancock, R. J. Hay, R. Hunter-Merrill, C. Huynh, H. D. Hosgood, C. O. Johnson, J. B. Jonas, J. Khubchandani, G. A. Kumar, M. Kutz, Q. Lan, H. J. Larson, X. F. Liang, S. S. Lim, A. D. Lopez, M. F. MacIntyre, L. Marczak, N. Marquez, A. H. Mokdad, C. Pinho, F. Pourmalek, J. A. Salomon, J. R. Sanabria, L. Sandar, B. Sartorius, S. M. Schwartz, K. A. Shackelford, K. Shibuya, J. Stanaway, C. Steiner, J. D. Sun, K. Takahashi, S. E. Vollset, T. Vos, J. A. Wagner, H. D. Wang, R. Westerman, H. Zeeb, L. Zoeckler, F. Abd-Allah, M. B. Ahmed, S. Alabed, N. K. Alam, S. F. Aldhahri, G. Alem, M. A. Alemayohu, R. Ali, R. Al-Raddadi, A. Amare, Y. Amoako, A. Artaman, H. Asayesh, N. Atnafu, A. Awasthi, H. B. Saleem, A. Barac, N. Bedi, I. Bensenor, A. Berhane, E. Bemabé, B. Betsu, A. Binagwaho, D. Boneya, I. Campos-Nonato, C. Castañeda-Orjuela, F. Catalá-López, P. Chiang, C. Chibueze, A. Chitheer, J. Y. Choi, B. Cowie, S. Damtew, J. das Neves, S. Dey, S. Dharmaratne, P. Dhillon, E. Ding, T. Driscoll, D. Ekwueme, A. Y. Endries, M. Farvid, F. Farzadfar, J. Fernandes, F. Fischer, T. T. Ghiwot, A. Gebru, S. Gopalani, A. Hailu, M. Horino, N. Horita, A. Husseini, I. Huybrechts, M. Inoue, F. Islami, M. Jakovljevic, S. James, M. Javanbakht, S. H. Jee, A. Kasaeian, M. S. Kedir, Y. S. Khader, Y. H. Khang, D. Kim, J. Leigh, S. Linn, R. Lunevicius, H. M. Abd El Razek, R. Malekzadeh, D. C. Malta, W. Marcenes, D. Markos, Y. A. Melaku, K. G. Meles, W. Mendoza, D. T. Mengiste, T. J. Meretoja, T. R. Miller, K. A. Mohammad, A. Mohammadi, S. Mohammed, M. Moradi-Lakeh, G. Nagel, D. Nand, Q. Le Nguyen, S. Nolte, F. A. Ogbo, K. E. Oladimeji, E. Oren, M. Pa, E. K. Park, D. M. Pereira, D. Plass, M. Qorbani, A. Radfar, A. Rafay, M. Rahman, S. M. Rana, K. Soreide, M. Satpathy, M. Sawhney, S. G. Sepanlou, M. A. Shaikh, J. She, I. Shiue, H. R. Shore, M. G. Shrime, S. So, S. Soneji, V. Stathopoulou, K. Stroumpoulis, M. B. Sufiyan, B. L. Sykes, R. Tabares-Seisdedos, F. Tadese, B. A. Tedla, G. A. Tessema, J. S. Thakur, B. X. Tran, K. N. Ukwaja, B. S. C. Uzochukwu, V. V. Vlassov, E. Weiderpass, M. W. Terefe, H. G. Yebyo, H. H. Yimam, N. Yonemoto, M. Z. Younis, C. H. Yu, Z. Zaidi, M. E. Zaki, Z. M. Zenebe, C. J. L. Murray, M. Naghavi and C. Global Bourden Disease Cancer | 2017 | Global, Regional, and National Cancer Incidence, Mortality, Years of Life Lost, Years Lived With Disability, and Disability-Adjusted Life-years for 32 Cancer Groups, 1990 to 2015 A Systematic Analysis for the Global Burden of Disease Study | Jama Oncology | 10.1001/jamaoncol.2016.5688 |
| **223** | S. Flashner, C. Martin, N. Matsuura, M. Shimonosono, Y. Tomita, M. Morimoto, O. Okolo, V. X. Yu, A. S. Parikh, A. J. P. Klein-Szanto, K. Yan, J. T. Gabre, C. Lu, F. Momen-Heravi, A. K. Rustgi and H. Nakagawa | 2022 | Modeling Oral-Esophageal Squamous Cell Carcinoma in 3D Organoids | Jove-Journal of Visualized Experiments | 10.3791/64676 |
| **224** | L. Flores-Luna, M. M. Bravo, E. Kasamatsu, E. C. L. Ponce, T. Martinez, J. Torres, M. Camorlinga-Ponce and I. Kato | 2020 | Risk factors for gastric precancerous and cancers lesions in Latin American counties with difference gastric cancer risk | Cancer Epidemiology | 10.1016/j.canep.2019.101630 |
| **225** | C. Folgueira, S. Barja-Fernandez, L. Prado, O. Al-Massadi, C. Castelao, V. Pena-Leon, P. Gonzalez-Saenz, J. Baltar, I. Baamonde, R. Leis, C. Dieguez, U. Pagotto, F. F. Casanueva, S. A. Tovar, R. Nogueiras and L. M. Seoane | 2017 | Pharmacological inhibition of cannabinoid receptor 1 stimulates gastric release of nesfatin-1 <i>via</i> the mTOR pathway | World Journal of Gastroenterology | 10.3748/wjg.v23.i35.6403 |
| **226** | K. Fukai, N. Kojimahara, K. Hoshi, A. Toyota and M. Tatemichi | 2020 | Combined effects of occupational exposure to hazardous operations and lifestyle-related factors on cancer incidence | Cancer Science | 10.1111/cas.14663 |
| **227** | T. Fukuchi, K. Hirasawa, C. Sato, M. Makazu, H. Kaneko, R. Kobayashi, M. Nishio, R. Ikeda, A. Sawada, Y. Ozeki, M. Sugimori, Y. Inayama, Y. Tateishi and S. Maeda | 2021 | Potential roles of gastroesophageal reflux in patients with superficial esophageal squamous cell carcinoma without major causative risk factors | Journal of Gastroenterology | 10.1007/s00535-021-01815-x |
| **228** | S. Fukunaga, M. Mukasa, T. Nakane, D. Nakano, T. Tsutsumi, T. Chou, H. Tanaka, D. Hayashi, S. Minami, A. Ohuchi, T. Nagata, K. Takaki, H. Takaki, I. Miyajima, R. Nouno, T. Araki, T. Morita, T. Torimura, Y. Okabe and T. Kawaguchi | 2024 | Impact of non-obese metabolic dysfunction-associated fatty liver disease on risk factors for the recurrence of esophageal squamous cell carcinoma treated with endoscopic submucosal dissection: A multicenter study | Hepatology Research | 10.1111/hepr.13973 |
| **229** | A. Gado, B. Ebeid, A. Abdelmohsen and A. Axon | 2015 | Prevalence of reflux esophagitis among patients undergoing endoscopy in a secondary referral hospital in Giza, Egypt | Alexandria Journal of Medicine | 10.1016/j.ajme.2013.09.002 |
| **230** | M. S. Gallaway, S. J. Henley, C. B. Steele, B. Momin, C. C. Thomas, A. Jamal, K. F. Trivers, S. D. Singh and S. L. Stewart | 2018 | Surveillance for Cancers Associated with Tobacco Use - United States, 2010-2014 | Mmwr Surveillance Summaries | 10.15585/mmwr.ss6712a1 |
| **231** | T. Y. Gao, Y. T. Tao, H. Y. Li, X. Liu, Y. T. Ma, H. J. Li, C. Y. Xian-Yu, N. J. Deng, W. D. Leng, J. Luo and C. Zhang | 2024 | Cancer burden and risk in the Chinese population aged 55 years and above: A systematic analysis and comparison with the USA and Western Europe | Journal of Global Health | 10.7189/jogh.14.04014 |
| **232** | Y. Gao, X. Y. Wu, Y. H. Li, Y. F. Li, Q. Y. Zhou, Q. Q. Wang, C. Y. Wei, D. L. Shi, C. Y. Xie and H. L. Pan | 2020 | The Predictive Value of MLR for Radiation Pneumonia During Radiotherapy of Thoracic Tumor Patients | Cancer Management and Research | 10.2147/cmar.S268964 |
| **233** | S. M. Gapstur, V. Bouvard, S. T. Nethan, J. L. Freudenheim, C. C. Abnet, D. R. English, J. Rehm, S. Balbo, P. Buykx, D. Crabb, D. I. Conway, F. Islami, D. W. Lachenmeier, K. A. McGlynn, M. Salaspuro, N. Sawada, M. B. Terry, T. Toporcov and B. Lauby-Secretan | 2023 | The IARC Perspective on Alcohol Reduction or Cessation and Cancer Risk | New England Journal of Medicine | 10.1056/NEJMsr2306723 |
| **234** | A. Garant, G. Spears, D. Routman, T. Whitaker, Z. X. Liao, W. Harmsen, A. Liu, M. Haddock, C. Hallemeier, S. Lin and K. Merrell | 2021 | A Multi-Institutional Analysis of Radiation Dosimetric Predictors of Toxicity After Trimodality Therapy for Esophageal Cancer | Practical Radiation Oncology | 10.1016/j.prro.2021.01.004 |
| **235** | M. Garau, C. Musetti, R. Alonso and E. Barrios | 2019 | Trends in cancer incidence in Uruguay: 2002-2015 | Colombia Medica | 10.25100/cm.v50i4.4212 |
| **236** | M. A. García-González, L. Bujanda, E. Quintero, S. Santolaria, R. Benito, M. Strunk, F. Sopeña, C. Thomson, A. Pérez-Aisa, D. Nicolás-Pérez, E. Hijona, P. Carrera-Lasfuentes, E. Piazuelo, P. Jiménez, J. Espinel, R. Campo, M. Manzano, F. Geijo, M. Pellise, M. Zaballa, F. González-Huix, J. Espinós, L. Titó, L. Barranco, R. Pazo-Cid and A. Lanas | 2015 | Association of <i>PSCA</i> rs2294008 gene variants with poor prognosis and increased susceptibility to gastric cancer and decreased risk of duodenal ulcer disease | International Journal of Cancer | 10.1002/ijc.29500 |
| **237** | J. A. García-Lavandeira, A. Ruano-Ravina, M. Torres-Durán, I. Parente-Lamelas, M. Provencio, L. Varela-Lema, A. Fernández-Villar, M. Piñeiro, J. M. Barros-Dios and M. Pérez-Ríos | 2022 | Fruits and Vegetables and Lung Cancer Risk in Never Smokers. A Multicentric and Pooled Case-Control Study | Nutrition and Cancer-an International Journal | 10.1080/01635581.2021.1918732 |
| **238** | N. Garg, C. Stoehr, Y. S. Zhao, H. Rojas and C. T. Hsueh | 2017 | Metastatic squamous cell carcinoma of colon from esophageal cancer | Experimental Hematology & Oncology | 10.1186/s40164-017-0069-2 |
| **239** | R. Gaspar, S. Rodrigues, M. Silva, P. Costa-Moreira, R. Morais, P. Andrade, H. Cardoso, A. Albuquerque, R. Liberal and G. Macedo | 2019 | Predictive models of mortality and hospital readmission of patients with decompensated liver cirrhosis | Digestive and Liver Disease | 10.1016/j.dld.2019.03.016 |
| **240** | V. Gatzinsky, G. Wennergren, L. Jönsson, L. Ekerljung, B. Houltz, S. Redfors, U. Sillén and P. Gustafsson | 2014 | Impaired peripheral airway function in adults following repair of esophageal atresia | Journal of Pediatric Surgery | 10.1016/j.jpedsurg.2013.12.027 |
| **241** | V. Gehlot, A. Mathur, K. Das, S. Mahant and R. Das | 2024 | No Association between <i>Helicobacter pylori</i> and Gastroesophageal Reflux Disease: A Comprehensive Risk Factor Analysis in North Indian Patients | Biomedical and Biotechnology Research Journal | 10.4103/bbrj.bbrj_157_24 |
| **242** | F. H. Geng, M. H. Liu, J. H. Chen, Y. L. Ge, S. X. Wei, F. Y. Li, C. S. Yang, J. W. Sun, L. J. Gou, J. Y. Zhang, S. K. Tang, Y. Wan, J. Y. Yang and J. Zhang | 2023 | Clinical characteristics of second primary malignancies among first primary malignancy survivors: A single-center study, 2005-2020 | Oncology Letters | 10.3892/ol.2022.13610 |
| **243** | S. K. George, B. Tlou, S. Ponnusamy and D. P. Naidoo | 2020 | Does acid reflux precipitate ischaemia in subjects with acute coronary syndrome? | Cardiovascular Journal of Africa | 10.5830/cvja-2019-048 |
| **244** | D. Georgescu, O. E. Ancusa, D. Azoulay, A. Lascu, I. Ionita, D. Calamar-Popovici, M. Ionita, C. I. Rosca, G. M. Breaza, D. Reisz and D. Lighezan | 2023 | Portal Vein Thrombosis in Patients with Liver Cirrhosis: What Went Wrong? | International Journal of General Medicine | 10.2147/ijgm.S413438 |
| **245** | E. Ghaderi, K. Hassanzadeh, K. Rahmani, G. Moradi, N. Esmailnasab, D. Roshani and A. Azadnia | 2020 | Prevalence of self-medication and its associated factors: a case study of Kurdistan province | International Journal of Human Rights in Health Care | 10.1108/ijhrh-09-2019-0075 |
| **246** | K. Ghanadi and K. Anbari | 2018 | Risk Factors of Peptic Ulcer Disease in Khorramabad city, Southwest of Iran: A Case Control Study | World Family Medicine | 10.5742/mewfm.2018.93215 |
| **247** | K. Ghanadi, K. Anbari, B. Khodadadi and M. S. Farahani | 2018 | Correlation between Histopathological Findings and Endoscopy in Esophageal Cance: Results in Khorramabad, Iran, Western Iran | Bangladesh Journal of Medical Science | 10.3329/bjms.v17i4.38325 |
| **248** | P. Gharahkhani, J. Tung, D. Hinds, A. Mishra, T. L. Vaughan, D. C. Whiteman, S. MacGregor, E. Barrett's and B. S. Investigators | 2016 | Chronic gastroesophageal reflux disease shares genetic background with esophageal adenocarcinoma and Barrett's esophagus | Human Molecular Genetics | 10.1093/hmg/ddv512 |
| **249** | H. Ghaznavi, F. Allaveisi and F. Taghizadeh-Hesary | 2022 | Baseline cardiac risk profile determines radiation-induced cardiac toxicity in patients with mid-lower esophageal cancer | Journal of Radiotherapy in Practice | 10.1017/s146039692200036x |
| **250** | Y. T. Ghebre and G. Raghu | 2016 | Idiopathic Pulmonary Fibrosis: Novel Concepts of Proton Pump Inhibitors as Antifibrotic Drugs | American Journal of Respiratory and Critical Care Medicine | 10.1164/rccm.201512-2316PP |
| **251** | Z. Ghorbani, A. Hekmatdoost, H. E. Zinab, S. Farrokhzad, R. Rahimi, R. Malekzadeh and A. Pourshams | 2015 | Dietary food groups intake and cooking methods associations with pancreatic cancer: A case-control study | Indian Journal of Gastroenterology | 10.1007/s12664-015-0573-4 |
| **252** | P. Ghosh, S. Mandal, S. M. Mustafi and N. Murmu | 2021 | Clinicopathological Characteristics and Incidence of Gastric Cancer in Eastern India: A Retrospective Study | Journal of Gastrointestinal Cancer | 10.1007/s12029-020-00478-w |
| **253** | S. Ghosh, B. Bankura, S. Ghosh, M. L. Saha, A. K. Pattanayak, S. Ghatak, M. Guha, S. K. Nachimuthu, C. K. Panda, S. Maji, S. Chakraborty, B. Maity and M. Das | 2017 | Polymorphisms in <i>ADH1B</i> and <i>ALDH2</i> genes associated with the increased risk of gastric cancer in West Bengal, India | Bmc Cancer | 10.1186/s12885-017-3713-7 |
| **254** | S. Ghosh, S. Ghosh, B. Bankura, M. L. Saha, S. Maji, S. Ghatak, A. K. Pattanayak, S. Sadhukhan, M. Guha, S. K. Nachimuthu, C. K. Panda, B. Maity and M. Das | 2016 | Association of DNA repair and xenobiotic pathway gene polymorphisms with genetic susceptibility to gastric cancer patients in West Bengal, India | Tumor Biology | 10.1007/s13277-015-4780-5 |
| **255** | G. F. Gil, J. A. Anderson, A. Aravkin, K. Bhangdia, S. Carr, X. C. Dai, L. S. Flor, S. I. Hay, M. J. Malloy, S. A. McLaughlin, E. C. Mullany, C. J. L. Murray, E. M. O'Connell, C. Okereke, R. J. D. Sorensen, J. Whisnant, P. Zheng and E. Gakidou | 2024 | Health effects associated with chewing tobacco: a Burden of Proof study | Nature Communications | 10.1038/s41467-024-45074-9 |
| **256** | J. Gilhodes, A. Belot, A. M. Bouvier, L. Remontet, P. Delafosse, K. Ligier and A. Rogel | 2015 | Incidence of major smoking-related cancers: Trends among adults aged 20-44 in France from 1982 to 2012 | Cancer Epidemiology | 10.1016/j.canep.2015.07.001 |
| **257** | L. Giraldi, J. Stojanovic, D. Arzani, R. Persiani, J. F. Hu, K. C. Johnson, Z. F. Zhang, M. Ferraroni, D. Palli, G. P. Yu, C. La Vecchia, C. Pelucchi, N. Lunet, A. Ferro, R. Malekzadeh, J. Muscat, D. Zaridze, D. Maximovich, N. Aragones, V. Martin, J. Vioque, E. M. Navarrete-Munoz, M. Pakseresht, E. Negri, M. Rota, F. Pourfarzi, L. N. Mu, R. C. Kurtz, A. Lagiou, P. Lagiou, R. Pastorino and S. Boccia | 2023 | Adult height and risk of gastric cancer: a pooled analysis within the Stomach cancer Pooling Project | European Journal of Cancer Prevention | 10.1097/cej.0000000000000613 |
| **258** | F. Giusti, C. Martos, M. Bettio, R. N. Carvalho, M. Zorzi, S. Guzzinati and M. Rugge | 2024 | Geographical and temporal differences in gastric and oesophageal cancer registration by subsite and morphology in Europe | Frontiers in Oncology | 10.3389/fonc.2024.1250107 |
| **259** | E. Gkogkou, G. Barnasas, K. Vougas and I. P. Trougakos | 2020 | Expression profiling meta-analysis of ACE2 and TMPRSS2, the putative anti-inflammatory receptor and priming protease of SARS-CoV-2 in human cells, and identification of putative modulators | Redox Biology | 10.1016/j.redox.2020.101615 |
| **260** | A. Golozar, A. Etemadi, F. Kamangar, A. F. Malekshah, F. Islami, D. Nasrollahzadeh, B. Abedi-Ardekani, M. Khoshnia, A. Pourshams, S. Semnani, H. A. Marjani, R. Shakeri, M. Sotoudeh, P. Brennan, P. Taylor, P. Boffetta, C. Abnet, S. Dawsey and R. Malekzadeh | 2016 | Food preparation methods, drinking water source, and esophageal squamous cell carcinoma in the high-risk area of Golestan, Northeast Iran | European Journal of Cancer Prevention | 10.1097/cej.0000000000000156 |
| **261** | A. Gonçalves, D. Simas, P. Gomes, S. Barbeiro, I. Cotrim and H. Vasconcelos | 2024 | Unveiling the complex nexus: dermatomyositis and esophageal adenocarcinoma-a case report | Annals of Esophagus | 10.21037/aoe-23-23 |
| **262** | H. Goto, T. Oshikiri, T. Kato, R. Sawada, H. Harada, N. Urakawa, H. Hasegawa, S. Kanaji, K. Yamashita, T. Matsuda and Y. Kakeji | 2022 | Short- and long-term outcomes of thoracoscopic esophagectomy in the prone position for esophageal squamous cell carcinoma in patients with obstructive ventilatory disorder: a propensity score-matched study | Surgical Endoscopy and Other Interventional Techniques | 10.1007/s00464-022-09309-4 |
| **263** | H. Goto, T. Oshikiri, T. Kato, R. Sawada, H. Harada, N. Urakawa, H. Hasegawa, S. Kanaji, K. Yamashita, T. Matsuda and Y. Kakeji | 2023 | The Influence of Preoperative Smoking Status on Postoperative Complications and Long-Term Outcome Following Thoracoscopic Esophagectomy in Prone Position for Esophageal Carcinoma | Annals of Surgical Oncology | 10.1245/s10434-022-12898-y |
| **264** | L. M. Grande, F. A. M. Herbella, A. M. Bigatao, H. Abrao, J. R. Jardim and M. G. Patti | 2016 | Pathophysiology of Gastroesophageal Reflux in Patients with Chronic Pulmonary Obstructive Disease Is Linked to an Increased Transdiaphragmatic Pressure Gradient and not to a Defective Esophagogastric Barrier | Journal of Gastrointestinal Surgery | 10.1007/s11605-015-2955-4 |
| **265** | W. B. Grant | 2024 | Cancer Incidence Rates in the US in 2016-2020 with Respect to Solar UVB Doses, Diabetes and Obesity Prevalence, Lung Cancer Incidence Rates, and Alcohol Consumption: An Ecological Study | Nutrients | 10.3390/nu16101450 |
| **266** | T. Grantham, R. Ramachandran, S. Parvataneni, D. Budh, S. Gollapalli and V. Gaduputi | 2023 | Epidemiology of Gastric Cancer: Global Trends, Risk Factors and Premalignant Conditions | Journal of Community Hospital Internal Medicine Perspectives | 10.55729/2000-9666.1252 |
| **267** | A. Gressier, G. Gourier, J. P. Metges, J. D. Dewitte, B. Loddé and D. Lucas | 2022 | Occupational Exposures and Esophageal Cancer: Prog Study | International Journal of Environmental Research and Public Health | 10.3390/ijerph19169782 |
| **268** | X. Grevers, Y. B. Ruan, A. E. Poirier, S. D. Walter, P. J. Villeneuve, C. M. Friedenreich, D. R. Brenner, E. Franco, W. King, P. Demers, P. De, L. Smith, E. Holmes, D. O'Sullivan, K. Volesky, Z. El-Masri, R. Nuttall, M. El-Zein, T. Narain, P. Gogna and P. S. T. Com | 2019 | Estimates of the current and future burden of cancer attributable to alcohol consumption in Canada | Preventive Medicine | 10.1016/j.ypmed.2019.03.020 |
| **269** | L. Guadagnoli, H. Mashimo and W. K. Lo | 2023 | Assessment of Post-traumatic Stress Disorder Among Objective Esophageal Motility and Reflux Phenotypes in Symptomatic Veterans | Journal of Clinical Psychology in Medical Settings | 10.1007/s10880-022-09920-6 |
| **270** | O. Gunduz, C. Bakar, C. Simsek, A. Baba, A. Elci, H. Gurleyuk, M. Mutlu and A. Cakir | 2017 | The Health Risk Associated with Chronic Diseases in Villages with High Arsenic Levels in Drinking Water Supplies | Exposure and Health | 10.1007/s12403-016-0238-2 |
| **271** | L. W. Guo, S. K. Zhang, S. Z. Liu, L. Y. Zheng, Q. Chen, X. Q. Cao, X. B. Sun, Y. L. Qiao and J. G. Zhang | 2019 | Determinants of participation and detection rate of upper gastrointestinal cancer from population-based screening program in China | Cancer Medicine | 10.1002/cam4.2578 |
| **272** | Q. H. Guo, H. Lu, J. Wang, Z. F. Chen, Y. P. Wang, R. Ji, Q. Li, Y. Zhao, H. L. Zhang and Y. N. Zhou | 2016 | Association between TAP1 gene polymorphism and esophageal cancer in a Han Gansu population | International Journal of Clinical and Experimental Medicine |  |
| **273** | W. Guo, H. L. Xiao, Z. Ma, H. J. Liu, Y. J. Wang, L. Y. Mei, X. H. Liu, Y. G. Jiang and R. W. Wang | 2014 | Should Stage T2 Esophageal Squamous Cell Carcinoma Be Subclassified? | Annals of Surgical Oncology | 10.1245/s10434-014-3636-3 |
| **274** | X. K. Guo, W. Q. Ke, X. Yang, X. Y. Zhao and M. Z. Li | 2023 | Association of DLT versus SLT with postoperative pneumonia during esophagectomy in China: a retrospective comparison study | Bmc Anesthesiology | 10.1186/s12871-023-02252-4 |
| **275** | Y. B. Guo, Y. W. Liu, H. J. Yang, N. T. Dai, F. Y. Zhou, H. Yang, W. Sun, J. Y. Kong, X. Yuan and S. G. Gao | 2021 | RETRACTED: Associations of <i>Porphyromonas gingivalis</i> Infection and Low Beclin1 Expression With Clinicopathological Parameters and Survival of Esophageal Squamous Cell Carcinoma Patients (Retracted Article) | Pathology & Oncology Research | 10.3389/pore.2021.1609976 |
| **276** | D. R. Gupta, Y. Liu, R. J. Jiang, S. Walid, K. Higgins, J. Landry, M. McDonald, F. F. Willingham, B. F. El-Rayes and N. F. Saba | 2019 | Racial Disparities, Outcomes, and Surgical Utilization among Hispanics with Esophageal Cancer: A Surveillance, Epidemiology, and End Results Program Database Analysis | Oncology | 10.1159/000499716 |
| **277** | E. Ha and J. H. Bae | 2018 | Zinc transporter <i>SLC39A11</i> polymorphisms are associated with chronic gastritis in the Korean population: the possible effect on spicy food intake | Nutrition Research | 10.1016/j.nutres.2018.04.014 |
| **278** | Y. B. Hadi, A. A. Khan, S. F. Z. Naqvi and J. T. Kupec | 2020 | Independent association of obstructive sleep apnea with Barrett's esophagus | Journal of Gastroenterology and Hepatology | 10.1111/jgh.14779 |
| **279** | M. Hadji, H. Rashidian, M. Marzban, M. Gholipour, A. Naghibzadeh-Tahami, E. Mohebbi, E. Ebrahimi, B. Hosseini, A. A. Haghdoost, A. Rezaianzadeh, A. Rahimi-Movaghar, A. Moradi, M. S. Seyyedsalehi, R. Shirkoohi, H. Poustchi, S. Eghtesad, F. Najafi, R. Safari-Faramani, R. Alizadeh-Navaei, A. R. A. Moghadam, M. Bakhshi, A. Nejatizadeh, M. Mahmudi, S. Shahid-Sales, S. Ahmadi-Simab, O. Nabavian, P. Boffetta, E. Pukkala, E. Weiderpass, F. Kamangar and K. Zendehdel | 2021 | The Iranian Study of Opium and Cancer (IROPICAN): Rationale, Design, and Initial Findings | Archives of Iranian Medicine | 10.34172/aim.2021.27 |
| **280** | M. B. Hadley, M. Nalini, S. Adhikari, J. Szymonifka, A. Etemadi, F. Kamangar, M. Khoshnia, T. McChane, A. Pourshams, H. Poustchi, S. G. Sepanlou, C. Abnet, N. D. Freedman, P. Boffetta, R. Malekzadeh and R. Vedanthan | 2022 | Spatial environmental factors predict cardiovascular and all-cause mortality: Results of the SPACE study | Plos One | 10.1371/journal.pone.0269650 |
| **281** | H. Hagström, J. Höijer, H. U. Marschall, C. Williamson, M. A. Heneghan, R. H. Westbrook, J. F. Ludvigsson and O. Stephansson | 2018 | Outcomes of Pregnancy in Mothers With Cirrhosis: A National Population-Based Cohort Study of 1.3 Million Pregnancies | Hepatology Communications | 10.1002/hep4.1255 |
| **282** | M. B. Haider, B. Basida and J. Kaur | 2023 | Major depressive disorders in patients with inflammatory bowel disease and rheumatoid arthritis | World Journal of Clinical Cases | 10.12998/wjcc.v11.i4.764 |
| **283** | M. Hall, I. Bogdanovica and J. Britton | 2016 | Research funding for addressing tobacco-related disease: an analysis of UK investment between 2008 and 2012 | Bmj Open | 10.1136/bmjopen-2016-011609 |
| **284** | N. Hamade, G. Weng, M. Desai, V. T. Chandrasekar, C. Dasari, K. Kennedy and P. Sharma | 2021 | Significant decline in the prevalence of Barrett's esophagus among patients with gastroesophageal reflux disease | Diseases of the Esophagus | 10.1093/dote/doaa131 |
| **285** | J. M. Han, X. L. Guo, L. Zhao, H. Zhang, S. Q. Ma, Y. Li, D. L. Zhao, J. L. Wang and F. Z. Xue | 2023 | Development and Validation of Esophageal Squamous Cell Carcinoma Risk Prediction Models Based on an Endoscopic Screening Program | Jama Network Open | 10.1001/jamanetworkopen.2022.53148 |
| **286** | S. J. Han, S. J. Baik, Y. H. Yoon, J. H. Kim, H. S. Lee, S. Jeon and H. Park | 2023 | Risk of Metabolic Syndrome and Fatty Liver Diseases in Gastric Cancer Survivors: A Propensity Score-Matched Analysis | Korean Journal of Gastroenterology | 10.4166/kjg.2022.113 |
| **287** | Y. T. Han, X. Zhu, Y. Z. Hu, C. Q. Yu, Y. Guo, D. Hang, Y. J. Pang, P. Pei, H. X. Ma, D. J. Y. Sun, L. Yang, Y. P. Chen, H. D. Du, M. Yu, J. S. Chen, Z. M. Chen, D. Z. Huo, G. F. Jin, J. Lv, Z. B. Hu, H. B. Shen and L. M. Li | 2023 | Electronic Health Record-Based Absolute Risk Prediction Model for Esophageal Cancer in the Chinese Population: Model Development and External Validation | Jmir Public Health and Surveillance | 10.2196/43725 |
| **288** | H. A. Hanson, C. L. Leiser, B. O'Neil, C. Martin, S. Gupta, K. R. Smith, C. Dechet, W. T. Lowrance, M. J. Madsen and N. J. Camp | 2020 | Harnessing Population Pedigree Data and Machine Learning Methods to Identify Patterns of Familial Bladder Cancer Risk | Cancer Epidemiology Biomarkers & Prevention | 10.1158/1055-9965.Epi-19-0681 |
| **289** | T. Haque, A. Bin Nabhan, F. Akhter and H. N. Albagieh | 2023 | The analysis of periodontal diseases and squamous cell esophageal cancer: A retrospective study | Saudi Dental Journal | 10.1016/j.sdentj.2023.05.030 |
| **290** | S. Harari, M. Davi, A. Biffi, A. Caminati, A. Ghirardini, V. Lovato, C. Cricelli and F. Lapi | 2020 | Epidemiology of idiopathic pulmonary fibrosis: a population-based study in primary care | Internal and Emergency Medicine | 10.1007/s11739-019-02195-0 |
| **291** | S. Hardikar, L. Onstad, X. L. Song, A. M. Wilson, T. J. Montine, M. Kratz, G. L. Anderson, P. L. Blount, B. J. Reid, E. White and T. L. Vaughan | 2014 | Inflammation and Oxidative Stress Markers and Esophageal Adenocarcinoma Incidence in a Barrett's Esophagus Cohort | Cancer Epidemiology Biomarkers & Prevention | 10.1158/1055-9965.Epi-14-0384 |
| **292** | J. Z. Hashmi, M. Hiraj, F. Saleem, U. Malik and I. K. Mazari | 2022 | Double Peptic Ulcer Perforation due to Cumulative Effects of Post-surgery Stress and NSAIDs: A Rare Event in Surgical Practice | Jcpsp-Journal of the College of Physicians and Surgeons Pakistan | 10.29271/jcpsp.2022.JCPSPCR.CR21 |
| **293** | A. Hata, M. Yanagawa, T. Miyata, Y. Hiraoka, M. Shirae, K. Ninomiya, S. Doi, K. Yamagata, Y. Yoshida, N. Kikuchi, R. Ogawa, H. Hatabu and N. Tomiyama | 2024 | Association between interstitial lung abnormality and mortality in patients with esophageal cancer | Japanese Journal of Radiology | 10.1007/s11604-024-01563-x |
| **294** | E. Hayano, Y. Gon, Y. Kimura, L. Zha, T. Morishima, Y. Ohno, H. Mochizuki, T. Sobue and I. Miyashiro | 2024 | Risk of Parkinson's disease-related death in cancer survivors: A population-based study in Japan | Parkinsonism & Related Disorders | 10.1016/j.parkreldis.2023.105966 |
| **295** | Y. Hayashi, H. Iijima, F. Isohashi, Y. Tsujii, T. Fujinaga, K. Nagai, S. Yoshii, A. Sakatani, S. Hiyama, S. Shinzaki, T. Makino, M. Yamasaki, K. Ogawa, Y. Doki and T. Takehara | 2019 | The heart's exposure to radiation increases the risk of cardiac toxicity after chemoradiotherapy for superficial esophageal cancer: a retrospective cohort study | Bmc Cancer | 10.1186/s12885-019-5421-y |
| **296** | A. Hazarika, P. P. Bora and K. S. Kumar | 2016 | A CLINICAL STUDY ON INCIDENCE, PATHOLOGICAL PATTERN AND MANAGEMENT OF GASTRIC CARCINOMA IN RURAL SETUP (ADICHUNCHANAGIRI INSTITUTE OF MEDICAL SCIENCES), MANDYA | Journal of Evolution of Medical and Dental Sciences-Jemds | 10.14260/jemds/2016/496 |
| **297** | W. D. Hazelton, K. Curtius, J. M. Inadomi, T. L. Vaughan, R. Meza, J. H. Rubenstein, C. Hur and E. G. Luebeck | 2015 | The Role of Gastroesophageal Reflux and Other Factors during Progression to Esophageal Adenocarcinoma | Cancer Epidemiology Biomarkers & Prevention | 10.1158/1055-9965.Epi-15-0323-t |
| **298** | H. H. He, J. H. Fu, Z. X. Hao, H. F. Wu, Q. Zhong, F. Wang, H. H. Liu, X. S. Gu, B. Wang, H. D. Huang, Z. Y. Li and J. X. He | 2020 | Impact of metformin on survival outcome of esophageal squamous cell carcinomas patients undergoing surgical resection: a multicenter retrospective study | Journal of Thoracic Disease | 10.21037/jtd.2019.12.98 |
| **299** | H. Q. He, N. Z. Chen, Y. Hou, Z. Wang, Y. Zhang, G. J. Zhang and J. K. Fu | 2020 | Trends in the incidence and survival of patients with esophageal cancer: A SEER database analysis | Thoracic Cancer | 10.1111/1759-7714.13311 |
| **300** | P. P. Hekking, M. Amelink, R. R. Wener, M. L. Bouvy and E. H. Bel | 2018 | Comorbidities in Difficult-to-Control Asthma | Journal of Allergy and Clinical Immunology-in Practice | 10.1016/j.jaip.2017.06.008 |
| **301** | K. Hemminki, K. Sundquist, J. Sundquist, A. Försti, V. Liska, A. Hemminki and X. J. Li | 2022 | Familial Risks for Liver, Gallbladder and Bile Duct Cancers and for Their Risk Factors in Sweden, a Low-Incidence Country | Cancers | 10.3390/cancers14081938 |
| **302** | K. Henau, E. Van Eycken, G. Silversmit and E. Pukkala | 2015 | Regional variation in incidence for smoking and alcohol related cancers in Belgium | Cancer Epidemiology | 10.1016/j.canep.2014.10.009 |
| **303** | S. Hermann and V. Arndt | 2017 | Cancer - a global challenge | Onkologe | 10.1007/s00761-017-0287-6 |
| **304** | B. Y. Hernandez, R. A. Bordallo, M. D. Green and R. L. Haddock | 2017 | Cancer in Guam and Hawaii: A comparison of two US Island populations | Cancer Epidemiology | 10.1016/j.canep.2017.08.005 |
| **305** | M. Hirata, Y. Kamatani, A. Nagai, Y. Kiyohara, T. Ninomiya, A. Tamakoshi, Z. Yamagata, M. Kubo, K. Muto, T. Mushiroda, Y. Murakami, K. Yuji, Y. Furukawa, H. Zembutsu, T. Tanaka, Y. Ohnishi, Y. Nakamura, K. Matsuda and G. BioBank Japan Cooperative Hosp | 2017 | Cross-sectional analysis of BioBank Japan clinical data: A large cohort of 200,000 patients with 47 common diseases | Journal of Epidemiology | 10.1016/j.je.2016.12.003 |
| **306** | T. Hisamatsu, A. Kadota, T. Hayakawa, Y. Kita, A. Harada, Y. Okami, K. Kondo, T. Ohkubo, T. Okamura, A. Okayama, H. Ueshima, K. Miura and N. D. R. Grp | 2024 | High blood pressure and colorectal cancer mortality in a 29-year follow-up of the Japanese general population: NIPPON DATA80 | Hypertension Research | 10.1038/s41440-023-01497-3 |
| **307** | K. L. Hoffman, D. S. Hutchinson, J. Fowler, D. P. Smith, N. J. Ajami, H. Zhao, P. Scheet, W. H. Chow, J. F. Petrosino and C. R. Daniel | 2018 | Oral microbiota reveals signs of acculturation in Mexican American women | Plos One | 10.1371/journal.pone.0194100 |
| **308** | S. A. Hojati, S. Kokabpeyk, S. Yaghoubi, F. Joukar, M. Asgharnezhad and F. Mansour-Ghanaei | 2021 | <i>Helicobacter pylori</i> infection in Iran: demographic, endoscopic and pathological factors | Bmc Gastroenterology | 10.1186/s12876-021-01931-1 |
| **309** | K. J. Holzer, M. G. Vaughn, T. M. Loux, M. A. Mancini, N. E. Fearn and C. L. Wallace | 2022 | Prevalence and correlates of antisocial personality disorder in older adults | Aging & Mental Health | 10.1080/13607863.2020.1839867 |
| **310** | Z. N. Hong, K. Weng, Z. Chen, K. M. Peng and M. Q. Kang | 2022 | Difference between "Lung Age" and Real Age as a Novel Predictor of Postoperative Complications, Long-Term Survival for Patients with Esophageal Cancer after Minimally Invasive Esophagectomy | Frontiers in Surgery | 10.3389/fsurg.2022.794553 |
| **311** | T. Horinouchi, N. Yoshida, S. Shiraishi, Y. Hara, C. Matsumoto, T. Toihata, K. Kosumi, K. Harada, K. Eto, K. Ogawa, H. Sawayama, M. Iwatsuki, Y. Baba, Y. Miyamoto and H. Baba | 2024 | Relationship between the severity of emphysematous change in the lung and morbidity after esophagectomy for esophageal cancer: A retrospective study on a novel strategy for risk prediction | Thoracic Cancer | 10.1111/1759-7714.15146 |
| **312** | Y. Horiuchi, J. Fujisaki, N. Ishizuka, M. Omae, A. Ishiyama, T. Yoshio, T. Hirasawa, Y. Yamamoto, M. Nagahama, H. Takahashi and T. Tsuchida | 2017 | Study on Clinical Factors Involved in <i>Helicobacter pylori</i>-Uninfected, Undifferentiated-Type Early Gastric Cancer | Digestion | 10.1159/000481817 |
| **313** | C. C. Horn | 2014 | The Medical Implications of Gastrointestinal Vagal Afferent Pathways in Nausea and Vomiting | Current Pharmaceutical Design | 10.2174/13816128113199990568 |
| **314** | R. Howard, K. Singh and M. Englesbe | 2021 | Prevalence and Trends in Smoking Among Surgical Patients in Michigan, 2012-2019 | Jama Network Open | 10.1001/jamanetworkopen.2021.0553 |
| **315** | K. Y. Hsu, Y. F. Tsai, C. C. Huang, W. L. Yeh, K. P. Chang, C. C. Ling, C. Y. Chen and H. L. Lee | 2018 | Tobacco-Smoking, Alcohol-Drinking, and Betel-Quid-Chewing Behaviors: Development and Use of a Web-Based Survey System | Jmir Mhealth and Uhealth | 10.2196/mhealth.9783 |
| **316** | Q. Huang, Y. Q. Cheng, E. Lew, J. Shi, D. Wiener and H. C. Weber | 2023 | Patients with esophageal adenocarcinoma showed better prognosis than those with adenocarcinoma of the gastroesophageal junction | Journal of Digestive Diseases | 10.1111/1751-2980.13167 |
| **317** | Q. Y. Huang, K. J. Luo, H. Yang, J. Wen, S. S. Zhang, J. H. Li, A. E. Bella, Q. W. Liu, F. Yang, Y. Z. Zheng, R. G. Hu, J. Y. Chen and J. H. Fu | 2014 | Impact of alcohol consumption on survival in patients with esophageal carcinoma: A large cohort with long-term follow-up | Cancer Science | 10.1111/cas.12552 |
| **318** | X. C. Huang, S. H. Guan, J. F. Wang, L. L. Zhao, Y. B. Jia, Z. L. Lu, C. P. Yin, S. S. Yang, Q. X. Song, L. H. Han, C. Wang, J. Y. Li, W. Zhou, X. L. Guo and Y. F. Cheng | 2017 | The effects of air pollution on mortality and clinicopathological features of esophageal cancer | Oncotarget | 10.18632/oncotarget.17266 |
| **319** | Y. C. Huang, M. C. Lee, S. Y. Huang, C. M. Chou, H. W. Yang and I. C. Chen | 2024 | Polygenic Risk Score in Predicting Esophageal, Oropharyngeal, and Hypopharynx Cancer Risk among Taiwanese Population | Cancers | 10.3390/cancers16040707 |
| **320** | Y. H. Huang, Q. R. Hu, Z. X. Wei, L. Chen, Y. Luo, X. J. Li and C. P. Li | 2023 | Influence of <i>MTHFR</i> polymorphism, alone or in combination with smoking and alcohol consumption, on cancer susceptibility | Open Life Sciences | 10.1515/biol-2022-0680 |
| **321** | M. P. Hunter and N. J. Crowther | 2019 | The prevalence of gastroesophageal reflux disease in an adult, South African black population, and the association with obesity | Minerva Gastroenterologica E Dietologica | 10.23736/s1121-421x.18.02495-9 |
| **322** | T. Inoue, S. Ito, M. Ando, M. Nagaya, H. Aso, Y. Mizuno, K. Hattori, H. Nakajima, Y. Nishida, Y. Niwa, Y. Kodera, M. Koike and Y. Hasegawa | 2016 | Changes in exercise capacity, muscle strength, and health-related quality of life in esophageal cancer patients undergoing esophagectomy | Bmc Sports Science Medicine and Rehabilitation | 10.1186/s13102-016-0060-y |
| **323** | M. Z. Irani, N. J. Talley, J. Ronkainen, P. Aro, A. Andreasson, L. Agreus, M. Vieth, M. P. Jones and M. M. Walker | 2021 | Neutrophils, eosinophils, and intraepithelial lymphocytes in the squamous esophagus in subjects with and without gastroesophageal reflux symptoms | Human Pathology | 10.1016/j.humpath.2021.06.004 |
| **324** | N. S. Irhayyim, M. A. A. Ahmed and H. J. Mahmood | 2018 | Evaluation of salivary Aspartate Aminotransferase Enzyme level in Smoker Patients with Peptic Ulcer in Relation to Periodontal Condition | Research Journal of Pharmaceutical Biological and Chemical Sciences |  |
| **325** | Y. Iwaya, Y. Shimamura, K. Goda, E. R. de Santiago, J. G. Coneys, J. D. Mosko, G. Kandel, P. Kortan, G. May, N. Marcon and C. Teshima | 2019 | Clinical characteristics of young patients with early Barrett's neoplasia | World Journal of Gastroenterology | 10.3748/wjg.v25.i24.3069 |
| **326** | Y. Iwaya, Y. Shimamura, J. D. Mosko, G. Kandel, P. P. Kortan, G. R. May, N. E. Marcon and C. W. Teshima | 2019 | Clinical characteristics may distinguish patients with esophageal adenocarcinoma arising from long- versus short-segment Barrett's esophagus | Digestive and Liver Disease | 10.1016/j.dld.209.05.000 |
| **327** | N. Izadi, K. Etemad, P. Mohseni, A. Khosravi and M. E. Akbari | 2022 | Mortality Rates and Years of Life Lost Due to Cancer in Iran: Analysis of Data from the National Death Registration System, 2016 | International Journal of Cancer Management | 10.5812/ijcm-123633 |
| **328** | A. Izzotti, M. Longobardi, S. La Maestra, R. T. Micale, A. Pulliero, A. Camoirano, M. Geretto, F. D'Agostini, R. Balansky, M. S. Miller, V. E. Steele and S. De Flora | 2018 | Release of MicroRNAs into Body Fluids from Ten Organs of Mice Exposed to Cigarette Smoke | Theranostics | 10.7150/thno.22726 |
| **329** | P. Jaehn, B. Holleczek, H. Becher and V. Winkler | 2016 | Histologic types of gastric cancer among migrants from the former Soviet Union and the general population in Germany: what kind of prevention do we need? | European Journal of Gastroenterology & Hepatology | 10.1097/meg.0000000000000645 |
| **330** | S. Jain and S. Dhingra | 2017 | Pathology of esophageal cancer and Barrett's esophagus | Annals of Cardiothoracic Surgery | 10.21037/acs.2017.03.06 |
| **331** | S. Javali, M. Madan, M. L. Harendrakumar and M. S. Mahesh | 2015 | Role of endoscopy in evaluating upper gastrointestinal tract lesions in rural population | Journal of Digestive Endoscopy | 10.4103/0976-5042.159238 |
| **332** | M. Javeed, H. Gruhonjic, T. Kirkman, C. Pitarys and R. Akel | 2022 | A Unique Case of a Right Atrial Myxoma Infected With Escherichia coli | Cureus Journal of Medical Science | 10.7759/cureus.25394 |
| **333** | H. Jayasekara, R. J. MacInnis, L. Lujan-Barroso, A. L. Mayen-Chacon, A. J. Cross, B. Wallner, D. Palli, F. Ricceri, V. Pala, S. Panico, R. Tumino, T. Kühn, R. Kaaks, K. Tsilidis, M. J. Sánchez, P. Amiano, E. Ardanaz, M. D. C. López, S. Merino, J. A. Rothwell, M. C. Boutron-Ruault, G. Severi, H. Sternby, E. Sonestedt, B. Bueno-de-Mesquita, H. Boeing, R. Travis, T. M. Sandanger, A. Trichopoulou, A. Karakatsani, E. Peppa, A. Tjonneland, Y. Yang, A. M. Hodge, H. Mitchell, A. Haydon, R. Room, J. L. Hopper, E. Weiderpass, M. J. Gunter, E. Riboli, G. G. Giles, R. L. Milne, A. Agudo, D. R. English and P. Ferrari | 2021 | Lifetime alcohol intake, drinking patterns over time and risk of stomach cancer: A pooled analysis of data from two prospective cohort studies | International Journal of Cancer | 10.1002/ijc.33504 |
| **334** | J. W. Jeon, S. J. Kim, J. Y. Jang, S. M. Kim, C. H. Lim, J. M. Park, S. J. Hong, C. G. Kim, S. W. Jeon, S. H. Lee, J. K. Sung and G. H. Baik | 2021 | Clinical Outcomes of Endoscopic Resection for Low-Grade Dysplasia and High-Grade Dysplasia on Gastric Pretreatment Biopsy: Korea ESD Study Group | Gut and Liver | 10.5009/gnl19275 |
| **335** | Y. J. Jeon, K. Y. D. Han, S. W. Lee, J. E. Lee, J. Park, I. Y. Cho, J. H. Cho and D. W. Shin | 2024 | Metabolic dysfunction-associated steatotic liver disease and risk of esophageal cancer in patients with diabetes mellitus: a nationwide cohort study | Diseases of the Esophagus | 10.1093/dote/doae029 |
| **336** | A. Jia, Y. Wu, W. L. Ren, P. Han and Y. Shao | 2020 | Genetic variations of <i>CARMN</i> affect risk of esophageal cancer in northwest China | Gene | 10.1016/j.gene.2020.144680 |
| **337** | S. C. Jia, H. Li, H. M. Zeng, R. S. Zheng, J. Li, J. F. Shi, Z. X. Yang, M. M. Cao and W. Q. Chen | 2019 | Association of cancer prevention awareness with esophageal cancer screening participation rates: Results from a population-based cancer screening program in rural China | Chinese Journal of Cancer Research | 10.21147/j.issn.1000-9604.2019.04.04 |
| **338** | X. X. Jia, C. Sheng, X. X. Han, M. Y. Li and K. J. Wang | 2024 | Global burden of stomach cancer attributable to smoking from 1990 to 2019 and predictions to 2044 | Public Health | 10.1016/j.puhe.2023.11.019 |
| **339** | D. X. Jiang, Q. Song, F. H. Zhang, C. Xu, X. J. Li, H. Y. Zeng, J. Su, J. Huang, Y. F. Xu, S. H. Lu and Y. Y. Hou | 2023 | Prognostic significance of CCND1 amplification/overexpression in smoking patients with esophageal squamous cell carcinoma | Cancer Genetics | 10.1016/j.cancergen.2023.07.004 |
| **340** | Q. W. Jiang, Y. Y. Shu, Z. Y. Jiang, Y. Q. Zhang, S. W. Pan, W. H. Jiang, J. X. Liang, X. D. Cheng and Z. Y. Xu | 2024 | Burdens of stomach and esophageal cancer from 1990 to 2019 and projection to 2030 in China: Findings from the 2019 Global Burden of Disease Study | Journal of Global Health | 10.7189/jogh.14.04025 |
| **341** | S. J. Jiang, A. C. Diaconescu, D. P. McEwen, L. N. McEwen, A. C. Chang, J. L. Lin, R. M. Reddy, W. R. Lynch, S. Bonner and K. H. Lagisetty | 2023 | Factors affecting timing of surgery following neoadjuvant chemoradiation for esophageal cancer | Heliyon | 10.1016/j.heliyon.2023.e23212 |
| **342** | Y. Jiang, Y. C. Lin, Y. K. Wen, W. H. Fu, R. Wang, J. X. He, J. R. Zhang, Z. F. Wang, F. Ge, Z. Y. Huo, R. C. Wang, H. X. Peng, X. R. Wu, J. X. He and S. B. Li | 2023 | Global trends in the burden of esophageal cancer, 1990-2019 Results from the Global Burden of Disease Study 2019 | Journal of Thoracic Disease | 10.21037/jtd-22-856 |
| **343** | W. P. Jiao, J. Y. Zhang, Y. Y. Wei, J. H. Feng, M. Ma, H. Z. Zhao, L. H. Wang and W. J. Jiao | 2019 | MiR-139-5p regulates VEGFR and downstream signaling pathways to inhibit the development of esophageal cancer | Digestive and Liver Disease | 10.1016/j.dld.2018.07.017 |
| **344** | B. Jideh, M. Weltman, Y. Wu and C. H. Y. Chan | 2017 | Esophageal squamous papilloma lacks clear clinicopathological associations | World Journal of Clinical Cases | 10.12998/wjcc.v5.i4.134 |
| **345** | C. A. Jiménez-Ruiz, M. García, M. A. Martínez, J. Sellarés, M. A. Jiménez-Fuentes, L. Lázaroo, E. Rodríguez, C. Rodriguez, O. Armengo, E. Abad, T. Peña, A. Domenech and J. A. Riescos | 2017 | Varenicline in smokers with severe or very severe COPD after 24 weeks of treatment. A descriptive analysis: VALUE study | Monaldi Archives for Chest Disease | 10.4081/monaldi.2017.874 |
| **346** | Z. Y. Jin, G. Wallar, J. Y. Zhou, J. Yang, R. Q. Han, P. H. Wang, A. M. Liu, X. P. Gu, X. F. Zhang, X. S. Wang, M. Su, X. Hu, Z. Sun, G. Li, L. N. Mu, Q. Y. Lu, X. Liu, L. M. Li, N. He, M. Wu, J. K. Zhao and Z. F. Zhang | 2019 | Consumption of garlic and its interactions with tobacco smoking and alcohol drinking on esophageal cancer in a Chinese population | European Journal of Cancer Prevention | 10.1097/cej.0000000000000456 |
| **347** | O. M. P. Jolobe | 2021 | Differential diagnosis of the association of gastrointestinal symptoms and ST segment elevation, in the absence of chest pain | American Journal of Emergency Medicine | 10.1016/j.ajem.2021.05.067 |
| **348** | Y. S. Jung and S. J. Yoon | 2022 | Burden of Cancer Due to Cigarette Smoking and Alcohol Consumption in Korea | International Journal of Environmental Research and Public Health | 10.3390/ijerph19063493 |
| **349** | S. Kageyama, T. Takeshita, K. Takeuchi, M. Asakawa, R. Matsumi, M. Furuta, Y. Shibata, K. Nagai, M. Ikebe, M. Morita, M. Masuda, Y. Toh, Y. Kiyohara, T. Ninomiya and Y. Yamashita | 2019 | Characteristics of the Salivary Microbiota in Patients With Various Digestive Tract Cancers | Frontiers in Microbiology | 10.3389/fmicb.2019.01780 |
| **350** | B. Kaimila, Y. X. Chen, G. Mulima, C. Kajombo, A. Salima, Y. Yano, S. Gopal, S. M. Dawsey and C. C. Abnet | 2023 | Survival After Diagnosis of Esophageal Squamous Cell Carcinoma in Malawi | Jco Global Oncology | 10.1200/go.23.00173 |
| **351** | Y. Kamiide, N. Inomata, M. Furuya and T. Yada | 2015 | Ghrelin ameliorates catabolic conditions and respiratory dysfunction in a chronic obstructive pulmonary disease model of chronic cigarette smoke-exposed rats | European Journal of Pharmacology | 10.1016/j.ejphar.2015.02.049 |
| **352** | K. J. Kamp and M. Stommel | 2021 | Health-Related Quality of Life Among Patients With Inflammatory Bowel Disease A Case Control Study | Gastroenterology Nursing | 10.1097/sga.0000000000000491 |
| **353** | G. Kanagalingam, Y. Achuo-Egbe, M. F. Ahmed, O. Oluaderounmu and J. Harley | 2022 | A Rare Case of Esophageal Leukoplakia in Achalasia | Cureus Journal of Medical Science | 10.7759/cureus.23735 |
| **354** | K. Kanamori, D. Kurita, Y. Hirano, K. Ishiyama, J. Oguma, K. Masutomi and H. Daiko | 2022 | Does synchronous early head and neck cancer with esophageal cancer need treatment after preoperative chemotherapy? | General Thoracic and Cardiovascular Surgery | 10.1007/s11748-021-01744-9 |
| **355** | H. Kaneko, Y. Suzuki, K. Ueno, A. Okada, K. Fujiu, S. Matsuoka, N. Michihata, T. Jo, N. Takeda, H. Morita, K. Kamiya, K. Node, H. Yasunaga and I. Komuro | 2022 | Association of Life's Simple 7 with incident cardiovascular disease in 53 974 patients with cancer | European Journal of Preventive Cardiology | 10.1093/eurjpc/zwac195 |
| **356** | S. H. Kang, Y. Lim, H. Lee, J. Kim, S. Chi, Y. W. Min, B. H. Min, J. H. Lee, H. J. Son, S. Ryu, P. L. Rhee and J. J. Kim | 2016 | A Model for Predicting the Future Risk of Incident Erosive Esophagitis in an Asymptomatic Population Undergoing Regular Check-ups | Medicine | 10.1097/md.0000000000002591 |
| **357** | Y. Kanie, A. Okamura, K. Tomizuka, T. Uchiyama, J. Kanamori, Y. Imamura, T. Ebata and M. Watanabe | 2023 | Quantitative Evaluation of Periodontitis for Predicting the Occurrence of Postoperative Pneumonia After Oncologic Esophagectomy | Annals of Surgical Oncology | 10.1245/s10434-023-14030-0 |
| **358** | C. H. Kao, L. M. Sun, Y. S. Chen, C. L. Lin, J. A. Liang, C. H. Kao and M. W. Weng | 2016 | Risk of Nongenitourinary Cancers in Patients With Spinal Cord Injury A Population-based Cohort Study | Medicine | 10.1097/md.0000000000002462 |
| **359** | A. Karbasi, R. Aliannejad, M. Ghanei, M. N. Sanamy, F. Alaeddini and A. A. Harandi | 2015 | Frequency distribution of gastro esophageal reflux disease in inhalation injury: A historical cohort study | Journal of Research in Medical Sciences | 10.4103/1735-1995.166199 |
| **360** | A. Karlsson, A. Ellonen, H. Irjala, V. Väliaho, K. Mattila, L. Nissi, E. Kytö, S. Kurki, R. Ristamäki, P. Vihinen, T. Laitinen, A. Ålgars, S. Jyrkkiö, H. Minn and E. Heervä | 2021 | Impact of deep learning-determined smoking status on mortality of cancer patients: never too late to quit | Esmo Open | 10.1016/j.esmoop.2021.100175 |
| **361** | E. Kasap, A. Ayer, H. Bozoglan, C. Ozen, I. Eslek and H. Yüceyar | 2015 | Schizophrenia and gastroesophageal reflux symptoms | Indian Journal of Psychiatry | 10.4103/0019-5545.148529 |
| **362** | S. Katuwal, P. Jousilahti and E. Pukkala | 2021 | Causes of death among women with breast cancer: A follow-up study of 50 481 women with breast cancer in Finland | International Journal of Cancer | 10.1002/ijc.33607 |
| **363** | J. H. Kauppila, K. Wahlin, P. Lagergren and J. Lagergren | 2019 | Sex differences in the prognosis after surgery for esophageal squamous cell carcinoma and adenocarcinoma | International Journal of Cancer | 10.1002/ijc.31840 |
| **364** | H. G. Kay, B. Campbell, J. N. Gallant, C. Carlile, P. Wright, B. Stephens and S. L. Rohde | 2021 | Delayed Upper Aerodigestive Tract Perforation from Anterior Cervical Spine Hardware: Treatment and Swallowing Outcomes | Dysphagia | 10.1007/s00455-021-10361-w |
| **365** | Z. Kaya and S. Gursoy | 2023 | Association Between CYP1A1 Polymorphisms and Esophageal Cancer Susceptibility: A Case-control Study | In Vivo | 10.21873/invivo.13155 |
| **366** | J. Z. Ke, T. Lin, X. L. Liu, K. Wu, X. N. Ruan, Y. B. Ding, W. B. Liu, H. Qiu, X. J. Tan, X. N. Wang, X. Chen, Z. T. Li and G. W. Cao | 2021 | Glucose Intolerance and Cancer Risk: A Community-Based Prospective Cohort Study in Shanghai, China | Frontiers in Oncology | 10.3389/fonc.2021.726672 |
| **367** | L. E. Kelemen, M. Earp, B. L. Fridley, G. Chenevix-Trench, P. A. Fasching, M. W. Beckmann, A. B. Ekici, A. Hein, D. Lambrechts, S. Lambrechts, E. Van Nieuwenhuysen, I. Vergote, M. A. Rossing, J. A. Doherty, J. Chang-Claude, S. Behrens, K. B. Moysich, R. Cannioto, S. Lele, K. Odunsi, M. T. Goodman, Y. B. Shvetsov, P. J. Thompson, L. R. Wilkens, T. Dörk, N. Antonenkova, N. Bogdanova, P. Hillemanns, I. B. Runnebaum, A. du Bois, P. Harter, F. Heitz, I. Schwaab, R. Butzow, L. M. Pelttari, H. Nevanlinna, F. Modugno, R. P. Edwards, J. L. Kelley, R. B. Ness, B. Y. Karlan, J. Lester, S. Orsulic, C. Walsh, S. K. Kjær, A. Jensen, J. M. Cunningham, R. A. Vierkant, G. G. Giles, F. Bruinsma, M. C. Southey, M. A. T. Hildebrandt, D. Liang, K. R. Lu, X. F. Wu, T. A. Sellers, D. A. Levine, J. M. Schildkraut, E. S. Iversen, K. L. Terry, D. W. Cramer, S. S. Tworoger, E. M. Poole, E. V. Bandera, S. H. Olson, I. Orlow, L. C. V. Thomsen, L. Bjorge, C. Krakstad, I. L. Tangen, L. A. Kiemeney, K. K. H. Aben, L. Massuger, A. M. van Altena, T. Pejovic, Y. Bean, M. Kellar, L. S. Cook, N. D. Le, A. Brooks-Wilson, J. Gronwald, C. Cybulski, A. Jakubowska, J. Lubinski, N. Wentzensen, L. A. Brinton, J. Lissowska, E. Hogdall, S. A. Engelholm, C. Hogdall, L. Lundvall, L. Nedergaard, P. D. P. Pharoah, E. Dicks, H. L. Song, J. P. Tyrer, I. McNeish, N. Siddiqui, K. Carty, R. Glasspool, J. Paul, I. G. Campbell, D. Eccles, A. S. Whittemore, V. McGuire, J. H. Rothstein, W. Sieh, S. A. Narod, C. M. Phelan, J. R. McLaughlin, H. A. Risch, H. Anton-Culver, A. Ziogas, U. Menon, S. A. Gayther, A. Gentry-Maharaj, S. J. Ramus, A. H. Wu, C. L. Pearce, A. W. Lee, M. C. Pike, J. Kupryjanczyk, A. Podgorska, J. Plisiecka-Halasa, W. Sawicki, E. L. Goode, A. Berchuck, G. Australian Ovarian Canc Study and C. Ovarian Cancr Assoc | 2018 | rs495139 in the <i>TYMS</i>-<i>ENOSF1</i> Region and Risk of Ovarian Carcinoma of Mucinous Histology | International Journal of Molecular Sciences | 10.3390/ijms19092473 |
| **368** | J. Keller, G. Bassotti, J. Clarke, P. Dinning, M. Fox, M. Grover, P. M. Hellström, M. Y. Ke, P. Layer, C. Malagelada, H. P. Parkman, S. M. Scott, J. Tack, M. Simren, H. Törnblom, M. Camilleri and G. Int Working Grp Disorders | 2018 | Advances in the diagnosis and classification of gastric and intestinal motility disorders | Nature Reviews Gastroenterology & Hepatology | 10.1038/nrgastro.2018.7 |
| **369** | A. Khaledifar, M. Hashemzadeh, K. Solati, H. Poustchi, V. Bollati, A. Ahmadi, S. Kheiri, K. G. Samani, M. Banitalebi, M. Sedehi and R. Malekzadeh | 2018 | The protocol of a population-based prospective cohort study in southwest of Iran to analyze common non-communicable diseases: Shahrekord cohort study | Bmc Public Health | 10.1186/s12889-018-5364-2 |
| **370** | H. Khalid, A. Zahid and M. W. Zahid | 2018 | A CROSS SECTIONAL STUDY; GERD AS A RISK FACTOR OF ACUTE EXACERBATION OF COPD | Indo American Journal of Pharmaceutical Sciences | 10.5281/zenodo.1245715 |
| **371** | D. H. Kim, E. J. Gong, H. Y. Jung, H. Lim, J. Y. Ahn, K. S. Choi, J. H. Lee, K. D. Choi, H. J. Song, G. H. Lee, J. H. Kim, J. L. Roh, S. H. Choi, S. Y. Nam, S. Y. Kim and S. Baek | 2014 | Clinical significance of intensive endoscopic screening for synchronous esophageal neoplasm in patients with head and neck squamous cell carcinoma | Scandinavian Journal of Gastroenterology | 10.3109/00365521.2013.832369 |
| **372** | E. H. Kim, S. Nam, C. H. Park, Y. Kim, M. Lee, J. B. Ahn, S. J. Shin, Y. R. Park, H. I. Jung, B. I. Kim, I. Jung and H. S. Kim | 2022 | Periodontal disease and cancer risk: A nationwide population-based cohort study | Frontiers in Oncology | 10.3389/fonc.2022.901098 |
| **373** | H. J. Kim, N. Kim, H. Y. Kim, H. S. Lee, H. Yoon, C. M. Shin, Y. S. Park, D. J. Park, H. H. Kim, K. H. Lee, Y. H. Kim, H. M. Kim and D. H. Lee | 2015 | Relationship between body mass index and the risk of early gastric cancer and dysplasia regardless of <i>Helicobacter pylori</i> infection | Gastric Cancer | 10.1007/s10120-014-0429-0 |
| **374** | H. S. Kim, S. E. Lee, Y. S. Bae, D. J. Kim, C. G. Lee, J. Hur, H. Chung, J. C. Park, D. H. Jung, S. K. Shin, S. K. Lee, Y. C. Lee, H. R. Kim, Y. W. Moon, J. H. Kim, Y. M. Shim, S. S. Jewell, H. Kim, Y. L. Choi and B. C. Cho | 2015 | Fibroblast growth factor receptor 1 gene amplification is associated with poor survival in patients with resected esophageal squamous cell carcinoma | Oncotarget | 10.18632/oncotarget.2944 |
| **375** | M. Kim, K. S. Choi, M. Suh, J. K. Jun, K. W. Chuck and B. Park | 2018 | Risky Lifestyle Behaviors among Gastric Cancer Survivors Compared with Matched Non-cancer Controls: Results from Baseline Result of Community Based Cohort Study | Cancer Research and Treatment | 10.4143/crt.2017.129 |
| **376** | S. W. Kim, J. H. Lee, Y. S. Sim, Y. J. Ryu and J. H. Chang | 2014 | Prevalence and risk factors for reflux esophagitis in patients with chronic obstructive pulmonary disease | Korean Journal of Internal Medicine | 10.3904/kjim.2014.29.4.466 |
| **377** | S. Y. Kim, H. K. Jung, J. Lim, T. O. Kim, A. R. Choe, C. H. Tae, K. N. Shim, C. M. Moon, S. E. Kim and S. A. Jung | 2019 | Gender Specific Differences in Prevalence and Risk Factors for Gastro-Esophageal Reflux Disease | Journal of Korean Medical Science | 10.3346/jkms.2019.34.e158 |
| **378** | Y. Kim, S. Ganocy and R. Fass | 2020 | Proton-pump inhibitor use and the development of new ischemic heart disease in non-cardiac chest pain patients | Neurogastroenterology and Motility | 10.1111/nmo.13844 |
| **379** | Y. Kim, S. Sharp, S. Hwang and S. H. Jee | 2019 | Exercise and incidence of myocardial infarction, stroke, hypertension, type 2 diabetes and site-specific cancers: prospective cohort study of 257 854 adults in South Korea | Bmj Open | 10.1136/bmjopen-2018-025590 |
| **380** | Y. J. Kim, W. C. Chung, I. H. Cho, J. Kim and S. Kim | 2019 | Prognostic effect of different etiologies in patients with gastric cardia cancer | Medicine | 10.1097/md.0000000000018397 |
| **381** | A. Kimura, N. Morinaga, W. Wada, K. Ogata, T. Okuyama, H. Kato, M. Sohda, K. Shirabe and H. Saeki | 2022 | Patient with gastric cancer who underwent distal gastrectomy after treatment of COVID-19 infection diagnosed by preoperative PCR screening | Surgical Case Reports | 10.1186/s40792-022-01367-z |
| **382** | R. Kinoshita-Daitoku, K. Kiga, M. Miyakoshi, R. Otsubo, Y. Ogura, T. Sanada, Z. Bo, T. V. Phuoc, T. Okano, T. Iida, R. Yokomori, E. Kuroda, S. Hirukawa, M. Tanaka, A. Sood, P. Subsomwong, H. Ashida, T. T. Binh, L. T. Nguyen, K. V. Van, D. Q. D. Ho, K. Nakai, T. Suzuki, Y. Yamaoka, T. Hayashi and H. Mimuro | 2021 | A bacterial small RNA regulates the adaptation of <i>Helicobacter pylori</i> to the host environment | Nature Communications | 10.1038/s41467-021-22317-7 |
| **383** | Y. Kishida, T. Tsushima, M. Endo, S. Hamauchi, A. Todaka, T. Yokota, N. Machida, K. Yamazaki, A. Fukutomi, Y. Onozawa and H. Yasui | 2018 | Risk Analysis of Pneumonitis in Taxane Therapy After Chemoradiotherapy for Patients With Metastatic or Recurrent Esophageal Cancer | American Journal of Clinical Oncology-Cancer Clinical Trials | 10.1097/coc.0000000000000232 |
| **384** | S. Kizildag, F. Hosgorler, G. Güvendi, T. B. Koc, S. Kandis, A. Argon, M. Ates and N. Uysal | 2021 | Nicotine lowers TNF-α, IL-1b secretion and leukocyte accumulation via nAChR in rat stomach | Toxin Reviews | 10.1080/15569543.2020.1790604 |
| **385** | M. Kohailan, M. Alanazi, M. Rouabhia, A. Alamri, N. R. Parine, A. Alhadheq, S. Basavarajappa, A. A. A. Al-Kheraif and B. Semlali | 2016 | Effect of smoking on the genetic makeup of toll-like receptors 2 and 6 | Oncotargets and Therapy | 10.2147/ott.S109650 |
| **386** | H. K. Koo, I. Jeong, S. W. Lee, J. Park, J. H. Kim, S. Y. Park, H. Y. Park, C. K. Rhee, Y. H. Kim, J. Y. Jung, S. K. Kim, Y. H. Kim, E. Y. Choi, J. Y. Moon, J. W. Shin, J. W. Kim, K. H. Min, S. W. Kim, K. H. Yoo, J. H. Kim, S. H. Jang, H. K. Yoon, H. J. Kim, K. S. Jung and D. K. Kim | 2016 | Prevalence of chronic cough and possible causes in the general population based on the Korean National Health and Nutrition Examination Survey | Medicine | 10.1097/md.0000000000004595 |
| **387** | G. J. Korpanty, L. Eng, X. Qiu, O. O. Faluyi, D. J. Renouf, D. X. Cheng, D. Patel, Z. Chen, B. C. Tse, J. J. Knox, L. Dodbiba, J. Teichman, A. K. Azad, R. Wong, G. Darling, D. Reisman, S. Cuffe, G. Liu and W. Xu | 2017 | Association of BRM promoter polymorphisms and esophageal adenocarcinoma outcome | Oncotarget | 10.18632/oncotarget.15890 |
| **388** | H. O. Koskela, A. M. Lätti and M. K. Purokivi | 2017 | Long-term prognosis of chronic cough: a prospective, observational cohort study | Bmc Pulmonary Medicine | 10.1186/s12890-017-0496-1 |
| **389** | I. O. Kostitska, B. M. Mankovsky, A. M. Urbanovych, O. Y. Zhurakivska, O. V. Tymoshchuk and I. O. Basiuha | 2019 | Risk factors and early detection of gastroparesis in patients with type 2 diabetes mellitus | Regulatory Mechanisms in Biosystems | 10.15421/021909 |
| **390** | K. Kou, P. D. Baade, X. L. Guo, M. Gatton, S. Cramb, Z. L. Lu, Z. T. Fu, J. Chu, A. Q. Xu and J. D. Sun | 2019 | Area socioeconomic status is independently associated with esophageal cancer mortality in Shandong, China | Scientific Reports | 10.1038/s41598-019-42774-x |
| **391** | O. V. Krapivnaia | 2023 | Comparison of the effectiveness of rabeprazole original and generic products in the monotherapy of gastroesophageal reflux disease | Terapevticheskii Arkhiv | 10.26442/00403660.2023.08.202346 |
| **392** | T. B. Kratzer, A. Jemal, K. D. Miller, S. Nash, C. Wiggins, D. Redwood, R. Smith and R. L. Siegel | 2023 | Cancer statistics for American Indian and Alaska Native individuals, 2022: Including increasing disparities in early onset colorectal cancer | Ca-a Cancer Journal for Clinicians | 10.3322/caac.21757 |
| **393** | L. Kravchenko, I. Borisyuk, N. Fizor, L. Unhurian, E. Zolotukhina and O. Goncharenko | 2020 | Local Use of Apisan Gel, A New Oral Care Product in the Treatment of Experimental Periodontitis Against the Background of Hyperacid Gastritis and Intoxication with Tobacco Smoke | Turkish Journal of Pharmaceutical Sciences | 10.4274/tjps.galenos.2018.53386 |
| **394** | M. Kreuzer, V. Deffner, M. Schnelzer and N. Fenske | 2021 | Mortality in Underground Miners in a Former Uranium Ore Mine Results of a Cohort Study Among Former Employees of Wismut AG in Saxony and Thuringia | Deutsches Arzteblatt International | 10.3238/arztebl.m2021.0001 |
| **395** | K. Kridin, S. Zelber-Sagi, D. Comaneshter and A. D. Cohen | 2018 | Coexistent Solid Malignancies in Pemphigus A Population-Based Study | Jama Dermatology | 10.1001/jamadermatol.2017.6334 |
| **396** | A. Krishnamoorthy and K. Kuberan | 2021 | Clinico Pathological Profiles of Patients with Proximal Versus Distal Gastric Adenocarcinoma | Journal of Research in Medical and Dental Science |  |
| **397** | C. Kromer, J. Xu, Q. T. Ostrom, H. Gittleman, C. Kruchko, R. Sawaya and J. S. Barnholtz-Sloan | 2017 | Estimating the annual frequency of synchronous brain metastasis in the United States 2010-2013: a population-based study | Journal of Neuro-Oncology | 10.1007/s11060-017-2516-7 |
| **398** | H. S. Küçükerdem, M. Arslan, E. M. Koç and H. Can | 2017 | Retrospective Evaluation of Family Medicine Outpatient Clinic Profile at a Tertiary Hospital in Izmir | Journal of Academic Research in Medicine-Jarem | 10.5152/jarem.2017.1165 |
| **399** | S. Kumar, D. S. Goldberg and D. E. Kaplan | 2022 | Ranitidine Use and Gastric Cancer Among Persons with <i>Helicobacter pylori</i> | Digestive Diseases and Sciences | 10.1007/s10620-021-06972-w |
| **400** | S. Kumar, D. C. Metz, S. Ellenberg, D. E. Kaplan and D. S. Goldberg | 2020 | Risk Factors and Incidence of Gastric Cancer After Detection of <i>Helicobacter pylori</i> Infection: A Large Cohort Study | Gastroenterology | 10.1053/j.gastro.2019.10.019 |
| **401** | S. S. Kumar, V. Gunda, D. M. Reinartz, K. W. Pond, C. A. Thorne, P. V. S. Raj, M. D. L. Johnson and J. E. Wilson | 2024 | Oral streptococci <i>S. anginosus</i> and <i>S. mitis</i> induce distinct morphological, inflammatory, and metabolic signatures in macrophages | Infection and Immunity | 10.1128/iai.00536-23 |
| **402** | B. Kumaraswamy | 2015 | ACUTE PERFORATED PEPTIC ULCER: A CLINICAL, DIAGNOSTIC AND MANAGEMENT ANALYSIS IN A TERTIARY HOSPITAL OF TELANGANA | Journal of Evolution of Medical and Dental Sciences-Jemds | 10.14260/jemds/2015/2417 |
| **403** | A. T. Kunzmann, A. P. Thrift, C. R. Cardwell, J. Lagergren, S. H. Xie, B. T. Johnston, L. A. Anderson, J. Busby, U. C. McMenamin, A. D. Spence and H. G. Coleman | 2018 | Model for Identifying Individuals at Risk for Esophageal Adenocarcinoma | Clinical Gastroenterology and Hepatology | 10.1016/j.cgh.2018.03.014 |
| **404** | L. S. Kuze, J. P. De Carli, J. S. Presotto, K. Collares and A. Della Bona | 2023 | Genotoxicity in the oral cells of older people from a Brazilian rural area: a population-based study | Brazilian Oral Research | 10.1590/1807-3107bor-2023.vol37.0067 |
| **405** | M. A. Laaksonen, S. Q. Li, K. Canfell, R. J. MacInnis, G. G. Giles, E. Banks, J. L. Byles, D. J. Magliano, J. E. Shaw, T. K. Gill, V. Hirani, R. G. Cumming, P. Mitchell, M. Bonello, C. M. Vajdic, B. A. Adelstein, A. W. Taylor, K. Price and P. A. F. C. c. Australian Canc | 2023 | The future burden of oesophageal and stomach cancers attributable to modifiable behaviours in Australia: a pooled cohort study | British Journal of Cancer | 10.1038/s41416-022-02104-x |
| **406** | J. Labenz, H. Koop, A. Tannapfel, R. Kiesslich and A. H. Hölscher | 2015 | The Epidemiology, Diagnosis, and Treatment of Barrett's Carcinoma | Deutsches Arzteblatt International | 10.3238/arztebl.2015.0224 |
| **407** | C. Lam, W. F. Liu, R. D. Bel, K. Chan, L. Miller, M. C. Brown, Z. Chen, D. Cheng, D. Patel, W. Xu, G. E. Darling and G. Liu | 2017 | Polymorphisms of the FOXF1 and MHC locus genes in individuals undergoing esophageal acid reflux assessments | Diseases of the Esophagus | 10.1111/dote.12456 |
| **408** | M. Latorre, A. M. da Silva, D. Chinzon, J. N. Eisig and T. R. P. Dias-Bastos | 2014 | Epidemiology of upper gastrointestinal symptoms in Brazil (EpiGastro): A population-based study according to sex and age group | World Journal of Gastroenterology | 10.3748/wjg.v20.i46.17388 |
| **409** | E. Lee, D. O. Stram, W. E. Ek, L. E. Onstad, S. MacGregor, P. Gharahkhani, W. M. Ye, J. Lagergren, N. J. Shaheen, L. J. Murray, L. J. Hardie, M. D. Gammon, W. H. Chow, H. A. Risch, D. A. Corley, D. M. Levine, D. C. Whiteman, L. Bernstein, N. C. Bird, T. L. Vaughan and A. H. Wu | 2015 | Pleiotropic Analysis of Cancer Risk Loci on Esophageal Adenocarcinoma Risk | Cancer Epidemiology Biomarkers & Prevention | 10.1158/1055-9965.Epi-15-0596 |
| **410** | H. K. Lee, M. J. Kwon, Y. J. Ra, H. S. Lee, H. S. Kim, E. S. Nam, S. J. Cho, H. R. Park, S. K. Min, J. Seo, J. Y. Choe, K. W. Min and S. Y. Kang | 2020 | Significance of druggable targets (PD-L1, KRAS, BRAF, PIK3CA, MSI, and HPV) on curatively resected esophageal squamous cell carcinoma | Diagnostic Pathology | 10.1186/s13000-020-01045-4 |
| **411** | H. W. Lee, D. Huang, W. K. Shin, K. de la Torre, M. Song, A. Shin, J. K. Lee and D. Kang | 2022 | Frequent low dose alcohol intake increases gastric cancer risk: the Health Examinees-Gem (HEXA-G) study | Cancer Biology & Medicine | 10.20892/j.issn.2095-3941.2021.0642 |
| **412** | J. H. Lee, E. Y. Kim, C. K. Park, S. Y. Lee, M. K. Lee, S. H. Yoon, J. E. Lee, S. H. Lee, S. J. Kim, S. Y. Lee, J. H. Lim, T. W. Jang, S. H. Jang, K. Y. Lee, S. H. Lee, S. H. Yang, D. W. Park, C. K. Park, H. S. Kang, C. D. Yeo, C. M. Choi and J. C. Lee | 2023 | Real-World Study of Osimertinib in Korean Patients with Epidermal Growth Factor Receptor T790M Mutation-Positive Non-Small Cell Lung Cancer | Cancer Research and Treatment | 10.4143/crt.2022.381 |
| **413** | K. Lee, J. S. Lee, J. Kim, H. Lee, Y. Chang, H. G. Woo, J. W. Kim and T. J. Song | 2020 | Oral health and gastrointestinal cancer: A nationwide cohort study | Journal of Clinical Periodontology | 10.1111/jcpe.13304 |
| **414** | W. Lee, J. Kim, S. S. Lim, Y. Kim, Y. S. Ahn and J. H. Yoon | 2020 | External Airborne-agent Exposure Increase Risk of Digestive Tract Cancer | Scientific Reports | 10.1038/s41598-020-65312-6 |
| **415** | Y. J. Lee, M. Redd, L. Bayman, N. Frederickson, J. Valestin and R. Schey | 2015 | Comparison of clinical features in patients with eosinophilic esophagitis living in an urban and rural environment | Diseases of the Esophagus | 10.1111/dote.12164 |
| **416** | H. L. Lehman, X. B. Yang, P. A. Welsh and D. B. Stairs | 2015 | p120-Catenin Down-Regulation and Epidermal Growth Factor Receptor Overexpression Results in a Transformed Epithelium That Mimics Esophageal Squamous Cell Carcinoma | American Journal of Pathology | 10.1016/j.ajpath.2014.09.008 |
| **417** | C. Lesseur, A. Ferreiro-Iglesias, J. D. McKay, Y. Bossé, M. Johansson, V. Gaborieau, M. T. Landi, D. C. Christiani, N. C. Caporaso, S. E. Bojesen, C. I. Amos, S. Shete, G. Liu, G. Rennert, D. Albanes, M. C. Aldrich, A. Tardon, C. Chen, L. Triantafillos, J. K. Field, M. D. Teare, L. A. Kiemeney, B. Diergaarde, R. L. Ferris, S. Zienolddiny, S. Lam, A. F. Olshan, M. C. Weissler, M. Lacko, A. Risch, H. Bickeböller, A. R. Ness, S. Thomas, L. Le Marchand, M. B. Schabath, V. Wünsch, E. H. Tajara, A. S. Andrew, G. M. Clifford, P. Lazarus, K. Grankvist, M. Johansson, S. Arnold, O. Melander, H. Brunnström, S. Boccia, G. Cadoni, W. Timens, M. Obeidat, X. J. Xiao, R. S. Houlston, R. Y. J. Hung and P. Brennan | 2021 | Genome-wide association meta-analysis identifies pleiotropic risk loci for aerodigestive squamous cell cancers | Plos Genetics | 10.1371/journal.pgen.1009254 |
| **418** | H. Y. Li, X. Z. Yang, A. Q. Zhang, G. Y. Liang, Y. Sun and J. Zhang | 2024 | Age-period-cohort analysis of incidence, mortality and disability-adjusted life years of esophageal cancer in global, regional and national regions from 1990 to 2019 | Bmc Public Health | 10.1186/s12889-024-17706-8 |
| **419** | J. Li, H. L. Xu, B. D. Yao, W. X. Li, H. Fang, D. L. Xu and Z. F. Zhang | 2020 | Environmental tobacco smoke and cancer risk, a prospective cohort study in a Chinese population | Environmental Research | 10.1016/j.envres.2020.110015 |
| **420** | J. X. Li, J. Chen and W. F. Tang | 2019 | The consensus of integrative diagnosis and treatment of acute pancreatitis-2017 | Journal of Evidence Based Medicine | 10.1111/jebm.12342 |
| **421** | M. D. Li, L. Qiu, G. S. Jia, R. Q. Guo and Q. B. Leng | 2020 | Single-cell expression profiles of <i>ACE2</i> and <i>TMPRSS2</i> reveals potential vertical transmission and fetus infection of SARS-CoV-2 | Aging-Us | 10.18632/aging.104015 |
| **422** | P. Li, J. Jing, W. J. Liu, J. Z. Wang, X. Qi and G. J. Zhang | 2023 | Spatiotemporal Patterns of Esophageal Cancer Burden Attributable to Behavioral, Metabolic, and Dietary Risk Factors From 1990 to 2019: Longitudinal Observational Study | Jmir Public Health and Surveillance | 10.2196/46051 |
| **423** | Q. M. Li, L. Y. Zhu, T. Wei, Z. P. Zang, X. R. Zhang, Y. J. Wang, R. Gao, Y. J. Zhang, X. T. Zheng and F. Liu | 2023 | Secular trends and attributable risk factors of esophageal cancer deaths among non-elderly adults based on Global Burden of Disease Study | Journal of Cancer Research and Clinical Oncology | 10.1007/s00432-023-05380-z |
| **424** | R. Q. Li, J. Y. Sun, T. Wang, L. H. Huang, S. W. Wang, P. L. Sun and C. H. Yu | 2022 | Comparison of Secular Trends in Esophageal Cancer Mortality in China and Japan during 1990-2019: An Age-Period-Cohort Analysis | International Journal of Environmental Research and Public Health | 10.3390/ijerph191610302 |
| **425** | S. B. Li, H. Chen, J. Y. Man, T. C. Zhang, X. L. Yin, Q. F. He, X. R. Yang and M. Lu | 2021 | Changing trends in the disease burden of esophageal cancer in China from 1990 to 2017 and its predicted level in 25 years | Cancer Medicine | 10.1002/cam4.3775 |
| **426** | S. Y. Li, Y. Yoshida, E. Kobayashi, A. Adachi, S. Hirono, T. Matsutani, S. Mine, T. Machida, M. Ohno, E. Nishi, Y. Maezawa, M. Takemoto, K. Yokote, K. Kitamura, M. Sumazaki, M. Ito, H. Shimada, H. Takizawa, K. Kashiwado, G. Tomiyoshi, N. Shinmen, R. Nakamura, H. Kuroda, X. M. Zhang, H. Wang, K. Goto, Y. Iwadate and T. Hiwasa | 2020 | Association between serum anti-ASXL2 antibody levels and acute ischemic stroke, acute myocardial infarction, diabetes mellitus, chronic kidney disease and digestive organ cancer, and their possible association with atherosclerosis and hypertension | International Journal of Molecular Medicine | 10.3892/ijmm.2020.4690 |
| **427** | W. Li, L. Y. Zhang, B. B. Guo, J. Q. Deng, S. Q. Wu, F. Li, Y. R. Wang, J. C. Lu and Y. F. Zhou | 2019 | Exosomal <i>FMR1-AS1</i> facilitates maintaining cancer stem-like cell dynamic equilibrium via TLR7/NFB/c-Myc signaling in female esophageal carcinoma | Molecular Cancer | 10.1186/s12943-019-0949-7 |
| **428** | X. X. Li, L. Yu, M. Fu, J. N. Yang and H. Y. Tan | 2024 | Perioperative Risk Factors for Postoperative Pulmonary Complications After Minimally Invasive Esophagectomy | International Journal of General Medicine | 10.2147/ijgm.S449530 |
| **429** | X. Y. Li, C. Q. Yu, Y. Guo, Z. Bian, Z. W. Shen, L. Yang, Y. P. Chen, Y. Y. Wei, H. Zhang, Z. Qiu, J. S. Chen, F. Chen, Z. M. Chen, J. Lv, L. M. Li and C. China Kadoorie Biobank | 2019 | Association between tea consumption and risk of cancer: a prospective cohort study of 0.5 million Chinese adults | European Journal of Epidemiology | 10.1007/s10654-019-00530-5 |
| **430** | Y. Li, A. J. Byun, J. K. Choe, S. H. Lu, D. Restle, T. Eguchi, K. S. Tan, J. Saini, J. Huang, G. Rocco, D. R. Jones, W. D. Travis and P. S. Adusumilli | 2023 | Micropapillary and Solid Histologic Patterns in N1 and N2 Lymph Node Metastases Are Independent Factors of Poor Prognosis in Patients With Stages II to III Lung Adenocarcinoma | Journal of Thoracic Oncology | 10.1016/j.jtho.2023.01.002 |
| **431** | Y. X. Li, Z. M. He, J. Wei, R. J. Xu, T. T. Liu, Z. H. Zhong, L. K. Liu, S. H. Liang, Y. Zheng, G. B. Chen, Z. Q. Lv, S. L. Huang, X. Chen, H. Sun and Y. W. Liu | 2024 | Long-term exposure to ambient fine particulate matter constituents and mortality from total and site-specific gastrointestinal cancer | Environmental Research | 10.1016/j.envres.2023.117927 |
| **432** | Y. Y. Li, R. Ghanbari, W. Pathmasiri, S. McRitchie, H. Poustchi, A. Shayanrad, G. Roshandel, A. Etemadi, J. D. Pollock, R. Malekzadeh and S. C. J. Sumner | 2020 | Untargeted Metabolomics: Biochemical Perturbations in Golestan Cohort Study Opium Users Inform Intervention Strategies | Frontiers in Nutrition | 10.3389/fnut.2020.584585 |
| **433** | Z. F. Liang, R. Wu, W. Xie, H. Geng, L. Zhao, C. F. Xie, J. S. Wu, S. S. Geng, X. T. Li, M. M. Zhu, W. W. Zhu, J. Y. Zhu, C. Huang, X. Ma, C. Y. Zhong and H. Y. Han | 2015 | Curcumin Suppresses MAPK Pathways to Reverse Tobacco Smoke-induced Gastric Epithelial-Mesenchymal Transition in Mice | Phytotherapy Research | 10.1002/ptr.5398 |
| **434** | K. M. Liao, C. H. Yu, Y. C. Wu, J. J. Wang, F. W. Liang and C. H. Ho | 2024 | Risk of Atrial Fibrillation in Patients with Different Cancer Types in Taiwan | Life-Basel | 10.3390/life14050621 |
| **435** | J. H. Lim, J. H. Song, S. J. Chung, G. E. Chung and J. S. Kim | 2021 | Characteristics of interval gastric neoplasms detected within two years after negative screening endoscopy among Koreans | Bmc Cancer | 10.1186/s12885-021-07929-y |
| **436** | C. Y. Lin, H. Y. Fang, C. L. Feng, C. C. Li and C. R. Chien | 2016 | Cost-effectiveness of neoadjuvant concurrent chemoradiotherapy versus esophagectomy for locally advanced esophageal squamous cell carcinoma: A population-based matched case-control study | Thoracic Cancer | 10.1111/1759-7714.12326 |
| **437** | L. F. Lin, Z. Y. Li, L. Yan, Y. L. Liu, H. J. Yang and H. Li | 2021 | Global, regional, and national cancer incidence and death for 29 cancer groups in 2019 and trends analysis of the global cancer burden, 1990-2019 | Journal of Hematology & Oncology | 10.1186/s13045-021-01213-z |
| **438** | T. F. Lin, C. L. Bi, Y. Song, H. Y. Guo, L. S. Liu, Z. Y. Zhou, B. Y. Wang, G. F. Tang, C. Z. Liu, Y. Yang, W. H. Ling, J. G. Yang, Y. M. Cui, C. G. Zhang, G. Li, J. A. Li, J. P. Li, Y. Zhang, Y. Huo, X. B. Wang, H. Zhang, X. H. Qin and X. P. Xu | 2021 | Plasma Magnesium Concentrations and Risk of Incident Cancer in Adults with Hypertension: A Nested Case-Control Study | Annals of Nutrition and Metabolism | 10.1159/000510214 |
| **439** | X. Q. Lin, L. Peng, X. J. Xu, Y. R. Chen, Y. L. Zhang and X. Huo | 2018 | Connecting gastrointestinal cancer risk to cadmium and lead exposure in the Chaoshan population of Southeast China | Environmental Science and Pollution Research | 10.1007/s11356-018-1914-5 |
| **440** | S. Lipka, A. Kumar and J. E. Richter | 2016 | Impact of Diagnostic Delay and Other Risk Factors on Eosinophilic Esophagitis Phenotype and Esophageal Diameter | Journal of Clinical Gastroenterology | 10.1097/mcg.0000000000000297 |
| **441** | B. D. Liu, S. C. Udemba, S. Saleh, H. Hill, G. Q. Song and R. Fass | 2023 | Raloxifene increases the risk of gastroesophageal reflux disease, Barrett's esophagus, and esophageal stricture in postmenopausal women with osteoporosis | Neurogastroenterology and Motility | 10.1111/nmo.14689 |
| **442** | G. Y. Liu, L. Peng, B. Liu, K. N. Wang and Y. T. Han | 2019 | Analysis of risk factors for pulmonary infection in patients with minimally invasive esophagectomy | Oncology Letters | 10.3892/ol.2019.9987 |
| **443** | J. J. Liu, D. M. Freedman, M. P. Little, M. M. Doody, B. H. Alexander, C. M. Kitahara, T. Lee, P. Rajaraman, J. S. Miller, D. M. Kampa, S. L. Simon, D. L. Preston and M. S. Linet | 2014 | Work history and mortality risks in 90 268 US radiological technologists | Occupational and Environmental Medicine | 10.1136/oemed-2013-101859 |
| **444** | J. W. Liu, Y. J. Chen, X. Y. Zhan, Y. F. Yu and H. R. Yao | 2022 | Effect of prior cancer history on survival of patients with esophageal carcinoma: a propensity score matching, population-based study | Journal of Thoracic Disease | 10.21037/jtd-21-1707 |
| **445** | L. L. Liu, C. Y. Huang, W. Liao, S. W. Chen and S. H. Cai | 2020 | Smoking behavior and smoking index as prognostic indicators for patients with esophageal squamous cell carcinoma who underwent surgery: A large cohort study in Guangzhou, China | Tobacco Induced Diseases | 10.18332/tid/117428 |
| **446** | Q. Liu, Y. Yang, X. S. Fan, X. Y. Xin, Q. Y. Pan, Y. H. Zhang, B. R. Liu and J. Wei | 2021 | Heterogeneity response to afatinib in gastric cancer patient with uncommon epidermal growth factor receptor (EGFR) mutations: a case report | Annals of Translational Medicine | 10.21037/atm-20-7312 |
| **447** | S. H. Liu, J. Y. Qian, Q. R. Li, D. H. Liu, B. Zhang and X. X. Chen | 2024 | Case Report: foetal gastroschisis with ideal pregnancy outcomes under multidisciplinary treatment management | Frontiers in Pediatrics | 10.3389/fped.2024.1358856 |
| **448** | S. Y. Liu, W. Chen, E. A. Chughtai, Z. Qiao, J. T. Jiang, S. M. Li, W. Zhang and J. Zhang | 2017 | <i>PIK3CA</i> gene mutations in Northwest Chinese esophageal squamous cell carcinoma | World Journal of Gastroenterology | 10.3748/wjg.v23.i14.2585 |
| **449** | W. J. Liu, J. M. Snell, W. R. Jeck, K. A. Hoadley, M. D. Wilkerson, J. S. Parker, N. Patel, Y. B. Mlombe, G. Mulima, N. G. Liomba, L. L. Wolf, C. G. Shores, S. Gopal and N. E. Sharpless | 2016 | Subtyping sub-Saharan esophageal squamous cell carcinoma by comprehensive molecular analysis | Jci Insight | 10.1172/jci.insight.88755 |
| **450** | X. D. Liu, X. R. Wang, S. H. Lin, X. Q. Lao, J. Zhao, Q. K. Song, X. F. Su and I. T. S. Yu | 2017 | Dietary patterns and the risk of esophageal squamous cell carcinoma: A population-based case control study in a rural population | Clinical Nutrition | 10.1016/j.clnu.2015.11.009 |
| **451** | X. L. Liu, R. C. Wang, Y. Y. Liu, H. Chen, C. Qi, L. W. Hu, J. Yi and W. Wang | 2021 | Risk prediction nomogram for major morbidity related to primary resection for esophageal squamous cancer | Medicine | 10.1097/md.0000000000026189 |
| **452** | X. X. Liu, M. G. Zhou, F. Wang, S. Mubarik, Y. F. Wang, R. T. Meng, F. Shi, H. Y. Wen and C. H. Yu | 2020 | Secular Trend of Cancer Death and Incidence in 29 Cancer Groups in China, 1990-2017: A Joinpoint and Age-Period-Cohort Analysis | Cancer Management and Research | 10.2147/cmar.S247648 |
| **453** | Y. Liu, H. J. Lai, R. Zhang, L. Xia and L. X. Liu | 2023 | Causal relationship between gastro-esophageal reflux disease and risk of lung cancer: insights from multivariable Mendelian randomization and mediation analysis | International Journal of Epidemiology | 10.1093/ije/dyad090 |
| **454** | Y. T. Liu, J. H. Lee, M. K. Tsai, J. C. C. Wei and C. P. Wen | 2022 | The effects of modest drinking on life expectancy and mortality risks: a population-based cohort study | Scientific Reports | 10.1038/s41598-022-11427-x |
| **455** | Z. J. Liu, L. Wei and H. G. Ding | 2022 | Clinical characteristics of reflux esophagitis among patients with liver cirrhosis: a case-control study | Scandinavian Journal of Gastroenterology | 10.1080/00365521.2021.2018489 |
| **456** | Z. Q. Liu, C. Q. Lin, C. Suo, R. J. Zhao, L. Jin, T. J. Zhang and X. D. Chen | 2022 | Metabolic dysfunction-associated fatty liver disease and the risk of 24 specific cancers | Metabolism-Clinical and Experimental | 10.1016/j.metabol.2021.154955 |
| **457** | W. C. Lo, C. C. Ku, S. T. Chiou, C. C. Chan, C. L. Chen, M. S. Lai and H. H. Lin | 2017 | Adult mortality of diseases and injuries attributable to selected metabolic, lifestyle, environmental, and infectious risk factors in Taiwan: a comparative risk assessment | Population Health Metrics | 10.1186/s12963-017-0134-4 |
| **458** | L. Long and K. F. Lai | 2019 | Characteristics of Chinese chronic cough patients | Pulmonary Pharmacology & Therapeutics | 10.1016/j.pupt.2019.101811 |
| **459** | E. Loots, B. Sartorius, T. E. Madiba, C. J. J. Mulder and D. L. Clarke | 2017 | Oesophageal squamous cell cancer in a South African tertiary hospital: a risk factor and presentation analysis | South African Journal of Surgery |  |
| **460** | A. B. Lopes, M. Metzdorf, L. Metzdorf, M. P. R. Sousa, C. Kavalco, A. Etemadi, N. R. Pritchett, G. Murphy, A. M. Calafat, C. C. Abnet, S. M. Dawsey and R. B. Fagundes | 2018 | Urinary Concentrations of Polycyclic Aromatic Hydrocarbon Metabolites in <i>Mate</i> Drinkers in Rio Grande do Sul, Brazil | Cancer Epidemiology Biomarkers & Prevention | 10.1158/1055-9965.Epi-17-0773 |
| **461** | G. López-Abente, N. Aragonés, B. Pérez-Gómez, M. Pollán, J. García-Pérez, R. Ramis and P. Fernández-Navarro | 2014 | Time trends in municipal distribution patterns of cancer mortality in Spain | Bmc Cancer | 10.1186/1471-2407-14-535 |
| **462** | L. L. Lu, C. S. Mullins, C. Schafmayer, S. Zeissig and M. Linnebacher | 2021 | A global assessment of recent trends in gastrointestinal cancer and lifestyle-associated risk factors | Cancer Communications | 10.1002/cac2.12220 |
| **463** | P. P. Lu, J. H. Gu, N. Zhang, Y. W. Sun and J. L. Wang | 2020 | Risk factors for precancerous lesions of esophageal squamous cell carcinoma in high-risk areas of rural China A population-based screening study | Medicine | 10.1097/md.0000000000021426 |
| **464** | D. J. Lubin, R. Mick, S. G. Shroff, K. Stashek and E. E. Furth | 2018 | The notch pathway is activated in neoplastic progression in esophageal squamous cell carcinoma | Human Pathology | 10.1016/j.humpath.2017.11.004 |
| **465** | I. Lund and J. Scheffels | 2014 | Perceptions of Relative Risk of Disease and Addiction From Cigarettes and Snus | Psychology of Addictive Behaviors | 10.1037/a0032657 |
| **466** | L. Lundell, J. Hatlebakk, J. P. Galmiche, S. E. Attwood, C. Ell, R. Fiocca, T. Persson, P. Nagy, S. Eklund and T. Lind | 2015 | Long-term effect on symptoms and quality of life of maintenance therapy with esomeprazole 20 mg daily: a <i>post hoc</i> analysis of the LOTUS trial | Current Medical Research and Opinion | 10.1185/03007995.2014.980500 |
| **467** | C. Ma, S. E. Congly, D. E. Chyou, K. Ross-Driscoll, N. Forbes, E. S. Tsang, D. A. Sussman and D. S. Goldberg | 2022 | Factors Associated With Geographic Disparities in Gastrointestinal Cancer Mortality in the United States | Gastroenterology | 10.1053/j.gastro.2022.04.019 |
| **468** | M. Ma, S. Shroff, M. Feldman, M. DeMarshall, C. Price, A. Tierney and G. W. Falk | 2017 | Risk of malignant progression in Barrett's esophagus indefinite for dysplasia | Diseases of the Esophagus | 10.1093/dote/dow025 |
| **469** | M. J. Machiela, C. A. Hsiung, X. O. Shu, W. J. Seow, Z. M. Wang, K. Matsuo, Y. C. Hong, A. Seow, C. Wu, H. D. Hosgood, K. X. Chen, J. C. Wang, W. Q. Wen, R. Cawthon, N. Chatterjee, W. Hu, N. E. Caporaso, J. Y. Park, C. J. Chen, Y. H. Kim, Y. T. Kim, M. T. Landi, H. B. Shen, C. Lawrence, L. Burdett, M. Yeager, I. S. Chang, T. Mitsudomi, H. N. Kim, G. C. Chang, B. A. Bassig, M. Tucker, F. S. Wei, Z. H. Yin, S. J. An, B. Y. Qian, V. H. F. Lee, D. R. Lu, J. J. Liu, H. S. Jeon, C. F. Hsiao, J. S. Sung, J. H. Kim, Y. T. Gao, Y. H. Tsai, Y. J. Jung, H. Guo, Z. B. Hu, A. Hutchinson, W. C. Wang, R. J. Klein, C. C. Chung, I. J. Oh, K. Y. Chen, S. I. Berndt, W. Wu, J. Chang, X. C. Zhang, M. S. Huang, H. Zheng, J. W. Wang, X. Y. Zhao, Y. Q. Li, J. E. Choi, W. C. Su, K. H. Park, S. W. Sung, Y. M. Chen, L. Liu, C. H. Kang, L. M. Hu, C. H. Chen, W. Pao, Y. C. Kim, T. Y. Yang, J. Xu, P. Guan, W. Tan, J. Su, C. L. Wang, H. X. Li, A. D. L. Sihoe, Z. H. Zhao, Y. Chen, Y. Y. Choi, J. Y. Hung, J. S. Kim, H. I. Yoon, Q. Y. Cai, C. C. Lin, I. K. Park, P. Xu, J. Dong, C. Kim, Q. C. He, R. P. Perng, T. Kohno, S. S. Kweon, C. Y. Chen, R. C. H. Vermeulen, J. J. Wu, W. Y. Lim, K. C. Chen, W. H. Chow, B. T. Ji, J. K. C. Chan, M. J. Chu, Y. J. Li, J. Yokota, J. H. Li, H. Y. Chen, Y. B. Xiang, C. J. Yu, H. Kunitoh, G. P. Wu, L. Jin, Y. L. Lo, K. Shiraishi, Y. H. Chen, H. C. Lin, T. C. Wu, M. P. Wong, Y. L. Wu, P. C. Yang, B. S. Zhou, M. H. Shin, J. F. Fraumeni, W. Zheng, D. X. Lin, S. J. Chanock, N. Rothman and Q. Lan | 2015 | Genetic variants associated with longer telomere length are associated with increased lung cancer risk among never-smoking women in Asia: a report from the female lung cancer consortium in Asia | International Journal of Cancer | 10.1002/ijc.29393 |
| **470** | J. S. MacLeod, M. A. Harris, M. Tjepkema, P. A. Peters and P. A. Demers | 2017 | Cancer Risks among Welders and Occasional Welders in a National Population-Based Cohort Study: Canadian Census Health and Environmental Cohort | Safety and Health at Work | 10.1016/j.shaw.2016.12.001 |
| **471** | S. Maeda, K. Mure, K. Mugitani, Y. Watanabe, M. Iwane, O. Mohara and T. Takeshita | 2014 | Roles of the ALDH2 and ADH1B Genotypes on the Association Between Alcohol Intake and Serum Adiponectin Levels Among Japanese Male Workers | Alcoholism-Clinical and Experimental Research | 10.1111/acer.12406 |
| **472** | F. B. Maguire, A. S. Movsisyan, C. R. Morris, A. Parikh-Patel, T. H. M. Keegan and E. K. Tong | 2022 | Evaluation of Cancer Deaths Attributable to Tobacco in California, 2014-2019 | Jama Network Open | 10.1001/jamanetworkopen.2022.46651 |
| **473** | R. Mahfouz, A. Barchuk, A. E. Obeidat, M. M. Mansour, D. Hernandez, M. Darweesh, M. Aldiabat, M. H. Al-Khateeb, M. H. Yusuf and Y. Aljabiri | 2022 | The Relationship Between Obstructive Sleep Apnea (OSA) and Gastroesophageal Reflux Disease (GERD) in Inpatient Settings: A Nationwide Study | Cureus Journal of Medical Science | 10.7759/cureus.22810 |
| **474** | K. R. Majeed, W. Y. Al-Ani and M. M. AlShock | 2022 | Study the effect of Sex hormones in patients with stomach ulcers in Anbar Governorate | Journal of Pharmaceutical Negative Results | 10.47750/pnr.2022.13.S01.218 |
| **475** | A. F. T. Malekshah, M. Zaroudi, A. Etemadi, F. Islami, S. Sepanlou, M. Sharafkhah, A. A. Keshtkar, H. Khademi, H. Poustchi, A. Hekmatdoost, A. Pourshams, A. F. Sani, E. Jafari, F. Kamangar, S. M. Dawsey, C. C. Abnet, P. D. Pharoah, P. J. Berennan, P. Boffetta, A. Esmaillzadeh and R. Malekzadeh | 2016 | The Combined Effects of Healthy Lifestyle Behaviors on All-Cause Mortality: The Golestan Cohort Study | Archives of Iranian Medicine |  |
| **476** | Z. Malik, L. Bayman, J. Valestin, A. Rizvi-Toner, S. Hashmi and R. Schey | 2017 | Dronabinol increases pain threshold in patients with functional chest pain: a pilot double-blind placebo-controlled trial | Diseases of the Esophagus | 10.1111/dote.12455 |
| **477** | S. Manavalan, B. Getachew, K. F. Manaye, S. J. Khundmiri, A. B. Csoka, R. McKinley, A. Tamas, D. Reglodi and Y. Tizabi | 2017 | PACAP Protects Against Ethanol and Nicotine Toxicity in SH-SY5Y Cells: Implications for Drinking-Smoking Co-morbidity | Neurotoxicity Research | 10.1007/s12640-017-9727-8 |
| **478** | F. Mano, K. Ikeda, T. Sato, T. Nakayama, D. Tanaka, E. Joo, Y. Takahashi, S. Kosugi, A. Sekine, Y. Tabara, F. Matsuda, N. Inagaki and G. Nagahama Study | 2018 | Reduction in Gastroesophageal Reflux Disease Symptoms Is Associated with <i>Miso</i> Soup Intake in a Population-Based Cross-Sectional Study: The Nagahama Study | Journal of Nutritional Science and Vitaminology | 10.3177/jnsv.64.367 |
| **479** | N. M. Mansour, H. B. El-Serag and S. Anandasabapathy | 2017 | Barrett's esophagus: best practices for treatment and post-treatment surveillance | Annals of Cardiothoracic Surgery | 10.21037/acs.2017.03.05 |
| **480** | A. Mantovani, G. Petracca, G. Beatrice, A. Csermely, H. Tilg, C. D. Byrne and G. Targher | 2022 | Non-alcoholic fatty liver disease and increased risk of incident extrahepatic cancers: a meta-analysis of observational cohort studies | Gut | 10.1136/gutjnl-2021-324191 |
| **481** | S. Mantziari, A. Pomoni, J. O. Prior, M. Winiker, P. Allemann, N. Demartines and M. Schäfer | 2020 | <SUP>18</SUP>F- FDG PET/CT-derived parameters predict clinical stage and prognosis of esophageal cancer | Bmc Medical Imaging | 10.1186/s12880-019-0401-x |
| **482** | N. Mao, S. Y. Nie, B. Hong, C. Li, X. Y. Shen and T. Xiong | 2016 | Association between alcohol dehydrogenase-2 gene polymorphism and esophageal cancer risk: a meta-analysis | World Journal of Surgical Oncology | 10.1186/s12957-016-0937-y |
| **483** | J. Maret-Ouda, G. Santoni, S. H. Xie, A. Rosengren and J. Lagergren | 2022 | Proton Pump Inhibitor and Clopidogrel Use After Percutaneous Coronary Intervention and Risk of Major Cardiovascular Events | Cardiovascular Drugs and Therapy | 10.1007/s10557-021-07219-6 |
| **484** | M. Mariani, R. Pastorino, D. P. Marafon, K. C. Johnson, J. F. Hu, A. J. M. de la Torre, G. Fernández-Tardón, D. Zaridze, D. Maximovich, E. Negri, C. La Vecchia, Z. F. Zhang, R. C. Kurtz, C. Pelucchi, M. Rota and S. Boccia | 2023 | Leisure-time physical activity and gastric cancer risk: A pooled study within the Stomach cancer Pooling (StoP) Project | Plos One | 10.1371/journal.pone.0286958 |
| **485** | I. Marijanovic, M. Kraljevic, D. B. Glibo, T. Buhovac and E. C. Obrdalj | 2021 | THE ROLE OF FAMILY PHYSICIANS IN THE PREVENTION AND EARLY DETECTION OF CANCER IN HERZEGOVINA-NERETVA AND WEST-HERZEGOVINA CANTON | Psychiatria Danubina |  |
| **486** | T. A. Markel, C. Proctor, J. Ying and P. D. Winchester | 2015 | Environmental pesticides increase the risk of developing hypertrophic pyloric stenosis | Journal of Pediatric Surgery | 10.1016/j.jpedsurg.2014.12.009 |
| **487** | M. A. Marks and E. A. Engels | 2014 | Venous Thromboembolism and Cancer Risk among Elderly Adults in the United States | Cancer Epidemiology Biomarkers & Prevention | 10.1158/1055-9965.Epi-13-1138 |
| **488** | G. Martimianaki, P. Bertuccio, G. Alicandro, C. Pelucchi, F. Bravi, G. Carioli, R. Bonzi, C. S. Rabkin, L. M. Liao, R. Sinha, K. Johnson, J. F. Hu, D. Palli, M. Ferraroni, N. Lunet, S. Morais, S. Tsugane, A. Hidaka, G. S. Hamada, L. López-Carrillo, R. U. Hernández-Ramírez, D. Zaridze, D. Maximovitch, N. Aragonés, V. Martin, M. H. Ward, J. Vioque, M. G. de la Hera, Z. F. Zhang, R. C. Kurtz, P. Lagiou, A. Lagiou, A. Trichopoulou, A. Karakatsani, R. Malekzadeh, M. C. Camargo, M. P. Curado, S. Boccia, P. Boffetta, E. Negri and C. La Vecchia | 2022 | Coffee consumption and gastric cancer: a pooled analysis from the Stomach cancer Pooling Project consortium | European Journal of Cancer Prevention | 10.1097/cej.0000000000000680 |
| **489** | M. C. L. Martins, D. L. Miyazaki, C. C. T. Gabiatti, L. P. Silva, L. T. Macedo, N. S. Siqueira, N. A. Andreollo and J. B. C. Carvalheira | 2019 | Chagasic Megaesophagus-Associated Carcinoma: Clinical Pattern and Outcomes | Journal of Global Oncology | 10.1200/jgo.19.00143 |
| **490** | M. Matejcic, C. G. Mathew and M. I. Parker | 2019 | The Relationship Between Environmental Exposure and Genetic Architecture of the 2q33 Locus With Esophageal Cancer in South Africa | Frontiers in Genetics | 10.3389/fgene.2019.00406 |
| **491** | K. Matsueda, R. Ishihara, T. Morishima, Y. Okubo, Y. Kawakami, H. Sakurai, T. Nakamura, Y. Tani, M. Miyake, S. Shichijo, A. Maekawa, T. Kanesaka, S. Yamamoto, Y. Takeuchi, K. Higashino, N. Uedo, T. Michida, T. Matsunaga, Y. Ohno, T. Sobue and I. Miyashiro | 2022 | Impact of endoscopic surveillance on mortality of metachronous esophageal and head and neck cancer after esophageal endoscopic resection | Journal of Gastroenterology and Hepatology | 10.1111/jgh.15984 |
| **492** | K. Matsuo, S. W. Lee, R. Tanaka, Y. Imai, K. Honda, K. Taniguchi, H. Tomiyama and K. Uchiyama | 2021 | T stage and venous invasion are crucial prognostic factors for long-term survival of patients with remnant gastric cancer: a cohort study | World Journal of Surgical Oncology | 10.1186/s12957-021-02400-5 |
| **493** | J. A. McDonald and L. J. Paulozzi | 2019 | Parsing the Paradox: Hispanic Mortality in the US by Detailed Cause of Death | Journal of Immigrant and Minority Health | 10.1007/s10903-018-0737-2 |
| **494** | D. M. McElvenny, B. G. Miller, L. A. MacCalman, A. Sleeuwenhoek, M. van Tongeren, K. Shepherd, A. J. Darnton and J. W. Cherrie | 2015 | Mortality of a cohort of workers in Great Britain with blood lead measurements | Occupational and Environmental Medicine | 10.1136/oemed-2014-102637 |
| **495** | D. M. McElvenny, W. Mueller, P. Ritchie, J. W. Cherrie, M. Hidajat, A. J. Darnton, R. M. Agius and F. de Vocht | 2018 | British rubber and cable industry cohort: 49-year mortality follow-up | Occupational and Environmental Medicine | 10.1136/oemed-2017-104834 |
| **496** | G. A. Medhanie, S. A. Fedewa, H. Adissu, C. E. DeSantis, R. L. Siegel and A. Jemal | 2017 | Cancer Incidence Profile in Sub-Saharan African-Born Blacks in the United States: Similarities and Differences With US-Born Non-Hispanic Blacks | Cancer | 10.1002/cncr.30701 |
| **497** | Y. Mei, D. Liang, T. J. Wang and D. Yu | 2021 | Gaining insights into relevance across cancers based on mutation features of TP53 gene | Biochemistry and Biophysics Reports | 10.1016/j.bbrep.2021.101165 |
| **498** | D. Menya, N. Kigen, M. Oduor, S. K. Maina, F. Some, D. Chumba, P. Ayuo, O. Osano, D. R. S. Middleton, J. Schüz and V. A. McCormack | 2019 | Traditional and commercial alcohols and esophageal cancer risk in Kenya | International Journal of Cancer | 10.1002/ijc.31804 |
| **499** | T. J. Meyers, S. C. Chang, P. Y. Chang, H. Morgenstern, D. P. Tashkin, J. Y. Rao, W. Cozen, T. M. Mack and Z. F. Zhang | 2017 | Case-control study of cumulative cigarette tar exposure and lung and upper aerodigestive tract cancers | International Journal of Cancer | 10.1002/ijc.30632 |
| **500** | D. S. Michaud, K. T. Kelsey, E. Papathanasiou, C. A. Genco and E. Giovannucci | 2016 | Periodontal disease and risk of all cancers among male never smokers: an updated analysis of the Health Professionals Follow-up Study | Annals of Oncology | 10.1093/annonc/mdw028 |
| **501** | D. R. S. Middleton, D. Menya, N. Kigen, M. Oduor, S. K. Maina, F. Some, D. Chumba, P. Ayuo, O. Osano, J. Schüz and V. McCormack | 2019 | Hot beverages and oesophageal cancer risk in western Kenya: Findings from the ESCCAPE case-control study | International Journal of Cancer | 10.1002/ijc.32032 |
| **502** | S. Mignozzi, C. Santucci, H. N. Medina, E. Negri, C. La Vecchia and P. S. Pinheiro | 2024 | Cancer mortality in Germany-born Americans and Germans | Cancer Epidemiology | 10.1016/j.canep.2023.102519 |
| **503** | S. S. Mitter, R. Vedanthan, F. Islami, A. Pourshams, H. Khademi, F. Kamangar, C. C. Abnet, S. M. Dawsey, P. D. Pharoah, P. Brennan, V. Fuster, P. Boffetta and R. Malekzadeh | 2016 | Household Fuel Use and Cardiovascular Disease Mortality Golestan Cohort Study | Circulation | 10.1161/circulationaha.115.020288 |
| **504** | B. T. Mmbaga, A. Mwasamwaja, G. Mushi, A. Mremi, G. Nyakunga, I. Kiwelu, R. Swai, G. Kiwelu, S. Mustapha, E. Mghase, A. McHome, R. D. Shao, E. Mallya, D. S. Rwakatema, K. Kilonzo, O. M. Munishi, B. Abedi-Ardekani, D. Middleton, J. Schuz and V. McCormack | 2021 | Missing and decayed teeth, oral hygiene and dental staining in relation to esophageal cancer risk: ESCCAPE case-control study in Kilimanjaro, Tanzania | International Journal of Cancer | 10.1002/ijc.33433 |
| **505** | F. Mohammadzadeh, H. Noorkojuri, M. A. Pourhoseingholi, S. Saadat and A. R. Baghestani | 2015 | Predicting the probability of mortality of gastric cancer patients using decision tree | Irish Journal of Medical Science | 10.1007/s11845-014-1100-9 |
| **506** | N. Mohy-ud-Din, T. S. Krill, A. R. Shah, A. T. Chatila, S. Singh, M. Bilal and S. Parupudi | 2020 | Barrett's esophagus: What do we need to know? | Dm Disease-a-Month | 10.1016/j.disamonth.2019.02.003 |
| **507** | Y. Mok, D. K. Son, Y. D. Yun, S. H. Jee and J. M. Samet | 2016 | γ-Glutamyltransferase and cancer risk: The Korean cancer prevention study | International Journal of Cancer | 10.1002/ijc.29659 |
| **508** | A. J. Montiel-Jarquín, L. G. V. de Lara-Cisneros, A. López-Colombo, H. A. Solís-Mendoza, M. L. Palmer-Márquez and M. S. Romero-Figueroa | 2019 | Expression of metalloproteinase-9 in patients with mild and severe forms of gastroesophageal reflux disease | Cirugia Y Cirujanos | 10.24875/ciru.18000691 |
| **509** | S. C. Moore, I. M. Lee, E. Weiderpass, P. T. Campbell, J. N. Sampson, C. M. Kitahara, S. K. Keadle, H. Arem, A. B. de Gonzalez, P. Hartge, H. O. Adami, C. K. Blair, K. B. Borch, E. Boyd, D. P. Check, A. Fournier, N. D. Freedman, M. Gunter, M. Johannson, K. T. Khaw, M. S. Linet, N. Orsini, Y. Park, E. Riboli, K. Robien, C. Schairer, H. Sesso, M. Spriggs, R. Van Dusen, A. Wolk, C. E. Matthews and A. V. Patel | 2016 | Association of Leisure-Time Physical Activity With Risk of 26 Types of Cancer in 1.44 Million Adults | Jama Internal Medicine | 10.1001/jamainternmed.2016.1548 |
| **510** | S. Moossavi, M. Mohamadnejad, A. Pourshams, H. Poustchi, F. Islami, M. Sharafkhah, B. Mirminachi, S. Nasseri-Moghaddam, S. Semnani, R. Shakeri, A. Etemadi, S. Merat, M. Khoshnia, S. M. Dawsey, P. D. Pharoah, P. Brennan, C. C. Abnet, P. Boffetta, F. Kamangar and R. Malekzadeh | 2018 | Opium Use and Risk of Pancreatic Cancer: A Prospective Cohort Study | Cancer Epidemiology Biomarkers & Prevention | 10.1158/1055-9965.Epi-17-0592 |
| **511** | J. M. Mora-Luján, A. Iriarte, E. Alba, M. A. Sánchez-Corral, A. Berrozpe, P. Cerdà, F. Cruellas, J. Ribas, J. Castellote and A. Riera-Mestre | 2020 | Gastrointestinal Bleeding in Patients with Hereditary Hemorrhagic Telangiectasia: Risk Factors and Endoscopic Findings | Journal of Clinical Medicine | 10.3390/jcm9010082 |
| **512** | S. Morais, B. Peleteiro, N. Araújo, R. Malekzadeh, W. M. Ye, A. Plymoth, S. Tsugane, A. Hidaka, G. S. Hamada, L. López-Carrillo, D. Zaridze, D. Maximovich, N. Aragonés, G. Castaño-Vinyals, M. Pakseresht, R. U. Hernández-Ramírez, M. López-Cervantes, M. Leja, E. Gasenko, F. Pourfarzi, Z. F. Zhang, G. P. Yu, M. H. Derakhshan, C. Pelucchi, E. Negri, C. La Vecchia and N. Lunet | 2022 | Identifying the Profile of <i>Helicobacter pylori</i>-Negative Gastric Cancers: A Case-Only Analysis within the Stomach Cancer Pooling (StoP) Project | Cancer Epidemiology Biomarkers & Prevention | 10.1158/1055-9965.Epi-21-0402 |
| **513** | C. T. Morgan, J. P. Kanne, E. E. Lewis, J. D. Maloney, M. M. DeCamp and D. P. McCarthy | 2023 | One hundred cases of primary spontaneous pneumomediastinum: leukocytosis is common, pleural effusions and age over 40 are rare | Journal of Thoracic Disease | 10.21037/jtd-22-1136 |
| **514** | J. G. Morland, P. Magnus, S. E. Vollset, D. A. Leon, R. Selmer and A. Tverdal | 2023 | Associations between serum high-density lipoprotein cholesterol levels and cause-specific mortality in a general population of 345 000 men and women aged 20-79 years | International Journal of Epidemiology | 10.1093/ije/dyad011 |
| **515** | J. N. Morris, J. Loyer and J. Blunt | 2024 | Stigma, risks, and benefits of medicinal cannabis use among Australians with cancer | Supportive Care in Cancer | 10.1007/s00520-024-08439-w |
| **516** | M. J. Morris, R. J. Walter, E. T. McCann, J. H. Sherner, C. G. Murillo, B. S. Barber, J. C. Hunninghake and A. B. Holley | 2020 | Clinical Evaluation of Deployed Military Personnel With Chronic Respiratory Symptoms Study of Active Duty Military for Pulmonary Disease Related to Environmental Deployment Exposures (STAMPEDE) III | Chest | 10.1016/j.chest.2020.01.024 |
| **517** | N. Mostafalou, Y. Yahyapour, S. Sedaghat, J. S. Shirvani, M. HajiAhmadi, S. Siadati and S. Shafaei | 2015 | Human papilloma virus infection in non-cancerous versus normal esophageal tissue samples by endoscopy | Caspian Journal of Internal Medicine |  |
| **518** | L. C. Murnane, A. K. Forsyth, J. Koukounaras, C. H. C. Pilgrim, K. Shaw, W. A. Brown, M. Mourtzakis, A. C. Tierney and P. R. Burton | 2021 | Low muscularity increases the risk for post-operative pneumonia and delays recovery from complications after oesophago-gastric cancer resection | Anz Journal of Surgery | 10.1111/ans.17203 |
| **519** | M. F. Mushi, N. Ngeta, M. M. Mirambo and S. E. Mshana | 2018 | Predictors of esophageal candidiasis among patients attending endoscopy unit in a tertiary hospital, Tanzania: a retrospective cross-sectional study | African Health Sciences | 10.4314/ahs.v18i1.10 |
| **520** | M. M. Mwachiro, N. Pritchett, A. M. Calafat, R. K. Parker, J. O. Lando, G. Murphy, R. Chepkwony, S. L. Burgert, C. C. Abnet, M. D. Topazian, R. E. White, S. M. Dawsey and A. Etemadi | 2021 | Indoor wood combustion, carcinogenic exposure and esophageal cancer in southwest Kenya | Environment International | 10.1016/j.envint.2021.106485 |
| **521** | D. Mysíková, I. Adkins, N. Hradilová, O. Palata, J. Simonek, J. Pozniak, J. Kolarík, A. Skallová-Fialová, R. Spísek and R. Lischke | 2017 | Case-Control Study: Smoking History Affects the Production of Tumor Antigen-Specific Antibodies NY-ESO-1 in Patients with Lung Cancer in Comparison with Cancer Disease-Free Group | Journal of Thoracic Oncology | 10.1016/j.jtho.2016.09.136 |
| **522** | D. Myti, M. Gunjak, F. Casado, S. K. Raziabad, C. Nardiello, I. Vadász, S. Herold, G. Pryhuber, W. Seeger and R. E. Morty | 2020 | Elevated FiO<sub>2</sub> increases SARS-CoV-2 co-receptor expression in respiratory tract epithelium | American Journal of Physiology-Lung Cellular and Molecular Physiology | 10.1152/ajplung.00345.2020 |
| **523** | N. Nagata, T. Nishijima, R. Niikura, T. Yokoyama, Y. Matsushita, K. Watanabe, K. Teruya, Y. Kikuchi, J. Akiyama, M. Yanase, N. Uemura, S. Oka and H. Gatanaga | 2018 | Increased risk of non-AIDS-defining cancers in Asian HIV-infected patients: a long-term cohort study | Bmc Cancer | 10.1186/s12885-018-4963-8 |
| **524** | G. Nagel, M. Stafoggia, M. Pedersen, Z. J. Andersen, C. Galassi, J. Munkenast, A. Jaensch, J. Sommar, B. Forsberg, D. Olsson, B. Oftedal, N. H. Krog, G. Aamodt, A. Pyko, G. Pershagen, M. Korek, U. De Faire, N. L. Pedersen, C. G. Östenson, L. Fratiglioni, M. Sorensen, A. Tjonneland, P. H. Peeters, B. Bueno-de-Mesquita, R. Vermeulen, M. Eeftens, M. Plusquin, T. J. Key, H. Concin, A. Lang, M. Wang, M. Y. Tsai, S. Grioni, A. Marcon, V. Krogh, F. Ricceri, C. Sacerdote, A. Ranzi, G. Cesaroni, F. Forastiere, I. Tamayo-Uria, P. Amiano, M. Dorronsoro, K. de Hoogh, R. Beelen, P. Vineis, B. Brunekreef, G. Hoek, O. Raaschou-Nielsen and G. Weinmayr | 2018 | Air pollution and incidence of cancers of the stomach and the upper aerodigestive tract in the European Study of Cohorts for Air Pollution Effects (ESCAPE) | International Journal of Cancer | 10.1002/ijc.31564 |
| **525** | A. Naghibzadeh-Tahami, M. Marzban, V. Yazdi-Feyzabadi, S. Dabiri, S. Mohseni, R. A. Rayeni, M. S. Fekri, M. H. Larizadeh, B. Karimpour and N. Khanjani | 2020 | Is opium use associated with an increased risk of lung cancer? A case-control study | Bmc Cancer | 10.1186/s12885-020-07296-0 |
| **526** | A. Naghibzadeh-Tahami, M. Marzban, V. Yazdi-Feyzabadi, Z. Khazaei, M. J. Zahedi, V. Moazed and A. A. Haghdoost | 2021 | Opium use as an independent risk factor for pancreatic cancer: A case-control study | Cancer Epidemiology | 10.1016/j.canep.2021.102017 |
| **527** | M. Nalini, E. Oranuba, H. Poustchi, S. G. Sepanlou, A. Pourshams, M. Khoshnia, A. Gharavi, S. M. Dawsey, C. C. Abnet, P. Boffetta, P. Brennan, M. Sotoudeh, A. Nikmanesh, S. Merat, A. Etemadi, R. Shakeri, A. A. Sohrabpour, S. Nasseri-Moghaddam, F. Kamangar and R. Malekzadeh | 2018 | Causes of premature death and their associated risk factors in the Golestan Cohort Study, Iran | Bmj Open | 10.1136/bmjopen-2018-021479 |
| **528** | M. Nalini, S. G. Sepanlou, A. Pourshams, H. Poustchi, M. Sharafkhah, H. Bahrami, F. Kamangar and R. Malekzadeh | 2018 | Drug Use for Secondary Prevention of Cardiovascular Diseases in Golestan, Iran: Results From the Golestan Cohort Study | Archives of Iranian Medicine |  |
| **529** | S. Y. Nam, J. Jo, S. W. Jeon and H. Chun | 2023 | Sex-specific effects of fruit, vegetable, and red meat intake on the risk of gastric and esophageal cancer in a large cohort | Digestive and Liver Disease | 10.1016/j.dld.2023.02.021 |
| **530** | M. C. Narendra, B. Ramakrishna, Y. Mutheeswaraiah, N. Rukmangadha, K. A. Sarma and A. M. Pavan | 2022 | A Retrospective Clinical Study of Gastroduodenal Perforation Peritonitis | Journal of Research in Medical and Dental Science |  |
| **531** | C. T. Narh, C. P. Dzamalala, B. T. Mmbaga, D. Menya, Y. Mlombe, P. Finch, G. Nyakunga, J. Schüz, V. McCormack and E. Team | 2021 | Geophagia and risk of squamous cell esophageal cancer in the African esophageal cancer corridor: Findings from the ESCCAPE multicountry case-control studies | International Journal of Cancer | 10.1002/ijc.33688 |
| **532** | U. Nasir, B. Rodgers, D. Panchal, C. Choi, S. Ahmed and S. Ahlawat | 2020 | Ferrous Sulfate-Induced Esophageal Injury Leading to Esophagitis Dissecans Superficialis | Case Reports in Gastroenterology | 10.1159/000506935 |
| **533** | M. Nasrazadani, M. R. Maracy, E. Dreassi and B. Mahaki | 2018 | Mapping of Stomach, Colorectal, and Bladder Cancers in Iran, 2004-2009: Applying Bayesian Polytomous Logit Model | International Journal of Preventive Medicine | 10.4103/ijpvm.IJPVM_30_17 |
| **534** | D. Nasrollahzadeh, G. Roshandel, T. M. Delhomme, P. H. Avogbe, M. Foll, F. Saidi, H. Poustchi, M. Sotoudeh, R. Malekzadeh, P. Brennan, J. McKay, P. Hainaut and B. Abedi-Ardekani | 2021 | <i>TP53</i> Targeted Deep Sequencing of Cell-Free DNA in Esophageal Squamous Cell Carcinoma Using Low-Quality Serum: Concordance with Tumor Mutation | International Journal of Molecular Sciences | 10.3390/ijms22115627 |
| **535** | D. Nasrollahzadeh, W. M. Ye, R. Shakeri, M. Sotoudeh, S. Merat, F. Kamangar, C. C. Abnet, F. Islami, P. Boffetta, S. M. Dawsey, P. Brennan and R. Malekzadeh | 2015 | Contact with ruminants is associated with esophageal squamous cell carcinoma risk | International Journal of Cancer | 10.1002/ijc.29109 |
| **536** | A. Nath, K. SathishKumar, P. Das, S. K. Lakshminarayana, S. Santhappan, S. Natarajan, S. Karuppasamy, S. Narasimhan, R. Venkataiah and P. Mathur | 2023 | Need for accelerating tobacco control in India: findings from the national cancer registry programme | European Journal of Cancer Prevention | 10.1097/cej.0000000000000759 |
| **537** | F. Navab, B. H. Nathanson and D. J. Desilets | 2015 | The impact of lifestyle on Barrett's Esophagus: A precursor to esophageal adenocarcinoma | Cancer Epidemiology | 10.1016/j.canep.2015.10.013 |
| **538** | C. Nelson, J. Lee, K. Ko, A. G. Sikora, M. D. Bonnen, P. Enkhbaatar and Y. T. Ghebre | 2017 | Therapeutic Efficacy of Esomeprazole in Cotton Smoke-Induced Lung Injury Model | Frontiers in Pharmacology | 10.3389/fphar.2017.00016 |
| **539** | S. Nemati, E. Saeedi, F. Lotfi, A. Nahvijou, E. Mohebbi, Z. Ravankhah, A. Rezaeianzadeh, M. Yaghoobi-Ashrafi, H. Pirnejad, A. Golpazir, R. Dolatkhah, S. Alvand, S. V. Ahmadi-Tabatabaei, M. Cheraghi, E. Weiderpass, F. Bray, M. P. Coleman, A. Etemadi, A. Khosravi, F. Najafi, M. A. Mohagheghi, G. Roshandel, R. Malekzadeh and K. Zendehdel | 2022 | National surveillance of cancer survival in Iran (IRANCANSURV): Analysis of data of 15 cancer sites from nine population-based cancer registries | International Journal of Cancer | 10.1002/ijc.34224 |
| **540** | E. Ness-Jensen, A. Hammer and L. A. Hopstock | 2023 | Trends in gastro-oesophageal reflux in a Norwegian general population: the Tromso Study 1979-2016 | Scandinavian Journal of Gastroenterology | 10.1080/00365521.2023.2183733 |
| **541** | E. Ness-Jensen, K. Hveem, H. El-Serag and J. Lagergren | 2016 | Lifestyle Intervention in Gastroesophageal Reflux Disease | Clinical Gastroenterology and Hepatology | 10.1016/j.cgh.2015.04.176 |
| **542** | E. Ness-Jensen and J. Lagergren | 2017 | Tobacco smoking, alcohol consumption and gastro-oesophageal reflux disease | Best Practice & Research Clinical Gastroenterology | 10.1016/j.bpg.2017.09.004 |
| **543** | E. Ness-Jensen, G. Santoni, E. Gottlieb-Vedi, A. Lindam, N. Pedersen and J. Lagergren | 2020 | Mortality in gastro-oesophageal reflux disease in a population-based nationwide cohort study of Swedish twins | Bmj Open | 10.1136/bmjopen-2020-037456 |
| **544** | N. L. T. Nguyen, N. D. T. Dang, Q. H. Dang, V. C. Tran, H. L. Vo, M. Yamaguchi and T. V. Ta | 2021 | Polymorphism of <i>MUC1</i> Gene in Vietnamese Gastric Cancer Patients: A Multicenter Case-Control Study | Frontiers in Oncology | 10.3389/fonc.2021.694977 |
| **545** | N. L. T. Nguyen, N. D. T. Dang, Q. V. Vu, A. K. Dang and T. V. Ta | 2023 | A Model for Gastric Cancer Risk Prediction Based on<i> MUC1</i> Polymorphisms and Health-risk Behaviors in a Vietnamese Population | In Vivo | 10.21873/invivo.13339 |
| **546** | T. Nguyen, Z. W. Tang, M. Younes, A. Alsarraj, D. Ramsey, S. Fitzgerald, J. R. Kramer and H. B. El-Serag | 2015 | Esophageal COX-2 Expression Is Increased in Barrett's Esophagus, Obesity, and Smoking | Digestive Diseases and Sciences | 10.1007/s10620-014-3333-x |
| **547** | T. X. T. Nguyen, M. Han and J. K. Oh | 2019 | The economic burden of cancers attributable to smoking in Korea, 2014 | Tobacco Induced Diseases | 10.18332/tid/102673 |
| **548** | R. Nikkilä, E. Hirvonen, A. Haapaniemi, L. Tapiovaara, J. Pitkäniemi, N. Malila and A. Mäkitie | 2023 | Significant risk of second primary cancer among laryngeal squamous cell carcinoma patients even after 20 years | Acta Oncologica | 10.1080/0284186x.2023.2254482 |
| **549** | R. Nikkilä, E. Hirvonen, J. Pitkäniemi, J. Räsänen, N. K. Malila and A. Makitie | 2024 | Risk of Second Primary Cancer Among Patients with Cardio-Esophageal Cancer in Finland: A Nationwide Population-Based Study | Clinical Epidemiology | 10.2147/clep.S471802 |
| **550** | R. Nikkilä, M. Peltomaa, T. Carpén, J. I. Martinsen, S. Heikkinen, J. Selander, I. S. Mehlum, J. E. Torfadottir, A. Mäkitie and E. Pukkala | 2023 | Cancer incidence among visual artists: 45 years of follow-up in four Nordic countries | Acta Oncologica | 10.1080/0284186x.2023.2263150 |
| **551** | A. N. Niles and A. O'Donovan | 2019 | Comparing Anxiety and Depression to Obesity and Smoking as Predictors of Major Medical Illnesses and Somatic Symptoms | Health Psychology | 10.1037/hea0000707 |
| **552** | F. L. Ning, J. Lyu, J. P. Pei, W. J. Gu, N. N. Zhang, S. Y. Cao, Y. J. Zeng, M. Abe, K. Nishiyama and C. D. Zhang | 2022 | The burden and trend of gastric cancer and possible risk factors in five Asian countries from 1990 to 2019 | Scientific Reports | 10.1038/s41598-022-10014-4 |
| **553** | T. Nishino, T. Yoshida, S. Inoue, S. Fujiwara, M. Goto, T. Minato, Y. Furukita, Y. Yamamoto, Y. Yuasa, H. Yamai, H. Takechi, H. Toba, H. Takizawa, M. Yoshida, J. Seike, T. Miyoshi and A. Tangoku | 2017 | Gender differences in clinicopathological features and prognosis of squamous cell carcinoma of the esophagus | Esophagus | 10.1007/s10388-016-0554-4 |
| **554** | T. B. Nobel, J. Livschitz, M. Eljalby, Y. Y. Janjigian, M. S. Bains, P. S. Adusumilli, D. R. Jones and D. Molena | 2020 | Unique Considerations for Females Undergoing Esophagectomy | Annals of Surgery | 10.1097/sla.0000000000003202 |
| **555** | A. M. Noone, K. A. Cronin, S. F. Altekruse, N. Howlader, D. R. Lewis, V. I. Petkov and L. Penberthy | 2017 | Cancer Incidence and Survival Trends by Subtype Using Data from the Surveillance Epidemiology and End Results Program, 1992-2013 | Cancer Epidemiology Biomarkers & Prevention | 10.1158/1055-9965.Epi-16-0520 |
| **556** | R. Nopour | 2023 | Prediction of five-year survival among esophageal cancer patients using machine learning | Heliyon | 10.1016/j.heliyon.2023.e22654 |
| **557** | G. Ntentas, K. Dedeckova, M. Andrlik, M. C. Aznar, R. Shakir, J. Ramroth, R. Begum, J. Kubes, S. C. Darby, N. G. Mikhaeel and D. J. Cutter | 2022 | Proton Therapy in Supradiaphragmatic Lymphoma: Predicting Treatment-Related Mortality to Help Optimize Patient Selection | International Journal of Radiation Oncology Biology Physics | 10.1016/j.ijrobp.2021.10.151 |
| **558** | N. N. Nwizu, J. R. Marshall, K. Moysich, R. J. Genco, K. M. Hovey, X. D. Mai, M. J. LaMonte, J. L. Freudenheim and J. Wactawski-Wende | 2017 | Periodontal Disease and Incident Cancer Risk among Postmenopausal Women: Results from the Women's Health Initiative Observational Cohort | Cancer Epidemiology Biomarkers & Prevention | 10.1158/1055-9965.Epi-17-0212 |
| **559** | A. H. Nyberg, E. Sadikova, C. Cheetham, K. M. Chiang, J. X. X. Shi, S. Caparosa, Z. M. Younossi and L. M. Nyberg | 2020 | Increased cancer rates in patients with chronic hepatitis C | Liver International | 10.1111/liv.14305 |
| **560** | S. Obayo, Y. Mulumba, C. L. Thompson, M. K. Gibson, M. M. Cooney and J. Orem | 2023 | Clinicopathological characteristics and treatment outcomes of esophageal cancer patients in Uganda | Ecancermedicalscience | 10.3332/ecancer.2023.1576 |
| **561** | A. A. Ocampo, R. M. Genta and E. S. Dellon | 2023 | Mast Cell Esophagitis: A Novel Entity in Patients with Unexplained Esophageal Symptoms | Dysphagia | 10.1007/s00455-023-10616-8 |
| **562** | S. Ohashi, T. Maruno, K. Fukuyama, O. Kikuchi, T. Sunami, Y. Kondo, S. Imai, A. Matsushima, K. Suzuki, F. Usui, M. Yakami, A. Yamada, H. Isoda, S. Matsumoto, H. Seno, M. Muto and M. Inoue | 2021 | Visceral fat obesity is the key risk factor for the development of reflux erosive esophagitis in 40-69-years subjects | Esophagus | 10.1007/s10388-021-00859-5 |
| **563** | E. Okada, S. Ukawa, K. Nakamura, M. Hirata, A. Nagai, K. Matsuda, T. Ninomiya, Y. Kiyohara, K. Muto, Y. Kamatani, Z. Yamagata, M. Kubo, Y. Nakamura, A. Tamakoshi and G. BioBank Japan Cooperative Hosp | 2017 | Demographic and lifestyle factors and survival among patients with esophageal and gastric cancer: The Biobank Japan Project | Journal of Epidemiology | 10.1016/j.je.2016.12.002 |
| **564** | F. Okada | 2014 | Inflammation-Related Carcinogenesis: Current Findings in Epidemiological Trends, Causes and Mechanisms | Yonago Acta Medica |  |
| **565** | M. Okuyama, O. Takaishi, K. Nakahara, N. Iwakura, T. Hasegawa, M. Oyama, A. Inoue, H. Ishizu, H. Satoh and Y. Fujiwara | 2017 | Associations among gastroesophageal reflux disease, psychological stress, and sleep disturbances in Japanese adults | Scandinavian Journal of Gastroenterology | 10.1080/00365521.2016.1224383 |
| **566** | G. H. Oliveira-Paula, L. C. Pinheiro and J. E. Tanus-Santos | 2019 | Mechanisms impairing blood pressure responses to nitrite and nitrate | Nitric Oxide-Biology and Chemistry | 10.1016/j.niox.2019.01.015 |
| **567** | J. S. Ong, J. Y. An, X. K. Han, M. H. Law, P. Nandakumar, J. Schumacher, I. Gockel, A. Bohmer, J. Jankowski, C. Palles, C. M. Olsen, R. E. Neale, R. Fitzgerald, A. P. Thrift, T. L. Vaughan, M. F. Buas, D. A. Hinds, P. Gharahkhani, B. J. Kendall, S. MacGregor, T. andMe Res and C. Esophageal Canc | 2022 | Multitrait genetic association analysis identifies 50 new risk loci for gastro-oesophageal reflux, seven new loci for Barrett's oesophagus and provides insights into clinical heterogeneity in reflux diagnosis | Gut | 10.1136/gutjnl-2020-323906 |
| **568** | J. S. Ong, P. Gharahkhani, T. L. Vaughan, D. Whiteman, B. J. Kendall and S. MacGregor | 2022 | Assessing the genetic relationship between gastro-esophageal reflux disease and risk of COVID-19 infection | Human Molecular Genetics | 10.1093/hmg/ddab253 |
| **569** | A. Örmeci, B. Çavu, R. Akas, Z. Istemihan, Z. Imanov, V. Senkal, K. Nuriyev, A. Bayraktar, C. B. Külle, M. Keskin, K. Demir, F. Besisik, S. Kaymakoglu and F. Akyüz | 2022 | What is the effect of subepithelial lesions of the esophagus on esophageal motility? | European Review for Medical and Pharmacological Sciences |  |
| **570** | H. A. Osman, S. S. Aly, H. S. Mahmoud, E. H. Ahmed, E. M. S. Eldin, E. A. Abdelrahim, M. A. El Masry, R. A. Herdan and M. H. Hassan | 2019 | Effect of Acid Suppression on Peripheral T-Lymphocyte Subsets and Immunohistochemical Esophageal Mucosal Changes in Patients With Gastroesophageal Reflux Disease | Journal of Clinical Gastroenterology | 10.1097/mcg.0000000000001098 |
| **571** | P. Özdemir, M. Erdinç, R. Vardar, A. Veral, S. Akyildiz, Ö. Özdemir and S. Bor | 2017 | The Role of Microaspiration in the Pathogenesis of Gastroesophageal Reflux-related Chronic Cough | Journal of Neurogastroenterology and Motility | 10.5056/jnm16057 |
| **572** | D. Pan, M. Su, T. Zhang, C. Y. Miao, L. M. Fu, L. G. Yang, G. Song, P. J. Raine, S. K. Wang and G. J. Sun | 2019 | A Distinct Epidemiologic Pattern of Precancerous Lesions of Esophageal Squamous Cell Carcinoma in a High-risk Area of Huai'an, Jiangsu Province, China | Cancer Prevention Research | 10.1158/1940-6207.Capr-18-0462 |
| **573** | D. Pan, G. J. Sun, M. Su, X. Wang, Q. Y. Yan, G. Song, Y. Y. Wang, D. F. Xu, N. N. Wang and S. K. Wang | 2022 | Inverse relations between <i>Helicobacter pylori</i> infection and risk of esophageal precancerous lesions in drinkers and peanut consumption | World Journal of Gastrointestinal Oncology | 10.4251/wjgo.v14.i9.1689 |
| **574** | K. F. Pan, L. Zhang, M. Gerhard, J. L. Ma, W. D. Liu, K. Ulm, J. X. Wang, L. Zhang, Y. Zhang, M. Bajbouj, L. F. Zhang, M. Li, M. Vieth, R. Y. Liu, M. Quante, L. H. Wang, S. Suchanek, T. Zhou, W. X. Guan, R. Schmid, M. Classen and W. C. You | 2016 | A large randomised controlled intervention trial to prevent gastric cancer by eradication of <i>Helicobacter pylori</i> in Linqu County, China: baseline results and factors affecting the eradication | Gut | 10.1136/gutjnl-2015-309197 |
| **575** | A. Pandey, S. C. Tripathi, S. Mahata, K. Vishnoi, S. Shukla, S. P. Misra, V. Misra, S. Hedau, R. Mehrotra, M. Dwivedi and A. C. Bharti | 2014 | Carcinogenic <i>Helicobacter pylori</i> in gastric pre-cancer and cancer lesions: Association with tobacco-chewing | World Journal of Gastroenterology | 10.3748/wjg.v20.i22.6860 |
| **576** | K. Parikh and L. Khaitan | 2016 | Radiofrequency ablation coupled with Roux-en-Y gastric bypass: a treatment option for morbidly obese patients with Barrett's esophagus | Journal of Surgical Case Reports | 10.1093/jscr/rjw007 |
| **577** | E. Park, H. Y. Kang, M. K. Lim, B. Kim and J. K. Oh | 2024 | Cancer Risk Following Smoking Cessation in Korea | Jama Network Open | 10.1001/jamanetworkopen.2023.54958 |
| **578** | G. W. Park, S. K. Kim, C. H. Lee, C. R. Kim, H. J. Jeong and D. K. Kim | 2015 | Effect of Chronic Obstructive Pulmonary Disease on Swallowing Function in Stroke Patients | Annals of Rehabilitation Medicine-Arm | 10.5535/arm.2015.39.2.218 |
| **579** | J. H. Park, J. Y. Hong, J. J. Shen, K. Han, J. O. Park, Y. S. Park and H. Y. Lim | 2023 | Increased Risk of Young-Onset Digestive Tract Cancers Among Young Adults Age 20-39 Years With Nonalcoholic Fatty Liver Disease: A Nationwide Cohort Study | Journal of Clinical Oncology | 10.1200/jco.22.01740 |
| **580** | W. Park, J. K. Lee, C. R. Kim and J. Y. Shin | 2015 | Factors Associated with Fatigue in Korean Gastric Cancer Survivors | Korean Journal of Family Medicine | 10.4082/kjfm.2015.36.6.328 |
| **581** | D. Pasquier, B. Bataille, F. Le Tinier, R. Bennadji, H. Langin, A. Escande, E. Tresch, F. Darloy, D. Carlier, F. Crop and E. Lartigau | 2021 | Correlation between toxicity and dosimetric parameters for adjuvant intensity modulated radiation therapy of breast cancer: a prospective study | Scientific Reports | 10.1038/s41598-021-83159-3 |
| **582** | S. Pasricha, A. Gupta, C. C. Reed, O. Speck, J. T. Woosley and E. S. Dellon | 2016 | Lymphocytic Esophagitis: An Emerging Clinicopathologic Disease Associated with Dysphagia | Digestive Diseases and Sciences | 10.1007/s10620-016-4230-2 |
| **583** | T. S. Pasricha and B. Kochar | 2021 | Vaping-associated esophagitis | Bmc Gastroenterology | 10.1186/s12876-021-01695-8 |
| **584** | A. W. Pastuszak, N. Thirumavalavan, T. P. Kohn, L. I. Lipshultz and M. L. Eisenberg | 2019 | Increased Risk of Cancer in Men With Peyronie's Disease: A Cohort Study Using a Large United States Insurance Claims Database | Sexual Medicine | 10.1016/j.esxm.2019.08.007 |
| **585** | J. Patel, T. Khanna, A. Sohal, A. Dhaliwal, H. Chaudhry, S. Kalra, I. Singh, D. Dukovic and K. Bains | 2024 | Impact of aspirin use on rates of metastasis in patients with esophageal cancer: insights from the National Inpatient Sample | Diseases of the Esophagus | 10.1093/dote/doae022 |
| **586** | K. A. Patterson, P. J. Roberts-Thomson, S. Lester, J. A. Tan, P. Hakendorf, M. Rischmueller, J. Zochling, J. Sahhar, P. Nash, J. Roddy, C. Hill, M. Nikpour, W. Stevens, S. M. Proudman and J. G. Walker | 2015 | Interpretation of an Extended Autoantibody Profile in a Well-Characterized Australian Systemic Sclerosis (Scleroderma) Cohort Using Principal Components Analysis | Arthritis & Rheumatology | 10.1002/art.39316 |
| **587** | G. Paul, W. Bohle and W. Zoller | 2019 | Risk Factors for the Development of Esophagorespiratory Fistula in Esophageal Cancer | Journal of Gastrointestinal and Liver Diseases | 10.15403/jgld-271 |
| **588** | M. W. Pawlik, S. Kwiecien, R. Pajdo, A. Ptak-Belowska, B. Brzozowski, G. Krzysiek-Maczka, M. Strzalka, S. J. Konturek and T. Brzozowski | 2014 | ESOPHAGOPROTECTIVE ACTIVITY OF ANGIOTENSIN-(1-7) IN EXPERIMENTAL MODEL OF ACUTE REFLUX ESOPHAGITIS. EVIDENCE FOR THE ROLE OF NITRIC OXIDE, SENSORY NERVES, HYPDXIA-INDUCIBLE FACTOR-1ALPHA AND PROINFLAMMATORY CYTOKINES | Journal of Physiology and Pharmacology |  |
| **589** | J. Y. Peng, Y. H. Yu, W. M. Chen, B. Shia, M. Chen and S. Y. Wu | 2023 | Association of Antihistamine Use with Increased Risk of Esophageal Squamous Cell Carcinoma: A Nationwide, Long-Term Follow-Up Study Using Propensity Score Matching | Biomedicines | 10.3390/biomedicines11020578 |
| **590** | A. Perisetti and P. Sharma | 2023 | A Practical Approach to Diagnosis and Treatment of Barrett's Esophagus | Practical Gastroenterology |  |
| **591** | S. Peter, A. Pendergraft, W. VanderPol, C. M. Wilcox, K. Baig, C. Morrow, J. Izard and P. J. Mannon | 2020 | Mucosa-Associated Microbiota in Barrett's Esophagus, Dysplasia, and Esophageal Adenocarcinoma Differ Similarly Compared With Healthy Controls | Clinical and Translational Gastroenterology |  |
| **592** | B. A. Peters, J. Wu, Z. H. Pei, L. Y. Yang, M. P. Purdue, N. D. Freedman, E. J. Jacobs, S. M. Gapstur, R. B. Hayes and J. Ahn | 2017 | Oral Microbiome Composition Reflects Prospective Risk for Esophageal Cancers | Cancer Research | 10.1158/0008-5472.Can-17-1296 |
| **593** | J. L. Petrick, N. Li, L. A. Anderson, L. Bernstein, D. A. Corley, H. B. El Serag, S. Hardikar, L. M. Liao, G. Liu, L. J. Murray, J. H. Rubenstein, J. L. Schneider, N. J. Shaheen, A. P. Thrift, P. A. van den Brandt, T. L. Vaughan, D. C. Whiteman, A. H. Wu, W. K. Zhao, M. D. Gammon and M. B. Cook | 2019 | Diabetes in relation to Barrett's esophagus and adenocarcinomas of the esophagus: A pooled study from the International Barrett's and Esophageal Adenocarcinoma Consortium | Cancer | 10.1002/cncr.32444 |
| **594** | B. L. Phelps, Y. M. Tiley, J. L. Skrove, A. C. Berry and K. Mohan | 2019 | Acute Dysphagia Caused by sarcomatoid Squamous Cell Carcinoma of the Esophagus | Cureus Journal of Medical Science | 10.7759/cureus.4129 |
| **595** | J. L. Pierce, K. Tanner, R. M. Merrill, K. L. Miller, K. A. Kendall and N. Roy | 2016 | Swallowing Disorders in Sjogren's Syndrome: Prevalence, Risk Factors, and Effects on Quality of Life | Dysphagia | 10.1007/s00455-015-9657-7 |
| **596** | S. D. Pinto, L. H. D. Rodriguez, F. R. Takeda, M. R. Tacconi, R. A. A. Sallum, I. Cecconello and U. Ribeiro | 2022 | CHARACTERIZATION OF THE PSYCHOLOGICAL TYPOLOGY IN ESOPHAGEAL CANCER PATIENTS | Abcd-Arquivos Brasileiros De Cirurgia Digestiva-Brazilian Archives of Digestive Surgery | 10.1590/0102-672020220002e1715 |
| **597** | V. A. Pinto, E. Nascimento, A. P. L. Cunha, B. P. S. Assis, M. F. Lasmar, H. R. Vianna and R. A. Fabreti-Oliveira | 2022 | Malignancy Diseases in Kidney Transplantation, Clinical Outcomes, Patient, and Allograft Survival: A Case-Control Study | Transplantation Proceedings | 10.1016/j.transproceed.2022.02.063 |
| **598** | A. Poosari, T. Nutravong, P. Sa-ngiamwibool, W. Namwat, S. Chatrchaiwiwatana and P. Ungareewittaya | 2021 | Association between infection with <i>Campylobacter</i> species, poor oral health and environmental risk factors on esophageal cancer: a hospital-based case-control study in Thailand | European Journal of Medical Research | 10.1186/s40001-021-00561-3 |
| **599** | S. J. Pournaghi, S. K. Hojjat, F. B. Noveyri, H. T. Ghouchani, A. Ahmadi, A. Hamedi, J. Rahimi, H. Mohamaddoust and H. Lashkardoost | 2019 | Tobacco consumption, opium use, alcohol drinking and the risk of esophageal cancer in North Khorasan, Iran | Journal of Substance Use | 10.1080/14659891.2018.1523962 |
| **600** | A. Prabhu, K. Obi, D. Lieberman and J. H. Rubenstein | 2016 | The Race-Specific Incidence of Esophageal Squamous Cell Carcinoma in Individuals With Exposure to Tobacco and Alcohol | American Journal of Gastroenterology | 10.1038/ajg.2016.346 |
| **601** | D. Praud, P. Bertuccio, C. Bosetti, F. Turati, M. Ferraroni and C. La Vecchia | 2014 | Adherence to the Mediterranean diet and gastric cancer risk in Italy | International Journal of Cancer | 10.1002/ijc.28620 |
| **602** | D. Praud, M. Rota, C. Pelucchi, P. Bertuccio, T. Rosso, C. Galeone, Z. F. Zhang, K. Matsuo, H. Ito, J. F. Hu, K. C. Johnson, G. P. Yu, D. Palli, M. Ferraroni, J. Muscat, N. Lunet, B. Peleteiro, R. Malekzadeh, W. M. Ye, H. Song, D. Zaridze, D. Maximovitch, N. Aragonés, G. Castaño-Vinyals, J. Vioque, E. M. Navarrete-Muñoz, M. Pakseresht, F. Pourfarzi, A. Wolk, N. Orsini, A. Bellavia, N. Håkansson, L. N. Mu, R. Pastorino, R. C. Kurtz, M. H. Derakhshan, A. Lagiou, P. Lagiou, P. Boffetta, S. Boccia, E. Negri and C. La Vecchia | 2018 | Cigarette smoking and gastric cancer in the Stomach Cancer Pooling (StoP) Project | European Journal of Cancer Prevention | 10.1097/cej.0000000000000290 |
| **603** | E. Pukkala, M. Peltomaa, A. Mäkitie, S. Heikkinen, K. Kjærheim, J. I. Martinsen, P. Sparén, L. Tryggvadottir and E. Weiderpass | 2021 | Cancer incidence among musicians: 45 years of follow-up in four Nordic countries | Acta Oncologica | 10.1080/0284186x.2021.1924403 |
| **604** | M. A. Qayyum, M. H. Sultan, Z. Farooq, K. Muddassir, T. Farooq and A. Irfan | 2022 | Quantitative estimation of essential/toxic elemental levels in the serum of esophagus cancer patients in relation to controls | Environmental Science and Pollution Research | 10.1007/s11356-022-21651-7 |
| **605** | H. Qiu, X. T. Lin, W. F. Tang, C. Liu, Y. Chen, H. Ding, M. Q. Kang and S. C. Chen | 2017 | Investigation of <i>TCF7L2</i>, <i>LEP</i> and <i>LEPR</i> polymorphisms with esophageal squamous cell carcinomas | Oncotarget | 10.18632/oncotarget.22619 |
| **606** | H. Qiu, Y. F. Wang, M. Q. Kang, H. Ding, C. Liu, W. F. Tang, Z. Z. Xiao and Y. Chen | 2017 | The relationship between <i>IGF2BP2</i> and <i>PPARG</i> polymorphisms and susceptibility to esophageal squamous-cell carcinomas in the eastern Chinese Han population | Oncotargets and Therapy | 10.2147/ott.S145776 |
| **607** | H. B. Qiu, S. M. Cao and R. H. Xu | 2021 | Cancer incidence, mortality, and burden in China: a time-trend analysis and comparison with the United States and United Kingdom based on the global epidemiological data released in 2020 | Cancer Communications | 10.1002/cac2.12197 |
| **608** | G. R. Quinn, D. Ranum, E. Song, M. Linets, C. Keohane, H. Riah and P. Greenberg | 2017 | Missed Diagnosis of Cardiovascular Disease in Outpatient General Medicine: Insights from Malpractice Claims Data | Joint Commission Journal on Quality and Patient Safety | 10.1016/j.jcjq.2017.05.001 |
| **609** | H. K. Quintana, V. Herrera, C. Niño, B. Gómez and R. Roa | 2019 | Assessing the knowledge, attitudes and perceptions of tobacco-associated diseases and how it is influenced by tobacco products advertisement, promotion and sponsorship while enforcing a strong and comprehensive ban in Panama: a cross-sectional study | Bmj Open | 10.1136/bmjopen-2018-024373 |
| **610** | B. Qumseya, S. Yang and Y. Guo | 2024 | Trends in prevalence of esophageal adenocarcinoma: Findings from a statewide database of over 6 million patients | Endoscopy International Open | 10.1055/a-2221-7974 |
| **611** | B. Rabiee, N. Motamed, V. Hosseini, G. R. Hemasi, M. Maadi and F. Zamani | 2016 | Gastro esophageal reflux disease (GERD) prevalence and related risk factors in north of Iran | Esophagus | 10.1007/s10388-016-0536-6 |
| **612** | L. Radulovic, J. Erakovic and M. Roganovic | 2020 | Attitudes of patients with relapsing-remitting form of multiple sclerosis using disease-modifying drugs in Montenegro regarding COVID-19 pandemic | Multiple Sclerosis and Related Disorders | 10.1016/j.msard.2020.102380 |
| **613** | R. Rafiq, I. A. Shah, G. A. Bhat, M. M. Lone, F. Islami, P. Boffetta and N. A. Dar | 2016 | Secondhand Smoking and the Risk of Esophageal Squamous Cell Carcinoma in a High Incidence Region, Kashmir, India <i>A Case</i>-<i>control</i>-<i>observational Study</i> | Medicine | 10.1097/md.0000000000002340 |
| **614** | M. M. Rahman, M. A. Sarker, M. M. Hossain, M. S. Alam, M. Islam, L. Shirin, R. Sultana and G. N. N. Sultana | 2019 | Association of <i>p53</i> Gene Mutation With <i>Helicobacter pylori</i> Infection in Gastric Cancer Patients and Its Correlation With Clinicopathological and Environmental Factors | World Journal of Oncology | 10.14740/wjon1087 |
| **615** | M. G. Rajanandh, S. Suresh, K. Manobala, R. Nandhakumar, G. Jaswanthi and S. Neha | 2018 | Prediction of cardiovascular risk in cancer patients of South India using WHO/ISH risk prediction charts and Framingham score - A prospective study | Journal of Oncology Pharmacy Practice | 10.1177/1078155217707334 |
| **616** | J. M. Ramsay, M. J. Madsen, J. J. Horns, H. A. Hanson, N. J. Camp, B. R. Emery, K. Aston, E. Ferlic and J. M. Hotaling | 2024 | Describing patterns of familial cancer risk in subfertile men using population pedigree data | Human Reproduction | 10.1093/humrep/dead270 |
| **617** | M. Rastogi, D. Rastogi, S. Singh, A. Agarwal, B. P. Priyadarshi and T. Middha | 2015 | Prevalence of <i>Helicobacter pylori</i> in asymptomatic adult patients in a tertiary care hospital: A cross sectional study | Biomedical Research-India |  |
| **618** | A. C. F. Ratin and I. R. B. Orso | 2015 | MINIMAL ENDOSCOPIC CHANGES IN NON-EROSIVE REFLUX DISEASE | Abcd-Arquivos Brasileiros De Cirurgia Digestiva-Brazilian Archives of Digestive Surgery | 10.1590/s0102-67202015000100006 |
| **619** | M. Ravanbakhsh, H. Yousefi, E. Lak, M. J. Ansari, W. Suksatan, Q. A. Qasim, P. Asban, M. Kianizadeh and M. J. Mohammadi | 2023 | Effect of Polycyclic Aromatic Hydrocarbons (PAHs) on Respiratory Diseases and the Risk Factors Related to Cancer | Polycyclic Aromatic Compounds | 10.1080/10406638.2022.2149569 |
| **620** | A. S. Reece and G. K. Hulse | 2022 | Geotemporospatial and causal inferential epidemiological overview and survey of USA cannabis, cannabidiol and cannabinoid genotoxicity expressed in cancer incidence 2003-2017: part 1-continuous bivariate analysis | Archives of Public Health | 10.1186/s13690-022-00811-8 |
| **621** | A. S. Reece and G. K. Hulse | 2022 | Epidemiology of Δ8THC-Related Carcinogenesis in USA: A Panel Regression and Causal Inferential Study | International Journal of Environmental Research and Public Health | 10.3390/ijerph19137726 |
| **622** | A. S. Reece and G. K. Hulse | 2022 | Geotemporospatial and causal inferential epidemiological overview and survey of USA cannabis, cannabidiol and cannabinoid genotoxicity expressed in cancer incidence 2003-2017: part 2-categorical bivariate analysis and attributable fractions | Archives of Public Health | 10.1186/s13690-022-00812-7 |
| **623** | A. S. Reece and G. K. Hulse | 2023 | Congenital Gastrointestinal Anomalies in Europe 2010-2019: A Geo-Spatiotemporal and Causal Inferential Study of Epidemiological Patterns in Relationship to Cannabis- and Substance Exposure | Gastroenterology Insights | 10.3390/gastroent14010007 |
| **624** | J. Rehm, I. Soerjomataram, C. Ferreira-Borges and K. D. Shield | 2019 | Does Alcohol Use Affect Cancer Risk? | Current Nutrition Reports | 10.1007/s13668-019-0267-0 |
| **625** | Z. W. Reichenbach, J. Sloan, A. Rizvi-Toner, L. Bayman, J. Valestin and R. Schey | 2015 | A 4-week Pilot Study With the Cannabinoid Receptor Agonist Dronabinol and Its Effect on Metabolic Parameters in a Randomized Trial | Clinical Therapeutics | 10.1016/j.clinthera.2015.07.023 |
| **626** | C. Ren, X. Y. Cai, M. Z. Qiu, D. S. Wang, F. H. Wang, H. Y. Luo and R. H. Xu | 2015 | Impact of body mass index on survival of esophageal squamous carcinoma patients in southern China | Journal of Thoracic Disease | 10.3978/j.issn.2072-1439.2014.10.12 |
| **627** | F. P. Ren, Z. L. Shi, X. Shen, G. F. Xiao, C. Y. Zhang and Y. Q. Cheng | 2024 | The global, regional, and national burden of stomach cancer attributed to smoking in 204 countries, 1990-2019: A systematic analysis for the Global Burden of Disease Study 2019 | Tobacco Induced Diseases | 10.18332/tid/183803 |
| **628** | L. Renaud, M. N. Hilleret, E. Thimonier, O. Guillaud, F. Arbib, G. Ferretti, A. Jankowski, C. Chambon-Augoyard, D. Erard-Poinsot, T. Decaens, O. Boillot, V. Leroy and J. Dumortier | 2018 | De Novo Malignancies Screening After Liver Transplantation for Alcoholic Liver Disease: A Comparative Opportunistic Study | Liver Transplantation | 10.1002/lt.25336 |
| **629** | K. Repp, R. Lorbeer, T. Ittermann, S. Gläser, U. John, W. Hoffmann and H. Vötlzke | 2015 | OCCUPATIONAL EXPOSURE TO ASBESTOS IS ASSOCIATED WITH INCREASED MORTALITY IN MEN RECRUITED FOR A POPULATION-BASED STUDY IN GERMANY | International Journal of Occupational Medicine and Environmental Health | 10.13075/ijomeh.1896.00549 |
| **630** | D. B. Richardson, E. Rage, P. A. Demers, M. T. Do, N. DeBono, N. Fenske, V. Deffner, M. Kreuzer, J. Samet, C. Wiggins, M. K. Schubauer-Berigan, K. Kelly-Reif, L. Tomasek, L. B. Zablotska and D. Laurier | 2021 | Mortality among uranium miners in North America and Europe: the Pooled Uranium Miners Analysis (PUMA) | International Journal of Epidemiology | 10.1093/ije/dyaa195 |
| **631** | J. E. Richter and J. H. Rubenstein | 2018 | Presentation and Epidemiology of Gastroesophageal Reflux Disease | Gastroenterology | 10.1053/j.gastro.2017.07.045 |
| **632** | M. Riegler, I. Kristo, M. Nikolic, E. Rieder and S. F. Schoppmann | 2017 | Update on the management of Barrett's esophagus in Austria | European Surgery-Acta Chirurgica Austriaca | 10.1007/s10353-017-0504-y |
| **633** | C. B. Rim and S. M. Kim | 2020 | Phlegmonous Gastritis Caused by Penetration of a Toothpick | Korean Journal of Gastroenterology | 10.4166/kjg.2020.75.3.157 |
| **634** | L. S. Riquelme, J. E. G. García and D. M. Macías | 2022 | Characterization of patients with esophageal tumors treated at the Celestino Hernandez Robau Hospital (2016-2017) | Medisur-Revista De Ciencias Medicas De Cienfuegos |  |
| **635** | L. Rizzolo-Brime, A. Farran-Codina, R. Bou, L. Luján-Barroso, J. R. Quirós, P. Amiano, M. J. Sánchez, M. Rodríguez-Barranco, M. Guevara, C. Moreno-Iribas, A. Gasque, M. D. Chirlaque, S. M. Colorado-Yohar, J. M. H. Castaño, A. Agudo and P. Jakszyn | 2024 | Nitrosyl-Heme and Heme Iron Intake from Processed Meats in Subjects from the EPIC-Spain Cohort | Nutrients | 10.3390/nu16060878 |
| **636** | F. Roesch-Dietlen, A. D. Cano-Contreras, Y. J. Sánchez-Maza, J. M. Espinosa-González, M. A. Vázquez-Prieto, E. J. Valdés-de la, F. Díaz-Roesch, M. A. Carrasco-Arroniz, A. Cruz-Palacios, P. Grube-Pagola, A. Sumoza-Toledo, H. Vivanco-Cid, G. Mellado-Sánchez, A. Meixueiro-Daza, C. S. Silva-Cañetas, M. G. Carrillo-Toledo, R. Lagunes-Torres, M. Amieva-Balmori, P. C. Gómez-Castaño, J. U. Reyes-Huerta and J. M. Remes-Troche | 2018 | Frequency of human papillomavirus infection in patients with gastrointestinal cancer | Revista De Gastroenterologia De Mexico | 10.1016/j.rgmx.2017.09.003 |
| **637** | A. J. Romain, J. Marleau and A. Baillot | 2018 | Impact of obesity and mood disorders on physical comorbidities, psychological well-being, health behaviours and use of health services | Journal of Affective Disorders | 10.1016/j.jad.2017.08.065 |
| **638** | C. Rong, S. H. Shen, L. W. Xiao, Q. Huang, H. T. Lu, H. X. Wang, Z. X. Li and X. M. Wang | 2019 | A Comparative Study on the Health Status and Behavioral Lifestyle of Centenarians and Non-centenarians in Zhejiang Province, China-A Cross-Sectional Study | Frontiers in Public Health | 10.3389/fpubh.2019.00344 |
| **639** | A. Rosenfeld, D. G. Graham, S. Jevons, J. Ariza, D. Hagan, A. Wilson, S. J. Lovat, S. S. Sami, O. F. Ahmad, M. Novelli, M. R. Justo, A. Winstanley, E. M. Heifetz, M. Ben-Zecharia, U. Noiman, R. C. Fitzgerald, P. Sasieni, L. B. Lovat and B. S. Grp | 2020 | Development and validation of a risk prediction model to diagnose Barrett's oesophagus (MARK-BE): a case-control machine learning approach | Lancet Digital Health | 10.1016/s2589-7500(19)30216-x |
| **640** | S. Roshini, S. S. Kanna, K. N. Siri and S. G. Thomas | 2023 | A Study on Definitive Role of Smoking Over Alcohol on Peptic Ulcer Disease | International Journal of Life Science and Pharma Research | 10.22376/ijlpr.2023.13.3.L88-L93 |
| **641** | B. L. Rostron, J. Wang, A. Etemadi, S. Thakur, J. T. Chang, D. Bhandari, J. C. Botelho, V. R. De Jesús, J. Feng, M. H. Gail, M. Inoue-Choi, R. Malekzadeh, A. Pourshams, H. Poustchi, G. Roshandel, M. S. Shiels, Q. Wang, Y. S. Wang, B. Y. Xia, P. Boffetta, P. Brennan, C. C. Abnet, A. M. Calafat, L. Q. Wang, B. C. Blount, N. D. Freedman and C. M. Chang | 2021 | Associations between Biomarkers of Exposure and Lung Cancer Risk among Exclusive Cigarette Smokers in the Golestan Cohort Study | International Journal of Environmental Research and Public Health | 10.3390/ijerph18147349 |
| **642** | M. Rota, G. Alicandro, C. Pelucchi, R. Bonzi, P. Bertuccio, J. F. Hu, Z. F. Zhang, K. C. Johnson, D. Palli, M. Ferraroni, G. P. Yu, C. Galeone, L. López-Carrillo, J. Muscat, N. Lunet, A. Ferro, W. M. Ye, A. Plymoth, R. Malekzadeh, D. Zaridze, D. Maximovitch, M. Kogevinas, N. F. de Larrea, J. Vioque, E. M. Navarrete-Muñoz, S. Tsugane, G. S. Hamada, A. Hidaka, M. Pakseresht, A. Wolk, N. Hakansson, R. U. Hernández-Ramírez, M. López-Cervantes, M. Ward, F. Pourfarzi, L. Mu, R. C. Kurtz, A. Lagiou, P. Lagiou, P. Boffetta, S. Boccia, E. Negri and C. La Vecchia | 2020 | Education and gastric cancer risk-An individual participant data meta-analysis in the StoP project consortium | International Journal of Cancer | 10.1002/ijc.32298 |
| **643** | S. Roy, T. Reang and A. Kumar | 2018 | TOBACCO USE AND BODY MASS INDEX AMONG ADULT POPULATION IN SELECTED URBAN AREA OF AGARTALA CITY- A CROSS-SECTIONAL STUDY | Journal of Evolution of Medical and Dental Sciences-Jemds | 10.14260/jemds/2018/114 |
| **644** | W. Rui, C. C. Li, Q. Da, Y. Yue, L. Jing, R. R. Guo, Y. B. Cui, T. Y. Lu and B. Li | 2024 | Analysis of the influencing factors in the long-term survival of esophageal cancer | Frontiers in Oncology | 10.3389/fonc.2023.1274014 |
| **645** | T. M. Runge, J. A. Abrams and N. J. Shaheen | 2015 | Epidemiology of Barrett's Esophagus and Esophageal Adenocarcinoma | Gastroenterology Clinics of North America | 10.1016/j.gtc.2015.02.001 |
| **646** | S. Sadafi, A. Azizi, Y. Pasdar, E. Shakiba and M. Darbandi | 2024 | Risk factors for gastroesophageal reflux disease: a population-based study | Bmc Gastroenterology | 10.1186/s12876-024-03143-9 |
| **647** | S. Saez-Atienzar, S. Bandres-Ciga, R. G. Langston, J. J. Kim, S. W. Choi, R. H. Reynolds, Y. Abramzon, R. Dewan, S. Ahmed, J. E. Landers, R. Chia, M. Ryten, M. R. Cookson, M. A. Nalls, A. Chiò, B. J. Traynor, A. L. S. G. C. Int and Italsgen | 2021 | Genetic analysis of amyotrophic lateral sclerosis identifies contributing pathways and cell types | Science Advances | 10.1126/sciadv.abd9036 |
| **648** | A. Safari, M. Reazai, A. Tangestaninejad, A. R. Mafi and S. A. J. Mousavi | 2016 | Opium consumption: A potential risk factor for lung cancer and pulmonary tuberculosis | Indian Journal of Cancer | 10.4103/0019-509x.204755 |
| **649** | R. Sagami, K. Hayasaka, T. Ujihara, T. Iwaki, Y. Katsuyama, H. Harada, Y. Ome, G. Honda, S. I. Horiguchi, K. Murakami and Y. Amano | 2023 | Role of EUS combined with a newly modified scoring system to detect pancreatic high-grade precancerous lesions | Endoscopic Ultrasound | 10.4103/eus-d-21-00187 |
| **650** | S. K. Sah, N. Neupane, A. Pradhan, S. Shah and A. Sharma | 2020 | Prevalence of glue-sniffing among street children | Nursing Open | 10.1002/nop2.380 |
| **651** | E. Saito, S. Tanaka, S. K. Abe, M. Hirayabashi, J. Ishihara, K. Katanoda, Y. S. Lin, C. Nagata, N. Sawada, R. Takachi, A. Goto, J. Tanaka, K. Ueda, M. Hori, T. Matsuda and M. Inoue | 2023 | Economic burden of cancer attributable to modifiable risk factors in Japan | Global Health & Medicine | 10.35772/ghm.2023.01001 |
| **652** | Y. Sakata, K. Tominaga, M. Kato, H. Takeda, Y. Shimoyama, T. Takeuchi, R. Iwakiri, K. Furuta, K. Sakurai, T. Odaka, H. Kusunoki, A. Nagahara, K. Iwakiri, T. Furuta, K. Murakami, H. Miwa, Y. Kinoshita, K. Haruma, S. Takahashi, S. Watanabe, K. Higuchi, K. Fujimoto, M. Kusano, T. Arakawa and G. P. S. Grp | 2014 | Clinical characteristics of elderly patients with proton pump inhibitor-refractory non-erosive reflux disease from the G-PRIDE study who responded to rikkunshito | Bmc Gastroenterology | 10.1186/1471-230x-14-116 |
| **653** | T. Sakurai, A. Hoshino, K. Miyoshi, E. Yamada, M. Enomoto, J. Mazaki, H. Kuwabara, K. Iwasaki, Y. Ota, S. Tachibana, Y. Hayashi, T. Ishizaki and Y. Nagakawa | 2024 | Long-term outcomes of robot-assisted versus minimally invasive esophagectomy in patients with thoracic esophageal cancer: a propensity score-matched study | World Journal of Surgical Oncology | 10.1186/s12957-024-03358-w |
| **654** | S. Saleh, B. D. Liu, S. Trujillo, C. Thomas and R. Fass | 2023 | The effect of combined oral contraceptives and Nexplanon on gastroesophageal reflux disease in premenopausal women: A nationwide database analysis | Neurogastroenterology and Motility | 10.1111/nmo.14542 |
| **655** | S. S. Sami, K. Ragunath and P. G. Iyer | 2015 | PERSPECTIVES IN CLINICAL GASTROENTEROLOGY AND HEPATOLOGY | Clinical Gastroenterology and Hepatology | 10.1016/j.cgh.2014.03.036 |
| **656** | S. A. Samjo, Z. Abbas, M. Asim and K. Tahir | 2020 | The Pattern of Alcohol Consumption and the Severity of Alcohol-related Liver Disease in Patients Visiting the Liver Clinic | Cureus Journal of Medical Science | 10.7759/cureus.7251 |
| **657** | J. N. Sampson, W. A. Wheeler, M. Yeager, O. Panagiotou, Z. Wang, S. I. Berndt, Q. Lan, C. C. Abnet, L. T. Amundadottir, J. D. Figueroa, M. T. Landi, L. Mirabello, S. A. Savage, P. R. Taylor, I. De Vivo, K. A. McGlynn, M. P. Purdue, P. Rajaraman, H. O. Adami, A. Ahlbom, D. Albanes, M. F. Amary, S. J. An, U. Andersson, G. Andriole, I. L. Andrulis, E. Angelucci, S. M. Ansell, C. Arici, B. K. Armstrong, A. A. Arslan, M. A. Austin, D. Baris, D. A. Barkauskas, B. A. Bassig, N. Becker, Y. Benavente, S. Benhamou, C. Berg, D. Van Den Berg, L. Bernstein, K. A. Bertrand, B. M. Birmann, A. Black, H. Boeing, P. Boffetta, M. C. Boutron-Ruault, P. M. Bracci, L. Brinton, A. R. Brooks-Wilson, H. B. Bueno-De-Mesquita, L. Burdett, J. Buring, M. A. Butler, Q. Y. Cai, G. Cancel-Tassin, F. Canzian, A. Carrato, T. Carreon, A. Carta, J. K. C. Chan, E. T. Chang, G. C. Chang, I. S. Chang, J. Chang, J. Chang-Claude, C. J. Chen, C. Y. Chen, C. Chen, C. H. Chen, C. Chen, H. Y. Chen, K. X. Chen, K. Y. Chen, K. C. Chen, Y. Chen, Y. H. Chen, Y. S. Chen, Y. M. Chen, L. H. Chien, M. D. Chirlaque, J. E. Choi, Y. Y. Choi, W. H. Chow, C. C. Chung, J. Clavel, F. Clavel-Chapelon, P. Cocco, J. S. Colt, E. Comperat, L. Conde, J. M. Connors, D. Conti, V. K. Cortessis, M. Cotterchio, W. Cozen, S. Crouch, M. Crous-Bou, O. Cussenot, F. G. Davis, T. Ding, W. R. Diver, M. Dorronsoro, L. Dossus, E. J. Duell, M. G. Ennas, R. L. Erickson, M. Feychting, A. M. Flanagan, L. Foretova, J. F. Fraumeni, N. D. Freedman, L. E. B. Freeman, C. Fuchs, M. Gago-Dominguez, S. Gallinger, Y. T. Gao, S. M. Gapstur, M. Garcia-Closas, R. García-Closas, R. D. Gascoyne, J. Gastier-Foster, M. M. Gaudet, J. M. Gaziano, C. Giffen, G. G. Giles, E. Giovannucci, B. Glimelius, M. Goggins, N. Gokgoz, A. M. Goldstein, R. Gorlick, M. Gross, R. Grubb, J. Gu, P. Guan, M. Gunter, H. Guo, T. M. Habermann, C. A. Haiman, D. Halai, G. Hallmans, M. Hassan, C. Hattinger, Q. C. He, X. Z. He, K. Helzlsouer, B. Henderson, R. Henriksson, H. Hjalgrim, J. Hoffman-Bolton, C. Hohensee, T. R. Holford, E. A. Holly, Y. C. Hong, R. N. Hoover, P. L. Horn-Ross, G. M. M. Hosain, H. D. Hosgood, C. F. Hsiao, N. Hu, W. Hu, Z. B. Hu, M. S. Huang, J. M. Huerta, J. Y. Hung, A. Hutchinson, P. D. Inskip, R. D. Jackson, E. J. Jacobs, M. Jenab, H. S. Jeon, B. T. Ji, G. F. Jin, L. Jin, C. Johansen, A. Johnson, Y. J. Jung, R. Kaaks, A. Kamineni, E. Kane, C. H. Kang, M. R. Karagas, R. S. Kelly, K. T. Khaw, C. Kim, H. N. Kim, J. H. Kim, J. S. Kim, Y. H. Kim, Y. T. Kim, Y. C. Kim, C. M. Kitahara, A. P. Klein, R. J. Klein, M. Kogevinas, T. Kohno, L. N. Kolonel, C. Kooperberg, A. Kricker, V. Krogh, H. Kunitoh, R. C. Kurtz, S. S. Kweon, A. LaCroix, C. Lawrence, F. Lecanda, V. H. F. Lee, D. H. Li, H. X. Li, J. H. Li, Y. J. Li, Y. Q. Li, L. M. Liao, M. Liebow, T. Lightfoot, W. Y. Lim, C. C. Lin, D. X. Lin, S. Lindstrom, M. S. Linet, B. K. Link, C. W. Liu, J. J. Liu, L. Liu, B. Ljungberg, J. Lloreta, S. Di Lollo, D. Lu, E. Lund, N. Malats, S. Mannisto, L. Le Marchand, N. Marina, G. Masala, G. Mastrangelo, K. Matsuo, M. Maynadie, J. McKay, R. McKean-Cowdin, M. Melbye, B. S. Melin, D. S. Michaud, T. Mitsudomi, A. Monnereau, R. Montalvan, L. E. Moore, L. M. Mortensen, A. Nieters, K. E. North, A. J. Novak, A. L. Oberg, K. Offit, I. J. Oh, S. H. Olson, D. Palli, W. Pao, I. K. Park, J. Y. Park, K. H. Park, A. Patiño-Garcia, S. Pavanello, P. H. M. Peeters, R. P. Perng, U. Peters, G. M. Petersen, P. Picci, M. C. Pike, S. Porru, J. Prescott, L. Prokunina-Olsson, B. Qian, Y. L. Qiao, M. Rais, E. Riboli, J. Riby, H. A. Risch, C. Rizzato, R. Rodabough, E. Roman, M. Roupret, A. M. Ruder, S. de Sanjose, G. Scelo, A. Schned, F. Schumacher, K. Schwartz, M. Schwenn, K. Scotlandi, A. Seow, C. Serra, M. Serra, H. D. Sesso, V. W. Setiawan, G. Severi, R. K. Severson, T. D. Shanafelt, H. B. Shen, W. Shen, M. H. Shin, K. Shiraishi, X. O. Shu, A. Siddiq, L. Sierrasesúmaga, A. D. L. Sihoe, C. F. Skibola, A. Smith, M. T. Smith, M. C. Southey, J. J. Spinelli, A. Staines, M. Stampfer, M. C. Stern, V. L. Stevens, R. S. Stolzenberg-Solomon, J. Su, W. C. Su, M. Sund, J. S. Sung, S. W. Sung, W. Tan, W. Tang, A. Tardón, D. Thomas, C. A. Thompson, L. F. Tinker, R. Tirabosco, A. Tjonneland, R. C. Travis, D. Trichopoulos, F. Y. Tsai, Y. H. Tsai, M. Tucker, J. Turner, C. M. Vajdic, R. C. H. Vermeulen, D. J. Villano, P. Vineis, J. Virtamo, K. Visvanathan, J. Wactawski-Wende, C. Y. Wang, C. L. Wang, J. C. Wang, J. W. Wang, F. S. Wei, E. Weiderpass, G. J. Weiner, S. Weinstein, N. Wentzensen, E. White, T. E. Witzig, B. M. Wolpin, M. P. Wong, C. Wu, G. P. Wu, J. J. Wu, T. C. Wu, W. Wu, X. F. Wu, Y. L. Wu, J. S. Wunder, Y. B. Xiang, J. Xu, P. Xu, P. C. Yang, T. Y. Yang, Y. Q. Ye, Z. H. Yin, J. Yokota, H. I. Yoon, C. J. Yu, H. Yu, K. Yu, J. M. Yuan, A. Zelenetz, A. Zeleniuch-Jacquotte, X. C. Zhang, Y. W. Zhang, X. Y. Zhao, Z. H. Zhao, H. Zheng, T. Z. Zheng, W. Zheng, B. S. Zhou, M. Zhu, M. Zucca, S. M. Boca, J. R. Cerhan, G. M. Ferri, P. Hartge, C. A. Hsiung, C. Magnani, L. Miligi, L. M. Morton, K. E. Smedby, L. R. Teras, J. Vijai, S. S. Wang, P. Brennan, N. E. Caporaso, D. J. Hunter, P. Kraft, N. Rothman, D. T. Silverman, S. L. Slager, S. J. Chanock and N. Chatterjee | 2015 | Analysis of Heritability and Shared Heritability Based on Genome-Wide Association Studies for 13 Cancer Types | Jnci-Journal of the National Cancer Institute | 10.1093/jnci/djv279 |
| **658** | Y. H. Sang, L. Shi, Y. H. Wu, W. T. Yang, H. Y. Gu, J. Yin, L. R. X. Yuan, C. Liu, X. Wang, Y. J. Shi, W. F. Tang and Y. B. Chen | 2016 | <i>Epiregulin</i> rs1460008 A&gt;G polymorphism is associated with decreased risk of esophageal squamous cell carcinoma in a Chinese population | International Journal of Clinical and Experimental Medicine |  |
| **659** | C. Santucci, H. N. Medina, G. Carioli, E. Negri, C. La Vecchia and P. S. Pinheiro | 2022 | Cancer mortality in Italian populations: differences between Italy and the USA | European Journal of Cancer Prevention | 10.1097/cej.0000000000000712 |
| **660** | S. Sarvepalli, S. K. Garg, S. S. Sarvepalli, C. Anugwom, V. Wadhwa, P. N. Thota and M. R. Sanaka | 2019 | Hospital Utilization in Patients With Gastric Cancer and Factors Affecting In-Hospital Mortality, Length of Stay, and Costs | Journal of Clinical Gastroenterology | 10.1097/mcg.0000000000001016 |
| **661** | H. K. Sarvestani, R. D. Ghazvini, S. J. Hashemi, M. G. Shoar, S. Ansari, Z. Rafat, A. Ahmadi, P. Borghei, M. Elahi, A. R. Foroushani, M. I. Getso, S. Aboutalebian, F. Safari and P. Ardi | 2022 | Molecular Characterization of Fungal Colonization on the Provox™ Tracheoesophageal Voice Prosthesis in Post Laryngectomy Patients | Iranian Journal of Public Health |  |
| **662** | F. Sarwar, M. Saleem and F. Z. Zaidi | 2021 | Prevalence of Gastroesophageal Reflux Disease in rural women presenting to a primary care hospital | Rawal Medical Journal |  |
| **663** | H. Sato, Y. Nishikawa, H. Abe, H. Shiwaku, J. Shiota, C. Sato, H. Sakae, M. Ominami, Y. Hata, H. Fukuda, R. Ogawa, J. Nakamura, T. Tatsuta, Y. Ikebuchi, H. Yokomichi, S. Terai and H. Inoue | 2022 | Esophageal carcinoma in achalasia patients managed with endoscopic submucosal dissection and peroral endoscopic myotomy: Japan Achalasia Multicenter Study | Digestive Endoscopy | 10.1111/den.14197 |
| **664** | M. Sattar, S. Kanwal and A. Abbas | 2019 | A COMPREHENSIVE STUDY ON GASTRO-ESOPHAGEAL REFLUX DISEASE AND ITS RISK FACTORS AMONG LOCAL POPULATION OF PAKISTAN | Indo American Journal of Pharmaceutical Sciences | 10.5281/zenodo.2529622 |
| **665** | S. Sawai, I. Arshad, K. Hussain, A. Ahmed, P. Kumar and M. Sadiq | 2021 | Esophageal Carcinoma and Predisposing Factors Among Patients Presented at Isra University Hospital Hyderabad | Pakistan Journal of Medical & Health Sciences | 10.53350/pjmhs2115103058 |
| **666** | M. Schmidt, D. P. Ankerst, Y. Y. Chen, M. Wiethaler, J. Slotta-Huspenina, K. F. Becker, J. Horstmann, F. Kohlmayer, A. Lehmann, B. Linkohr, K. Strauch, R. M. Schmid, A. S. Quante and M. Quante | 2020 | Epidemiologic Risk Factors in a Comparison of a Barrett Esophagus Registry (BarrettNET) and a Case-Control Population in Germany | Cancer Prevention Research | 10.1158/1940-6207.Capr-19-0474 |
| **667** | C. V. Schneider, K. M. Schneider, A. Teumer, K. L. Rudolph, D. Hartmann, D. J. Rader and P. Strnad | 2022 | Association of Telomere Length With Risk of Disease and Mortality | Jama Internal Medicine | 10.1001/jamainternmed.2021.7804 |
| **668** | J. Schwartz, C. Bashian, L. Kushnir, C. Nituica and G. J. Slotman | 2017 | Variation in Clinical Characteristics of Women versus Men Preoperative for Laparoscopic Roux-en-Y Gastric Bypass: Analysis of 83,059 Patients | American Surgeon |  |
| **669** | T. Schweiger, D. Kollmann, C. Nikolowsky, D. Traxler, E. Guenova, G. Lang, P. Birner, W. Klepetko, H. J. Ankersmit and K. Hoetzenecker | 2014 | Carbonic anhydrase IX is associated with early pulmonary spreading of primary colorectal carcinoma and tobacco smoking | European Journal of Cardio-Thoracic Surgery | 10.1093/ejcts/ezt542 |
| **670** | I. Sen, L. Yohanathan, J. M. Kärkkäinen and D. M. Nagorney | 2021 | Current Indications and Long-Term Outcomes of Surgical Portosystemic Shunts in Adults | Journal of Gastrointestinal Surgery | 10.1007/s11605-020-04643-1 |
| **671** | G. Senna, M. Latorre, M. Bugiani, M. Caminati, E. Heffler, D. Morrone, G. Paoletti, P. Parronchi, F. Puggioni, F. Blasi, G. W. Canonica, P. Paggiaro and S. Network | 2021 | Sex Differences in Severe Asthma: Results From Severe Asthma Network in Italy-SANI | Allergy Asthma & Immunology Research | 10.4168/aair.2021.13.2.219 |
| **672** | A. A. Senusi, J. Mather, D. Ola, L. A. Bergmeier, B. Gokani and F. Fortune | 2022 | The impact of multifactorial factors on the Quality of Life of Behcet's patients over 10 years | Frontiers in Medicine | 10.3389/fmed.2022.996571 |
| **673** | G. Senyondo, A. Khan, F. Malik and A. Oranu | 2022 | Esophagitis Dissecans Superficialis: A Frequently Missed and Rarely Reported Diagnosis | Cureus Journal of Medical Science | 10.7759/cureus.21647 |
| **674** | J. H. Seo, Y. D. Kim, C. S. Park, K. D. Han and Y. H. Joo | 2020 | Hypertension is associated with oral, laryngeal, and esophageal cancer: a nationwide population-based study | Scientific Reports | 10.1038/s41598-020-67329-3 |
| **675** | W. J. Seow, K. Matsuo, C. A. Hsiung, K. Shiraishi, M. S. Song, H. N. Kim, M. P. Wong, Y. C. Hong, H. D. Hosgood, Z. M. Wang, I. S. Chang, J. C. Wang, N. Chatterjee, M. Tucker, H. Wei, T. Mitsudomi, W. Zheng, J. H. Kim, B. S. Zhou, N. E. Caporaso, D. Albanes, M. H. Shin, L. P. Chung, S. J. An, P. Wang, H. Zheng, Y. Yatabe, X. C. Zhang, Y. T. Kim, X. O. Shu, Y. C. Kim, B. A. Bassig, J. Chang, J. C. M. Ho, B. T. Ji, M. Kubo, Y. Daigo, H. Ito, Y. Momozawa, K. Ashikawa, Y. Kamatani, T. Honda, H. Sakamoto, H. Kunitoh, K. Tsuta, S. I. Watanabe, H. Nokihara, Y. Miyagi, H. Nakayama, S. Matsumoto, M. Tsuboi, K. Goto, Z. H. Yin, J. X. Shi, A. Takahashi, A. Goto, Y. Minamiya, K. Shimizu, K. Tanaka, T. C. Wu, F. S. Wei, J. Y. Y. Wong, F. Matsuda, J. Su, Y. H. Kim, I. J. Oh, F. J. Song, V. H. F. Lee, W. C. Su, Y. M. Chen, G. C. Chang, K. Y. Chen, M. S. Huang, P. C. Yang, H. C. Lin, Y. B. Xiang, A. Seow, J. Y. Park, S. S. Kweon, C. J. Chen, H. X. Li, Y. T. Gao, C. Wu, B. Y. Qian, D. R. Lu, J. J. Liu, H. S. Jeon, C. F. Hsiao, J. S. Sung, Y. H. Tsai, Y. J. Jung, H. Guo, Z. B. Hu, W. C. Wang, C. C. Chung, C. Lawrence, L. Burdett, M. Yeager, K. B. Jacobs, A. Hutchinson, S. I. Berndt, X. Z. He, W. Wu, J. W. Wang, Y. Q. Li, J. E. Choi, K. H. Park, S. W. Sung, L. Liu, C. H. Kang, L. M. Hu, C. H. Chen, T. Y. Yang, J. Xu, P. Guan, W. Tan, C. L. Wang, A. D. L. Sihoe, Y. Chen, Y. Y. Choi, J. Y. Hung, J. S. Kim, H. I. Yoon, Q. Y. Cai, C. C. Lin, I. K. Park, P. Xu, J. Dong, C. Kim, Q. C. He, R. P. Perng, C. Y. Chen, R. Vermeulen, J. J. Wu, W. Y. Lim, K. C. Chen, J. K. C. Chan, M. J. Chu, Y. J. Li, J. H. Li, H. Y. Chen, C. J. Yu, L. Jin, Y. L. Lo, Y. H. Chen, J. F. Fraumeni, J. Liu, T. Yamaji, Y. Yang, B. Hicks, K. Wyatt, S. A. Li, J. C. Dai, H. X. Ma, G. F. Jin, B. Song, Z. H. Wang, S. S. Cheng, X. L. Li, Y. W. Ren, P. Cui, M. Iwasaki, T. Shimazu, S. Tsugane, J. J. Zhu, G. N. Jiang, K. Fei, G. P. Wu, L. H. Chien, H. L. Chen, Y. C. Su, F. Y. Tsai, Y. S. Chen, J. M. Yu, V. L. Stevens, I. A. Laird-Offringa, C. N. Marconett, D. X. Lin, K. X. Chen, Y. L. Wu, M. T. Landi, H. B. Shen, N. Rothman, T. Kohno, S. J. Chanock and Q. Lan | 2017 | Association between GWAS-identified lung adenocarcinoma susceptibility loci and <i>EGFR</i> mutations in never-smoking Asian women, and comparison with findings from Western populations | Human Molecular Genetics | 10.1093/hmg/ddw414 |
| **676** | M. S. Seyyedi, V. Zangouri, Z. Dehghani, A. Dehghanian and M. G. Jahromi | 2024 | A rare occurrence of breast, thyroid, and stomach tumors in a single patient: A case report | International Journal of Surgery Case Reports | 10.1016/j.ijscr.2024.109670 |
| **677** | R. Shakeri, F. Kamangar, M. Mohamadnejad, R. Tabrizi, F. Zamani, A. Mohamadkhani, S. Nikfam, A. Nikmanesh, M. Sotoudeh, R. Sotoudehmanesh, B. Shahbazkhani, M. R. Ostovaneh, F. Islami, H. Poustchi, P. Boffetta, R. Malekzadeh and A. Pourshams | 2016 | Opium use, cigarette smoking, and alcohol consumption in relation to pancreatic cancer | Medicine | 10.1097/md.0000000000003922 |
| **678** | R. Sharma | 2024 | Burden of Stomach Cancer Incidence, Mortality, Disability-Adjusted Life Years, and Risk Factors in 204 Countries, 1990-2019: An Examination of Global Burden of Disease 2019 | Journal of Gastrointestinal Cancer | 10.1007/s12029-023-01005-3 |
| **679** | R. Sharma, H. Abbastabar, D. M. Abdulah, H. Abidi, H. Abolhassani, Z. Abrehdari-Tafreshi, A. Absalan, H. A. Ali, E. Abu-Gharbieh, J. M. Acuna, N. Adib, Q. E. S. Adnani, A. Aghaei, A. Ahmad, S. Ahmad, A. Ahmadi, S. Ahmadi, L. A. Ahmed, M. Ajami, H. Al Hamad, S. M. Al Hasan, F. M. Alanezi, A. A. S. Al-Gheethi, M. K. Al-Hanawi, A. Ali, B. A. Ali, Y. Alimohamadi, S. M. Aljunid, S. A. A. Al-Maweri, S. A. Alqahatni, M. AlQudah, R. M. Al-Raddadi, A. B. Al-Tammemi, A. Ansari-Moghaddam, S. L. Anwar, R. Anwer, M. Aqeel, J. Arabloo, M. Arab-Zozani, H. Ariffin, A. Artaman, J. Arulappan, T. Ashraf, E. Askari, M. Athar, M. M. W. Atout, S. Azadnajafabad, M. Badar, A. D. Badiye, N. Baghcheghi, S. Bagherieh, R. H. Bai, K. Bajbouj, S. Baliga, M. Bardhan, A. Bashiri, P. Baskaran, S. Basu, U. I. Belgaumi, A. N. C. Bermudez, B. Bhandari, N. Bhardwaj, A. N. Bhat, S. Bitaraf, A. Boloor, M. B. Hashemi, Z. A. Butt, J. Chadwick, J. S. K. Chan, V. K. Chattu, P. Chaturvedi, W. C. S. Cho, A. M. Darwesh, N. R. Dash, A. Dehghan, A. Dhali, M. Dianatinasab, M. Dibas, A. Dixit, S. G. Dixit, F. Dorostkar, H. L. Dsouza, I. Elbarazi, N. M. Elemam, W. El-Huneidi, E. Elkord, O. A. A. Elmeligy, M. H. Emamian, R. Erkhembayar, R. Ezzeddini, Z. Fadoo, R. Faiz, I. R. Fakhradiyev, A. Fallahzadeh, M. E. M. Faris, H. Farrokhpour, A. Fatehizadeh, H. Fattahi, G. Fekadu, T. Fukumoto, A. M. Gaidhane, N. Galehdar, P. Garg, F. Ghadirian, M. Ghafourifard, M. Ghasemi, M. G. Nour, F. Ghassemi, M. Gholamalizadeh, A. Gholamian, E. Ghotbi, M. Golechha, P. Goleij, S. Goyal, M. I. M. Gubari, D. S. Gunasekera, D. A. Gunawardane, S. Gupta, P. Habibzadeh, H. S. H. Boroojeni, E. S. Halboub, R. R. Hamadeh, R. Hamoudi, M. Harorani, M. Hasanian, T. S. Hassan, S. Hay, M. Heidari, M. Heidari-Foroozan, K. Hessami, K. Hezam, Y. Hiraike, R. Holla, M. Hoseini, M. M. Hossain, S. Hossain, V. C. R. Hsieh, J. J. Huang, N. R. Hussein, B. F. Hwang, F. Iravanpour, N. E. Ismail, M. Iwagami, J. L. Merin, F. Jadidi-Niaragh, M. Jafarinia, M. A. Jahani, H. Jahrami, A. Jaiswal, M. Jakovljevic, M. Jalili, E. Jamshidi, U. Jayarajah, S. Jayaram, S. S. Jha, M. Jokar, N. Joseph, A. Kabir, M. A. Kabir, D. H. Kadir, P. V. Kakodkar, L. R. Kalankesh, L. R. Kalankesh, R. Kalhor, F. Kaliyadan, V. K. Kamal, Z. Kamal, A. Kamath, S. S. Kar, H. Karimi, N. Kaur, L. Keikavoosi-Arani, M. Keykhaei, Y. S. Khader, H. Khajuria, E. A. Khan, M. N. Khan, M. Khan, M. A. B. Khan, Y. H. Khan, S. Khanmohammadi, M. M. Khatatbeh, S. Khateri, M. Khayamzadeh, H. R. K. Kashani, M. S. Kim, F. Kompani, H. R. Koohestani, S. L. K. Laxminarayana, K. Krishan, N. Kumar, N. Kumar, T. Kutluk, A. Kuttikkattu, D. T. C. Lai, D. K. Lal, F. H. Lami, S. Lasrado, S. W. Lee, S. W. Lee, Y. Y. Lee, Y. H. Lee, E. Leong, M. C. Li, J. Liu, F. Madadizadeh, A. R. Mafi, S. Mahjoub, R. Malekzadeh, A. A. Malik, I. Malik, T. H. Mallhi, M. A. Mansournia, S. Martini, E. Mathews, M. R. Mathur, J. K. Meena, R. G. Menezes, R. Mirfakhraie, S. K. Mirinezhad, M. Mirza-Aghazadeh-Attari, P. Mithra, A. Mohamadkhani, S. Mohammadi, M. Mohammadzadeh, S. Mohan, A. H. Mokdad, A. Al Montasir, F. Montazeri, M. Moradi, M. M. Sarabi, F. Moradpour, M. Moradzadeh, P. Moraga, A. Mosapour, M. Motaghinejad, S. Mubarik, J. S. Muhammad, C. J. L. Murray, A. J. Nagarajan, M. Naghavi, S. Nargus, Z. S. Natto, B. P. Nayak, S. A. Nejadghaderi, P. T. Nguyen, R. K. Niazi, N. Noroozi, H. Okati-Aliabad, A. P. Okekunle, S. Ong, A. M. Oommen, J. R. Padubidri, A. Pandey, E. K. Park, S. Park, S. Pati, S. Patil, R. Paudel, U. Paudel, M. Pirestani, I. Podder, G. Pourali, M. Pourjafar, A. Pourshams, Z. Q. Syed, R. A. Radhakrishnan, V. Radhakrishnan, M. Rahman, S. Rahmani, V. Rahmanian, P. S. Ramesh, J. Rana, I. R. Rao, S. J. Rao, S. Rashedi, M. M. Rashidi, N. Rezaei, N. Rezaei, N. Rezaei, S. Rezaei, M. Rezaeian, G. Roshandel, S. N. Chandan, M. M. Saber-Ayad, S. Sabour, L. Sabzmakan, B. Saddik, U. Saeed, S. Z. Safi, F. S. Sharif-Askari, A. Sahebkar, H. Sahoo, S. A. Sajedi, M. R. Sajid, M. A. Salehi, A. S. Farrokhi, M. A. Sarasmita, S. Sargazi, G. S. Sarode, S. C. Sarode, B. Sathian, M. Satpathy, P. Semwal, S. Senthilkumaran, S. G. Sepanlou, M. Shafeghat, S. Shahabi, A. Shahbandi, F. Shahraki-Sanavi, M. A. Shaikh, M. Shannawaz, R. A. Sheikhi, P. Shobeiri, S. A. Shorofi, S. Shrestha, S. Siabani, G. Singh, P. Singh, S. Singh, D. N. Sinha, S. S. Siwal, S. Sreeram, M. Suleman, R. S. Abdulkader, I. Sultan, A. Sultana, M. Tabish, T. Tabuchi, M. Taheri, I. M. Talaat, A. Tehrani-Banihashemi, M. H. Temsah, P. Thangaraju, N. Thomas, N. K. Thomas, A. Tiyuri, R. Tobe-Gai, R. Toghroli, M. R. T. Palone, S. Ullah, B. Unnikrishnan, E. Upadhyay, S. V. Tahbaz, R. Valizadeh, S. B. Varthya, Y. Waheed, S. Wang, D. P. Wickramasinghe, N. D. Wickramasinghe, H. Xiao, N. Yonemoto, M. Z. Younis, C. H. Yu, M. Zahir, N. Zaki, M. Zamanian, Z. J. Zhang, H. Q. Zhao, O. A. Zitoun, M. Zoladl and G. B. D. A. A. Canc | 2024 | Temporal patterns of cancer burden in Asia, 1990-2019: a systematic examination for the Global Burden of Disease 2019 study | Lancet Regional Health - Southeast Asia | 10.1016/j.lansea.2023.100333 |
| **680** | R. Sharma and B. Rakshit | 2023 | Global burden of cancers attributable to tobacco smoking, 1990-2019: an ecological study | Epma Journal | 10.1007/s13167-022-00308-y |
| **681** | F. M. Shebl, A. W. Hsing, Y. Park, A. R. Hollenbeck, L. W. Chu, T. E. Meyer and J. Koshiol | 2014 | Non-Steroidal Anti-Inflammatory Drugs Use Is Associated with Reduced Risk of Inflammation-Associated Cancers: NIH-AARP Study | Plos One | 10.1371/journal.pone.0114633 |
| **682** | M. Sheikh, H. Poustchi, A. Pourshams, A. Etemadi, F. Islami, M. Khoshnia, A. Gharavi, M. Hashemian, G. Roshandel, H. Khademi, M. Zahedi, B. Abedi-Ardekani, P. Boffetta, F. Kamangar, S. M. Dawsey, P. D. Pharaoh, C. C. Abnet, N. E. Day, P. Brennan and R. Malekzadeh | 2019 | Individual and Combined Effects of Environmental Risk Factors for Esophageal Cancer Based on Results From the Golestan Cohort Study | Gastroenterology | 10.1053/j.gastro.2018.12.024 |
| **683** | M. Sheikh, R. Shakeri, H. Poustchi, A. Pourshams, A. Etemadi, F. Islami, M. Khoshnia, A. Gharavi, G. Roshandel, H. Khademi, S. G. Sepanlou, M. Hashemian, A. Fazel, M. Zahedi, B. Abedi-Ardekani, P. Boffetta, S. M. Dawsey, P. D. Pharoah, M. Sotoudeh, N. D. Freedman, C. C. Abnet, N. E. Day, P. Brennan, F. Kamangar and R. Malekzadeh | 2020 | Opium use and subsequent incidence of cancer: results from the Golestan Cohort Study | Lancet Global Health |  |
| **684** | C. Shen, C. M. Schooling, W. M. Chan, S. Y. Lee, G. M. Leung and T. H. Lam | 2014 | Self-reported diabetes and mortality in a prospective Chinese elderly cohort Study in Hong Kong | Preventive Medicine | 10.1016/j.ypmed.2014.03.021 |
| **685** | S. Shen, J. L. Araujo, N. K. Altorki, J. R. Sonett, A. Rodriguez, K. Sungur-Stasik, C. F. Spinelli, A. I. Neugut and J. A. Abrams | 2017 | Variation by stage in the effects of prediagnosis weight loss on mortality in a prospective cohort of esophageal cancer patients | Diseases of the Esophagus | 10.1093/dote/dox073 |
| **686** | R. J. Shephard | 2017 | Cancers of the Esophagus and Stomach: Potential Mechanisms Behind the Beneficial Influence of Physical Activity | Clinical Journal of Sport Medicine | 10.1097/jsm.0000000000000353 |
| **687** | J. X. Shi, K. Shiraishi, J. Y. Choi, K. Matsuo, T. Y. Chen, J. C. Dai, R. J. Hung, K. X. Chen, X. O. Shu, Y. T. Kim, M. T. Landi, D. X. Lin, W. Zheng, Z. H. Yin, B. S. Zhou, B. Song, J. C. Wang, W. J. Seow, L. Song, I. S. Chang, W. Hu, L. H. Chien, Q. Y. Cai, Y. C. Hong, H. N. Kim, Y. L. Wu, M. P. Wong, B. D. Richardson, K. M. Funderburk, S. L. Li, T. W. Zhang, C. Breeze, Z. M. Wang, B. Blechter, B. A. Bassig, J. H. Kim, D. Albanes, J. Y. Y. Wong, M. H. Shin, L. P. Chung, Y. Yang, S. J. An, H. Zheng, Y. Yatabe, X. C. Zhang, Y. C. Kim, N. E. Caporaso, J. Chang, J. C. M. Ho, M. Kubo, Y. Daigo, M. Song, Y. Momozawa, Y. Kamatani, M. Kobayashi, K. Okubo, T. Honda, D. H. Hosgood, H. Kunitoh, H. Patel, S. Watanabe, Y. Miyagi, H. Nakayama, S. Matsumoto, H. Horinouchi, M. Tsuboi, R. Hamamoto, K. Goto, Y. Ohe, A. Takahashi, A. Goto, Y. Minamiya, M. Hara, Y. Nishida, K. Takeuchi, K. Wakai, K. Matsuda, Y. Murakami, K. Shimizu, H. Suzuki, M. Saito, Y. Ohtaki, K. Tanaka, T. Wu, F. Wei, H. Dai, M. J. Machiela, J. Su, Y. H. Kim, I. J. Oh, V. H. F. Lee, G. C. Chang, Y. H. Tsai, K. Y. Chen, M. S. Huang, W. C. Su, Y. M. Chen, A. Seow, J. Y. Park, S. S. Kweon, K. C. Chen, Y. T. Gao, B. Y. Qian, C. Wu, D. R. Lu, J. J. Liu, A. G. Schwartz, R. Houlston, M. R. Spitz, I. P. Gorlov, X. F. Wu, P. Yang, S. Lam, A. Tardon, C. Chen, S. E. Bojesen, M. Johansson, A. Risch, H. Bickeböller, B. T. Ji, H. E. Wichmann, D. C. Christiani, G. Rennert, S. Arnold, P. Brennan, J. McKay, J. K. Field, S. S. Shete, L. Le Marchand, G. Liu, A. Andrew, L. A. Kiemeney, S. Zienolddiny-Narui, K. Grankvist, M. Johansson, A. Cox, F. Taylor, J. M. Yuan, P. Lazarus, M. B. Schabath, M. C. Aldrich, H. S. Jeon, S. S. Jiang, J. S. Sung, C. H. Chen, C. F. Hsiao, Y. J. Jung, H. Guo, Z. B. Hu, L. Burdett, M. Yeager, A. Hutchinson, B. Hicks, J. Liu, B. Zhu, S. I. Berndt, W. Wu, J. W. Wang, Y. Q. Li, J. E. Choi, K. H. Park, S. W. Sung, L. Liu, C. H. Kang, W. C. Wang, J. Xu, P. Guan, W. Tan, C. J. Yu, G. Yang, A. D. L. Sihoe, Y. Chen, Y. Y. Choi, J. S. Kim, H. I. Yoon, I. K. Park, P. Xu, Q. C. He, C. L. Wang, H. H. Hung, R. C. H. Vermeulen, I. Cheng, J. J. Wu, W. Y. Lim, F. Y. Tsai, J. K. C. Chan, J. H. Li, H. Y. Chen, H. C. Lin, L. Jin, J. Liu, N. Sawada, T. Yamaji, K. Wyatt, S. A. Li, H. X. Ma, M. Zhu, Z. H. Wang, S. S. Cheng, X. L. Li, Y. W. Ren, A. Chao, M. Iwasaki, J. J. Zhu, G. N. Jiang, K. Fei, G. P. Wu, C. Y. Chen, C. J. Chen, P. C. Yang, J. M. Yu, V. L. Stevens, J. F. Fraumeni, N. Chatterjee, O. Y. Gorlova, C. A. Hsiung, C. I. Amos, H. B. Shen, S. J. Chanock, N. Rothman, T. Kohno and Q. Lan | 2023 | Genome-wide association study of lung adenocarcinoma in East Asia and comparison with a European population | Nature Communications | 10.1038/s41467-023-38196-z |
| **688** | B. S. Shiflett, L. S. Ekanayake, A. L. Rodriguez, I. Ikramuddin and C. Myers | 2020 | Esophageal Adenocarcinoma in the Proximal Esophageal Segment: A Unique Presentation in a Male With Alcohol Abuse | Cureus Journal of Medical Science | 10.7759/cureus.8863 |
| **689** | H. Shigaki, Y. Imamura, S. Mine, A. Okamura, T. Kurogochi, K. Yamashita and M. Watanabe | 2017 | Clinicopathological features of esophageal squamous cell carcinoma in never smoker-never drinkers | Diseases of the Esophagus | 10.1093/dote/dow019 |
| **690** | J. Shin, J. I. Zo and J. Lee | 2016 | Management of long-term lung cancer survivors in Korea | Journal of the Korean Medical Association | 10.5124/jkma.2016.59.4.294 |
| **691** | A. Shiomi, T. Miyake, S. Furukawa, B. Matsuura, O. Yoshida, T. Watanabe, A. Kanamoto, M. Miyazaki, H. Nakaguchi, Y. Tokumoto, M. Hirooka, M. Abe and Y. Hiasa | 2024 | Combined effect of histological findings and diabetes mellitus on liver-related events in patients with metabolic dysfunction-associated steatotic liver disease | Hepatology Research | 10.1111/hepr.14049 |
| **692** | J. S. Shirvani, M. Salehi, A. R. Majd, F. Sadeghi, E. Ferdosi-Shahandashti, S. Khafri and M. Rajabnia | 2023 | Expression Assessment of the <i>Helicobacter pylori babA</i> and <i>sabA</i> Genes in Patients with Peptic Ulcer, Duodenal Ulcer and Gastric Cancer | International Journal of Molecular and Cellular Medicine | 10.22088/ijmcm.Bums.12.2.211 |
| **693** | N. Shivappa, J. R. Hébert and B. Rashidkhani | 2015 | Dietary Inflammatory Index and Risk of Esophageal Squamous Cell Cancer in a Case-Control Study from Iran | Nutrition and Cancer-an International Journal | 10.1080/01635581.2015.1082108 |
| **694** | N. Shivappa, A. Zucchetto, D. Serraino, M. Rossi, C. La Vecchia and J. R. Hébert | 2015 | Dietary inflammatory index and risk of esophageal squamous cell cancer in a case-control study from Italy | Cancer Causes & Control | 10.1007/s10552-015-0636-y |
| **695** | H. Shoji, H. Isomoto, A. Yoshida, H. Ikeda, H. Minami, T. Kanda, S. Urabe, K. Matsushima, F. Takeshima, K. Nakao and H. Inoue | 2017 | MicroRNA-130a is highly expressed in the esophageal mucosa of achalasia patients | Experimental and Therapeutic Medicine | 10.3892/etm.2017.4598 |
| **696** | P. Shokuhi, N. J. O'Sullivan, H. C. Temperley, T. Russell, P. D. McEntee, B. J. Mehigan, P. H. McCormick, D. Gallagher, C. Gillham, J. Kennedy, M. E. Kelly and J. O. Larkin | 2024 | Prognostic value of pre-operative mean corpuscular volume (MCV) in colorectal cancer | Irish Journal of Medical Science | 10.1007/s11845-023-03571-8 |
| **697** | M. W. Short, K. G. Burgers and V. T. Fry | 2017 | Esophageal Cancer | American Family Physician |  |
| **698** | G. Shrestha, R. K. Thakur, R. Singh, R. Mulmi, A. Shrestha and P. M. S. Pradhan | 2021 | Cancer burden in Nepal, 1990-2017: An analysis of the Global Burden of Disease study | Plos One | 10.1371/journal.pone.0255499 |
| **699** | M. Shteiner, S. Kleinman, A. Shuster, V. Raiser, C. Ianculovici, H. Rachima and I. Kaplan | 2021 | Submucosal Fibrotic Bands in Oral Lichen Planus: A Clinico-Pathological Investigation of a Newly Described Phenomenon | Head & Neck Pathology | 10.1007/s12105-020-01203-6 |
| **700** | F. Shweikeh, G. Hong, J. Walter, M. Hoscheit, A. Lembo, M. Mouchli and J. Lane | 2024 | SMARCA4-Deficient Undifferentiated Esophageal Carcinoma: A Clinical Case Series and Literature Review | Journal of Gastrointestinal Cancer | 10.1007/s12029-024-01060-4 |
| **701** | K. Siddiqi, S. Husain, A. Vidyasagaran, A. Readshaw, M. P. Mishu and A. Sheikh | 2020 | Global burden of disease due to smokeless tobacco consumption in adults: an updated analysis of data from 127 countries | Bmc Medicine | 10.1186/s12916-020-01677-9 |
| **702** | G. Sidorenkov, J. M. Vonk, M. Grzegorczyk, F. O. Cortés-Ibañez and G. H. de Bock | 2023 | Factors associated with SARS-COV-2 positive test in Lifelines | Plos One | 10.1371/journal.pone.0294556 |
| **703** | F. Sierra-Arango, D. M. Castaño, J. D. Forero, E. D. Pérez-Riveros, G. A. Duarte, M. L. Botero, A. Cárdenas and J. De la Hoz-Valle | 2019 | A Randomized Placebo-Controlled <i>N</i>-of-1 Trial: The Effect of Proton Pump Inhibitor in the Management of Gastroesophageal Reflux Disease | Canadian Journal of Gastroenterology and Hepatology | 10.1155/2019/3926051 |
| **704** | G. A. E. Silva, L. F. M. de Rezende, F. D. Gomes, P. R. B. de Souza, C. L. Szwarcwald and J. Eluf-Neto | 2016 | Lifestyle among former cancer patients in Brazil in 2013 | Ciencia & Saude Coletiva | 10.1590/1413-81232015211.24722015 |
| **705** | P. G. D. Silva, R. O. de Sant'ana, C. G. Picanço-Albuquerque, I. J. D. Silva-Fernandes, M. J. B. Bezerra, M. C. D. Luciano and M. V. A. Lima | 2021 | Are pathogenic BRCA1 mutations associated with parotid mucoepidermoid carcinoma? A case report | Oral Surgery Oral Medicine Oral Pathology Oral Radiology | 10.1016/j.oooo.2020.08.017 |
| **706** | P. Sinha, B. H. Haughey, D. Kallogjeri and R. S. Jackson | 2019 | Long-term analysis of transorally resected p16+Oropharynx cancer: Outcomes and prognostic factors | Laryngoscope | 10.1002/lary.27472 |
| **707** | M. D. Skancke, R. A. Grossman, G. Marino, F. J. Brody and G. D. Trachiotis | 2017 | Analysis of Minimally Invasive Esophagectomy at a Single Veterans Affairs Medical Center | Journal of Laparoendoscopic & Advanced Surgical Techniques | 10.1089/lap.2017.0240 |
| **708** | I. Slavu, L. Alecu, A. Tulin, D. Mihaila, V. Braga, T. Voiosu, L. Tomescu and S. Constantinoiu | 2018 | Reintervention Rate Following Emergency Surgery for Crohn Disease | Chirurgia | 10.21614/chirurgia.113.2.227 |
| **709** | K. E. Smedby and M. Ponzoni | 2017 | The aetiology of B-cell lymphoid malignancies with a focus on chronic inflammation and infections | Journal of Internal Medicine | 10.1111/joim.12684 |
[truncated: 73,039 more chars]
