# Supplementary material for: Gastroesophageal disease risk and inhalational exposure a systematic review and meta-analysis
Source: Sci Rep. 2025 Jul 2;15:22581. doi: 10.1038/s41598-025-06620-7 (PMC12218983; doi:10.1038/s41598-025-06620-7)
Supplement: Supplementary file 2 — Supplementary Material 2. [file 41598_2025_6620_MOESM2_ESM.docx]

| **Supplemental Table 2. Studies Removed that didn’t meet Inclusion Criteria (N = 842)** | | | | | | | |
| --- | --- | --- | --- | --- | --- | --- | --- |
| **#** | **Author(s)** | **Year** | **Title** | | | **Journal** | **DOI** |
|  | **PubMed (N = 216)** | | | | | | |
| **1** | No Authors Listed | 2013 | Images of the month: Arytenoid swelling causing dysphagia secondary to ingestion of a hot object | | | Am J Gastroenterol | 10.1038/ajg.2013.29 |
| **2** | No Authors Listed | 2018 | Hot Tea Consumption and the Risk for Esophageal Cancer | | | Ann Intern Med | 10.7326/p17-9054 |
| **3** | Abbes, L., Perrod, G., Rahmi, G. and Cellier, C. | 2017 | Esophageal intramural pseudodiverticulosis, a rare cause of stenosis | | | Clin Res Hepatol Gastroenterol | 10.1016/j.clinre.2017.04.001 |
| **4** | Abiko, S., Shimizu, Y., Miyamoto, S., Ishikawa, M., Matsuda, K., Tsuda, M., Mizushima, T., Yamamoto, K., Ono, S., Kudo, T., Ono, K. and Sakamoto, N. | 2018 | Risk assessment of metachronous squamous cell carcinoma after endoscopic resection for esophageal carcinoma based on the genetic polymorphisms of alcoholdehydrogense-1B aldehyde dehydrogenase-2: temperance reduces the risk | | | J Gastroenterol | 10.1007/s00535-018-1441-7 |
| **5** | Adami, H. O. and Nyrén, O. | 2016 | Enigmas, priorities and opportunities in cancer epidemiology | | | Eur J Epidemiol | 10.1007/s10654-016-0218-7 |
| **6** | Adejumo, A. C., Li, J., Akanbi, O., Adejumo, K. L. and Bukong, T. N. | 2019 | Reduced Prevalence of Alcoholic Gastritis in Hospitalized Individuals Who Consume Cannabis | | | Alcohol Clin Exp Res | 10.1111/acer.13930 |
| **7** | Akizue, N., Okimoto, K., Arai, M., Hirotsu, Y., Amemiya, K., Oura, H., Kaneko, T., Tokunaga, M., Ishikawa, K., Ohta, Y., Taida, T., Saito, K., Maruoka, D., Matsumura, T., Nakagawa, T., Nishimura, M., Chiba, T., Matsushita, K., Mochizuki, H., Yokosuka, O., Omata, M. and Kato, N. | 2021 | Comprehensive mutational analysis of background mucosa in patients with Lugol-voiding lesions | | | Cancer Med | 10.1002/cam4.3905 |
| **8** | Aksamit, T. R., O'Donnell, A. E., Barker, A., Olivier, K. N., Winthrop, K. L., Daniels, M. L. A., Johnson, M., Eden, E., Griffith, D., Knowles, M., Metersky, M., Salathe, M., Thomashow, B., Tino, G., Turino, G., Carretta, B. and Daley, C. L. | 2017 | Adult Patients With Bronchiectasis: A First Look at the US Bronchiectasis Research Registry | | | Chest | 10.1016/j.chest.2016.10.055 |
| **9** | Al-Qadasi, F. A., Shah, S. A. and Ghazi, H. F. | 2017 | Tobacco chewing and risk of gastric cancer: a case-control study in Yemen | | | East Mediterr Health J | 10.26719/2016.22.10.719 |
| **10** | Albert, R. K., Smith, B., Perlman, C. E. and Schwartz, D. A. | 2019 | Is Progression of Pulmonary Fibrosis due to Ventilation-induced Lung Injury? | | | Am J Respir Crit Care Med | 10.1164/rccm.201903-0497PP |
| **11** | Alwan, H., La Rosa, S., Andreas Kopp, P., Germann, S., Maspoli-Conconi, M., Sempoux, C. and Bulliard, J. L. | 2020 | Incidence trends of lung and gastroenteropancreatic neuroendocrine neoplasms in Switzerland | | | Cancer Med | 10.1002/cam4.3524 |
| **12** | Ami, R., Hatta, W., Iijima, K., Koike, T., Ohkata, H., Kondo, Y., Ara, N., Asanuma, K., Asano, N., Imatani, A. and Shimosegawa, T. | 2017 | Factors Associated With Metachronous Gastric Cancer Development After Endoscopic Submucosal Dissection for Early Gastric Cancer | | | J Clin Gastroenterol | 10.1097/mcg.0000000000000620 |
| **13** | Arul, P., Vinoth, B., Alexander, T., Phansalkar, M. and Padhi, S. | 2015 | Correlation of narrow band imaging endoscopy and histopathology in the diagnosis of nonerosive reflux disease | | | Saudi J Gastroenterol | 10.4103/1319-3767.164205 |
| **14** | Asombang, A. W., Kayamba, V., Lisulo, M. M., Trinkaus, K., Mudenda, V., Sinkala, E., Mwanamakondo, S., Banda, T., Soko, R. and Kelly, P. | 2016 | Esophageal squamous cell cancer in a highly endemic region | | | World J Gastroenterol | 10.3748/wjg.v22.i9.2811 |
| **15** | Austin, S., Bailey, D., Chandu, A., Dastaran, M. and Judge, R. | 2015 | Analysis of commonly reported medical conditions amongst patients receiving dental implant therapy in private practice | | | Aust Dent J | 10.1111/adj.12237 |
| **16** | Baba, Y., Yoshida, N., Kinoshita, K., Iwatsuki, M., Yamashita, Y. I., Chikamoto, A., Watanabe, M. and Baba, H. | 2018 | Clinical and Prognostic Features of Patients With Esophageal Cancer and Multiple Primary Cancers: A Retrospective Single-institution Study | | | Ann Surg | 10.1097/sla.0000000000002118 |
| **17** | Badulak, J. H., Schurr, M., Sauaia, A., Ivashchenko, A. and Peltz, E. | 2018 | Defining the criteria for intubation of the patient with thermal burns | | | Burns | 10.1016/j.burns.2018.02.016 |
| **18** | Baldwin-Hunter, B. L., Knotts, R. M., Leeds, S. D., Rubenstein, J. H., Lightdale, C. J. and Abrams, J. A. | 2019 | Use of the Electronic Health Record to Target Patients for Non-endoscopic Barrett's Esophagus Screening | | | Dig Dis Sci | 10.1007/s10620-019-05707-2 |
| **19** | Bang, C. S., Baik, G. H., Kim, J. H., Kim, J. B., Suk, K. T., Yoon, J. H., Kim, Y. S. and Kim, D. J. | 2014 | Peptic ulcer disease in liver cirrhosis and chronic hepatitis: impact of portal hypertension | | | Scand J Gastroenterol | 10.3109/00365521.2014.923501 |
| **20** | Barrea, L., Muscogiuri, G., Modica, R., Altieri, B., Pugliese, G., Minotta, R., Faggiano, A., Colao, A. and Savastano, S. | 2021 | Cardio-Metabolic Indices and Metabolic Syndrome as Predictors of Clinical Severity of Gastroenteropancreatic Neuroendocrine Tumors | | | Front Endocrinol (Lausanne) | 10.3389/fendo.2021.649496 |
| **21** | Bawankule, R., Singh, A., Kumar, K. and Pedgaonkar, S. | 2019 | Oral problems and associated risk indicators in adults in the Russian Federation, India, and China | | | BMC Oral Health | 10.1186/s12903-019-0811-8 |
| **22** | Bhandari, S. and Venkatesan, T. | 2017 | Clinical Characteristics, Comorbidities and Hospital Outcomes in Hospitalizations with Cyclic Vomiting Syndrome: A Nationwide Analysis | | | Dig Dis Sci | 10.1007/s10620-016-4432-7 |
| **23** | Bhat, G. A., Bhat, A. B., Lone, M. M. and Dar, N. A. | 2017 | Association of Genetic Variants of CYP2C19 and CYP2D6 with Esophageal Squamous Cell Carcinoma Risk in Northern India, Kashmir | | | Nutr Cancer | 10.1080/01635581.2017.1299874 |
| **24** | Biagioni, M., Olmos, J. I., Antelo, P., Waldbaum, C., Wonaga, A. and Sordá, J. | 2018 | Dysphagia caused by intramural oesophageal pseudodiverticulosis: An unusual endoscopic finding | | | Gastroenterol Hepatol | 10.1016/j.gastrohep.2017.08.011 |
| **25** | Bikov, A., Lazar, Z., Gyulai, N., Szentkereszty, M., Losonczy, G., Horvath, I. and Galffy, G. | 2015 | Exhaled Breath Condensate pH in Lung Cancer, the Impact of Clinical Factors | | | Lung | 10.1007/s00408-015-9778-7 |
| **26** | Botteri, E., Gallus, S. and Lugo, A. | 2021 | Response to Hoffmeister et al | | | Am J Gastroenterol | 10.14309/ajg.0000000000001152 |
| **27** | Brewczyński, A., Jabłońska, B., Mrowiec, S., Składowski, K. and Rutkowski, T. | 2020 | Nutritional Support in Head and Neck Radiotherapy Patients Considering HPV Status | | | Nutrients | 10.3390/nu13010057 |
| **28** | Brewczyński, A., Jabłońska, B. and Pawlicki, K. | 2017 | Associations Between Nutritional Parameters and Clinicopathologic Factors in Patients with Gastric Cancer: A Comprehensive Study | | | Nutr Cancer | 10.1080/01635581.2017.1324993 |
| **29** | Brodin, N. P., Kabarriti, R., Pankuch, M., Schechter, C. B., Gondi, V., Kalnicki, S., Guha, C., Garg, M. K. and Tomé, W. A. | 2019 | A Quantitative Clinical Decision-Support Strategy Identifying Which Patients With Oropharyngeal Head and Neck Cancer May Benefit the Most From Proton Radiation Therapy | | | Int J Radiat Oncol Biol Phys | 10.1016/j.ijrobp.2018.11.039 |
| **30** | Brouwer, A. F., Eisenberg, M. C. and Meza, R. | 2018 | Case Studies of Gastric, Lung, and Oral Cancer Connect Etiologic Agent Prevalence to Cancer Incidence | | | Cancer Res | 10.1158/0008-5472.Can-17-3467 |
| **31** | Bruley des Varannes, S., Cestari, R., Usova, L., Triantafyllou, K., Alvarez Sanchez, A., Keim, S., Bergmans, P., Marelli, S., Grahl, E. and Ducrotté, P. | 2014 | Classification of adults suffering from typical gastroesophageal reflux disease symptoms: contribution of latent class analysis in a European observational study | | | BMC Gastroenterol | 10.1186/1471-230x-14-112 |
| **32** | Bugter, O., van Iwaarden, D. L. P., Dronkers, E. A. C., de Herdt, M. J., Wieringa, M. H., Verduijn, G. M., Mureau, M. A. M., Ten Hove, I., van Meerten, E., Hardillo, J. A. and Baatenburg de Jong, R. J. | 2019 | Survival of patients with head and neck cancer with metachronous multiple primary tumors is surprisingly favorable | | | Head Neck | 10.1002/hed.25595 |
| **33** | Busch, E. L., Zevallos, J. P. and Olshan, A. F. | 2016 | Gastroesophageal reflux disease and odds of head and neck squamous cell carcinoma in North Carolina | | | Laryngoscope | 10.1002/lary.25716 |
| **34** | Canseco-Ávila, L. M., Zamudio-Castellanos, F. Y., Sánchez-González, R. A., Trujillo-Vizuet, M. G., Domínguez-Arrevillaga, S. and López-López, C. A. | 2019 | Gastric cancer epidemiology in tertiary healthcare in Chiapas | | | Rev Gastroenterol Mex (Engl Ed) | 10.1016/j.rgmx.2018.06.006 |
| **35** | Cao, J., Chen, Z., Tian, C., Yu, J., Zhang, H., Yang, J. and Yang, W. | 2020 | A Shared Susceptibility Locus in the p53 Gene for both Gastric and Esophageal Cancers in a Northwestern Chinese Population | | | Genet Test Mol Biomarkers | 10.1089/gtmb.2020.0192 |
| **36** | Chan, P. K., To, K. F., Tsang, S. H., Lau, C. H., Kwong, W. H. and Chan, Y. H. | 2017 | Human papillomavirus infection and squamous cell carcinoma in Hong Kong: a case-control study | | | Hong Kong Med J |  |
| **37** | Chang, C. C., Chung, Y. H., Liou, C. B., Lee, Y. C., Weng, W. L., Yu, Y. C., Yen, T. H. and Wu, J. M. | 2015 | Influence of residential environment and lifestyle on multiple primary malignancies in Taiwan | | | Asian Pac J Cancer Prev | 10.7314/apjcp.2015.16.8.3533 |
| **38** | Chang, W. L., Lin, M. Y., Kuo, H. Y., Yang, H. B., Cheng, H. C., Lu, C. C. and Sheu, B. S. | 2017 | Osteopontin polymorphism increases gastric precancerous intestinal metaplasia susceptibility in Helicobacter pylori infected male | | | Future Oncol | 10.2217/fon-2017-0006 |
| **39** | Chatzopoulos, G. S., Cisneros, A., Sanchez, M. and Wolff, L. F. | 2018 | Systemic medical conditions and periodontal status in older individuals | | | Spec Care Dentist | 10.1111/scd.12319 |
| **40** | Chen, W., Chen, S., Zhao, L., Zhang, M., Geng, H., Dong, C. and Li, R. | 2022 | Effects of real-ambient PM(2.5) exposure plus lipopolysaccharide on multiple organ damage in mice | | | Hum Exp Toxicol | 10.1177/09603271211061505 |
| **41** | Choi, J. H., Lee, J., Choi, I. J., Kim, Y. W., Ryu, K. W. and Kim, J. | 2016 | Genetic Variation in the TAS2R38 Bitter Taste Receptor and Gastric Cancer Risk in Koreans | | | Sci Rep | 10.1038/srep26904 |
| **42** | Choi, Y. J., Lee, D. H., Han, K. D., Yoon, H., Shin, C. M., Park, Y. S. and Kim, N. | 2017 | Elevated serum gamma-glutamyltransferase is associated with an increased risk of oesophageal carcinoma in a cohort of 8,388,256 Korean subjects | | | PLoS One | 10.1371/journal.pone.0177053 |
| **43** | Chubachi, S., Sato, M., Kameyama, N., Tsutsumi, A., Sasaki, M., Tateno, H., Nakamura, H., Asano, K. and Betsuyaku, T. | 2016 | Identification of five clusters of comorbidities in a longitudinal Japanese chronic obstructive pulmonary disease cohort | | | Respir Med | 10.1016/j.rmed.2016.07.002 |
| **44** | Chung, H. H., Kim, K. O., Lee, S. H., Jang, B. I. and Kim, T. N. | 2017 | Frequency and risk factors of colorectal adenoma in patients with early gastric cancer | | | Intern Med J | 10.1111/imj.13542 |
| **45** | Çolak, Y., Nordestgaard, B. G., Laursen, L. C., Afzal, S., Lange, P. and Dahl, M. | 2017 | Risk Factors for Chronic Cough Among 14,669 Individuals From the General Population | | | Chest | 10.1016/j.chest.2017.05.038 |
| **46** | Dąbrowska, M., Grabczak, E. M., Arcimowicz, M., Domeracka-Kołodziej, A., Domagała-Kulawik, J., Krenke, R., Maskey-Warzęchowska, M., Tarchalska, B. and Chazan, R. | 2015 | Causes of Chronic Cough in Non-smoking Patients | | | Adv Exp Med Biol | 10.1007/5584_2015_153 |
| **47** | Dahiya, D. S., Kichloo, A., Shaka, H., Singh, J., Edigin, E., Solanki, D., Eseaton, P. O. and Wani, F. | 2021 | Gastroparesis with Cannabis Use: A Retrospective Study from the Nationwide Inpatient Sample | | | Postgrad Med | 10.1080/00325481.2021.1940219 |
| **48** | Dai, Q., Cantwell, M. M., Murray, L. J., Zheng, W., Anderson, L. A. and Coleman, H. G. | 2016 | Dietary magnesium, calcium:magnesium ratio and risk of reflux oesophagitis, Barrett's oesophagus and oesophageal adenocarcinoma: a population-based case-control study | | | Br J Nutr | 10.1017/s0007114515004444 |
| **49** | Deng, J., Zhang, J., Wang, Ch, Wei, Q., Zhou, D. and Zhao, K. | 2016 | Methylation and expression of PTPN22 in esophageal squamous cell carcinoma | | | Oncotarget | 10.18632/oncotarget.11581 |
| **50** | Deng, N., Liu, J. W., Sun, L. P., Xu, Q., Duan, Z. P., Dong, N. N. and Yuan, Y. | 2014 | Expression of XPG protein in the development, progression and prognosis of gastric cancer | | | PLoS One | 10.1371/journal.pone.0108704 |
| **51** | Ding, H., Duan, Z., Yang, D., Zhang, Z., Wang, L., Sun, X., Yao, Y., Lin, X., Yang, H., Wang, S. and Chen, J. D. Z. | 2017 | High-resolution manometry in patients with and without globus pharyngeus and/or symptoms of laryngopharyngeal reflux | | | BMC Gastroenterol | 10.1186/s12876-017-0666-x |
| **52** | Dittrich, L., Schwenninger, M. V., Dittrich, K., Pratschke, J., Aigner, F. and Raakow, J. | 2020 | Marginal ulcers after laparoscopic Roux-en-Y gastric bypass: analysis of the amount of daily and lifetime smoking on postoperative risk | | | Surg Obes Relat Dis | 10.1016/j.soard.2019.11.022 |
| **53** | Dong, Y., Chen, J., Chen, Z., Tian, C., Lu, H., Ruan, J. and Yang, W. | 2015 | Evaluating the Association of Eight Polymorphisms with Cancer Susceptibility in a Han Chinese Population | | | PLoS One | 10.1371/journal.pone.0132797 |
| **54** | Dugué, P. A., Bassett, J. K., Wong, E. M., Joo, J. E., Li, S., Yu, C., Schmidt, D. F., Makalic, E., Doo, N. W., Buchanan, D. D., Hodge, A. M., English, D. R., Hopper, J. L., Giles, G. G., Southey, M. C. and Milne, R. L. | 2021 | Biological Aging Measures Based on Blood DNA Methylation and Risk of Cancer: A Prospective Study | | | JNCI Cancer Spectr | 10.1093/jncics/pkaa109 |
| **55** | Eisenberg, J. D., Rosato, E. L., Lavu, H., Yeo, C. J. and Winter, J. M. | 2015 | Delayed Gastric Emptying After Pancreaticoduodenectomy: an Analysis of Risk Factors and Cost | | | J Gastrointest Surg | 10.1007/s11605-015-2865-5 |
| **56** | Elsamadicy, A. A., Adogwa, O., Sergesketter, A., Vuong, V. D., Lydon, E., Behrens, S., Cheng, J., Bagley, C. A. and Karikari, I. O. | 2017 | Reduced Impact of Smoking Status on 30-Day Complication and Readmission Rates After Elective Spinal Fusion (≥3 Levels) for Adult Spine Deformity: A Single Institutional Study of 839 Patients | | | World Neurosurg | 10.1016/j.wneu.2017.07.174 |
| **57** | Enderes, J., Teschke, J., von Websky, M., Manekeller, S., Kalff, J. C. and Glowka, T. R. | 2021 | Active smokers show ameliorated delayed gastric emptying after pancreatoduodenectomy | | | BMC Surg | 10.1186/s12893-021-01311-2 |
| **58** | Endo, K., Nakada, H., Kadota, Y., Mizutani, Y., Shinkawa, N., Onoe, K., Yoshinaga, N., Azuma, M. and Hirai, T. | 2018 | Risk factors for atrophic gastritis in the Japanese young and middle-aged: a study using double-contrast upper gastrointestinal barium X-ray radiography | | | Jpn J Radiol | 10.1007/s11604-018-0782-8 |
| **59** | Fahey, P. P., Page, A., Stone, G. and Astell-Burt, T. | 2020 | Augmenting cancer registry data with health survey data with no cases in common: the relationship between pre-diagnosis health behaviour and post-diagnosis survival in oesophageal cancer | | | BMC Cancer | 10.1186/s12885-020-06990-3 |
| **60** | Floud, S., Hermon, C., Simpson, R. F. and Reeves, G. K. | 2023 | Alcohol consumption and cancer incidence in women: interaction with smoking, body mass index and menopausal hormone therapy | | | BMC Cancer | 10.1186/s12885-023-11184-8 |
| **61** | Fukai, K., Kojimahara, N., Hoshi, K., Toyota, A. and Tatemichi, M. | 2020 | Combined effects of occupational exposure to hazardous operations and lifestyle-related factors on cancer incidence | | | Cancer Sci | 10.1111/cas.14663 |
| **62** | Gaetti-Jardim, E., Jr., Jardim, E. C. G., Schweitzer, C. M., da Silva, J. C. L., Oliveira, M. M., Masocatto, D. C. and Dos Santos, C. M. | 2018 | Supragingival and subgingival microbiota from patients with poor oral hygiene submitted to radiotherapy for head and neck cancer treatment | | | Arch Oral Biol | 10.1016/j.archoralbio.2018.01.003 |
| **63** | Gallagher, L. G., Li, W., Ray, R. M., Romano, M. E., Wernli, K. J., Gao, D. L., Thomas, D. B. and Checkoway, H. | 2015 | Occupational exposures and risk of stomach and esophageal cancers: update of a cohort of female textile workers in Shanghai, China | | | Am J Ind Med | 10.1002/ajim.22412 |
| **64** | Gasenko, E., Isajevs, S., Camargo, M. C., Offerhaus, G. J. A., Polaka, I., Gulley, M. L., Skapars, R., Sivins, A., Kojalo, I., Kirsners, A., Santare, D., Pavlova, J., Sjomina, O., Liepina, E., Tzivian, L., Rabkin, C. S. and Leja, M. | 2019 | Clinicopathological characteristics of Epstein-Barr virus-positive gastric cancer in Latvia | | | Eur J Gastroenterol Hepatol | 10.1097/meg.0000000000001521 |
| **65** | Golpe, R., Martín-Robles, I., Sanjuán-López, P., Cano-Jiménez, E., Castro-Añon, O., Mengual-Macenlle, N. and Pérez-de-Llano, L. | 2017 | Prevalence of Major Comorbidities in Chronic Obstructive Pulmonary Disease Caused by Biomass Smoke or Tobacco | | | Respiration | 10.1159/000472718 |
| **66** | Gong, E. J., Kim, D. H., Jung, H. Y., Lim, H., Ahn, J. Y., Choi, K. S., Lee, J. H., Choi, K. D., Song, H. J., Lee, G. H., Kim, J. H. and Baek, S. | 2014 | Pneumonia after endoscopic resection for gastric neoplasm | | | Dig Dis Sci | 10.1007/s10620-014-3223-2 |
| **67** | Goto, H., Oshikiri, T., Kato, T., Sawada, R., Harada, H., Urakawa, N., Hasegawa, H., Kanaji, S., Yamashita, K., Matsuda, T. and Kakeji, Y. | 2023 | The Influence of Preoperative Smoking Status on Postoperative Complications and Long-Term Outcome Following Thoracoscopic Esophagectomy in Prone Position for Esophageal Carcinoma | | | Ann Surg Oncol | 10.1245/s10434-022-12898-y |
| **68** | Grøtting, M. S., Løberg, E. M., Johannessen, H. O. and Johnson, E. | 2016 | Resection for oesophageal cancer - complications and survival | | | Tidsskr Nor Laegeforen | 10.4045/tidsskr.15.1136 |
| **69** | Hammad, T. A., Thrift, A. P., El-Serag, H. B. and Husain, N. S. | 2019 | Missed Opportunities for Screening and Surveillance of Barrett's Esophagus in Veterans with Esophageal Adenocarcinoma | | | Dig Dis Sci | 10.1007/s10620-018-5336-5 |
| **70** | Hamzaoui, L., Bouassida, M., Ben Mansour, I., Medhioub, M., Ezzine, H., Touinsi, H. and Azouz, M. M. | 2015 | Balloon dilatation in patients with gastric outlet obstruction related to peptic ulcer disease | | | Arab J Gastroenterol | 10.1016/j.ajg.2015.07.004 |
| **71** | Han, R., Chen, G., Li, M., Peng, Z. M. and Xu, L. | 2021 | Screening and clinical significance of lymph node metastasis-related genes within esophagogastric junction adenocarcinoma | | | Cancer Med | 10.1002/cam4.4065 |
| **72** | Hanu, C., Timotin, E., Wong, R., Sur, R. K., Hayward, J. E., Seymour, C. B. and Mothersill, C. E. | 2016 | The influence of smoking on radiation-induced bystander signal production in esophageal cancer patients | | | Environ Res | 10.1016/j.envres.2015.12.030 |
| **73** | Hashibe, M., Morgenstern, H., Cui, Y., Tashkin, D. P., Zhang, Z. F., Cozen, W., Mack, T. M. and Greenland, S. | 2006 | Marijuana use and the risk of lung and upper aerodigestive tract cancers: results of a population-based case-control study | | | Cancer Epidemiol Biomarkers Prev | 10.1158/1055-9965.Epi-06-0330 |
| **74** | Hatta, W., Koike, T., Asonuma, S., Okata, H., Uno, K., Oikawa, T., Iwai, W., Yonechi, M., Fukushi, D., Kayaba, S., Kikuchi, R., Ohyauchi, M., Fushiya, J., Maejima, R., Abe, Y., Kawamura, M., Honda, J., Kondo, Y., Dairaku, N., Norita, K., Watanabe, K., Takahashi, K., Echigo, H., Abe, Y., Endo, H., Okata, T., Hoshi, T., Nakamura, T., Nakaya, N., Iijima, K. and Masamune, A. | 2023 | Smoking history and severe atrophic gastritis assessed by pepsinogen are risk factors for the prevalence of synchronous gastric cancers in patients with gastric endoscopic submucosal dissection: a multicenter prospective cohort study | | | J Gastroenterol | 10.1007/s00535-023-01967-y |
| **75** | He, Y. T., Christos, P. J. and Reisacher, W. R. | 2018 | Airborne and food sensitization patterns in children and adults with eosinophilic esophagitis | | | Int Forum Allergy Rhinol | 10.1002/alr.22095 |
| **76** | Hekking, P. P., Amelink, M., Wener, R. R., Bouvy, M. L. and Bel, E. H. | 2018 | Comorbidities in Difficult-to-Control Asthma | | | J Allergy Clin Immunol Pract | 10.1016/j.jaip.2017.06.008 |
| **77** | Henry, M. A., Lerco, M. M., Ribeiro, P. W. and Rodrigues, M. A. | 2014 | Epidemiological features of esophageal cancer. Squamous cell carcinoma versus adenocarcinoma | | | Acta Cir Bras | 10.1590/s0102-86502014000600007 |
| **78** | Hill, D. A. and Spergel, J. M. | 2018 | Is eosinophilic esophagitis a member of the atopic march? | | | Ann Allergy Asthma Immunol | 10.1016/j.anai.2017.10.003 |
| **79** | Hirabayashi, M., Inoue, M., Sawada, N., Saito, E., Abe, S. K., Hidaka, A., Iwasaki, M., Yamaji, T., Shimazu, T. and Tsugane, S. | 2019 | Helicobacter pylori infection, atrophic gastritis, and risk of pancreatic cancer: A population-based cohort study in a large Japanese population: the JPHC Study | | | Sci Rep | 10.1038/s41598-019-42365-w |
| **80** | Ho, T. W., Tsai, Y. J., Huang, C. T., Lien, A. S. and Lai, F. | 2020 | Impact of tobacco-related chronic obstructive pulmonary disease on developmental trajectories of comorbidities in the Taiwan population | | | Sci Rep | 10.1038/s41598-020-78325-y |
| **81** | Horiuchi, Y., Fujisaki, J., Ishizuka, N., Omae, M., Ishiyama, A., Yoshio, T., Hirasawa, T., Yamamoto, Y., Nagahama, M., Takahashi, H. and Tsuchida, T. | 2017 | Study on Clinical Factors Involved in Helicobacter pylori-Uninfected, Undifferentiated-Type Early Gastric Cancer | | | Digestion | 10.1159/000481817 |
| **82** | Hsu, C. C., Hsu, Y. C., Chang, K. H., Lee, C. Y., Chong, L. W., Lin, C. L., Shang, C. S., Sung, F. C. and Kao, C. H. | 2015 | Depression and the Risk of Peptic Ulcer Disease: A Nationwide Population-Based Study | | | Medicine (Baltimore) | 10.1097/md.0000000000002333 |
| **83** | Huang, Z. S., Chen, W. L., Huang, Z. Q. and Yang, Z. H. | 2016 | Dysphagia in Tongue Cancer Patients Before and After Surgery | | | J Oral Maxillofac Surg | 10.1016/j.joms.2016.03.031 |
| **84** | Ishioka, K., Masaoka, H., Ito, H., Oze, I., Ito, S., Tajika, M., Shimizu, Y., Niwa, Y., Nakamura, S. and Matsuo, K. | 2018 | Association between ALDH2 and ADH1B polymorphisms, alcohol drinking and gastric cancer: a replication and mediation analysis | | | Gastric Cancer | 10.1007/s10120-018-0823-0 |
| **85** | Janson, C., Johannessen, A., Franklin, K., Svanes, C., Schiöler, L., Malinovschi, A., Gislason, T., Benediktsdottir, B., Schlünssen, V., Jõgi, R., Jarvis, D. and Lindberg, E. | 2018 | Change in the prevalence asthma, rhinitis and respiratory symptom over a 20 year period: associations to year of birth, life style and sleep related symptoms | | | BMC Pulm Med | 10.1186/s12890-018-0690-9 |
| **86** | Jayasekara, H., MacInnis, R. J., Lujan-Barroso, L., Mayen-Chacon, A. L., Cross, A. J., Wallner, B., Palli, D., Ricceri, F., Pala, V., Panico, S., Tumino, R., Kühn, T., Kaaks, R., Tsilidis, K., Sánchez, M. J., Amiano, P., Ardanaz, E., Chirlaque López, M. D., Merino, S., Rothwell, J. A., Boutron-Ruault, M. C., Severi, G., Sternby, H., Sonestedt, E., Bueno-de-Mesquita, B., Boeing, H., Travis, R., Sandanger, T. M., Trichopoulou, A., Karakatsani, A., Peppa, E., Tjønneland, A., Yang, Y., Hodge, A. M., Mitchell, H., Haydon, A., Room, R., Hopper, J. L., Weiderpass, E., Gunter, M. J., Riboli, E., Giles, G. G., Milne, R. L., Agudo, A., English, D. R. and Ferrari, P. | 2021 | Lifetime alcohol intake, drinking patterns over time and risk of stomach cancer: A pooled analysis of data from two prospective cohort studies | | | Int J Cancer | 10.1002/ijc.33504 |
| **87** | Jee, Y. H., Shin, A., Lee, J. K. and Oh, C. M. | 2016 | Decreases in Smoking-Related Cancer Mortality Rates Are Associated with Birth Cohort Effects in Korean Men | | | Int J Environ Res Public Health | 10.3390/ijerph13121208 |
| **88** | Jehangir, A. and Parkman, H. P. | 2019 | Cannabinoid Use in Patients With Gastroparesis and Related Disorders: Prevalence and Benefit | | | Am J Gastroenterol | 10.14309/ajg.0000000000000181 |
| **89** | Ji, W., Zheng, W., Li, B., Cao, C. and Mao, W. | 2016 | Influence of body mass index on the long-term outcomes of patients with esophageal squamous cell carcinoma who underwent esophagectomy as a primary treatment: A 10-year medical experience | | | Medicine (Baltimore) | 10.1097/md.0000000000004204 |
| **90** | Jin, Z. Y., Wallar, G., Zhou, J. Y., Yang, J., Han, R. Q., Wang, P. H., Liu, A. M., Gu, X. P., Zhang, X. F., Wang, X. S., Su, M., Hu, X., Sun, Z., Li, G., Mu, L. N., Lu, Q. Y., Liu, X., Li, L. M., He, N., Wu, M., Zhao, J. K. and Zhang, Z. F. | 2019 | Consumption of garlic and its interactions with tobacco smoking and alcohol drinking on esophageal cancer in a Chinese population | | | Eur J Cancer Prev | 10.1097/cej.0000000000000456 |
| **91** | Jo, S., Kim, T. J., Lee, H., Min, Y. W., Min, B. H., Lee, J. H., Son, H. J., Rhee, P. L., Baek, S. Y., Kim, S. W. and Kim, J. J. | 2018 | Associations between Atopic Dermatitis and Risk of Gastric Cancer: A Nationwide Population-based Study | | | Korean J Gastroenterol | 10.4166/kjg.2018.71.1.38 |
| **92** | Jung, K. H., Kim, S. M., Choi, M. G., Lee, J. H., Noh, J. H., Sohn, T. S., Bae, J. M. and Kim, S. | 2015 | Preoperative smoking cessation can reduce postoperative complications in gastric cancer surgery | | | Gastric Cancer | 10.1007/s10120-014-0415-6 |
| **93** | Kakaje, A., Alhalabi, M. M., Alyousbashi, A., Hamid, A. and Mahmoud, Y. | 2020 | Laryngopharyngeal reflux in war-torn Syria and its association with smoking and other risks: an online cross-sectional population study | | | BMJ Open | 10.1136/bmjopen-2020-041183 |
| **94** | Kamangar, F. and Freedman, N. D. | 2018 | Hot Tea and Esophageal Cancer | | | Ann Intern Med | 10.7326/m17-3370 |
| **95** | Kamarajah, S. K., Madhavan, A., Chmelo, J., Navidi, M., Wahed, S., Immanuel, A., Hayes, N., Griffin, S. M. and Phillips, A. W. | 2021 | Impact of Smoking Status on Perioperative Morbidity, Mortality, and Long-Term Survival Following Transthoracic Esophagectomy for Esophageal Cancer | | | Ann Surg Oncol | 10.1245/s10434-021-09720-6 |
| **96** | Kang, M. Y., Jung, J., Koo, J. W., Kim, I., Kim, H. R. and Myong, J. P. | 2021 | Increased risk of gastric cancer in workers with occupational dust exposure | | | Korean J Intern Med | 10.3904/kjim.2019.421 |
| **97** | Kang, S. H., Lim, Y., Lee, H., Kim, J., Chi, S., Min, Y. W., Min, B. H., Lee, J. H., Son, H. J., Ryu, S., Rhee, P. L. and Kim, J. J. | 2016 | A Model for Predicting the Future Risk of Incident Erosive Esophagitis in an Asymptomatic Population Undergoing Regular Check-ups | | | Medicine (Baltimore) | 10.1097/md.0000000000002591 |
| **98** | Kayalı Dinc, A. S., Cayonu, M., Sengezer, T. and Sahin, M. M. | 2020 | Smoking Cessation Improves the Symptoms and the Findings of Laryngeal Irritation | | | Ear Nose Throat J | 10.1177/0145561319881559 |
| **99** | Kayamba, V., Zyambo, K., Mulenga, C., Mwakamui, S., Tembo, M. J., Shibemba, A., Heimburger, D. C., Atadzhanov, M. and Kelly, P. | 2020 | Biomass Smoke Exposure Is Associated With Gastric Cancer and Probably Mediated Via Oxidative Stress and DNA Damage: A Case-Control Study | | | JCO Glob Oncol | 10.1200/go.20.00002 |
| **100** | Khalid, S. I., Eldridge, C., Singh, R., Shanker, R. M., MacDonald, A. M., Chilakapati, S., Smith, J., Mehta, A. I. and Adogwa, O. | 2022 | The impact of smoking and smoking cessation interventions on outcomes following single-level anterior cervical discectomy and fusion procedures | | | Clin Neurol Neurosurg | 10.1016/j.clineuro.2022.107319 |
| **101** | Kilbane, K. S., Girgla, N., Zhao, L., Barnett, S. L., Berezovsky, A., Lagisetty, K., Lin, J. and Reddy, R. M. | 2021 | Adaptive and Maladaptive Coping Mechanisms Used by Patients With Esophageal Cancer After Esophagectomy | | | J Surg Res | 10.1016/j.jss.2020.07.043 |
| **102** | Kim, H. J., Kim, D. K., Sohn, T. S., Lee, J. H. and Lee, G. H. | 2015 | A laparoscopic gastrectomy approach decreases the incidence and severity of emergence agitation after sevoflurane anesthesia | | | J Anesth | 10.1007/s00540-014-1905-8 |
| **103** | Kim, M., Choi, K. S., Suh, M., Jun, J. K., Chuck, K. W. and Park, B. | 2018 | Risky Lifestyle Behaviors among Gastric Cancer Survivors Compared with Matched Non-cancer Controls: Results from Baseline Result of Community Based Cohort Study | | | Cancer Res Treat | 10.4143/crt.2017.129 |
| **104** | Kim, S. A., Kwak, J. H., Eun, C. S., Han, D. S., Kim, Y. S., Song, K. S., Choi, B. Y. and Kim, H. J. | 2023 | Association of Dietary Antioxidant Vitamin Intake and Gastric Cancer Risk According to Smoking Status and Histological Subtypes of Gastric Cancer: A Case-Control Study in Korea | | | Nutr Cancer | 10.1080/01635581.2022.2147274 |
| **105** | Kim, S. H., Yun, J. M., Chang, C. B., Piao, H., Yu, S. J. and Shin, D. W. | 2016 | Prevalence of upper gastrointestinal bleeding risk factors among the general population and osteoarthritis patients | | | World J Gastroenterol | 10.3748/wjg.v22.i48.10643 |
| **106** | Kim, S. W., Lee, J. H., Sim, Y. S., Ryu, Y. J. and Chang, J. H. | 2014 | Prevalence and risk factors for reflux esophagitis in patients with chronic obstructive pulmonary disease | | | Korean J Intern Med | 10.3904/kjim.2014.29.4.466 |
| **107** | Kirenga, B., Chakaya, J., Yimer, G., Nyale, G., Haile, T., Muttamba, W., Mugenyi, L., Katagira, W., Worodria, W., Aanyu-Tukamuhebwa, H., Lugogo, N., Joloba, M., Bekele, A., Makumbi, F., Green, C., de Jong, C., Kamya, M. and van der Molen, T. | 2020 | Phenotypic characteristics and asthma severity in an East African cohort of adults and adolescents with asthma: findings from the African severe asthma project | | | BMJ Open Respir Res | 10.1136/bmjresp-2019-000484 |
| **108** | Kubo, A., Block, G., Quesenberry, C. P., Jr., Buffler, P. and Corley, D. A. | 2014 | Dietary guideline adherence for gastroesophageal reflux disease | | | BMC Gastroenterol | 10.1186/1471-230x-14-144 |
| **109** | Kumar, A., Kim, M. and Lukin, D. J. | 2018 | Helicobacter pylori is associated with increased risk of serrated colonic polyps: Analysis of serrated polyp risk factors | | | Indian J Gastroenterol | 10.1007/s12664-018-0855-8 |
| **110** | Kumar, S., Metz, D. C., Ellenberg, S., Kaplan, D. E. and Goldberg, D. S. | 2020 | Risk Factors and Incidence of Gastric Cancer After Detection of Helicobacter pylori Infection: A Large Cohort Study | | | Gastroenterology | 10.1053/j.gastro.2019.10.019 |
| **111** | La Vecchia, C., Bosetti, C., Bertuccio, P., Castro, C., Pelucchi, C. and Negri, E. | 2014 | Trends in alcohol consumption in Europe and their impact on major alcohol-related cancers | | | Eur J Cancer Prev | 10.1097/CEJ.0b013e32836562f1 |
| **112** | Lee, E., Liu, L., Zhang, J., Stern, M. C., Barzi, A., Hwang, A., Kim, A. E., Hamilton, A. S., Wu, A. H. and Deapen, D. | 2017 | Stomach Cancer Disparity among Korean Americans by Tumor Characteristics: Comparison with Non-Hispanic Whites, Japanese Americans, South Koreans, and Japanese | | | Cancer Epidemiol Biomarkers Prev | 10.1158/1055-9965.Epi-16-0573 |
| **113** | Lee, S. P., Lee, S. Y., Kim, J. H., Sung, I. K., Park, H. S. and Shim, C. S. | 2017 | Factors Related to Upper Gastrointestinal Symptom Generation in 2275 Helicobacter pylori Seroprevalent Adults | | | Dig Dis Sci | 10.1007/s10620-017-4529-7 |
| **114** | Lee, Y. B., Yu, J., Choi, H. H., Jeon, B. S., Kim, H. K., Kim, S. W., Kim, S. S., Park, Y. G. and Chae, H. S. | 2017 | The association between peptic ulcer diseases and mental health problems: A population-based study: a STROBE compliant article | | | Medicine (Baltimore) | 10.1097/md.0000000000007828 |
| **115** | Lee, Y. J., Redd, M., Bayman, L., Frederickson, N., Valestin, J. and Schey, R. | 2015 | Comparison of clinical features in patients with eosinophilic esophagitis living in an urban and rural environment | | | Dis Esophagus | 10.1111/dote.12164 |
| **116** | Li, X., Yu, C., Guo, Y., Bian, Z., Shen, Z., Yang, L., Chen, Y., Wei, Y., Zhang, H., Qiu, Z., Chen, J., Chen, F., Chen, Z., Lv, J. and Li, L. | 2019 | Association between tea consumption and risk of cancer: a prospective cohort study of 0.5 million Chinese adults | | | Eur J Epidemiol | 10.1007/s10654-019-00530-5 |
| **117** | Lin, S., Wang, X., Huang, C., Liu, X., Zhao, J., Yu, I. T. and Christiani, D. C. | 2015 | Consumption of salted meat and its interactions with alcohol drinking and tobacco smoking on esophageal squamous-cell carcinoma | | | Int J Cancer | 10.1002/ijc.29406 |
| **118** | Lin, S., Wang, X., Yano, E., Yu, I., Lan, Y., Courtice, M. N. and Christiani, D. C. | 2014 | Exposure to chrysotile mining dust and digestive cancer mortality in a Chinese miner/miller cohort | | | Occup Environ Med | 10.1136/oemed-2013-101360 |
| **119** | Lin, W. C., Ding, Y. F., Hsu, H. L., Chang, J. H., Yuan, K. S., Wu, A. T. H., Chow, J. M., Chang, C. L., Chen, S. U. and Wu, S. Y. | 2017 | Value and application of trimodality therapy or definitive concurrent chemoradiotherapy in thoracic esophageal squamous cell carcinoma | | | Cancer | 10.1002/cncr.30823 |
| **120** | Liu, X., Wang, X., Lin, S., Lao, X., Zhao, J., Song, Q., Su, X. and Tak-Sun Yu, I. | 2017 | Dietary patterns and the risk of esophageal squamous cell carcinoma: A population-based case-control study in a rural population | | | Clin Nutr | 10.1016/j.clnu.2015.11.009 |
| **121** | Liu, X. L., Wang, R. C., Liu, Y. Y., Chen, H., Qi, C., Hu, L. W., Yi, J. and Wang, W. | 2021 | Risk prediction nomogram for major morbidity related to primary resection for esophageal squamous cancer | | | Medicine (Baltimore) | 10.1097/md.0000000000026189 |
| **122** | Löhler, J., Gerstner, A. O., Bootz, F. and Walther, L. E. | 2014 | Incidence and localization of abnormal mucosa findings in patients consulting ENT outpatient clinics and data analysis of a cancer registry | | | Eur Arch Otorhinolaryngol | 10.1007/s00405-013-2738-z |
| **123** | Ma, G., Zhang, X., Ma, Q., Rong, T., Long, H., Lin, P., Fu, J. and Zhang, L. | 2015 | A novel multivariate scoring system for determining the prognosis of lymph node-negative esophageal squamous cell carcinoma following surgical therapy: an observational study | | | Eur J Surg Oncol | 10.1016/j.ejso.2015.01.013 |
| **124** | Malik, Z., Bayman, L., Valestin, J., Rizvi-Toner, A., Hashmi, S. and Schey, R. | 2017 | Dronabinol increases pain threshold in patients with functional chest pain: a pilot double-blind placebo-controlled trial | | | Dis Esophagus | 10.1111/dote.12455 |
| **125** | Mantziari, S., Hübner, M., Demartines, N. and Schäfer, M. | 2014 | Impact of preoperative risk factors on morbidity after esophagectomy: is there room for improvement? | | | World J Surg | 10.1007/s00268-014-2686-9 |
| **126** | Milliet, F., Bozec, A., Schiappa, R., Viotti, J., Modesto, A., Dassonville, O., Poissonnet, G., Guelfucci, B., Bizeau, A., Vergez, S., Dupret-Bories, A., Garrel, R., Fakhry, N., Santini, L., Lallemant, B., Chambon, G., Sudaka, A., Peyrade, F., Saada-Bouzid, E., Benezery, K., Jourdan-Soulier, F., Chapel, F., Sophie Ramay, A., Roger, P., Galissier, T., Coste, V., Ben Lakdar, A., Guerlain, J., Temam, S., Mirghani, H., Gorphe, P., Chamorey, E. and Culié, D. | 2021 | Synchronous primary neoplasia in patients with oropharyngeal cancer: Impact of tumor HPV status. A GETTEC multicentric study | | | Oral Oncol | 10.1016/j.oraloncology.2020.105041 |
| **127** | Moberg, L., Nilsson, P. M., Samsioe, G., Sallsten, G., Barregard, L., Engström, G. and Borgfeldt, C. | 2017 | Increased blood cadmium levels were not associated with increased fracture risk but with increased total mortality in women: the Malmö Diet and Cancer Study | | | Osteoporos Int | 10.1007/s00198-017-4047-7 |
| **128** | Mochizuki, N., Fujita, T., Kobayashi, M., Yamazaki, Y., Terao, S., Sanuki, T., Okada, A., Adachi, M., Murakami, M., Arisaka, Y., Uno, K., Masuda, A., Yoshida, M., Umegaki, E., Kutsumi, H. and Azuma, T. | 2018 | Factors associated with the presentation of erosive esophagitis symptoms in health checkup subjects: A prospective, multicenter cohort study | | | PLoS One | 10.1371/journal.pone.0196848 |
| **129** | Moeller, M., Pink, C., Endlich, N., Endlich, K., Grabe, H. J., Völzke, H., Dörr, M., Nauck, M., Lerch, M. M., Köhling, R., Holtfreter, B., Kocher, T. and Fuellen, G. | 2017 | Mortality is associated with inflammation, anemia, specific diseases and treatments, and molecular markers | | | PLoS One | 10.1371/journal.pone.0175909 |
| **130** | Mohammadi, N., Alimohammadian, M., Feizesani, A., Poustchi, H., Alizadeh, A., Yaseri, M., Mansournia, M. A. and Sadjadi, A. | 2021 | The marginal causal effect of opium consumption on the upper gastrointestinal cancer death using parametric g-formula: An analysis of 49,946 cases in the Golestan Cohort Study, Iran | | | PLoS One | 10.1371/journal.pone.0246004 |
| **131** | Morais, S., Antunes, L., Bento, M. J. and Lunet, N. | 2019 | Risk and survival of third primary cancers in a population-based cohort of gastric cancer patients | | | Dig Liver Dis | 10.1016/j.dld.2018.12.003 |
| **132** | Morimoto, C., Matsumoto, H., Nagasaki, T., Kanemitsu, Y., Ishiyama, Y., Sunadome, H., Oguma, T., Ito, I., Murase, K., Kawaguchi, T., Tabara, Y., Niimi, A., Muro, S., Matsuda, F., Chin, K. and Hirai, T. | 2021 | Gastroesophageal reflux disease is a risk factor for sputum production in the general population: the Nagahama study | | | Respir Res | 10.1186/s12931-020-01601-y |
| **133** | Mozzanica, F., Ginocchio, D., Barillari, R., Barozzi, S., Maruzzi, P., Ottaviani, F. and Schindler, A. | 2016 | Prevalence and Voice Characteristics of Laryngeal Pathology in an Italian Voice Therapy-seeking Population | | | J Voice | 10.1016/j.jvoice.2015.11.018 |
| **134** | Nguyen, T. H., Thrift, A. P., Ramsey, D., Green, L., Shaib, Y. H., Graham, D. Y. and El-Serag, H. B. | 2014 | Risk factors for Barrett's esophagus compared between African Americans and non-Hispanic Whites | | | Am J Gastroenterol | 10.1038/ajg.2014.351 |
| **135** | Nobel, T. B., Livschitz, J., Xing, X. X., Barbetta, A., Hsu, M., Tan, K. S., Sihag, S., Jones, D. R. and Molena, D. | 2019 | Surveillance Implications of Recurrence Patterns in Early Node-Negative Esophageal Adenocarcinoma | | | Ann Thorac Surg | 10.1016/j.athoracsur.2019.05.066 |
| **136** | Nolen, L. D., Bruden, D., Miernyk, K., McMahon, B. J., Sacco, F., Varner, W., Mezzetti, T., Hurlburt, D., Tiesinga, J. and Bruce, M. G. | 2018 | H. pylori-associated pathologic findings among Alaska native patients | | | Int J Circumpolar Health | 10.1080/22423982.2018.1510715 |
| **137** | Oh, T. K., Jeon, J. H., Lee, J. M., Kim, M. S., Kim, J. H., Lee, S. J. and Eom, W. | 2018 | Chronic Smoking is Not Associated with Increased Postoperative Opioid Use in Patients with Lung Cancer or Esophageal Cancer | | | Pain Physician |  |
| **138** | Okada, E., Nakamura, K., Ukawa, S., Sakata, K., Date, C., Iso, H. and Tamakoshi, A. | 2016 | Dietary Patterns and Risk of Esophageal Cancer Mortality: The Japan Collaborative Cohort Study | | | Nutr Cancer | 10.1080/01635581.2016.1192202 |
| **139** | Özden Mat, D., Firat, S., Aksu, K., Aksu, F. and Duyar, SŞ | 2021 | Obstructive sleep apnea is a determinant of asthma control independent of smoking, reflux, and rhinitis | | | Allergy Asthma Proc | 10.2500/aap.2021.42.200098 |
| **140** | Pandey, A., Tripathi, S. C., Mahata, S., Vishnoi, K., Shukla, S., Misra, S. P., Misra, V., Hedau, S., Mehrotra, R., Dwivedi, M. and Bharti, A. C. | 2014 | Carcinogenic Helicobacter pylori in gastric pre-cancer and cancer lesions: association with tobacco-chewing | | | World J Gastroenterol | 10.3748/wjg.v20.i22.6860 |
| **141** | Parasa, S., Vennalaganti, S., Gaddam, S., Vennalaganti, P., Young, P., Gupta, N., Thota, P., Cash, B., Mathur, S., Sampliner, R., Moawad, F., Lieberman, D., Bansal, A., Kennedy, K. F., Vargo, J., Falk, G., Spaander, M., Bruno, M. and Sharma, P. | 2018 | Development and Validation of a Model to Determine Risk of Progression of Barrett's Esophagus to Neoplasia | | | Gastroenterology | 10.1053/j.gastro.2017.12.009 |
| **142** | Parkman, H. P., Sharkey, E. P., Nguyen, L. A., Yates, K. P., Abell, T. L., Hasler, W. L., Snape, W., Clarke, J., Schey, R., Koch, K. L., Kuo, B., McCallum, R. W., Sarosiek, I., Grover, M., Farrugia, G., Tonascia, J. and Pasricha, P. J. | 2020 | Marijuana Use in Patients with Symptoms of Gastroparesis: Prevalence, Patient Characteristics, and Perceived Benefit | | | Dig Dis Sci | 10.1007/s10620-019-05963-2 |
| **143** | Parmar, G. S., Das, S. and Ingledew, P. A. | 2023 | Quality of Online Information for Esophageal Cancer | | | J Cancer Educ | 10.1007/s13187-022-02198-0 |
| **144** | Parsel, S. M., Iarocci, A. L., Gastañaduy, M., Winters, R. D., Marino, J. P. and McCoul, E. D. | 2020 | Reflux Disease and Laryngeal Neoplasia in Nonsmokers and Nondrinkers | | | Otolaryngol Head Neck Surg | 10.1177/0194599820917669 |
| **145** | Paul, G., Bohle, W. and Zoller, W. | 2019 | Risk Factors for the Development of Esophagorespiratory Fistula in Esophageal Cancer | | | J Gastrointestin Liver Dis | 10.15403/jgld-271 |
| **146** | Pavlidou, E., Papadopoulou, S. K., Tolia, M., Mentzelou, M., Tsoukalas, N., Alexatou, O., Tsiouda, T., Tsourouflis, G., Psara, E., Bikos, V., Kavantzas, N., Kotta-Loizou, I., Dakanalis, A., Vorvolakos, T. and Giaginis, C. | 2023 | Association of Mediterranean Diet Adherence with Disease Progression Characteristics, Lifestyle Factors and Overall Survival in Gastric Cancer Patients | | | Med Sci (Basel) | 10.3390/medsci11040074 |
| **147** | Pedersen, S. B., Nielsen, J. C., Bøtker, H. E., Farkas, D. K., Schmidt, M. and Sørensen, H. T. | 2015 | Implantable cardioverter-defibrillators and subsequent cancer risk: a nationwide population-based cohort study | | | Europace | 10.1093/europace/euv076 |
| **148** | Peng, L. C., Hui, X., Cheng, Z., Bowers, M. R., Moore, J., Cecil, E., Choflet, A., Thompson, A., Muse, M., Kiess, A. P., Page, B. R., Gourin, C. G., Fakhry, C., Szczesniak, M., Maclean, J., Wu, P., Cook, I., McNutt, T. R. and Quon, H. | 2018 | Prospective evaluation of patient reported swallow function with the Functional Assessment of Cancer Therapy (FACT), MD Anderson Dysphagia Inventory (MDADI) and the Sydney Swallow Questionnaire (SSQ) in head and neck cancer patients | | | Oral Oncol | 10.1016/j.oraloncology.2018.05.012 |
| **149** | Phuoc, L. H., Sengngam, K., Ogawa, T., Ngatu, N. R., Ikeda, S., Hoc, T. H., Phu, P. V., Minh, D. T. and Ngoan, L. T. | 2020 | Fruit and Vegetable Intake and Stomach Cancer among Male Adults: A Case-Control Study in Northern Viet Nam | | | Asian Pac J Cancer Prev | 10.31557/apjcp.2020.21.7.2109 |
| **150** | Pilakasiri, A. and Mahakit, P. | 2018 | Prospective study of the prevalence and co-morbidities of obstructive sleep apnea in active-duty army personnel in the three southernmost provinces of Thailand using questionnaire screening | | | Mil Med Res | 10.1186/s40779-018-0186-1 |
| **151** | Plumejeaud, S., Reis, A. P., Tassistro, V., Patinha, C., Noack, Y. and Orsière, T. | 2018 | Potentially harmful elements in house dust from Estarreja, Portugal: characterization and genotoxicity of the bioaccessible fraction | | | Environ Geochem Health | 10.1007/s10653-016-9888-z |
| **152** | Poosari, A., Nutravong, T., Sa-Ngiamwibool, P., Namwat, W., Chatrchaiwiwatana, S. and Ungareewittaya, P. | 2021 | Association between infection with Campylobacter species, poor oral health and environmental risk factors on esophageal cancer: a hospital-based case-control study in Thailand | | | Eur J Med Res | 10.1186/s40001-021-00561-3 |
| **153** | Prabhu, A., Obi, K., Lieberman, D. and Rubenstein, J. H. | 2016 | The Race-Specific Incidence of Esophageal Squamous Cell Carcinoma in Individuals With Exposure to Tobacco and Alcohol | | | Am J Gastroenterol | 10.1038/ajg.2016.346 |
| **154** | Praud, D., Bertuccio, P., Bosetti, C., Turati, F., Ferraroni, M. and La Vecchia, C. | 2014 | Adherence to the Mediterranean diet and gastric cancer risk in Italy | | | Int J Cancer | 10.1002/ijc.28620 |
| **155** | Quan, H., Ouyang, L., Zhou, H., Ouyang, Y. and Xiao, H. | 2019 | The effect of preoperative smoking cessation and smoking dose on postoperative complications following radical gastrectomy for gastric cancer: a retrospective study of 2469 patients | | | World J Surg Oncol | 10.1186/s12957-019-1607-7 |
| **156** | Raghu, G., Morrow, E., Collins, B. F., Ho, L. A., Hinojosa, M. W., Hayes, J. M., Spada, C. A., Oelschlager, B., Li, C., Yow, E., Anstrom, K. J., Mart, D., Xiao, K. and Pellegrini, C. A. | 2016 | Laparoscopic anti-reflux surgery for idiopathic pulmonary fibrosis at a single centre | | | Eur Respir J | 10.1183/13993003.00488-2016 |
| **157** | Ranaldo, N., Losurdo, G., Iannone, A., Principi, M., Barone, M., De Carne, M., Ierardi, E. and Di Leo, A. | 2017 | Tailored therapy guided by multichannel intraluminal impedance pH monitoring for refractory non-erosive reflux disease | | | Cell Death Dis | 10.1038/cddis.2017.436 |
| **158** | Rantanen, T., Oksala, N. and Sand, J. | 2016 | Adenocarcinoma of the Oesophagus and Oesophagogastric Junction: Analysis of Incidence and Risk Factors | | | Anticancer Res |  |
| **159** | Refaat, T., Choi, M., Thomas, T. O., Bacchus, I., Agulnik, M., Pelzer, H. J., Mellott, A. L., Rademaker, A. W., Liu, D., Sathiaseelan, V. and Mittal, B. B. | 2015 | Whole-Field Sequential Intensity-Modulated Radiotherapy for Local-Regional Advanced Head-and-Neck Squamous Cell Carcinoma | | | Am J Clin Oncol | 10.1097/coc.0000000000000001 |
| **160** | Rubenstein, J. H., Morgenstern, H. and Longstreth, K. | 2019 | Clustering of esophageal cancer among white men in the United States | | | Dis Esophagus | 10.1093/dote/doy081 |
| **161** | Saleem, S., Tarar, Z. I., Aziz, M., Alsamman, M. A., Tansel, A. and Abell, T. L. | 2023 | Cannabis Use in Patients with Gastroparesis | | | Cannabis Cannabinoid Res | 10.1089/can.2022.0189 |
| **162** | Şanlı, A., Bekmez, E., Yıldız, G., Erdoğan, B. A., Yılmaz, H. B. and Altın, G. | 2016 | Relationship between smoking and otorhinolaryngological symptoms | | | Kulak Burun Bogaz Ihtis Derg | 10.5606/kbbihtisas.2016.87059 |
| **163** | Schiöler, L., Ruth, M., Jõgi, R., Gislason, T., Storaas, T., Janson, C., Forsberg, B., Sigsgaard, T., Torén, K. and Hellgren, J. | 2015 | Nocturnal GERD - a risk factor for rhinitis/rhinosinusitis: the RHINE study | | | Allergy | 10.1111/all.12615 |
| **164** | Schuman, A. D., Birkeland, A. C., Farlow, J. L., Lyden, T., Blakely, A., Spector, M. E. and Rosko, A. J. | 2021 | Predictors of Stricture and Swallowing Function Following Salvage Laryngectomy | | | Laryngoscope | 10.1002/lary.29215 |
| **165** | Sengngam, K., Hoc, T. H., Phuoc, L. H., Hang, D. V. and Ngoan, L. T. | 2022 | Interaction of Helicobacter pylori Infection with Waterpipe Tobacco Smoking in the Development of Stomach Cancer in Vietnamese Men | | | Asian Pac J Cancer Prev | 10.31557/apjcp.2022.23.4.1199 |
| **166** | Shah, S. C., Nunez, H., Chiu, S., Hazan, A., Chen, S., Wang, S., Itzkowitz, S. and Jandorf, L. | 2020 | Low baseline awareness of gastric cancer risk factors amongst at-risk multiracial/ethnic populations in New York City: results of a targeted, culturally sensitive pilot gastric cancer community outreach program | | | Ethn Health | 10.1080/13557858.2017.1398317 |
| **167** | Shaw, D. E., Sousa, A. R., Fowler, S. J., Fleming, L. J., Roberts, G., Corfield, J., Pandis, I., Bansal, A. T., Bel, E. H., Auffray, C., Compton, C. H., Bisgaard, H., Bucchioni, E., Caruso, M., Chanez, P., Dahlén, B., Dahlen, S. E., Dyson, K., Frey, U., Geiser, T., Gerhardsson de Verdier, M., Gibeon, D., Guo, Y. K., Hashimoto, S., Hedlin, G., Jeyasingham, E., Hekking, P. P., Higenbottam, T., Horváth, I., Knox, A. J., Krug, N., Erpenbeck, V. J., Larsson, L. X., Lazarinis, N., Matthews, J. G., Middelveld, R., Montuschi, P., Musial, J., Myles, D., Pahus, L., Sandström, T., Seibold, W., Singer, F., Strandberg, K., Vestbo, J., Vissing, N., von Garnier, C., Adcock, I. M., Wagers, S., Rowe, A., Howarth, P., Wagener, A. H., Djukanovic, R., Sterk, P. J. and Chung, K. F. | 2015 | Clinical and inflammatory characteristics of the European U-BIOPRED adult severe asthma cohort | | | Eur Respir J | 10.1183/13993003.00779-2015 |
| **168** | Shephard, E. A., Parkinson, M. A. and Hamilton, W. T. | 2019 | Recognising laryngeal cancer in primary care: a large case-control study using electronic records | | | Br J Gen Pract | 10.3399/bjgp19X700997 |
| **169** | Shivappa, N., Hébert, J. R. and Rashidkhani, B. | 2015 | Dietary Inflammatory Index and Risk of Esophageal Squamous Cell Cancer in a Case-Control Study from Iran | | | Nutr Cancer | 10.1080/01635581.2015.1082108 |
| **170** | Soldatova, L., Hrelec, C. and Matrka, L. | 2016 | Can PFTS Differentiate PVFMD From Subglottic Stenosis? | | | Ann Otol Rhinol Laryngol | 10.1177/0003489416665195 |
| **171** | Song, J. H., Yang, S. Y., Lim, J. H., Choi, J. M. and Kim, S. G. | 2017 | The Effect of Helicobacter pylori Eradication on the Metachronous Neoplasm after Endoscopic Resection for Gastric Dysplasia | | | Korean J Gastroenterol | 10.4166/kjg.2017.70.1.27 |
| **172** | Soroush, A., Malekzadeh, R., Roshandel, G., Khoshnia, M., Poustchi, H., Kamangar, F., Brennan, P., Boffetta, P., Dawsey, S. M., Abnet, C. C., Abrams, J. A. and Etemadi, A. | 2023 | Sex and smoking differences in the association between gastroesophageal reflux and risk of esophageal squamous cell carcinoma in a high-incidence area: Golestan Cohort Study | | | Int J Cancer | 10.1002/ijc.34313 |
| **173** | Spaniolas, K., Yang, J., Crowley, S., Yin, D., Docimo, S., Bates, A. T. and Pryor, A. D. | 2018 | Association of Long-term Anastomotic Ulceration After Roux-en-Y Gastric Bypass With Tobacco Smoking | | | JAMA Surg | 10.1001/jamasurg.2018.1616 |
| **174** | Spantideas, N., Drosou, E., Karatsis, A. and Assimakopoulos, D. | 2015 | Voice disorders in the general Greek population and in patients with laryngopharyngeal reflux. Prevalence and risk factors | | | J Voice | 10.1016/j.jvoice.2014.08.006 |
| **175** | Sun, D. and Ye, Q. | 2023 | Mendelian randomization analysis suggests no causal influence of gastroesophageal reflux disease on the susceptibility and prognosis of idiopathic pulmonary fibrosis | | | BMC Pulm Med | 10.1186/s12890-023-02788-8 |
| **176** | Taha, F., Lipsitz, J. D., Galea, S., Demmer, R. T., Talley, N. J. and Goodwin, R. D. | 2014 | Anxiety disorders and risk of self-reported ulcer: a 10-year longitudinal study among US adults | | | Gen Hosp Psychiatry | 10.1016/j.genhosppsych.2014.07.005 |
| **177** | Takeuchi, T., Oota, K., Harada, S., Edogawa, S., Kojima, Y., Sanomura, M., Sakaguchi, M., Hayashi, K., Hongoh, Y., Itabashi, T., Kitae, H., Hoshimoto, M., Takeuchi, N. and Higuchi, K. | 2015 | Characteristics of refractory gastroesophageal reflux disease (GERD) symptoms -is switching proton pump inhibitors based on the patient's CYP2C19 genotype an effective management strategy? | | | Intern Med | 10.2169/internalmedicine.54.3412 |
| **178** | Talagala, I. A., Nawarathne, M. and Arambepola, C. | 2018 | Novel risk factors for primary prevention of oesophageal carcinoma: a case-control study from Sri Lanka | | | BMC Cancer | 10.1186/s12885-018-4975-4 |
| **179** | Tedla, M., Chakrabarti, S., Suchankova, M. and Weickert, M. O. | 2016 | Voice outcomes after thyroidectomy without superior and recurrent laryngeal nerve injury: VoiSS questionnaire and GRBAS tool assessment | | | Eur Arch Otorhinolaryngol | 10.1007/s00405-016-4163-6 |
| **180** | Teixeira, L., Manso, M. C. and Manarte-Monteiro, P. | 2018 | Oral Health-Related Quality of Life Among a Portuguese Sample of Institutionalised Alcoholic Patients under Rehabilitation Therapy | | | Oral Health Prev Dent | 10.3290/j.ohpd.a40719 |
| **181** | Thrift, A. P., Kramer, J. R., Hartman, C. M., Royse, K., Richardson, P., Dong, Y., Raychaudhury, S., Desiderio, R., Sanchez, D., Anandasabapathy, S., White, D. L. and Chiao, E. Y. | 2019 | Risk and Predictors of Esophageal and Stomach Cancers in HIV-Infected Veterans: A Matched Cohort Study | | | J Acquir Immune Defic Syndr | 10.1097/qai.0000000000002038 |
| **182** | Thrumurthy, S. G., Chaudry, M. A., Thrumurthy, S. S. D. and Mughal, M. | 2019 | Oesophageal cancer: risks, prevention, and diagnosis | | | Bmj | 10.1136/bmj.l4373 |
| **183** | Trigueros, J. A., Plaza, V., Domínguez-Ortega, J., Serrano, J., Cisneros, C., Padilla, A., Antón Gironés, M., Mosteiro, M., Martínez Moragón, E., Olaguíbel Rivera, J. M., Delgado, J., García Rivero, J. L., Martínez Rivera, C., Garrido, J. J. and Quirce, S. | 2020 | Asthma, Comorbidities, and Aggravating Circumstances: The GEMA-FORUM II Task Force | | | J Investig Allergol Clin Immunol | 10.18176/jiaci.0460 |
| **184** | Uchihara, T., Yoshida, N., Baba, Y., Yagi, T., Toihata, T., Oda, E., Kuroda, D., Eto, T., Ohuchi, M., Nakamura, K., Sawayama, H., Kinoshita, K., Iwatsuki, M., Ishimoto, T., Sakamoto, Y. and Baba, H. | 2018 | Risk factors for pulmonary morbidities after minimally invasive esophagectomy for esophageal cancer | | | Surg Endosc | 10.1007/s00464-017-5993-z |
| **185** | Udasin, I. G., Sunderram, J. and Calvert, G. | 2023 | The World Trade Center Health Program: Obstructive sleep apnea best practices | | | Arch Environ Occup Health | 10.1080/19338244.2023.2195604 |
| **186** | Ueda, K., Ohishi, W., Cullings, H., Fujiwara, S., Suzuki, G., Hayashi, T., Mitsui, F., Hida, A., Ozasa, K., Ito, M., Chayama, K. and Tahara, E. | 2020 | Modifying Effect of Chronic Atrophic Gastritis on Radiation Risk for Noncardia Gastric Cancer According to Histological Type | | | Radiat Res | 10.1667/rr15482.1 |
| **187** | Ugwuegbu, O., Shibli, F., Kim, Y., Rangan, V., Kurin, M., Ayoub, F., Ganocy, S., Kavitt, R. and Fass, R. | 2024 | The Impact of Chronic Cannabis Use on Esophageal Motility in Patients Referred for Esophageal Manometry | | | J Clin Gastroenterol | 10.1097/mcg.0000000000001887 |
| **188** | Valdez-Solis, E. M., Ramírez-Rentería, C., Ferreira-Hermosillo, A., Molina-Ayala, M., Mendoza-Zubieta, V. and Rodríguez-Pérez, V. | 2017 | Gastroesophageal reflux disease in patients with long standing type 1 diabetes mellitus: utility of two self-report questionnaires in a multifactorial disease | | | Colomb Med (Cali) | 10.25100/cm.v48i3.2801 |
| **189** | van Boven, J. F., Román-Rodríguez, M., Palmer, J. F., Toledo-Pons, N., Cosío, B. G. and Soriano, J. B. | 2016 | Comorbidome, Pattern, and Impact of Asthma-COPD Overlap Syndrome in Real Life | | | Chest | 10.1016/j.chest.2015.12.002 |
| **190** | Vogtmann, E., Flores, R., Yu, G., Freedman, N. D., Shi, J., Gail, M. H., Dye, B. A., Wang, G. Q., Klepac-Ceraj, V., Paster, B. J., Wei, W. Q., Guo, H. Q., Dawsey, S. M., Qiao, Y. L. and Abnet, C. C. | 2015 | Association between tobacco use and the upper gastrointestinal microbiome among Chinese men | | | Cancer Causes Control | 10.1007/s10552-015-0535-2 |
| **191** | Vyas, M. V., Laupacis, A., Austin, P. C., Fang, J., Silver, F. L. and Kapral, M. K. | 2020 | Association Between Immigration Status and Acute Stroke Care: A Retrospective Study | | | Stroke | 10.1161/strokeaha.119.027791 |
| **192** | Waki, K., Ishihara, R., Maekawa, A., Inoue, T., Shoji, A., Matsueda, K., Miyake, M., Fukuda, H., Shichijo, S., Kanesaka, T., Takeuchi, Y., Higashino, K., Uedo, N. and Michida, T. | 2021 | Endoscopic findings in the soft palatal mucosa are associated with the risk of esophageal squamous cell carcinoma | | | J Gastroenterol Hepatol | 10.1111/jgh.15291 |
| **193** | Wang, C., Guan, S., Chen, X., Liu, B., Liu, F., Han, L., Un Nesa, E., Song, Q., Bao, C., Wang, X. and Cheng, Y. | 2015 | Clinical potential of miR-3651 as a novel prognostic biomarker for esophageal squamous cell cancer | | | Biochem Biophys Res Commun | 10.1016/j.bbrc.2015.07.109 |
| **194** | Wang, Q. L., Lagergren, J. and Xie, S. H. | 2019 | Prediction of individuals at high absolute risk of esophageal squamous cell carcinoma | | | Gastrointest Endosc | 10.1016/j.gie.2018.10.025 |
| **195** | Wang, T., Ma, L., Yang, D. L., Wang, H., Bai, Z. L., Zhang, L. J. and Ding, W. Y. | 2017 | Factors predicting dysphagia after anterior cervical surgery: A multicenter retrospective study for 2 years of follow-up | | | Medicine (Baltimore) | 10.1097/md.0000000000007916 |
| **196** | Wang, V. L., Jalilvand, A. D., Gupta, A., Chen, J., Vadlamudi, C. and Perry, K. A. | 2021 | Tobacco use is not associated with increased risk of recurrent reflux 5 years after laparoscopic anti-reflux surgery | | | Surg Endosc | 10.1007/s00464-020-07956-z |
| **197** | Wang, W. L., Chang, I. W., Chen, C. C., Chang, C. Y., Mo, L. R., Lin, J. T., Wang, H. P. and Lee, C. T. | 2015 | Radiofrequency Ablation Versus Endoscopic Submucosal Dissection in Treating Large Early Esophageal Squamous Cell Neoplasia | | | Medicine (Baltimore) | 10.1097/md.0000000000002240 |
| **198** | Wang, Y., Shen, C., Ge, J. and Duan, H. | 2015 | Regular aspirin use and stomach cancer risk in China | | | Eur J Surg Oncol | 10.1016/j.ejso.2015.02.006 |
| **199** | Wang, Z., Koh, W. P., Jin, A., Wang, R. and Yuan, J. M. | 2017 | Composite protective lifestyle factors and risk of developing gastric adenocarcinoma: the Singapore Chinese Health Study | | | Br J Cancer | 10.1038/bjc.2017.7 |
| **200** | Wang, Z., Koh, W. P., Jin, A., Wang, R. and Yuan, J. M. | 2018 | Telomere length and risk of developing gastric adenocarcinoma: The Singapore Chinese Health Study | | | Gastric Cancer | 10.1007/s10120-017-0783-9 |
| **201** | Wienecke, A., Barnes, B., Neuhauser, H. and Kraywinkel, K. | 2015 | Incident cancers attributable to alcohol consumption in Germany, 2010 | | | Cancer Causes Control | 10.1007/s10552-015-0566-8 |
| **202** | Wu, W., Li, L., Qu, C., Wang, M., Liang, S., Gao, X., Bao, X., Wang, L., Liu, H., Han, H., Xu, B., Zhou, Y., Li, B., Zhang, Y., Wang, G. and Zhong, C. | 2019 | Reflux finding score is associated with gastroesophageal flap valve status in patients with laryngopharyngeal reflux disease: a retrospective study | | | Sci Rep | 10.1038/s41598-019-52349-5 |
| **203** | Xie, Y., Huang, S. and Su, Y. | 2016 | Dietary Flavonols Intake and Risk of Esophageal and Gastric Cancer: A Meta-Analysis of Epidemiological Studies | | | Nutrients | 10.3390/nu8020091 |
| **204** | Xiong, G. L., Atkin, A., Moquin, K., Candido, M., Beilenson, P., Kasirye, O., Wasserman, M., Blum, P. and Hilty, D. | 2020 | COVID-19 Transmission in a Psychiatric Long-Term Care Rehabilitation Facility: An Observational Study | | | Prim Care Companion CNS Disord | 10.4088/PCC.20m02765 |
| **205** | Yang, H. Y., Huang, S. H., Shie, R. H. and Chen, P. C. | 2016 | Cancer mortality in a population exposed to nephrite processing | | | Occup Environ Med | 10.1136/oemed-2016-103586 |
| **206** | Yang, J., Wu, H., Wei, S., Xiong, H., Fu, X., Qi, Z., Jiang, Q., Li, W., Hu, G., Yuan, X. and Liao, Z. | 2014 | HPV seropositivity joints with susceptibility loci identified in GWASs at apoptosis associated genes to increase the risk of Esophageal Squamous Cell Carcinoma (ESCC) | | | BMC Cancer | 10.1186/1471-2407-14-501 |
| **207** | Yen, Y. C., Chang, J. H., Lin, W. C., Chiou, J. F., Chang, Y. C., Chang, C. L., Hsu, H. L., Chow, J. M., Yuan, K. S., Wu, A. T. H. and Wu, S. Y. | 2017 | Effectiveness of esophagectomy in patients with thoracic esophageal squamous cell carcinoma receiving definitive radiotherapy or concurrent chemoradiotherapy through intensity-modulated radiation therapy techniques | | | Cancer | 10.1002/cncr.30565 |
| **208** | Yim, M. H., Kim, K. H. and Lee, B. J. | 2021 | The number of household members as a risk factor for peptic ulcer disease | | | Sci Rep | 10.1038/s41598-021-84892-5 |
| **209** | Yoshida, N., Baba, Y., Hiyoshi, Y., Shigaki, H., Kurashige, J., Sakamoto, Y., Miyamoto, Y., Iwatsuki, M., Ishimoto, T., Kosumi, K., Sugihara, H., Harada, K., Tokunaga, R., Izumi, D., Watanabe, M. and Baba, H. | 2016 | Duration of Smoking Cessation and Postoperative Morbidity After Esophagectomy for Esophageal Cancer: How Long Should Patients Stop Smoking Before Surgery? | | | World J Surg | 10.1007/s00268-015-3236-9 |
| **210** | Yoshida, N., Baba, Y., Kuroda, D., Miyamoto, Y., Iwatsuki, M., Hiyoshi, Y., Ishimoto, T., Sawayama, H., Imamura, Y., Watanabe, M. and Baba, H. | 2018 | Clinical utility of exhaled carbon monoxide in assessing preoperative smoking status and risks of postoperative morbidity after esophagectomy | | | Dis Esophagus | 10.1093/dote/doy024 |
| **211** | Yoshida, N., Nakamura, K., Kuroda, D., Baba, Y., Miyamoto, Y., Iwatsuki, M., Hiyoshi, Y., Ishimoto, T., Imamura, Y., Watanabe, M. and Baba, H. | 2018 | Preoperative Smoking Cessation is Integral to the Prevention of Postoperative Morbidities in Minimally Invasive Esophagectomy | | | World J Surg | 10.1007/s00268-018-4572-3 |
| **212** | Yu, C., Tang, H., Guo, Y., Bian, Z., Yang, L., Chen, Y., Tang, A., Zhou, X., Yang, X., Chen, J., Chen, Z., Lv, J. and Li, L. | 2018 | Hot Tea Consumption and Its Interactions With Alcohol and Tobacco Use on the Risk for Esophageal Cancer: A Population-Based Cohort Study | | | Ann Intern Med | 10.7326/m17-2000 |
| **213** | Zamora-Ros, R., Luján-Barroso, L., Bueno-de-Mesquita, H. B., Dik, V. K., Boeing, H., Steffen, A., Tjønneland, A., Olsen, A., Bech, B. H., Overvad, K., Boutron-Ruault, M. C., Racine, A., Fagherazzi, G., Kuhn, T., Katzke, V., Trichopoulou, A., Lagiou, P., Trichopoulos, D., Tumino, R., Panico, S., Vineis, P., Grioni, S., Palli, D., Weiderpass, E., Skeie, G., Huerta, J. M., Sánchez, M. J., Argüelles, M., Amiano, P., Ardanaz, E., Nilsson, L., Wallner, B., Lindkvist, B., Wallström, P., Peeters, P. H., Key, T. J., Khaw, K. T., Wareham, N. J., Freisling, H., Stepien, M., Ferrari, P., Gunter, M. J., Murphy, N., Riboli, E. and González, C. A. | 2014 | Tea and coffee consumption and risk of esophageal cancer: the European prospective investigation into cancer and nutrition study | | | Int J Cancer | 10.1002/ijc.28789 |
| **214** | Zhang, H., Liang, H., Gao, Y., Shang, X., Gong, L., Ma, Z., Sun, K., Tang, P. and Yu, Z. | 2016 | Metastatic lymph node ratio demonstrates better prognostic stratification than pN staging in patients with esophageal squamous cell carcinoma after esophagectomy | | | Sci Rep | 10.1038/srep38804 |
| **215** | Zheng, Y., Cao, X., Wen, J., Yang, H., Luo, K., Liu, Q., Huang, Q., Chen, J. and Fu, J. | 2015 | Smoking affects treatment outcome in patients with resected esophageal squamous cell carcinoma who received chemotherapy | | | PLoS One | 10.1371/journal.pone.0123246 |
| **216** | Zhu, J. F., Feng, X. Y., Zhang, X. W., Wen, Y. S., Lin, P., Cai, L. and Zhang, L. J. | 2015 | Time distribution of recurrence risk of oesophageal squamous cell carcinoma with complete resection (R0) in a Chinese population | | | Eur J Cardiothorac Surg | 10.1093/ejcts/ezv147 |
|  | **Web of Science (N = 626)** | | | | | | |
| **1** | A. I. A. Abd Alrheam, M. M. M. Makhlouf, H. F. Gomaa and A. I. Abd Elneam | 2018 | | Biochemical and Histological Studies on the Effect of Nicotine on the Mucosa of Albino Rat Stomach | Research Journal of Pharmaceutical Biological and Chemical Sciences | |  |
| **2** | M. M. Abd El-Mawgod, N. A. H. Alanazi, M. S. F. Alenezi, M. A. M. Almesned and A. F. K. Alenezi | 2022 | | Awareness of esophageal cancer among the adult population in Arar city, Saudi Arabia | Medical Science | | 10.54905/disssi/v26i129/ms475e2555 |
| **3** | A. H. M. Abdelraheem, A. Z. A. Alharthi, F. B. H. Alziyadi, A. M. A. Sharahili, A. S. M. Alsgoor, M. Alshehri, A. A. M. Alyala and A. M. Albusaamara | 2019 | | PEPTIC ULCER DISEASE AMONG ADULT MALE AND FEMALE PATIENTS AT KING KHALID HOSPITAL FROM 4-7/1434 | Indo American Journal of Pharmaceutical Sciences | | 10.5281/zenodo.2556195 |
| **4** | O. Abdihamid, H. Abdourahman, A. Ibrahim, T. Kareu, A. Hadi, A. Omar and M. Mutebi | 2024 | | Landscape of esophageal cancer in Northern Kenya: experience from Garissa Regional Cancer Center | Ecancermedicalscience | | 10.3332/ecancer.2024.1694 |
| **5** | A. C. Adejumo, J. J. Li, O. Akanbi, K. L. Adejumo and T. N. Bukong | 2019 | | Reduced Prevalence of Alcoholic Gastritis in Hospitalized Individuals Who Consume Cannabis | Alcoholism-Clinical and Experimental Research | | 10.1111/acer.13930 |
| **6** | A. Afzal, M. A. Qayyum and M. H. Shah | 2021 | | Comparative Assessment of Trace Elements in the Blood of Gastric Cancer Patients and Healthy Subjects | Biointerface Research in Applied Chemistry | | 10.33263/briac113.1082410843 |
| **7** | I. Agalliu, Z. G. Chen, T. Wang, R. B. Hayes, N. D. Freedman, S. M. Gapstur and R. D. Burk | 2018 | | Oral Alpha, Beta, and Gamma HPV Types and Risk of Incident Esophageal Cancer | Cancer Epidemiology Biomarkers & Prevention | | 10.1158/1055-9965.Epi-18-0287 |
| **8** | B. Ahmadi, M. Alimohammadian, M. Yaseri, A. Majidi, M. Boreiri, F. Islami, H. Poustchi, M. H. Derakhshan, A. Feizesani, A. Pourshams, C. C. Abnet, P. Brennan, S. M. Dawsey, F. Kamangar, P. Boffetta, A. Sadjadi and R. Malekzadeh | 2016 | | Multimorbidity: Epidemiology and Risk Factors in the Golestan Cohort Study, Iran: A Cross-Sectional Analysis | Medicine | | 10.1097/md.0000000000002756 |
| **9** | M. F. Akl, M. A. Ibrahem, A. Khater, E. El-zahaf, K. Farag and H. Abdallah | 2018 | | Etiologic and Clinicopathological Correlates of Gastric Carcinoma in the Egyptian Delta | Indian Journal of Surgical Oncology | | 10.1007/s13193-018-0754-6 |
| **10** | F. Al Gharaibeh and L. Gibson | 2022 | | The impact of COVID-19 quarantine measures on the mental health of families | Journal of Social Work | | 10.1177/14680173211011705 |
| **11** | M. Al-Azri, J. Al-Kindi, T. Al-Harthi, M. Al-Dahri, S. M. Panchatcharam and A. Al-Maniri | 2019 | | Awareness of Stomach and Colorectal Cancer Risk Factors, Symptoms and Time Taken to Seek Medical Help Among Public Attending Primary Care Setting in Muscat Governorate, Oman | Journal of Cancer Education | | 10.1007/s13187-017-1266-8 |
| **12** | M. A. Al-Ghamdi, M. A. Murad, R. M. Alshiakh, H. J. Abousada, M. B. Alharbi, R. A. Almehdhar, T. S. Aljuhani, A. M. H. Alsobyei, A. H. Alharbi, G. A. Al Ghanem, S. A. Alharbi, A. S. Alonezi, R. S. A. Alghamdi, R. T. Almowllad and F. M. Alfaqih | 2022 | | Prevalence and Complications of Inflammatory Bowel Disease among Saudi Population: A Cross-Sectional Study | Journal of Research in Medical and Dental Science | |  |
| **13** | A. Al-Kaabi, N. S. Baranov, R. S. van der Post, E. J. Schoon, C. Rosman, H. W. M. van Laarhoven, M. Verheij, R. H. A. Verhoeven and P. D. Siersema | 2022 | | Age-specific incidence, treatment, and survival trends in esophageal cancer: a Dutch population-based cohort study | Acta Oncologica | | 10.1080/0284186x.2021.2024878 |
| **14** | R. T. Al-Kasasbeh, N. Korenevskiy, M. S. Alshamasin, F. Ionescu, E. Boitcova and E. Ai-Kasasbeh | 2019 | | Fuzzy prediction and early detection of stomach diseases by means of combined iteration fuzzy models | International Journal of Biomedical Engineering and Technology | |  |
| **15** | A. G. Alghamdi, A. M. Alshareef, A. T. Alzahrani, Z. S. Alharthi, S. S. Alghamdi, A. M. Alghamdi, F. A. Alzahrani and R. A. Alzahrani | 2023 | | Knowledge and Awareness About Gastric Cancer Among the General Population in Al-Baha City, Saudi Arabia | Cureus Journal of Medical Science | | 10.7759/cureus.39589 |
| **16** | A. B. Ali, N. A. Khan, D. T. Nguyen, R. Chihara, E. Y. Chan, E. A. Graviss, B. J. Dunkin and M. P. Kim | 2020 | | Robotic and per-oral endoscopic myotomy have fewer technical complications compared to laparoscopic Heller myotomy | Surgical Endoscopy and Other Interventional Techniques | | 10.1007/s00464-019-07093-2 |
| **17** | I. Ali, Q. Abdo, S. M. Al-Hihi and A. Shawabkeh | 2022 | | Association between ulcerative colitis and <i>Helicobacter pylori</i> infection: A case-control study | Heliyon | | 10.1016/j.heliyon.2022.e08930 |
| **18** | G. Alicandro, P. Bertuccio, G. Collatuzzo, C. Pelucchi, R. Bonzi, L. M. Liao, C. S. Rabkin, R. Sinha, E. Negri, M. Dalmartello, D. Zaridze, D. Maximovich, J. Vioque, M. G. de la Hera, S. Tsugane, A. Hidaka, G. S. Hamada, L. López-Carrillo, R. U. Hernández-Ramírez, R. Malekzadeh, F. Pourfarzi, Z. F. Zhang, R. C. Kurtz, M. C. Camargo, M. P. Curado, N. Lunet, P. Boffetta and C. La Vecchia | 2022 | | The mediating role of combined lifestyle factors on the relationship between education and gastric cancer in the Stomach cancer Pooling (StoP) Project | British Journal of Cancer | | 10.1038/s41416-022-01857-9 |
| **19** | M. Alimohammadian, A. Majidi, M. Yaseri, B. Ahmadi, F. Islami, M. Derakhshan, A. Delavari, M. Amani, A. Feyz-Sani, H. Poustchi, A. Pourshams, A. M. Sadjadi, M. Khoshnia, S. Qaravi, C. C. Abnet, S. Dawsey, P. Brennan, F. Kamangar, P. Boffetta, A. Sadjadi and R. Malekzadeh | 2017 | | Multimorbidity as an important issue among women: results of a gender difference investigation in a large population-based cross-sectional study in West Asia | Bmj Open | | 10.1136/bmjopen-2016-013548 |
| **20** | A. G. R. Alkushi and N. A. M. Elsawy | 2017 | | Quercetin attenuates, indomethacin-induced acute gastric ulcer in rats | Folia Morphologica | | 10.5603/FM.a2016.0067 |
| **21** | E. M. Allen, B. H. Alexander, R. F. MacLehose, H. H. Nelson, G. Ramachandran and J. H. Mandel | 2015 | | Cancer incidence among Minnesota taconite mining industry workers | Annals of Epidemiology | | 10.1016/j.annepidem.2015.08.003 |
| **22** | J. E. Allen, M. Desai, C. A. M. Roumans, S. Vennalaganti, P. Vennalaganti, A. Bansal, G. Falk, D. Lieberman, R. Sampliner, P. Thota, J. Vargo, N. Gupta, F. Moawad, M. Bruno, K. F. Kennedy, S. Gaddam, P. Young, S. Mathur, B. Cash, M. Spaander and P. Sharma | 2021 | | Low Risk of Progression of Barrett's Esophagus to Neoplasia in Women | Journal of Clinical Gastroenterology | | 10.1097/mcg.0000000000001362 |
| **23** | A. E. Almazar, J. D. Penfield, Y. A. Saito and N. J. Talley | 2021 | | Survival Times of Patients With Menetrier's Disease and Risk of Gastric Cancer | Clinical Gastroenterology and Hepatology | | 10.1016/j.cgh.2020.03.017 |
| **24** | A. M. Alsaihati, B. E. Almasoud, S. T. Al Omran, A. H. A. Ali, A. S. Alsulaim, G. S. Almarzoqi, M. S. AlAbbad, B. I. AlKhalifah, M. F. Al Hemaid, M. S. M. Alhagbanim, M. N. A. Al Hajjar and M. A. Almakhayitah | 2018 | | PUBLIC AWARENESS TOWARDS GERD AMONG SAUDI POPULATION IN AL-DAMMAM CITY, SAUDI ARABIA | Indo American Journal of Pharmaceutical Sciences | | 10.5281/zenodo.1480868 |
| **25** | F. M. AlTassan, S. S. Al-Khowaiter, H. E. Alsubki, W. A. Alhamoud, A. K. Niazi and B. M. AlJarallah | 2020 | | Prevalence of gastro-esophageal reflux in diabetic patients at a tertiary hospital in Central Saudi Arabia | Saudi Medical Journal | | 10.15537/smj.2020.2.24844 |
| **26** | A. Amani, A. A. A. Kamrani, R. Fadayevatan, B. Eshrati and M. Rafiee | 2023 | | Burden of Important Risk Factors for Common Cancers Among Older Adults in Markazi Province, Iran in 2016 | Salmand-Iranian Journal of Ageing | | 10.32598/sija.2023.3517.1 |
| **27** | K. Amenu, B. Wieland, B. Szonyi and D. Grace | 2019 | | Milk handling practices and consumption behavior among Borana pastoralists in southern Ethiopia | Journal of Health Population and Nutrition | | 10.1186/s41043-019-0163-7 |
| **28** | F. H. Amin, M. Ghaemi, S. M. Mostafavi, L. Goshayeshi, K. Rezaei, M. Vahed and B. Kiani | 2021 | | A Geospatial database of gastric cancer patients and associated potential risk factors including lifestyle and air pollution | Bmc Research Notes | | 10.1186/s13104-021-05506-x |
| **29** | T. Anuk, S. Kahramanca and O. Kaya | 2018 | | Predictive Parameters for Barrett's Esophagus: Percent Body Fat (PBF) and Visceral Fat Area (VFA) are more Valuable than Body Mass Index (BMI) | Kuwait Medical Journal | |  |
| **30** | B. L. A. Arias, J. P. D. Ríos and J. D. O. Olarte | 2023 | | Association between gastroesophageal reflux and lifestyle: which non-pharmacological interventions improve the management of the disease? | Archivos De Medicina | | 10.30554/archmed.23.2.4967.2023 |
| **31** | E. Armand, D. Boulate, A. Fourdrain, N. A. T. Nguyen, N. Resseguier, G. Brioude, D. Trousse, C. Doddoli, X. B. D'Journo and P. A. Thomas | 2022 | | Benignant and malignant epidemiology among surgical resections for suspicious solitary lung cancer without preoperative tissue diagnosis | European Journal of Cardio-Thoracic Surgery | | 10.1093/ejcts/ezac590 |
| **32** | I. Arshad, S. Zeb, S. H. Keerio, K. Almani, S. A. Raza and M. M. Naeem | 2021 | | Risk Factors Associated with Oral Manifestations and Oral Health Impact of Gastro-Oesophageal Reflux Disease | Pakistan Journal of Medical & Health Sciences | | 10.53350/pjmhs211582202 |
| **33** | A. Aryzbekova, K. T. Juszkiewicz, D. E. Burgess, A. Polski and E. Poleszak | 2015 | | A brief analysis of patients suffering from stomach or duodenal ulcers in Almaty hospital <i>No</i> 1 | Current Issues in Pharmacy and Medical Sciences | | 10.1515/cipms-2015-0079 |
| **34** | M. Asadollahi, O. Firuzi, F. H. Jamebozorgi, M. Alizadeh and A. R. Jassbi | 2019 | | Ethnopharmacological studies, chemical composition, antibacterial and cytotoxic activities of essential oils of eleven <i>Salvia</i> in Iran | Journal of Herbal Medicine | | 10.1016/j.hermed.2018.11.006 |
| **35** | S. Asghar, S. Asghar, S. Shahid, H. Sajjad, J. A. Nasir and M. Usman | 2023 | | Gastroparesis-Related Symptoms in Patients With Type 2 Diabetes Mellitus: Early Detection, Risk Factors, and Prevalence | Cureus Journal of Medical Science | | 10.7759/cureus.35787 |
| **36** | M. O. Avinçsal, H. Shinomiya, M. Teshima, M. Kubo, N. Otsuki, N. Kyota, R. Sasaki, Y. Zen and K. Nibu | 2018 | | Impact of alcohol dehydrogenase-aldehyde dehydrogenase polymorphism on clinical outcome in patients with hypopharyngeal cancer | Head and Neck-Journal for the Sciences and Specialties of the Head and Neck | | 10.1002/hed.25050 |
| **37** | A. Awaya and Y. Kuroiwa | 2020 | | The Relationship between Annual Airborne Pollen Levels and Occurrence of All Cancers, and Lung, Stomach, Colorectal, Pancreatic and Breast Cancers: A Retrospective Study from the National Registry Database of Cancer Incidence in Japan, 1975-2015 | International Journal of Environmental Research and Public Health | | 10.3390/ijerph17113950 |
| **38** | V. Babaei, Y. Saghaei, H. Z. Gohardani, F. Vali and S. Teimourian | 2017 | | Effects of Different Environmental Factors and Virulence Factors, <i>dupA</i> and <i>iceA</i> Genes, of <i>Helicobacter pylori</i> on Peptic Ulcer | Jundishapur Journal of Microbiology | | 10.5812/jjm.40161 |
| **39** | N. Babhadiashar, M. Sotoudeh, E. Azizi, J. Bashiri, R. Didevar, R. Malekzadeh and M. H. Ghahremani | 2014 | | Correlation between Cigarette Smoking and Urine Cotinine Level in Gastric Cancer Patients | Iranian Journal of Pharmaceutical Research | |  |
| **40** | B. Babic, R. R. Datta, W. Schröder, L. M. Schiffmann, T. Schmidt, C. J. Bruns and H. F. Fuchs | 2021 | | Impact of COVID-19 on oncological surgery of the upper gastrointestinal tract | Chirurg | | 10.1007/s00104-021-01489-4 |
| **41** | D. Baik, J. Sheng, K. Schlaffer, F. K. Friedenberg, M. S. Smith and A. C. Ehrlich | 2017 | | Abdominal diameter index is a stronger predictor of prevalent Barrett's esophagus than BMI or waist-to-hip ratio | Diseases of the Esophagus | | 10.1093/dote/dox056 |
| **42** | M. E. Bailey, L. F. Borges, H. J. Goldberg, K. E. Hathorn, S. Gavini, W. K. Lo and W. W. Chan | 2023 | | Abnormal bolus reflux on impedance-pH testing independently predicts 3-year pulmonary outcome and mortality in pulmonary fibrosis | Journal of Gastroenterology and Hepatology | | 10.1111/jgh.16325 |
| **43** | O. Bakr, W. Zhao and D. Corley | 2018 | | Gastroesophageal Reflux Frequency, Severity, Age of Onset, Family History and Acid Suppressive Therapy Predict Barrett Esophagus in a Large Population | Journal of Clinical Gastroenterology | | 10.1097/mcg.0000000000000983 |
| **44** | N. Baras, S. Dahm, J. Haberland, M. Janz, K. Emrich, K. Kraywinkel and A. Salama | 2017 | | Subsequent malignancies among long-term survivors of Hodgkin lymphoma and non-Hodgkin lymphoma: a pooled analysis of German cancer registry data (1990-2012) | British Journal of Haematology | | 10.1111/bjh.14530 |
| **45** | G. D. Batty, C. M. Calvin, C. E. Brett, I. Cukic and I. J. Deary | 2015 | | Childhood Body Weight in Relation to Morbidity From Cardiovascular Disease and Cancer in Older Adulthood: 67-Year Follow-up of Participants in the 1947 Scottish Mental Survey | American Journal of Epidemiology | | 10.1093/aje/kwv154 |
| **46** | C. Bazin, A. Benezech, M. Alessandrini, J. C. Grimaud and V. Vitton | 2018 | | Esophageal Motor Disorders Are a Strong and Independant Associated Factor of Barrett's Esophagus | Journal of Neurogastroenterology and Motility | | 10.5056/jnm17090 |
| **47** | P. Bertuccio, G. Alicandro, M. Rota, C. Pelucchi, R. Bonzi, C. Galeone, F. Bravi, K. C. Johnson, J. Hu, D. Palli, M. Ferraroni, L. López-Carrillo, N. Lunet, A. Ferro, R. Malekzadeh, D. Zaridze, D. Maximovitch, J. Vioque, E. M. Navarrete-Munoz, M. Pakseresht, R. U. Hernández-Ramírez, M. López-Cervantes, M. Ward, F. Pourfarzi, S. Tsugane, A. Hidaka, Z. F. Zhang, R. C. Kurtz, P. Lagiou, A. Lagiou, P. Boffetta, S. Boccia, E. Negri and C. La Vecchia | 2019 | | Citrus fruit intake and gastric cancer: The stomach cancer pooling (StoP) project consortium | International Journal of Cancer | | 10.1002/ijc.32046 |
| **48** | P. P. Bessonov and N. G. Bessonova | 2019 | | CONCOMITANT DISEASES AND RISK FACTORS OF GASTRODUODENAL EROSION IN THE CONDITIONS OF YAKUTIA | Yakut Medical Journal | | 10.25789/ymj.2019.68.20 |
| **49** | D. Bhandari, Y. Y. Zhu, C. Zhang, W. Z. Zhu, A. Alexandridis, A. Etemadi, N. D. Freedman, C. Y. Chang, C. C. Abnet, S. M. Dawsey, M. Inoue-Choi, H. Poustchi, A. Pourshams, P. Boffetta, R. Malekzadeh and B. Blount | 2023 | | Smoke exposure associated with higher urinary benzene biomarker muconic acid (MUCA) in Golestan Cohort Study participants | Biomarkers | | 10.1080/1354750x.2023.2276030 |
| **50** | J. H. Bi, H. Y. Yuan, Y. Jiang, Y. Zhang, W. W. Zheng, L. Zhang, Z. Y. Li, H. L. Li, Y. T. Tan, W. S. Zhao and Y. B. Xiang | 2022 | | Incidence, Mortality Features and Lifetime Risk Estimation of Digestive Tract Cancers in an Urban District of Shanghai, China | Journal of Epidemiology and Global Health | | 10.1007/s44197-022-00047-3 |
| **51** | Y. H. Bi, J. J. Pei, C. F. Hao, W. Yao and H. X. Wang | 2021 | | The relationship between chronic diseases and depression in middle-aged and older adults: A 4-year follow-up study from the China Health and Retirement Longitudinal Study | Journal of Affective Disorders | | 10.1016/j.jad.2021.04.032 |
| **52** | P. J. C. Biselli, J. P. Kirkness, L. Grote, K. Fricke, A. R. Schwartz, P. Smith and H. Schneider | 2017 | | Nasal high-flow therapy reduces work of breathing compared with oxygen during sleep in COPD and smoking controls: a prospective observational study | Journal of Applied Physiology | | 10.1152/japplphysiol.00279.2016 |
| **53** | F. Böhme, K. Racz, C. Sebesta and C. Sebesta | 2023 | | Esophageal Cancer | Wiener Medizinische Wochenschrift | | 10.1007/s10354-022-00972-9 |
| **54** | J. D. Boice, B. Quinn, I. Al-Nabulsi, A. Ansari, P. K. Blake, S. R. Blattnig, E. A. Caffrey, S. S. Cohen, A. P. Golden, K. D. Held, D. W. Jokisch, R. W. Leggett, M. T. Mumma, C. Samuels, J. E. Till, S. Y. Tolmachev, R. C. Yoder, J. Y. Zhou and L. T. Dauer | 2022 | | A million persons, a million dreams: a vision for a national center of radiation epidemiology and biology | International Journal of Radiation Biology | | 10.1080/09553002.2021.1988183 |
| **55** | L. F. Borges, V. Jagadeesan, H. Goldberg, S. Gavini, W. K. Lo, R. Burakoff, N. Feldman and W. W. Chan | 2018 | | Abnormal Bolus Reflux Is Associated With Poor Pulmonary Outcome in Patients With Idiopathic Pulmonary Fibrosis | Journal of Neurogastroenterology and Motility | | 10.5056/jnm18023 |
| **56** | L. F. Borges, S. Salgado, K. E. Hathorn, N. Feldman, T. L. Carroll and W. W. Chan | 2022 | | Failed Swallows on High-Resolution Manometry Independently Correlates With Severity of LPR Symptoms | Journal of Voice | | 10.1016/j.jvoice.2020.09.003 |
| **57** | C. Bosetti, E. Traini, T. Alam, C. A. Allen, G. Carreras, K. Compton, C. Fitzmaurice, L. M. Force, S. Gallus, G. Gorini, J. D. Harvey, J. M. Kocarnik, C. La Vecchia, A. Lugo, M. Naghavi, A. Pennini, C. Piccinelli, L. Ronfani, R. X. Xu and L. Monasta | 2020 | | National burden of cancer in Italy, 1990-2017: a systematic analysis for the global burden of disease study 2017 | Scientific Reports | | 10.1038/s41598-020-79176-3 |
| **58** | E. Botteri, G. Peveri, P. Berstad, V. Bagnardi, G. Hoff, A. K. Heath, A. J. Cross, P. Vineis, L. Dossus, M. Johansson, H. Freisling, K. Matta, I. Huybrechts, S. L. F. Chen, K. B. Borch, T. M. Sandanger, T. H. Nost, C. C. Dahm, C. S. Antoniussen, S. T. Tin, A. Fournier, C. Marques, F. Artaud, M. J. Sanchez, M. Guevara, C. Santiuste, A. Agudo, R. Bajracharya, V. Katzke, F. Ricceri, C. Agnoli, M. M. Bergmann, M. B. Schulze, S. Panico, G. Masala, A. Tjonneland, A. Olsen, T. Stocks, J. Manjer, A. Aizpurua-Atxega, E. Weiderpass, E. Riboli, M. J. Gunter and P. Ferrari | 2024 | | Lifestyle changes in middle age and risk of cancer: evidence from the European Prospective Investigation into Cancer and Nutrition | European Journal of Epidemiology | | 10.1007/s10654-023-01059-4 |
| **59** | E. Bouchard, R. Sharma, N. Bachand, A. A. Gajadhar and E. J. Jenkins | 2017 | | Pathology, clinical signs, and tissue distribution of <i>Toxoplasma gondii</i> in experimentally infected reindeer (<i>Rangifer tarandus</i>) | International Journal for Parasitology-Parasites and Wildlife | | 10.1016/j.ijppaw.2017.08.004 |
| **60** | J. M. Brandenburg, A. C. Jenke, A. Stern, M. T. J. Daum, A. Schulze, R. Younis, P. Petrynowski, T. Davitashvili, V. Vanat, N. Bhasker, S. Schneider, L. Mündermann, A. Reinke, F. R. Kolbinger, V. Jörns, F. Fritz-Kebede, M. Dugas, L. Maier-Hein, R. Klotz, M. Distler, J. Weitz, B. P. Müller-Stich, S. Speidel, S. Bodenstedt and M. Wagner | 2023 | | Active learning for extracting surgomic features in robot-assisted minimally invasive esophagectomy: a prospective annotation study | Surgical Endoscopy and Other Interventional Techniques | | 10.1007/s00464-023-10447-6 |
| **61** | C. S. Brown, B. Lapin, C. Wang, J. L. Goldstein, J. G. Linn, W. Denham, S. P. Haggerty, M. S. Talamonti, J. A. Howington, J. Carbray and M. B. Ujiki | 2015 | | Reflux control is important in the management of Barrett's Esophagus: results from a retrospective 1,830 patient cohort | Surgical Endoscopy and Other Interventional Techniques | | 10.1007/s00464-015-4103-3 |
| **62** | N. Brusselaers, J. Maret-Ouda, P. Konings, H. B. El-Serag and J. Lagergren | 2017 | | Menopausal hormone therapy and the risk of esophageal and gastric cancer | International Journal of Cancer | | 10.1002/ijc.30588 |
| **63** | J. Budzynski, M. Ziólkowski, M. Klopocka and D. Czarnecki | 2016 | | Blood glucose and lipid concentrations after overload are not associated with the risk of alcohol relapse | Drug and Alcohol Dependence | | 10.1016/j.drugalcdep.2016.02.029 |
| **64** | Y. Q. Cai, J. X. Lin, W. B. Wei, P. X. Chen and K. T. Yao | 2022 | | Burden of esophageal cancer and its attributable risk factors in 204 countries and territories from 1990 to 2019 | Frontiers in Public Health | | 10.3389/fpubh.2022.952087 |
| **65** | L. Calderón-Garcidueñas, R. Reynoso-Robles, B. Pérez-Guillé, P. S. Mukherjee and A. Gónzalez-Maciel | 2017 | | Combustion-derived nanoparticles, the neuroenteric system, cervical vagus, hyperphosphorylated alpha synuclein and tau in young Mexico City residents | Environmental Research | | 10.1016/j.envres.2017.08.008 |
| **66** | S. J. Callahan, M. Xia, S. Murray and K. R. Flaherty | 2016 | | Clinical characteristics in patients with asymmetric idiopathic pulmonary fibrosis | Respiratory Medicine | | 10.1016/j.rmed.2016.08.028 |
| **67** | C. M. Calvin, G. D. Batty, G. Der, C. E. Brett, A. Taylor, A. Pattie, I. Cukic and I. J. Deary | 2017 | | Childhood intelligence in relation to major causes of death in 68 year follow-up: prospective population study | Bmj-British Medical Journal | | 10.1136/bmj.j2708 |
| **68** | J. J. Cao, H. X. Xu, W. Li, Z. Q. Guo, Y. Lin, Y. Y. Shi, W. Hu, Y. Ba, S. Y. Li, Z. N. Li, K. H. Wang, J. Wu, Y. He, J. J. Yang, C. H. Xie, F. X. Zhou, X. X. Song, G. Y. Chen, W. J. Ma, S. X. Luo, Z. H. Chen, M. H. Cong, H. Ma, C. L. Zhou, W. Wang, Q. Luo, Y. M. Shi, Y. M. Qi, H. P. Jiang, W. X. Guan, J. Q. Chen, J. X. Chen, Y. Fang, L. Zhou, Y. D. Feng, R. S. Tan, J. W. Ou, Q. C. Zhao, J. X. Wu, X. Lin, L. Q. Yang, Z. M. Fu, C. Wang, L. Deng, T. Li, C. H. Song, H. P. Shi, O. Invest Nutr Status Clinical and O. Chinese Soc Nutr | 2021 | | Nutritional assessment and risk factors associated to malnutrition in patients with esophageal cancer | Current Problems in Cancer | | 10.1016/j.currproblcancer.2020.100638 |
| **69** | A. M. Carvalho, A. M. Miranda, F. A. Santos, A. P. M. Loureiro, R. M. Fisberg and D. M. Marchioni | 2015 | | High intake of heterocyclic amines from meat is associated with oxidative stress | British Journal of Nutrition | | 10.1017/s0007114515000628 |
| **70** | L. Cattelan, F. M. Ghazawi, M. Le, F. Lagacé, E. Savin, A. Zubarev, J. Gantchev, M. Tomaszewski, D. Sasseville, K. Waschke and I. V. Litvinov | 2020 | | Epidemiologic trends and geographic distribution of esophageal cancer in Canada: A national population-based study | Cancer Medicine | | 10.1002/cam4.2700 |
| **71** | M. K. A. Chaar, A. Godin, W. S. Harmsen, C. Wzientek, S. A. Saddoughi, C. L. Hallemeier, S. D. Cassivi, F. C. Nichols, J. S. Reisenauer, K. R. Shen, L. F. Tapias, D. A. Wigle and S. H. Blackmon | 2023 | | Determinants of Long-term Survival Decades After Esophagectomy for Esophageal Cancer | Annals of Thoracic Surgery | | 10.1016/j.athoracsur.2023.05.033 |
| **72** | W. C. Chan, I. Y. Millwood, C. Kartsonaki, H. D. Du, Y. Guo, Y. P. Chen, Z. Bian, R. G. Walters, J. Lv, P. He, C. Hu, L. M. Li, L. Yang, Z. M. Chen and C. K. B. C. Grp | 2021 | | Spicy food consumption and risk of gastrointestinal-tract cancers: findings from the China Kadoorie Biobank | International Journal of Epidemiology | | 10.1093/ije/dyaa275 |
| **73** | P. Y. Chang, W. Y. Huang, C. L. Lin, T. C. Huang, Y. Y. Wu, J. H. Chen and C. H. Kao | 2015 | | Propranolol Reduces Cancer Risk A Population-Based Cohort Study | Medicine | | 10.1097/md.0000000000001097 |
| **74** | Y. K. Chao, H. Y. Ku, C. Y. Chen and T. W. Liu | 2017 | | Induction therapy before surgery improves survival in patients with clinical T3N0 esophageal cancer: a nationwide study in Taiwan | Diseases of the Esophagus | | 10.1093/dote/dox103 |
| **75** | C. Chen, X. Q. Cheng, S. Y. Li, H. H. Chen, M. J. Cui, L. L. Bian and H. Jin | 2021 | | A Novel Signature for Predicting Prognosis of Smoking-Related Squamous Cell Carcinoma | Frontiers in Genetics | | 10.3389/fgene.2021.666371 |
| **76** | C. L. Chen, W. C. Chang, C. H. Yi, J. S. Hung, T. T. Liu, W. Y. Lei and C. S. Hsu | 2019 | | Association of coffee consumption and liver fibrosis progression in patients with HBeAg-negative chronic hepatitis B: A 5-year population-based cohort study | Journal of the Formosan Medical Association | | 10.1016/j.jfma.2018.08.002 |
| **77** | C. X. Chen, T. M. Wen and Q. Y. Zhao | 2019 | | The Change of Laboratory Tests Could Be Predictive Factors for Infection after McKeown Esophagogastrectomy | Biomed Research International | | 10.1155/2019/9718705 |
| **78** | D. N. Chen, N. B. Fan, J. X. Mo, W. D. Wang, R. Q. Wang, Y. F. Chen, J. Hu and Z. S. Wen | 2019 | | Multiple primary malignancies for squamous cell carcinoma and adenocarcinoma of the esophagus | Journal of Thoracic Disease | | 10.21037/jtd.2019.08.51 |
| **79** | J. H. Chen, Z. T. Wu, H. Gao, L. Li, Y. L. Wang, J. J. Han, C. Zhang, P. P. Ding and J. Wu | 2023 | | Association between air temperature and emergency admission for esophagogastric variceal bleeding: a case-crossover study in Beijing, China | Bmc Gastroenterology | | 10.1186/s12876-023-02683-w |
| **80** | L. Chen, G. Y. Zhu, L. She, Y. N. Ding, C. Q. Yang and F. S. Zhu | 2021 | | Analysis of Risk Factors and Establishment of a Prediction Model for Endoscopic Primary Bile Reflux: A Single-Center Retrospective Study | Frontiers in Medicine | | 10.3389/fmed.2021.758771 |
| **81** | W. C. Chen, E. Singh, M. Muchengeti, D. Bradshaw, C. G. Mathew, C. B. de Villiers, C. M. Lewis, T. Waterboer, R. Newton and F. Sitas | 2020 | | Johannesburg Cancer Study (JCS): contribution to knowledge and opportunities arising from 20 years of data collection in an African setting | Cancer Epidemiology | | 10.1016/j.canep.2020.101701 |
| **82** | X. D. Chen, Z. Y. Yuan, M. Lu, Y. C. Zhang, L. Jin and W. M. Ye | 2017 | | Poor oral health is associated with an increased risk of esophageal squamous cell carcinoma - a population-based case-control study in China | International Journal of Cancer | | 10.1002/ijc.30484 |
| **83** | X. Y. Chen, Y. J. Zhang, X. J. Zhou, M. Wang, F. F. Na, L. Zhou, Y. Xu, B. W. Zou, J. X. Xue, Y. M. Liu and Y. L. Gong | 2023 | | Involved-field irradiation or elective-nodal irradiation in neoadjuvant chemo-radiotherapy for locally-advanced esophageal cancer: comprehensive analysis for dosimetry, treatment-related complications, impact on lymphocyte, patterns of failure and survival | Frontiers in Oncology | | 10.3389/fonc.2023.1274924 |
| **84** | Y. Chen, Z. Zhang, G. L. Jiang and K. L. Zhao | 2016 | | Gross tumor volume is the prognostic factor for squamous cell esophageal cancer patients treated with definitive radiotherapy | Journal of Thoracic Disease | | 10.21037/jtd.2016.04.08 |
| **85** | Y. F. Chen, Q. Li, D. T. Chen, J. H. Pan, Y. H. Chen, Z. S. Wen and W. A. Zeng | 2016 | | Prognostic value of pre-operative serum uric acid levels in esophageal squamous cell carcinoma patients who undergo R0 esophagectomy | Cancer Biomarkers | | 10.3233/cbm-160621 |
| **86** | Y. H. Chen, T. M. Jao, Y. L. Shiue, I. J. Feng and P. I. Hsu | 2022 | | Prevalence and risk factors for <i>Candida </i>esophagitis among human immunodeficiency virus-negative individuals | World Journal of Clinical Cases | | 10.12998/wjcc.v10.i30.10896 |
| **87** | Y. L. Chen, T. H. Chen and J. Y. Fang | 2023 | | Burden of gastrointestinal cancers in China from 1990 to 2019 and projection through 2029 | Cancer Letters | | 10.1016/j.canlet.2023.216127 |
| **88** | S. M. Cheng, K. L. Hung, Y. J. Wang, S. P. Ng and H. F. Chiang | 2021 | | Influence of gastric morphology on gastroesophageal reflux in adults An observational study | Medicine | | 10.1097/md.0000000000027241 |
| **89** | Y. L. Cheng, M. H. Yu, Q. Yao, T. He, R. F. Zhang and Z. Q. Long | 2023 | | The impact of indirect notification of a cancer diagnosis and a risk model based on it to predict the prognosis of postoperative stage T3 esophageal cancer patients | Medicine | | 10.1097/md.0000000000035895 |
| **90** | R. Chia, S. Saez-Atienzar, N. Murphy, A. Chiò, C. Blauwendraat, R. H. Roda, P. J. Tienari, H. J. Kaminski, R. Ricciardi, M. Guida, A. De Rosa, L. Petrucci, A. Evoli, C. Provenzano, D. B. Drachman, B. J. Traynor and C. Int Myasthenia Gravis Genomics | 2022 | | Identification of genetic risk loci and prioritization of genes and pathways for myasthenia gravis: a genome-wide association study | Proceedings of the National Academy of Sciences of the United States of America | | 10.1073/pnas.2108672119 |
| **91** | C. L. Chiang, Y. W. Hu, C. H. Wu, Y. T. Chen, C. J. Liu, Y. H. Luo, Y. M. Chen, T. J. Chen, K. C. Su and K. T. Chou | 2016 | | Spectrum of cancer risk among Taiwanese with chronic obstructive pulmonary disease | International Journal of Clinical Oncology | | 10.1007/s10147-016-0983-z |
| **92** | S. H. Chien, C. J. Liu, Y. C. Hong, C. J. Teng, Y. W. Hu, F. C. Ku, C. M. Yeh, T. J. Chiou, J. P. Gau and C. H. Tzeng | 2015 | | Development of second primary malignancy in patients with non-Hodgkin lymphoma: a nationwide population-based study | Journal of Cancer Research and Clinical Oncology | | 10.1007/s00432-015-1979-1 |
| **93** | S. S. Chisholm, J. E. Khoury, M. M. Jamal, C. Palacio, S. Pudhota and K. J. Vega | 2017 | | The frequency of histologically confirmed Barrett's esophagus varies by the combination of ethnicity and gender | Journal of Gastrointestinal Oncology | | 10.21037/jgo.2016.12.07 |
| **94** | B. Chitti, A. Pham, S. Marcott, X. Wang, L. Potters, A. G. Wernicke and B. Parashar | 2018 | | Temporal Changes in Esophageal Cancer Mortality by Geographic Region: A Population-based Analysis | Cureus Journal of Medical Science | | 10.7759/cureus.3596 |
| **95** | S. W. Cho, W. G. Jeong, J. E. Lee, I. J. Oh, S. Y. Song, H. M. Park, H. J. Lee and Y. H. Kim | 2022 | | Clinical implication of interstitial lung abnormality in elderly patients with early-stage non-small cell lung cancer | Thoracic Cancer | | 10.1111/1759-7714.14341 |
| **96** | B. H. Choi, J. Church, J. Sonett and R. P. Kiran | 2023 | | Colonic interposition in esophagectomy: an ACS-NSQIP study | Surgical Endoscopy and Other Interventional Techniques | | 10.1007/s00464-023-10420-3 |
| **97** | R. Chowdhury, S. E. Sarnat, L. Darrow, W. McClellan and K. Steenland | 2014 | | Mortality among participants in a lead surveillance program | Environmental Research | | 10.1016/j.envres.2014.03.008 |
| **98** | S. Christakoudi, A. Kakourou, G. Markozannes, I. Tzoulaki, E. Weiderpass, P. Brennan, M. Gunter, C. C. Dahm, K. Overvad, A. Olsen, A. Tjonneland, M. C. Boutron-Ruault, A. L. Madika, G. Severi, V. Katzke, T. Kühn, M. M. Bergmann, H. Boeing, A. Karakatsani, G. Martimianaki, P. Thriskos, G. Masala, S. Sieri, S. Panico, R. Tumino, F. Ricceri, A. Agudo, D. Redondo-Sánchez, S. M. Colorado-Yohar, O. Mokoroa, O. Melander, T. Stocks, C. Häggström, S. Harlid, B. Bueno-de-Mesquita, C. H. van Gils, R. C. Vermeulen, K. T. Khaw, N. J. Wareham, T. Y. N. Tong, H. Freisling, M. Johansson, H. Lennon, D. Aune, E. Ribolil, D. Trichopoulos, A. Trichopoulou and K. K. Tsilidis | 2020 | | Blood pressure and risk of cancer in the European Prospective Investigation into Cancer and Nutrition | International Journal of Cancer | | 10.1002/ijc.32576 |
| **99** | A. Christopoulou, A. Ardavanis, C. Papandreou, G. Koumakis, G. Papatsimpas, P. Papakotoulas, N. Tsoukalas, C. Andreadis, G. Samelis, P. Papakostas, G. Aravantinos, N. Ziras, M. Souggleri, C. Kalofonos, E. Samantas, P. Makrantonakis, G. Pentheroudakis, A. Athanasiadis, H. Stergiou, A. Bokas, A. Grivas, E. S. Tripodaki, I. Varthalitis, E. Timotheadou and I. Boukovinas | 2022 | | Prophylaxis of cancer-associated venous thromboembolism with low-molecular-weight heparin-tinzaparin: Real world evidence | Oncology Letters | | 10.3892/ol.2022.13235 |
| **100** | T. Cleverley, I. Meredith, D. Sika-Paotonu and J. Gurney | 2023 | | Cancer incidence, mortality and survival for Pacific Peoples in Aotearoa New Zealand | New Zealand Medical Journal | |  |
| **101** | H. Çolak, F. E. Günes, Y. Alahdab and B. Karakoyun | 2022 | | Investigation of Eating Habits in Patients with Functional Dyspepsia | Turkish Journal of Gastroenterology | | 10.5152/tjg.2022.21502 |
| **102** | H. G. Coleman, R. T. Gray, K. W. Lau, C. McCaughey, P. V. Coyle, L. J. Murray and B. T. Johnston | 2016 | | Socio-economic status and lifestyle factors are associated with achalasia risk: A population-based case-control study | World Journal of Gastroenterology | | 10.3748/wjg.v22.i15.4002 |
| **103** | G. Collatuzzo, G. Alicandro, P. Bertuccio, C. Pelucchi, R. Bonzi, D. Palli, M. Ferraroni, W. M. Ye, A. Plymoth, D. Zaridze, D. Maximovich, N. Aragones, G. Castaño-Vinyals, J. Vioque, M. G. de la Hera, Z. F. Zhang, J. F. Hu, L. Lopez-Carrillo, M. López-Cervantes, M. Dalmartello, L. N. Mu, M. H. Ward, C. Rabkin, G. P. Yu, M. C. Camargo, M. P. Curado, N. Lunet, E. Negri, C. La Vecchia and P. Boffetta | 2022 | | Peptic ulcer as mediator of the association between risk of gastric cancer and socioeconomic status, tobacco smoking, alcohol drinking and salt intake | Journal of Epidemiology and Community Health | | 10.1136/jech-2022-219074 |
| **104** | M. B. Cook, S. N. Wood, B. D. Cash, P. Young, R. D. Acosta, R. T. Falk, R. M. Pfeiffer, N. Hu, H. Su, L. M. Wang, C. Y. Wang, B. Gherman, C. Giffen, C. Dykes, V. Turcotte, P. Caron, C. Guillemette, S. M. Dawsey, C. C. Abnet, P. L. Hyland and P. R. Taylor | 2015 | | Association Between Circulating Levels of Sex Steroid Hormones and Barrett's Esophagus in Men: A Case-Control Analysis | Clinical Gastroenterology and Hepatology | | 10.1016/j.cgh.2014.08.027 |
| **105** | C. Correa, C. Mallarino, R. Peña, L. C. Rincón, G. Gracia and I. Zarante | 2014 | | Congenital malformations of pediatric surgical interest: Prevalence, risk factors, and prenatal diagnosis between 2005 and 2012 in the capital city of a developing country. Bogota, Colombia | Journal of Pediatric Surgery | | 10.1016/j.jpedsurg.2014.03.001 |
| **106** | D. S. Dahiya, A. Kichloo, H. Shaka, J. Singh, E. Edigin, D. Solanki, P. O. Eseaton and F. Wani | 2021 | | Gastroparesis with Cannabis Use: A Retrospective Study from the Nationwide Inpatient Sample | Postgraduate Medicine | | 10.1080/00325481.2021.1940219 |
| **107** | G. E. Darling, F. Li, D. Patsios, C. Massey, A. G. Wallis, L. Coate, S. Keshavjee, A. Pierre, M. De Perrot, K. Yasufuku, M. Cypel and T. Waddell | 2015 | | Neoadjuvant chemoradiation and surgery improves survival outcomes compared with definitive chemoradiation in the treatment of stage IIIA N2 non-small-cell lung cancer | European Journal of Cardio-Thoracic Surgery | | 10.1093/ejcts/ezu504 |
| **108** | R. de Mutsert, Q. Sun, W. C. Willett, F. B. Hu and R. M. van Dam | 2014 | | Overweight in Early Adulthood, Adult Weight Change, and Risk of Type 2 Diabetes, Cardiovascular Diseases, and Certain Cancers in Men: a Cohort Study | American Journal of Epidemiology | | 10.1093/aje/kwu052 |
| **109** | D. De Rocchi, A. Zona, R. Turnino, V. Egidi and R. Pasetto | 2021 | | Mortality temporal trends and cancer incidence profiles of residents in the petrochemical industrially contaminated town of Gela (Sicily, Italy) | Annali Dell Istituto Superiore Di Sanita | | 10.4415/ann_21_02_10 |
| **110** | A. Del Monaco, C. Dimitriadis, S. Xie, G. Benke, M. R. Sim and K. Walker-Bone | 2023 | | Workers in Australian prebake aluminium smelters: update on risk of mortality and cancer incidence in the Healthwise cohort | Occupational and Environmental Medicine | | 10.1136/oemed-2022-108605 |
| **111** | D. J. Desilets, B. H. Nathanson and F. Navab | 2014 | | Barrett's Esophagus in Practice: Gender and Screening Issues | Journal of Mens Health | | 10.1089/jomh.2014.0037 |
| **112** | L. Dhaliwal, D. C. Codipilly, P. Gandhi, M. L. Johnson, R. Lansing, K. N. K. Wang, C. L. Leggett, D. A. Katzka and P. G. Iyer | 2021 | | Neoplasia Detection Rate in Barrett's Esophagus and Its Impact on Missed Dysplasia: Results from a Large Population-Based Database | Clinical Gastroenterology and Hepatology | | 10.1016/j.cgh.2020.07.034 |
| **113** | P. K. Dhillon, P. Mathur, A. Nandakumar, C. Fitzmaurice, G. A. Kumar, R. Mehrotra, D. K. Shukla, G. K. Rath, P. C. Gupta, R. Swaminathan, J. S. Thakur, S. Dey, C. Allen, R. A. Badwe, R. Dikshit, R. S. Dhaliwal, T. Kaur, A. C. Kataki, R. N. Visvveswara, P. Gangadharan, E. Dutta, M. Furtado, C. M. Varghese, D. Bhardwaj, P. Muraleedharan, C. M. Odell, S. Glenn, M. S. Bal, P. P. Bapsy, J. Bennett, V. K. Bodal, J. K. Chakma, S. Chakravarty, M. Chaturvedi, P. Das, V. Deshmane, N. Gangane, J. Harvey, P. Jayalekshmi, K. Jerang, S. C. Johnson, P. K. Julka, D. Kaushik, V. Khamo, S. Koyande, M. Kutz, W. B. Langstieh, K. B. Lingegowda, R. C. Mahajan, J. Mahanta, G. Majumdar, N. Manoharan, A. Mathew, B. M. Nene, S. Pati, P. K. Pradhan, V. Raina, R. Rama, C. Ramesh, K. Sathishkumar, K. Schelonka, P. Sebastian, K. Shackelford, J. Shah, Shanta, J. D. Sharma, A. Shrivastava, S. Tawsik, B. B. Tyagi, K. Vaitheeswaran, E. Vallikad, Y. Verma, E. Zomawia, S. S. Lim, T. Vos, R. Dandona, K. S. Reddy, M. Naghavi, C. J. L. Murray, S. Swaminathan, L. Dandona and I. India State-Level Dis Burden | 2018 | | The burden of cancers and their variations across the states of India: the Global Burden of Disease Study 1990-2016 | Lancet Oncology | | 10.1016/s1470-2045(18)30447-9 |
| **114** | C. G. Dietrich, T. Kottmann, J. Labenz, K. Streetz and P. Hellebrandt | 2019 | | The "Aachen sings" study ("Aachen choir engagement study into GERD symptoms"): moderate singing and breathing exercises in a choir reduce reflux symptoms - a cohort study in non-specialist choristers | Zeitschrift Fur Gastroenterologie | | 10.1055/a-0855-4339 |
| **115** | N. N. Ding, Y. S. Mao, J. He, S. G. Gao, Y. Zhao, D. Yang, K. L. Sun, G. Y. Cheng, J. W. Mu, Q. Xue, D. L. Wang, J. Zhao, Y. S. Gao, X. Y. Liu, D. K. Fang, J. Li, Y. G. Wang, J. F. Huang, B. Wang and L. Z. Zhang | 2017 | | Experiences in the management of anastomotic leakages and analysis of the factors affecting leakage healing in patients with esophagogastric junction cancer | Journal of Thoracic Disease | | 10.21037/jtd.2017.02.34 |
| **116** | S. Docimo, A. Mathew, A. J. Shope, J. S. Winder, R. S. Haluck and E. M. Pauli | 2017 | | Reduced postoperative pain scores and narcotic use favor per-oral endoscopic myotomy over laparoscopic Heller myotomy | Surgical Endoscopy and Other Interventional Techniques | | 10.1007/s00464-016-5034-3 |
| **117** | J. Dong, M. F. Buas, P. Gharahkhani, B. J. Kendall, L. Onstad, S. S. Zhao, L. A. Anderson, A. H. Wu, W. M. Ye, N. C. Bird, L. Bernstein, W. H. Chow, M. D. Gammon, G. Liu, C. Caldas, P. D. Pharoah, H. A. Risch, P. G. Iyer, B. J. Reid, L. J. Hardie, J. Lagergren, N. J. Shaheen, D. A. Corley, R. C. Fitzgerald, D. C. Whiteman, T. L. Vaughan, A. P. Thrift, Stomach and S. Oesophageal Canc | 2018 | | Determining Risk of Barrett's Esophagus and Esophageal Adenocarcinoma Based on Epidemiologic Factors and Genetic Variants | Gastroenterology | | 10.1053/j.gastro.2017.12.003 |
| **118** | J. Dong, P. Gharahkhani, W. H. Chow, M. D. Gammon, G. Liu, C. Caldas, A. H. Wu, W. M. Ye, L. Onstad, L. A. Anderson, L. Bernstein, P. D. Pharoah, H. A. Risch, D. A. Corley, R. C. Fitzgerald, P. G. Iyer, B. J. Reid, J. Lagergren, N. J. Shaheen, T. L. Vaughan, S. MacGregor, S. Love, C. Palles, I. Tomlinson, I. Gockel, A. May, C. Gerges, M. Anders, A. C. Böhmer, J. Becker, N. Kreuser, R. Thieme, T. Noder, M. Venerito, L. Veits, T. Schmidt, C. Schmidt, J. R. Izbicki, A. H. Hölscher, H. K. Lang, D. Lorenz, B. Schumacher, R. Mayershofer, Y. Vashist, K. Ott, M. Vieth, J. Weismüller, M. M. Nöthen, S. Moebus, M. Knapp, W. H. M. Peters, H. Neuhaus, T. Rösch, C. Ell, J. Jankowski, J. Schumacher, R. E. Neale, D. C. Whiteman, A. P. Thrift and S. Stomach Esophageal Canc | 2019 | | No Association Between Vitamin D Status and Risk of Barrett's Esophagus or Esophageal Adenocarcinoma: A Mendelian Randomization Study | Clinical Gastroenterology and Hepatology | | 10.1016/j.cgh.2019.01.041 |
| **119** | N. M. Donin, L. Kwan, A. T. Lenis, A. Drakaki and K. Chamie | 2019 | | Second primary lung cancer in United States Cancer Survivors, 1992-2008 | Cancer Causes & Control | | 10.1007/s10552-019-01161-7 |
| **120** | S. Dorosti, S. J. Ghoushchi, E. Sobhrakhshankhah, M. Ahmadi and A. Sharifi | 2020 | | Application of gene expression programming and sensitivity analyses in analyzing effective parameters in gastric cancer tumor size and location | Soft Computing | | 10.1007/s00500-019-04507-0 |
| **121** | J. Dudley, T. Wieczorek, M. Selig, H. Cheung, J. Shen, R. Odze, V. Deshpande and L. Zukerberg | 2017 | | Clinicopathological characteristics of invasive gastric <i>Helicobacter pylori</i> | Human Pathology | | 10.1016/j.humpath.2016.09.029 |
| **122** | K. Dzobo, N. Hassen, D. A. Senthebane, N. E. Thomford, A. Rowe, H. Shipanga, A. Wonkam, M. I. Parker, S. Mowla and C. Dandara | 2018 | | Chemoresistance to Cancer Treatment: Benzo-α-Pyrene as Friend or Foe? | Molecules | | 10.3390/molecules23040930 |
| **123** | B. Ege, T. Dinç, B. D. Yildiz, Z. Balci and H. Bozkaya | 2015 | | Utility of Endoscopy for Diagnosis of Barrett in a Non-Western Society: Endoscopic and Histopathologic Correlation | International Surgery | | 10.9738/intsurg-d-14-00167.1 |
| **124** | M. Eisa, A. Sandhu, R. Prakash, S. J. Ganocy and R. Fass | 2020 | | The Risk of Acute Myocardial Infarction in Patients With Gastroesophageal Reflux Disease | Journal of Neurogastroenterology and Motility | | 10.5056/jnm19192 |
| **125** | J. A. Elliott, S. Casey, C. F. Murphy, N. G. Docherty, N. Ravi, P. Beddy, J. V. Reynolds and C. W. le Roux | 2019 | | Risk factors for loss of bone mineral density after curative esophagectomy | Archives of Osteoporosis | | 10.1007/s11657-018-0556-z |
| **126** | O. Eslami, M. Shahraki, A. Bahari and T. Shahraki | 2017 | | Dietary habits and obesity indices in patients with gastro-esophageal reflux disease: a comparative cross-sectional study | Bmc Gastroenterology | | 10.1186/s12876-017-0699-1 |
| **127** | A. Etemadi, S. Hariri, H. Hassanian-moghaddam, H. Poustchi, G. Roshandel, A. Shayanrad, F. Kamangar, P. Boffetta, P. Brennan, P. I. Dargan, S. M. Dawsey, R. L. Jones, N. D. Freedman, R. Malekzadeh and C. C. Abnet | 2022 | | Lead poisoning among asymptomatic individuals with a long-term history of opiate use in Golestan Cohort Study | International Journal of Drug Policy | | 10.1016/j.drugpo.2022.103695 |
| **128** | A. Etemadi, F. Kamangar, F. Islami, H. Poustchi, A. Pourshams, P. Brennan, P. Boffetta, R. Malekzadeh, S. M. Dawsey, C. C. Abnet and A. Emadi | 2015 | | Mortality and cancer in relation to ABO blood group phenotypes in the Golestan Cohort Study | Bmc Medicine | | 10.1186/s12916-014-0237-8 |
| **129** | A. Etemadi, H. Khademi, F. Kamangar, N. D. Freedman, C. C. Abnet, P. Brennan, R. Malekzadeh and T. Golestan Cohort Study | 2017 | | Hazards of cigarettes, smokeless tobacco and waterpipe in a Middle Eastern Population: a Cohort Study of 50 000 individuals from Iran | Tobacco Control | | 10.1136/tobaccocontrol-2016-053245 |
| **130** | R. Everatt, I. Kuzmickiene, E. Davidaviciene and S. Cicenas | 2017 | | Non-pulmonary cancer risk following tuberculosis: a nationwide retrospective cohort study in Lithuania | Infectious Agents and Cancer | | 10.1186/s13027-017-0143-8 |
| **131** | C. Y. Fan, W. Y. Huang, C. S. Lin, Y. F. Su, C. H. Lo, C. C. Tsao, M. Y. Liu, C. L. Lin and C. H. Kao | 2017 | | Risk of second primary malignancies among patients with prostate cancer: A population-based cohort study | Plos One | | 10.1371/journal.pone.0175217 |
| **132** | J. H. Fan, W. Y. Sun, H. Yang, X. K. Wang, C. C. Abnet and Y. L. Qiao | 2023 | | Short-term and long-term effect of nutrition intervention in the Linxian Dysplasia Nutrition Intervention Trial and the reason for disappearance of the intervention effect: A cohort study | Cancer | | 10.1002/cncr.34761 |
| **133** | S. Faramarzi, B. Kiani, S. Faramarzi and N. Firouraghi | 2024 | | Cancer patterns in Iran: a gender-specific spatial modelling of cancer incidence during 2014-2017 | Bmc Cancer | | 10.1186/s12885-024-11940-4 |
| **134** | E. H. Farbu, M. Skandfer, C. Nielsen, T. Brenn, A. Stubhaug and A. C. Höper | 2019 | | Working in a cold environment, feeling cold at work and chronic pain: a cross-sectional analysis of the Tromso Study | Bmj Open | | 10.1136/bmjopen-2019-031248 |
| **135** | M. Fasullo, A. Sreenivasen, E. Holzwanger, C. Lavender, M. Patel, T. Shah, P. Mutha, R. F. Yacavone, K. Sultan, A. J. Trindade and G. Smallfield | 2021 | | Co-existing inflammatory bowel disease and Barrett's esophagus is associated with esophageal dysplasia: a propensity score-matched cohort | Endoscopy International Open | | 10.1055/a-1526-0507 |
| **136** | M. Fattouh, G. Y. Chang, T. J. Ow, K. Shifteh, G. Rosenblatt, V. M. Patel, R. V. Smith, M. B. Prystowsky and N. F. Schlecht | 2019 | | Association between pretreatment obesity, sarcopenia, and survival in patients with head and neck cancer | Head and Neck-Journal for the Sciences and Specialties of the Head and Neck | | 10.1002/hed.25420 |
| **137** | X. Feng, Z. L. Hua, Q. Zhou, A. W. Shi, T. Q. Song, D. F. Qian, R. Chen, G. Q. Wang, W. Q. Wei, J. Y. Zhou, J. J. Wang, G. Shao and X. Wang | 2021 | | Prevalence and coprevalence of modifiable risk factors for upper digestive tract cancer among residents aged 40-69 years in Yangzhong city, China: a cross-sectional study | Bmj Open | | 10.1136/bmjopen-2020-042006 |
| **138** | X. Feng, J. H. Zhu, Z. L. Hua, Q. P. Shi, J. Y. Zhou and P. F. Luo | 2023 | | The prevalence and determinant of overweight and obesity among residents aged 40-69 years in high-risk regions for upper gastrointestinal cancer in southeast China | Scientific Reports | | 10.1038/s41598-023-35477-x |
| **139** | A. Ferro, V. Rosato, M. Rota, A. R. Costa, S. Morais, C. Pelucchi, K. C. Johnson, J. F. Hu, D. Palli, M. Ferraroni, Z. F. Zhang, R. Bonzi, G. P. Yu, B. Peleteiro, L. López-Carrillo, S. Tsugane, G. S. Hamada, A. Hidaka, D. Zaridze, D. Maximovitch, J. Vioque, E. M. Navarrete-Munoz, N. Aragonés, V. Martín, R. U. Hernández-Ramírez, P. Bertuccio, M. H. Ward, R. Malekzadeh, F. Pourfarzi, L. N. Mu, M. López-Cervantes, R. Persiani, R. C. Kurtz, A. Lagiou, P. Lagiou, P. Boffetta, S. Boccia, E. Negri, M. C. Camargo, M. P. Curado, C. La Vecchia and N. Lunet | 2020 | | Meat intake and risk of gastric cancer in the Stomach cancer Pooling (StoP) project | International Journal of Cancer | | 10.1002/ijc.32707 |
| **140** | E. Finocchio, F. Locatelli, F. Sanna, R. Vesentini, P. Marchetti, G. Spiteri, L. Antonicelli, S. Battaglia, R. Bono, A. G. Corsico, M. Ferrari, N. Murgia, P. Pirina, M. Olivieri and G. Verlato | 2021 | | Gastritis and gastroesophageal reflux disease are strongly associated with non-allergic nasal disorders | Bmc Pulmonary Medicine | | 10.1186/s12890-020-01364-8 |
| **141** | S. Flashner, C. Martin, N. Matsuura, M. Shimonosono, Y. Tomita, M. Morimoto, O. Okolo, V. X. Yu, A. S. Parikh, A. J. P. Klein-Szanto, K. Yan, J. T. Gabre, C. Lu, F. Momen-Heravi, A. K. Rustgi and H. Nakagawa | 2022 | | Modeling Oral-Esophageal Squamous Cell Carcinoma in 3D Organoids | Jove-Journal of Visualized Experiments | | 10.3791/64676 |
| **142** | K. Fukai, N. Kojimahara, K. Hoshi, A. Toyota and M. Tatemichi | 2020 | | Combined effects of occupational exposure to hazardous operations and lifestyle-related factors on cancer incidence | Cancer Science | | 10.1111/cas.14663 |
| **143** | T. Fukuchi, K. Hirasawa, C. Sato, M. Makazu, H. Kaneko, R. Kobayashi, M. Nishio, R. Ikeda, A. Sawada, Y. Ozeki, M. Sugimori, Y. Inayama, Y. Tateishi and S. Maeda | 2021 | | Potential roles of gastroesophageal reflux in patients with superficial esophageal squamous cell carcinoma without major causative risk factors | Journal of Gastroenterology | | 10.1007/s00535-021-01815-x |
| **144** | S. Fukunaga, M. Mukasa, T. Nakane, D. Nakano, T. Tsutsumi, T. Chou, H. Tanaka, D. Hayashi, S. Minami, A. Ohuchi, T. Nagata, K. Takaki, H. Takaki, I. Miyajima, R. Nouno, T. Araki, T. Morita, T. Torimura, Y. Okabe and T. Kawaguchi | 2024 | | Impact of non-obese metabolic dysfunction-associated fatty liver disease on risk factors for the recurrence of esophageal squamous cell carcinoma treated with endoscopic submucosal dissection: A multicenter study | Hepatology Research | | 10.1111/hepr.13973 |
| **145** | M. S. Gallaway, S. J. Henley, C. B. Steele, B. Momin, C. C. Thomas, A. Jamal, K. F. Trivers, S. D. Singh and S. L. Stewart | 2018 | | Surveillance for Cancers Associated with Tobacco Use - United States, 2010-2014 | Mmwr Surveillance Summaries | | 10.15585/mmwr.ss6712a1 |
| **146** | Y. Gao, X. Y. Wu, Y. H. Li, Y. F. Li, Q. Y. Zhou, Q. Q. Wang, C. Y. Wei, D. L. Shi, C. Y. Xie and H. L. Pan | 2020 | | The Predictive Value of MLR for Radiation Pneumonia During Radiotherapy of Thoracic Tumor Patients | Cancer Management and Research | | 10.2147/cmar.S268964 |
| **147** | S. M. Gapstur, V. Bouvard, S. T. Nethan, J. L. Freudenheim, C. C. Abnet, D. R. English, J. Rehm, S. Balbo, P. Buykx, D. Crabb, D. I. Conway, F. Islami, D. W. Lachenmeier, K. A. McGlynn, M. Salaspuro, N. Sawada, M. B. Terry, T. Toporcov and B. Lauby-Secretan | 2023 | | The IARC Perspective on Alcohol Reduction or Cessation and Cancer Risk | New England Journal of Medicine | | 10.1056/NEJMsr2306723 |
| **148** | A. Garant, G. Spears, D. Routman, T. Whitaker, Z. X. Liao, W. Harmsen, A. Liu, M. Haddock, C. Hallemeier, S. Lin and K. Merrell | 2021 | | A Multi-Institutional Analysis of Radiation Dosimetric Predictors of Toxicity After Trimodality Therapy for Esophageal Cancer | Practical Radiation Oncology | | 10.1016/j.prro.2021.01.004 |
| **149** | M. Garau, C. Musetti, R. Alonso and E. Barrios | 2019 | | Trends in cancer incidence in Uruguay: 2002-2015 | Colombia Medica | | 10.25100/cm.v50i4.4212 |
| **150** | J. A. García-Lavandeira, A. Ruano-Ravina, M. Torres-Durán, I. Parente-Lamelas, M. Provencio, L. Varela-Lema, A. Fernández-Villar, M. Piñeiro, J. M. Barros-Dios and M. Pérez-Ríos | 2022 | | Fruits and Vegetables and Lung Cancer Risk in Never Smokers. A Multicentric and Pooled Case-Control Study | Nutrition and Cancer-an International Journal | | 10.1080/01635581.2021.1918732 |
| **151** | R. Gaspar, S. Rodrigues, M. Silva, P. Costa-Moreira, R. Morais, P. Andrade, H. Cardoso, A. Albuquerque, R. Liberal and G. Macedo | 2019 | | Predictive models of mortality and hospital readmission of patients with decompensated liver cirrhosis | Digestive and Liver Disease | | 10.1016/j.dld.2019.03.016 |
| **152** | V. Gatzinsky, G. Wennergren, L. Jönsson, L. Ekerljung, B. Houltz, S. Redfors, U. Sillén and P. Gustafsson | 2014 | | Impaired peripheral airway function in adults following repair of esophageal atresia | Journal of Pediatric Surgery | | 10.1016/j.jpedsurg.2013.12.027 |
| **153** | V. Gehlot, A. Mathur, K. Das, S. Mahant and R. Das | 2024 | | No Association between <i>Helicobacter pylori</i> and Gastroesophageal Reflux Disease: A Comprehensive Risk Factor Analysis in North Indian Patients | Biomedical and Biotechnology Research Journal | | 10.4103/bbrj.bbrj_157_24 |
| **154** | F. H. Geng, M. H. Liu, J. H. Chen, Y. L. Ge, S. X. Wei, F. Y. Li, C. S. Yang, J. W. Sun, L. J. Gou, J. Y. Zhang, S. K. Tang, Y. Wan, J. Y. Yang and J. Zhang | 2023 | | Clinical characteristics of second primary malignancies among first primary malignancy survivors: A single-center study, 2005-2020 | Oncology Letters | | 10.3892/ol.2022.13610 |
| **155** | S. K. George, B. Tlou, S. Ponnusamy and D. P. Naidoo | 2020 | | Does acid reflux precipitate ischaemia in subjects with acute coronary syndrome? | Cardiovascular Journal of Africa | | 10.5830/cvja-2019-048 |
| **156** | D. Georgescu, O. E. Ancusa, D. Azoulay, A. Lascu, I. Ionita, D. Calamar-Popovici, M. Ionita, C. I. Rosca, G. M. Breaza, D. Reisz and D. Lighezan | 2023 | | Portal Vein Thrombosis in Patients with Liver Cirrhosis: What Went Wrong? | International Journal of General Medicine | | 10.2147/ijgm.S413438 |
| **157** | E. Ghaderi, K. Hassanzadeh, K. Rahmani, G. Moradi, N. Esmailnasab, D. Roshani and A. Azadnia | 2020 | | Prevalence of self-medication and its associated factors: a case study of Kurdistan province | International Journal of Human Rights in Health Care | | 10.1108/ijhrh-09-2019-0075 |
| **158** | K. Ghanadi, K. Anbari, B. Khodadadi and M. S. Farahani | 2018 | | Correlation between Histopathological Findings and Endoscopy in Esophageal Cance: Results in Khorramabad, Iran, Western Iran | Bangladesh Journal of Medical Science | | 10.3329/bjms.v17i4.38325 |
| **159** | H. Ghaznavi, F. Allaveisi and F. Taghizadeh-Hesary | 2022 | | Baseline cardiac risk profile determines radiation-induced cardiac toxicity in patients with mid-lower esophageal cancer | Journal of Radiotherapy in Practice | | 10.1017/s146039692200036x |
| **160** | Y. T. Ghebre and G. Raghu | 2016 | | Idiopathic Pulmonary Fibrosis: Novel Concepts of Proton Pump Inhibitors as Antifibrotic Drugs | American Journal of Respiratory and Critical Care Medicine | | 10.1164/rccm.201512-2316PP |
| **161** | Z. Ghorbani, A. Hekmatdoost, H. E. Zinab, S. Farrokhzad, R. Rahimi, R. Malekzadeh and A. Pourshams | 2015 | | Dietary food groups intake and cooking methods associations with pancreatic cancer: A case-control study | Indian Journal of Gastroenterology | | 10.1007/s12664-015-0573-4 |
| **162** | J. Gilhodes, A. Belot, A. M. Bouvier, L. Remontet, P. Delafosse, K. Ligier and A. Rogel | 2015 | | Incidence of major smoking-related cancers: Trends among adults aged 20-44 in France from 1982 to 2012 | Cancer Epidemiology | | 10.1016/j.canep.2015.07.001 |
| **163** | F. Giusti, C. Martos, M. Bettio, R. N. Carvalho, M. Zorzi, S. Guzzinati and M. Rugge | 2024 | | Geographical and temporal differences in gastric and oesophageal cancer registration by subsite and morphology in Europe | Frontiers in Oncology | | 10.3389/fonc.2024.1250107 |
| **164** | A. Golozar, A. Etemadi, F. Kamangar, A. F. Malekshah, F. Islami, D. Nasrollahzadeh, B. Abedi-Ardekani, M. Khoshnia, A. Pourshams, S. Semnani, H. A. Marjani, R. Shakeri, M. Sotoudeh, P. Brennan, P. Taylor, P. Boffetta, C. Abnet, S. Dawsey and R. Malekzadeh | 2016 | | Food preparation methods, drinking water source, and esophageal squamous cell carcinoma in the high-risk area of Golestan, Northeast Iran | European Journal of Cancer Prevention | | 10.1097/cej.0000000000000156 |
| **165** | H. Goto, T. Oshikiri, T. Kato, R. Sawada, H. Harada, N. Urakawa, H. Hasegawa, S. Kanaji, K. Yamashita, T. Matsuda and Y. Kakeji | 2022 | | Short- and long-term outcomes of thoracoscopic esophagectomy in the prone position for esophageal squamous cell carcinoma in patients with obstructive ventilatory disorder: a propensity score-matched study | Surgical Endoscopy and Other Interventional Techniques | | 10.1007/s00464-022-09309-4 |
| **166** | H. Goto, T. Oshikiri, T. Kato, R. Sawada, H. Harada, N. Urakawa, H. Hasegawa, S. Kanaji, K. Yamashita, T. Matsuda and Y. Kakeji | 2023 | | The Influence of Preoperative Smoking Status on Postoperative Complications and Long-Term Outcome Following Thoracoscopic Esophagectomy in Prone Position for Esophageal Carcinoma | Annals of Surgical Oncology | | 10.1245/s10434-022-12898-y |
| **167** | L. M. Grande, F. A. M. Herbella, A. M. Bigatao, H. Abrao, J. R. Jardim and M. G. Patti | 2016 | | Pathophysiology of Gastroesophageal Reflux in Patients with Chronic Pulmonary Obstructive Disease Is Linked to an Increased Transdiaphragmatic Pressure Gradient and not to a Defective Esophagogastric Barrier | Journal of Gastrointestinal Surgery | | 10.1007/s11605-015-2955-4 |
| **168** | W. B. Grant | 2024 | | Cancer Incidence Rates in the US in 2016-2020 with Respect to Solar UVB Doses, Diabetes and Obesity Prevalence, Lung Cancer Incidence Rates, and Alcohol Consumption: An Ecological Study | Nutrients | | 10.3390/nu16101450 |
| **169** | A. Gressier, G. Gourier, J. P. Metges, J. D. Dewitte, B. Loddé and D. Lucas | 2022 | | Occupational Exposures and Esophageal Cancer: Prog Study | International Journal of Environmental Research and Public Health | | 10.3390/ijerph19169782 |
| **170** | X. Grevers, Y. B. Ruan, A. E. Poirier, S. D. Walter, P. J. Villeneuve, C. M. Friedenreich, D. R. Brenner, E. Franco, W. King, P. Demers, P. De, L. Smith, E. Holmes, D. O'Sullivan, K. Volesky, Z. El-Masri, R. Nuttall, M. El-Zein, T. Narain, P. Gogna and P. S. T. Com | 2019 | | Estimates of the current and future burden of cancer attributable to alcohol consumption in Canada | Preventive Medicine | | 10.1016/j.ypmed.2019.03.020 |
| **171** | L. Guadagnoli, H. Mashimo and W. K. Lo | 2023 | | Assessment of Post-traumatic Stress Disorder Among Objective Esophageal Motility and Reflux Phenotypes in Symptomatic Veterans | Journal of Clinical Psychology in Medical Settings | | 10.1007/s10880-022-09920-6 |
| **172** | O. Gunduz, C. Bakar, C. Simsek, A. Baba, A. Elci, H. Gurleyuk, M. Mutlu and A. Cakir | 2017 | | The Health Risk Associated with Chronic Diseases in Villages with High Arsenic Levels in Drinking Water Supplies | Exposure and Health | | 10.1007/s12403-016-0238-2 |
| **173** | W. Guo, H. L. Xiao, Z. Ma, H. J. Liu, Y. J. Wang, L. Y. Mei, X. H. Liu, Y. G. Jiang and R. W. Wang | 2014 | | Should Stage T2 Esophageal Squamous Cell Carcinoma Be Subclassified? | Annals of Surgical Oncology | | 10.1245/s10434-014-3636-3 |
| **174** | X. K. Guo, W. Q. Ke, X. Yang, X. Y. Zhao and M. Z. Li | 2023 | | Association of DLT versus SLT with postoperative pneumonia during esophagectomy in China: a retrospective comparison study | Bmc Anesthesiology | | 10.1186/s12871-023-02252-4 |
| **175** | Y. B. Guo, Y. W. Liu, H. J. Yang, N. T. Dai, F. Y. Zhou, H. Yang, W. Sun, J. Y. Kong, X. Yuan and S. G. Gao | 2021 | | RETRACTED: Associations of <i>Porphyromonas gingivalis</i> Infection and Low Beclin1 Expression With Clinicopathological Parameters and Survival of Esophageal Squamous Cell Carcinoma Patients (Retracted Article) | Pathology & Oncology Research | | 10.3389/pore.2021.1609976 |
| **176** | D. R. Gupta, Y. Liu, R. J. Jiang, S. Walid, K. Higgins, J. Landry, M. McDonald, F. F. Willingham, B. F. El-Rayes and N. F. Saba | 2019 | | Racial Disparities, Outcomes, and Surgical Utilization among Hispanics with Esophageal Cancer: A Surveillance, Epidemiology, and End Results Program Database Analysis | Oncology | | 10.1159/000499716 |
| **177** | Y. B. Hadi, A. A. Khan, S. F. Z. Naqvi and J. T. Kupec | 2020 | | Independent association of obstructive sleep apnea with Barrett's esophagus | Journal of Gastroenterology and Hepatology | | 10.1111/jgh.14779 |
| **178** | M. Hadji, H. Rashidian, M. Marzban, M. Gholipour, A. Naghibzadeh-Tahami, E. Mohebbi, E. Ebrahimi, B. Hosseini, A. A. Haghdoost, A. Rezaianzadeh, A. Rahimi-Movaghar, A. Moradi, M. S. Seyyedsalehi, R. Shirkoohi, H. Poustchi, S. Eghtesad, F. Najafi, R. Safari-Faramani, R. Alizadeh-Navaei, A. R. A. Moghadam, M. Bakhshi, A. Nejatizadeh, M. Mahmudi, S. Shahid-Sales, S. Ahmadi-Simab, O. Nabavian, P. Boffetta, E. Pukkala, E. Weiderpass, F. Kamangar and K. Zendehdel | 2021 | | The Iranian Study of Opium and Cancer (IROPICAN): Rationale, Design, and Initial Findings | Archives of Iranian Medicine | | 10.34172/aim.2021.27 |
| **179** | M. B. Hadley, M. Nalini, S. Adhikari, J. Szymonifka, A. Etemadi, F. Kamangar, M. Khoshnia, T. McChane, A. Pourshams, H. Poustchi, S. G. Sepanlou, C. Abnet, N. D. Freedman, P. Boffetta, R. Malekzadeh and R. Vedanthan | 2022 | | Spatial environmental factors predict cardiovascular and all-cause mortality: Results of the SPACE study | Plos One | | 10.1371/journal.pone.0269650 |
| **180** | H. Hagström, J. Höijer, H. U. Marschall, C. Williamson, M. A. Heneghan, R. H. Westbrook, J. F. Ludvigsson and O. Stephansson | 2018 | | Outcomes of Pregnancy in Mothers With Cirrhosis: A National Population-Based Cohort Study of 1.3 Million Pregnancies | Hepatology Communications | | 10.1002/hep4.1255 |
| **181** | M. B. Haider, B. Basida and J. Kaur | 2023 | | Major depressive disorders in patients with inflammatory bowel disease and rheumatoid arthritis | World Journal of Clinical Cases | | 10.12998/wjcc.v11.i4.764 |
| **182** | M. Hall, I. Bogdanovica and J. Britton | 2016 | | Research funding for addressing tobacco-related disease: an analysis of UK investment between 2008 and 2012 | Bmj Open | | 10.1136/bmjopen-2016-011609 |
| **183** | N. Hamade, G. Weng, M. Desai, V. T. Chandrasekar, C. Dasari, K. Kennedy and P. Sharma | 2021 | | Significant decline in the prevalence of Barrett's esophagus among patients with gastroesophageal reflux disease | Diseases of the Esophagus | | 10.1093/dote/doaa131 |
| **184** | J. M. Han, X. L. Guo, L. Zhao, H. Zhang, S. Q. Ma, Y. Li, D. L. Zhao, J. L. Wang and F. Z. Xue | 2023 | | Development and Validation of Esophageal Squamous Cell Carcinoma Risk Prediction Models Based on an Endoscopic Screening Program | Jama Network Open | | 10.1001/jamanetworkopen.2022.53148 |
| **185** | S. J. Han, S. J. Baik, Y. H. Yoon, J. H. Kim, H. S. Lee, S. Jeon and H. Park | 2023 | | Risk of Metabolic Syndrome and Fatty Liver Diseases in Gastric Cancer Survivors: A Propensity Score-Matched Analysis | Korean Journal of Gastroenterology | | 10.4166/kjg.2022.113 |
| **186** | Y. T. Han, X. Zhu, Y. Z. Hu, C. Q. Yu, Y. Guo, D. Hang, Y. J. Pang, P. Pei, H. X. Ma, D. J. Y. Sun, L. Yang, Y. P. Chen, H. D. Du, M. Yu, J. S. Chen, Z. M. Chen, D. Z. Huo, G. F. Jin, J. Lv, Z. B. Hu, H. B. Shen and L. M. Li | 2023 | | Electronic Health Record-Based Absolute Risk Prediction Model for Esophageal Cancer in the Chinese Population: Model Development and External Validation | Jmir Public Health and Surveillance | | 10.2196/43725 |
| **187** | H. A. Hanson, C. L. Leiser, B. O'Neil, C. Martin, S. Gupta, K. R. Smith, C. Dechet, W. T. Lowrance, M. J. Madsen and N. J. Camp | 2020 | | Harnessing Population Pedigree Data and Machine Learning Methods to Identify Patterns of Familial Bladder Cancer Risk | Cancer Epidemiology Biomarkers & Prevention | | 10.1158/1055-9965.Epi-19-0681 |
| **188** | T. Haque, A. Bin Nabhan, F. Akhter and H. N. Albagieh | 2023 | | The analysis of periodontal diseases and squamous cell esophageal cancer: A retrospective study | Saudi Dental Journal | | 10.1016/j.sdentj.2023.05.030 |
| **189** | S. Harari, M. Davi, A. Biffi, A. Caminati, A. Ghirardini, V. Lovato, C. Cricelli and F. Lapi | 2020 | | Epidemiology of idiopathic pulmonary fibrosis: a population-based study in primary care | Internal and Emergency Medicine | | 10.1007/s11739-019-02195-0 |
| **190** | A. Hata, M. Yanagawa, T. Miyata, Y. Hiraoka, M. Shirae, K. Ninomiya, S. Doi, K. Yamagata, Y. Yoshida, N. Kikuchi, R. Ogawa, H. Hatabu and N. Tomiyama | 2024 | | Association between interstitial lung abnormality and mortality in patients with esophageal cancer | Japanese Journal of Radiology | | 10.1007/s11604-024-01563-x |
| **191** | E. Hayano, Y. Gon, Y. Kimura, L. Zha, T. Morishima, Y. Ohno, H. Mochizuki, T. Sobue and I. Miyashiro | 2024 | | Risk of Parkinson's disease-related death in cancer survivors: A population-based study in Japan | Parkinsonism & Related Disorders | | 10.1016/j.parkreldis.2023.105966 |
| **192** | Y. Hayashi, H. Iijima, F. Isohashi, Y. Tsujii, T. Fujinaga, K. Nagai, S. Yoshii, A. Sakatani, S. Hiyama, S. Shinzaki, T. Makino, M. Yamasaki, K. Ogawa, Y. Doki and T. Takehara | 2019 | | The heart's exposure to radiation increases the risk of cardiac toxicity after chemoradiotherapy for superficial esophageal cancer: a retrospective cohort study | Bmc Cancer | | 10.1186/s12885-019-5421-y |
| **193** | W. D. Hazelton, K. Curtius, J. M. Inadomi, T. L. Vaughan, R. Meza, J. H. Rubenstein, C. Hur and E. G. Luebeck | 2015 | | The Role of Gastroesophageal Reflux and Other Factors during Progression to Esophageal Adenocarcinoma | Cancer Epidemiology Biomarkers & Prevention | | 10.1158/1055-9965.Epi-15-0323-t |
| **194** | H. H. He, J. H. Fu, Z. X. Hao, H. F. Wu, Q. Zhong, F. Wang, H. H. Liu, X. S. Gu, B. Wang, H. D. Huang, Z. Y. Li and J. X. He | 2020 | | Impact of metformin on survival outcome of esophageal squamous cell carcinomas patients undergoing surgical resection: a multicenter retrospective study | Journal of Thoracic Disease | | 10.21037/jtd.2019.12.98 |
| **195** | H. Q. He, N. Z. Chen, Y. Hou, Z. Wang, Y. Zhang, G. J. Zhang and J. K. Fu | 2020 | | Trends in the incidence and survival of patients with esophageal cancer: A SEER database analysis | Thoracic Cancer | | 10.1111/1759-7714.13311 |
| **196** | P. P. Hekking, M. Amelink, R. R. Wener, M. L. Bouvy and E. H. Bel | 2018 | | Comorbidities in Difficult-to-Control Asthma | Journal of Allergy and Clinical Immunology-in Practice | | 10.1016/j.jaip.2017.06.008 |
| **197** | K. Hemminki, K. Sundquist, J. Sundquist, A. Försti, V. Liska, A. Hemminki and X. J. Li | 2022 | | Familial Risks for Liver, Gallbladder and Bile Duct Cancers and for Their Risk Factors in Sweden, a Low-Incidence Country | Cancers | | 10.3390/cancers14081938 |
| **198** | K. Henau, E. Van Eycken, G. Silversmit and E. Pukkala | 2015 | | Regional variation in incidence for smoking and alcohol related cancers in Belgium | Cancer Epidemiology | | 10.1016/j.canep.2014.10.009 |
| **199** | B. Y. Hernandez, R. A. Bordallo, M. D. Green and R. L. Haddock | 2017 | | Cancer in Guam and Hawaii: A comparison of two US Island populations | Cancer Epidemiology | | 10.1016/j.canep.2017.08.005 |
| **200** | T. Hisamatsu, A. Kadota, T. Hayakawa, Y. Kita, A. Harada, Y. Okami, K. Kondo, T. Ohkubo, T. Okamura, A. Okayama, H. Ueshima, K. Miura and N. D. R. Grp | 2024 | | High blood pressure and colorectal cancer mortality in a 29-year follow-up of the Japanese general population: NIPPON DATA80 | Hypertension Research | | 10.1038/s41440-023-01497-3 |
| **201** | K. L. Hoffman, D. S. Hutchinson, J. Fowler, D. P. Smith, N. J. Ajami, H. Zhao, P. Scheet, W. H. Chow, J. F. Petrosino and C. R. Daniel | 2018 | | Oral microbiota reveals signs of acculturation in Mexican American women | Plos One | | 10.1371/journal.pone.0194100 |
| **202** | S. A. Hojati, S. Kokabpeyk, S. Yaghoubi, F. Joukar, M. Asgharnezhad and F. Mansour-Ghanaei | 2021 | | <i>Helicobacter pylori</i> infection in Iran: demographic, endoscopic and pathological factors | Bmc Gastroenterology | | 10.1186/s12876-021-01931-1 |
| **203** | K. J. Holzer, M. G. Vaughn, T. M. Loux, M. A. Mancini, N. E. Fearn and C. L. Wallace | 2022 | | Prevalence and correlates of antisocial personality disorder in older adults | Aging & Mental Health | | 10.1080/13607863.2020.1839867 |
| **204** | Z. N. Hong, K. Weng, Z. Chen, K. M. Peng and M. Q. Kang | 2022 | | Difference between "Lung Age" and Real Age as a Novel Predictor of Postoperative Complications, Long-Term Survival for Patients with Esophageal Cancer after Minimally Invasive Esophagectomy | Frontiers in Surgery | | 10.3389/fsurg.2022.794553 |
| **205** | T. Horinouchi, N. Yoshida, S. Shiraishi, Y. Hara, C. Matsumoto, T. Toihata, K. Kosumi, K. Harada, K. Eto, K. Ogawa, H. Sawayama, M. Iwatsuki, Y. Baba, Y. Miyamoto and H. Baba | 2024 | | Relationship between the severity of emphysematous change in the lung and morbidity after esophagectomy for esophageal cancer: A retrospective study on a novel strategy for risk prediction | Thoracic Cancer | | 10.1111/1759-7714.15146 |
| **206** | Y. Horiuchi, J. Fujisaki, N. Ishizuka, M. Omae, A. Ishiyama, T. Yoshio, T. Hirasawa, Y. Yamamoto, M. Nagahama, H. Takahashi and T. Tsuchida | 2017 | | Study on Clinical Factors Involved in <i>Helicobacter pylori</i>-Uninfected, Undifferentiated-Type Early Gastric Cancer | Digestion | | 10.1159/000481817 |
| **207** | C. C. Horn | 2014 | | The Medical Implications of Gastrointestinal Vagal Afferent Pathways in Nausea and Vomiting | Current Pharmaceutical Design | | 10.2174/13816128113199990568 |
| **208** | R. Howard, K. Singh and M. Englesbe | 2021 | | Prevalence and Trends in Smoking Among Surgical Patients in Michigan, 2012-2019 | Jama Network Open | | 10.1001/jamanetworkopen.2021.0553 |
| **209** | K. Y. Hsu, Y. F. Tsai, C. C. Huang, W. L. Yeh, K. P. Chang, C. C. Ling, C. Y. Chen and H. L. Lee | 2018 | | Tobacco-Smoking, Alcohol-Drinking, and Betel-Quid-Chewing Behaviors: Development and Use of a Web-Based Survey System | Jmir Mhealth and Uhealth | | 10.2196/mhealth.9783 |
| **210** | Q. Huang, Y. Q. Cheng, E. Lew, J. Shi, D. Wiener and H. C. Weber | 2023 | | Patients with esophageal adenocarcinoma showed better prognosis than those with adenocarcinoma of the gastroesophageal junction | Journal of Digestive Diseases | | 10.1111/1751-2980.13167 |
| **211** | Q. Y. Huang, K. J. Luo, H. Yang, J. Wen, S. S. Zhang, J. H. Li, A. E. Bella, Q. W. Liu, F. Yang, Y. Z. Zheng, R. G. Hu, J. Y. Chen and J. H. Fu | 2014 | | Impact of alcohol consumption on survival in patients with esophageal carcinoma: A large cohort with long-term follow-up | Cancer Science | | 10.1111/cas.12552 |
| **212** | Y. C. Huang, M. C. Lee, S. Y. Huang, C. M. Chou, H. W. Yang and I. C. Chen | 2024 | | Polygenic Risk Score in Predicting Esophageal, Oropharyngeal, and Hypopharynx Cancer Risk among Taiwanese Population | Cancers | | 10.3390/cancers16040707 |
| **213** | M. P. Hunter and N. J. Crowther | 2019 | | The prevalence of gastroesophageal reflux disease in an adult, South African black population, and the association with obesity | Minerva Gastroenterologica E Dietologica | | 10.23736/s1121-421x.18.02495-9 |
| **214** | T. Inoue, S. Ito, M. Ando, M. Nagaya, H. Aso, Y. Mizuno, K. Hattori, H. Nakajima, Y. Nishida, Y. Niwa, Y. Kodera, M. Koike and Y. Hasegawa | 2016 | | Changes in exercise capacity, muscle strength, and health-related quality of life in esophageal cancer patients undergoing esophagectomy | Bmc Sports Science Medicine and Rehabilitation | | 10.1186/s13102-016-0060-y |
| **215** | Y. Iwaya, Y. Shimamura, K. Goda, E. R. de Santiago, J. G. Coneys, J. D. Mosko, G. Kandel, P. Kortan, G. May, N. Marcon and C. Teshima | 2019 | | Clinical characteristics of young patients with early Barrett's neoplasia | World Journal of Gastroenterology | | 10.3748/wjg.v25.i24.3069 |
| **216** | Y. Iwaya, Y. Shimamura, J. D. Mosko, G. Kandel, P. P. Kortan, G. R. May, N. E. Marcon and C. W. Teshima | 2019 | | Clinical characteristics may distinguish patients with esophageal adenocarcinoma arising from long- versus short-segment Barrett's esophagus | Digestive and Liver Disease | | 10.1016/j.dld.209.05.000 |
| **217** | N. Izadi, K. Etemad, P. Mohseni, A. Khosravi and M. E. Akbari | 2022 | | Mortality Rates and Years of Life Lost Due to Cancer in Iran: Analysis of Data from the National Death Registration System, 2016 | International Journal of Cancer Management | | 10.5812/ijcm-123633 |
| **218** | P. Jaehn, B. Holleczek, H. Becher and V. Winkler | 2016 | | Histologic types of gastric cancer among migrants from the former Soviet Union and the general population in Germany: what kind of prevention do we need? | European Journal of Gastroenterology & Hepatology | | 10.1097/meg.0000000000000645 |
| **219** | S. Javali, M. Madan, M. L. Harendrakumar and M. S. Mahesh | 2015 | | Role of endoscopy in evaluating upper gastrointestinal tract lesions in rural population | Journal of Digestive Endoscopy | | 10.4103/0976-5042.159238 |
| **220** | J. W. Jeon, S. J. Kim, J. Y. Jang, S. M. Kim, C. H. Lim, J. M. Park, S. J. Hong, C. G. Kim, S. W. Jeon, S. H. Lee, J. K. Sung and G. H. Baik | 2021 | | Clinical Outcomes of Endoscopic Resection for Low-Grade Dysplasia and High-Grade Dysplasia on Gastric Pretreatment Biopsy: Korea ESD Study Group | Gut and Liver | | 10.5009/gnl19275 |
| **221** | Y. J. Jeon, K. Y. D. Han, S. W. Lee, J. E. Lee, J. Park, I. Y. Cho, J. H. Cho and D. W. Shin | 2024 | | Metabolic dysfunction-associated steatotic liver disease and risk of esophageal cancer in patients with diabetes mellitus: a nationwide cohort study | Diseases of the Esophagus | | 10.1093/dote/doae029 |
| **222** | S. C. Jia, H. Li, H. M. Zeng, R. S. Zheng, J. Li, J. F. Shi, Z. X. Yang, M. M. Cao and W. Q. Chen | 2019 | | Association of cancer prevention awareness with esophageal cancer screening participation rates: Results from a population-based cancer screening program in rural China | Chinese Journal of Cancer Research | | 10.21147/j.issn.1000-9604.2019.04.04 |
| **223** | X. X. Jia, C. Sheng, X. X. Han, M. Y. Li and K. J. Wang | 2024 | | Global burden of stomach cancer attributable to smoking from 1990 to 2019 and predictions to 2044 | Public Health | | 10.1016/j.puhe.2023.11.019 |
| **224** | Q. W. Jiang, Y. Y. Shu, Z. Y. Jiang, Y. Q. Zhang, S. W. Pan, W. H. Jiang, J. X. Liang, X. D. Cheng and Z. Y. Xu | 2024 | | Burdens of stomach and esophageal cancer from 1990 to 2019 and projection to 2030 in China: Findings from the 2019 Global Burden of Disease Study | Journal of Global Health | | 10.7189/jogh.14.04025 |
| **225** | S. J. Jiang, A. C. Diaconescu, D. P. McEwen, L. N. McEwen, A. C. Chang, J. L. Lin, R. M. Reddy, W. R. Lynch, S. Bonner and K. H. Lagisetty | 2023 | | Factors affecting timing of surgery following neoadjuvant chemoradiation for esophageal cancer | Heliyon | | 10.1016/j.heliyon.2023.e23212 |
| **226** | Y. Jiang, Y. C. Lin, Y. K. Wen, W. H. Fu, R. Wang, J. X. He, J. R. Zhang, Z. F. Wang, F. Ge, Z. Y. Huo, R. C. Wang, H. X. Peng, X. R. Wu, J. X. He and S. B. Li | 2023 | | Global trends in the burden of esophageal cancer, 1990-2019 Results from the Global Burden of Disease Study 2019 | Journal of Thoracic Disease | | 10.21037/jtd-22-856 |
| **227** | C. A. Jiménez-Ruiz, M. García, M. A. Martínez, J. Sellarés, M. A. Jiménez-Fuentes, L. Lázaroo, E. Rodríguez, C. Rodriguez, O. Armengo, E. Abad, T. Peña, A. Domenech and J. A. Riescos | 2017 | | Varenicline in smokers with severe or very severe COPD after 24 weeks of treatment. A descriptive analysis: VALUE study | Monaldi Archives for Chest Disease | | 10.4081/monaldi.2017.874 |
| **228** | Z. Y. Jin, G. Wallar, J. Y. Zhou, J. Yang, R. Q. Han, P. H. Wang, A. M. Liu, X. P. Gu, X. F. Zhang, X. S. Wang, M. Su, X. Hu, Z. Sun, G. Li, L. N. Mu, Q. Y. Lu, X. Liu, L. M. Li, N. He, M. Wu, J. K. Zhao and Z. F. Zhang | 2019 | | Consumption of garlic and its interactions with tobacco smoking and alcohol drinking on esophageal cancer in a Chinese population | European Journal of Cancer Prevention | | 10.1097/cej.0000000000000456 |
| **229** | O. M. P. Jolobe | 2021 | | Differential diagnosis of the association of gastrointestinal symptoms and ST segment elevation, in the absence of chest pain | American Journal of Emergency Medicine | | 10.1016/j.ajem.2021.05.067 |
| **230** | S. Kageyama, T. Takeshita, K. Takeuchi, M. Asakawa, R. Matsumi, M. Furuta, Y. Shibata, K. Nagai, M. Ikebe, M. Morita, M. Masuda, Y. Toh, Y. Kiyohara, T. Ninomiya and Y. Yamashita | 2019 | | Characteristics of the Salivary Microbiota in Patients With Various Digestive Tract Cancers | Frontiers in Microbiology | | 10.3389/fmicb.2019.01780 |
| **231** | Y. Kamiide, N. Inomata, M. Furuya and T. Yada | 2015 | | Ghrelin ameliorates catabolic conditions and respiratory dysfunction in a chronic obstructive pulmonary disease model of chronic cigarette smoke-exposed rats | European Journal of Pharmacology | | 10.1016/j.ejphar.2015.02.049 |
| **232** | K. J. Kamp and M. Stommel | 2021 | | Health-Related Quality of Life Among Patients With Inflammatory Bowel Disease A Case Control Study | Gastroenterology Nursing | | 10.1097/sga.0000000000000491 |
| **233** | K. Kanamori, D. Kurita, Y. Hirano, K. Ishiyama, J. Oguma, K. Masutomi and H. Daiko | 2022 | | Does synchronous early head and neck cancer with esophageal cancer need treatment after preoperative chemotherapy? | General Thoracic and Cardiovascular Surgery | | 10.1007/s11748-021-01744-9 |
| **234** | H. Kaneko, Y. Suzuki, K. Ueno, A. Okada, K. Fujiu, S. Matsuoka, N. Michihata, T. Jo, N. Takeda, H. Morita, K. Kamiya, K. Node, H. Yasunaga and I. Komuro | 2022 | | Association of Life's Simple 7 with incident cardiovascular disease in 53 974 patients with cancer | European Journal of Preventive Cardiology | | 10.1093/eurjpc/zwac195 |
| **235** | Y. Kanie, A. Okamura, K. Tomizuka, T. Uchiyama, J. Kanamori, Y. Imamura, T. Ebata and M. Watanabe | 2023 | | Quantitative Evaluation of Periodontitis for Predicting the Occurrence of Postoperative Pneumonia After Oncologic Esophagectomy | Annals of Surgical Oncology | | 10.1245/s10434-023-14030-0 |
| **236** | C. H. Kao, L. M. Sun, Y. S. Chen, C. L. Lin, J. A. Liang, C. H. Kao and M. W. Weng | 2016 | | Risk of Nongenitourinary Cancers in Patients With Spinal Cord Injury A Population-based Cohort Study | Medicine | | 10.1097/md.0000000000002462 |
| **237** | A. Karbasi, R. Aliannejad, M. Ghanei, M. N. Sanamy, F. Alaeddini and A. A. Harandi | 2015 | | Frequency distribution of gastro esophageal reflux disease in inhalation injury: A historical cohort study | Journal of Research in Medical Sciences | | 10.4103/1735-1995.166199 |
| **238** | A. Karlsson, A. Ellonen, H. Irjala, V. Väliaho, K. Mattila, L. Nissi, E. Kytö, S. Kurki, R. Ristamäki, P. Vihinen, T. Laitinen, A. Ålgars, S. Jyrkkiö, H. Minn and E. Heervä | 2021 | | Impact of deep learning-determined smoking status on mortality of cancer patients: never too late to quit | Esmo Open | | 10.1016/j.esmoop.2021.100175 |
| **239** | E. Kasap, A. Ayer, H. Bozoglan, C. Ozen, I. Eslek and H. Yüceyar | 2015 | | Schizophrenia and gastroesophageal reflux symptoms | Indian Journal of Psychiatry | | 10.4103/0019-5545.148529 |
| **240** | S. Katuwal, P. Jousilahti and E. Pukkala | 2021 | | Causes of death among women with breast cancer: A follow-up study of 50 481 women with breast cancer in Finland | International Journal of Cancer | | 10.1002/ijc.33607 |
| **241** | J. H. Kauppila, K. Wahlin, P. Lagergren and J. Lagergren | 2019 | | Sex differences in the prognosis after surgery for esophageal squamous cell carcinoma and adenocarcinoma | International Journal of Cancer | | 10.1002/ijc.31840 |
| **242** | H. G. Kay, B. Campbell, J. N. Gallant, C. Carlile, P. Wright, B. Stephens and S. L. Rohde | 2021 | | Delayed Upper Aerodigestive Tract Perforation from Anterior Cervical Spine Hardware: Treatment and Swallowing Outcomes | Dysphagia | | 10.1007/s00455-021-10361-w |
| **243** | J. Z. Ke, T. Lin, X. L. Liu, K. Wu, X. N. Ruan, Y. B. Ding, W. B. Liu, H. Qiu, X. J. Tan, X. N. Wang, X. Chen, Z. T. Li and G. W. Cao | 2021 | | Glucose Intolerance and Cancer Risk: A Community-Based Prospective Cohort Study in Shanghai, China | Frontiers in Oncology | | 10.3389/fonc.2021.726672 |
| **244** | A. Khaledifar, M. Hashemzadeh, K. Solati, H. Poustchi, V. Bollati, A. Ahmadi, S. Kheiri, K. G. Samani, M. Banitalebi, M. Sedehi and R. Malekzadeh | 2018 | | The protocol of a population-based prospective cohort study in southwest of Iran to analyze common non-communicable diseases: Shahrekord cohort study | Bmc Public Health | | 10.1186/s12889-018-5364-2 |
| **245** | H. Khalid, A. Zahid and M. W. Zahid | 2018 | | A CROSS SECTIONAL STUDY; GERD AS A RISK FACTOR OF ACUTE EXACERBATION OF COPD | Indo American Journal of Pharmaceutical Sciences | | 10.5281/zenodo.1245715 |
| **246** | E. H. Kim, S. Nam, C. H. Park, Y. Kim, M. Lee, J. B. Ahn, S. J. Shin, Y. R. Park, H. I. Jung, B. I. Kim, I. Jung and H. S. Kim | 2022 | | Periodontal disease and cancer risk: A nationwide population-based cohort study | Frontiers in Oncology | | 10.3389/fonc.2022.901098 |
| **247** | H. J. Kim, N. Kim, H. Y. Kim, H. S. Lee, H. Yoon, C. M. Shin, Y. S. Park, D. J. Park, H. H. Kim, K. H. Lee, Y. H. Kim, H. M. Kim and D. H. Lee | 2015 | | Relationship between body mass index and the risk of early gastric cancer and dysplasia regardless of <i>Helicobacter pylori</i> infection | Gastric Cancer | | 10.1007/s10120-014-0429-0 |
| **248** | M. Kim, K. S. Choi, M. Suh, J. K. Jun, K. W. Chuck and B. Park | 2018 | | Risky Lifestyle Behaviors among Gastric Cancer Survivors Compared with Matched Non-cancer Controls: Results from Baseline Result of Community Based Cohort Study | Cancer Research and Treatment | | 10.4143/crt.2017.129 |
| **249** | Y. Kim, S. Ganocy and R. Fass | 2020 | | Proton-pump inhibitor use and the development of new ischemic heart disease in non-cardiac chest pain patients | Neurogastroenterology and Motility | | 10.1111/nmo.13844 |
| **250** | Y. Kim, S. Sharp, S. Hwang and S. H. Jee | 2019 | | Exercise and incidence of myocardial infarction, stroke, hypertension, type 2 diabetes and site-specific cancers: prospective cohort study of 257 854 adults in South Korea | Bmj Open | | 10.1136/bmjopen-2018-025590 |
| **251** | Y. J. Kim, W. C. Chung, I. H. Cho, J. Kim and S. Kim | 2019 | | Prognostic effect of different etiologies in patients with gastric cardia cancer | Medicine | | 10.1097/md.0000000000018397 |
| **252** | A. Kimura, N. Morinaga, W. Wada, K. Ogata, T. Okuyama, H. Kato, M. Sohda, K. Shirabe and H. Saeki | 2022 | | Patient with gastric cancer who underwent distal gastrectomy after treatment of COVID-19 infection diagnosed by preoperative PCR screening | Surgical Case Reports | | 10.1186/s40792-022-01367-z |
| **253** | Y. Kishida, T. Tsushima, M. Endo, S. Hamauchi, A. Todaka, T. Yokota, N. Machida, K. Yamazaki, A. Fukutomi, Y. Onozawa and H. Yasui | 2018 | | Risk Analysis of Pneumonitis in Taxane Therapy After Chemoradiotherapy for Patients With Metastatic or Recurrent Esophageal Cancer | American Journal of Clinical Oncology-Cancer Clinical Trials | | 10.1097/coc.0000000000000232 |
| **254** | H. K. Koo, I. Jeong, S. W. Lee, J. Park, J. H. Kim, S. Y. Park, H. Y. Park, C. K. Rhee, Y. H. Kim, J. Y. Jung, S. K. Kim, Y. H. Kim, E. Y. Choi, J. Y. Moon, J. W. Shin, J. W. Kim, K. H. Min, S. W. Kim, K. H. Yoo, J. H. Kim, S. H. Jang, H. K. Yoon, H. J. Kim, K. S. Jung and D. K. Kim | 2016 | | Prevalence of chronic cough and possible causes in the general population based on the Korean National Health and Nutrition Examination Survey | Medicine | | 10.1097/md.0000000000004595 |
| **255** | H. O. Koskela, A. M. Lätti and M. K. Purokivi | 2017 | | Long-term prognosis of chronic cough: a prospective, observational cohort study | Bmc Pulmonary Medicine | | 10.1186/s12890-017-0496-1 |
| **256** | I. O. Kostitska, B. M. Mankovsky, A. M. Urbanovych, O. Y. Zhurakivska, O. V. Tymoshchuk and I. O. Basiuha | 2019 | | Risk factors and early detection of gastroparesis in patients with type 2 diabetes mellitus | Regulatory Mechanisms in Biosystems | | 10.15421/021909 |
| **257** | K. Kou, P. D. Baade, X. L. Guo, M. Gatton, S. Cramb, Z. L. Lu, Z. T. Fu, J. Chu, A. Q. Xu and J. D. Sun | 2019 | | Area socioeconomic status is independently associated with esophageal cancer mortality in Shandong, China | Scientific Reports | | 10.1038/s41598-019-42774-x |
| **258** | O. V. Krapivnaia | 2023 | | Comparison of the effectiveness of rabeprazole original and generic products in the monotherapy of gastroesophageal reflux disease | Terapevticheskii Arkhiv | | 10.26442/00403660.2023.08.202346 |
| **259** | T. B. Kratzer, A. Jemal, K. D. Miller, S. Nash, C. Wiggins, D. Redwood, R. Smith and R. L. Siegel | 2023 | | Cancer statistics for American Indian and Alaska Native individuals, 2022: Including increasing disparities in early onset colorectal cancer | Ca-a Cancer Journal for Clinicians | | 10.3322/caac.21757 |
| **260** | L. Kravchenko, I. Borisyuk, N. Fizor, L. Unhurian, E. Zolotukhina and O. Goncharenko | 2020 | | Local Use of Apisan Gel, A New Oral Care Product in the Treatment of Experimental Periodontitis Against the Background of Hyperacid Gastritis and Intoxication with Tobacco Smoke | Turkish Journal of Pharmaceutical Sciences | | 10.4274/tjps.galenos.2018.53386 |
| **261** | M. Kreuzer, V. Deffner, M. Schnelzer and N. Fenske | 2021 | | Mortality in Underground Miners in a Former Uranium Ore Mine Results of a Cohort Study Among Former Employees of Wismut AG in Saxony and Thuringia | Deutsches Arzteblatt International | | 10.3238/arztebl.m2021.0001 |
| **262** | K. Kridin, S. Zelber-Sagi, D. Comaneshter and A. D. Cohen | 2018 | | Coexistent Solid Malignancies in Pemphigus A Population-Based Study | Jama Dermatology | | 10.1001/jamadermatol.2017.6334 |
| **263** | A. Krishnamoorthy and K. Kuberan | 2021 | | Clinico Pathological Profiles of Patients with Proximal Versus Distal Gastric Adenocarcinoma | Journal of Research in Medical and Dental Science | |  |
| **264** | C. Kromer, J. Xu, Q. T. Ostrom, H. Gittleman, C. Kruchko, R. Sawaya and J. S. Barnholtz-Sloan | 2017 | | Estimating the annual frequency of synchronous brain metastasis in the United States 2010-2013: a population-based study | Journal of Neuro-Oncology | | 10.1007/s11060-017-2516-7 |
| **265** | H. S. Küçükerdem, M. Arslan, E. M. Koç and H. Can | 2017 | | Retrospective Evaluation of Family Medicine Outpatient Clinic Profile at a Tertiary Hospital in Izmir | Journal of Academic Research in Medicine-Jarem | | 10.5152/jarem.2017.1165 |
| **266** | S. Kumar, D. S. Goldberg and D. E. Kaplan | 2022 | | Ranitidine Use and Gastric Cancer Among Persons with <i>Helicobacter pylori</i> | Digestive Diseases and Sciences | | 10.1007/s10620-021-06972-w |
| **267** | B. Kumaraswamy | 2015 | | ACUTE PERFORATED PEPTIC ULCER: A CLINICAL, DIAGNOSTIC AND MANAGEMENT ANALYSIS IN A TERTIARY HOSPITAL OF TELANGANA | Journal of Evolution of Medical and Dental Sciences-Jemds | | 10.14260/jemds/2015/2417 |
| **268** | L. S. Kuze, J. P. De Carli, J. S. Presotto, K. Collares and A. Della Bona | 2023 | | Genotoxicity in the oral cells of older people from a Brazilian rural area: a population-based study | Brazilian Oral Research | | 10.1590/1807-3107bor-2023.vol37.0067 |
| **269** | M. Latorre, A. M. da Silva, D. Chinzon, J. N. Eisig and T. R. P. Dias-Bastos | 2014 | | Epidemiology of upper gastrointestinal symptoms in Brazil (EpiGastro): A population-based study according to sex and age group | World Journal of Gastroenterology | | 10.3748/wjg.v20.i46.17388 |
| **270** | H. W. Lee, D. Huang, W. K. Shin, K. de la Torre, M. Song, A. Shin, J. K. Lee and D. Kang | 2022 | | Frequent low dose alcohol intake increases gastric cancer risk: the Health Examinees-Gem (HEXA-G) study | Cancer Biology & Medicine | | 10.20892/j.issn.2095-3941.2021.0642 |
| **271** | K. Lee, J. S. Lee, J. Kim, H. Lee, Y. Chang, H. G. Woo, J. W. Kim and T. J. Song | 2020 | | Oral health and gastrointestinal cancer: A nationwide cohort study | Journal of Clinical Periodontology | | 10.1111/jcpe.13304 |
| **272** | W. Lee, J. Kim, S. S. Lim, Y. Kim, Y. S. Ahn and J. H. Yoon | 2020 | | External Airborne-agent Exposure Increase Risk of Digestive Tract Cancer | Scientific Reports | | 10.1038/s41598-020-65312-6 |
| **273** | Y. J. Lee, M. Redd, L. Bayman, N. Frederickson, J. Valestin and R. Schey | 2015 | | Comparison of clinical features in patients with eosinophilic esophagitis living in an urban and rural environment | Diseases of the Esophagus | | 10.1111/dote.12164 |
| **274** | H. Y. Li, X. Z. Yang, A. Q. Zhang, G. Y. Liang, Y. Sun and J. Zhang | 2024 | | Age-period-cohort analysis of incidence, mortality and disability-adjusted life years of esophageal cancer in global, regional and national regions from 1990 to 2019 | Bmc Public Health | | 10.1186/s12889-024-17706-8 |
| **275** | J. X. Li, J. Chen and W. F. Tang | 2019 | | The consensus of integrative diagnosis and treatment of acute pancreatitis-2017 | Journal of Evidence Based Medicine | | 10.1111/jebm.12342 |
| **276** | P. Li, J. Jing, W. J. Liu, J. Z. Wang, X. Qi and G. J. Zhang | 2023 | | Spatiotemporal Patterns of Esophageal Cancer Burden Attributable to Behavioral, Metabolic, and Dietary Risk Factors From 1990 to 2019: Longitudinal Observational Study | Jmir Public Health and Surveillance | | 10.2196/46051 |
| **277** | R. Q. Li, J. Y. Sun, T. Wang, L. H. Huang, S. W. Wang, P. L. Sun and C. H. Yu | 2022 | | Comparison of Secular Trends in Esophageal Cancer Mortality in China and Japan during 1990-2019: An Age-Period-Cohort Analysis | International Journal of Environmental Research and Public Health | | 10.3390/ijerph191610302 |
| **278** | X. X. Li, L. Yu, M. Fu, J. N. Yang and H. Y. Tan | 2024 | | Perioperative Risk Factors for Postoperative Pulmonary Complications After Minimally Invasive Esophagectomy | International Journal of General Medicine | | 10.2147/ijgm.S449530 |
| **279** | X. Y. Li, C. Q. Yu, Y. Guo, Z. Bian, Z. W. Shen, L. Yang, Y. P. Chen, Y. Y. Wei, H. Zhang, Z. Qiu, J. S. Chen, F. Chen, Z. M. Chen, J. Lv, L. M. Li and C. China Kadoorie Biobank | 2019 | | Association between tea consumption and risk of cancer: a prospective cohort study of 0.5 million Chinese adults | European Journal of Epidemiology | | 10.1007/s10654-019-00530-5 |
| **280** | Y. Li, A. J. Byun, J. K. Choe, S. H. Lu, D. Restle, T. Eguchi, K. S. Tan, J. Saini, J. Huang, G. Rocco, D. R. Jones, W. D. Travis and P. S. Adusumilli | 2023 | | Micropapillary and Solid Histologic Patterns in N1 and N2 Lymph Node Metastases Are Independent Factors of Poor Prognosis in Patients With Stages II to III Lung Adenocarcinoma | Journal of Thoracic Oncology | | 10.1016/j.jtho.2023.01.002 |
| **281** | K. M. Liao, C. H. Yu, Y. C. Wu, J. J. Wang, F. W. Liang and C. H. Ho | 2024 | | Risk of Atrial Fibrillation in Patients with Different Cancer Types in Taiwan | Life-Basel | | 10.3390/life14050621 |
| **282** | C. Y. Lin, H. Y. Fang, C. L. Feng, C. C. Li and C. R. Chien | 2016 | | Cost-effectiveness of neoadjuvant concurrent chemoradiotherapy versus esophagectomy for locally advanced esophageal squamous cell carcinoma: A population-based matched case-control study | Thoracic Cancer | | 10.1111/1759-7714.12326 |
| **283** | L. F. Lin, Z. Y. Li, L. Yan, Y. L. Liu, H. J. Yang and H. Li | 2021 | | Global, regional, and national cancer incidence and death for 29 cancer groups in 2019 and trends analysis of the global cancer burden, 1990-2019 | Journal of Hematology & Oncology | | 10.1186/s13045-021-01213-z |
| **284** | X. Q. Lin, L. Peng, X. J. Xu, Y. R. Chen, Y. L. Zhang and X. Huo | 2018 | | Connecting gastrointestinal cancer risk to cadmium and lead exposure in the Chaoshan population of Southeast China | Environmental Science and Pollution Research | | 10.1007/s11356-018-1914-5 |
| **285** | S. Lipka, A. Kumar and J. E. Richter | 2016 | | Impact of Diagnostic Delay and Other Risk Factors on Eosinophilic Esophagitis Phenotype and Esophageal Diameter | Journal of Clinical Gastroenterology | | 10.1097/mcg.0000000000000297 |
| **286** | B. D. Liu, S. C. Udemba, S. Saleh, H. Hill, G. Q. Song and R. Fass | 2023 | | Raloxifene increases the risk of gastroesophageal reflux disease, Barrett's esophagus, and esophageal stricture in postmenopausal women with osteoporosis | Neurogastroenterology and Motility | | 10.1111/nmo.14689 |
| **287** | G. Y. Liu, L. Peng, B. Liu, K. N. Wang and Y. T. Han | 2019 | | Analysis of risk factors for pulmonary infection in patients with minimally invasive esophagectomy | Oncology Letters | | 10.3892/ol.2019.9987 |
| **288** | J. J. Liu, D. M. Freedman, M. P. Little, M. M. Doody, B. H. Alexander, C. M. Kitahara, T. Lee, P. Rajaraman, J. S. Miller, D. M. Kampa, S. L. Simon, D. L. Preston and M. S. Linet | 2014 | | Work history and mortality risks in 90 268 US radiological technologists | Occupational and Environmental Medicine | | 10.1136/oemed-2013-101859 |
| **289** | J. W. Liu, Y. J. Chen, X. Y. Zhan, Y. F. Yu and H. R. Yao | 2022 | | Effect of prior cancer history on survival of patients with esophageal carcinoma: a propensity score matching, population-based study | Journal of Thoracic Disease | | 10.21037/jtd-21-1707 |
| **290** | L. L. Liu, C. Y. Huang, W. Liao, S. W. Chen and S. H. Cai | 2020 | | Smoking behavior and smoking index as prognostic indicators for patients with esophageal squamous cell carcinoma who underwent surgery: A large cohort study in Guangzhou, China | Tobacco Induced Diseases | | 10.18332/tid/117428 |
| **291** | X. D. Liu, X. R. Wang, S. H. Lin, X. Q. Lao, J. Zhao, Q. K. Song, X. F. Su and I. T. S. Yu | 2017 | | Dietary patterns and the risk of esophageal squamous cell carcinoma: A population-based case control study in a rural population | Clinical Nutrition | | 10.1016/j.clnu.2015.11.009 |
| **292** | X. L. Liu, R. C. Wang, Y. Y. Liu, H. Chen, C. Qi, L. W. Hu, J. Yi and W. Wang | 2021 | | Risk prediction nomogram for major morbidity related to primary resection for esophageal squamous cancer | Medicine | | 10.1097/md.0000000000026189 |
| **293** | X. X. Liu, M. G. Zhou, F. Wang, S. Mubarik, Y. F. Wang, R. T. Meng, F. Shi, H. Y. Wen and C. H. Yu | 2020 | | Secular Trend of Cancer Death and Incidence in 29 Cancer Groups in China, 1990-2017: A Joinpoint and Age-Period-Cohort Analysis | Cancer Management and Research | | 10.2147/cmar.S247648 |
| **294** | Y. T. Liu, J. H. Lee, M. K. Tsai, J. C. C. Wei and C. P. Wen | 2022 | | The effects of modest drinking on life expectancy and mortality risks: a population-based cohort study | Scientific Reports | | 10.1038/s41598-022-11427-x |
| **295** | Z. Q. Liu, C. Q. Lin, C. Suo, R. J. Zhao, L. Jin, T. J. Zhang and X. D. Chen | 2022 | | Metabolic dysfunction-associated fatty liver disease and the risk of 24 specific cancers | Metabolism-Clinical and Experimental | | 10.1016/j.metabol.2021.154955 |
| **296** | W. C. Lo, C. C. Ku, S. T. Chiou, C. C. Chan, C. L. Chen, M. S. Lai and H. H. Lin | 2017 | | Adult mortality of diseases and injuries attributable to selected metabolic, lifestyle, environmental, and infectious risk factors in Taiwan: a comparative risk assessment | Population Health Metrics | | 10.1186/s12963-017-0134-4 |
| **297** | L. Long and K. F. Lai | 2019 | | Characteristics of Chinese chronic cough patients | Pulmonary Pharmacology & Therapeutics | | 10.1016/j.pupt.2019.101811 |
| **298** | E. Loots, B. Sartorius, T. E. Madiba, C. J. J. Mulder and D. L. Clarke | 2017 | | Oesophageal squamous cell cancer in a South African tertiary hospital: a risk factor and presentation analysis | South African Journal of Surgery | |  |
| **299** | G. López-Abente, N. Aragonés, B. Pérez-Gómez, M. Pollán, J. García-Pérez, R. Ramis and P. Fernández-Navarro | 2014 | | Time trends in municipal distribution patterns of cancer mortality in Spain | Bmc Cancer | | 10.1186/1471-2407-14-535 |
| **300** | I. Lund and J. Scheffels | 2014 | | Perceptions of Relative Risk of Disease and Addiction From Cigarettes and Snus | Psychology of Addictive Behaviors | | 10.1037/a0032657 |
| **301** | L. Lundell, J. Hatlebakk, J. P. Galmiche, S. E. Attwood, C. Ell, R. Fiocca, T. Persson, P. Nagy, S. Eklund and T. Lind | 2015 | | Long-term effect on symptoms and quality of life of maintenance therapy with esomeprazole 20 mg daily: a <i>post hoc</i> analysis of the LOTUS trial | Current Medical Research and Opinion | | 10.1185/03007995.2014.980500 |
| **302** | M. Ma, S. Shroff, M. Feldman, M. DeMarshall, C. Price, A. Tierney and G. W. Falk | 2017 | | Risk of malignant progression in Barrett's esophagus indefinite for dysplasia | Diseases of the Esophagus | | 10.1093/dote/dow025 |
| **303** | J. S. MacLeod, M. A. Harris, M. Tjepkema, P. A. Peters and P. A. Demers | 2017 | | Cancer Risks among Welders and Occasional Welders in a National Population-Based Cohort Study: Canadian Census Health and Environmental Cohort | Safety and Health at Work | | 10.1016/j.shaw.2016.12.001 |
| **304** | F. B. Maguire, A. S. Movsisyan, C. R. Morris, A. Parikh-Patel, T. H. M. Keegan and E. K. Tong | 2022 | | Evaluation of Cancer Deaths Attributable to Tobacco in California, 2014-2019 | Jama Network Open | | 10.1001/jamanetworkopen.2022.46651 |
| **305** | R. Mahfouz, A. Barchuk, A. E. Obeidat, M. M. Mansour, D. Hernandez, M. Darweesh, M. Aldiabat, M. H. Al-Khateeb, M. H. Yusuf and Y. Aljabiri | 2022 | | The Relationship Between Obstructive Sleep Apnea (OSA) and Gastroesophageal Reflux Disease (GERD) in Inpatient Settings: A Nationwide Study | Cureus Journal of Medical Science | | 10.7759/cureus.22810 |
| **306** | A. F. T. Malekshah, M. Zaroudi, A. Etemadi, F. Islami, S. Sepanlou, M. Sharafkhah, A. A. Keshtkar, H. Khademi, H. Poustchi, A. Hekmatdoost, A. Pourshams, A. F. Sani, E. Jafari, F. Kamangar, S. M. Dawsey, C. C. Abnet, P. D. Pharoah, P. J. Berennan, P. Boffetta, A. Esmaillzadeh and R. Malekzadeh | 2016 | | The Combined Effects of Healthy Lifestyle Behaviors on All-Cause Mortality: The Golestan Cohort Study | Archives of Iranian Medicine | |  |
| **307** | Z. Malik, L. Bayman, J. Valestin, A. Rizvi-Toner, S. Hashmi and R. Schey | 2017 | | Dronabinol increases pain threshold in patients with functional chest pain: a pilot double-blind placebo-controlled trial | Diseases of the Esophagus | | 10.1111/dote.12455 |
| **308** | S. Manavalan, B. Getachew, K. F. Manaye, S. J. Khundmiri, A. B. Csoka, R. McKinley, A. Tamas, D. Reglodi and Y. Tizabi | 2017 | | PACAP Protects Against Ethanol and Nicotine Toxicity in SH-SY5Y Cells: Implications for Drinking-Smoking Co-morbidity | Neurotoxicity Research | | 10.1007/s12640-017-9727-8 |
| **309** | F. Mano, K. Ikeda, T. Sato, T. Nakayama, D. Tanaka, E. Joo, Y. Takahashi, S. Kosugi, A. Sekine, Y. Tabara, F. Matsuda, N. Inagaki and G. Nagahama Study | 2018 | | Reduction in Gastroesophageal Reflux Disease Symptoms Is Associated with <i>Miso</i> Soup Intake in a Population-Based Cross-Sectional Study: The Nagahama Study | Journal of Nutritional Science and Vitaminology | | 10.3177/jnsv.64.367 |
| **310** | J. Maret-Ouda, G. Santoni, S. H. Xie, A. Rosengren and J. Lagergren | 2022 | | Proton Pump Inhibitor and Clopidogrel Use After Percutaneous Coronary Intervention and Risk of Major Cardiovascular Events | Cardiovascular Drugs and Therapy | | 10.1007/s10557-021-07219-6 |
| **311** | M. Mariani, R. Pastorino, D. P. Marafon, K. C. Johnson, J. F. Hu, A. J. M. de la Torre, G. Fernández-Tardón, D. Zaridze, D. Maximovich, E. Negri, C. La Vecchia, Z. F. Zhang, R. C. Kurtz, C. Pelucchi, M. Rota and S. Boccia | 2023 | | Leisure-time physical activity and gastric cancer risk: A pooled study within the Stomach cancer Pooling (StoP) Project | Plos One | | 10.1371/journal.pone.0286958 |
| **312** | I. Marijanovic, M. Kraljevic, D. B. Glibo, T. Buhovac and E. C. Obrdalj | 2021 | | THE ROLE OF FAMILY PHYSICIANS IN THE PREVENTION AND EARLY DETECTION OF CANCER IN HERZEGOVINA-NERETVA AND WEST-HERZEGOVINA CANTON | Psychiatria Danubina | |  |
| **313** | T. A. Markel, C. Proctor, J. Ying and P. D. Winchester | 2015 | | Environmental pesticides increase the risk of developing hypertrophic pyloric stenosis | Journal of Pediatric Surgery | | 10.1016/j.jpedsurg.2014.12.009 |
| **314** | M. A. Marks and E. A. Engels | 2014 | | Venous Thromboembolism and Cancer Risk among Elderly Adults in the United States | Cancer Epidemiology Biomarkers & Prevention | | 10.1158/1055-9965.Epi-13-1138 |
| **315** | G. Martimianaki, P. Bertuccio, G. Alicandro, C. Pelucchi, F. Bravi, G. Carioli, R. Bonzi, C. S. Rabkin, L. M. Liao, R. Sinha, K. Johnson, J. F. Hu, D. Palli, M. Ferraroni, N. Lunet, S. Morais, S. Tsugane, A. Hidaka, G. S. Hamada, L. López-Carrillo, R. U. Hernández-Ramírez, D. Zaridze, D. Maximovitch, N. Aragonés, V. Martin, M. H. Ward, J. Vioque, M. G. de la Hera, Z. F. Zhang, R. C. Kurtz, P. Lagiou, A. Lagiou, A. Trichopoulou, A. Karakatsani, R. Malekzadeh, M. C. Camargo, M. P. Curado, S. Boccia, P. Boffetta, E. Negri and C. La Vecchia | 2022 | | Coffee consumption and gastric cancer: a pooled analysis from the Stomach cancer Pooling Project consortium | European Journal of Cancer Prevention | | 10.1097/cej.0000000000000680 |
| **316** | M. C. L. Martins, D. L. Miyazaki, C. C. T. Gabiatti, L. P. Silva, L. T. Macedo, N. S. Siqueira, N. A. Andreollo and J. B. C. Carvalheira | 2019 | | Chagasic Megaesophagus-Associated Carcinoma: Clinical Pattern and Outcomes | Journal of Global Oncology | | 10.1200/jgo.19.00143 |
| **317** | K. Matsueda, R. Ishihara, T. Morishima, Y. Okubo, Y. Kawakami, H. Sakurai, T. Nakamura, Y. Tani, M. Miyake, S. Shichijo, A. Maekawa, T. Kanesaka, S. Yamamoto, Y. Takeuchi, K. Higashino, N. Uedo, T. Michida, T. Matsunaga, Y. Ohno, T. Sobue and I. Miyashiro | 2022 | | Impact of endoscopic surveillance on mortality of metachronous esophageal and head and neck cancer after esophageal endoscopic resection | Journal of Gastroenterology and Hepatology | | 10.1111/jgh.15984 |
| **318** | K. Matsuo, S. W. Lee, R. Tanaka, Y. Imai, K. Honda, K. Taniguchi, H. Tomiyama and K. Uchiyama | 2021 | | T stage and venous invasion are crucial prognostic factors for long-term survival of patients with remnant gastric cancer: a cohort study | World Journal of Surgical Oncology | | 10.1186/s12957-021-02400-5 |
| **319** | J. A. McDonald and L. J. Paulozzi | 2019 | | Parsing the Paradox: Hispanic Mortality in the US by Detailed Cause of Death | Journal of Immigrant and Minority Health | | 10.1007/s10903-018-0737-2 |
| **320** | D. M. McElvenny, B. G. Miller, L. A. MacCalman, A. Sleeuwenhoek, M. van Tongeren, K. Shepherd, A. J. Darnton and J. W. Cherrie | 2015 | | Mortality of a cohort of workers in Great Britain with blood lead measurements | Occupational and Environmental Medicine | | 10.1136/oemed-2014-102637 |
| **321** | D. M. McElvenny, W. Mueller, P. Ritchie, J. W. Cherrie, M. Hidajat, A. J. Darnton, R. M. Agius and F. de Vocht | 2018 | | British rubber and cable industry cohort: 49-year mortality follow-up | Occupational and Environmental Medicine | | 10.1136/oemed-2017-104834 |
| **322** | D. M. McElvenny, W. Mueller, P. Ritchie, J. W. Cherrie, M. Hidajat, A. J. Darnton, R. M. Agius and F. de Vocht | 2018 | | British rubber and cable industry cohort: 49-year mortality follow-up | Occupational and Environmental Medicine | | 10.1136/oemed-2017-104834 |
| **323** | G. A. Medhanie, S. A. Fedewa, H. Adissu, C. E. DeSantis, R. L. Siegel and A. Jemal | 2017 | | Cancer Incidence Profile in Sub-Saharan African-Born Blacks in the United States: Similarities and Differences With US-Born Non-Hispanic Blacks | Cancer | | 10.1002/cncr.30701 |
| **324** | G. A. Medhanie, S. A. Fedewa, H. Adissu, C. E. DeSantis, R. L. Siegel and A. Jemal | 2017 | | Cancer Incidence Profile in Sub-Saharan African-Born Blacks in the United States: Similarities and Differences With US-Born Non-Hispanic Blacks | Cancer | | 10.1002/cncr.30701 |
| **325** | D. Menya, N. Kigen, M. Oduor, S. K. Maina, F. Some, D. Chumba, P. Ayuo, O. Osano, D. R. S. Middleton, J. Schüz and V. A. McCormack | 2019 | | Traditional and commercial alcohols and esophageal cancer risk in Kenya | International Journal of Cancer | | 10.1002/ijc.31804 |
| **326** | D. S. Michaud, K. T. Kelsey, E. Papathanasiou, C. A. Genco and E. Giovannucci | 2016 | | Periodontal disease and risk of all cancers among male never smokers: an updated analysis of the Health Professionals Follow-up Study | Annals of Oncology | | 10.1093/annonc/mdw028 |
| **327** | D. R. S. Middleton, D. Menya, N. Kigen, M. Oduor, S. K. Maina, F. Some, D. Chumba, P. Ayuo, O. Osano, J. Schüz and V. McCormack | 2019 | | Hot beverages and oesophageal cancer risk in western Kenya: Findings from the ESCCAPE case-control study | International Journal of Cancer | | 10.1002/ijc.32032 |
| **328** | S. Mignozzi, C. Santucci, H. N. Medina, E. Negri, C. La Vecchia and P. S. Pinheiro | 2024 | | Cancer mortality in Germany-born Americans and Germans | Cancer Epidemiology | | 10.1016/j.canep.2023.102519 |
| **329** | S. S. Mitter, R. Vedanthan, F. Islami, A. Pourshams, H. Khademi, F. Kamangar, C. C. Abnet, S. M. Dawsey, P. D. Pharoah, P. Brennan, V. Fuster, P. Boffetta and R. Malekzadeh | 2016 | | Household Fuel Use and Cardiovascular Disease Mortality Golestan Cohort Study | Circulation | | 10.1161/circulationaha.115.020288 |
| **330** | B. T. Mmbaga, A. Mwasamwaja, G. Mushi, A. Mremi, G. Nyakunga, I. Kiwelu, R. Swai, G. Kiwelu, S. Mustapha, E. Mghase, A. McHome, R. D. Shao, E. Mallya, D. S. Rwakatema, K. Kilonzo, O. M. Munishi, B. Abedi-Ardekani, D. Middleton, J. Schuz and V. McCormack | 2021 | | Missing and decayed teeth, oral hygiene and dental staining in relation to esophageal cancer risk: ESCCAPE case-control study in Kilimanjaro, Tanzania | International Journal of Cancer | | 10.1002/ijc.33433 |
| **331** | F. Mohammadzadeh, H. Noorkojuri, M. A. Pourhoseingholi, S. Saadat and A. R. Baghestani | 2015 | | Predicting the probability of mortality of gastric cancer patients using decision tree | Irish Journal of Medical Science | | 10.1007/s11845-014-1100-9 |
| **332** | Y. Mok, D. K. Son, Y. D. Yun, S. H. Jee and J. M. Samet | 2016 | | γ-Glutamyltransferase and cancer risk: The Korean cancer prevention study | International Journal of Cancer | | 10.1002/ijc.29659 |
| **333** | A. J. Montiel-Jarquín, L. G. V. de Lara-Cisneros, A. López-Colombo, H. A. Solís-Mendoza, M. L. Palmer-Márquez and M. S. Romero-Figueroa | 2019 | | Expression of metalloproteinase-9 in patients with mild and severe forms of gastroesophageal reflux disease | Cirugia Y Cirujanos | | 10.24875/ciru.18000691 |
| **334** | S. C. Moore, I. M. Lee, E. Weiderpass, P. T. Campbell, J. N. Sampson, C. M. Kitahara, S. K. Keadle, H. Arem, A. B. de Gonzalez, P. Hartge, H. O. Adami, C. K. Blair, K. B. Borch, E. Boyd, D. P. Check, A. Fournier, N. D. Freedman, M. Gunter, M. Johannson, K. T. Khaw, M. S. Linet, N. Orsini, Y. Park, E. Riboli, K. Robien, C. Schairer, H. Sesso, M. Spriggs, R. Van Dusen, A. Wolk, C. E. Matthews and A. V. Patel | 2016 | | Association of Leisure-Time Physical Activity With Risk of 26 Types of Cancer in 1.44 Million Adults | Jama Internal Medicine | | 10.1001/jamainternmed.2016.1548 |
| **335** | S. Moossavi, M. Mohamadnejad, A. Pourshams, H. Poustchi, F. Islami, M. Sharafkhah, B. Mirminachi, S. Nasseri-Moghaddam, S. Semnani, R. Shakeri, A. Etemadi, S. Merat, M. Khoshnia, S. M. Dawsey, P. D. Pharoah, P. Brennan, C. C. Abnet, P. Boffetta, F. Kamangar and R. Malekzadeh | 2018 | | Opium Use and Risk of Pancreatic Cancer: A Prospective Cohort Study | Cancer Epidemiology Biomarkers & Prevention | | 10.1158/1055-9965.Epi-17-0592 |
| **336** | S. Morais, B. Peleteiro, N. Araújo, R. Malekzadeh, W. M. Ye, A. Plymoth, S. Tsugane, A. Hidaka, G. S. Hamada, L. López-Carrillo, D. Zaridze, D. Maximovich, N. Aragonés, G. Castaño-Vinyals, M. Pakseresht, R. U. Hernández-Ramírez, M. López-Cervantes, M. Leja, E. Gasenko, F. Pourfarzi, Z. F. Zhang, G. P. Yu, M. H. Derakhshan, C. Pelucchi, E. Negri, C. La Vecchia and N. Lunet | 2022 | | Identifying the Profile of <i>Helicobacter pylori</i>-Negative Gastric Cancers: A Case-Only Analysis within the Stomach Cancer Pooling (StoP) Project | Cancer Epidemiology Biomarkers & Prevention | | 10.1158/1055-9965.Epi-21-0402 |
| **337** | C. T. Morgan, J. P. Kanne, E. E. Lewis, J. D. Maloney, M. M. DeCamp and D. P. McCarthy | 2023 | | One hundred cases of primary spontaneous pneumomediastinum: leukocytosis is common, pleural effusions and age over 40 are rare | Journal of Thoracic Disease | | 10.21037/jtd-22-1136 |
| **338** | J. G. Morland, P. Magnus, S. E. Vollset, D. A. Leon, R. Selmer and A. Tverdal | 2023 | | Associations between serum high-density lipoprotein cholesterol levels and cause-specific mortality in a general population of 345 000 men and women aged 20-79 years | International Journal of Epidemiology | | 10.1093/ije/dyad011 |
| **339** | J. N. Morris, J. Loyer and J. Blunt | 2024 | | Stigma, risks, and benefits of medicinal cannabis use among Australians with cancer | Supportive Care in Cancer | | 10.1007/s00520-024-08439-w |
| **340** | M. J. Morris, R. J. Walter, E. T. McCann, J. H. Sherner, C. G. Murillo, B. S. Barber, J. C. Hunninghake and A. B. Holley | 2020 | | Clinical Evaluation of Deployed Military Personnel With Chronic Respiratory Symptoms Study of Active Duty Military for Pulmonary Disease Related to Environmental Deployment Exposures (STAMPEDE) III | Chest | | 10.1016/j.chest.2020.01.024 |
| **341** | N. Mostafalou, Y. Yahyapour, S. Sedaghat, J. S. Shirvani, M. HajiAhmadi, S. Siadati and S. Shafaei | 2015 | | Human papilloma virus infection in non-cancerous versus normal esophageal tissue samples by endoscopy | Caspian Journal of Internal Medicine | |  |
| **342** | L. C. Murnane, A. K. Forsyth, J. Koukounaras, C. H. C. Pilgrim, K. Shaw, W. A. Brown, M. Mourtzakis, A. C. Tierney and P. R. Burton | 2021 | | Low muscularity increases the risk for post-operative pneumonia and delays recovery from complications after oesophago-gastric cancer resection | Anz Journal of Surgery | | 10.1111/ans.17203 |
| **343** | M. F. Mushi, N. Ngeta, M. M. Mirambo and S. E. Mshana | 2018 | | Predictors of esophageal candidiasis among patients attending endoscopy unit in a tertiary hospital, Tanzania: a retrospective cross-sectional study | African Health Sciences | | 10.4314/ahs.v18i1.10 |
| **344** | M. F. Mushi, N. Ngeta, M. M. Mirambo and S. E. Mshana | 2018 | | Predictors of esophageal candidiasis among patients attending endoscopy unit in a tertiary hospital, Tanzania: a retrospective cross-sectional study | African Health Sciences | | 10.4314/ahs.v18i1.10 |
| **345** | M. M. Mwachiro, N. Pritchett, A. M. Calafat, R. K. Parker, J. O. Lando, G. Murphy, R. Chepkwony, S. L. Burgert, C. C. Abnet, M. D. Topazian, R. E. White, S. M. Dawsey and A. Etemadi | 2021 | | Indoor wood combustion, carcinogenic exposure and esophageal cancer in southwest Kenya | Environment International | | 10.1016/j.envint.2021.106485 |
| **346** | D. Mysíková, I. Adkins, N. Hradilová, O. Palata, J. Simonek, J. Pozniak, J. Kolarík, A. Skallová-Fialová, R. Spísek and R. Lischke | 2017 | | Case-Control Study: Smoking History Affects the Production of Tumor Antigen-Specific Antibodies NY-ESO-1 in Patients with Lung Cancer in Comparison with Cancer Disease-Free Group | Journal of Thoracic Oncology | | 10.1016/j.jtho.2016.09.136 |
| **347** | D. Myti, M. Gunjak, F. Casado, S. K. Raziabad, C. Nardiello, I. Vadász, S. Herold, G. Pryhuber, W. Seeger and R. E. Morty | 2020 | | Elevated FiO<sub>2</sub> increases SARS-CoV-2 co-receptor expression in respiratory tract epithelium | American Journal of Physiology-Lung Cellular and Molecular Physiology | | 10.1152/ajplung.00345.2020 |
| **348** | N. Nagata, T. Nishijima, R. Niikura, T. Yokoyama, Y. Matsushita, K. Watanabe, K. Teruya, Y. Kikuchi, J. Akiyama, M. Yanase, N. Uemura, S. Oka and H. Gatanaga | 2018 | | Increased risk of non-AIDS-defining cancers in Asian HIV-infected patients: a long-term cohort study | Bmc Cancer | | 10.1186/s12885-018-4963-8 |
| **349** | A. Naghibzadeh-Tahami, M. Marzban, V. Yazdi-Feyzabadi, S. Dabiri, S. Mohseni, R. A. Rayeni, M. S. Fekri, M. H. Larizadeh, B. Karimpour and N. Khanjani | 2020 | | Is opium use associated with an increased risk of lung cancer? A case-control study | Bmc Cancer | | 10.1186/s12885-020-07296-0 |
| **350** | A. Naghibzadeh-Tahami, M. Marzban, V. Yazdi-Feyzabadi, Z. Khazaei, M. J. Zahedi, V. Moazed and A. A. Haghdoost | 2021 | | Opium use as an independent risk factor for pancreatic cancer: A case-control study | Cancer Epidemiology | | 10.1016/j.canep.2021.102017 |
| **351** | A. Naghibzadeh-Tahami, M. Marzban, V. Yazdi-Feyzabadi, Z. Khazaei, M. J. Zahedi, V. Moazed and A. A. Haghdoost | 2021 | | Opium use as an independent risk factor for pancreatic cancer: A case-control study | Cancer Epidemiology | | 10.1016/j.canep.2021.102017 |
| **352** | M. Nalini, E. Oranuba, H. Poustchi, S. G. Sepanlou, A. Pourshams, M. Khoshnia, A. Gharavi, S. M. Dawsey, C. C. Abnet, P. Boffetta, P. Brennan, M. Sotoudeh, A. Nikmanesh, S. Merat, A. Etemadi, R. Shakeri, A. A. Sohrabpour, S. Nasseri-Moghaddam, F. Kamangar and R. Malekzadeh | 2018 | | Causes of premature death and their associated risk factors in the Golestan Cohort Study, Iran | Bmj Open | | 10.1136/bmjopen-2018-021479 |
| **353** | M. Nalini, S. G. Sepanlou, A. Pourshams, H. Poustchi, M. Sharafkhah, H. Bahrami, F. Kamangar and R. Malekzadeh | 2018 | | Drug Use for Secondary Prevention of Cardiovascular Diseases in Golestan, Iran: Results From the Golestan Cohort Study | Archives of Iranian Medicine | |  |
| **354** | S. Y. Nam, J. Jo, S. W. Jeon and H. Chun | 2023 | | Sex-specific effects of fruit, vegetable, and red meat intake on the risk of gastric and esophageal cancer in a large cohort | Digestive and Liver Disease | | 10.1016/j.dld.2023.02.021 |
| **355** | M. C. Narendra, B. Ramakrishna, Y. Mutheeswaraiah, N. Rukmangadha, K. A. Sarma and A. M. Pavan | 2022 | | A Retrospective Clinical Study of Gastroduodenal Perforation Peritonitis | Journal of Research in Medical and Dental Science | |  |
| **356** | C. T. Narh, C. P. Dzamalala, B. T. Mmbaga, D. Menya, Y. Mlombe, P. Finch, G. Nyakunga, J. Schüz, V. McCormack and E. Team | 2021 | | Geophagia and risk of squamous cell esophageal cancer in the African esophageal cancer corridor: Findings from the ESCCAPE multicountry case-control studies | International Journal of Cancer | | 10.1002/ijc.33688 |
| **357** | U. Nasir, B. Rodgers, D. Panchal, C. Choi, S. Ahmed and S. Ahlawat | 2020 | | Ferrous Sulfate-Induced Esophageal Injury Leading to Esophagitis Dissecans Superficialis | Case Reports in Gastroenterology | | 10.1159/000506935 |
| **358** | M. Nasrazadani, M. R. Maracy, E. Dreassi and B. Mahaki | 2018 | | Mapping of Stomach, Colorectal, and Bladder Cancers in Iran, 2004-2009: Applying Bayesian Polytomous Logit Model | International Journal of Preventive Medicine | | 10.4103/ijpvm.IJPVM_30_17 |
| **359** | D. Nasrollahzadeh, W. M. Ye, R. Shakeri, M. Sotoudeh, S. Merat, F. Kamangar, C. C. Abnet, F. Islami, P. Boffetta, S. M. Dawsey, P. Brennan and R. Malekzadeh | 2015 | | Contact with ruminants is associated with esophageal squamous cell carcinoma risk | International Journal of Cancer | | 10.1002/ijc.29109 |
| **360** | A. Nath, K. SathishKumar, P. Das, S. K. Lakshminarayana, S. Santhappan, S. Natarajan, S. Karuppasamy, S. Narasimhan, R. Venkataiah and P. Mathur | 2023 | | Need for accelerating tobacco control in India: findings from the national cancer registry programme | European Journal of Cancer Prevention | | 10.1097/cej.0000000000000759 |
| **361** | F. Navab, B. H. Nathanson and D. J. Desilets | 2015 | | The impact of lifestyle on Barrett's Esophagus: A precursor to esophageal adenocarcinoma | Cancer Epidemiology | | 10.1016/j.canep.2015.10.013 |
| **362** | C. Nelson, J. Lee, K. Ko, A. G. Sikora, M. D. Bonnen, P. Enkhbaatar and Y. T. Ghebre | 2017 | | Therapeutic Efficacy of Esomeprazole in Cotton Smoke-Induced Lung Injury Model | Frontiers in Pharmacology | | 10.3389/fphar.2017.00016 |
| **363** | S. Nemati, E. Saeedi, F. Lotfi, A. Nahvijou, E. Mohebbi, Z. Ravankhah, A. Rezaeianzadeh, M. Yaghoobi-Ashrafi, H. Pirnejad, A. Golpazir, R. Dolatkhah, S. Alvand, S. V. Ahmadi-Tabatabaei, M. Cheraghi, E. Weiderpass, F. Bray, M. P. Coleman, A. Etemadi, A. Khosravi, F. Najafi, M. A. Mohagheghi, G. Roshandel, R. Malekzadeh and K. Zendehdel | 2022 | | National surveillance of cancer survival in Iran (IRANCANSURV): Analysis of data of 15 cancer sites from nine population-based cancer registries | International Journal of Cancer | | 10.1002/ijc.34224 |
| **364** | E. Ness-Jensen, G. Santoni, E. Gottlieb-Vedi, A. Lindam, N. Pedersen and J. Lagergren | 2020 | | Mortality in gastro-oesophageal reflux disease in a population-based nationwide cohort study of Swedish twins | Bmj Open | | 10.1136/bmjopen-2020-037456 |
| **365** | T. X. T. Nguyen, M. Han and J. K. Oh | 2019 | | The economic burden of cancers attributable to smoking in Korea, 2014 | Tobacco Induced Diseases | | 10.18332/tid/102673 |
| **366** | R. Nikkilä, E. Hirvonen, A. Haapaniemi, L. Tapiovaara, J. Pitkäniemi, N. Malila and A. Mäkitie | 2023 | | Significant risk of second primary cancer among laryngeal squamous cell carcinoma patients even after 20 years | Acta Oncologica | | 10.1080/0284186x.2023.2254482 |
| **367** | R. Nikkilä, E. Hirvonen, J. Pitkäniemi, J. Räsänen, N. K. Malila and A. Makitie | 2024 | | Risk of Second Primary Cancer Among Patients with Cardio-Esophageal Cancer in Finland: A Nationwide Population-Based Study | Clinical Epidemiology | | 10.2147/clep.S471802 |
| **368** | R. Nikkilä, M. Peltomaa, T. Carpén, J. I. Martinsen, S. Heikkinen, J. Selander, I. S. Mehlum, J. E. Torfadottir, A. Mäkitie and E. Pukkala | 2023 | | Cancer incidence among visual artists: 45 years of follow-up in four Nordic countries | Acta Oncologica | | 10.1080/0284186x.2023.2263150 |
| **369** | A. N. Niles and A. O'Donovan | 2019 | | Comparing Anxiety and Depression to Obesity and Smoking as Predictors of Major Medical Illnesses and Somatic Symptoms | Health Psychology | | 10.1037/hea0000707 |
| **370** | T. Nishino, T. Yoshida, S. Inoue, S. Fujiwara, M. Goto, T. Minato, Y. Furukita, Y. Yamamoto, Y. Yuasa, H. Yamai, H. Takechi, H. Toba, H. Takizawa, M. Yoshida, J. Seike, T. Miyoshi and A. Tangoku | 2017 | | Gender differences in clinicopathological features and prognosis of squamous cell carcinoma of the esophagus | Esophagus | | 10.1007/s10388-016-0554-4 |
| **371** | T. B. Nobel, J. Livschitz, M. Eljalby, Y. Y. Janjigian, M. S. Bains, P. S. Adusumilli, D. R. Jones and D. Molena | 2020 | | Unique Considerations for Females Undergoing Esophagectomy | Annals of Surgery | | 10.1097/sla.0000000000003202 |
| **372** | A. M. Noone, K. A. Cronin, S. F. Altekruse, N. Howlader, D. R. Lewis, V. I. Petkov and L. Penberthy | 2017 | | Cancer Incidence and Survival Trends by Subtype Using Data from the Surveillance Epidemiology and End Results Program, 1992-2013 | Cancer Epidemiology Biomarkers & Prevention | | 10.1158/1055-9965.Epi-16-0520 |
| **373** | R. Nopour | 2023 | | Prediction of five-year survival among esophageal cancer patients using machine learning | Heliyon | | 10.1016/j.heliyon.2023.e22654 |
| **374** | G. Ntentas, K. Dedeckova, M. Andrlik, M. C. Aznar, R. Shakir, J. Ramroth, R. Begum, J. Kubes, S. C. Darby, N. G. Mikhaeel and D. J. Cutter | 2022 | | Proton Therapy in Supradiaphragmatic Lymphoma: Predicting Treatment-Related Mortality to Help Optimize Patient Selection | International Journal of Radiation Oncology Biology Physics | | 10.1016/j.ijrobp.2021.10.151 |
| **375** | N. N. Nwizu, J. R. Marshall, K. Moysich, R. J. Genco, K. M. Hovey, X. D. Mai, M. J. LaMonte, J. L. Freudenheim and J. Wactawski-Wende | 2017 | | Periodontal Disease and Incident Cancer Risk among Postmenopausal Women: Results from the Women's Health Initiative Observational Cohort | Cancer Epidemiology Biomarkers & Prevention | | 10.1158/1055-9965.Epi-17-0212 |
| **376** | A. H. Nyberg, E. Sadikova, C. Cheetham, K. M. Chiang, J. X. X. Shi, S. Caparosa, Z. M. Younossi and L. M. Nyberg | 2020 | | Increased cancer rates in patients with chronic hepatitis C | Liver International | | 10.1111/liv.14305 |
| **377** | S. Obayo, Y. Mulumba, C. L. Thompson, M. K. Gibson, M. M. Cooney and J. Orem | 2023 | | Clinicopathological characteristics and treatment outcomes of esophageal cancer patients in Uganda | Ecancermedicalscience | | 10.3332/ecancer.2023.1576 |
| **378** | A. A. Ocampo, R. M. Genta and E. S. Dellon | 2023 | | Mast Cell Esophagitis: A Novel Entity in Patients with Unexplained Esophageal Symptoms | Dysphagia | | 10.1007/s00455-023-10616-8 |
| **379** | E. Okada, S. Ukawa, K. Nakamura, M. Hirata, A. Nagai, K. Matsuda, T. Ninomiya, Y. Kiyohara, K. Muto, Y. Kamatani, Z. Yamagata, M. Kubo, Y. Nakamura, A. Tamakoshi and G. BioBank Japan Cooperative Hosp | 2017 | | Demographic and lifestyle factors and survival among patients with esophageal and gastric cancer: The Biobank Japan Project | Journal of Epidemiology | | 10.1016/j.je.2016.12.002 |
| **380** | F. Okada | 2014 | | Inflammation-Related Carcinogenesis: Current Findings in Epidemiological Trends, Causes and Mechanisms | Yonago Acta Medica | |  |
| **381** | M. Okuyama, O. Takaishi, K. Nakahara, N. Iwakura, T. Hasegawa, M. Oyama, A. Inoue, H. Ishizu, H. Satoh and Y. Fujiwara | 2017 | | Associations among gastroesophageal reflux disease, psychological stress, and sleep disturbances in Japanese adults | Scandinavian Journal of Gastroenterology | | 10.1080/00365521.2016.1224383 |
| **382** | G. H. Oliveira-Paula, L. C. Pinheiro and J. E. Tanus-Santos | 2019 | | Mechanisms impairing blood pressure responses to nitrite and nitrate | Nitric Oxide-Biology and Chemistry | | 10.1016/j.niox.2019.01.015 |
| **383** | A. Örmeci, B. Çavu, R. Akas, Z. Istemihan, Z. Imanov, V. Senkal, K. Nuriyev, A. Bayraktar, C. B. Külle, M. Keskin, K. Demir, F. Besisik, S. Kaymakoglu and F. Akyüz | 2022 | | What is the effect of subepithelial lesions of the esophagus on esophageal motility? | European Review for Medical and Pharmacological Sciences | |  |
| **384** | H. A. Osman, S. S. Aly, H. S. Mahmoud, E. H. Ahmed, E. M. S. Eldin, E. A. Abdelrahim, M. A. El Masry, R. A. Herdan and M. H. Hassan | 2019 | | Effect of Acid Suppression on Peripheral T-Lymphocyte Subsets and Immunohistochemical Esophageal Mucosal Changes in Patients With Gastroesophageal Reflux Disease | Journal of Clinical Gastroenterology | | 10.1097/mcg.0000000000001098 |
| **385** | P. Özdemir, M. Erdinç, R. Vardar, A. Veral, S. Akyildiz, Ö. Özdemir and S. Bor | 2017 | | The Role of Microaspiration in the Pathogenesis of Gastroesophageal Reflux-related Chronic Cough | Journal of Neurogastroenterology and Motility | | 10.5056/jnm16057 |
| **386** | K. F. Pan, L. Zhang, M. Gerhard, J. L. Ma, W. D. Liu, K. Ulm, J. X. Wang, L. Zhang, Y. Zhang, M. Bajbouj, L. F. Zhang, M. Li, M. Vieth, R. Y. Liu, M. Quante, L. H. Wang, S. Suchanek, T. Zhou, W. X. Guan, R. Schmid, M. Classen and W. C. You | 2016 | | A large randomised controlled intervention trial to prevent gastric cancer by eradication of <i>Helicobacter pylori</i> in Linqu County, China: baseline results and factors affecting the eradication | Gut | | 10.1136/gutjnl-2015-309197 |
| **387** | A. Pandey, S. C. Tripathi, S. Mahata, K. Vishnoi, S. Shukla, S. P. Misra, V. Misra, S. Hedau, R. Mehrotra, M. Dwivedi and A. C. Bharti | 2014 | | Carcinogenic <i>Helicobacter pylori</i> in gastric pre-cancer and cancer lesions: Association with tobacco-chewing | World Journal of Gastroenterology | | 10.3748/wjg.v20.i22.6860 |
| **388** | K. Parikh and L. Khaitan | 2016 | | Radiofrequency ablation coupled with Roux-en-Y gastric bypass: a treatment option for morbidly obese patients with Barrett's esophagus | Journal of Surgical Case Reports | | 10.1093/jscr/rjw007 |
| **389** | E. Park, H. Y. Kang, M. K. Lim, B. Kim and J. K. Oh | 2024 | | Cancer Risk Following Smoking Cessation in Korea | Jama Network Open | | 10.1001/jamanetworkopen.2023.54958 |
| **390** | G. W. Park, S. K. Kim, C. H. Lee, C. R. Kim, H. J. Jeong and D. K. Kim | 2015 | | Effect of Chronic Obstructive Pulmonary Disease on Swallowing Function in Stroke Patients | Annals of Rehabilitation Medicine-Arm | | 10.5535/arm.2015.39.2.218 |
| **391** | J. H. Park, J. Y. Hong, J. J. Shen, K. Han, J. O. Park, Y. S. Park and H. Y. Lim | 2023 | | Increased Risk of Young-Onset Digestive Tract Cancers Among Young Adults Age 20-39 Years With Nonalcoholic Fatty Liver Disease: A Nationwide Cohort Study | Journal of Clinical Oncology | | 10.1200/jco.22.01740 |
| **392** | W. Park, J. K. Lee, C. R. Kim and J. Y. Shin | 2015 | | Factors Associated with Fatigue in Korean Gastric Cancer Survivors | Korean Journal of Family Medicine | | 10.4082/kjfm.2015.36.6.328 |
| **393** | D. Pasquier, B. Bataille, F. Le Tinier, R. Bennadji, H. Langin, A. Escande, E. Tresch, F. Darloy, D. Carlier, F. Crop and E. Lartigau | 2021 | | Correlation between toxicity and dosimetric parameters for adjuvant intensity modulated radiation therapy of breast cancer: a prospective study | Scientific Reports | | 10.1038/s41598-021-83159-3 |
| **394** | S. Pasricha, A. Gupta, C. C. Reed, O. Speck, J. T. Woosley and E. S. Dellon | 2016 | | Lymphocytic Esophagitis: An Emerging Clinicopathologic Disease Associated with Dysphagia | Digestive Diseases and Sciences | | 10.1007/s10620-016-4230-2 |
| **395** | A. W. Pastuszak, N. Thirumavalavan, T. P. Kohn, L. I. Lipshultz and M. L. Eisenberg | 2019 | | Increased Risk of Cancer in Men With Peyronie's Disease: A Cohort Study Using a Large United States Insurance Claims Database | Sexual Medicine | | 10.1016/j.esxm.2019.08.007 |
| **396** | J. Patel, T. Khanna, A. Sohal, A. Dhaliwal, H. Chaudhry, S. Kalra, I. Singh, D. Dukovic and K. Bains | 2024 | | Impact of aspirin use on rates of metastasis in patients with esophageal cancer: insights from the National Inpatient Sample | Diseases of the Esophagus | | 10.1093/dote/doae022 |
| **397** | K. A. Patterson, P. J. Roberts-Thomson, S. Lester, J. A. Tan, P. Hakendorf, M. Rischmueller, J. Zochling, J. Sahhar, P. Nash, J. Roddy, C. Hill, M. Nikpour, W. Stevens, S. M. Proudman and J. G. Walker | 2015 | | Interpretation of an Extended Autoantibody Profile in a Well-Characterized Australian Systemic Sclerosis (Scleroderma) Cohort Using Principal Components Analysis | Arthritis & Rheumatology | | 10.1002/art.39316 |
| **398** | G. Paul, W. Bohle and W. Zoller | 2019 | | Risk Factors for the Development of Esophagorespiratory Fistula in Esophageal Cancer | Journal of Gastrointestinal and Liver Diseases | | 10.15403/jgld-271 |
| **399** | M. W. Pawlik, S. Kwiecien, R. Pajdo, A. Ptak-Belowska, B. Brzozowski, G. Krzysiek-Maczka, M. Strzalka, S. J. Konturek and T. Brzozowski | 2014 | | ESOPHAGOPROTECTIVE ACTIVITY OF ANGIOTENSIN-(1-7) IN EXPERIMENTAL MODEL OF ACUTE REFLUX ESOPHAGITIS. EVIDENCE FOR THE ROLE OF NITRIC OXIDE, SENSORY NERVES, HYPDXIA-INDUCIBLE FACTOR-1ALPHA AND PROINFLAMMATORY CYTOKINES | Journal of Physiology and Pharmacology | |  |
| **400** | J. Y. Peng, Y. H. Yu, W. M. Chen, B. Shia, M. Chen and S. Y. Wu | 2023 | | Association of Antihistamine Use with Increased Risk of Esophageal Squamous Cell Carcinoma: A Nationwide, Long-Term Follow-Up Study Using Propensity Score Matching | Biomedicines | | 10.3390/biomedicines11020578 |
| **401** | S. Peter, A. Pendergraft, W. VanderPol, C. M. Wilcox, K. Baig, C. Morrow, J. Izard and P. J. Mannon | 2020 | | Mucosa-Associated Microbiota in Barrett's Esophagus, Dysplasia, and Esophageal Adenocarcinoma Differ Similarly Compared With Healthy Controls | Clinical and Translational Gastroenterology | |  |
| **402** | B. A. Peters, J. Wu, Z. H. Pei, L. Y. Yang, M. P. Purdue, N. D. Freedman, E. J. Jacobs, S. M. Gapstur, R. B. Hayes and J. Ahn | 2017 | | Oral Microbiome Composition Reflects Prospective Risk for Esophageal Cancers | Cancer Research | | 10.1158/0008-5472.Can-17-1296 |
| **403** | J. L. Pierce, K. Tanner, R. M. Merrill, K. L. Miller, K. A. Kendall and N. Roy | 2016 | | Swallowing Disorders in Sjogren's Syndrome: Prevalence, Risk Factors, and Effects on Quality of Life | Dysphagia | | 10.1007/s00455-015-9657-7 |
| **404** | S. D. Pinto, L. H. D. Rodriguez, F. R. Takeda, M. R. Tacconi, R. A. A. Sallum, I. Cecconello and U. Ribeiro | 2022 | | CHARACTERIZATION OF THE PSYCHOLOGICAL TYPOLOGY IN ESOPHAGEAL CANCER PATIENTS | Abcd-Arquivos Brasileiros De Cirurgia Digestiva-Brazilian Archives of Digestive Surgery | | 10.1590/0102-672020220002e1715 |
| **405** | V. A. Pinto, E. Nascimento, A. P. L. Cunha, B. P. S. Assis, M. F. Lasmar, H. R. Vianna and R. A. Fabreti-Oliveira | 2022 | | Malignancy Diseases in Kidney Transplantation, Clinical Outcomes, Patient, and Allograft Survival: A Case-Control Study | Transplantation Proceedings | | 10.1016/j.transproceed.2022.02.063 |
| **406** | D. Praud, P. Bertuccio, C. Bosetti, F. Turati, M. Ferraroni and C. La Vecchia | 2014 | | Adherence to the Mediterranean diet and gastric cancer risk in Italy | International Journal of Cancer | | 10.1002/ijc.28620 |
| **407** | E. Pukkala, M. Peltomaa, A. Mäkitie, S. Heikkinen, K. Kjærheim, J. I. Martinsen, P. Sparén, L. Tryggvadottir and E. Weiderpass | 2021 | | Cancer incidence among musicians: 45 years of follow-up in four Nordic countries | Acta Oncologica | | 10.1080/0284186x.2021.1924403 |
| **408** | M. A. Qayyum, M. H. Sultan, Z. Farooq, K. Muddassir, T. Farooq and A. Irfan | 2022 | | Quantitative estimation of essential/toxic elemental levels in the serum of esophagus cancer patients in relation to controls | Environmental Science and Pollution Research | | 10.1007/s11356-022-21651-7 |
| **409** | G. R. Quinn, D. Ranum, E. Song, M. Linets, C. Keohane, H. Riah and P. Greenberg | 2017 | | Missed Diagnosis of Cardiovascular Disease in Outpatient General Medicine: Insights from Malpractice Claims Data | Joint Commission Journal on Quality and Patient Safety | | 10.1016/j.jcjq.2017.05.001 |
| **410** | H. K. Quintana, V. Herrera, C. Niño, B. Gómez and R. Roa | 2019 | | Assessing the knowledge, attitudes and perceptions of tobacco-associated diseases and how it is influenced by tobacco products advertisement, promotion and sponsorship while enforcing a strong and comprehensive ban in Panama: a cross-sectional study | Bmj Open | | 10.1136/bmjopen-2018-024373 |
| **411** | B. Qumseya, S. Yang and Y. Guo | 2024 | | Trends in prevalence of esophageal adenocarcinoma: Findings from a statewide database of over 6 million patients | Endoscopy International Open | | 10.1055/a-2221-7974 |
| **412** | L. Radulovic, J. Erakovic and M. Roganovic | 2020 | | Attitudes of patients with relapsing-remitting form of multiple sclerosis using disease-modifying drugs in Montenegro regarding COVID-19 pandemic | Multiple Sclerosis and Related Disorders | | 10.1016/j.msard.2020.102380 |
| **413** | M. G. Rajanandh, S. Suresh, K. Manobala, R. Nandhakumar, G. Jaswanthi and S. Neha | 2018 | | Prediction of cardiovascular risk in cancer patients of South India using WHO/ISH risk prediction charts and Framingham score - A prospective study | Journal of Oncology Pharmacy Practice | | 10.1177/1078155217707334 |
| **414** | J. M. Ramsay, M. J. Madsen, J. J. Horns, H. A. Hanson, N. J. Camp, B. R. Emery, K. Aston, E. Ferlic and J. M. Hotaling | 2024 | | Describing patterns of familial cancer risk in subfertile men using population pedigree data | Human Reproduction | | 10.1093/humrep/dead270 |
| **415** | M. Rastogi, D. Rastogi, S. Singh, A. Agarwal, B. P. Priyadarshi and T. Middha | 2015 | | Prevalence of <i>Helicobacter pylori</i> in asymptomatic adult patients in a tertiary care hospital: A cross sectional study | Biomedical Research-India | |  |
| **416** | A. S. Reece and G. K. Hulse | 2022 | | Epidemiology of Δ8THC-Related Carcinogenesis in USA: A Panel Regression and Causal Inferential Study | International Journal of Environmental Research and Public Health | | 10.3390/ijerph19137726 |
| **417** | A. S. Reece and G. K. Hulse | 2023 | | Congenital Gastrointestinal Anomalies in Europe 2010-2019: A Geo-Spatiotemporal and Causal Inferential Study of Epidemiological Patterns in Relationship to Cannabis- and Substance Exposure | Gastroenterology Insights | | 10.3390/gastroent14010007 |
| **418** | J. Rehm, I. Soerjomataram, C. Ferreira-Borges and K. D. Shield | 2019 | | Does Alcohol Use Affect Cancer Risk? | Current Nutrition Reports | | 10.1007/s13668-019-0267-0 |
| **419** | Z. W. Reichenbach, J. Sloan, A. Rizvi-Toner, L. Bayman, J. Valestin and R. Schey | 2015 | | A 4-week Pilot Study With the Cannabinoid Receptor Agonist Dronabinol and Its Effect on Metabolic Parameters in a Randomized Trial | Clinical Therapeutics | | 10.1016/j.clinthera.2015.07.023 |
| **420** | C. Ren, X. Y. Cai, M. Z. Qiu, D. S. Wang, F. H. Wang, H. Y. Luo and R. H. Xu | 2015 | | Impact of body mass index on survival of esophageal squamous carcinoma patients in southern China | Journal of Thoracic Disease | | 10.3978/j.issn.2072-1439.2014.10.12 |
| **421** | L. Renaud, M. N. Hilleret, E. Thimonier, O. Guillaud, F. Arbib, G. Ferretti, A. Jankowski, C. Chambon-Augoyard, D. Erard-Poinsot, T. Decaens, O. Boillot, V. Leroy and J. Dumortier | 2018 | | De Novo Malignancies Screening After Liver Transplantation for Alcoholic Liver Disease: A Comparative Opportunistic Study | Liver Transplantation | | 10.1002/lt.25336 |
| **422** | K. Repp, R. Lorbeer, T. Ittermann, S. Gläser, U. John, W. Hoffmann and H. Vötlzke | 2015 | | OCCUPATIONAL EXPOSURE TO ASBESTOS IS ASSOCIATED WITH INCREASED MORTALITY IN MEN RECRUITED FOR A POPULATION-BASED STUDY IN GERMANY | International Journal of Occupational Medicine and Environmental Health | | 10.13075/ijomeh.1896.00549 |
| **423** | D. B. Richardson, E. Rage, P. A. Demers, M. T. Do, N. DeBono, N. Fenske, V. Deffner, M. Kreuzer, J. Samet, C. Wiggins, M. K. Schubauer-Berigan, K. Kelly-Reif, L. Tomasek, L. B. Zablotska and D. Laurier | 2021 | | Mortality among uranium miners in North America and Europe: the Pooled Uranium Miners Analysis (PUMA) | International Journal of Epidemiology | | 10.1093/ije/dyaa195 |
| **424** | M. Riegler, I. Kristo, M. Nikolic, E. Rieder and S. F. Schoppmann | 2017 | | Update on the management of Barrett's esophagus in Austria | European Surgery-Acta Chirurgica Austriaca | | 10.1007/s10353-017-0504-y |
| **425** | L. S. Riquelme, J. E. G. García and D. M. Macías | 2022 | | Characterization of patients with esophageal tumors treated at the Celestino Hernandez Robau Hospital (2016-2017) | Medisur-Revista De Ciencias Medicas De Cienfuegos | |  |
| **426** | L. Rizzolo-Brime, A. Farran-Codina, R. Bou, L. Luján-Barroso, J. R. Quirós, P. Amiano, M. J. Sánchez, M. Rodríguez-Barranco, M. Guevara, C. Moreno-Iribas, A. Gasque, M. D. Chirlaque, S. M. Colorado-Yohar, J. M. H. Castaño, A. Agudo and P. Jakszyn | 2024 | | Nitrosyl-Heme and Heme Iron Intake from Processed Meats in Subjects from the EPIC-Spain Cohort | Nutrients | | 10.3390/nu16060878 |
| **427** | F. Roesch-Dietlen, A. D. Cano-Contreras, Y. J. Sánchez-Maza, J. M. Espinosa-González, M. A. Vázquez-Prieto, E. J. Valdés-de la, F. Díaz-Roesch, M. A. Carrasco-Arroniz, A. Cruz-Palacios, P. Grube-Pagola, A. Sumoza-Toledo, H. Vivanco-Cid, G. Mellado-Sánchez, A. Meixueiro-Daza, C. S. Silva-Cañetas, M. G. Carrillo-Toledo, R. Lagunes-Torres, M. Amieva-Balmori, P. C. Gómez-Castaño, J. U. Reyes-Huerta and J. M. Remes-Troche | 2018 | | Frequency of human papillomavirus infection in patients with gastrointestinal cancer | Revista De Gastroenterologia De Mexico | | 10.1016/j.rgmx.2017.09.003 |
| **428** | A. J. Romain, J. Marleau and A. Baillot | 2018 | | Impact of obesity and mood disorders on physical comorbidities, psychological well-being, health behaviours and use of health services | Journal of Affective Disorders | | 10.1016/j.jad.2017.08.065 |
| **429** | C. Rong, S. H. Shen, L. W. Xiao, Q. Huang, H. T. Lu, H. X. Wang, Z. X. Li and X. M. Wang | 2019 | | A Comparative Study on the Health Status and Behavioral Lifestyle of Centenarians and Non-centenarians in Zhejiang Province, China-A Cross-Sectional Study | Frontiers in Public Health | | 10.3389/fpubh.2019.00344 |
| **430** | A. Rosenfeld, D. G. Graham, S. Jevons, J. Ariza, D. Hagan, A. Wilson, S. J. Lovat, S. S. Sami, O. F. Ahmad, M. Novelli, M. R. Justo, A. Winstanley, E. M. Heifetz, M. Ben-Zecharia, U. Noiman, R. C. Fitzgerald, P. Sasieni, L. B. Lovat and B. S. Grp | 2020 | | Development and validation of a risk prediction model to diagnose Barrett's oesophagus (MARK-BE): a case-control machine learning approach | Lancet Digital Health | | 10.1016/s2589-7500(19)30216-x |
| **431** | S. Roshini, S. S. Kanna, K. N. Siri and S. G. Thomas | 2023 | | A Study on Definitive Role of Smoking Over Alcohol on Peptic Ulcer Disease | International Journal of Life Science and Pharma Research | | 10.22376/ijlpr.2023.13.3.L88-L93 |
| **432** | B. L. Rostron, J. Wang, A. Etemadi, S. Thakur, J. T. Chang, D. Bhandari, J. C. Botelho, V. R. De Jesús, J. Feng, M. H. Gail, M. Inoue-Choi, R. Malekzadeh, A. Pourshams, H. Poustchi, G. Roshandel, M. S. Shiels, Q. Wang, Y. S. Wang, B. Y. Xia, P. Boffetta, P. Brennan, C. C. Abnet, A. M. Calafat, L. Q. Wang, B. C. Blount, N. D. Freedman and C. M. Chang | 2021 | | Associations between Biomarkers of Exposure and Lung Cancer Risk among Exclusive Cigarette Smokers in the Golestan Cohort Study | International Journal of Environmental Research and Public Health | | 10.3390/ijerph18147349 |
| **433** | S. Roy, T. Reang and A. Kumar | 2018 | | TOBACCO USE AND BODY MASS INDEX AMONG ADULT POPULATION IN SELECTED URBAN AREA OF AGARTALA CITY- A CROSS-SECTIONAL STUDY | Journal of Evolution of Medical and Dental Sciences-Jemds | | 10.14260/jemds/2018/114 |
| **434** | W. Rui, C. C. Li, Q. Da, Y. Yue, L. Jing, R. R. Guo, Y. B. Cui, T. Y. Lu and B. Li | 2024 | | Analysis of the influencing factors in the long-term survival of esophageal cancer | Frontiers in Oncology | | 10.3389/fonc.2023.1274014 |
| **435** | S. Saez-Atienzar, S. Bandres-Ciga, R. G. Langston, J. J. Kim, S. W. Choi, R. H. Reynolds, Y. Abramzon, R. Dewan, S. Ahmed, J. E. Landers, R. Chia, M. Ryten, M. R. Cookson, M. A. Nalls, A. Chiò, B. J. Traynor, A. L. S. G. C. Int and Italsgen | 2021 | | Genetic analysis of amyotrophic lateral sclerosis identifies contributing pathways and cell types | Science Advances | | 10.1126/sciadv.abd9036 |
| **436** | A. Safari, M. Reazai, A. Tangestaninejad, A. R. Mafi and S. A. J. Mousavi | 2016 | | Opium consumption: A potential risk factor for lung cancer and pulmonary tuberculosis | Indian Journal of Cancer | | 10.4103/0019-509x.204755 |
| **437** | R. Sagami, K. Hayasaka, T. Ujihara, T. Iwaki, Y. Katsuyama, H. Harada, Y. Ome, G. Honda, S. I. Horiguchi, K. Murakami and Y. Amano | 2023 | | Role of EUS combined with a newly modified scoring system to detect pancreatic high-grade precancerous lesions | Endoscopic Ultrasound | | 10.4103/eus-d-21-00187 |
| **438** | E. Saito, S. Tanaka, S. K. Abe, M. Hirayabashi, J. Ishihara, K. Katanoda, Y. S. Lin, C. Nagata, N. Sawada, R. Takachi, A. Goto, J. Tanaka, K. Ueda, M. Hori, T. Matsuda and M. Inoue | 2023 | | Economic burden of cancer attributable to modifiable risk factors in Japan | Global Health & Medicine | | 10.35772/ghm.2023.01001 |
| **439** | Y. Sakata, K. Tominaga, M. Kato, H. Takeda, Y. Shimoyama, T. Takeuchi, R. Iwakiri, K. Furuta, K. Sakurai, T. Odaka, H. Kusunoki, A. Nagahara, K. Iwakiri, T. Furuta, K. Murakami, H. Miwa, Y. Kinoshita, K. Haruma, S. Takahashi, S. Watanabe, K. Higuchi, K. Fujimoto, M. Kusano, T. Arakawa and G. P. S. Grp | 2014 | | Clinical characteristics of elderly patients with proton pump inhibitor-refractory non-erosive reflux disease from the G-PRIDE study who responded to rikkunshito | Bmc Gastroenterology | | 10.1186/1471-230x-14-116 |
| **440** | T. Sakurai, A. Hoshino, K. Miyoshi, E. Yamada, M. Enomoto, J. Mazaki, H. Kuwabara, K. Iwasaki, Y. Ota, S. Tachibana, Y. Hayashi, T. Ishizaki and Y. Nagakawa | 2024 | | Long-term outcomes of robot-assisted versus minimally invasive esophagectomy in patients with thoracic esophageal cancer: a propensity score-matched study | World Journal of Surgical Oncology | | 10.1186/s12957-024-03358-w |
| **441** | S. Saleh, B. D. Liu, S. Trujillo, C. Thomas and R. Fass | 2023 | | The effect of combined oral contraceptives and Nexplanon on gastroesophageal reflux disease in premenopausal women: A nationwide database analysis | Neurogastroenterology and Motility | | 10.1111/nmo.14542 |
| **442** | S. A. Samjo, Z. Abbas, M. Asim and K. Tahir | 2020 | | The Pattern of Alcohol Consumption and the Severity of Alcohol-related Liver Disease in Patients Visiting the Liver Clinic | Cureus Journal of Medical Science | | 10.7759/cureus.7251 |
| **443** | C. Santucci, H. N. Medina, G. Carioli, E. Negri, C. La Vecchia and P. S. Pinheiro | 2022 | | Cancer mortality in Italian populations: differences between Italy and the USA | European Journal of Cancer Prevention | | 10.1097/cej.0000000000000712 |
| **444** | S. Sarvepalli, S. K. Garg, S. S. Sarvepalli, C. Anugwom, V. Wadhwa, P. N. Thota and M. R. Sanaka | 2019 | | Hospital Utilization in Patients With Gastric Cancer and Factors Affecting In-Hospital Mortality, Length of Stay, and Costs | Journal of Clinical Gastroenterology | | 10.1097/mcg.0000000000001016 |
| **445** | H. K. Sarvestani, R. D. Ghazvini, S. J. Hashemi, M. G. Shoar, S. Ansari, Z. Rafat, A. Ahmadi, P. Borghei, M. Elahi, A. R. Foroushani, M. I. Getso, S. Aboutalebian, F. Safari and P. Ardi | 2022 | | Molecular Characterization of Fungal Colonization on the Provox™ Tracheoesophageal Voice Prosthesis in Post Laryngectomy Patients | Iranian Journal of Public Health | |  |
| **446** | F. Sarwar, M. Saleem and F. Z. Zaidi | 2021 | | Prevalence of Gastroesophageal Reflux Disease in rural women presenting to a primary care hospital | Rawal Medical Journal | |  |
| **447** | H. Sato, Y. Nishikawa, H. Abe, H. Shiwaku, J. Shiota, C. Sato, H. Sakae, M. Ominami, Y. Hata, H. Fukuda, R. Ogawa, J. Nakamura, T. Tatsuta, Y. Ikebuchi, H. Yokomichi, S. Terai and H. Inoue | 2022 | | Esophageal carcinoma in achalasia patients managed with endoscopic submucosal dissection and peroral endoscopic myotomy: Japan Achalasia Multicenter Study | Digestive Endoscopy | | 10.1111/den.14197 |
| **448** | M. Sattar, S. Kanwal and A. Abbas | 2019 | | A COMPREHENSIVE STUDY ON GASTRO-ESOPHAGEAL REFLUX DISEASE AND ITS RISK FACTORS AMONG LOCAL POPULATION OF PAKISTAN | Indo American Journal of Pharmaceutical Sciences | | 10.5281/zenodo.2529622 |
| **449** | S. Sawai, I. Arshad, K. Hussain, A. Ahmed, P. Kumar and M. Sadiq | 2021 | | Esophageal Carcinoma and Predisposing Factors Among Patients Presented at Isra University Hospital Hyderabad | Pakistan Journal of Medical & Health Sciences | | 10.53350/pjmhs2115103058 |
| **450** | M. Schmidt, D. P. Ankerst, Y. Y. Chen, M. Wiethaler, J. Slotta-Huspenina, K. F. Becker, J. Horstmann, F. Kohlmayer, A. Lehmann, B. Linkohr, K. Strauch, R. M. Schmid, A. S. Quante and M. Quante | 2020 | | Epidemiologic Risk Factors in a Comparison of a Barrett Esophagus Registry (BarrettNET) and a Case-Control Population in Germany | Cancer Prevention Research | | 10.1158/1940-6207.Capr-19-0474 |
| **451** | J. Schwartz, C. Bashian, L. Kushnir, C. Nituica and G. J. Slotman | 2017 | | Variation in Clinical Characteristics of Women versus Men Preoperative for Laparoscopic Roux-en-Y Gastric Bypass: Analysis of 83,059 Patients | American Surgeon | |  |
| **452** | T. Schweiger, D. Kollmann, C. Nikolowsky, D. Traxler, E. Guenova, G. Lang, P. Birner, W. Klepetko, H. J. Ankersmit and K. Hoetzenecker | 2014 | | Carbonic anhydrase IX is associated with early pulmonary spreading of primary colorectal carcinoma and tobacco smoking | European Journal of Cardio-Thoracic Surgery | | 10.1093/ejcts/ezt542 |
| **453** | I. Sen, L. Yohanathan, J. M. Kärkkäinen and D. M. Nagorney | 2021 | | Current Indications and Long-Term Outcomes of Surgical Portosystemic Shunts in Adults | Journal of Gastrointestinal Surgery | | 10.1007/s11605-020-04643-1 |
| **454** | G. Senna, M. Latorre, M. Bugiani, M. Caminati, E. Heffler, D. Morrone, G. Paoletti, P. Parronchi, F. Puggioni, F. Blasi, G. W. Canonica, P. Paggiaro and S. Network | 2021 | | Sex Differences in Severe Asthma: Results From Severe Asthma Network in Italy-SANI | Allergy Asthma & Immunology Research | | 10.4168/aair.2021.13.2.219 |
| **455** | A. A. Senusi, J. Mather, D. Ola, L. A. Bergmeier, B. Gokani and F. Fortune | 2022 | | The impact of multifactorial factors on the Quality of Life of Behcet's patients over 10 years | Frontiers in Medicine | | 10.3389/fmed.2022.996571 |
| **456** | G. Senyondo, A. Khan, F. Malik and A. Oranu | 2022 | | Esophagitis Dissecans Superficialis: A Frequently Missed and Rarely Reported Diagnosis | Cureus Journal of Medical Science | | 10.7759/cureus.21647 |
| **457** | J. H. Seo, Y. D. Kim, C. S. Park, K. D. Han and Y. H. Joo | 2020 | | Hypertension is associated with oral, laryngeal, and esophageal cancer: a nationwide population-based study | Scientific Reports | | 10.1038/s41598-020-67329-3 |
| **458** | R. Shakeri, F. Kamangar, M. Mohamadnejad, R. Tabrizi, F. Zamani, A. Mohamadkhani, S. Nikfam, A. Nikmanesh, M. Sotoudeh, R. Sotoudehmanesh, B. Shahbazkhani, M. R. Ostovaneh, F. Islami, H. Poustchi, P. Boffetta, R. Malekzadeh and A. Pourshams | 2016 | | Opium use, cigarette smoking, and alcohol consumption in relation to pancreatic cancer | Medicine | | 10.1097/md.0000000000003922 |
| **459** | F. M. Shebl, A. W. Hsing, Y. Park, A. R. Hollenbeck, L. W. Chu, T. E. Meyer and J. Koshiol | 2014 | | Non-Steroidal Anti-Inflammatory Drugs Use Is Associated with Reduced Risk of Inflammation-Associated Cancers: NIH-AARP Study | Plos One | | 10.1371/journal.pone.0114633 |
| **460** | M. Sheikh, R. Shakeri, H. Poustchi, A. Pourshams, A. Etemadi, F. Islami, M. Khoshnia, A. Gharavi, G. Roshandel, H. Khademi, S. G. Sepanlou, M. Hashemian, A. Fazel, M. Zahedi, B. Abedi-Ardekani, P. Boffetta, S. M. Dawsey, P. D. Pharoah, M. Sotoudeh, N. D. Freedman, C. C. Abnet, N. E. Day, P. Brennan, F. Kamangar and R. Malekzadeh | 2020 | | Opium use and subsequent incidence of cancer: results from the Golestan Cohort Study | Lancet Global Health | |  |
| **461** | C. Shen, C. M. Schooling, W. M. Chan, S. Y. Lee, G. M. Leung and T. H. Lam | 2014 | | Self-reported diabetes and mortality in a prospective Chinese elderly cohort Study in Hong Kong | Preventive Medicine | | 10.1016/j.ypmed.2014.03.021 |
| **462** | S. Shen, J. L. Araujo, N. K. Altorki, J. R. Sonett, A. Rodriguez, K. Sungur-Stasik, C. F. Spinelli, A. I. Neugut and J. A. Abrams | 2017 | | Variation by stage in the effects of prediagnosis weight loss on mortality in a prospective cohort of esophageal cancer patients | Diseases of the Esophagus | | 10.1093/dote/dox073 |
| **463** | H. Shigaki, Y. Imamura, S. Mine, A. Okamura, T. Kurogochi, K. Yamashita and M. Watanabe | 2017 | | Clinicopathological features of esophageal squamous cell carcinoma in never smoker-never drinkers | Diseases of the Esophagus | | 10.1093/dote/dow019 |
| **464** | J. Shin, J. I. Zo and J. Lee | 2016 | | Management of long-term lung cancer survivors in Korea | Journal of the Korean Medical Association | | 10.5124/jkma.2016.59.4.294 |
| **465** | A. Shiomi, T. Miyake, S. Furukawa, B. Matsuura, O. Yoshida, T. Watanabe, A. Kanamoto, M. Miyazaki, H. Nakaguchi, Y. Tokumoto, M. Hirooka, M. Abe and Y. Hiasa | 2024 | | Combined effect of histological findings and diabetes mellitus on liver-related events in patients with metabolic dysfunction-associated steatotic liver disease | Hepatology Research | | 10.1111/hepr.14049 |
| **466** | N. Shivappa, J. R. Hébert and B. Rashidkhani | 2015 | | Dietary Inflammatory Index and Risk of Esophageal Squamous Cell Cancer in a Case-Control Study from Iran | Nutrition and Cancer-an International Journal | | 10.1080/01635581.2015.1082108 |
| **467** | N. Shivappa, A. Zucchetto, D. Serraino, M. Rossi, C. La Vecchia and J. R. Hébert | 2015 | | Dietary inflammatory index and risk of esophageal squamous cell cancer in a case-control study from Italy | Cancer Causes & Control | | 10.1007/s10552-015-0636-y |
| **468** | P. Shokuhi, N. J. O'Sullivan, H. C. Temperley, T. Russell, P. D. McEntee, B. J. Mehigan, P. H. McCormick, D. Gallagher, C. Gillham, J. Kennedy, M. E. Kelly and J. O. Larkin | 2024 | | Prognostic value of pre-operative mean corpuscular volume (MCV) in colorectal cancer | Irish Journal of Medical Science | | 10.1007/s11845-023-03571-8 |
| **469** | M. Shteiner, S. Kleinman, A. Shuster, V. Raiser, C. Ianculovici, H. Rachima and I. Kaplan | 2021 | | Submucosal Fibrotic Bands in Oral Lichen Planus: A Clinico-Pathological Investigation of a Newly Described Phenomenon | Head & Neck Pathology | | 10.1007/s12105-020-01203-6 |
| **470** | G. Sidorenkov, J. M. Vonk, M. Grzegorczyk, F. O. Cortés-Ibañez and G. H. de Bock | 2023 | | Factors associated with SARS-COV-2 positive test in Lifelines | Plos One | | 10.1371/journal.pone.0294556 |
| **471** | F. Sierra-Arango, D. M. Castaño, J. D. Forero, E. D. Pérez-Riveros, G. A. Duarte, M. L. Botero, A. Cárdenas and J. De la Hoz-Valle | 2019 | | A Randomized Placebo-Controlled <i>N</i>-of-1 Trial: The Effect of Proton Pump Inhibitor in the Management of Gastroesophageal Reflux Disease | Canadian Journal of Gastroenterology and Hepatology | | 10.1155/2019/3926051 |
| **472** | M. D. Skancke, R. A. Grossman, G. Marino, F. J. Brody and G. D. Trachiotis | 2017 | | Analysis of Minimally Invasive Esophagectomy at a Single Veterans Affairs Medical Center | Journal of Laparoendoscopic & Advanced Surgical Techniques | | 10.1089/lap.2017.0240 |
| **473** | I. Slavu, L. Alecu, A. Tulin, D. Mihaila, V. Braga, T. Voiosu, L. Tomescu and S. Constantinoiu | 2018 | | Reintervention Rate Following Emergency Surgery for Crohn Disease | Chirurgia | | 10.21614/chirurgia.113.2.227 |
| **474** | K. E. Smedby and M. Ponzoni | 2017 | | The aetiology of B-cell lymphoid malignancies with a focus on chronic inflammation and infections | Journal of Internal Medicine | | 10.1111/joim.12684 |
| **475** | K. K. Sogaard, D. K. Farkas, L. Pedersen, J. L. Lund, R. W. Thomsen and H. T. Sorensen | 2016 | | Long-term risk of gastrointestinal cancers in persons with gastric or duodenal ulcers | Cancer Medicine | | 10.1002/cam4.680 |
| **476** | M. Sohda, T. Yokobori, A. Kimura, T. Kosaka, K. Yoshida, K. Hakamada, S. Natsugoe, A. Taketomi, H. Saeki, K. Shirabe and H. Kuwano | 2023 | | Efficacy of chemotherapy for comorbid cancer in patients with simultaneous double cancers: a multicenter study | Surgery Today | | 10.1007/s00595-022-02542-3 |
| **477** | E. Y. Song, P. Venkat, M. Fradley, J. M. Frakes, F. Klocksieben, J. Fontaine, R. Mehta, S. Saeed, S. E. Hoffe and J. M. Pimiento | 2020 | | Clinical factors associated with the development of postoperative atrial fibrillation in esophageal cancer patients receiving multimodality therapy before surgery | Journal of Gastrointestinal Oncology | | 10.21037/jgo.2019.12.05 |
| **478** | H. Song, C. C. Abnet, Å. Andrén-Sandberg, A. K. Chaturvedi and W. M. Ye | 2016 | | Risk of Gastrointestinal Cancers among Patients with Appendectomy: A Large-Scale Swedish Register-Based Cohort Study during 1970-2009 | Plos One | | 10.1371/journal.pone.0151262 |
| **479** | H. Song, M. Held, S. Sandin, H. Rautelin, M. Eliasson, S. Söderberg, G. Hallmans, L. Engstrand, O. Nyrén and W. M. Ye | 2015 | | Increase in the Prevalence of Atrophic Gastritis Among Adults Age 35 to 44 Years Old in Northern Sweden Between 1990 and 2009 | Clinical Gastroenterology and Hepatology | | 10.1016/j.cgh.2015.04.001 |
| **480** | P. G. Song, M. M. Zha, W. Xia, C. X. Zeng and Y. J. Zhu | 2020 | | Asthma-chronic obstructive pulmonary disease overlap in China: prevalence, associated factors and comorbidities in middle-aged and older adults | Current Medical Research and Opinion | | 10.1080/03007995.2020.1722082 |
| **481** | S. Soutome, T. Hasegawa, T. Yamguchi, K. Aoki, N. Kanamura, T. Mukai, J. Yamazoe, M. Nishikawa, E. Isomura, K. Hoshi, M. Umeda, J. Y. Kogami, K. Sawaki, H. Nobuhara, S. Tachibana, Y. Kojima, M. Kubota, S. Baba, C. Yamauchi, K. Ono, M. Morioka, Y. Yamaguchi, T. Yamauchi, C. Nakajo, K. Tomihara, T. Yamamoto, I. Yamamori and O. Joint Res Comm Japanese Soc | 2020 | | Prevention of postoperative pneumonia by perioperative oral care in patients with esophageal cancer undergoing surgery: a multicenter retrospective study of 775 patients | Supportive Care in Cancer | | 10.1007/s00520-019-05242-w |
| **482** | N. Spantideas, E. Drosou, A. Bougea and D. Assimakopoulos | 2016 | | Gastroesophageal reflux disease symptoms in the Greek general population: prevalence and risk factors | Clinical and Experimental Gastroenterology | | 10.2147/ceg.S103485 |
| **483** | A. Spreafico, L. Coate, R. H. Zhai, W. Xu, Z. F. Chen, Z. Chen, D. Patel, B. Tse, M. C. Brown, R. S. Heist, L. Dodbiba, J. Teichman, M. Kulke, L. Su, L. Eng, J. Knox, R. Wong, G. E. Darling, D. C. Christiani and G. Liu | 2017 | | Early adulthood body mass index, cumulative smoking, and esophageal adenocarcinoma survival | Cancer Epidemiology | | 10.1016/j.canep.2016.11.009 |
| **484** | S. Srinivasan, S. Sundaram, K. F. Kennedy, D. R. Kohli, F. Emura, P. Sharma and M. Desai | 2022 | | Trends and predictors of 30-day readmissions in subjects with eosinophilic esophagitis: results from a national cohort | Diseases of the Esophagus | | 10.1093/dote/doab060 |
| **485** | N. Stabellini, A. K. Chandar, A. Chak, A. J. Barda, M. Dmukauskas, K. Waite and J. S. Barnholtz-Sloan | 2022 | | Sex differences in esophageal cancer overall and by histological subtype | Scientific Reports | | 10.1038/s41598-022-09193-x |
| **486** | K. Steenland, V. Barry, A. Anttila, M. Sallmen, W. Mueller, P. Ritchie, D. M. McElvenny and K. Straif | 2019 | | Cancer incidence among workers with blood lead measurements in two countries | Occupational and Environmental Medicine | | 10.1136/oemed-2019-105786 |
| **487** | J. Su, Y. C. Jiang, X. K. Fan, R. Tao, M. Wu, Y. Lu, Y. J. Hua, J. R. Jin, Y. Guo, J. Lv, P. Pei, Z. M. Chen, L. M. Li and J. Y. Zhou | 2022 | | Association between physical activity and cancer risk among Chinese adults: a 10-year prospective study | International Journal of Behavioral Nutrition and Physical Activity | | 10.1186/s12966-022-01390-1 |
| **488** | T. Sugase, K. Sugimura, T. Kanemura, T. Takeoka, M. Yamamoto, N. Shinno, H. Hara, T. Omori, M. Yasui and H. Miyata | 2023 | | Long-term changes in bone mineral density in postoperative patients with esophageal cancer | Annals of Gastroenterological Surgery | | 10.1002/ags3.12640 |
| **489** | C. Sun, H. Xu, S. X. Wang, K. Li, P. Z. Qin, B. H. Liang and L. Xu | 2023 | | Lifestyle, clinical and histological indices-based prediction models for survival in cancer patients: a city-wide prospective cohort study in China | Journal of Cancer Research and Clinical Oncology | | 10.1007/s00432-023-04888-8 |
| **490** | L. M. Sun, C. L. Lin, M. C. Lin, J. A. Liang and C. H. Kao | 2015 | | Radiotherapy- and Chemotherapy-Induced Myelodysplasia Syndrome <i>A Nationwide Population</i>-<i>Based Nested Case</i>-<i>Control Study</i> | Medicine | | 10.1097/md.0000000000000737 |
| **491** | L. P. Sun, H. K. Tu, J. W. Liu, Y. H. Gong, Q. Xu, J. J. Jing, N. N. Dong and Y. Yuan | 2014 | | A comprehensive evaluation of fasting serum gastrin-17 as a predictor of diseased stomach in Chinese population | Scandinavian Journal of Gastroenterology | | 10.3109/00365521.2014.950693 |
| **492** | P. Sun, C. Chen, F. Zhang, H. Yang, X. W. Bi, X. An, F. H. Wang and W. Q. Jiang | 2016 | | Combined heavy smoking and drinking predicts overall but not disease-free survival after curative resection of locoregional esophageal squamous cell carcinoma | Oncotargets and Therapy | | 10.2147/ott.S104182 |
| **493** | S. Y. Sun, P. P. Chen, L. X. Meng, L. Li, Z. X. Mo, C. H. Sun, Y. Wang and F. H. Liang | 2019 | | High preoperative plasma fibrinogen and serum albumin score is associated with poor survival in operable esophageal squamous cell carcinoma | Diseases of the Esophagus | | 10.1093/dote/doy057 |
| **494** | W. J. Sun, C. P. Wen, J. Lin, C. Wen, X. Pu, M. S. Huang, M. K. Tsai, C. K. Tsao, X. F. Wu and W. H. Chow | 2015 | | ABO blood types and cancer risk-A cohort study of 339,432 subjects in Taiwan | Cancer Epidemiology | | 10.1016/j.canep.2014.12.006 |
| **495** | H. Suzuki, J. Matsuzaki, T. Masaoka and J. M. Inadomi | 2014 | | Greater loss of productivity among Japanese workers with gastro-esophageal reflux disease (GERD) symptoms that persist vs resolve on medical therapy | Neurogastroenterology and Motility | | 10.1111/nmo.12319 |
| **496** | Y. Suzuki, T. Iizuka, A. Hosoi, D. Kikuchi, T. Okamura, Y. Ochiai, J. Hayasaka, N. O. Dan, Y. Mitsunaga, M. Tanaka, H. Odagiri, K. Nomura, S. Yamashita, A. Matsui and S. Hoteya | 2022 | | Clinicopathological Differences between Eosinophilic Esophagitis and Asymptomatic Esophageal Eosinophilia | Internal Medicine | | 10.2169/internalmedicine.8241-21 |
| **497** | S. Sweetser, N. L. Jacobs and L. Song | 2014 | | Endoscopic diagnosis and treatment of esophageal verrucous squamous cell cancer | Diseases of the Esophagus | | 10.1111/j.1442-2050.2012.01434.x |
| **498** | T. Tabuchi, K. Ozaki, A. Ioka and I. Miyashiro | 2015 | | Joint and independent effect of alcohol and tobacco use on the risk of subsequent cancer incidence among cancer survivors: A cohort study using cancer registries | International Journal of Cancer | | 10.1002/ijc.29575 |
| **499** | S. A. Taghavi, S. K. Majd, M. Sianati and M. Sepehrimanesh | 2016 | | Prevalence of IgG-4-associated cholangiopathy based on serum IgG-4 levels in patients with primary sclerosing cholangitis and its relationship with inflammatory bowel disease | Turkish Journal of Gastroenterology | | 10.5152/tjg.2016.16344 |
| **500** | E. W. Tai, G. P. Guy, C. B. Steele, S. J. Henley, M. S. Gallaway and L. C. Richardson | 2018 | | Cost of Tobacco-related Cancer Hospitalizations in the US, 2014 | American Journal of Preventive Medicine | | 10.1016/j.amepre.2017.12.004 |
| **501** | J. Takada, H. Araki, T. Mizutani, N. Ozawa, T. Sugiyama, M. Kubota, T. Ibuka and M. Shimizu | 2019 | | Safety of Carbon Dioxide Insufflation during Endoscopic Submucosal Dissection for Esophageal Squamous Cell Carcinoma | Digestive Diseases | | 10.1159/000492870 |
| **502** | S. Takahashi, T. Matsumura, T. Kaneko, M. Tokunaga, H. Oura, T. Ishikawa, A. Nagashima, W. Shiratori, N. Akizue, Y. Ohta, A. Kikuchi, M. Fujie, K. Saito, K. Okimoto, D. Maruoka, T. Nakagawa, M. Arai, J. Kato and N. Kato | 2021 | | Clinical Characteristics of Esophageal Motility Disorders in Patients With Heartburn | Journal of Neurogastroenterology and Motility | | 10.5056/jnm20131 |
| **503** | Y. Takahashi, N. Nagata, T. Shimbo, T. Nishijima, K. Watanabe, T. Aoki, K. Sekine, H. Okubo, K. Watanabe, T. Sakurai, C. Yokoi, M. Kobayakawa, H. Yazaki, K. Teruya, H. Gatanaga, Y. Kikuchi, S. Mine, T. Igari, Y. Takahashi, A. Mimori, S. Oka, J. Akiyama and N. Uemura | 2015 | | Long-Term Trends in Esophageal Candidiasis Prevalence and Associated Risk Factors with or without HIV Infection: Lessons from an Endoscopic Study of 80,219 Patients | Plos One | | 10.1371/journal.pone.0133589 |
| **504** | Y. Takenaka, N. Takemoto, R. Oya, N. Ashida, T. Kitamura, K. Shimizu, K. Takemura, T. Michiba, A. Hanamoto, M. Suzuki, Y. Yamamoto, A. Uno and H. Inohara | 2017 | | Development and validation of a new comorbidity index for patients with head and neck squamous cell carcinoma in Japan | Scientific Reports | | 10.1038/s41598-017-07752-1 |
| **505** | T. Takeshita, K. Matsumoto, M. Furuta, S. Fukuyama, K. Takeuchi, H. Ogata, M. Asakawa, S. Kageyama, J. Hata, T. Ninomiya, H. Inoue and Y. Yamashita | 2021 | | Airflow limitation and tongue microbiota in community-dwelling elderly individuals | Erj Open Research | | 10.1183/23120541.00616-2020 |
| **506** | H. Takeuchi, H. Miyata, M. Gotoh, Y. Kitagawa, H. Baba, W. Kimura, N. Tomita, T. Nakagoe, M. Shimada, K. Sugihara and M. Mori | 2014 | | A Risk Model for Esophagectomy Using Data of 5354 Patients Included in a Japanese Nationwide Web-Based Database | Annals of Surgery | | 10.1097/sla.0000000000000644 |
| **507** | Z. H. Tan, H. Yang, J. Wen, K. J. Luo, Q. W. Liu, Y. H. Hu, L. J. Zhang, M. Z. Liu, J. P. Yun and J. H. Fu | 2018 | | Clinical predictors of pathologically response after neoadjuvant chemoradiotherapy for esophageal squamous cell carcinoma: long term outcomes of a phase II study | Journal of Thoracic Disease | | 10.21037/jtd.2018.08.88 |
| **508** | H. Tanaka, T. Takeuchi, S. Nishida, H. Hongo, M. Takii, T. Higashino, M. Sanomura, H. Miyazaki, M. Hoshimoto, T. Kimura, M. Sakaguchi, T. Abe, A. Hakoda, N. Sugawara, T. Iwatsubo, S. Kawaguchi, K. Ota, Y. Kojima and K. Higuchi | 2023 | | Examination on Factors Affecting Symptom Change after Drug Withdrawal in Patients with Mild Erosive Gastroesophageal Reflux Disease Undergoing Symptom-Controlled Maintenance Therapy with Acid-Secretion Inhibition Drugs | Digestion | | 10.1159/000528418 |
| **509** | J. Tang, J. Z. Zhao, K. M. Ren, F. S. Zheng, X. W. Wang, H. J. Liu, J. G. Zhao and J. B. Lu | 2019 | | Risk factors of atrial fibrillation occurring after radical surgery of esophageal carcinoma | Journal of Cardiothoracic Surgery | | 10.1186/s13019-019-0885-z |
| **510** | Y. X. Tang, W. J. Zhao, J. Li, P. Xie, S. Y. Wang, L. B. Yan, X. B. Xing, J. H. Lu, L. A. Tse, H. H. X. Wang and X. D. Liu | 2022 | | Dietary intake of monounsaturated and polyunsaturated fatty acids is related to the reduced risk of esophageal squamous cell carcinoma | Lipids in Health and Disease | | 10.1186/s12944-022-01624-y |
| **511** | C. Taniguchi, A. Narisada, H. Ando, A. Hashimoto, A. Nakayama, M. Ito, H. Tanaka and K. Suzuki | 2023 | | Smoking cessation behavior in patients with a diagnosis of a non-communicable disease: The impact of perceived disease severity of and susceptibility to the disease | Tobacco Induced Diseases | | 10.18332/tid/170430 |
| **512** | H. Teragawa, C. Oshita and T. Ueda | 2019 | | History of gastroesophageal reflux disease in patients with suspected coronary artery disease | Heart and Vessels | | 10.1007/s00380-019-01413-1 |
| **513** | M. Thomas, G. Defraene, M. Lambrecht, W. Deng, J. Moons, P. Nafteux, S. H. Lin and K. Haustermans | 2019 | | NTCP model for postoperative complications and one-year mortality after trimodality treatment in oesophageal cancer | Radiotherapy and Oncology | | 10.1016/j.radonc.2019.09.015 |
| **514** | S. J. Thomas, L. Almers, J. Schneider, J. E. Graham, P. J. Havel and D. A. Corley | 2016 | | Ghrelin and Leptin Have a Complex Relationship with Risk of Barrett's Esophagus | Digestive Diseases and Sciences | | 10.1007/s10620-015-3867-6 |
| **515** | A. P. Thrift, J. R. Kramer, C. M. Hartman, K. Royse, P. Richardson, Y. Q. Dong, S. Raychaudhury, R. Desiderio, D. Sanchez, S. Anandasabapathy, D. L. White and E. Y. Chiao | 2019 | | Risk and Predictors of Esophageal and Stomach Cancers in HIV-Infected Veterans: A Matched Cohort Study | Jaids-Journal of Acquired Immune Deficiency Syndromes | | 10.1097/qai.0000000000002038 |
| **516** | A. P. Thrift, N. J. Shaheen, M. D. Gammon, L. Bernstein, B. J. Reid, L. Onstad, H. A. Risch, G. Liu, N. C. Bird, A. H. Wu, D. A. Corley, Y. Romero, S. J. Chanock, W. H. Chow, A. G. Casson, D. M. Levine, R. Zhang, W. E. Ek, S. MacGregor, W. M. Ye, L. J. Hardie, T. L. Vaughan and D. C. Whiteman | 2014 | | Obesity and Risk of Esophageal Adenocarcinoma and Barrett's Esophagus: A Mendelian Randomization Study | Jnci-Journal of the National Cancer Institute | | 10.1093/jnci/dju252 |
| **517** | R. Tian, H. Yan, F. Zhang, P. Sun, A. R. Wu, M. Zhang, Y. L. Jiang, J. Wu, Y. H. Lu, Q. Y. Xu, X. H. Zhan, R. X. Zhang, L. T. Qian and J. He | 2016 | | Cumulative score based on preoperative plasma fibrinogen and serum C-reactive protein could predict long-term survival for esophageal squamous cell carcinoma | Oncotarget | | 10.18632/oncotarget.11145 |
| **518** | S. C. Tinker, J. Reefhuis, R. H. Bitsko, S. M. Gilboa, A. A. Mitchell, E. L. Tran, M. M. Werler, C. S. Broussard and S. Natl Birth Defects Prevention | 2019 | | Use of benzodiazepine medications during pregnancy and potential risk for birth defects, National Birth Defects Prevention Study, 1997-2011 | Birth Defects Research | | 10.1002/bdr2.1497 |
| **519** | G. Tomisa, A. Horváth, Z. Szalai, V. Müller and L. Tamási | 2019 | | Prevalence and impact of risk factors for poor asthma outcomes in a large, specialist-managed patient cohort: a real-life study | Journal of Asthma and Allergy | | 10.2147/jaa.S211246 |
| **520** | L. A. Torre, A. M. G. Sauer, M. S. Chen, M. Kagawa-Singer, A. Jemal and R. L. Siegel | 2016 | | Cancer Statistics for Asian Americans, Native Hawaiians, and Pacific Islanders, 2016: Converging Incidence in Males and Females | Ca-a Cancer Journal for Clinicians | | 10.3322/caac.21335 |
| **521** | A. N. Townsend, A. Denton, N. Gohel, J. Lozano, P. R. de la Vega, G. Castro and R. Seetharamaiah | 2023 | | An Association Between Comorbidities and Postsurgical Complications in Adults Who Underwent Esophagectomy | Cureus Journal of Medical Science | | 10.7759/cureus.36395 |
| **522** | C. L. Tran, M. Han, B. Kim, E. Y. Park, Y. I. Kim and J. K. Oh | 2023 | | Gastroesophageal reflux disease and risk of cancer: Findings from the Korean National Health Screening Cohort | Cancer Medicine | | 10.1002/cam4.6500 |
| **523** | M. C. Tsai, Y. C. Chou, Y. K. Lee, W. L. Hsu, C. S. Tang, S. Y. Chen, S. P. Huang, Y. C. Chen and J. M. Lee | 2022 | | Secular Trends in Incidence of Esophageal Cancer in Taiwan from 1985 to 2019: An Age-Period-Cohort Analysis | Cancers | | 10.3390/cancers14235844 |
| **524** | S. C. Tseng, T. Hino, H. Hatabu, H. Park, N. N. Sanford, G. G. Lin, M. Nishino and H. Mamon | 2022 | | Interstitial Lung Abnormalities in Patients With Locally Advanced Esophageal Cancer: Prevalence, Risk Factors, and Clinical Implications | Journal of Computer Assisted Tomography | | 10.1097/rct.0000000000001366 |
| **525** | Y. A. Tseng, Y. L. Ou, J. H. Geng, C. W. Wang, D. W. Wu, S. C. Chen and P. L. Lu | 2023 | | The association between alcohol, betel nut, and cigarette use with hepatitis C virus infection in Taiwan | Scientific Reports | | 10.1038/s41598-023-50588-1 |
| **526** | P. Tshering, S. Dorjee, T. Dendup, T. Dorji and D. Wangmo | 2020 | | Epidemiological and histopathological characteristics of head and neck cancers in Bhutan from 2011 to 2017: a retrospective descriptive study | Ecancermedicalscience | | 10.3332/ecancer.2020.1024 |
| **527** | G. K. Turdumambetova, A. Osmanov and D. W. Denning | 2019 | | The Burden of Serious Fungal Infections in Kyrgyzstan | Journal of Fungi | | 10.3390/jof5030066 |
| **528** | F. P. Turkoz, M. Solak, S. Kilickap, A. Ulas, O. Esbah, B. Oksuzoglu and S. Yalcin | 2014 | | Bone Metastasis from Gastric Cancer: The Incidence, Clinicopathological Features, and Influence on Survival | Journal of Gastric Cancer | | 10.5230/jgc.2014.14.3.164 |
| **529** | M. C. Turner, D. Krewski, W. R. Diver, C. A. Pope, R. T. Burnett, M. Jerrett, J. D. Marshall and S. M. Gapstur | 2017 | | Ambient Air Pollution and Cancer Mortality in the Cancer Prevention Study II | Environmental Health Perspectives | | 10.1289/ehp1249 |
| **530** | S. Tursun, Y. Sahin, M. E. Alçigir, M. Çínar and I. Karahan | 2024 | | Cafeteria diet can cause systemic inflammation and oxidative damage in the various tissues | Mediterranean Journal of Nutrition and Metabolism | | 10.3233/mnm-230068 |
| **531** | M. Uguen, J. D. Dewitte, P. Marcorelles, B. Loddé, R. Pougnet, P. Saliou, M. De Braekeleer and A. Uguen | 2017 | | Asbestos-related lung cancers: A retrospective clinical and pathological study | Molecular and Clinical Oncology | | 10.3892/mco.2017.1277 |
| **532** | O. Ugwuegbu, F. Shibli, Y. Kim, V. Rangan, M. Kurin, F. Ayoub, S. Ganocy, R. Kavitt and R. Fass | 2024 | | The Impact of Chronic Cannabis Use on Esophageal Motility in Patients Referred for Esophageal Manometry | Journal of Clinical Gastroenterology | | 10.1097/mcg.0000000000001887 |
| **533** | J. M. Underwood, T. B. Richards, S. J. Henley, B. Momin, K. Houston, I. Rolle, C. Holmes and S. L. Stewart | 2015 | | Decreasing Trend in Tobacco-Related Cancer Incidence, United States 2005-2009 | Journal of Community Health | | 10.1007/s10900-014-9951-6 |
| **534** | A. J. Usher-Smith, C. Häggström, P. Wennberg, K. Lindvall, J. Strelitz, J. S. Sharp and J. S. Griffin | 2021 | | Impact of achievement and change in achievement of lifestyle recommendations in middle-age on risk of the most common potentially preventable cancers | Preventive Medicine | | 10.1016/j.ypmed.2021.106712 |
| **535** | D. P. Vageli, P. G. Doukas, S. G. Doukas, A. Tsatsakis and B. L. Judson | 2022 | | Noxious Combination of Tobacco Smoke Nitrosamines with Bile, Deoxycholic Acid, Promotes Hypopharyngeal Squamous Cell Carcinoma, via NFκB, <i>In Vivo</i> | Cancer Prevention Research | | 10.1158/1940-6207.Capr-21-0529 |
| **536** | A. T. Valappil, A. A. Mehta, A. Kunoor and N. Haridas | 2018 | | Spectrum of diffuse parenchymal lung diseases: An Experience from A Tertiary Care Referral Centre From South India | Egyptian Journal of Chest Diseases and Tuberculosis | | 10.4103/ejcdt.ejcdt_52_18 |
| **537** | M. van Gelder, A. R. T. Donders, O. Devine, N. Roeleveld, J. Reefhuis and S. Natl Birth Defects Prevention | 2014 | | Using Bayesian Models to Assess the Effects of Under-reporting of Cannabis Use on the Association with Birth Defects, National Birth Defects Prevention Study, 1997-2005 | Paediatric and Perinatal Epidemiology | | 10.1111/ppe.12140 |
| **538** | H. S. van Monsjou, M. Schaapveld, O. Hamming-Vrieze, J. P. de Boer, M. W. M. van den Brekel and A. J. M. Balm | 2016 | | Cause-specific excess mortality in patients treated for cancer of the oral cavity and oropharynx: A population-based study | Oral Oncology | | 10.1016/j.oraloncology.2015.10.013 |
| **539** | Y. Veisani and A. Delpisheh | 2017 | | Late Diagnosis, Smoking History and Socioeconomic Inequality in Gastric Carcinoma: A Decomposition Approach | Iranian Journal of Cancer Prevention | | 10.5812/ijcm.3754 |
| **540** | F. W. T. Vergouwe, H. Ijsselstijn, K. Biermann, N. S. Erler, R. M. H. Wijnen, M. J. Bruno and M. C. W. Spaander | 2018 | | High Prevalence of Barrett's Esophagus and Esophageal Squamous Cell Carcinoma After Repair of Esophageal Atresia | Clinical Gastroenterology and Hepatology | | 10.1016/j.cgh.2017.11.008 |
| **541** | A. S. M. Vernon, J. S. Hoch, L. Fejerman and T. H. Keegan | 2024 | | Cancer incidence among Armenians in California | Cancer Medicine | | 10.1002/cam4.7100 |
| **542** | E. Vogtmann, R. Flores, G. Q. Yu, N. D. Freedman, J. X. Shi, M. H. Gail, B. A. Dye, G. Q. Wang, V. Klepac-Ceraj, B. J. Paster, W. Q. Wei, H. Q. Guo, S. M. Dawsey, Y. L. Qiao and C. C. Abnet | 2015 | | Association between tobacco use and the upper gastrointestinal microbiome among Chinese men | Cancer Causes & Control | | 10.1007/s10552-015-0535-2 |
| **543** | S. Y. Wan, D. Pan, M. Su, S. K. Wang, Y. Y. Wang, D. F. Xu, J. H. Sun, W. Xie, X. Wang, Q. Y. Yan, H. Xia, C. Yang and G. J. Sun | 2024 | | Association between socio-demographic factors, lifestyle, eating habits and hypertension risk among middle-aged and older rural Chinese adults | Nutrition Metabolism and Cardiovascular Diseases | | 10.1016/j.numecd.2023.11.012 |
| **544** | C. B. Wang, Q. X. Fu, H. Y. Liu and R. Wang | 2015 | | Fish consumption doesn't reduce the risk of hepatocellular carcinoma | International Journal of Clinical and Experimental Medicine | |  |
| **545** | G. H. Wang, X. Y. Xu, Q. Q. Cheng, J. M. Hu, X. Y. Xu, Y. W. Zhang, S. Guo, Y. C. Ji, C. G. Zhou, F. Gao, L. Yang, Y. X. Liu, S. Y. Yin and C. Y. Su | 2023 | | Preparation of sustainable release mesoporous silica nano-pesticide for control of Monochamus alternatus | Sustainable Materials and Technologies | | 10.1016/j.susmat.2022.e00538 |
| **546** | H. Wang, Y. Q. Pan, C. H. Guo, F. L. Li, R. P. Xu, M. F. Liu, Z. Liu, F. F. Liu, H. Cai, Y. Ke and Z. H. He | 2018 | | Health-related quality of life among rural residents aged 45-69 years in Hua County, Henan Province, China: Results of ESECC Trial for esophageal cancer screening with endoscopy | Chinese Journal of Cancer Research | | 10.21147/j.issn.1000-9604.2018.02.07 |
| **547** | H. W. Wang, C. J. Kuo, W. R. Lin, C. M. Hsu, Y. P. Ho, C. J. Lin, K. H. Chen, M. Y. Su and C. T. Chiu | 2015 | | Barrett's esophagus and risk of esophageal adenocarcinoma: A retrospective analysis | Advances in Digestive Medicine | | 10.1016/j.aidm.2014.06.011 |
| **548** | H. W. Wang, C. J. Kuo, W. R. Lin, C. M. Hsu, Y. P. Ho, C. J. Lin, M. Y. Su, C. T. Chiu, C. L. Wang and K. H. Chen | 2016 | | The clinical characteristics and manifestations of cytomegalovirus esophagitis | Diseases of the Esophagus | | 10.1111/dote.12340 |
| **549** | J. Wang, S. N. Gao, Y. J. Wang, J. J. Zhou, J. Lin, L. Wang and Y. Du | 2018 | | Cancer incidence and mortality patterns in Luwan district of Shanghai during 2002-2011 | Drug Discoveries and Therapeutics | | 10.5582/ddt.2018.01009 |
| **550** | N. N. Wang, B. X. Tan, F. L. Cao, Q. X. Song, J. B. Wang, Y. B. Jia and Y. F. Cheng | 2015 | | Prognostic influence of smoking on esophageal squamous cell carcinoma | International Journal of Clinical and Experimental Medicine | |  |
| **551** | Q. Wang and Z. M. Yang | 2018 | | Does chronic disease influence susceptibility to the effects of air pollution on depressive symptoms in China? | International Journal of Mental Health Systems | | 10.1186/s13033-018-0212-4 |
| **552** | S. M. Wang, N. D. Freedman, H. A. Katki, C. Matthews, B. I. Graubard, L. L. Kahle and C. C. Abnet | 2021 | | Gastroesophageal reflux disease: A risk factor for laryngeal squamous cell carcinoma and esophageal squamous cell carcinoma in the NIH-AARP Diet and Health Study cohort | Cancer | | 10.1002/cncr.33427 |
| **553** | T. F. Wang, R. Zreik and B. Leng | 2023 | | The Landscape of Primary Gastric Leiomyosarcoma in Texas Population: Analysis of Texas Cancer Registry Data | Cureus Journal of Medical Science | | 10.7759/cureus.49403 |
| **554** | X. L. Wang, Z. Wright, J. Y. Wang, S. Roy, R. Fass and G. Q. Song | 2023 | | Elucidating the Link: Chronic Obstructive Pulmonary Disease and the Complex Interplay of Gastroesophageal Reflux Disease and Reflux-Related Complications | Medicina-Lithuania | | 10.3390/medicina59071270 |
| **555** | X. X. Wang, Y. Peng, F. B. Liu, P. Wang, C. Y. Si, J. X. Gong, H. J. Zhou, M. Zhang and F. F. Song | 2024 | | Joint association of biological aging and lifestyle with risks of cancer incidence and mortality: A cohort study in the UK Biobank | Preventive Medicine | | 10.1016/j.ypmed.2024.107928 |
| **556** | Y. Wang, J. Wang and X. F. Yan | 2023 | | Analysis of the factors influencing chronic diseases in middle-aged and elderly men: a cross-sectional survey in China | Journal of Mens Health | | 10.22514/jomh.2023.050 |
| **557** | Y. Wang, G. Xu, J. Wang, X. H. Li, P. Sun, W. Zhang, J. X. Li and C. Y. Wu | 2017 | | Relationship of Th17/Treg Cells and Radiation Pneumonia in Locally Advanced Esophageal Carcinoma | Anticancer Research | | 10.21873/anticanres.11866 |
| **558** | Z. Wang, N. J. Shaheen, D. C. Whiteman, L. A. Anderson, T. L. Vaughan, D. A. Corley, H. B. El-Serag, J. H. Rubenstein and A. P. Thrift | 2018 | | <i>Helicobacter pylori</i> Infection Is Associated With Reduced Risk of Barrett's Esophagus: An Analysis of the Barrett's and Esophageal Adenocarcinoma Consortium | American Journal of Gastroenterology | | 10.1038/s41395-018-0070-3 |
| **559** | Z. G. Wang, Z. W. Hu, J. M. Wu, F. Ji, H. T. Wang, Y. G. Lai, X. Gao, Y. C. Ning, C. C. Zhang, Z. T. Li, W. T. Liang and J. J. Liu | 2015 | | Insult of gastroesophageal reflux on airway: clinical significance of pharyngeal nozzle | Frontiers of Medicine | | 10.1007/s11684-014-0343-1 |
| **560** | Z. S. Wang, W. P. Koh, A. Z. Jin, R. W. Wang and J. M. Yuan | 2017 | | Composite protective lifestyle factors and risk of developing gastric adenocarcinoma: the Singapore Chinese Health Study | British Journal of Cancer | | 10.1038/bjc.2017.7 |
| **561** | S. Q. Wani, T. Khan, S. Y. Wani, L. R. Mir, M. M. Lone, T. R. Malik, A. M. Najmi, F. Afroz, M. A. Teli and N. A. Khan | 2016 | | Nasopharyngeal Carcinoma: A 15 Year Study with Respect to Clinicodemography and Survival Analysis | Indian Journal of Otolaryngology and Head & Neck Surgery | | 10.1007/s12070-016-1018-9 |
| **562** | M. M. Ward and S. Alehashemi | 2020 | | Risks of solid cancers in elderly persons with osteoarthritis or ankylosing spondylitis | Rheumatology | | 10.1093/rheumatology/keaa166 |
| **563** | E. T. Warner, E. R. Park, C. M. Luberto, J. Rabin, G. K. Perez and J. S. Ostroff | 2022 | | Internalized stigma among cancer patients enrolled in a smoking cessation trial: The role of cancer type and associations with psychological distress | Psycho-Oncology | | 10.1002/pon.5859 |
| **564** | R. Watkins, G. A. Soliman, J. Mwaiselage, C. Kahesa, K. Msami and M. L. Wilson | 2022 | | Distance to Health Care Facilities, Lifestyle Risk Factors, and Stage at Diagnosis in relation to Geographic Pattern of Esophageal Cancer in Tanzania, 2006-2016 | Journal of Cancer Epidemiology | | 10.1155/2022/7873588 |
| **565** | A. Wéber, M. Laversanne, P. Nagy, I. Kenessey, I. Soerjomataram and F. Bray | 2023 | | Gains in life expectancy from decreasing cardiovascular disease and cancer mortality - an analysis of 28 european countries 1995-2019 | European Journal of Epidemiology | | 10.1007/s10654-023-01039-8 |
| **566** | R. Wedekind, P. Keski-Rahkonen, N. Robinot, V. Viallon, P. Ferrari, E. Engel, M. C. Boutron-Ruault, Y. Mahamat-Saleh, F. R. Mancini, T. Kühn, T. Johnson, H. Boeing, M. Bergmann, A. Karakatsani, A. Trichopoulou, H. Peppa, C. Agnoli, M. S. de Magistris, D. Palli, C. Sacerdote, R. Tumino, M. J. Gunter, I. Huybrechts and A. Scalbert | 2019 | | Syringol metabolites as new biomarkers for smoked meat intake | American Journal of Clinical Nutrition | | 10.1093/ajcn/nqz222 |
| **567** | Z. H. Wei, Z. G. Ren, S. Hu, Y. Gao, R. R. Sun, S. Lv, G. J. Yang, Z. J. Yu and Q. C. Kan | 2020 | | Development and validation of a simple risk model to predict major cancers for patients with nonalcoholic fatty liver disease | Cancer Medicine | | 10.1002/cam4.2777 |
| **568** | S. Wernly, V. Paar, A. Völkerer, G. Semmler, C. Datz, M. Lichtenauer and B. Wernly | 2023 | | sST2 Levels Show No Association with <i>Helicobacter pylori</i> Infection in Asymptomatic Patients: Implications for Biomarker Research | Digestive Diseases and Sciences | | 10.1007/s10620-023-08005-0 |
| **569** | D. P. Wickramasinghe and D. N. Samarasekera | 2017 | | Incidence of esophageal cancer in Sri Lanka: Analysis of cancer registry data and comparison with other South Asian populations | Asia-Pacific Journal of Clinical Oncology | | 10.1111/ajco.12481 |
| **570** | A. M. Wood, H. Jonsson, G. Nagel, C. Häggström, J. Manjer, H. Ulmer, A. Engeland, E. Zitt, S. H. J. Jochems, S. Ghaderi, P. Stattin, T. Bjorge and T. Stocks | 2021 | | The Inverse Association of Body Mass Index with Lung Cancer: Exploring Residual Confounding, Metabolic Aberrations and Within-Person Variability in Smoking | Cancer Epidemiology Biomarkers & Prevention | | 10.1158/1055-9965.Epi-21-0058 |
| **571** | H. Wu and B. Shen | 2021 | | Endoscopic and histologic evaluation of the gastrointestinal tract in patients with sarcoidosis | European Journal of Gastroenterology & Hepatology | | 10.1097/meg.0000000000001943 |
| **572** | Y. Wu, Y. P. Li and E. Giovannucci | 2021 | | Potential Impact of Time Trend of Lifestyle Risk Factors on Burden of Major Gastrointestinal Cancers in China | Gastroenterology | | 10.1053/j.gastro.2021.08.006 |
| **573** | X. J. Xi, Z. L. Fu, T. W. Liu, Y. F. Lin, W. B. Wu, J. M. Li, M. Luo and B. P. Zhang | 2021 | | Establishment and Verification of Scoring System for Colorectal Adenoma Recurrence | Risk Management and Healthcare Policy | | 10.2147/rmhp.S316408 |
| **574** | S. H. Xie and J. Lagergren | 2016 | | A model for predicting individuals' absolute risk of esophageal adenocarcinoma: Moving toward tailored screening and prevention | International Journal of Cancer | | 10.1002/ijc.29988 |
| **575** | S. H. Xie, E. Ness-Jensen, H. Langseth, R. E. Gislefoss, F. Mattsson and J. Lagergren | 2021 | | Prediagnostic circulating levels of sex hormones and survival in esophageal adenocarcinoma | International Journal of Cancer | | 10.1002/ijc.33285 |
| **576** | C. R. Xu, H. Lin, J. Su, X. C. Zhang, Y. S. Huang, X. N. Yang, Q. Zhou, J. J. Yang, W. Z. Zhong and Y. L. Wu | 2019 | | Familial association of lung cancer with liver cancer in first-degree relatives | Cancer Management and Research | | 10.2147/cmar.S199462 |
| **577** | S. J. Xu, L. Q. Lin, C. Chen, T. Y. Chen, C. X. You, R. Q. Chen, C. Deana, C. J. Wakefield, J. B. Shrager, D. Molena, C. F. J. Yang, J. H. Lin and S. C. Chen | 2022 | | Textbook outcome after minimally invasive esophagectomy is an important prognostic indicator for predicting long-term oncological outcomes with locally advanced esophageal squamous cell carcinoma | Annals of Translational Medicine | | 10.21037/atm-22-506 |
| **578** | X. Xu, X. Zhang, J. Lu, J. X. Dai, R. Q. Lin, F. X. Tian, B. Liang, Y. N. Guo, H. Y. Luo, N. Li, D. P. Fang, R. H. Zhao and C. M. Huang | 2016 | | The Effects of Dinner-to-Bed Time and Post-Dinner Walk on Gastric Cancer Across Different Age Groups <i>A Multicenter Case</i>-<i>Control Study in Southeast China</i> | Medicine | | 10.1097/md.0000000000003397 |
| **579** | Y. P. Xu, Y. D. Zheng, X. J. Sun, X. M. Yu, J. L. Gu, W. Wu, G. Zhang, J. L. Hu, W. Y. Sun and W. M. Mao | 2015 | | Concurrent radiotherapy with gefitinib in elderly patients with esophageal squamous cell carcinoma: Preliminary results of a phase II study | Oncotarget | | 10.18632/oncotarget.5193 |
| **580** | Y. W. Xu, A. Miremadi, A. Link, P. Malfertheiner, R. C. Fitzgerald and J. Bornschein | 2019 | | Feasibility of combined screening for upper gastrointestinal adenocarcinoma risk by serology and Cytosponge testing: the SUGAR study | Journal of Clinical Pathology | | 10.1136/jclinpath-2019-205700 |
| **581** | A. Yago, Y. Ohkura, M. Ueno, K. Fujisawa, Y. Ogawa, H. Shimoyama, S. Haruta and H. Udagawa | 2022 | | Importance of long-term surveillance after curative esophagectomy for esophageal squamous cell carcinoma | Diseases of the Esophagus | | 10.1093/dote/doab098 |
| **582** | J. Yakoob, S. S. Fatima, Z. Abbas, S. F. Mustafa, H. A. R. Khan, M. F. Raghib, S. Awan and Z. Ahmad | 2017 | | Distribution of gastric carcinoma in an area with a high prevalence of <i>Helicobacter pylori</i> | Turkish Journal of Gastroenterology | | 10.5152/tjg.2017.17607 |
| **583** | T. Yamaguchi, K. Kato, K. Nagashima, S. Iwasa, Y. Honma, A. Takashima, T. Hamaguchi, Y. Ito, J. Itami, N. Boku and K. Higuchi | 2018 | | Type of second primary malignancy after achieving complete response by definitive chemoradiation therapy in patients with esophageal squamous cell carcinoma | International Journal of Clinical Oncology | | 10.1007/s10147-018-1258-7 |
| **584** | Y. Yamaji, H. Yasunaga, Y. Hirata, A. Yamada, S. Yoshida, H. Horiguchi, K. Fushimi and K. Koike | 2016 | | Association Between Colorectal Cancer and Atherosclerotic Diseases: A Study Using a National Inpatient Database in Japan | Digestive Diseases and Sciences | | 10.1007/s10620-016-4041-5 |
| **585** | L. H. Yan, Z. Y. Shan, Y. Sun, Y. Yan and Z. Q. Lu | 2014 | | Association between esophageal cancer in middle-aged and elderly patients and body mass index and waist-to-hip ratio | Family Medicine and Community Health | | 10.15212/fmch.2014.0127 |
| **586** | M. Yanes, G. Santoni, J. Maret-Ouda, E. Ness-Jensen, M. Färkkilä, E. Lynge, E. Pukkala, P. Romundstad, L. Tryggvadóttir, M. von Euler-Chelpin and J. Lagergren | 2022 | | Laryngeal and Pharyngeal Squamous Cell Carcinoma After Antireflux Surgery in the 5 Nordic Countries | Annals of Surgery | | 10.1097/sla.0000000000004423 |
| **587** | C. K. Yang, A. Teng, D. Y. Lee and K. Rose | 2015 | | Pulmonary complications after major abdominal surgery: National Surgical Quality Improvement Program analysis | Journal of Surgical Research | | 10.1016/j.jss.2015.03.028 |
| **588** | F. Yang, S. Zhang, H. Yang, K. Luo, J. Wen, Y. Hu, R. Hu, Q. Huang, J. Chen and J. Fu | 2015 | | Prognostic significance of gamma-glutamyltransferase in patients with resectable esophageal squamous cell carcinoma | Diseases of the Esophagus | | 10.1111/dote.12227 |
| **589** | H. Y. Yang, S. H. Huang, R. H. Shie and P. C. Chen | 2016 | | Cancer mortality in a population exposed to nephrite processing | Occupational and Environmental Medicine | | 10.1136/oemed-2016-103586 |
| **590** | J. Yang, L. Zhao, N. Zhang, Z. H. Du, Y. Y. Li, X. Li, D. L. Zhao and J. L. Wang | 2021 | | Cancer death and potential years of life lost in Feicheng City, China Trends from 2013 to 2018 | Medicine | | 10.1097/md.0000000000027370 |
| **591** | X. R. Yang, Y. C. Ni, Z. Y. Yuan, H. Chen, A. Plymoth, L. Jin, X. D. Chen, M. Lu and W. M. Ye | 2018 | | Very hot tea drinking increases esophageal squamous cell carcinoma risk in a high-risk area of China: a population-based case-control study | Clinical Epidemiology | | 10.2147/clep.S171615 |
| **592** | Y. Yang, X. F. Xu, X. Zhou, W. A. Bao, D. H. Zhang, F. Y. Gu, X. H. Du, Q. X. Chen and G. Q. Qiu | 2020 | | Impact of Radiation Dose on Survival for Esophageal Squamous Cell Carcinoma Treated With Neoadjuvant Chemoradiotherapy | Frontiers in Oncology | | 10.3389/fonc.2020.01431 |
| **593** | Y. Yano, C. C. Abnet, G. Roshandel, A. Graf, H. Poustchi, M. Khoshnia, A. Pourshams, F. Kamangar, P. Boffetta, P. Brennan, S. M. Dawsey, E. Vogtmann, R. Malekzadeh and A. Etemadi | 2024 | | Dental health and lung cancer risk in the Golestan Cohort Study | Bmc Cancer | | 10.1186/s12885-024-11850-5 |
| **594** | S. S. Yao, G. Y. Cao, L. Han, Z. S. Chen, Z. T. Huang, P. Gong, Y. H. Hu and B. B. Xu | 2020 | | Prevalence and Patterns of Multimorbidity in a Nationally Representative Sample of Older Chinese: Results From the China Health and Retirement Longitudinal Study | Journals of Gerontology Series a-Biological Sciences and Medical Sciences | | 10.1093/gerona/glz185 |
| **595** | Y. C. Yen, J. H. Chang, W. C. Lin, J. F. Chiou, Y. C. Chang, C. L. Chang, H. L. Hsu, J. M. Chow, K. S. P. Yuan, A. T. H. Wu and S. Y. Wu | 2017 | | Effectiveness of Esophagectomy in Patients With Thoracic Esophageal Squamous Cell Carcinoma Receiving Definitive Radiotherapy or Concurrent Chemoradiotherapy Through Intensity-Modulated Radiation Therapy Techniques | Cancer | | 10.1002/cncr.30565 |
| **596** | X. L. Yin, X. R. Yang, T. C. Zhang, Z. Y. Yuan, H. Chen, L. Jin, X. D. Chen, M. Lu and W. M. Ye | 2021 | | Changes of Body Mass Index and Body Shape in relation to risk of Gastric Cancer: A population-based case-control study | Journal of Cancer | | 10.7150/jca.56149 |
| **597** | M. Yoosefian, A. Pakpour and M. Zahedi | 2020 | | Carboxylated single-walled carbon nanotubes as a semiconductor for adsorption of acrylamide in mainstream cigarette smoke | Physica E-Low-Dimensional Systems & Nanostructures | | 10.1016/j.physe.2020.114299 |
| **598** | N. Yoshida, Y. Tamaoki, Y. Baba, Y. Sakamoto, Y. Miyamoto, M. Iwatsuki, T. Shono, H. Miyamoto, M. Imuta, J. Kurashige, H. Sawayama, R. Tokunaga, M. Watanabe, Y. Sasaki, Y. Yamashita and H. Baba | 2016 | | Incidence and risk factors of synchronous colorectal cancer in patients with esophageal cancer: an analysis of 480 consecutive colonoscopies before surgery | International Journal of Clinical Oncology | | 10.1007/s10147-016-1015-8 |
| **599** | C. L. Yu, Z. T. Zhang, Y. G. Liu, Y. Zong, Y. C. Chen, X. M. Du, J. K. Chen, S. J. Feng, J. L. Hu, S. F. Cui and G. C. Lu | 2016 | | Toxicity of Smokeless Tobacco Extract after 184-Day Repeated Oral Administration in Rats | International Journal of Environmental Research and Public Health | | 10.3390/ijerph13030281 |
| **600** | C. Q. Yu, H. J. Tang, Y. Guo, Z. Bian, L. Yang, Y. P. Chen, A. Y. Tang, X. Zhou, X. Yang, J. S. Chen, Z. M. Chen, J. Lv, L. M. Li and C. China Kadoorie Biobank | 2018 | | Hot Tea Consumption and Its Interactions With Alcohol and Tobacco Use on the Risk for Esophageal Cancer A Population-Based Cohort Study | Annals of Internal Medicine | | 10.7326/m17-2000 |
| **601** | H. Y. Yu, O. Hemminki, A. Försti, K. Sundquist and K. Hemminki | 2018 | | Familial Urinary Bladder Cancer with Other Cancers | European Urology Oncology | | 10.1016/j.euo.2018.06.002 |
| **602** | W. Q. Yu, H. J. Gao, G. D. Shi, J. Y. Tang, H. F. Wang, S. Y. Hu and Y. C. Wei | 2021 | | Development and validation of a nomogram to predict anastomotic leakage after esophagectomy for esophageal carcinoma | Journal of Thoracic Disease | | 10.21037/jtd-21-209 |
| **603** | Z. F. Yu, T. T. Zuo, H. H. Yu, Y. Zhao, Y. Zhang, J. H. Liu, S. L. Dong, Y. Wu and Y. Y. Liu | 2022 | | Outcomes of upper gastrointestinal cancer screening in high-risk individuals: a population-based prospective study in Northeast China | Bmj Open | | 10.1136/bmjopen-2020-046134 |
| **604** | K. E. Yun, Y. Chang, S. C. Yun, G. D. Smith, S. Ryu, S. I. Cho, E. C. Chung, H. Shin and Y. H. Khang | 2017 | | Alcohol and coronary artery calcification: an investigation using alcohol flushing as an instrumental variable | International Journal of Epidemiology | | 10.1093/ije/dyw237 |
| **605** | M. Zaitsu, R. Kaneko, T. Takeuchi, Y. Sato, Y. Kobayashi and I. Kawachi | 2018 | | Occupational inequalities in female cancer incidence in Japan: Hospital-based matched case-control study with occupational class | Ssm-Population Health | | 10.1016/j.ssmph.2018.06.001 |
| **606** | M. Zaitsu, R. Kaneko, T. Takeuchi, Y. Sato, Y. Kobayashi and I. Kawachi | 2019 | | Occupational class and male cancer incidence: Nationwide, multicenter, hospital-based case-control study in Japan | Cancer Medicine | | 10.1002/cam4.1945 |
| **607** | K. Y. Zee, P. S. Chan, J. C. S. Ho, S. M. L. Lai, E. F. Corbet and W. K. Leung | 2016 | | Adjunctive use of modified <i>Yunu</i>-<i>Jian</i> in the non-surgical treatment of male smokers with chronic periodontitis: a randomized double-blind, placebo-controlled clinical trial | Chinese Medicine | | 10.1186/s13020-016-0111-z |
| **608** | J. F. Zeng, Y. Y. Tang, P. Wu, X. Fang, W. Wang, Y. H. Fan, X. Li and S. P. Zhao | 2019 | | Alcohol consumption, tobacco smoking, betel quid chewing and oral health associations with hypopharyngeal cancer among men in Central South China: a case-control study | Cancer Management and Research | | 10.2147/cmar.S203439 |
| **609** | S. K. Zhan, X. Q. Wu and F. P. Zheng | 2021 | | Clinical Characteristics and Manifestations of Fungal Esophagitis: A Single-Center Experience in South China | Gastroenterology Research and Practice | | 10.1155/2021/8869494 |
| **610** | C. Zhang, F. Xu, Y. Qiang, Z. Z. Cong, Q. Wang, Z. Zhang, C. Luo, B. M. Qiu, L. W. Hu and Y. Shen | 2023 | | Prognostic significance of tumor regression grade in esophageal squamous cell carcinoma after neoadjuvant chemoradiation | Frontiers in Surgery | | 10.3389/fsurg.2022.1029575 |
| **611** | L. J. Zhang and W. Wang | 2020 | | Computed Tomography and Statistical Health Informatics Analysis of <i>Helicobacter pylori</i> Infection in Gastrointestinal Patients | Journal of Medical Imaging and Health Informatics | | 10.1166/jmihi.2020.3243 |
| **612** | Q. Zhang, X. Han, X. Y. Zhao and Y. Wang | 2022 | | Multimorbidity patterns and associated factors in older Chinese: results from the China health and retirement longitudinal study | Bmc Geriatrics | | 10.1186/s12877-022-03154-9 |
| **613** | S. S. Zhang, J. Y. Chen, B. Li, X. L. Cai, K. X. Wang, Z. H. Tan, Y. Z. Zheng and Q. W. Liu | 2022 | | Family history of cancer is a prognostic factor for better survival in operable esophageal squamous cell carcinoma: A propensity score matching analysis | Frontiers in Oncology | | 10.3389/fonc.2022.945937 |
| **614** | S. S. Zhang, Y. Y. Lei, X. L. Cai, H. Yang, X. Xia, K. J. Luo, C. H. Su, J. Y. Zou, B. Zeng, Y. Hu and H. H. Luo | 2016 | | Preoperative serum fibrinogen is an independent prognostic factor in operable esophageal cancer | Oncotarget | | 10.18632/oncotarget.8171 |
| **615** | T. C. Zhang, X. R. Yang, X. L. Yin, Z. Y. Yuan, H. Chen, L. Jin, X. D. Chen, M. Lu and W. M. Ye | 2022 | | Poor oral hygiene behavior is associated with an increased risk of gastric cancer: A population-based case-control study in China | Journal of Periodontology | | 10.1002/jper.21-0301 |
| **616** | Y. Zhang, Z. J. Li, W. Zhang, W. Chen and Y. P. Song | 2018 | | Risk factors for esophageal fistula in patients with locally advanced esophageal carcinoma receiving chemoradiotherapy | Oncotargets and Therapy | | 10.2147/ott.S161803 |
| **617** | G. Zhao, E. Ronda, L. Cea, J. Pulido, G. Barrio and E. Regidor | 2019 | | Mortality by cause of death and risk behaviors in farmers versus non-farmers: the importance of avoiding the healthy worker effect | International Archives of Occupational and Environmental Health | | 10.1007/s00420-018-1396-2 |
| **618** | Y. Zhao, J. Guo, B. You, S. C. Hou, B. Hu and H. Li | 2016 | | The dynamic of nasogastric decompression after esophagectomy and its predictive value of postoperative complications | Journal of Thoracic Disease | | 10.3978/j.issn.2072-1439.2015.10.72 |
| **619** | Y. Zheng, Y. Li, J. J. Qin, W. Q. Xing, X. B. Liu, H. B. Sun and X. K. Chen | 2019 | | Dysphagia predict the response to second cycle neoadjuvant chemotherapy in first cycle no response esophageal carcinoma | Journal of Thoracic Disease | | 10.21037/jtd.2019.10.02 |
| **620** | Y. Q. Zheng, Y. Huang, X. W. Zheng, J. T. Peng, Y. Chen, K. X. Yu, Y. Yang, X. Wang, X. Yang, J. X. Qian, X. D. Wang, X. L. Gao and B. Wu | 2021 | | Deaths from COPD in patients with cancer: a population-based study | Aging-Us | |  |
| **621** | S. Zhou, N. Van Devanter, M. Fenstermaker, P. Cawkwell, S. Sherman and M. Weitzman | 2015 | | A Study of the Use, Knowledge, and Beliefs About Cigarettes and Alternative Tobacco Products Among Students at One US Medical School | Academic Medicine | | 10.1097/acm.0000000000000873 |
| **622** | G. V. Zhuntova, T. Azizova and E. S. Grigoryeva | 2020 | | Risk of stomach cancer incidence in a cohort of Mayak PA workers occupationally exposed to ionizing radiation | Plos One | | 10.1371/journal.pone.0231531 |
| **623** | E. Zomawia, Z. Zami, A. Vanlallawma, N. S. Kumar, J. Zothanzama, L. Tlau, L. Chhakchhuak, L. Pachuau, J. L. Pautu and E. V. L. Hmangaihzuali | 2023 | | Cancer awareness, diagnosis and treatment needs in Mizoram, India: evidence from 18 years trends (2003-2020) | Lancet Regional Health - Southeast Asia | | 10.1016/j.lansea.2023.100281 |
| **624** | B. W. Zou, Y. Tu, D. W. Liao, Y. Xu, J. Wang, M. J. Huang, L. Ren, J. Zhu, Y. L. Gong, Y. M. Liu, L. Zhou, X. J. Zhou, F. Peng and Y. Lu | 2020 | | Radical esophagectomy for stage II and III thoracic esophageal squamous cell carcinoma followed by adjuvant radiotherapy with or without chemotherapy: Which is more beneficial? | Thoracic Cancer | | 10.1111/1759-7714.13307 |
| **625** | T. H. Zou, R. H. Zheng, Q. Y. Gao, X. Kong, X. Y. Chen, Z. Z. Ge, Y. X. Chen, X. P. Zou and J. Y. Fang | 2016 | | Factors affecting occurrence of gastric varioliform lesions: A case-control study | World Journal of Gastroenterology | | 10.3748/wjg.v22.i22.5228 |
| **626** | A. I. A. Abd Alrheam, M. M. M. Makhlouf, H. F. Gomaa and A. I. Abd Elneam | 2018 | | Biochemical and Histological Studies on the Effect of Nicotine on the Mucosa of Albino Rat Stomach | Research Journal of Pharmaceutical Biological and Chemical Sciences | |  |
