# Supplementary material for: Gastroesophageal disease risk and inhalational exposure a systematic review and meta-analysis
Source: Sci Rep. 2025 Jul 2;15:22581. doi: 10.1038/s41598-025-06620-7 (PMC12218983; doi:10.1038/s41598-025-06620-7)
Supplement: Supplementary file 3 — Supplementary Material 3. [file 41598_2025_6620_MOESM3_ESM.docx]

| **Supplemental Table 3. Non-Original Research Studies Excluded (N = 238)** | | | | | |
| --- | --- | --- | --- | --- | --- |
|  | **Author(s)** | **Title** | **Year** | **Journal** | **DOI** |
|  | **PubMed (N = 141)** | | | | |
| **1** | Abioye, A. I., Odesanya, M. O., Abioye, A. I. and Ibrahim, N. A. | 2015 | Physical activity and risk of gastric cancer: a meta-analysis of observational studies | Br J Sports Med | 10.1136/bjsports-2013-092778 |
| **2** | Abnet, C. C., Arnold, M. and Wei, W. Q. | 2018 | Epidemiology of Esophageal Squamous Cell Carcinoma | Gastroenterology | 10.1053/j.gastro.2017.08.023 |
| **3** | Aceves, S. S. | 2019 | Local Antigen Deposition in Eosinophilic Esophagitis: Implications for Immune Activation | Gastroenterology | 10.1053/j.gastro.2019.05.044 |
| **4** | Achilleos, A. | 2016 | Evidence-based Evaluation and Management of Chronic Cough | Med Clin North Am | 10.1016/j.mcna.2016.04.008 |
| **5** | Ansari, A. Z., Bhatia, N. Y., Gharat, S. A., Godad, A. P. and Doshi, G. M. | 2023 | Exploring Cytokines as Potential Target in Peptic Ulcer Disease: A Systematic Update | Endocr Metab Immune Disord Drug Targets | 10.2174/1871530322666220829142124 |
| **6** | Arora, Z., Garber, A. and Thota, P. N. | 2016 | Risk factors for Barrett's esophagus | J Dig Dis | 10.1111/1751-2980.12332 |
| **7** | Asanuma, K., Iijima, K. and Shimosegawa, T. | 2016 | Gender difference in gastro-esophageal reflux diseases | World J Gastroenterol | 10.3748/wjg.v22.i5.1800 |
| **8** | Asombang, A. W., Chishinga, N., Nkhoma, A., Chipaila, J., Nsokolo, B., Manda-Mapalo, M., Montiero, J. F. G., Banda, L. and Dua, K. S. | 2019 | Systematic review and meta-analysis of esophageal cancer in Africa: Epidemiology, risk factors, management and outcomes | World J Gastroenterol | 10.3748/wjg.v25.i31.4512 |
| **9** | Bede-Ojimadu, O. and Orisakwe, O. E. | 2020 | Exposure to Wood Smoke and Associated Health Effects in Sub-Saharan Africa: A Systematic Review | Ann Glob Health | 10.5334/aogh.2725 |
| **10** | Butt, J., Varga, M. G., Wang, T., Tsugane, S., Shimazu, T., Zheng, W., Abnet, C. C., Yoo, K. Y., Park, S. K., Kim, J., Jee, S. H., Qiao, Y. L., Shu, X. O., Waterboer, T., Pawlita, M. and Epplein, M. | 2019 | Smoking, Helicobacter Pylori Serology, and Gastric Cancer Risk in Prospective Studies from China, Japan, and Korea | Cancer Prev Res (Phila) | 10.1158/1940-6207.Capr-19-0238 |
| **11** | Camilleri, M. and Zheng, T. | 2023 | Cannabinoids and the Gastrointestinal Tract | Clin Gastroenterol Hepatol | 10.1016/j.cgh.2023.07.031 |
| **12** | Castro, C., Peleteiro, B. and Lunet, N. | 2018 | Modifiable factors and esophageal cancer: a systematic review of published meta-analyses | J Gastroenterol | 10.1007/s00535-017-1375-5 |
| **13** | Cavatorta, O., Scida, S., Miraglia, C., Barchi, A., Nouvenne, A., Leandro, G., Meschi, T., De' Angelis, G. L. and Di Mario, F. | 2018 | Epidemiology of gastric cancer and risk factors | Acta Biomed | 10.23750/abm.v89i8-S.7966 |
| **14** | Chatila, A. T., Nguyen, M. T. T., Krill, T., Roark, R., Bilal, M. and Reep, G. | 2020 | Natural history, pathophysiology and evaluation of gastroesophageal reflux disease | Dis Mon | 10.1016/j.disamonth.2019.02.001 |
| **15** | Chetwood, J. D., Garg, P., Finch, P. and Gordon, M. | 2019 | Systematic review: the etiology of esophageal squamous cell carcinoma in low-income settings | Expert Rev Gastroenterol Hepatol | 10.1080/17474124.2019.1543024 |
| **16** | Coleman, H. G., Xie, S. H. and Lagergren, J. | 2018 | The Epidemiology of Esophageal Adenocarcinoma | Gastroenterology | 10.1053/j.gastro.2017.07.046 |
| **17** | Collatuzzo, G., Pelucchi, C., Negri, E., López-Carrillo, L., Tsugane, S., Hidaka, A., Shigueaki Hamada, G., Hernández-Ramírez, R. U., López-Cervantes, M., Malekzadeh, R., Pourfarzi, F., Mu, L., Zhang, Z. F., Lunet, N., La Vecchia, C. and Boffetta, P. | 2021 | Exploring the interactions between Helicobacter pylori (Hp) infection and other risk factors of gastric cancer: A pooled analysis in the Stomach cancer Pooling (StoP) Project | Int J Cancer | 10.1002/ijc.33678 |
| **18** | Cook, M. B., Corley, D. A., Murray, L. J., Liao, L. M., Kamangar, F., Ye, W., Gammon, M. D., Risch, H. A., Casson, A. G., Freedman, N. D., Chow, W. H., Wu, A. H., Bernstein, L., Nyrén, O., Pandeya, N., Whiteman, D. C. and Vaughan, T. L. | 2014 | Gastroesophageal reflux in relation to adenocarcinomas of the esophagus: a pooled analysis from the Barrett's and Esophageal Adenocarcinoma Consortium (BEACON) | PLoS One | 10.1371/journal.pone.0103508 |
| **19** | DiSiena, M., Perelman, A., Birk, J. and Rezaizadeh, H. | 2021 | Esophageal Cancer: An Updated Review | South Med J | 10.14423/smj.0000000000001226 |
| **20** | Dong, J. and Thrift, A. P. | 2017 | Alcohol, smoking and risk of oesophago-gastric cancer | Best Pract Res Clin Gastroenterol | 10.1016/j.bpg.2017.09.002 |
| **21** | Drahos, J., Xiao, Q., Risch, H. A., Freedman, N. D., Abnet, C. C., Anderson, L. A., Bernstein, L., Brown, L., Chow, W. H., Gammon, M. D., Kamangar, F., Liao, L. M., Murray, L. J., Ward, M. H., Ye, W., Wu, A. H., Vaughan, T. L., Whiteman, D. C. and Cook, M. B. | 2016 | Age-specific risk factor profiles of adenocarcinomas of the esophagus: A pooled analysis from the international BEACON consortium | Int J Cancer | 10.1002/ijc.29688 |
| **22** | Dzahini, O., Singh, N., Taylor, D. and Haddad, P. M. | 2018 | Antipsychotic drug use and pneumonia: Systematic review and meta-analysis | J Psychopharmacol | 10.1177/0269881118795333 |
| **23** | Eusebi, L. H., Ratnakumaran, R., Yuan, Y., Solaymani-Dodaran, M., Bazzoli, F. and Ford, A. C. | 2018 | Global prevalence of, and risk factors for, gastro-oesophageal reflux symptoms: a meta-analysis | Gut | 10.1136/gutjnl-2016-313589 |
| **24** | Eusebi, L. H., Telese, A., Marasco, G., Bazzoli, F. and Zagari, R. M. | 2020 | Gastric cancer prevention strategies: A global perspective | J Gastroenterol Hepatol | 10.1111/jgh.15037 |
| **25** | Fahey, P. P., Mallitt, K. A., Astell-Burt, T., Stone, G. and Whiteman, D. C. | 2015 | Impact of pre-diagnosis behavior on risk of death from esophageal cancer: a systematic review and meta-analysis | Cancer Causes Control | 10.1007/s10552-015-0635-z |
| **26** | Ferro, A., Morais, S., Pelucchi, C., Aragonés, N., Kogevinas, M., López-Carrillo, L., Malekzadeh, R., Tsugane, S., Hamada, G. S., Hidaka, A., Hernández-Ramírez, R. U., López-Cervantes, M., Zaridze, D., Maximovitch, D., Pourfarzi, F., Zhang, Z. F., Yu, G. P., Pakseresht, M., Ye, W., Plymoth, A., Leja, M., Gasenko, E., Derakhshan, M. H., Negri, E., La Vecchia, C., Peleteiro, B. and Lunet, N. | 2019 | Smoking and Helicobacter pylori infection: an individual participant pooled analysis (Stomach Cancer Pooling- StoP Project) | Eur J Cancer Prev | 10.1097/cej.0000000000000471 |
| **27** | Ferro, A., Morais, S., Rota, M., Pelucchi, C., Bertuccio, P., Bonzi, R., Galeone, C., Zhang, Z. F., Matsuo, K., Ito, H., Hu, J., Johnson, K. C., Yu, G. P., Palli, D., Ferraroni, M., Muscat, J., Malekzadeh, R., Ye, W., Song, H., Zaridze, D., Maximovitch, D., Aragonés, N., Castaño-Vinyals, G., Vioque, J., Navarrete-Muñoz, E. M., Pakseresht, M., Pourfarzi, F., Wolk, A., Orsini, N., Bellavia, A., Håkansson, N., Mu, L., Pastorino, R., Kurtz, R. C., Derakhshan, M. H., Lagiou, A., Lagiou, P., Boffetta, P., Boccia, S., Negri, E., La Vecchia, C., Peleteiro, B. and Lunet, N. | 2018 | Tobacco smoking and gastric cancer: meta-analyses of published data versus pooled analyses of individual participant data (StoP Project) | Eur J Cancer Prev | 10.1097/cej.0000000000000401 |
| **28** | Gallo, O., Locatello, L. G., Larotonda, G., Napoleone, V. and Cannavicci, A. | 2018 | Nomograms for prediction of postoperative complications in open partial laryngeal surgery | J Surg Oncol | 10.1002/jso.25232 |
| **29** | Galway, N. C. and Shields, M. D. | 2019 | The child with an incessant dry cough | Paediatr Respir Rev | 10.1016/j.prrv.2018.08.002 |
| **30** | Gil, G. F., Anderson, J. A., Aravkin, A., Bhangdia, K., Carr, S., Dai, X., Flor, L. S., Hay, S. I., Malloy, M. J., McLaughlin, S. A., Mullany, E. C., Murray, C. J. L., O'Connell, E. M., Okereke, C., Sorensen, R. J. D., Whisnant, J., Zheng, P. and Gakidou, E. | 2024 | Health effects associated with chewing tobacco: a Burden of Proof study | Nat Commun | 10.1038/s41467-024-45074-9 |
| **31** | Giraldi, L., Stojanovic, J., Arzani, D., Persiani, R., Hu, J., Johnson, K. C., Zhang, Z. F., Ferraroni, M., Palli, D., Yu, G. P., La Vecchia, C., Pelucchi, C., Lunet, N., Ferro, A., Malekzadeh, R., Muscat, J., Zaridze, D., Maximovich, D., Aragones, N., Martin, V., Vioque, J., Navarrete-Munoz, E. M., Pakseresht, M., Negri, E., Rota, M., Pourfarzi, F., Mu, L., Kurtz, R. C., Lagiou, A., Lagiou, P., Pastorino, R. and Boccia, S. | 2023 | Adult height and risk of gastric cancer: a pooled analysis within the Stomach cancer Pooling Project | Eur J Cancer Prev | 10.1097/cej.0000000000000613 |
| **32** | Global Burden of Disease (GBD) 2017 Oesophageal Cancer Collaborators | 2020 | The global, regional, and national burden of oesophageal cancer and its attributable risk factors in 195 countries and territories, 1990-2017: a systematic analysis for the Global Burden of Disease Study 2017 | Lancet Gastroenterol Hepatol | 10.1016/s2468-1253(20)30007-8 |
| **33** | Grossman, K., Beasley, M. B. and Braman, S. S. | 2016 | Hepatoid adenocarcinoma of the lung: Review of a rare form of lung cancer | Respir Med | 10.1016/j.rmed.2016.09.003 |
| **34** | Houghton, L. A., Lee, A. S., Badri, H., DeVault, K. R. and Smith, J. A. | 2016 | Respiratory disease and the oesophagus: reflux, reflexes and microaspiration | Nat Rev Gastroenterol Hepatol | 10.1038/nrgastro.2016.91 |
| **35** | Huang, I. H., Schol, J., Khatun, R., Carbone, F., Van den Houte, K., Colomier, E., Balsiger, L. M., Törnblom, H., Vanuytsel, T., Sundelin, E., Simrén, M., Palsson, O. S., Bangdiwala, S. I., Sperber, A. D. and Tack, J. | 2022 | Worldwide prevalence and burden of gastroparesis-like symptoms as defined by the United European Gastroenterology (UEG) and European Society for Neurogastroenterology and Motility (ESNM) consensus on gastroparesis | United European Gastroenterol J | 10.1002/ueg2.12289 |
| **36** | Hudler, P. | 2015 | Challenges of deciphering gastric cancer heterogeneity | World J Gastroenterol | 10.3748/wjg.v21.i37.10510 |
| **37** | Ireland, C. J., Thompson, S. K., Laws, T. A. and Esterman, A. | 2016 | Risk factors for Barrett's esophagus: a scoping review | Cancer Causes Control | 10.1007/s10552-015-0710-5 |
| **38** | Jia, X., Sheng, C., Han, X., Li, M. and Wang, K. | 2024 | Global burden of stomach cancer attributable to smoking from 1990 to 2019 and predictions to 2044 | Public Health | 10.1016/j.puhe.2023.11.019 |
| **39** | Kamarajah, S. K. and Phillips, A. W. | 2021 | ASO Author Reflections: Smoking Status Impact on Perioperative Morbidity and Long-Term Survival of Patients Undergoing Esophagectomy for Cancer | Ann Surg Oncol | 10.1245/s10434-021-09765-7 |
| **40** | Kamboj, A. K., Gibbens, Y. Y., Hagen, C. E., Wang, K. K., Iyer, P. G. and Katzka, D. A. | 2021 | Esophageal Epidermoid Metaplasia: Clinical Characteristics and Risk of Esophageal Squamous Neoplasia | Am J Gastroenterol | 10.14309/ajg.0000000000001225 |
| **41** | Kang, H. H., Seo, M., Lee, J., Ha, S. Y., Oh, J. H. and Lee, S. H. | 2021 | Reflux esophagitis in patients with chronic obstructive pulmonary disease | Medicine (Baltimore) | 10.1097/md.0000000000027091 |
| **42** | Kayamba, V., Heimburger, D. C., Morgan, D. R., Atadzhanov, M. and Kelly, P. | 2017 | Exposure to biomass smoke as a risk factor for oesophageal and gastric cancer in low-income populations: A systematic review | Malawi Med J | 10.4314/mmj.v29i2.25 |
| **43** | Khan, T., Relitti, N., Brindisi, M., Magnano, S., Zisterer, D., Gemma, S., Butini, S. and Campiani, G. | 2020 | Autophagy modulators for the treatment of oral and esophageal squamous cell carcinomas | Med Res Rev | 10.1002/med.21646 |
| **44** | Kim, D. H., Park, J. Y., Karm, M. H., Bae, H. Y., Lee, J. Y., Soo Ahn, H., Lee, K. and Leem, J. G. | 2017 | Smoking May Increase Postoperative Opioid Consumption in Patients Who Underwent Distal Gastrectomy With Gastroduodenostomy for Early Stomach Cancer: A Retrospective Analysis | Clin J Pain | 10.1097/ajp.0000000000000472 |
| **45** | Kim, S. A., Choi, B. Y., Song, K. S., Park, C. H., Eun, C. S., Han, D. S., Kim, Y. S. and Kim, H. J. | 2019 | Prediagnostic Smoking and Alcohol Drinking and Gastric Cancer Survival: A Korean Prospective Cohort Study | Korean J Gastroenterol | 10.4166/kjg.2019.73.3.141 |
| **46** | Kissiedu, J., Thota, P. N., Gohel, T., Lopez, R. and Gordon, I. O. | 2016 | Post-ablation lymphocytic esophagitis in Barrett esophagus with high grade dysplasia or intramucosal carcinoma | Mod Pathol | 10.1038/modpathol.2016.50 |
| **47** | Ko, K. P., Shin, A., Cho, S., Park, S. K. and Yoo, K. Y. | 2018 | Environmental contributions to gastrointestinal and liver cancer in the Asia-Pacific region | J Gastroenterol Hepatol | 10.1111/jgh.14005 |
| **48** | Kosaka, M., Yamazaki, Y., Maruno, T., Sakaguchi, K. and Sawaki, S. | 2021 | Corticosteroids as adjunctive therapy in the treatment of coronavirus disease 2019: A report of two cases and literature review | J Infect Chemother | 10.1016/j.jiac.2020.09.007 |
| **49** | Krishnamoorthi, R., Singh, S., Ragunathan, K., Visrodia, K., Wang, K. K., Katzka, D. A. and Iyer, P. G. | 2018 | Factors Associated With Progression of Barrett's Esophagus: A Systematic Review and Meta-analysis | Clin Gastroenterol Hepatol | 10.1016/j.cgh.2017.11.044 |
| **50** | Kurotschka, P. K., Serafini, A. and Ebell, M. H. | 2024 | [Top 4 Research Studies of the month for Italian Primary Care Physicians: December 2023.] | Recenti Prog Med | 10.1701/4169.41641 |
| **51** | Lai, K. and Long, L. | 2020 | Current Status and Future Directions of Chronic Cough in China | Lung | 10.1007/s00408-019-00319-z |
| **52** | Lam, A. K. | 2020 | Introduction: Esophageal Squamous Cell Carcinoma-Current Status and Future Advances | Methods Mol Biol | 10.1007/978-1-0716-0377-2_1 |
| **53** | Li, L. F., Chan, R. L., Lu, L., Shen, J., Zhang, L., Wu, W. K., Wang, L., Hu, T., Li, M. X. and Cho, C. H. | 2014 | Cigarette smoking and gastrointestinal diseases: the causal relationship and underlying molecular mechanisms (review) | Int J Mol Med | 10.3892/ijmm.2014.1786 |
| **54** | Li, N., Wu, P., Shen, Y., Yang, C., Zhang, L., Chen, Y., Wang, Z. and Jiang, J. | 2021 | Predictions of mortality related to four major cancers in China, 2020 to 2030 | Cancer Commun (Lond) | 10.1002/cac2.12143 |
| **55** | Li, P., Jing, J., Liu, W., Wang, J., Qi, X. and Zhang, G. | 2023 | Spatiotemporal Patterns of Esophageal Cancer Burden Attributable to Behavioral, Metabolic, and Dietary Risk Factors From 1990 to 2019: Longitudinal Observational Study | JMIR Public Health Surveill | 10.2196/46051 |
| **56** | Li, Q., Zhu, L., Wei, T., Zang, Z., Zhang, X., Wang, Y., Gao, R., Zhang, Y., Zheng, X. and Liu, F. | 2023 | Secular trends and attributable risk factors of esophageal cancer deaths among non-elderly adults based on Global Burden of Disease Study | J Cancer Res Clin Oncol | 10.1007/s00432-023-05380-z |
| **57** | Li, S., Chen, H., Man, J., Zhang, T., Yin, X., He, Q., Yang, X. and Lu, M. | 2021 | Changing trends in the disease burden of esophageal cancer in China from 1990 to 2017 and its predicted level in 25 years | Cancer Med | 10.1002/cam4.3775 |
| **58** | Li, W. Y., Han, Y., Xu, H. M., Wang, Z. N., Xu, Y. Y., Song, Y. X., Xu, H., Yin, S. C., Liu, X. Y. and Miao, Z. F. | 2019 | Smoking status and subsequent gastric cancer risk in men compared with women: a meta-analysis of prospective observational studies | BMC Cancer | 10.1186/s12885-019-5601-9 |
| **59** | Lie, T. M., Bomme, M., Hveem, K., Hansen, J. M. and Ness-Jensen, E. | 2017 | Snus and risk of gastroesophageal reflux. A population-based case-control study: the HUNT study | Scand J Gastroenterol | 10.1080/00365521.2016.1245775 |
| **60** | Lim, J. H., Lee, D. H., Lee, S. H., Kim, J. S., Jung, H. C. and Cho, S. H. | 2019 | Asthma under control is inversely related with erosive esophagitis among healthy adults | PLoS One | 10.1371/journal.pone.0210490 |
| **61** | Lin, R. A., Calvert, G. M. and Udasin, I. G. | 2023 | World Trade Center Health Program best practices for the diagnosis and treatment of gastroesophageal reflux disease | Arch Environ Occup Health | 10.1080/19338244.2023.2171958 |
| **62** | Lin, Y., Zheng, Y., Wang, H. L. and Wu, J. | 2021 | Global Patterns and Trends in Gastric Cancer Incidence Rates (1988-2012) and Predictions to 2030 | Gastroenterology | 10.1053/j.gastro.2021.03.023 |
| **63** | Lipka, S., Kumar, A. and Richter, J. E. | 2016 | Impact of Diagnostic Delay and Other Risk Factors on Eosinophilic Esophagitis Phenotype and Esophageal Diameter | J Clin Gastroenterol | 10.1097/mcg.0000000000000297 |
| **64** | Lleo, A. and Colapietro, F. | 2018 | Changes in the Epidemiology of Primary Biliary Cholangitis | Clin Liver Dis | 10.1016/j.cld.2018.03.001 |
| **65** | Long, L. and Lai, K. | 2019 | Characteristics of Chinese chronic cough patients | Pulm Pharmacol Ther | 10.1016/j.pupt.2019.101811 |
| **66** | Lund, I. and Scheffels, J. | 2014 | Perceptions of relative risk of disease and addiction from cigarettes and snus | Psychol Addict Behav | 10.1037/a0032657 |
| **67** | Luo, M. X., Long, B. B., Li, F., Zhang, C., Pan, M. T., Huang, Y. Q. and Chen, B. | 2019 | Roles of Cyclooxygenase-2 gene -765G > C (rs20417) and -1195G > A (rs689466) polymorphisms in gastric cancer: A systematic review and meta-analysis | Gene | 10.1016/j.gene.2018.10.077 |
| **68** | Lyons, K., Le, L. C., Pham, Y. T., Borron, C., Park, J. Y., Tran, C. T. D., Tran, T. V., Tran, H. T., Vu, K. T., Do, C. D., Pelucchi, C., La Vecchia, C., Zgibor, J., Boffetta, P. and Luu, H. N. | 2019 | Gastric cancer: epidemiology, biology, and prevention: a mini review | Eur J Cancer Prev | 10.1097/cej.0000000000000480 |
| **69** | Mahale, P., Sturgis, E. M., Tweardy, D. J., Ariza-Heredia, E. J. and Torres, H. A. | 2016 | Association Between Hepatitis C Virus and Head and Neck Cancers | J Natl Cancer Inst | 10.1093/jnci/djw035 |
| **70** | Marqués-Lespier, J. M., González-Pons, M. and Cruz-Correa, M. | 2016 | Current Perspectives on Gastric Cancer | Gastroenterol Clin North Am | 10.1016/j.gtc.2016.04.002 |
| **71** | Matejcic, M., Gunter, M. J. and Ferrari, P. | 2017 | Alcohol metabolism and oesophageal cancer: a systematic review of the evidence | Carcinogenesis | 10.1093/carcin/bgx067 |
| **72** | Matsueda, K., Manabe, N., Toshikuni, N., Sato, Y., Watanabe, T., Yamamoto, H. and Haruma, K. | 2017 | Clinical characteristics and associated factors of Japanese patients with adenocarcinoma of the esophagogastric junction: a multicenter clinicoepidemiological study | Dis Esophagus | 10.1093/dote/dox007 |
| **73** | May Maestas, M., Perry, K. D., Smith, K., Firszt, R., Allen-Brady, K., Robson, J., Joy, E. and Peterson, K. | 2019 | Food impactions in Eosinophilic esophagitis and acute exposures to fine particulate pollution | Allergy | 10.1111/all.13932 |
| **74** | McCallum, R. W. and Bashashati, M. | 2019 | Cannabis for Gastroparesis: Hype or Hope? | Am J Gastroenterol | 10.14309/ajg.0000000000000277 |
| **75** | McCarty, T. R., Chouairi, F., Hathorn, K. E., Chan, W. W. and Thompson, C. C. | 2022 | Trends and Socioeconomic Health Outcomes of Cannabis Use Among Patients With Gastroparesis: A United States Nationwide Inpatient Sample Analysis | J Clin Gastroenterol | 10.1097/mcg.0000000000001526 |
| **76** | Mehrtash, H., Duncan, K., Parascandola, M., David, A., Gritz, E. R., Gupta, P. C., Mehrotra, R., Amer Nordin, A. S., Pearlman, P. C., Warnakulasuriya, S., Wen, C. P., Zain, R. B. and Trimble, E. L. | 2017 | Defining a global research and policy agenda for betel quid and areca nut | Lancet Oncol | 10.1016/s1470-2045(17)30460-6 |
| **77** | Mills, R. and Hathorn, I. | 2016 | Aetiology and pathology of otitis media with effusion in adult life | J Laryngol Otol | 10.1017/s0022215116000943 |
| **78** | Minakari, M., Badihian, S., Jalalpour, P. and Sebghatollahi, V. | 2017 | Etiology and outcome in patients with upper gastrointestinal bleeding: Study on 4747 patients in the central region of Iran | J Gastroenterol Hepatol | 10.1111/jgh.13617 |
| **79** | Minowa, H. | 2016 | Respiratory inhibition after crying or gastroesophageal reflux and feeding hypoxemia in infants | J Matern Fetal Neonatal Med | 10.3109/14767058.2015.1085011 |
| **80** | Mohy-Ud-Din, N., Krill, T. S., Shah, A. R., Chatila, A. T., Singh, S., Bilal, M. and Parupudi, S. | 2020 | Barrett's esophagus: What do we need to know? | Dis Mon | 10.1016/j.disamonth.2019.02.003 |
| **81** | Montazeri, Z., Nyiraneza, C., El-Katerji, H. and Little, J. | 2017 | Waterpipe smoking and cancer: systematic review and meta-analysis | Tob Control | 10.1136/tobaccocontrol-2015-052758 |
| **82** | Morais, S., Rodrigues, S., Amorim, L., Peleteiro, B. and Lunet, N. | 2014 | Tobacco smoking and intestinal metaplasia: Systematic review and meta-analysis | Dig Liver Dis | 10.1016/j.dld.2014.08.034 |
| **83** | Naclerio, R. M. and Baroody, F. M. | 2016 | Other Phenotypes and Treatment of Chronic Rhinosinusitis | J Allergy Clin Immunol Pract | 10.1016/j.jaip.2016.03.016 |
| **84** | Najafi, F. | 2019 | Tobacco Smoking and Alcohol Drinking: Two Clinically Significant Risk Factors for Esophageal Squamous Cell Carcinoma | Gastroenterology | 10.1053/j.gastro.2019.04.054 |
| **85** | Ness-Jensen, E., Hveem, K., El-Serag, H. and Lagergren, J. | 2016 | Lifestyle Intervention in Gastroesophageal Reflux Disease | Clin Gastroenterol Hepatol | 10.1016/j.cgh.2015.04.176 |
| **86** | Ness-Jensen, E. and Lagergren, J. | 2017 | Tobacco smoking, alcohol consumption and gastro-oesophageal reflux disease | Best Pract Res Clin Gastroenterol | 10.1016/j.bpg.2017.09.004 |
| **87** | Nicolini, A., Barbagelata, E., Tagliabue, E., Colombo, D., Monacelli, F. and Braido, F. | 2018 | Gender differences in chronic obstructive pulmonary diseases: a narrative review | Panminerva Med | 10.23736/s0031-0808.18.03463-8 |
| **88** | Ohashi, S., Miyamoto, S., Kikuchi, O., Goto, T., Amanuma, Y. and Muto, M. | 2015 | Recent Advances From Basic and Clinical Studies of Esophageal Squamous Cell Carcinoma | Gastroenterology | 10.1053/j.gastro.2015.08.054 |
| **89** | Olsson, E. C., Jobson, M. and Lim, M. R. | 2015 | Risk factors for persistent dysphagia after anterior cervical spine surgery | Orthopedics | 10.3928/01477447-20150402-61 |
| **90** | Ovnat Tamir, S., Gershnabel Milk, D., Roth, Y., Cinamon, U., Winder, A., Brenner, R., Katz, A. and Marom, T. | 2016 | Laryngeal Side Effects of Tyrosine Kinase Inhibitors | J Voice | 10.1016/j.jvoice.2015.07.006 |
| **91** | Oze, I., Charvat, H., Matsuo, K., Ito, H., Tamakoshi, A., Nagata, C., Wada, K., Sugawara, Y., Sawada, N., Yamaji, T., Naito, M., Tanaka, K., Shimazu, T., Mizoue, T., Tsugane, S. and Inoue, M. | 2019 | Revisit of an unanswered question by pooled analysis of eight cohort studies in Japan: Does cigarette smoking and alcohol drinking have interaction for the risk of esophageal cancer? | Cancer Med | 10.1002/cam4.2514 |
| **92** | Parsel, S. M., Wu, E. L., Riley, C. A. and McCoul, E. D. | 2019 | Gastroesophageal and Laryngopharyngeal Reflux Associated With Laryngeal Malignancy: A Systematic Review and Meta-analysis | Clin Gastroenterol Hepatol | 10.1016/j.cgh.2018.10.028 |
| **93** | Pasricha, S., Gupta, A., Reed, C. C., Speck, O., Woosley, J. T. and Dellon, E. S. | 2016 | Lymphocytic Esophagitis: An Emerging Clinicopathologic Disease Associated with Dysphagia | Dig Dis Sci | 10.1007/s10620-016-4230-2 |
| **94** | Peleteiro, B., Castro, C., Morais, S., Ferro, A. and Lunet, N. | 2015 | Worldwide Burden of Gastric Cancer Attributable to Tobacco Smoking in 2012 and Predictions for 2020 | Dig Dis Sci | 10.1007/s10620-015-3624-x |
| **95** | Piloiu, C. and Dumitrascu, D. L. | 2020 | Barrett's Esophagus in Romania: what do we know? | Rom J Intern Med | 10.2478/rjim-2020-0007 |
| **96** | Popa, S. L., Chiarioni, G., David, L., Golea, G. I. and Dumitrascu, D. L. | 2019 | Functional Emesis | J Gastrointestin Liver Dis | 10.15403/jgld-236 |
| **97** | Prabhu, A., Obi, K. O. and Rubenstein, J. H. | 2014 | The synergistic effects of alcohol and tobacco consumption on the risk of esophageal squamous cell carcinoma: a meta-analysis | Am J Gastroenterol | 10.1038/ajg.2014.71 |
| **98** | Praud, D., Rota, M., Pelucchi, C., Bertuccio, P., Rosso, T., Galeone, C., Zhang, Z. F., Matsuo, K., Ito, H., Hu, J., Johnson, K. C., Yu, G. P., Palli, D., Ferraroni, M., Muscat, J., Lunet, N., Peleteiro, B., Malekzadeh, R., Ye, W., Song, H., Zaridze, D., Maximovitch, D., Aragonés, N., Castaño-Vinyals, G., Vioque, J., Navarrete-Muñoz, E. M., Pakseresht, M., Pourfarzi, F., Wolk, A., Orsini, N., Bellavia, A., Håkansson, N., Mu, L., Pastorino, R., Kurtz, R. C., Derakhshan, M. H., Lagiou, A., Lagiou, P., Boffetta, P., Boccia, S., Negri, E. and La Vecchia, C. | 2018 | Cigarette smoking and gastric cancer in the Stomach Cancer Pooling (StoP) Project | Eur J Cancer Prev | 10.1097/cej.0000000000000290 |
| **99** | Qin, Y., Tong, X., Fan, J., Liu, Z., Zhao, R., Zhang, T., Suo, C., Chen, X. and Zhao, G. | 2021 | Global Burden and Trends in Incidence, Mortality, and Disability of Stomach Cancer From 1990 to 2017 | Clin Transl Gastroenterol | 10.14309/ctg.0000000000000406 |
| **100** | Rai, S., Kulkarni, A. and Ghoshal, U. C. | 2021 | Prevalence and risk factors for gastroesophageal reflux disease in the Indian population: A meta-analysis and meta-regression study | Indian J Gastroenterol | 10.1007/s12664-020-01104-0 |
| **101** | Rameez, M. H. and Mayberry, J. F. | 2015 | Epidemiology and risk factors for Barrett's oesophagus | Br J Hosp Med (Lond) | 10.12968/hmed.2015.76.3.138 |
| **102** | Ramôa, C. P., Eissenberg, T. and Sahingur, S. E. | 2017 | Increasing popularity of waterpipe tobacco smoking and electronic cigarette use: Implications for oral healthcare | J Periodontal Res | 10.1111/jre.12458 |
| **103** | Rosenthal, D. I., Mohamed, A. S., Weber, R. S., Garden, A. S., Sevak, P. R., Kies, M. S., Morrison, W. H., Lewin, J. S., El-Naggar, A. K., Ginsberg, L. E., Kocak-Uzel, E., Ang, K. K. and Fuller, C. D. | 2015 | Long-term outcomes after surgical or nonsurgical initial therapy for patients with T4 squamous cell carcinoma of the larynx: A 3-decade survey | Cancer | 10.1002/cncr.29241 |
| **104** | Rota, M., Possenti, I., Valsassina, V., Santucci, C., Bagnardi, V., Corrao, G., Bosetti, C., Specchia, C., Gallus, S. and Lugo, A. | 2024 | Dose-response association between cigarette smoking and gastric cancer risk: a systematic review and meta-analysis | Gastric Cancer | 10.1007/s10120-023-01459-1 |
| **105** | Runge, T. M., Abrams, J. A. and Shaheen, N. J. | 2015 | Epidemiology of Barrett's Esophagus and Esophageal Adenocarcinoma | Gastroenterol Clin North Am | 10.1016/j.gtc.2015.02.001 |
| **106** | Sack, C. and Raghu, G. | 2019 | Idiopathic pulmonary fibrosis: unmasking cryptogenic environmental factors | Eur Respir J | 10.1183/13993003.01699-2018 |
| **107** | Sakthivel, P., Samy, K., Panda, S. and Amit Singh, C. | 2021 | 14 "S" in head and neck cancers | Oral Oncol | 10.1016/j.oraloncology.2020.105070 |
| **108** | Sardana, R. K., Chhikara, N., Tanwar, B. and Panghal, A. | 2018 | Dietary impact on esophageal cancer in humans: a review | Food Funct | 10.1039/c7fo01908d |
| **109** | Scida, S., Russo, M., Miraglia, C., Leandro, G., Franzoni, L., Meschi, T., De' Angelis, G. L. and Di Mario, F. | 2018 | Relationship between Helicobacter pylori infection and GERD | Acta Biomed | 10.23750/abm.v89i8-S.7918 |
| **110** | Sethi, S. and Richter, J. E. | 2017 | Diet and gastroesophageal reflux disease: role in pathogenesis and management | Curr Opin Gastroenterol | 10.1097/mog.0000000000000337 |
| **111** | Short, M. W., Burgers, K. G. and Fry, V. T. | 2017 | Esophageal Cancer | Am Fam Physician |  |
| **112** | Siddiqi, K., Shah, S., Abbas, S. M., Vidyasagaran, A., Jawad, M., Dogar, O. and Sheikh, A. | 2015 | Global burden of disease due to smokeless tobacco consumption in adults: analysis of data from 113 countries | BMC Med | 10.1186/s12916-015-0424-2 |
| **113** | Sonnenberg, W. R. | 2017 | Gastrointestinal Malignancies | Prim Care | 10.1016/j.pop.2017.07.013 |
| **114** | Splittgerber, M. and Velanovich, V. | 2015 | Barrett esophagus | Surg Clin North Am | 10.1016/j.suc.2015.02.011 |
| **115** | Tavaluc, R. and Tan-Geller, M. | 2019 | Reinke's Edema | Otolaryngol Clin North Am | 10.1016/j.otc.2019.03.006 |
| **116** | Thrift, A. P. | 2016 | Determination of risk for Barrett's esophagus and esophageal adenocarcinoma | Curr Opin Gastroenterol | 10.1097/mog.0000000000000274 |
| **117** | Thrift, A. P. | 2016 | The epidemic of oesophageal carcinoma: Where are we now? | Cancer Epidemiol | 10.1016/j.canep.2016.01.013 |
| **118** | Torre, L. A., Bray, F., Siegel, R. L., Ferlay, J., Lortet-Tieulent, J. and Jemal, A. | 2015 | Global cancer statistics, 2012 | CA Cancer J Clin | 10.3322/caac.21262 |
| **119** | Underner, M. | 2008 | [Underrated effects of tobacco and marijuana smoking on the thyroid, the esophagus, the kidney, the skeletal system and the mouth] | Rev Mal Respir | 10.1016/s0761-8425(08)75108-8 |
| **120** | Usai-Satta, P., Bellini, M., Morelli, O., Geri, F., Lai, M. and Bassotti, G. | 2020 | Gastroparesis: New insights into an old disease | World J Gastroenterol | 10.3748/wjg.v26.i19.2333 |
| **121** | Wang, Y., Jiang, Y. and Zhu, Z. | 2021 | Question on Databases Used to Examine the Association Between Gastric Cancer and Heavy Alcohol Use | Am J Gastroenterol | 10.14309/ajg.0000000000001392 |
| **122** | Weinmayr, G., Chen, J., Jaensch, A., Skodda, L., Rodopoulou, S., Strak, M., de Hoogh, K., Andersen, Z. J., Bellander, T., Brandt, J., Fecht, D., Forastiere, F., Gulliver, J., Hertel, O., Hoffmann, B., Hvidtfeldt, U. A., Katsouyanni, K., Ketzel, M., Leander, K., Magnusson, P. K. E., Pershagen, G., Rizzuto, D., Samoli, E., Severi, G., Stafoggia, M., Tjønneland, A., Vermeulen, R., Wolf, K., Zitt, E., Brunekreef, B., Thurston, G., Hoek, G., Raaschou-Nielsen, O. and Nagel, G. | 2024 | Long-term exposure to several constituents and sources of PM(2.5) is associated with incidence of upper aerodigestive tract cancers but not gastric cancer: Results from the large pooled European cohort of the ELAPSE project | Sci Total Environ | 10.1016/j.scitotenv.2023.168789 |
| **123** | Weinmayr, G., Pedersen, M., Stafoggia, M., Andersen, Z. J., Galassi, C., Munkenast, J., Jaensch, A., Oftedal, B., Krog, N. H., Aamodt, G., Pyko, A., Pershagen, G., Korek, M., De Faire, U., Pedersen, N. L., Östenson, C. G., Rizzuto, D., Sørensen, M., Tjønneland, A., Bueno-de-Mesquita, B., Vermeulen, R., Eeftens, M., Concin, H., Lang, A., Wang, M., Tsai, M. Y., Ricceri, F., Sacerdote, C., Ranzi, A., Cesaroni, G., Forastiere, F., de Hoogh, K., Beelen, R., Vineis, P., Kooter, I., Sokhi, R., Brunekreef, B., Hoek, G., Raaschou-Nielsen, O. and Nagel, G. | 2018 | Particulate matter air pollution components and incidence of cancers of the stomach and the upper aerodigestive tract in the European Study of Cohorts of Air Pollution Effects (ESCAPE) | Environ Int | 10.1016/j.envint.2018.07.030 |
| **124** | Weinreb, S. F., Piersiala, K., Hillel, A. T., Akst, L. M. and Best, S. R. | 2021 | Dysphonia and dysphagia as early manifestations of autoimmune inflammatory myopathy | Am J Otolaryngol | 10.1016/j.amjoto.2020.102747 |
| **125** | Westra, W. M., Lutzke, L. S., Mostafavi, N. S., Roes, A. L., Calpe, S., Wang, K. K. and Krishnadath, K. K. | 2018 | Smokeless Tobacco and Cigar and/or Pipe Are Risk Factors for Barrett Esophagus in Male Patients With Gastroesophageal Reflux Disease | Mayo Clin Proc | 10.1016/j.mayocp.2018.04.022 |
| **126** | Wu, G., Wu, Q., Xu, J., Gao, G., Chen, T. and Chen, G. | 2024 | Mortality burden and future projections of major risk factors for esophageal cancer in China from 1990 to 2019 | Gen Thorac Cardiovasc Surg | 10.1007/s11748-023-01987-8 |
| **127** | Wu, H. and Chen, H. L. | 2021 | The Association Between Heavy Alcohol Use and Gastric Cancer | Am J Gastroenterol | 10.14309/ajg.0000000000001325 |
| **128** | Wu, X. C., Zheng, Y. F., Tang, M., Li, X. F., Zeng, R. and Zhang, J. R. | 2015 | Association Between Smoking and p53 Mutation in Oesophageal Squamous Cell Carcinoma: A Meta-analysis | Clin Oncol (R Coll Radiol) | 10.1016/j.clon.2015.02.007 |
| **129** | Xie, F., Wang, D., Huang, Z. and Guo, Y. | 2014 | Coffee consumption and risk of gastric cancer: a large updated meta-analysis of prospective studies | Nutrients | 10.3390/nu6093734 |
| **130** | Xu, Y., Wang, J., He, Z., Rao, Z., Zhang, Z., Zhou, J., Zhou, T. and Wang, H. | 2024 | A review on the effect of COX-2-mediated mechanisms on development and progression of gastric cancer induced by nicotine | Biochem Pharmacol | 10.1016/j.bcp.2023.115980 |
| **131** | Yang, S., Lin, S., Li, N., Deng, Y., Wang, M., Xiang, D., Xiang, G., Wang, S., Ye, X., Zheng, Y., Yao, J., Zhai, Z., Wu, Y., Hu, J., Kang, H. and Dai, Z. | 2020 | Burden, trends, and risk factors of esophageal cancer in China from 1990 to 2017: an up-to-date overview and comparison with those in Japan and South Korea | J Hematol Oncol | 10.1186/s13045-020-00981-4 |
| **132** | Yaqoob, Z., Al-Kindi, S. G. and Zein, J. | 2016 | Association Between Celiac Disease and Asthma | Dig Dis Sci | 10.1007/s10620-016-4321-0 |
| **133** | Yu, J., Deng, Y. and Chen, J. P. | 2014 | N-acetyltransferase 2 status and gastric cancer risk: a meta-analysis | Tumour Biol | 10.1007/s13277-014-1847-7 |
| **134** | Yu, J., Yang, P., Qin, X., Li, C., Lv, Y. and Wang, X. | 2022 | Impact of smoking on the eradication of Helicobacter pylori | Helicobacter | 10.1111/hel.12860 |
| **135** | Zakko, L., Lutzke, L. and Wang, K. K. | 2017 | Screening for Barrett's esophagus | Minerva Med | 10.23736/s0026-4806.16.04864-3 |
| **136** | Zhang, L., Jiang, Y., Wu, Q., Li, Q., Chen, D., Xu, L., Zhang, C., Zhang, M. and Ye, L. | 2014 | Gene-environment interactions on the risk of esophageal cancer among Asian populations with the G48A polymorphism in the alcohol dehydrogenase-2 gene: a meta-analysis | Tumour Biol | 10.1007/s13277-014-1616-7 |
| **137** | Zhang, Y. and Tong, T. | 2018 | Clinical Significance of O-6-Methylguanine-DNA-Methyltransferase Promoter Methylation in Patients with Esophageal Carcinoma: A Systematic Meta-Analysis | Dig Dis | 10.1159/000481342 |
| **138** | Zhao, L. L., Huang, H., Wang, Y., Wang, T. B., Zhou, H., Ma, F. H., Ren, H., Niu, P. H., Zhao, D. B. and Chen, Y. T. | 2020 | Lifestyle factors and long-term survival of gastric cancer patients: A large bidirectional cohort study from China | World J Gastroenterol | 10.3748/wjg.v26.i14.1613 |
| **139** | Zhao, X. and Lim, F. | 2020 | Lifestyle Risk Factors in Esophageal Cancer: An Integrative Review | Crit Care Nurs Q | 10.1097/cnq.0000000000000295 |
| **140** | Zhao, Z., Yin, Z. and Zhang, C. | 2021 | Lifestyle interventions can reduce the risk of Barrett's esophagus: a systematic review and meta-analysis of 62 studies involving 250,157 participants | Cancer Med | 10.1002/cam4.4061 |
| **141** | Zheng, L. M., Zhang, Z. W., Wang, W., Li, Y. and Wen, F. | 2022 | Relationship between smoking and postoperative complications of cervical spine surgery: a systematic review and meta-analysis | Sci Rep | 10.1038/s41598-022-13198-x |
|  | **Web of Science (N = 97)** | | | | |
| **1** | A. Alcaraz, J. Caporale, A. Bardach, F. Augustovski and A. Pichon-Riviere | 2016 | Burden of disease attributable to tobacco use in Argentina and potential impact of price increases through taxes | Revista Panamericana De Salud Publica-Pan American Journal of Public Health |  |
| **2** | Z. Arora, A. Garber and P. N. Thota | 2016 | Risk factors for Barrett's esophagus | Journal of Digestive Diseases | 10.1111/1751-2980.12332 |
| **3** | Z. Bilgi and S. J. Swanson | 2019 | Current indications and outcomes for thoracoscopic segmentectomy for early stage lung cancer | Journal of Thoracic Disease | 10.21037/jtd.2019.07.06 |
| **4** | C. Bryce, M. Bucaj and R. Gazda | 2022 | Barrett Esophagus: Rapid Evidence Review | American Family Physician |  |
| **5** | G. Capurso and E. Lahner | 2017 | The interaction between smoking, alcohol and the gut microbiome | Best Practice & Research Clinical Gastroenterology | 10.1016/j.bpg.2017.10.006 |
| **6** | Y. S. Chuang, M. C. Wu, F. J. Yu, Y. K. Wang, C. Y. Lu, D. C. Wu, C. T. Kuo, M. T. Wu and I. C. Wu | 2017 | Effects of alcohol consumption, cigarette smoking, and betel quid chewing on upper digestive diseases: a large cross-sectional study and meta-analysis | Oncotarget | 10.18632/oncotarget.20831 |
| **7** | H. G. Coleman, S. H. Xie and J. Lagergren | 2018 | The Epidemiology of Esophageal Adenocarcinoma | Gastroenterology | 10.1053/j.gastro.2017.07.046 |
| **8** | G. Collatuzzo, J. C. Lainez, C. Pelucchi, E. Negri, R. Bonzi, D. Palli, M. Ferraroni, Z. F. Zhang, G. P. Yu, N. Lunet, S. Morais, L. Lopez-Carrillo, D. Zaridze, D. Maximovitch, M. Guevara, V. Santos-Sanchez, J. Vioque, M. G. de la Hera, M. H. Ward, R. Malekzadeh, M. Pakseresht, R. U. Hernández-Ramirez, F. Turati, C. S. Rabkin, L. M. Liao, R. Sinha, M. López-Cervantes, S. Tsugane, A. Hidaka, M. C. Camargo, M. P. Curado, N. Zubair, D. Kristjansson, S. Shah, C. La Vecchia and P. Boffetta | 2024 | The association between dietary fiber intake and gastric cancer: a pooled analysis of 11 case-control studies | European Journal of Nutrition | 10.1007/s00394-024-03388-w |
| **9** | G. Collatuzzo, C. Pelucchi, E. Negri, M. Kogevinas, J. M. Huerta, J. Vioque, M. G. de la Hera, S. Tsugane, G. S. Hamada, A. Hidaka, Z. F. Zhang, M. C. Camargo, M. P. Curado, N. Lunet, C. La Vecchia and P. Boffetta | 2023 | Sleep Duration and Stress Level in the Risk of Gastric Cancer: A Pooled Analysis of Case-Control Studies in the Stomach Cancer Pooling (StoP) Project | Cancers | 10.3390/cancers15174319 |
| **10** | G. Collatuzzo, C. Pelucchi, E. Negri, L. Lopez-Carrillo, S. Tsugane, A. Hidaka, G. S. Hamada, R. U. Hernandez-Ramirez, M. Lopez-Cervantes, R. Malekzadeh, F. Pourfarzi, L. N. Mu, Z. F. Zhang, N. Lunet, C. La Vecchia and P. Boffetta | 2021 | Exploring the interactions between Helicobacter pylori (Hp) infection and other risk factors of gastric cancer: A pooled analysis in the Stomach cancer Pooling (StoP) Project | International Journal of Cancer | 10.1002/ijc.33678 |
| **11** | G. Collatuzzo, F. Teglia, C. Pelucchi, E. Negri, C. S. Rabkin, L. M. Liao, R. Sinha, L. López-Carrillo, N. Lunet, S. Morais, N. Aragonés, V. Moreno, J. Vioque, M. G. de la Hera, M. H. Ward, R. Malekzadeh, M. Pakseresht, R. U. Hernández-Ramírez, M. López-Cervantes, R. Bonzi, M. Dalmartello, S. Tsugane, A. Hidaka, M. C. Camargo, M. P. Curado, Z. F. Zhang, N. Zubair, C. La Vecchia, S. Shah and P. Boffetta | 2022 | Inverse Association between Dietary Iron Intake and Gastric Cancer: A Pooled Analysis of Case-Control Studies of the Stop Consortium | Nutrients | 10.3390/nu14122555 |
| **12** | M. B. Cook, D. A. Corley, L. J. Murray, L. M. Liao, F. Kamangar, W. M. Ye, M. D. Gammon, H. A. Risch, A. G. Casson, N. D. Freedman, W. H. Chow, A. H. Wu, L. Bernstein, O. Nyrén, N. Pandeya, D. C. Whiteman and T. L. Vaughan | 2014 | Gastroesophageal Reflux in Relation to Adenocarcinomas of the Esophagus: A Pooled Analysis from the Barrett's and Esophageal Adenocarcinoma Consortium (BEACON) | Plos One | 10.1371/journal.pone.0103508 |
| **13** | B. Dabo, C. Pelucchi, M. Rota, H. Jain, P. Bertuccio, R. Bonzi, D. Palli, M. Ferraroni, Z. F. Zhang, A. Sanchez-Anguiano, Y. T. H. Pham, C. T. D. Tran, A. G. Pham, G. P. Yu, T. C. Nguyen, J. Muscat, S. Tsugane, A. Hidaka, G. S. Hamada, D. Zaridze, D. Maximovitch, M. Kogevinas, N. F. de Larrea, S. Boccia, R. Pastorino, R. C. Kurtz, A. Lagiou, P. Lagiou, J. Vioque, M. C. Camargo, M. P. Curado, N. Lunet, P. Boffetta, E. Negri, C. La Vecchia and H. N. Luu | 2022 | The association between diabetes and gastric cancer: results from the Stomach Cancer Pooling Project Consortium | European Journal of Cancer Prevention | 10.1097/cej.0000000000000703 |
| **14** | H. Y. Ding, S. Fan, L. Zhang, Z. Y. Hao and C. Z. Liang | 2017 | Does prostatitis increase the risk of prostate cancer? A meta-analysis | International Journal of Clinical and Experimental Medicine |  |
| **15** | P. Dobsch, A. Mehrl and A. Kandulski | 2021 | Gastrointestinal endoscopy in geriatric patients | Gastroenterologe | 10.1007/s11377-021-00550-2 |
| **16** | J. Drahos, Q. Xiao, H. A. Risch, N. D. Freedman, C. C. Abnet, L. A. Anderson, L. Bernstein, L. Brown, W. H. Chow, M. D. Gammon, F. Kamangar, L. M. Liao, L. J. Murray, M. H. Ward, W. M. Ye, A. H. Wu, T. L. Vaughan, D. C. Whiteman and M. B. Cook | 2016 | Age-specific risk factor profiles of adenocarcinomas of the esophagus: A pooled analysis from the international BEACON consortium | International Journal of Cancer | 10.1002/ijc.29688 |
| **17** | A. Etemadi, S. Safiri, S. G. Sepanlou, K. Ikuta, C. Bisignano, R. Shakeri, M. Amani, C. Fitzmaurice, M. R. Nixon, N. Abbasi, H. Abolhassani, S. M. Advani, M. Afarideh, T. Akinyemiju, T. Alam, M. Alikhani, V. Alipour, C. A. Allen, A. Almasi-Hashiani, J. Arabloo, R. Assadi, S. Atique, A. Awasthi, A. Bakhtiari, M. Behzadifar, K. Berhe, N. Bhala, A. Bijani, M. S. Bin Sayeed, T. Bjorge, A. M. Borzì, D. Braithwaite, H. Brenner, G. Carreras, F. Carvalho, C. A. Castañeda-Orjuela, F. Castro, D. T. Chu, V. M. Costa, A. Daryani, D. V. Davitoiu, G. T. Demoz, A. B. Demis, E. Denova-Gutiérrez, S. Dey, M. D. Nasab, S. Djalalinia, M. H. Emamian, M. Farahmand, J. C. Fernandes, F. Fischer, M. Foroutan, M. M. Gad, S. Gallus, G. G. Gebremeskel, G. A. Gedefew, F. Ghaseni-Kebria, G. Gorini, N. Hafezi-Nejad, A. Haj-Mirzaian, J. M. Haro, J. D. Harvey, A. Hasanzadeh, M. Hashemian, H. Y. Hassen, S. I. Hay, H. D. Hidru, M. Hostiuc, M. Househ, O. S. Ilesanmi, M. D. Ilic, K. Innos, F. Islami, S. L. James, E. Jenabi, R. Kalhor, F. Kamangar, A. Kasaeian, A. P. Kengne, Y. S. Khader, R. Khalilov, E. A. Khan, G. Khan, M. Khayamzadeh, M. Khazaee-Pool, S. Khazaei, A. T. Khoja, F. Khosravi Shadmani, Y. J. Kim, J. M. Kocarnik, H. Komaki, A. Koyanagi, V. Kumar, C. La Vecchia, A. D. Lopez, R. Lunevicius, N. Manafi, A. L. Manda, B. Geta, H. Meheretu, G. Mengistu, B. Miazgowski, S. M. Mir, K. A. Mohammad, N. M. G. Mezerji, M. Mohammadian, A. Mohammadian-Hafshejani, R. Mohammadpourhodki, S. Mohammed, F. Mohebi, A. H. Mokdad, L. Monasta, M. Moosazadeh, M. Moossavi, G. Moradi, F. Moradpour, R. Moradzadeh, I. M. Velasquez, A. Mosapour, M. Naderi, G. Naik, F. Najafi, A. Nahvijou, I. Negoi, R. Nikbakhsh, M. Nojomi, A. T. Olagunju, T. O. Olagunju, E. Oren, H. Parsian, C. Piccinelli, A. Pourshams, H. Poustchi, N. Rabiee, A. Radfar, A. Rafiei, M. Rahimi, M. Rahmati, A. M. N. Renzaho, N. Rezaei, A. I. Ribeiro, G. Roshandel, A. M. Saad, S. Saadatagah, H. Salimzadeh, A. M. Samy, J. Sanabria, M. M. S. Milicevic, A. Sarveazad, M. Sawhney, F. Shaahmadi, M. Sekerija, M. A. Shaikh, A. Shamshirian, S. K. S. Malleshappa, J. A. Singh, C. G. Smarandache, M. Soofi, T. Tabuchi, D. B. B. Tadesse, L. Tapak, B. E. Tesfay, E. Traini, B. Tran, K. B. Tran, M. Vacante, A. Vahedian-Azimi, Y. Veisani, K. Vosoughi, I. S. Vujcic, R. Westerman, A. B. Wondmieneh, R. X. Xu, S. Yaya, V. Yazdi-Feyzabadi, Z. Yousefi, B. Yousefi, T. Z. Moghadam, L. Zaki, M. Zamani, M. Zamanian, H. Zandian, A. Zarghi, Z. J. Zhang, M. Naghavi, R. Malekzadeh and G. B. D. S. C. Collaborator | 2020 | The global, regional, and national burden of stomach cancer in 195 countries, 1990-2017: a systematic analysis for the Global Burden of Disease study 2017 | Lancet Gastroenterology & Hepatology | 10.1016/s2468-1253(19)30328-0 |
| **18** | Y. G. Fan, Y. Jiang, L. Gong, Y. Wang, Z. Su, X. B. Li, H. Wu, H. L. Pan, J. Wang, Z. W. Meng, Q. H. Zhou and Y. L. Qiao | 2023 | Epidemiological and demographic drivers of lung cancer mortality from 1990 to 2019: results from the global burden of disease study 2019 | Frontiers in Public Health | 10.3389/fpubh.2023.1054200 |
| **19** | A. Ferro, S. Morais, C. Pelucchi, N. Aragonés, M. Kogevinas, L. López-Carrillo, R. Malekzadeh, S. Tsugane, G. S. Hamada, A. Hidaka, R. U. Hernández-Ramírez, M. López-Cervantes, D. Zaridze, D. Maximovitch, F. Pourfarzi, Z. F. Zhang, G. P. Yu, M. Pakseresht, W. M. Ye, A. Plymoth, M. Leja, E. Gasenko, M. H. Derakhshan, E. Negri, C. La Vecchia, B. Peleteiro and N. Lunet | 2019 | Smoking and <i>Helicobacter pylori</i> infection: an individual participant pooled analysis (Stomach Cancer Pooling- StoP Project) | European Journal of Cancer Prevention | 10.1097/cej.0000000000000471 |
| **20** | A. Ferro, S. Morais, C. Pelucchi, T. Dierssen-Sotos, V. Martín, L. López-Carrillo, R. Malekzadeh, S. Tsugane, G. S. Hamada, A. Hidaka, R. U. Hernández-Ramírez, M. López-Cervantes, D. Zaridze, D. Maximovitch, F. Pourfarzi, Z. F. Zhang, G. P. Yu, M. Pakseresht, W. M. Ye, A. Plymoth, M. Leja, E. Gasenko, M. H. Derakhshan, E. Negri, C. La Vecchia, B. Peleteiro and N. Lunet | 2019 | Sex differences in the prevalence of Helicobacter pylori infection: an individual participant data pooled analysis (StoP Project) | European Journal of Gastroenterology & Hepatology | 10.1097/meg.0000000000001389 |
| **21** | A. Ferro, S. Morais, M. Rota, C. Pelucchi, P. Bertuccio, R. Bonzi, C. Galeone, Z. F. Zhang, K. Matsuo, H. Ito, J. F. Hu, K. C. Johnson, G. P. Yu, D. Palli, M. Ferraroni, J. Muscat, R. Malekzadeh, W. M. Ye, H. Song, D. Zaridze, D. Maximovitch, N. Aragonés, G. Castaño-Vinyals, J. Vioque, E. M. Navarrete-Muñoz, M. Pakseresht, F. Pourfarzi, A. Wolk, N. Orsini, A. Bellavia, N. Håkansson, L. N. Mu, R. Pastorino, R. C. Kurtz, M. H. Derakhshan, A. Lagiou, P. Lagiou, P. Boffetta, S. Boccia, E. Negri, C. La Vecchia, B. Peleteiro and N. Lunet | 2018 | Tobacco smoking and gastric cancer: meta-analyses of published data versus pooled analyses of individual participant data (StoP Project) | European Journal of Cancer Prevention | 10.1097/cej.0000000000000401 |
| **22** | A. Ferro, S. Morais, M. Rota, C. Pelucchi, P. Bertuccio, R. Bonzi, C. Galeone, Z. F. Zhang, K. Matsuo, H. Ito, J. F. Hu, K. C. Johnson, G. P. Yu, D. Palli, M. Ferraroni, J. Muscat, R. Malekzadeh, W. M. Ye, H. Song, D. Zaridze, D. Maximovitch, N. F. de Larrea, M. Kogevinas, J. Vioque, E. M. Navarrete-Muñoz, M. Pakseresht, F. Pourfarzi, A. Wolk, N. Orsini, A. Bellavia, N. Håkansson, L. N. Mu, R. Pastorino, R. C. Kurtz, M. H. Derakhshan, A. Lagiou, P. Lagiou, P. Boffetta, S. Boccia, E. Negri, C. La Vecchia, B. Peleteiro and N. Lunet | 2018 | Alcohol intake and gastric cancer: Meta-analyses of published data versus individual participant data pooled analyses (StoP Project) | Cancer Epidemiology | 10.1016/j.canep.2018.04.009 |
| **23** | C. Fitzmaurice, C. Allen, R. M. Barber, L. Barregard, Z. A. Bhutta, H. Brenner, D. J. Dicker, O. Chimed-Orchir, R. Dandona, L. Dandona, T. Fleming, M. H. Forouzanfar, J. Hancock, R. J. Hay, R. Hunter-Merrill, C. Huynh, H. D. Hosgood, C. O. Johnson, J. B. Jonas, J. Khubchandani, G. A. Kumar, M. Kutz, Q. Lan, H. J. Larson, X. F. Liang, S. S. Lim, A. D. Lopez, M. F. MacIntyre, L. Marczak, N. Marquez, A. H. Mokdad, C. Pinho, F. Pourmalek, J. A. Salomon, J. R. Sanabria, L. Sandar, B. Sartorius, S. M. Schwartz, K. A. Shackelford, K. Shibuya, J. Stanaway, C. Steiner, J. D. Sun, K. Takahashi, S. E. Vollset, T. Vos, J. A. Wagner, H. D. Wang, R. Westerman, H. Zeeb, L. Zoeckler, F. Abd-Allah, M. B. Ahmed, S. Alabed, N. K. Alam, S. F. Aldhahri, G. Alem, M. A. Alemayohu, R. Ali, R. Al-Raddadi, A. Amare, Y. Amoako, A. Artaman, H. Asayesh, N. Atnafu, A. Awasthi, H. B. Saleem, A. Barac, N. Bedi, I. Bensenor, A. Berhane, E. Bemabé, B. Betsu, A. Binagwaho, D. Boneya, I. Campos-Nonato, C. Castañeda-Orjuela, F. Catalá-López, P. Chiang, C. Chibueze, A. Chitheer, J. Y. Choi, B. Cowie, S. Damtew, J. das Neves, S. Dey, S. Dharmaratne, P. Dhillon, E. Ding, T. Driscoll, D. Ekwueme, A. Y. Endries, M. Farvid, F. Farzadfar, J. Fernandes, F. Fischer, T. T. Ghiwot, A. Gebru, S. Gopalani, A. Hailu, M. Horino, N. Horita, A. Husseini, I. Huybrechts, M. Inoue, F. Islami, M. Jakovljevic, S. James, M. Javanbakht, S. H. Jee, A. Kasaeian, M. S. Kedir, Y. S. Khader, Y. H. Khang, D. Kim, J. Leigh, S. Linn, R. Lunevicius, H. M. Abd El Razek, R. Malekzadeh, D. C. Malta, W. Marcenes, D. Markos, Y. A. Melaku, K. G. Meles, W. Mendoza, D. T. Mengiste, T. J. Meretoja, T. R. Miller, K. A. Mohammad, A. Mohammadi, S. Mohammed, M. Moradi-Lakeh, G. Nagel, D. Nand, Q. Le Nguyen, S. Nolte, F. A. Ogbo, K. E. Oladimeji, E. Oren, M. Pa, E. K. Park, D. M. Pereira, D. Plass, M. Qorbani, A. Radfar, A. Rafay, M. Rahman, S. M. Rana, K. Soreide, M. Satpathy, M. Sawhney, S. G. Sepanlou, M. A. Shaikh, J. She, I. Shiue, H. R. Shore, M. G. Shrime, S. So, S. Soneji, V. Stathopoulou, K. Stroumpoulis, M. B. Sufiyan, B. L. Sykes, R. Tabares-Seisdedos, F. Tadese, B. A. Tedla, G. A. Tessema, J. S. Thakur, B. X. Tran, K. N. Ukwaja, B. S. C. Uzochukwu, V. V. Vlassov, E. Weiderpass, M. W. Terefe, H. G. Yebyo, H. H. Yimam, N. Yonemoto, M. Z. Younis, C. H. Yu, Z. Zaidi, M. E. Zaki, Z. M. Zenebe, C. J. L. Murray, M. Naghavi and C. Global Bourden Disease Cancer | 2017 | Global, Regional, and National Cancer Incidence, Mortality, Years of Life Lost, Years Lived With Disability, and Disability-Adjusted Life-years for 32 Cancer Groups, 1990 to 2015 A Systematic Analysis for the Global Burden of Disease Study | Jama Oncology | 10.1001/jamaoncol.2016.5688 |
| **24** | T. Y. Gao, Y. T. Tao, H. Y. Li, X. Liu, Y. T. Ma, H. J. Li, C. Y. Xian-Yu, N. J. Deng, W. D. Leng, J. Luo and C. Zhang | 2024 | Cancer burden and risk in the Chinese population aged 55 years and above: A systematic analysis and comparison with the USA and Western Europe | Journal of Global Health | 10.7189/jogh.14.04014 |
| **25** | N. Garg, C. Stoehr, Y. S. Zhao, H. Rojas and C. T. Hsueh | 2017 | Metastatic squamous cell carcinoma of colon from esophageal cancer | Experimental Hematology & Oncology | 10.1186/s40164-017-0069-2 |
| **26** | G. F. Gil, J. A. Anderson, A. Aravkin, K. Bhangdia, S. Carr, X. C. Dai, L. S. Flor, S. I. Hay, M. J. Malloy, S. A. McLaughlin, E. C. Mullany, C. J. L. Murray, E. M. O'Connell, C. Okereke, R. J. D. Sorensen, J. Whisnant, P. Zheng and E. Gakidou | 2024 | Health effects associated with chewing tobacco: a Burden of Proof study | Nature Communications | 10.1038/s41467-024-45074-9 |
| **27** | L. Giraldi, J. Stojanovic, D. Arzani, R. Persiani, J. F. Hu, K. C. Johnson, Z. F. Zhang, M. Ferraroni, D. Palli, G. P. Yu, C. La Vecchia, C. Pelucchi, N. Lunet, A. Ferro, R. Malekzadeh, J. Muscat, D. Zaridze, D. Maximovich, N. Aragones, V. Martin, J. Vioque, E. M. Navarrete-Munoz, M. Pakseresht, E. Negri, M. Rota, F. Pourfarzi, L. N. Mu, R. C. Kurtz, A. Lagiou, P. Lagiou, R. Pastorino and S. Boccia | 2023 | Adult height and risk of gastric cancer: a pooled analysis within the Stomach cancer Pooling Project | European Journal of Cancer Prevention | 10.1097/cej.0000000000000613 |
| **28** | T. Grantham, R. Ramachandran, S. Parvataneni, D. Budh, S. Gollapalli and V. Gaduputi | 2023 | Epidemiology of Gastric Cancer: Global Trends, Risk Factors and Premalignant Conditions | Journal of Community Hospital Internal Medicine Perspectives | 10.55729/2000-9666.1252 |
| **29** | S. Hermann and V. Arndt | 2017 | Cancer - a global challenge | Onkologe | 10.1007/s00761-017-0287-6 |
| **30** | S. Jain and S. Dhingra | 2017 | Pathology of esophageal cancer and Barrett's esophagus | Annals of Cardiothoracic Surgery | 10.21037/acs.2017.03.06 |
| **31** | H. Jayasekara, R. J. MacInnis, L. Lujan-Barroso, A. L. Mayen-Chacon, A. J. Cross, B. Wallner, D. Palli, F. Ricceri, V. Pala, S. Panico, R. Tumino, T. Kühn, R. Kaaks, K. Tsilidis, M. J. Sánchez, P. Amiano, E. Ardanaz, M. D. C. López, S. Merino, J. A. Rothwell, M. C. Boutron-Ruault, G. Severi, H. Sternby, E. Sonestedt, B. Bueno-de-Mesquita, H. Boeing, R. Travis, T. M. Sandanger, A. Trichopoulou, A. Karakatsani, E. Peppa, A. Tjonneland, Y. Yang, A. M. Hodge, H. Mitchell, A. Haydon, R. Room, J. L. Hopper, E. Weiderpass, M. J. Gunter, E. Riboli, G. G. Giles, R. L. Milne, A. Agudo, D. R. English and P. Ferrari | 2021 | Lifetime alcohol intake, drinking patterns over time and risk of stomach cancer: A pooled analysis of data from two prospective cohort studies | International Journal of Cancer | 10.1002/ijc.33504 |
| **32** | Y. S. Jung and S. J. Yoon | 2022 | Burden of Cancer Due to Cigarette Smoking and Alcohol Consumption in Korea | International Journal of Environmental Research and Public Health | 10.3390/ijerph19063493 |
| **33** | J. Keller, G. Bassotti, J. Clarke, P. Dinning, M. Fox, M. Grover, P. M. Hellström, M. Y. Ke, P. Layer, C. Malagelada, H. P. Parkman, S. M. Scott, J. Tack, M. Simren, H. Törnblom, M. Camilleri and G. Int Working Grp Disorders | 2018 | Advances in the diagnosis and classification of gastric and intestinal motility disorders | Nature Reviews Gastroenterology & Hepatology | 10.1038/nrgastro.2018.7 |
| **34** | J. Labenz, H. Koop, A. Tannapfel, R. Kiesslich and A. H. Hölscher | 2015 | The Epidemiology, Diagnosis, and Treatment of Barrett's Carcinoma | Deutsches Arzteblatt International | 10.3238/arztebl.2015.0224 |
| **35** | C. Lesseur, A. Ferreiro-Iglesias, J. D. McKay, Y. Bossé, M. Johansson, V. Gaborieau, M. T. Landi, D. C. Christiani, N. C. Caporaso, S. E. Bojesen, C. I. Amos, S. Shete, G. Liu, G. Rennert, D. Albanes, M. C. Aldrich, A. Tardon, C. Chen, L. Triantafillos, J. K. Field, M. D. Teare, L. A. Kiemeney, B. Diergaarde, R. L. Ferris, S. Zienolddiny, S. Lam, A. F. Olshan, M. C. Weissler, M. Lacko, A. Risch, H. Bickeböller, A. R. Ness, S. Thomas, L. Le Marchand, M. B. Schabath, V. Wünsch, E. H. Tajara, A. S. Andrew, G. M. Clifford, P. Lazarus, K. Grankvist, M. Johansson, S. Arnold, O. Melander, H. Brunnström, S. Boccia, G. Cadoni, W. Timens, M. Obeidat, X. J. Xiao, R. S. Houlston, R. Y. J. Hung and P. Brennan | 2021 | Genome-wide association meta-analysis identifies pleiotropic risk loci for aerodigestive squamous cell cancers | Plos Genetics | 10.1371/journal.pgen.1009254 |
| **36** | Q. M. Li, L. Y. Zhu, T. Wei, Z. P. Zang, X. R. Zhang, Y. J. Wang, R. Gao, Y. J. Zhang, X. T. Zheng and F. Liu | 2023 | Secular trends and attributable risk factors of esophageal cancer deaths among non-elderly adults based on Global Burden of Disease Study | Journal of Cancer Research and Clinical Oncology | 10.1007/s00432-023-05380-z |
| **37** | S. B. Li, H. Chen, J. Y. Man, T. C. Zhang, X. L. Yin, Q. F. He, X. R. Yang and M. Lu | 2021 | Changing trends in the disease burden of esophageal cancer in China from 1990 to 2017 and its predicted level in 25 years | Cancer Medicine | 10.1002/cam4.3775 |
| **38** | L. L. Lu, C. S. Mullins, C. Schafmayer, S. Zeissig and M. Linnebacher | 2021 | A global assessment of recent trends in gastrointestinal cancer and lifestyle-associated risk factors | Cancer Communications | 10.1002/cac2.12220 |
| **39** | C. Ma, S. E. Congly, D. E. Chyou, K. Ross-Driscoll, N. Forbes, E. S. Tsang, D. A. Sussman and D. S. Goldberg | 2022 | Factors Associated With Geographic Disparities in Gastrointestinal Cancer Mortality in the United States | Gastroenterology | 10.1053/j.gastro.2022.04.019 |
| **40** | N. M. Mansour, H. B. El-Serag and S. Anandasabapathy | 2017 | Barrett's esophagus: best practices for treatment and post-treatment surveillance | Annals of Cardiothoracic Surgery | 10.21037/acs.2017.03.05 |
| **41** | A. Mantovani, G. Petracca, G. Beatrice, A. Csermely, H. Tilg, C. D. Byrne and G. Targher | 2022 | Non-alcoholic fatty liver disease and increased risk of incident extrahepatic cancers: a meta-analysis of observational cohort studies | Gut | 10.1136/gutjnl-2021-324191 |
| **42** | N. Mao, S. Y. Nie, B. Hong, C. Li, X. Y. Shen and T. Xiong | 2016 | Association between alcohol dehydrogenase-2 gene polymorphism and esophageal cancer risk: a meta-analysis | World Journal of Surgical Oncology | 10.1186/s12957-016-0937-y |
| **43** | N. Mohy-ud-Din, T. S. Krill, A. R. Shah, A. T. Chatila, S. Singh, M. Bilal and S. Parupudi | 2020 | Barrett's esophagus: What do we need to know? | Dm Disease-a-Month | 10.1016/j.disamonth.2019.02.003 |
| **44** | G. Nagel, M. Stafoggia, M. Pedersen, Z. J. Andersen, C. Galassi, J. Munkenast, A. Jaensch, J. Sommar, B. Forsberg, D. Olsson, B. Oftedal, N. H. Krog, G. Aamodt, A. Pyko, G. Pershagen, M. Korek, U. De Faire, N. L. Pedersen, C. G. Östenson, L. Fratiglioni, M. Sorensen, A. Tjonneland, P. H. Peeters, B. Bueno-de-Mesquita, R. Vermeulen, M. Eeftens, M. Plusquin, T. J. Key, H. Concin, A. Lang, M. Wang, M. Y. Tsai, S. Grioni, A. Marcon, V. Krogh, F. Ricceri, C. Sacerdote, A. Ranzi, G. Cesaroni, F. Forastiere, I. Tamayo-Uria, P. Amiano, M. Dorronsoro, K. de Hoogh, R. Beelen, P. Vineis, B. Brunekreef, G. Hoek, O. Raaschou-Nielsen and G. Weinmayr | 2018 | Air pollution and incidence of cancers of the stomach and the upper aerodigestive tract in the European Study of Cohorts for Air Pollution Effects (ESCAPE) | International Journal of Cancer | 10.1002/ijc.31564 |
| **45** | E. Ness-Jensen, K. Hveem, H. El-Serag and J. Lagergren | 2016 | Lifestyle Intervention in Gastroesophageal Reflux Disease | Clinical Gastroenterology and Hepatology | 10.1016/j.cgh.2015.04.176 |
| **46** | E. Ness-Jensen and J. Lagergren | 2017 | Tobacco smoking, alcohol consumption and gastro-oesophageal reflux disease | Best Practice & Research Clinical Gastroenterology | 10.1016/j.bpg.2017.09.004 |
| **47** | F. L. Ning, J. Lyu, J. P. Pei, W. J. Gu, N. N. Zhang, S. Y. Cao, Y. J. Zeng, M. Abe, K. Nishiyama and C. D. Zhang | 2022 | The burden and trend of gastric cancer and possible risk factors in five Asian countries from 1990 to 2019 | Scientific Reports | 10.1038/s41598-022-10014-4 |
| **48** | A. Perisetti and P. Sharma | 2023 | A Practical Approach to Diagnosis and Treatment of Barrett's Esophagus | Practical Gastroenterology |  |
| **49** | J. L. Petrick, N. Li, L. A. Anderson, L. Bernstein, D. A. Corley, H. B. El Serag, S. Hardikar, L. M. Liao, G. Liu, L. J. Murray, J. H. Rubenstein, J. L. Schneider, N. J. Shaheen, A. P. Thrift, P. A. van den Brandt, T. L. Vaughan, D. C. Whiteman, A. H. Wu, W. K. Zhao, M. D. Gammon and M. B. Cook | 2019 | Diabetes in relation to Barrett's esophagus and adenocarcinomas of the esophagus: A pooled study from the International Barrett's and Esophageal Adenocarcinoma Consortium | Cancer | 10.1002/cncr.32444 |
| **50** | A. Prabhu, K. Obi, D. Lieberman and J. H. Rubenstein | 2016 | The Race-Specific Incidence of Esophageal Squamous Cell Carcinoma in Individuals With Exposure to Tobacco and Alcohol | American Journal of Gastroenterology | 10.1038/ajg.2016.346 |
| **51** | D. Praud, M. Rota, C. Pelucchi, P. Bertuccio, T. Rosso, C. Galeone, Z. F. Zhang, K. Matsuo, H. Ito, J. F. Hu, K. C. Johnson, G. P. Yu, D. Palli, M. Ferraroni, J. Muscat, N. Lunet, B. Peleteiro, R. Malekzadeh, W. M. Ye, H. Song, D. Zaridze, D. Maximovitch, N. Aragonés, G. Castaño-Vinyals, J. Vioque, E. M. Navarrete-Muñoz, M. Pakseresht, F. Pourfarzi, A. Wolk, N. Orsini, A. Bellavia, N. Håkansson, L. N. Mu, R. Pastorino, R. C. Kurtz, M. H. Derakhshan, A. Lagiou, P. Lagiou, P. Boffetta, S. Boccia, E. Negri and C. La Vecchia | 2018 | Cigarette smoking and gastric cancer in the Stomach Cancer Pooling (StoP) Project | European Journal of Cancer Prevention | 10.1097/cej.0000000000000290 |
| **52** | H. B. Qiu, S. M. Cao and R. H. Xu | 2021 | Cancer incidence, mortality, and burden in China: a time-trend analysis and comparison with the United States and United Kingdom based on the global epidemiological data released in 2020 | Cancer Communications | 10.1002/cac2.12197 |
| **53** | M. Ravanbakhsh, H. Yousefi, E. Lak, M. J. Ansari, W. Suksatan, Q. A. Qasim, P. Asban, M. Kianizadeh and M. J. Mohammadi | 2023 | Effect of Polycyclic Aromatic Hydrocarbons (PAHs) on Respiratory Diseases and the Risk Factors Related to Cancer | Polycyclic Aromatic Compounds | 10.1080/10406638.2022.2149569 |
| **54** | A. S. Reece and G. K. Hulse | 2022 | Geotemporospatial and causal inferential epidemiological overview and survey of USA cannabis, cannabidiol and cannabinoid genotoxicity expressed in cancer incidence 2003-2017: part 1-continuous bivariate analysis | Archives of Public Health | 10.1186/s13690-022-00811-8 |
| **55** | A. S. Reece and G. K. Hulse | 2022 | Geotemporospatial and causal inferential epidemiological overview and survey of USA cannabis, cannabidiol and cannabinoid genotoxicity expressed in cancer incidence 2003-2017: part 2-categorical bivariate analysis and attributable fractions | Archives of Public Health | 10.1186/s13690-022-00812-7 |
| **56** | F. P. Ren, Z. L. Shi, X. Shen, G. F. Xiao, C. Y. Zhang and Y. Q. Cheng | 2024 | The global, regional, and national burden of stomach cancer attributed to smoking in 204 countries, 1990-2019: A systematic analysis for the Global Burden of Disease Study 2019 | Tobacco Induced Diseases | 10.18332/tid/183803 |
| **57** | J. E. Richter and J. H. Rubenstein | 2018 | Presentation and Epidemiology of Gastroesophageal Reflux Disease | Gastroenterology | 10.1053/j.gastro.2017.07.045 |
| **58** | C. B. Rim and S. M. Kim | 2020 | Phlegmonous Gastritis Caused by Penetration of a Toothpick | Korean Journal of Gastroenterology | 10.4166/kjg.2020.75.3.157 |
| **59** | M. Rota, G. Alicandro, C. Pelucchi, R. Bonzi, P. Bertuccio, J. F. Hu, Z. F. Zhang, K. C. Johnson, D. Palli, M. Ferraroni, G. P. Yu, C. Galeone, L. López-Carrillo, J. Muscat, N. Lunet, A. Ferro, W. M. Ye, A. Plymoth, R. Malekzadeh, D. Zaridze, D. Maximovitch, M. Kogevinas, N. F. de Larrea, J. Vioque, E. M. Navarrete-Muñoz, S. Tsugane, G. S. Hamada, A. Hidaka, M. Pakseresht, A. Wolk, N. Hakansson, R. U. Hernández-Ramírez, M. López-Cervantes, M. Ward, F. Pourfarzi, L. Mu, R. C. Kurtz, A. Lagiou, P. Lagiou, P. Boffetta, S. Boccia, E. Negri and C. La Vecchia | 2020 | Education and gastric cancer risk-An individual participant data meta-analysis in the StoP project consortium | International Journal of Cancer | 10.1002/ijc.32298 |
| **60** | T. M. Runge, J. A. Abrams and N. J. Shaheen | 2015 | Epidemiology of Barrett's Esophagus and Esophageal Adenocarcinoma | Gastroenterology Clinics of North America | 10.1016/j.gtc.2015.02.001 |
| **61** | S. S. Sami, K. Ragunath and P. G. Iyer | 2015 | PERSPECTIVES IN CLINICAL GASTROENTEROLOGY AND HEPATOLOGY | Clinical Gastroenterology and Hepatology | 10.1016/j.cgh.2014.03.036 |
| **62** | R. Sharma | 2024 | Burden of Stomach Cancer Incidence, Mortality, Disability-Adjusted Life Years, and Risk Factors in 204 Countries, 1990-2019: An Examination of Global Burden of Disease 2019 | Journal of Gastrointestinal Cancer | 10.1007/s12029-023-01005-3 |
| **63** | R. Sharma, H. Abbastabar, D. M. Abdulah, H. Abidi, H. Abolhassani, Z. Abrehdari-Tafreshi, A. Absalan, H. A. Ali, E. Abu-Gharbieh, J. M. Acuna, N. Adib, Q. E. S. Adnani, A. Aghaei, A. Ahmad, S. Ahmad, A. Ahmadi, S. Ahmadi, L. A. Ahmed, M. Ajami, H. Al Hamad, S. M. Al Hasan, F. M. Alanezi, A. A. S. Al-Gheethi, M. K. Al-Hanawi, A. Ali, B. A. Ali, Y. Alimohamadi, S. M. Aljunid, S. A. A. Al-Maweri, S. A. Alqahatni, M. AlQudah, R. M. Al-Raddadi, A. B. Al-Tammemi, A. Ansari-Moghaddam, S. L. Anwar, R. Anwer, M. Aqeel, J. Arabloo, M. Arab-Zozani, H. Ariffin, A. Artaman, J. Arulappan, T. Ashraf, E. Askari, M. Athar, M. M. W. Atout, S. Azadnajafabad, M. Badar, A. D. Badiye, N. Baghcheghi, S. Bagherieh, R. H. Bai, K. Bajbouj, S. Baliga, M. Bardhan, A. Bashiri, P. Baskaran, S. Basu, U. I. Belgaumi, A. N. C. Bermudez, B. Bhandari, N. Bhardwaj, A. N. Bhat, S. Bitaraf, A. Boloor, M. B. Hashemi, Z. A. Butt, J. Chadwick, J. S. K. Chan, V. K. Chattu, P. Chaturvedi, W. C. S. Cho, A. M. Darwesh, N. R. Dash, A. Dehghan, A. Dhali, M. Dianatinasab, M. Dibas, A. Dixit, S. G. Dixit, F. Dorostkar, H. L. Dsouza, I. Elbarazi, N. M. Elemam, W. El-Huneidi, E. Elkord, O. A. A. Elmeligy, M. H. Emamian, R. Erkhembayar, R. Ezzeddini, Z. Fadoo, R. Faiz, I. R. Fakhradiyev, A. Fallahzadeh, M. E. M. Faris, H. Farrokhpour, A. Fatehizadeh, H. Fattahi, G. Fekadu, T. Fukumoto, A. M. Gaidhane, N. Galehdar, P. Garg, F. Ghadirian, M. Ghafourifard, M. Ghasemi, M. G. Nour, F. Ghassemi, M. Gholamalizadeh, A. Gholamian, E. Ghotbi, M. Golechha, P. Goleij, S. Goyal, M. I. M. Gubari, D. S. Gunasekera, D. A. Gunawardane, S. Gupta, P. Habibzadeh, H. S. H. Boroojeni, E. S. Halboub, R. R. Hamadeh, R. Hamoudi, M. Harorani, M. Hasanian, T. S. Hassan, S. Hay, M. Heidari, M. Heidari-Foroozan, K. Hessami, K. Hezam, Y. Hiraike, R. Holla, M. Hoseini, M. M. Hossain, S. Hossain, V. C. R. Hsieh, J. J. Huang, N. R. Hussein, B. F. Hwang, F. Iravanpour, N. E. Ismail, M. Iwagami, J. L. Merin, F. Jadidi-Niaragh, M. Jafarinia, M. A. Jahani, H. Jahrami, A. Jaiswal, M. Jakovljevic, M. Jalili, E. Jamshidi, U. Jayarajah, S. Jayaram, S. S. Jha, M. Jokar, N. Joseph, A. Kabir, M. A. Kabir, D. H. Kadir, P. V. Kakodkar, L. R. Kalankesh, L. R. Kalankesh, R. Kalhor, F. Kaliyadan, V. K. Kamal, Z. Kamal, A. Kamath, S. S. Kar, H. Karimi, N. Kaur, L. Keikavoosi-Arani, M. Keykhaei, Y. S. Khader, H. Khajuria, E. A. Khan, M. N. Khan, M. Khan, M. A. B. Khan, Y. H. Khan, S. Khanmohammadi, M. M. Khatatbeh, S. Khateri, M. Khayamzadeh, H. R. K. Kashani, M. S. Kim, F. Kompani, H. R. Koohestani, S. L. K. Laxminarayana, K. Krishan, N. Kumar, N. Kumar, T. Kutluk, A. Kuttikkattu, D. T. C. Lai, D. K. Lal, F. H. Lami, S. Lasrado, S. W. Lee, S. W. Lee, Y. Y. Lee, Y. H. Lee, E. Leong, M. C. Li, J. Liu, F. Madadizadeh, A. R. Mafi, S. Mahjoub, R. Malekzadeh, A. A. Malik, I. Malik, T. H. Mallhi, M. A. Mansournia, S. Martini, E. Mathews, M. R. Mathur, J. K. Meena, R. G. Menezes, R. Mirfakhraie, S. K. Mirinezhad, M. Mirza-Aghazadeh-Attari, P. Mithra, A. Mohamadkhani, S. Mohammadi, M. Mohammadzadeh, S. Mohan, A. H. Mokdad, A. Al Montasir, F. Montazeri, M. Moradi, M. M. Sarabi, F. Moradpour, M. Moradzadeh, P. Moraga, A. Mosapour, M. Motaghinejad, S. Mubarik, J. S. Muhammad, C. J. L. Murray, A. J. Nagarajan, M. Naghavi, S. Nargus, Z. S. Natto, B. P. Nayak, S. A. Nejadghaderi, P. T. Nguyen, R. K. Niazi, N. Noroozi, H. Okati-Aliabad, A. P. Okekunle, S. Ong, A. M. Oommen, J. R. Padubidri, A. Pandey, E. K. Park, S. Park, S. Pati, S. Patil, R. Paudel, U. Paudel, M. Pirestani, I. Podder, G. Pourali, M. Pourjafar, A. Pourshams, Z. Q. Syed, R. A. Radhakrishnan, V. Radhakrishnan, M. Rahman, S. Rahmani, V. Rahmanian, P. S. Ramesh, J. Rana, I. R. Rao, S. J. Rao, S. Rashedi, M. M. Rashidi, N. Rezaei, N. Rezaei, N. Rezaei, S. Rezaei, M. Rezaeian, G. Roshandel, S. N. Chandan, M. M. Saber-Ayad, S. Sabour, L. Sabzmakan, B. Saddik, U. Saeed, S. Z. Safi, F. S. Sharif-Askari, A. Sahebkar, H. Sahoo, S. A. Sajedi, M. R. Sajid, M. A. Salehi, A. S. Farrokhi, M. A. Sarasmita, S. Sargazi, G. S. Sarode, S. C. Sarode, B. Sathian, M. Satpathy, P. Semwal, S. Senthilkumaran, S. G. Sepanlou, M. Shafeghat, S. Shahabi, A. Shahbandi, F. Shahraki-Sanavi, M. A. Shaikh, M. Shannawaz, R. A. Sheikhi, P. Shobeiri, S. A. Shorofi, S. Shrestha, S. Siabani, G. Singh, P. Singh, S. Singh, D. N. Sinha, S. S. Siwal, S. Sreeram, M. Suleman, R. S. Abdulkader, I. Sultan, A. Sultana, M. Tabish, T. Tabuchi, M. Taheri, I. M. Talaat, A. Tehrani-Banihashemi, M. H. Temsah, P. Thangaraju, N. Thomas, N. K. Thomas, A. Tiyuri, R. Tobe-Gai, R. Toghroli, M. R. T. Palone, S. Ullah, B. Unnikrishnan, E. Upadhyay, S. V. Tahbaz, R. Valizadeh, S. B. Varthya, Y. Waheed, S. Wang, D. P. Wickramasinghe, N. D. Wickramasinghe, H. Xiao, N. Yonemoto, M. Z. Younis, C. H. Yu, M. Zahir, N. Zaki, M. Zamanian, Z. J. Zhang, H. Q. Zhao, O. A. Zitoun, M. Zoladl and G. B. D. A. A. Canc | 2024 | Temporal patterns of cancer burden in Asia, 1990-2019: a systematic examination for the Global Burden of Disease 2019 study | Lancet Regional Health - Southeast Asia | 10.1016/j.lansea.2023.100333 |
| **64** | R. Sharma and B. Rakshit | 2023 | Global burden of cancers attributable to tobacco smoking, 1990-2019: an ecological study | Epma Journal | 10.1007/s13167-022-00308-y |
| **65** | R. J. Shephard | 2017 | Cancers of the Esophagus and Stomach: Potential Mechanisms Behind the Beneficial Influence of Physical Activity | Clinical Journal of Sport Medicine | 10.1097/jsm.0000000000000353 |
| **66** | M. W. Short, K. G. Burgers and V. T. Fry | 2017 | Esophageal Cancer | American Family Physician |  |
| **67** | G. Shrestha, R. K. Thakur, R. Singh, R. Mulmi, A. Shrestha and P. M. S. Pradhan | 2021 | Cancer burden in Nepal, 1990-2017: An analysis of the Global Burden of Disease study | Plos One | 10.1371/journal.pone.0255499 |
| **68** | K. Siddiqi, S. Husain, A. Vidyasagaran, A. Readshaw, M. P. Mishu and A. Sheikh | 2020 | Global burden of disease due to smokeless tobacco consumption in adults: an updated analysis of data from 127 countries | Bmc Medicine | 10.1186/s12916-020-01677-9 |
| **69** | M. Song, H. Jayasekara, C. Pelucchi, C. S. Rabkin, K. C. Johnson, J. F. Hu, D. Palli, M. Ferraroni, L. M. Liao, R. Bonzi, D. Zaridze, D. Maximovitch, N. Aragonés, V. Martin, G. Castaño-Vinyals, M. Guevara, S. Tsugane, G. S. Hamada, A. Hidaka, E. Negri, M. H. Ward, R. Sinha, A. Lagiou, P. Lagiou, P. Boffetta, M. P. Curado, N. Lunet, J. Vioque, Z. F. Zhang, C. La Vecchia and M. C. Camargo | 2024 | Reproductive factors, hormonal interventions, and gastric cancer risk in the Stomach cancer Pooling (StoP) Project | Cancer Causes & Control | 10.1007/s10552-023-01829-1 |
| **70** | Y. X. Song, X. J. Liu, W. W. Cheng, H. Q. Li and D. C. Zhang | 2022 | The global, regional and national burden of stomach cancer and its attributable risk factors from 1990 to 2019 | Scientific Reports | 10.1038/s41598-022-15839-7 |
| **71** | V. Stanghellini, F. K. L. Chan, W. L. Hasler, J. R. Malagelada, H. Suzuki, J. Tack and N. J. Talley | 2016 | Gastroduodenal Disorders | Gastroenterology | 10.1053/j.gastro.2016.02.011 |
| **72** | J. A. Stephens, J. L. Fisher, J. L. Krok-Schoen, R. D. Baltic, H. L. Sobotka and E. D. Paskett | 2018 | Esophageal Adenocarcinoma: Opportunities for Targeted Prevention in Ohio | Clinical Medicine Insights-Gastroenterology | 10.1177/1179552218791170 |
| **73** | F. R. Takeda, R. A. A. Sallum, F. A. Fernandes and I. Cecconello | 2022 | McKeown-cervical anastomosis in minimally invasive esophagectomy | Annals of Esophagus | 10.21037/aoe-21-11 |
| **74** | S. H. Tella, K. Mara, S. Chakrabarti, Z. H. Jin and A. Mahipal | 2023 | A glimpse into the future of esophageal carcinoma in the United States: predicting the future incidence until 2040 based on the current epidemiological data | Journal of Gastrointestinal Oncology | 10.21037/jgo-22-729 |
| **75** | A. P. Thrift, M. B. Cook, T. L. Vaughan, L. A. Anderson, L. J. Murray, D. C. Whiteman, N. J. Shaheen and D. A. Corley | 2014 | Alcohol and the Risk of Barrett's Esophagus: A Pooled Analysis from the International BEACON Consortium | American Journal of Gastroenterology | 10.1038/ajg.2014.206 |
| **76** | T. Udo | 2019 | Chronic Medical Conditions in US Adults With Incarceration History | Health Psychology | 10.1037/hea0000720 |
| **77** | Z. S. Varniab, S. S. Moghaddam, A. P. Langroudi, S. Azadnajafabad, S. S. Mortazavi, A. Sheidaei, K. Gohari, Y. Farzi, Z. S. Moghaddam, H. Sohrabi and M. Shati | 2024 | The levels and trends of cancer incidence in the elderly population at national and sub-national scales in Iran from 1990 to 2016 | Cancer Reports | 10.1002/cnr2.1937 |
| **78** | F. Vitelli-Storelli, M. Rubín-García, C. Pelucchi, Y. Benavente, R. Bonzi, M. Rota, D. Palli, M. Ferraroni, N. Lunet, S. Morais, W. M. Ye, A. Plymoth, R. Malekzadeh, S. Tsugane, A. Hidaka, N. Aragonés, G. Castaño-Vinyals, D. G. Zaridze, D. Maximovich, J. Vioque, M. García-de-la-Hera, Z. F. Zhang, G. S. Hamada, M. Pakseresht, F. Pourfarzi, L. N. Mu, S. Boccia, R. Pastorino, G. P. Yu, A. Lagiou, P. Lagiou, E. Negri, C. La Vecchia and V. Martín | 2021 | Family History and Gastric Cancer Risk: A Pooled Investigation in the Stomach Cancer Pooling (STOP) Project Consortium | Cancers | 10.3390/cancers13153844 |
| **79** | L. Walsh, B. Grosche, M. Schnelzer, A. Tschense, M. Sogl and M. Kreuzer | 2015 | A review of the results from the German Wismut uranium miners cohort | Radiation Protection Dosimetry | 10.1093/rpd/ncu281 |
| **80** | E. C. M. Wennerström, R. A. Risques, D. Prunkard, C. Giffen, D. A. Corley, L. J. Murray, D. C. Whiteman, A. H. Wu, L. Bernstein, W. M. Ye, W. H. Chow, T. L. Vaughan and L. M. Liao | 2016 | Leukocyte telomere length in relation to the risk of Barrett's esophagus and esophageal adenocarcinoma | Cancer Medicine | 10.1002/cam4.810 |
| **81** | G. B. Wu, Q. X. Wu, J. Xu, G. H. Gao, T. T. Chen and G. W. Chen | 2024 | Mortality burden and future projections of major risk factors for esophageal cancer in China from 1990 to 2019 | General Thoracic and Cardiovascular Surgery | 10.1007/s11748-023-01987-8 |
| **82** | H. D. Wu, J. J. Zhang and B. J. Zhou | 2021 | Toothbrushing frequency and gastric and upper aerodigestive tract cancer risk: A meta-analysis | European Journal of Clinical Investigation | 10.1111/eci.13478 |
| **83** | S. L. Wu, W. F. Jiang, J. F. Li, Z. Q. Wu, C. Y. Xu and N. Xie | 2023 | Global burden of esophageal cancer attributable to smoking: a systematic analysis for the Global Burden of Disease Study 2019 | Frontiers in Oncology | 10.3389/fonc.2023.1223164 |
| **84** | Z. H. Wu, K. Zhang, W. J. Wang, M. K. Fan and R. Lin | 2024 | The differences in gastric cancer epidemiological data between SEER and GBD: a joinpoint and age-period-cohort analysis | Journal of Big Data | 10.1186/s40537-024-00907-8 |
| **85** | Q. H. Xu, M. G. Zhou, P. Yin and D. H. Jin | 2023 | Projections of cancer mortality by 2025 in central China: A modeling study of global burden of disease 2019 | Heliyon | 10.1016/j.heliyon.2023.e13432 |
| **86** | Z. S. Xu, F. Qi, Y. N. Wang, X. R. Jia, P. Lin, M. Y. Geng, R. Wang and S. P. Li | 2018 | Cancer mortality attributable to cigarette smoking in 2005, 2010 and 2015 in Qingdao, China | Plos One | 10.1371/journal.pone.0204221 |
| **87** | S. Yang, S. Lin, N. Li, Y. J. Deng, M. Wang, D. Xiang, G. Xiang, S. Q. Wang, X. H. Ye, Y. Zheng, J. Yao, Z. Zhai, Y. Wu, J. J. Hu, H. F. Kang and Z. J. Dai | 2020 | Burden, trends, and risk factors of esophageal cancer in China from 1990 to 2017: an up-to-date overview and comparison with those in Japan and South Korea | Journal of Hematology & Oncology | 10.1186/s13045-020-00981-4 |
| **88** | S. W. Yi, J. S. Hong, J. J. Yi and H. Ohrr | 2016 | Impact of alcohol consumption and body mass index on mortality from nonneoplastic liver diseases, upper aerodigestive tract cancers, and alcohol use disorders in Korean older middle-aged men: Prospective cohort study | Medicine | 10.1097/md.0000000000004876 |
| **89** | S. Z. Zhang, L. Zhang and L. Xie | 2022 | Cancer Burden in China during 1990-2019: Analysis of the Global Burden of Disease | Biomed Research International | 10.1155/2022/3918045 |
| **90** | Z. Q. Zhang, J. Wang, N. Song, L. B. Shi and J. J. Du | 2023 | The global, regional, and national burden of stomach cancer among adolescents and young adults in 204 countries and territories, 1990-2019: A population-based study | Frontiers in Public Health | 10.3389/fpubh.2023.1079248 |
| **91** | J. K. Zhao, M. Wu, C. H. Kim, Z. Y. Jin, J. Y. Zhou, R. Q. Han, J. Yang, X. F. Zhang, X. S. Wang, A. M. Liu, X. P. Gu, M. Su, X. Hu, Z. Sun, G. Li, L. M. Li, L. Mu and Z. F. Zhang | 2017 | Jiangsu Four Cancers Study: a large case-control study of lung, liver, stomach, and esophageal cancers in Jiangsu Province, China | European Journal of Cancer Prevention | 10.1097/cej.0000000000000262 |
| **92** | Q. Zou, H. Y. Tan, J. C. Li, Y. D. Li and K. Yang | 2023 | Metabolic-associated fatty liver disease and risk of esophagogastric cancer: a systematic review and meta-analysis | Japanese Journal of Clinical Oncology | 10.1093/jjco/hyad038 |
