# Supplementary material for: Gastroesophageal disease risk and inhalational exposure a systematic review and meta-analysis
Source: Sci Rep. 2025 Jul 2;15:22581. doi: 10.1038/s41598-025-06620-7 (PMC12218983; doi:10.1038/s41598-025-06620-7)
Supplement: Supplementary file 4 — Supplementary Material 4. [file 41598_2025_6620_MOESM4_ESM.docx]

| **Supplemental Table 4. Translational Studies Excluded (N = 211)** | | | | | |
| --- | --- | --- | --- | --- | --- |
|  | **Author(s)** | **Year** | **Title** | **Journal** | **DOI** |
|  | **PubMed (N = 85)** | | | | |
| **1** | Bai, F. and Xiao, K. | 2020 | Prediction of gastric cancer risk: association between ZBTB20 genetic variance and gastric cancer risk in Chinese Han population | Biosci Rep | 10.1042/bsr20202102 |
| **2** | Banday, A. R., Papenberg, B. W. and Prokunina-Olsson, L. | 2020 | When the Smoke Clears m(6)A from a Y Chromosome-Linked lncRNA, Men Get an Increased Risk of Cancer | Cancer Res | 10.1158/0008-5472.Can-20-0961 |
| **3** | Becskeházi, E., Korsós, M. M., Gál, E., Tiszlavicz, L., Hoyk, Z., Deli, M. A., Köhler, Z. M., Keller-Pintér, A., Horváth, A., Csekő, K., Helyes, Z., Hegyi, P. and Venglovecz, V. | 2021 | Inhibition of NHE-1 Increases Smoke-Induced Proliferative Activity of Barrett's Esophageal Cell Line | Int J Mol Sci | 10.3390/ijms221910581 |
| **4** | Benli Yavuz, B., Koç, M., Kozacıoğlu, S., Kanyılmaz, G. and Aktan, M. | 2019 | Prognostic importance of PTEN, EGFR, HER-2, and IGF-1R in gastric cancer patients treated with postoperative chemoradiation | Turk J Med Sci | 10.3906/sag-1802-34 |
| **5** | Buas, M. F., Onstad, L., Levine, D. M., Risch, H. A., Chow, W. H., Liu, G., Fitzgerald, R. C., Bernstein, L., Ye, W., Bird, N. C., Romero, Y., Casson, A. G., Corley, D. A., Shaheen, N. J., Wu, A. H., Gammon, M. D., Reid, B. J., Hardie, L. J., Peters, U., Whiteman, D. C. and Vaughan, T. L. | 2015 | MiRNA-Related SNPs and Risk of Esophageal Adenocarcinoma and Barrett's Esophagus: Post Genome-Wide Association Analysis in the BEACON Consortium | PLoS One | 10.1371/journal.pone.0128617 |
| **6** | Cai, M., Dai, S., Chen, W., Xia, C., Lu, L., Dai, S., Qi, J., Wang, M., Wang, M., Zhou, L., Lei, F., Zuo, T., Zeng, H. and Zhao, X. | 2017 | Environmental factors, seven GWAS-identified susceptibility loci, and risk of gastric cancer and its precursors in a Chinese population | Cancer Med | 10.1002/cam4.1038 |
| **7** | Camargo, M. C., Song, M., Ito, H., Oze, I., Koyanagi, Y. N., Kasugai, Y., Rabkin, C. S. and Matsuo, K. | 2021 | Associations of circulating mediators of inflammation, cell regulation and immune response with esophageal squamous cell carcinoma | J Cancer Res Clin Oncol | 10.1007/s00432-021-03687-3 |
| **8** | Chen, W. C., Bye, H., Matejcic, M., Amar, A., Govender, D., Khew, Y. W., Beynon, V., Kerr, R., Singh, E., Prescott, N. J., Lewis, C. M., Babb de Villiers, C., Parker, M. I. and Mathew, C. G. | 2019 | Association of genetic variants in CHEK2 with oesophageal squamous cell carcinoma in the South African Black population | Carcinogenesis | 10.1093/carcin/bgz026 |
| **9** | Cook, M. B., Barnett, M. J., Bock, C. H., Cross, A. J., Goodman, P. J., Goodman, G. E., Haiman, C. A., Khaw, K. T., McCullough, M. L., Newton, C. C., Boutron-Ruault, M. C., Lund, E., Rutegård, M., Thornquist, M. D., Spriggs, M., Giffen, C., Freedman, N. D., Kemp, T., Kroenke, C. H., Le Marchand, L., Park, J. Y., Simon, M., Wilkens, L. R., Pinto, L., Hildesheim, A. and Campbell, P. T. | 2019 | Prediagnostic circulating markers of inflammation and risk of oesophageal adenocarcinoma: a study within the National Cancer Institute Cohort Consortium | Gut | 10.1136/gutjnl-2018-316678 |
| **10** | Dai, J. Y., Tapsoba Jde, D., Buas, M. F., Risch, H. A. and Vaughan, T. L. | 2016 | Constrained Score Statistics Identify Genetic Variants Interacting with Multiple Risk Factors in Barrett's Esophagus | Am J Hum Genet | 10.1016/j.ajhg.2016.06.018 |
| **11** | Datta, K. K., Patil, S., Patel, K., Babu, N., Raja, R., Nanjappa, V., Mangalaparthi, K. K., Dhaka, B., Rajagopalan, P., Deolankar, S. C., Kannan, R., Kumar, P., Prasad, T. S. K., Mathur, P. P., Kumari, A., Manoharan, M., Coral, K., Murugan, S., Sidransky, D., Gupta, R., Gupta, R., Khanna-Gupta, A., Chatterjee, A. and Gowda, H. | 2019 | Chronic Exposure to Chewing Tobacco Induces Metabolic Reprogramming and Cancer Stem Cell-Like Properties in Esophageal Epithelial Cells | Cells | 10.3390/cells8090949 |
| **12** | El Khadir, M., Boukhris Alaoui, S., Benajah, D. A., Ibrahimi, S. A., Chbani, L., El Abkari, M. and Bennani, B. | 2020 | VacA genotypes and cagA-EPIYA-C motifs of Helicobacter pylori and gastric histopathological lesions | Int J Cancer | 10.1002/ijc.33158 |
| **13** | Fu, C. K., Mong, M. C., Tzeng, H. E., Yang, M. D., Chen, J. C., Hsia, T. C., Hsia, N. Y., Tsai, C. W., Chang, W. S., Chen, C. P. and Bau, D. T. | 2024 | The Significant Contribution of Interleukin-16 Genotypes, Smoking, Alcohol Drinking, and Helicobacter Pylori Infection to Gastric Cancer | In Vivo | 10.21873/invivo.13414 |
| **14** | Gallegos-Arreola, M. P., Zúñiga-González, G. M., Sánchez-López, J. Y., Cruz, A. Y. N., Peralta-Leal, V., Figuera, L. E., Puebla-Pérez, A. M., Ronquillo-Carreón, C. A. and Puebla-Mora, A. G. | 2018 | TYMS 2R3R polymorphism and DPYD [IVS]14+1G>A gene mutation in Mexican colorectal cancer patients | Acta Biochim Pol | 10.18388/abp.2017_2338 |
| **15** | Ghosh, P., Alam, N., Mandal, S., Mustafi, S. M. and Murmu, N. | 2020 | Association of mTOR pathway with risk of gastric cancer in male smoker with potential prognostic significance | Mol Biol Rep | 10.1007/s11033-020-05808-6 |
| **16** | Gong, J., Chu, Y., Xu, M., Huo, J. and Lv, L. | 2016 | Esophageal squamous cell carcinoma cell proliferation induced by exposure to low concentration of cigarette smoke extract is mediated via targeting miR-101-3p/COX-2 pathway | Oncol Rep | 10.3892/or.2015.4379 |
| **17** | Haider, S. H., Kwon, S., Lam, R., Lee, A. K., Caraher, E. J., Crowley, G., Zhang, L., Schwartz, T. M., Zeig-Owens, R., Liu, M., Prezant, D. J. and Nolan, A. | 2018 | Predictive Biomarkers of Gastroesophageal Reflux Disease and Barrett's Esophagus in World Trade Center Exposed Firefighters: a 15 Year Longitudinal Study | Sci Rep | 10.1038/s41598-018-21334-9 |
| **18** | Jeong, S., Jo, M. J., Yun, H. K., Kim, D. Y., Kim, B. R., Kim, J. L., Park, S. H., Na, Y. J., Jeong, Y. A., Kim, B. G., Ashktorab, H., Smoot, D. T., Heo, J. Y., Han, J., Il Lee, S., Do Kim, H., Kim, D. H., Oh, S. C. and Lee, D. H. | 2019 | Cannabidiol promotes apoptosis via regulation of XIAP/Smac in gastric cancer | Cell Death Dis | 10.1038/s41419-019-2001-7 |
| **19** | Jetté, M. E., Dill-McFarland, K. A., Hanshew, A. S., Suen, G. and Thibeault, S. L. | 2016 | The human laryngeal microbiome: effects of cigarette smoke and reflux | Sci Rep | 10.1038/srep35882 |
| **20** | Jetté, M. E., Seroogy, C. M. and Thibeault, S. L. | 2017 | Laryngeal T regulatory cells in the setting of smoking and reflux | Laryngoscope | 10.1002/lary.26223 |
| **21** | Jiang, D., Song, Q., Zhang, F., Xu, C., Li, X., Zeng, H., Su, J., Huang, J., Xu, Y., Lu, S. and Hou, Y. | 2023 | Prognostic significance of CCND1 amplification/overexpression in smoking patients with esophageal squamous cell carcinoma | Cancer Genet | 10.1016/j.cancergen.2023.07.004 |
| **22** | Jing, J. J., Sun, L. P., Xu, Q. and Yuan, Y. | 2015 | Effect of ERCC8 tagSNPs and their association with H. pylori infection, smoking, and alcohol consumption on gastric cancer and atrophic gastritis risk | Tumour Biol | 10.1007/s13277-015-3703-9 |
| **23** | Kawano, H., Saeki, H., Kitao, H., Tsuda, Y., Otsu, H., Ando, K., Ito, S., Egashira, A., Oki, E., Morita, M., Oda, Y. and Maehara, Y. | 2014 | Chromosomal instability associated with global DNA hypomethylation is associated with the initiation and progression of esophageal squamous cell carcinoma | Ann Surg Oncol | 10.1245/s10434-014-3818-z |
| **24** | Kaz, A. M., Wong, C. J., Varadan, V., Willis, J. E., Chak, A. and Grady, W. M. | 2016 | Global DNA methylation patterns in Barrett's esophagus, dysplastic Barrett's, and esophageal adenocarcinoma are associated with BMI, gender, and tobacco use | Clin Epigenetics | 10.1186/s13148-016-0273-7 |
| **25** | Keene, J. D., Jacobson, S., Kechris, K., Kinney, G. L., Foreman, M. G., Doerschuk, C. M., Make, B. J., Curtis, J. L., Rennard, S. I., Barr, R. G., Bleecker, E. R., Kanner, R. E., Kleerup, E. C., Hansel, N. N., Woodruff, P. G., Han, M. K., Paine, R., 3rd, Martinez, F. J., Bowler, R. P. and O'Neal, W. K. | 2017 | Biomarkers Predictive of Exacerbations in the SPIROMICS and COPDGene Cohorts | Am J Respir Crit Care Med | 10.1164/rccm.201607-1330OC |
| **26** | Kim, H. S., Lee, S. E., Bae, Y. S., Kim, D. J., Lee, C. G., Hur, J., Chung, H., Park, J. C., Jung, D. H., Shin, S. K., Lee, S. K., Lee, Y. C., Kim, H. R., Moon, Y. W., Kim, J. H., Shim, Y. M., Jewell, S. S., Kim, H., Choi, Y. L. and Cho, B. C. | 2015 | Fibroblast growth factor receptor 1 gene amplification is associated with poor survival in patients with resected esophageal squamous cell carcinoma | Oncotarget | 10.18632/oncotarget.2944 |
| **27** | Kumar, S., Kumari, N., Mittal, R. D. and Ghoshal, U. C. | 2016 | Pepsinogen-II 100 bp ins/del gene polymorphism and its elevated circulating levels are associated with gastric cancer, particularly with Helicobacter pylori infection and intestinal metaplasia | Gastric Cancer | 10.1007/s10120-015-0550-8 |
| **28** | Kuo, W. H., Huang, C. Y., Fu, C. K., Hsieh, Y. H., Liao, C. H., Hsu, C. M., Huang, Y. K., Tsai, C. W., Chang, W. S. and Bau, D. T. | 2014 | Effects of interleukin-10 polymorphisms and smoking on the risk of gastric cancer in Taiwan | In Vivo |  |
| **29** | Lachmann, G., von Haefen, C., Kurth, J., Yuerek, F., Wernecke, K. D. and Spies, C. | 2017 | Smoking, Gender, and Overweight Are Important Influencing Factors on Monocytic HLA-DR before and after Major Cancer Surgery | Biomed Res Int | 10.1155/2017/5216562 |
| **30** | Li, G., Wulan, H., Song, Z., Paik, P. A., Tsao, M. L., Goodman, G. M., MacEachern, P. T., Downey, R. S., Jankowska, A. J., Rabinowitz, Y. M., Learch, T. B., Song, D. Z., Yuan, J. J., Zheng, S. and Zheng, Z. | 2015 | Regulatory B Cell Function Is Suppressed by Smoking and Obesity in H. pylori-Infected Subjects and Is Correlated with Elevated Risk of Gastric Cancer | PLoS One | 10.1371/journal.pone.0134591 |
| **31** | Li, W. and Wang, W. | 2024 | Causal effects of exposure to ambient air pollution on cancer risk: Insights from genetic evidence | Sci Total Environ | 10.1016/j.scitotenv.2023.168843 |
| **32** | Liang, Z. F., Zhang, Y., Guo, W., Chen, B., Fang, S. and Qian, H. | 2022 | Gastric cancer stem cell-derived exosomes promoted tobacco smoke-triggered development of gastric cancer by inducing the expression of circ670 | Med Oncol | 10.1007/s12032-022-01906-6 |
| **33** | Lopes, A. B., Metzdorf, M., Metzdorf, L., Sousa, M. P. R., Kavalco, C., Etemadi, A., Pritchett, N. R., Murphy, G., Calafat, A. M., Abnet, C. C., Dawsey, S. M. and Fagundes, R. B. | 2018 | Urinary Concentrations of Polycyclic Aromatic Hydrocarbon Metabolites in Maté Drinkers in Rio Grande do Sul, Brazil | Cancer Epidemiol Biomarkers Prev | 10.1158/1055-9965.Epi-17-0773 |
| **34** | Lu, L., Chen, J., Li, M., Tang, L., Wu, R., Jin, L. and Liang, Z. | 2018 | β‑carotene reverses tobacco smoke‑induced gastric EMT via Notch pathway in vivo | Oncol Rep | 10.3892/or.2018.6246 |
| **35** | Lv, Z., Sun, L., Xu, Q., Gong, Y., Jing, J., Dong, N., Xing, C. and Yuan, Y. | 2018 | SNP interactions of PGC with its neighbor lncRNAs enhance the susceptibility to gastric cancer/atrophic gastritis and influence the expression of involved molecules | Cancer Med | 10.1002/cam4.1743 |
| **36** | Mantziari, S., Allemann, P., Winiker, M., Demartines, N. and Schäfer, M. | 2018 | Locoregional Tumor Extension and Preoperative Smoking are Significant Risk Factors for Early Recurrence After Esophagectomy for Cancer | World J Surg | 10.1007/s00268-017-4422-8 |
| **37** | Matejcic, M., Vogelsang, M., Wang, Y. and Iqbal Parker, M. | 2015 | NAT1 and NAT2 genetic polymorphisms and environmental exposure as risk factors for oesophageal squamous cell carcinoma: a case-control study | BMC Cancer | 10.1186/s12885-015-1105-4 |
| **38** | McCain, R. S., McManus, D. T., McQuaid, S., James, J. A., Salto-Tellez, M., Reid, N. B., Craig, S., Chisambo, C., Bingham, V., McCarron, E., Parkes, E., Turkington, R. C. and Coleman, H. G. | 2020 | Alcohol intake, tobacco smoking, and esophageal adenocarcinoma survival: a molecular pathology epidemiology cohort study | Cancer Causes Control | 10.1007/s10552-019-01247-2 |
| **39** | Miranti, E. H., Freedman, N. D., Weinstein, S. J., Abnet, C. C., Selhub, J., Murphy, G., Diaw, L., Männistö, S., Taylor, P. R., Albanes, D. and Stolzenberg-Solomon, R. Z. | 2016 | Prospective study of serum cysteine and cysteinylglycine and cancer of the head and neck, esophagus, and stomach in a cohort of male smokers | Am J Clin Nutr | 10.3945/ajcn.115.125799 |
| **40** | Montiel-Jarquín Á, J., Lara-Cisneros, L. G. V., López-Colombo, A., Solís-Mendoza, H. A., Palmer-Márquez, M. L. and Romero-Figueroa, M. S. | 2019 | Expression of metalloproteinase-9 in patients with mild and severe forms of gastroesophageal reflux disease | Cir Cir | 10.24875/ciru.18000691 |
| **41** | Muhsen, K., Sinnreich, R., Merom, D., Beer-Davidson, G., Nassar, H., Cohen, D. and Kark, J. D. | 2019 | Prevalence and determinants of serological evidence of atrophic gastritis among Arab and Jewish residents of Jerusalem: a cross-sectional study | BMJ Open | 10.1136/bmjopen-2018-024689 |
| **42** | Mukherjee, D., Devi, K. R., Deka, M., Malakar, M., Kaur, T., Barua, D., Mahanta, J. and Narain, K. | 2016 | Association of toll-like receptor 2 ∆22 and risk for gastric cancer considering main effects and interactions with smoking: a matched case-control study from Mizoram, India | Tumour Biol | 10.1007/s13277-016-4982-5 |
| **43** | Negovan, A., Iancu, M., Moldovan, V., Mocan, S. and Banescu, C. | 2017 | The Interaction between GSTT1, GSTM1, and GSTP1 Ile105Val Gene Polymorphisms and Environmental Risk Factors in Premalignant Gastric Lesions Risk | Biomed Res Int | 10.1155/2017/7365080 |
| **44** | Negovan, A., Iancu, M., Tripon, F., Crauciuc, A., Mocan, S. and Bănescu, C. | 2018 | The CAT-262 C>T, MnSOD Ala16Val, GPX1 Pro198Leu Polymorphisms Related to Oxidative Stress and the Presence of Gastric Lesions | J Gastrointestin Liver Dis | 10.15403/jgld.2014.1121.274.cat |
| **45** | Nguyen, T., Tang, Z., Younes, M., Alsarraj, A., Ramsey, D., Fitzgerald, S., Kramer, J. R. and El-Serag, H. B. | 2015 | Esophageal COX-2 expression is increased in Barrett's esophagus, obesity, and smoking | Dig Dis Sci | 10.1007/s10620-014-3333-x |
| **46** | Ong, J. S., Gharahkhani, P., Vaughan, T. L., Whiteman, D., Kendall, B. J. and MacGregor, S. | 2022 | Assessing the genetic relationship between gastro-esophageal reflux disease and risk of COVID-19 infection | Hum Mol Genet | 10.1093/hmg/ddab253 |
| **47** | Pan, W., Du, J., Shi, M., Jin, G. and Yang, M. | 2017 | Short leukocyte telomere length, alone and in combination with smoking, contributes to increased risk of gastric cancer or esophageal squamous cell carcinoma | Carcinogenesis | 10.1093/carcin/bgw111 |
| **48** | Pan, W., Yang, J., Wei, J., Chen, H., Ge, Y., Zhang, J., Wang, Z., Zhou, C., Yuan, Q., Zhou, L. and Yang, M. | 2015 | Functional BCL-2 regulatory genetic variants contribute to susceptibility of esophageal squamous cell carcinoma | Sci Rep | 10.1038/srep11833 |
| **49** | Pandey, A., Tripathi, S. C., Shukla, S., Mahata, S., Vishnoi, K., Misra, S. P., Misra, V., Mitra, S., Dwivedi, M. and Bharti, A. C. | 2018 | Differentially localized survivin and STAT3 as markers of gastric cancer progression: Association with Helicobacter pylori | Cancer Rep (Hoboken) | 10.1002/cnr2.1004 |
| **50** | Patterson, K. A., Roberts-Thomson, P. J., Lester, S., Tan, J. A., Hakendorf, P., Rischmueller, M., Zochling, J., Sahhar, J., Nash, P., Roddy, J., Hill, C., Nikpour, M., Stevens, W., Proudman, S. M. and Walker, J. G. | 2015 | Interpretation of an Extended Autoantibody Profile in a Well-Characterized Australian Systemic Sclerosis (Scleroderma) Cohort Using Principal Components Analysis | Arthritis Rheumatol | 10.1002/art.39316 |
| **51** | Rafiq, R., Bhat, G. A., Lone, M. M., Masood, A. and Dar, N. A. | 2016 | Potential risk of esophageal squamous cell carcinoma due to nucleotide excision repair XPA and XPC gene variants and their interaction among themselves and with environmental factors | Tumour Biol | 10.1007/s13277-016-4895-3 |
| **52** | Saikia, S., Rehman, A. U., Barooah, P., Sarmah, P., Bhattacharyya, M., Deka, M., Deka, M., Goswami, B., Husain, S. A. and Medhi, S. | 2017 | Alteration in the expression of MGMT and RUNX3 due to non-CpG promoter methylation and their correlation with different risk factors in esophageal cancer patients | Tumour Biol | 10.1177/1010428317701630 |
| **53** | Shah, I. A., Bhat, G. A., Mehta, P., Lone, M. M. and Dar, N. A. | 2016 | Genotypes of CYP1A1, SULT1A1 and SULT1A2 and risk of squamous cell carcinoma of esophagus: outcome of a case-control study from Kashmir, India | Dis Esophagus | 10.1111/dote.12427 |
| **54** | Shao, Y., Guo, X., Zhao, L., Shen, Y., Niu, C., Wei, W. and Liu, F. | 2020 | A Functional Variant of the miR-15 Family Is Associated with a Decreased Risk of Esophageal Squamous Cell Carcinoma | DNA Cell Biol | 10.1089/dna.2020.5606 |
| **55** | Song, Q., Zhang, Z., Liu, Y., Han, S. and Zhang, X. | 2015 | The tag SNP rs10746463 in decay-accelerating factor is associated with the susceptibility to gastric cancer | Mol Immunol | 10.1016/j.molimm.2014.10.006 |
| **56** | Sun, L., Tu, H., Liu, J., Gong, Y., Xu, Q., Jing, J., Dong, N. and Yuan, Y. | 2014 | A comprehensive evaluation of fasting serum gastrin-17 as a predictor of diseased stomach in Chinese population | Scand J Gastroenterol | 10.3109/00365521.2014.950693 |
| **57** | Tasneem, S., Sarwar, M. T., Bashir, M. R., Hussain, H., Ahmed, J. and Pervez, S. | 2018 | Expression analysis of cyclooxygenase-2 in patients suffering from esophageal squamous cell carcinoma | PLoS One | 10.1371/journal.pone.0205508 |
| **58** | Torres-Román, A. L., Rodríguez-Flores, K. L., Hernández-Mora, V. M., Ruiz-García, E., Prospero-García, O., Guijosa, A., Molina, A., Morales-Mulia, M., Aschner, M., Santamaría, A. and Ortega-Gómez, A. | 2023 | Examining the Role of Histaminergic, Orexinergic, and Cannabinergic Systems in Redox Regulation in Gastric Adenocarcinoma | Mini Rev Med Chem | 10.2174/1389557523666230221104504 |
| **59** | Vogelsang, M., Paccez, J. D., Schäfer, G., Dzobo, K., Zerbini, L. F. and Parker, M. I. | 2014 | Aberrant methylation of the MSH3 promoter and distal enhancer in esophageal cancer patients exposed to first-hand tobacco smoke | J Cancer Res Clin Oncol | 10.1007/s00432-014-1736-x |
| **60** | Wang, G., Ye, M., Zheng, S., Wu, K., Geng, H. and Liu, C. | 2020 | Cigarette Smoke Extract induces H19 in Esophageal Squamous Cell Carcinoma in Smoking Patients: Based on A Chronic Exposed Cell Model | Toxicol Lett | 10.1016/j.toxlet.2020.07.030 |
| **61** | Wang, J., Qiu, M., Xu, Y., Li, M., Dong, G., Mao, Q., Yin, R. and Xu, L. | 2015 | Long noncoding RNA CCAT2 correlates with smoking in esophageal squamous cell carcinoma | Tumour Biol | 10.1007/s13277-015-3220-x |
| **62** | Wang, J., Shen, H., Fu, G., Zhao, D. and Wang, W. | 2017 | Nuclear overexpression of the overexpressed in lung cancer 1 predicts worse prognosis in gastric adenocarcinoma | Oncotarget | 10.18632/oncotarget.14217 |
| **63** | Wang, L., Du, L., Xiong, X., Lin, Y., Zhu, J., Yao, Z., Wang, S., Guo, Y., Chen, Y., Geary, K., Pan, Y., Zhou, F., Gao, S., Zhang, D., Yeung, S. J. and Zhang, H. | 2021 | Repurposing dextromethorphan and metformin for treating nicotine-induced cancer by directly targeting CHRNA7 to inhibit JAK2/STAT3/SOX2 signaling | Oncogene | 10.1038/s41388-021-01682-z |
| **64** | Wang, L., Xiao, S., Zheng, Y. and Gao, Z. | 2022 | Interaction Between Vascular Endothelial Growth Factor Gene Polymorphism and Smoking on Gastric Cancer Risk in Chinese Han Population | Pathol Oncol Res | 10.3389/pore.2022.1610495 |
| **65** | Wang, N., Yang, J., Lu, J., Qiao, Q., Bao, G., Wu, T. and He, X. | 2014 | IL-17 gene polymorphism is associated with susceptibility to gastric cancer | Tumour Biol | 10.1007/s13277-014-2255-8 |
| **66** | Wen, J., Pang, Y., Zhou, T., Qi, X., Zhao, M., Xuan, B., Meng, X., Guo, Y., Liu, Q., Liang, H., Li, Y., Dong, H. and Wang, Y. | 2016 | Essential role of Na+/Ca2+ exchanger 1 in smoking-induced growth and migration of esophageal squamous cell carcinoma | Oncotarget | 10.18632/oncotarget.11695 |
| **67** | Wen, Q., Mao, X., Shi, X., Wang, Y. and Wang, J. | 2023 | Impacts of heavy smoking on non-coding RNA expression for patients with esophageal carcinoma | BMC Med Genomics | 10.1186/s12920-023-01574-z |
| **68** | Wu, S., Zhang, L., Deng, J., Guo, B., Li, F., Wang, Y., Wu, R., Zhang, S., Lu, J. and Zhou, Y. | 2020 | A Novel Micropeptide Encoded by Y-Linked LINC00278 Links Cigarette Smoking and AR Signaling in Male Esophageal Squamous Cell Carcinoma | Cancer Res | 10.1158/0008-5472.Can-19-3440 |
| **69** | Xi, S., Inchauste, S., Guo, H., Shan, J., Xiao, Z., Xu, H., Miettenen, M., Zhang, M. R., Hong, J. A., Raiji, M. T., Altorki, N. K., Casson, A. G., Beer, D. G., Robles, A. I., Bowman, E. D., Harris, C. C., Steinberg, S. M. and Schrump, D. S. | 2015 | Cigarette smoke mediates epigenetic repression of miR-217 during esophageal adenocarcinogenesis | Oncogene | 10.1038/onc.2015.10 |
| **70** | Xu, X., Mao, B., Wu, L., Liu, L., Rui, J. and Chen, G. | 2017 | A118G Polymorphism in μ-Opioid Receptor Gene and Interactions with Smoking and Drinking on Risk of Oesophageal Squamous Cell Carcinoma | J Clin Lab Anal | 10.1002/jcla.22018 |
| **71** | Yao, W., Qin, X., Qi, B., Lu, J., Guo, L., Liu, F., Liu, S. and Zhao, B. | 2014 | Association of p53 expression with prognosis in patients with esophageal squamous cell carcinoma | Int J Clin Exp Pathol |  |
| **72** | Yeh, J. M., Hur, C., Ward, Z., Schrag, D. and Goldie, S. J. | 2016 | Gastric adenocarcinoma screening and prevention in the era of new biomarker and endoscopic technologies: a cost-effectiveness analysis | Gut | 10.1136/gutjnl-2014-308588 |
| **73** | Yin, J., Wang, X., Wei, J., Wang, L., Shi, Y., Zheng, L., Tang, W., Ding, G., Liu, C., Liu, R., Chen, S., Xu, Z. and Gu, H. | 2015 | Interleukin 12B rs3212227 T > G polymorphism was associated with an increased risk of gastric cardiac adenocarcinoma in a Chinese population | Dis Esophagus | 10.1111/dote.12189 |
| **74** | Yokota, T., Serizawa, M., Hosokawa, A., Kusafuka, K., Mori, K., Sugiyama, T., Tsubosa, Y. and Koh, Y. | 2018 | PIK3CA mutation is a favorable prognostic factor in esophageal cancer: molecular profile by next-generation sequencing using surgically resected formalin-fixed, paraffin-embedded tissue | BMC Cancer | 10.1186/s12885-018-4733-7 |
| **75** | Yokoyama, A., Kakiuchi, N., Yoshizato, T., Nannya, Y., Suzuki, H., Takeuchi, Y., Shiozawa, Y., Sato, Y., Aoki, K., Kim, S. K., Fujii, Y., Yoshida, K., Kataoka, K., Nakagawa, M. M., Inoue, Y., Hirano, T., Shiraishi, Y., Chiba, K., Tanaka, H., Sanada, M., Nishikawa, Y., Amanuma, Y., Ohashi, S., Aoyama, I., Horimatsu, T., Miyamoto, S., Tsunoda, S., Sakai, Y., Narahara, M., Brown, J. B., Sato, Y., Sawada, G., Mimori, K., Minamiguchi, S., Haga, H., Seno, H., Miyano, S., Makishima, H., Muto, M. and Ogawa, S. | 2019 | Age-related remodelling of oesophageal epithelia by mutated cancer drivers | Nature | 10.1038/s41586-018-0811-x |
| **76** | Yuan, S., Chen, J., Ruan, X., Sun, Y., Zhang, K., Wang, X., Li, X., Gill, D., Burgess, S., Giovannucci, E. and Larsson, S. C. | 2023 | Smoking, alcohol consumption, and 24 gastrointestinal diseases: Mendelian randomization analysis | Elife | 10.7554/eLife.84051 |
| **77** | Zhang, C., Ding, Z., Lv, G., Li, J., Zhang, J. F. and Zhou, P. | 2015 | CD226 rs727088A>G polymorphism increases the susceptibility to gastric cancer in Chinese populations | Gene | 10.1016/j.gene.2014.12.022 |
| **78** | Zhang, J., Huang, X., Xiao, J., Yang, Y., Zhou, Y., Wang, X., Liu, Q., Yang, J., Wang, M., Qiu, L., Zheng, Y., Zhang, P., Li, J., Wang, Y., Wei, Q., Jin, L., Wang, J. and Wang, M. | 2014 | Pri-miR-124 rs531564 and pri-miR-34b/c rs4938723 polymorphisms are associated with decreased risk of esophageal squamous cell carcinoma in Chinese populations | PLoS One | 10.1371/journal.pone.0100055 |
| **79** | Zhang, J., Ju, H., Gao, J. R., Jiao, X. L. and Lu, Y. | 2017 | Polymorphisms in human telomerase reverse transcriptase (hTERT) gene, gene- gene and gene-smoking interaction with susceptibility to gastric cancer in Chinese Han population | Oncotarget | 10.18632/oncotarget.15664 |
| **80** | Zhang, W. B., Gu, H. Y., Shi, Y. J., Shao, A. Z., Chen, S. C., Yin, J. and Jiang, P. C. | 2015 | RANK rs1805034 T>C Polymorphism Is Associated with Susceptibility to Gastric Cardia Adenocarcinoma in a Chinese Population | Oncol Res Treat | 10.1159/000440855 |
| **81** | Zhang, X., Qin, Y., Pan, Z., Li, M., Liu, X., Chen, X., Qu, G., Zhou, L., Xu, M., Zheng, Q. and Li, D. | 2019 | Cannabidiol Induces Cell Cycle Arrest and Cell Apoptosis in Human Gastric Cancer SGC-7901 Cells | Biomolecules | 10.3390/biom9080302 |
| **82** | Zheng, T., BouSaba, J., Taylor, A., Dilmaghani, S., Busciglio, I., Carlson, P., Torres, M., Ryks, M., Burton, D., Harmsen, W. S. and Camilleri, M. | 2023 | A Randomized, Controlled Trial of Efficacy and Safety of Cannabidiol in Idiopathic and Diabetic Gastroparesis | Clin Gastroenterol Hepatol | 10.1016/j.cgh.2023.07.008 |
| **83** | Zhou, H., Sun, H., Liu, X., Chen, J., Zhang, L., Lin, S., Han, X., Nie, C., Liu, Y., Tian, W. and Zhao, Y. | 2019 | Combined effect between WT1 methylation and Helicobacter pylori infection, smoking, and alcohol consumption on the risk of gastric cancer | Helicobacter | 10.1111/hel.12650 |
| **84** | Zhou, R., Li, Y., Wang, N., Niu, C., Huang, X., Cao, S. and Huo, X. | 2021 | PARP1 rs1136410 C/C genotype associated with an increased risk of esophageal cancer in smokers | Mol Biol Rep | 10.1007/s11033-021-06169-4 |
| **85** | Zhu, M. L., He, J., Wang, M., Sun, M. H., Jin, L., Wang, X., Yang, Y. J., Wang, J. C., Zheng, L., Xiang, J. Q. and Wei, Q. Y. | 2014 | Potentially functional polymorphisms in the ERCC2 gene and risk of esophageal squamous cell carcinoma in Chinese populations | Sci Rep | 10.1038/srep06281 |
|  | **Web of Science (N = 126)** | | | | |
| **1** | A. F. Abbas, A. G. M. Al-Saadi and S. A. Fazaa | 2017 | Investigation of IgG and IgM seroprevalence of <i>Helicobacter pylori</i> infections and their relation toIL-6 and some risk factors among dyspeptic patients in Al-Qasim city of Babylon province | Research Journal of Pharmaceutical Biological and Chemical Sciences |  |
| **2** | V. B. Abhilash, M. K. Behera, S. C. U. Patne, S. K. Shukla and V. K. Dixit | 2023 | Clinicopathological Significance and Prognostic Role of Her2neu Protein Expression in Patients with Carcinoma Stomach: A Prospective Study from Northern India | South Asian Journal of Cancer | 10.1055/s-0042-1759601 |
| **3** | K. K. Adkison, J. J. Gan, L. Elko-Simms, S. Gardner, E. Dumont, L. S. Jones, J. Saunders, T. Marbury, W. Smith, J. Berg, C. Galloway and P. J. Stump | 2015 | Pharmacokinetics of hepatitis C virus NS5A inhibitor JNJ-56914845 (GSK2336805) in subjects with hepatic impairment | Journal of Clinical Pharmacology | 10.1002/jcph.512 |
| **4** | S. Akiyama, H. Saeki, Y. Nakashima, M. Iimori, H. Kitao, E. Oki, Y. Oda, Y. Nakabeppu, Y. Kakeji and Y. Maehara | 2017 | Prognostic impact of MutT homolog-1 expression on esophageal squamous cell carcinoma | Cancer Medicine | 10.1002/cam4.979 |
| **5** | B. M. R. Al-Aajem and N. H. Majeed | 2019 | Molecular Study of CagA gene in <i>Helicobacter pylori</i> Isolated from Gastritis | Pakistan Journal of Medical & Health Sciences |  |
| **6** | A. Alotaibi, V. P. Gadekar, P. S. Gundla, S. Mandarthi, N. Jayendra, A. Tungekar, B. V. Lavanya, A. K. Bhagavath, M. A. W. Cordero, J. Pitkaniemi, S. K. Niazi, R. Upadhya, A. Bepari and P. Hebbar | 2023 | Global comparative transcriptomes uncover novel and population-specific gene expression in esophageal squamous cell carcinoma | Infectious Agents and Cancer | 10.1186/s13027-023-00525-8 |
| **7** | X. P. Bai, E. Ihara, Y. Otsuka, S. Tsuruta, K. Hirano, Y. Tanaka, H. Ogino, M. Hirano, T. Chinen, H. Akiho, K. Nakamura, Y. Oda and Y. Ogawa | 2019 | Involvement of different receptor subtypes in prostaglandin E2-induced contraction and relaxation in the lower esophageal sphincter and esophageal body | European Journal of Pharmacology | 10.1016/j.ejphar.2019.172405 |
| **8** | S. Banerjee | 2020 | Risk Associated with NAD (P) H: Quinone Oxidoreductase 1 (NQO1) C609T Polymorphism for Cigarette Smoke Induced Cardiovascular Disease (CVD): A Study on Male Current Smokers from Eastern India | International Journal of Life Science and Pharma Research | 10.22376/ijpbs/lpr.2020.10.4.L29-35 |
| **9** | E. Becskeházi, M. M. Korsós, E. Gál, L. Tiszlavicz, Z. Hoyk, M. A. Deli, Z. M. Köhler, A. Keller-Pintér, A. Horváth, K. Cseko, Z. Helyes, P. Hegyi and V. Venglovecz | 2021 | Inhibition of NHE-1 Increases Smoke-Induced Proliferative Activity of Barrett's Esophageal Cell Line | International Journal of Molecular Sciences | 10.3390/ijms221910581 |
| **10** | Y. Benakli, A. Khenchouche, S. Rabea, A. A. Mansour, M. M. Salem-Bekhit, E. I. Taha, M. M. Salem, S. Singh, K. O. Ouali, Y. Benguerba and K. Houali | 2023 | Characterizing EBV-associated Gastric Carcinoma (EBVaGC): A deep dive into LMP1 expression patterns | Cellular and Molecular Biology | 10.14715/cmb/2023.69.13.33 |
| **11** | E. Brosens, M. Ploeg, Y. van Bever, A. E. Koopmans, H. Ijsselstijn, R. J. Rottier, R. Wijnen, D. Tibboel and A. de Klein | 2014 | Clinical and etiological heterogeneity in patients with tracheo-esophageal malformations and associated anomalies | European Journal of Medical Genetics | 10.1016/j.ejmg.2014.05.009 |
| **12** | M. F. Buas, H. W. Gu, D. Djukovic, J. J. Zhu, L. Onstad, B. J. Reid, D. Raftery and T. L. Vaughan | 2017 | Candidate serum metabolite biomarkers for differentiating gastroesophageal reflux disease, Barrett's esophagus, and high-grade dysplasia/esophageal adenocarcinoma | Metabolomics | 10.1007/s11306-016-1154-y |
| **13** | M. F. Buas, Q. C. He, L. G. Johnson, L. Onstad, D. M. Levine, A. P. Thrift, P. Gharahkhani, C. Palles, J. Lagergren, R. C. Fitzgerald, W. M. Ye, C. Caldas, N. C. Bird, N. J. Shaheen, L. Bernstein, M. D. Gammon, A. H. Wu, L. J. Hardie, P. D. Pharoah, G. Liu, P. Iyer, D. A. Corley, H. A. Risch, W. H. Chow, H. Prenen, L. Chegwidden, S. Love, S. Attwood, P. Moayyedi, D. MacDonald, R. Harrison, P. Watson, H. Barr, J. deCaestecker, I. Tomlinson, J. Jankowski, D. C. Whiteman, S. MacGregor, T. L. Vaughan and M. M. Madeleine | 2017 | Germline variation in inflammation-related pathways and risk of Barrett's oesophagus and oesophageal adenocarcinoma | Gut | 10.1136/gutjnl-2016-311622 |
| **14** | M. F. Buas, D. M. Levine, K. W. Makar, H. Utsugi, L. Onstad, X. H. Li, P. C. Galipeau, N. J. Shaheen, L. J. Hardie, Y. Romero, L. Bernstein, M. D. Gammon, A. G. Casson, N. C. Bird, H. A. Risch, W. M. Ye, G. Liu, D. A. Corley, P. L. Blount, R. C. Fitzgerald, D. C. Whiteman, A. H. Wu, B. J. Reid and T. L. Vaughan | 2014 | Integrative post-genome-wide association analysis of <i>CDKN2A</i> and <i>TP53</i> SNPs and risk of esophageal adenocarcinoma | Carcinogenesis | 10.1093/carcin/bgu207 |
| **15** | M. F. Buas, L. Onstad, D. M. Levine, H. A. Risch, W. H. Chow, G. Liu, R. C. Fitzgerald, L. Bernstein, W. M. Ye, N. C. Bird, Y. Romero, A. G. Casson, D. A. Corley, N. J. Shaheen, A. H. Wu, M. D. Gammon, B. J. Reid, L. J. Hardie, U. Peters, D. C. Whiteman and T. L. Vaughan | 2015 | MiRNA-Related SNPs and Risk of Esophageal Adenocarcinoma and Barrett's Esophagus: Post Genome-Wide Association Analysis in the BEACON Consortium | Plos One | 10.1371/journal.pone.0128617 |
| **16** | J. Cao, Z. Q. Chen, C. Y. Tian, J. Yu, H. F. Zhang, J. W. Yang and W. J. Yang | 2020 | A Shared Susceptibility Locus in the <i>p53</i> Gene for both Gastric and Esophageal Cancers in a Northwestern Chinese Population | Genetic Testing and Molecular Biomarkers | 10.1089/gtmb.2020.0192 |
| **17** | B. F. Chen, Y. W. Jiao, F. K. Yaolong, T. S. Li, Y. X. Liu, M. Q. Wang, X. L. Gu and X. H. Feng | 2019 | The <i>POLR2E</i> rs3787016 polymorphism is strongly associated with the risk of female breast and cervical cancer | Pathology Research and Practice | 10.1016/j.prp.2019.02.015 |
| **18** | S. Christie, R. O'Reilly, H. Li, G. A. Wittert and A. J. Page | 2020 | Biphasic effects of methanandamide on murine gastric vagal afferent mechanosensitivity | Journal of Physiology-London | 10.1113/jp278696 |
| **19** | C. Cotton, P. Alton, D. M. Hughes and S. S. Zhao | 2023 | Genetic liability to gastro-esophageal reflux disease, obesity, and risk of idiopathic pulmonary fibrosis | Respiratory Investigation | 10.1016/j.resinv.2023.02.005 |
| **20** | J. Y. Dai, J. D. Tapsoba, M. F. Buas, L. E. Onstad, D. M. Levine, H. A. Risch, W. H. Chow, L. Bernstein, W. M. Ye, J. Lagergren, N. C. Bird, D. A. Corley, N. J. Shaheen, A. H. Wu, B. J. Reid, L. J. Hardie, D. C. Whiteman and T. L. Vaughan | 2015 | A Newly Identified Susceptibility Locus near <i>FOXP1</i> Modifies the Association of Gastroesophageal Reflux with Barrett's Esophagus | Cancer Epidemiology Biomarkers & Prevention | 10.1158/1055-9965.Epi-15-0507 |
| **21** | J. Y. Dai, J. D. Tapsoba, M. F. Buas, H. A. Risch, T. L. Vaughan and B. Consortium | 2016 | Constrained Score Statistics Identify Genetic Variants Interacting with Multiple Risk Factors in Barrett's Esophagus | American Journal of Human Genetics | 10.1016/j.ajhg.2016.06.018 |
| **22** | J. Y. Deng, J. H. Zhang, C. Y. Wang, Q. Wei, D. Z. Zhou and K. L. Zhao | 2016 | Methylation and expression of PTPN22 in esophageal squamous cell carcinoma | Oncotarget | 10.18632/oncotarget.11581 |
| **23** | Y. X. Deng, T. Qiu, N. Patel, S. Zhou, T. Xue and H. J. Zhang | 2019 | Clinical Management of Risk of Radiation Pneumonia with Serum Markers During the Radiotherapy for Patients with Thoracic Malignant Tumors | Cancer Management and Research | 10.2147/cmar.S231995 |
| **24** | H. Ding, Y. M. Chen, H. Qiu, C. Liu, Y. F. Wang, M. Q. Kang and W. F. Tang | 2017 | PPARG c.1347C&gt;T polymorphism is associated with cancer susceptibility: from a case-control study to a meta-analysis | Oncotarget | 10.18632/oncotarget.20925 |
| **25** | J. Dong, D. M. Levine, M. F. Buas, R. Zhang, L. Onstad, R. C. Fitzgerald, D. A. Corley, N. J. Shaheen, J. Lagergren, L. J. Hardie, B. J. Reid, P. G. Iyer, H. A. Risch, C. Caldas, I. Caldas, P. D. Pharoah, G. Liu, M. D. Gammon, W. H. Chow, L. Bernstein, N. C. Bird, W. M. Ye, A. H. Wu, L. A. Anderson, S. MacGregor, D. C. Whiteman, T. L. Vaughan, A. P. Thrift and S. O. C. Stomach Oesophageal Canc Study | 2018 | Interactions Between Genetic Variants and Environmental Factors Affect Risk of Esophageal Adenocarcinoma and Barrett's Esophagus | Clinical Gastroenterology and Hepatology | 10.1016/j.cgh.2018.03.007 |
| **26** | F. J. Duan, C. H. Song, J. C. Shi, P. Wang, H. Ye, L. P. Dai, J. Y. Zhang and K. J. Wang | 2021 | Identification and epidemiological evaluation of gastric cancer risk factors: based on a field synopsis and meta-analysis in Chinese population | Aging-Us |  |
| **27** | A. Etemadi, H. Poustchi, A. M. Calafat, B. C. Blount, V. R. De Jesús, L. Q. Wang, A. Pourshams, R. Shakeri, M. Inoue-Choi, M. S. Shiels, G. Roshandel, G. Murphy, C. S. Sosnoff, D. Bhandari, J. Feng, B. Y. Xia, Y. S. Wang, L. Meng, F. Kamangar, P. Brennan, P. Boffetta, S. M. Dawsey, C. C. Abnet, R. Malekzadeh and N. D. Freedman | 2020 | Opiate and Tobacco Use and Exposure to Carcinogens and Toxicants in the Golestan Cohort Study | Cancer Epidemiology Biomarkers & Prevention | 10.1158/1055-9965.Epi-19-1212 |
| **28** | A. Etemadi, H. Poustchi, C. M. Chang, B. C. Blount, A. M. Calafat, L. Q. Wang, V. R. De Jesus, A. Pourshams, R. Shakeri, M. S. Shiels, M. Inoue-Choi, B. K. Ambrose, C. H. Christensen, B. G. Wang, G. Murphy, X. Y. Ye, D. Bhandari, J. Feng, B. Y. Xia, C. S. Sosnoff, F. Kamangar, P. Brennan, P. Boffetta, S. M. Dawsey, C. C. Abnet, R. Malekzadeh and N. D. Freedman | 2019 | Urinary Biomarkers of Carcinogenic Exposure among Cigarette, Waterpipe, and Smokeless Tobacco Users and Never Users of Tobacco in the Golestan Cohort Study | Cancer Epidemiology Biomarkers & Prevention | 10.1158/1055-9965.Epi-18-0743 |
| **29** | P. Fang, W. Jiang, R. Davuluri, C. Xu, S. Krishnan, R. Mohan, A. C. Koong, C. C. Hsu and S. H. Lin | 2018 | High lymphocyte count during neoadjuvant chemoradiotherapy is associated with improved pathologic complete response in esophageal cancer | Radiotherapy and Oncology | 10.1016/j.radonc.2018.02.025 |
| **30** | C. Folgueira, S. Barja-Fernandez, L. Prado, O. Al-Massadi, C. Castelao, V. Pena-Leon, P. Gonzalez-Saenz, J. Baltar, I. Baamonde, R. Leis, C. Dieguez, U. Pagotto, F. F. Casanueva, S. A. Tovar, R. Nogueiras and L. M. Seoane | 2017 | Pharmacological inhibition of cannabinoid receptor 1 stimulates gastric release of nesfatin-1 <i>via</i> the mTOR pathway | World Journal of Gastroenterology | 10.3748/wjg.v23.i35.6403 |
| **31** | M. A. García-González, L. Bujanda, E. Quintero, S. Santolaria, R. Benito, M. Strunk, F. Sopeña, C. Thomson, A. Pérez-Aisa, D. Nicolás-Pérez, E. Hijona, P. Carrera-Lasfuentes, E. Piazuelo, P. Jiménez, J. Espinel, R. Campo, M. Manzano, F. Geijo, M. Pellise, M. Zaballa, F. González-Huix, J. Espinós, L. Titó, L. Barranco, R. Pazo-Cid and A. Lanas | 2015 | Association of <i>PSCA</i> rs2294008 gene variants with poor prognosis and increased susceptibility to gastric cancer and decreased risk of duodenal ulcer disease | International Journal of Cancer | 10.1002/ijc.29500 |
| **32** | P. Gharahkhani, J. Tung, D. Hinds, A. Mishra, T. L. Vaughan, D. C. Whiteman, S. MacGregor, E. Barrett's and B. S. Investigators | 2016 | Chronic gastroesophageal reflux disease shares genetic background with esophageal adenocarcinoma and Barrett's esophagus | Human Molecular Genetics | 10.1093/hmg/ddv512 |
| **33** | S. Ghosh, B. Bankura, S. Ghosh, M. L. Saha, A. K. Pattanayak, S. Ghatak, M. Guha, S. K. Nachimuthu, C. K. Panda, S. Maji, S. Chakraborty, B. Maity and M. Das | 2017 | Polymorphisms in <i>ADH1B</i> and <i>ALDH2</i> genes associated with the increased risk of gastric cancer in West Bengal, India | Bmc Cancer | 10.1186/s12885-017-3713-7 |
| **34** | S. Ghosh, S. Ghosh, B. Bankura, M. L. Saha, S. Maji, S. Ghatak, A. K. Pattanayak, S. Sadhukhan, M. Guha, S. K. Nachimuthu, C. K. Panda, B. Maity and M. Das | 2016 | Association of DNA repair and xenobiotic pathway gene polymorphisms with genetic susceptibility to gastric cancer patients in West Bengal, India | Tumor Biology | 10.1007/s13277-015-4780-5 |
| **35** | E. Gkogkou, G. Barnasas, K. Vougas and I. P. Trougakos | 2020 | Expression profiling meta-analysis of ACE2 and TMPRSS2, the putative anti-inflammatory receptor and priming protease of SARS-CoV-2 in human cells, and identification of putative modulators | Redox Biology | 10.1016/j.redox.2020.101615 |
| **36** | Q. H. Guo, H. Lu, J. Wang, Z. F. Chen, Y. P. Wang, R. Ji, Q. Li, Y. Zhao, H. L. Zhang and Y. N. Zhou | 2016 | Association between TAP1 gene polymorphism and esophageal cancer in a Han Gansu population | International Journal of Clinical and Experimental Medicine |  |
| **37** | E. Ha and J. H. Bae | 2018 | Zinc transporter <i>SLC39A11</i> polymorphisms are associated with chronic gastritis in the Korean population: the possible effect on spicy food intake | Nutrition Research | 10.1016/j.nutres.2018.04.014 |
| **38** | S. Hardikar, L. Onstad, X. L. Song, A. M. Wilson, T. J. Montine, M. Kratz, G. L. Anderson, P. L. Blount, B. J. Reid, E. White and T. L. Vaughan | 2014 | Inflammation and Oxidative Stress Markers and Esophageal Adenocarcinoma Incidence in a Barrett's Esophagus Cohort | Cancer Epidemiology Biomarkers & Prevention | 10.1158/1055-9965.Epi-14-0384 |
| **39** | M. Hirata, Y. Kamatani, A. Nagai, Y. Kiyohara, T. Ninomiya, A. Tamakoshi, Z. Yamagata, M. Kubo, K. Muto, T. Mushiroda, Y. Murakami, K. Yuji, Y. Furukawa, H. Zembutsu, T. Tanaka, Y. Ohnishi, Y. Nakamura, K. Matsuda and G. BioBank Japan Cooperative Hosp | 2017 | Cross-sectional analysis of BioBank Japan clinical data: A large cohort of 200,000 patients with 47 common diseases | Journal of Epidemiology | 10.1016/j.je.2016.12.003 |
| **40** | Y. H. Huang, Q. R. Hu, Z. X. Wei, L. Chen, Y. Luo, X. J. Li and C. P. Li | 2023 | Influence of <i>MTHFR</i> polymorphism, alone or in combination with smoking and alcohol consumption, on cancer susceptibility | Open Life Sciences | 10.1515/biol-2022-0680 |
| **41** | M. Z. Irani, N. J. Talley, J. Ronkainen, P. Aro, A. Andreasson, L. Agreus, M. Vieth, M. P. Jones and M. M. Walker | 2021 | Neutrophils, eosinophils, and intraepithelial lymphocytes in the squamous esophagus in subjects with and without gastroesophageal reflux symptoms | Human Pathology | 10.1016/j.humpath.2021.06.004 |
| **42** | N. S. Irhayyim, M. A. A. Ahmed and H. J. Mahmood | 2018 | Evaluation of salivary Aspartate Aminotransferase Enzyme level in Smoker Patients with Peptic Ulcer in Relation to Periodontal Condition | Research Journal of Pharmaceutical Biological and Chemical Sciences |  |
| **43** | A. Izzotti, M. Longobardi, S. La Maestra, R. T. Micale, A. Pulliero, A. Camoirano, M. Geretto, F. D'Agostini, R. Balansky, M. S. Miller, V. E. Steele and S. De Flora | 2018 | Release of MicroRNAs into Body Fluids from Ten Organs of Mice Exposed to Cigarette Smoke | Theranostics | 10.7150/thno.22726 |
| **44** | A. Jia, Y. Wu, W. L. Ren, P. Han and Y. Shao | 2020 | Genetic variations of <i>CARMN</i> affect risk of esophageal cancer in northwest China | Gene | 10.1016/j.gene.2020.144680 |
| **45** | D. X. Jiang, Q. Song, F. H. Zhang, C. Xu, X. J. Li, H. Y. Zeng, J. Su, J. Huang, Y. F. Xu, S. H. Lu and Y. Y. Hou | 2023 | Prognostic significance of CCND1 amplification/overexpression in smoking patients with esophageal squamous cell carcinoma | Cancer Genetics | 10.1016/j.cancergen.2023.07.004 |
| **46** | W. P. Jiao, J. Y. Zhang, Y. Y. Wei, J. H. Feng, M. Ma, H. Z. Zhao, L. H. Wang and W. J. Jiao | 2019 | MiR-139-5p regulates VEGFR and downstream signaling pathways to inhibit the development of esophageal cancer | Digestive and Liver Disease | 10.1016/j.dld.2018.07.017 |
| **47** | Z. Kaya and S. Gursoy | 2023 | Association Between CYP1A1 Polymorphisms and Esophageal Cancer Susceptibility: A Case-control Study | In Vivo | 10.21873/invivo.13155 |
| **48** | L. E. Kelemen, M. Earp, B. L. Fridley, G. Chenevix-Trench, P. A. Fasching, M. W. Beckmann, A. B. Ekici, A. Hein, D. Lambrechts, S. Lambrechts, E. Van Nieuwenhuysen, I. Vergote, M. A. Rossing, J. A. Doherty, J. Chang-Claude, S. Behrens, K. B. Moysich, R. Cannioto, S. Lele, K. Odunsi, M. T. Goodman, Y. B. Shvetsov, P. J. Thompson, L. R. Wilkens, T. Dörk, N. Antonenkova, N. Bogdanova, P. Hillemanns, I. B. Runnebaum, A. du Bois, P. Harter, F. Heitz, I. Schwaab, R. Butzow, L. M. Pelttari, H. Nevanlinna, F. Modugno, R. P. Edwards, J. L. Kelley, R. B. Ness, B. Y. Karlan, J. Lester, S. Orsulic, C. Walsh, S. K. Kjær, A. Jensen, J. M. Cunningham, R. A. Vierkant, G. G. Giles, F. Bruinsma, M. C. Southey, M. A. T. Hildebrandt, D. Liang, K. R. Lu, X. F. Wu, T. A. Sellers, D. A. Levine, J. M. Schildkraut, E. S. Iversen, K. L. Terry, D. W. Cramer, S. S. Tworoger, E. M. Poole, E. V. Bandera, S. H. Olson, I. Orlow, L. C. V. Thomsen, L. Bjorge, C. Krakstad, I. L. Tangen, L. A. Kiemeney, K. K. H. Aben, L. Massuger, A. M. van Altena, T. Pejovic, Y. Bean, M. Kellar, L. S. Cook, N. D. Le, A. Brooks-Wilson, J. Gronwald, C. Cybulski, A. Jakubowska, J. Lubinski, N. Wentzensen, L. A. Brinton, J. Lissowska, E. Hogdall, S. A. Engelholm, C. Hogdall, L. Lundvall, L. Nedergaard, P. D. P. Pharoah, E. Dicks, H. L. Song, J. P. Tyrer, I. McNeish, N. Siddiqui, K. Carty, R. Glasspool, J. Paul, I. G. Campbell, D. Eccles, A. S. Whittemore, V. McGuire, J. H. Rothstein, W. Sieh, S. A. Narod, C. M. Phelan, J. R. McLaughlin, H. A. Risch, H. Anton-Culver, A. Ziogas, U. Menon, S. A. Gayther, A. Gentry-Maharaj, S. J. Ramus, A. H. Wu, C. L. Pearce, A. W. Lee, M. C. Pike, J. Kupryjanczyk, A. Podgorska, J. Plisiecka-Halasa, W. Sawicki, E. L. Goode, A. Berchuck, G. Australian Ovarian Canc Study and C. Ovarian Cancr Assoc | 2018 | rs495139 in the <i>TYMS</i>-<i>ENOSF1</i> Region and Risk of Ovarian Carcinoma of Mucinous Histology | International Journal of Molecular Sciences | 10.3390/ijms19092473 |
| **49** | H. S. Kim, S. E. Lee, Y. S. Bae, D. J. Kim, C. G. Lee, J. Hur, H. Chung, J. C. Park, D. H. Jung, S. K. Shin, S. K. Lee, Y. C. Lee, H. R. Kim, Y. W. Moon, J. H. Kim, Y. M. Shim, S. S. Jewell, H. Kim, Y. L. Choi and B. C. Cho | 2015 | Fibroblast growth factor receptor 1 gene amplification is associated with poor survival in patients with resected esophageal squamous cell carcinoma | Oncotarget | 10.18632/oncotarget.2944 |
| **50** | R. Kinoshita-Daitoku, K. Kiga, M. Miyakoshi, R. Otsubo, Y. Ogura, T. Sanada, Z. Bo, T. V. Phuoc, T. Okano, T. Iida, R. Yokomori, E. Kuroda, S. Hirukawa, M. Tanaka, A. Sood, P. Subsomwong, H. Ashida, T. T. Binh, L. T. Nguyen, K. V. Van, D. Q. D. Ho, K. Nakai, T. Suzuki, Y. Yamaoka, T. Hayashi and H. Mimuro | 2021 | A bacterial small RNA regulates the adaptation of <i>Helicobacter pylori</i> to the host environment | Nature Communications | 10.1038/s41467-021-22317-7 |
| **51** | S. Kizildag, F. Hosgorler, G. Güvendi, T. B. Koc, S. Kandis, A. Argon, M. Ates and N. Uysal | 2021 | Nicotine lowers TNF-α, IL-1b secretion and leukocyte accumulation via nAChR in rat stomach | Toxin Reviews | 10.1080/15569543.2020.1790604 |
| **52** | M. Kohailan, M. Alanazi, M. Rouabhia, A. Alamri, N. R. Parine, A. Alhadheq, S. Basavarajappa, A. A. A. Al-Kheraif and B. Semlali | 2016 | Effect of smoking on the genetic makeup of toll-like receptors 2 and 6 | Oncotargets and Therapy | 10.2147/ott.S109650 |
| **53** | G. J. Korpanty, L. Eng, X. Qiu, O. O. Faluyi, D. J. Renouf, D. X. Cheng, D. Patel, Z. Chen, B. C. Tse, J. J. Knox, L. Dodbiba, J. Teichman, A. K. Azad, R. Wong, G. Darling, D. Reisman, S. Cuffe, G. Liu and W. Xu | 2017 | Association of BRM promoter polymorphisms and esophageal adenocarcinoma outcome | Oncotarget | 10.18632/oncotarget.15890 |
| **54** | S. S. Kumar, V. Gunda, D. M. Reinartz, K. W. Pond, C. A. Thorne, P. V. S. Raj, M. D. L. Johnson and J. E. Wilson | 2024 | Oral streptococci <i>S. anginosus</i> and <i>S. mitis</i> induce distinct morphological, inflammatory, and metabolic signatures in macrophages | Infection and Immunity | 10.1128/iai.00536-23 |
| **55** | C. Lam, W. F. Liu, R. D. Bel, K. Chan, L. Miller, M. C. Brown, Z. Chen, D. Cheng, D. Patel, W. Xu, G. E. Darling and G. Liu | 2017 | Polymorphisms of the FOXF1 and MHC locus genes in individuals undergoing esophageal acid reflux assessments | Diseases of the Esophagus | 10.1111/dote.12456 |
| **56** | E. Lee, D. O. Stram, W. E. Ek, L. E. Onstad, S. MacGregor, P. Gharahkhani, W. M. Ye, J. Lagergren, N. J. Shaheen, L. J. Murray, L. J. Hardie, M. D. Gammon, W. H. Chow, H. A. Risch, D. A. Corley, D. M. Levine, D. C. Whiteman, L. Bernstein, N. C. Bird, T. L. Vaughan and A. H. Wu | 2015 | Pleiotropic Analysis of Cancer Risk Loci on Esophageal Adenocarcinoma Risk | Cancer Epidemiology Biomarkers & Prevention | 10.1158/1055-9965.Epi-15-0596 |
| **57** | H. K. Lee, M. J. Kwon, Y. J. Ra, H. S. Lee, H. S. Kim, E. S. Nam, S. J. Cho, H. R. Park, S. K. Min, J. Seo, J. Y. Choe, K. W. Min and S. Y. Kang | 2020 | Significance of druggable targets (PD-L1, KRAS, BRAF, PIK3CA, MSI, and HPV) on curatively resected esophageal squamous cell carcinoma | Diagnostic Pathology | 10.1186/s13000-020-01045-4 |
| **58** | J. H. Lee, E. Y. Kim, C. K. Park, S. Y. Lee, M. K. Lee, S. H. Yoon, J. E. Lee, S. H. Lee, S. J. Kim, S. Y. Lee, J. H. Lim, T. W. Jang, S. H. Jang, K. Y. Lee, S. H. Lee, S. H. Yang, D. W. Park, C. K. Park, H. S. Kang, C. D. Yeo, C. M. Choi and J. C. Lee | 2023 | Real-World Study of Osimertinib in Korean Patients with Epidermal Growth Factor Receptor T790M Mutation-Positive Non-Small Cell Lung Cancer | Cancer Research and Treatment | 10.4143/crt.2022.381 |
| **59** | H. L. Lehman, X. B. Yang, P. A. Welsh and D. B. Stairs | 2015 | p120-Catenin Down-Regulation and Epidermal Growth Factor Receptor Overexpression Results in a Transformed Epithelium That Mimics Esophageal Squamous Cell Carcinoma | American Journal of Pathology | 10.1016/j.ajpath.2014.09.008 |
| **60** | M. D. Li, L. Qiu, G. S. Jia, R. Q. Guo and Q. B. Leng | 2020 | Single-cell expression profiles of <i>ACE2</i> and <i>TMPRSS2</i> reveals potential vertical transmission and fetus infection of SARS-CoV-2 | Aging-Us | 10.18632/aging.104015 |
| **61** | S. Y. Li, Y. Yoshida, E. Kobayashi, A. Adachi, S. Hirono, T. Matsutani, S. Mine, T. Machida, M. Ohno, E. Nishi, Y. Maezawa, M. Takemoto, K. Yokote, K. Kitamura, M. Sumazaki, M. Ito, H. Shimada, H. Takizawa, K. Kashiwado, G. Tomiyoshi, N. Shinmen, R. Nakamura, H. Kuroda, X. M. Zhang, H. Wang, K. Goto, Y. Iwadate and T. Hiwasa | 2020 | Association between serum anti-ASXL2 antibody levels and acute ischemic stroke, acute myocardial infarction, diabetes mellitus, chronic kidney disease and digestive organ cancer, and their possible association with atherosclerosis and hypertension | International Journal of Molecular Medicine | 10.3892/ijmm.2020.4690 |
| **62** | W. Li, L. Y. Zhang, B. B. Guo, J. Q. Deng, S. Q. Wu, F. Li, Y. R. Wang, J. C. Lu and Y. F. Zhou | 2019 | Exosomal <i>FMR1-AS1</i> facilitates maintaining cancer stem-like cell dynamic equilibrium via TLR7/NFB/c-Myc signaling in female esophageal carcinoma | Molecular Cancer | 10.1186/s12943-019-0949-7 |
| **63** | Y. Y. Li, R. Ghanbari, W. Pathmasiri, S. McRitchie, H. Poustchi, A. Shayanrad, G. Roshandel, A. Etemadi, J. D. Pollock, R. Malekzadeh and S. C. J. Sumner | 2020 | Untargeted Metabolomics: Biochemical Perturbations in Golestan Cohort Study Opium Users Inform Intervention Strategies | Frontiers in Nutrition | 10.3389/fnut.2020.584585 |
| **64** | Z. F. Liang, R. Wu, W. Xie, H. Geng, L. Zhao, C. F. Xie, J. S. Wu, S. S. Geng, X. T. Li, M. M. Zhu, W. W. Zhu, J. Y. Zhu, C. Huang, X. Ma, C. Y. Zhong and H. Y. Han | 2015 | Curcumin Suppresses MAPK Pathways to Reverse Tobacco Smoke-induced Gastric Epithelial-Mesenchymal Transition in Mice | Phytotherapy Research | 10.1002/ptr.5398 |
| **65** | T. F. Lin, C. L. Bi, Y. Song, H. Y. Guo, L. S. Liu, Z. Y. Zhou, B. Y. Wang, G. F. Tang, C. Z. Liu, Y. Yang, W. H. Ling, J. G. Yang, Y. M. Cui, C. G. Zhang, G. Li, J. A. Li, J. P. Li, Y. Zhang, Y. Huo, X. B. Wang, H. Zhang, X. H. Qin and X. P. Xu | 2021 | Plasma Magnesium Concentrations and Risk of Incident Cancer in Adults with Hypertension: A Nested Case-Control Study | Annals of Nutrition and Metabolism | 10.1159/000510214 |
| **66** | S. Y. Liu, W. Chen, E. A. Chughtai, Z. Qiao, J. T. Jiang, S. M. Li, W. Zhang and J. Zhang | 2017 | <i>PIK3CA</i> gene mutations in Northwest Chinese esophageal squamous cell carcinoma | World Journal of Gastroenterology | 10.3748/wjg.v23.i14.2585 |
| **67** | W. J. Liu, J. M. Snell, W. R. Jeck, K. A. Hoadley, M. D. Wilkerson, J. S. Parker, N. Patel, Y. B. Mlombe, G. Mulima, N. G. Liomba, L. L. Wolf, C. G. Shores, S. Gopal and N. E. Sharpless | 2016 | Subtyping sub-Saharan esophageal squamous cell carcinoma by comprehensive molecular analysis | Jci Insight | 10.1172/jci.insight.88755 |
| **68** | Y. Liu, H. J. Lai, R. Zhang, L. Xia and L. X. Liu | 2023 | Causal relationship between gastro-esophageal reflux disease and risk of lung cancer: insights from multivariable Mendelian randomization and mediation analysis | International Journal of Epidemiology | 10.1093/ije/dyad090 |
| **69** | A. B. Lopes, M. Metzdorf, L. Metzdorf, M. P. R. Sousa, C. Kavalco, A. Etemadi, N. R. Pritchett, G. Murphy, A. M. Calafat, C. C. Abnet, S. M. Dawsey and R. B. Fagundes | 2018 | Urinary Concentrations of Polycyclic Aromatic Hydrocarbon Metabolites in <i>Mate</i> Drinkers in Rio Grande do Sul, Brazil | Cancer Epidemiology Biomarkers & Prevention | 10.1158/1055-9965.Epi-17-0773 |
| **70** | D. J. Lubin, R. Mick, S. G. Shroff, K. Stashek and E. E. Furth | 2018 | The notch pathway is activated in neoplastic progression in esophageal squamous cell carcinoma | Human Pathology | 10.1016/j.humpath.2017.11.004 |
| **71** | M. J. Machiela, C. A. Hsiung, X. O. Shu, W. J. Seow, Z. M. Wang, K. Matsuo, Y. C. Hong, A. Seow, C. Wu, H. D. Hosgood, K. X. Chen, J. C. Wang, W. Q. Wen, R. Cawthon, N. Chatterjee, W. Hu, N. E. Caporaso, J. Y. Park, C. J. Chen, Y. H. Kim, Y. T. Kim, M. T. Landi, H. B. Shen, C. Lawrence, L. Burdett, M. Yeager, I. S. Chang, T. Mitsudomi, H. N. Kim, G. C. Chang, B. A. Bassig, M. Tucker, F. S. Wei, Z. H. Yin, S. J. An, B. Y. Qian, V. H. F. Lee, D. R. Lu, J. J. Liu, H. S. Jeon, C. F. Hsiao, J. S. Sung, J. H. Kim, Y. T. Gao, Y. H. Tsai, Y. J. Jung, H. Guo, Z. B. Hu, A. Hutchinson, W. C. Wang, R. J. Klein, C. C. Chung, I. J. Oh, K. Y. Chen, S. I. Berndt, W. Wu, J. Chang, X. C. Zhang, M. S. Huang, H. Zheng, J. W. Wang, X. Y. Zhao, Y. Q. Li, J. E. Choi, W. C. Su, K. H. Park, S. W. Sung, Y. M. Chen, L. Liu, C. H. Kang, L. M. Hu, C. H. Chen, W. Pao, Y. C. Kim, T. Y. Yang, J. Xu, P. Guan, W. Tan, J. Su, C. L. Wang, H. X. Li, A. D. L. Sihoe, Z. H. Zhao, Y. Chen, Y. Y. Choi, J. Y. Hung, J. S. Kim, H. I. Yoon, Q. Y. Cai, C. C. Lin, I. K. Park, P. Xu, J. Dong, C. Kim, Q. C. He, R. P. Perng, T. Kohno, S. S. Kweon, C. Y. Chen, R. C. H. Vermeulen, J. J. Wu, W. Y. Lim, K. C. Chen, W. H. Chow, B. T. Ji, J. K. C. Chan, M. J. Chu, Y. J. Li, J. Yokota, J. H. Li, H. Y. Chen, Y. B. Xiang, C. J. Yu, H. Kunitoh, G. P. Wu, L. Jin, Y. L. Lo, K. Shiraishi, Y. H. Chen, H. C. Lin, T. C. Wu, M. P. Wong, Y. L. Wu, P. C. Yang, B. S. Zhou, M. H. Shin, J. F. Fraumeni, W. Zheng, D. X. Lin, S. J. Chanock, N. Rothman and Q. Lan | 2015 | Genetic variants associated with longer telomere length are associated with increased lung cancer risk among never-smoking women in Asia: a report from the female lung cancer consortium in Asia | International Journal of Cancer | 10.1002/ijc.29393 |
| **72** | S. Maeda, K. Mure, K. Mugitani, Y. Watanabe, M. Iwane, O. Mohara and T. Takeshita | 2014 | Roles of the ALDH2 and ADH1B Genotypes on the Association Between Alcohol Intake and Serum Adiponectin Levels Among Japanese Male Workers | Alcoholism-Clinical and Experimental Research | 10.1111/acer.12406 |
| **73** | K. R. Majeed, W. Y. Al-Ani and M. M. AlShock | 2022 | Study the effect of Sex hormones in patients with stomach ulcers in Anbar Governorate | Journal of Pharmaceutical Negative Results | 10.47750/pnr.2022.13.S01.218 |
| **74** | S. Mantziari, A. Pomoni, J. O. Prior, M. Winiker, P. Allemann, N. Demartines and M. Schäfer | 2020 | <SUP>18</SUP>F- FDG PET/CT-derived parameters predict clinical stage and prognosis of esophageal cancer | Bmc Medical Imaging | 10.1186/s12880-019-0401-x |
| **75** | M. Matejcic, C. G. Mathew and M. I. Parker | 2019 | The Relationship Between Environmental Exposure and Genetic Architecture of the 2q33 Locus With Esophageal Cancer in South Africa | Frontiers in Genetics | 10.3389/fgene.2019.00406 |
| **76** | Y. Mei, D. Liang, T. J. Wang and D. Yu | 2021 | Gaining insights into relevance across cancers based on mutation features of TP53 gene | Biochemistry and Biophysics Reports | 10.1016/j.bbrep.2021.101165 |
| **77** | Y. Mei, D. Liang, T. J. Wang and D. Yu | 2021 | Gaining insights into relevance across cancers based on mutation features of TP53 gene | Biochemistry and Biophysics Reports | 10.1016/j.bbrep.2021.101165 |
| **78** | D. Nasrollahzadeh, G. Roshandel, T. M. Delhomme, P. H. Avogbe, M. Foll, F. Saidi, H. Poustchi, M. Sotoudeh, R. Malekzadeh, P. Brennan, J. McKay, P. Hainaut and B. Abedi-Ardekani | 2021 | <i>TP53</i> Targeted Deep Sequencing of Cell-Free DNA in Esophageal Squamous Cell Carcinoma Using Low-Quality Serum: Concordance with Tumor Mutation | International Journal of Molecular Sciences | 10.3390/ijms22115627 |
| **79** | N. L. T. Nguyen, N. D. T. Dang, Q. H. Dang, V. C. Tran, H. L. Vo, M. Yamaguchi and T. V. Ta | 2021 | Polymorphism of <i>MUC1</i> Gene in Vietnamese Gastric Cancer Patients: A Multicenter Case-Control Study | Frontiers in Oncology | 10.3389/fonc.2021.694977 |
| **80** | N. L. T. Nguyen, N. D. T. Dang, Q. V. Vu, A. K. Dang and T. V. Ta | 2023 | A Model for Gastric Cancer Risk Prediction Based on<i> MUC1</i> Polymorphisms and Health-risk Behaviors in a Vietnamese Population | In Vivo | 10.21873/invivo.13339 |
| **81** | T. Nguyen, Z. W. Tang, M. Younes, A. Alsarraj, D. Ramsey, S. Fitzgerald, J. R. Kramer and H. B. El-Serag | 2015 | Esophageal COX-2 Expression Is Increased in Barrett's Esophagus, Obesity, and Smoking | Digestive Diseases and Sciences | 10.1007/s10620-014-3333-x |
| **82** | J. S. Ong, J. Y. An, X. K. Han, M. H. Law, P. Nandakumar, J. Schumacher, I. Gockel, A. Bohmer, J. Jankowski, C. Palles, C. M. Olsen, R. E. Neale, R. Fitzgerald, A. P. Thrift, T. L. Vaughan, M. F. Buas, D. A. Hinds, P. Gharahkhani, B. J. Kendall, S. MacGregor, T. andMe Res and C. Esophageal Canc | 2022 | Multitrait genetic association analysis identifies 50 new risk loci for gastro-oesophageal reflux, seven new loci for Barrett's oesophagus and provides insights into clinical heterogeneity in reflux diagnosis | Gut | 10.1136/gutjnl-2020-323906 |
| **83** | J. S. Ong, P. Gharahkhani, T. L. Vaughan, D. Whiteman, B. J. Kendall and S. MacGregor | 2022 | Assessing the genetic relationship between gastro-esophageal reflux disease and risk of COVID-19 infection | Human Molecular Genetics | 10.1093/hmg/ddab253 |
| **84** | H. Qiu, X. T. Lin, W. F. Tang, C. Liu, Y. Chen, H. Ding, M. Q. Kang and S. C. Chen | 2017 | Investigation of <i>TCF7L2</i>, <i>LEP</i> and <i>LEPR</i> polymorphisms with esophageal squamous cell carcinomas | Oncotarget | 10.18632/oncotarget.22619 |
| **85** | H. Qiu, Y. F. Wang, M. Q. Kang, H. Ding, C. Liu, W. F. Tang, Z. Z. Xiao and Y. Chen | 2017 | The relationship between <i>IGF2BP2</i> and <i>PPARG</i> polymorphisms and susceptibility to esophageal squamous-cell carcinomas in the eastern Chinese Han population | Oncotargets and Therapy | 10.2147/ott.S145776 |
| **86** | M. M. Rahman, M. A. Sarker, M. M. Hossain, M. S. Alam, M. Islam, L. Shirin, R. Sultana and G. N. N. Sultana | 2019 | Association of <i>p53</i> Gene Mutation With <i>Helicobacter pylori</i> Infection in Gastric Cancer Patients and Its Correlation With Clinicopathological and Environmental Factors | World Journal of Oncology | 10.14740/wjon1087 |
| **87** | J. N. Sampson, W. A. Wheeler, M. Yeager, O. Panagiotou, Z. Wang, S. I. Berndt, Q. Lan, C. C. Abnet, L. T. Amundadottir, J. D. Figueroa, M. T. Landi, L. Mirabello, S. A. Savage, P. R. Taylor, I. De Vivo, K. A. McGlynn, M. P. Purdue, P. Rajaraman, H. O. Adami, A. Ahlbom, D. Albanes, M. F. Amary, S. J. An, U. Andersson, G. Andriole, I. L. Andrulis, E. Angelucci, S. M. Ansell, C. Arici, B. K. Armstrong, A. A. Arslan, M. A. Austin, D. Baris, D. A. Barkauskas, B. A. Bassig, N. Becker, Y. Benavente, S. Benhamou, C. Berg, D. Van Den Berg, L. Bernstein, K. A. Bertrand, B. M. Birmann, A. Black, H. Boeing, P. Boffetta, M. C. Boutron-Ruault, P. M. Bracci, L. Brinton, A. R. Brooks-Wilson, H. B. Bueno-De-Mesquita, L. Burdett, J. Buring, M. A. Butler, Q. Y. Cai, G. Cancel-Tassin, F. Canzian, A. Carrato, T. Carreon, A. Carta, J. K. C. Chan, E. T. Chang, G. C. Chang, I. S. Chang, J. Chang, J. Chang-Claude, C. J. Chen, C. Y. Chen, C. Chen, C. H. Chen, C. Chen, H. Y. Chen, K. X. Chen, K. Y. Chen, K. C. Chen, Y. Chen, Y. H. Chen, Y. S. Chen, Y. M. Chen, L. H. Chien, M. D. Chirlaque, J. E. Choi, Y. Y. Choi, W. H. Chow, C. C. Chung, J. Clavel, F. Clavel-Chapelon, P. Cocco, J. S. Colt, E. Comperat, L. Conde, J. M. Connors, D. Conti, V. K. Cortessis, M. Cotterchio, W. Cozen, S. Crouch, M. Crous-Bou, O. Cussenot, F. G. Davis, T. Ding, W. R. Diver, M. Dorronsoro, L. Dossus, E. J. Duell, M. G. Ennas, R. L. Erickson, M. Feychting, A. M. Flanagan, L. Foretova, J. F. Fraumeni, N. D. Freedman, L. E. B. Freeman, C. Fuchs, M. Gago-Dominguez, S. Gallinger, Y. T. Gao, S. M. Gapstur, M. Garcia-Closas, R. García-Closas, R. D. Gascoyne, J. Gastier-Foster, M. M. Gaudet, J. M. Gaziano, C. Giffen, G. G. Giles, E. Giovannucci, B. Glimelius, M. Goggins, N. Gokgoz, A. M. Goldstein, R. Gorlick, M. Gross, R. Grubb, J. Gu, P. Guan, M. Gunter, H. Guo, T. M. Habermann, C. A. Haiman, D. Halai, G. Hallmans, M. Hassan, C. Hattinger, Q. C. He, X. Z. He, K. Helzlsouer, B. Henderson, R. Henriksson, H. Hjalgrim, J. Hoffman-Bolton, C. Hohensee, T. R. Holford, E. A. Holly, Y. C. Hong, R. N. Hoover, P. L. Horn-Ross, G. M. M. Hosain, H. D. Hosgood, C. F. Hsiao, N. Hu, W. Hu, Z. B. Hu, M. S. Huang, J. M. Huerta, J. Y. Hung, A. Hutchinson, P. D. Inskip, R. D. Jackson, E. J. Jacobs, M. Jenab, H. S. Jeon, B. T. Ji, G. F. Jin, L. Jin, C. Johansen, A. Johnson, Y. J. Jung, R. Kaaks, A. Kamineni, E. Kane, C. H. Kang, M. R. Karagas, R. S. Kelly, K. T. Khaw, C. Kim, H. N. Kim, J. H. Kim, J. S. Kim, Y. H. Kim, Y. T. Kim, Y. C. Kim, C. M. Kitahara, A. P. Klein, R. J. Klein, M. Kogevinas, T. Kohno, L. N. Kolonel, C. Kooperberg, A. Kricker, V. Krogh, H. Kunitoh, R. C. Kurtz, S. S. Kweon, A. LaCroix, C. Lawrence, F. Lecanda, V. H. F. Lee, D. H. Li, H. X. Li, J. H. Li, Y. J. Li, Y. Q. Li, L. M. Liao, M. Liebow, T. Lightfoot, W. Y. Lim, C. C. Lin, D. X. Lin, S. Lindstrom, M. S. Linet, B. K. Link, C. W. Liu, J. J. Liu, L. Liu, B. Ljungberg, J. Lloreta, S. Di Lollo, D. Lu, E. Lund, N. Malats, S. Mannisto, L. Le Marchand, N. Marina, G. Masala, G. Mastrangelo, K. Matsuo, M. Maynadie, J. McKay, R. McKean-Cowdin, M. Melbye, B. S. Melin, D. S. Michaud, T. Mitsudomi, A. Monnereau, R. Montalvan, L. E. Moore, L. M. Mortensen, A. Nieters, K. E. North, A. J. Novak, A. L. Oberg, K. Offit, I. J. Oh, S. H. Olson, D. Palli, W. Pao, I. K. Park, J. Y. Park, K. H. Park, A. Patiño-Garcia, S. Pavanello, P. H. M. Peeters, R. P. Perng, U. Peters, G. M. Petersen, P. Picci, M. C. Pike, S. Porru, J. Prescott, L. Prokunina-Olsson, B. Qian, Y. L. Qiao, M. Rais, E. Riboli, J. Riby, H. A. Risch, C. Rizzato, R. Rodabough, E. Roman, M. Roupret, A. M. Ruder, S. de Sanjose, G. Scelo, A. Schned, F. Schumacher, K. Schwartz, M. Schwenn, K. Scotlandi, A. Seow, C. Serra, M. Serra, H. D. Sesso, V. W. Setiawan, G. Severi, R. K. Severson, T. D. Shanafelt, H. B. Shen, W. Shen, M. H. Shin, K. Shiraishi, X. O. Shu, A. Siddiq, L. Sierrasesúmaga, A. D. L. Sihoe, C. F. Skibola, A. Smith, M. T. Smith, M. C. Southey, J. J. Spinelli, A. Staines, M. Stampfer, M. C. Stern, V. L. Stevens, R. S. Stolzenberg-Solomon, J. Su, W. C. Su, M. Sund, J. S. Sung, S. W. Sung, W. Tan, W. Tang, A. Tardón, D. Thomas, C. A. Thompson, L. F. Tinker, R. Tirabosco, A. Tjonneland, R. C. Travis, D. Trichopoulos, F. Y. Tsai, Y. H. Tsai, M. Tucker, J. Turner, C. M. Vajdic, R. C. H. Vermeulen, D. J. Villano, P. Vineis, J. Virtamo, K. Visvanathan, J. Wactawski-Wende, C. Y. Wang, C. L. Wang, J. C. Wang, J. W. Wang, F. S. Wei, E. Weiderpass, G. J. Weiner, S. Weinstein, N. Wentzensen, E. White, T. E. Witzig, B. M. Wolpin, M. P. Wong, C. Wu, G. P. Wu, J. J. Wu, T. C. Wu, W. Wu, X. F. Wu, Y. L. Wu, J. S. Wunder, Y. B. Xiang, J. Xu, P. Xu, P. C. Yang, T. Y. Yang, Y. Q. Ye, Z. H. Yin, J. Yokota, H. I. Yoon, C. J. Yu, H. Yu, K. Yu, J. M. Yuan, A. Zelenetz, A. Zeleniuch-Jacquotte, X. C. Zhang, Y. W. Zhang, X. Y. Zhao, Z. H. Zhao, H. Zheng, T. Z. Zheng, W. Zheng, B. S. Zhou, M. Zhu, M. Zucca, S. M. Boca, J. R. Cerhan, G. M. Ferri, P. Hartge, C. A. Hsiung, C. Magnani, L. Miligi, L. M. Morton, K. E. Smedby, L. R. Teras, J. Vijai, S. S. Wang, P. Brennan, N. E. Caporaso, D. J. Hunter, P. Kraft, N. Rothman, D. T. Silverman, S. L. Slager, S. J. Chanock and N. Chatterjee | 2015 | Analysis of Heritability and Shared Heritability Based on Genome-Wide Association Studies for 13 Cancer Types | Jnci-Journal of the National Cancer Institute | 10.1093/jnci/djv279 |
| **88** | Y. H. Sang, L. Shi, Y. H. Wu, W. T. Yang, H. Y. Gu, J. Yin, L. R. X. Yuan, C. Liu, X. Wang, Y. J. Shi, W. F. Tang and Y. B. Chen | 2016 | <i>Epiregulin</i> rs1460008 A&gt;G polymorphism is associated with decreased risk of esophageal squamous cell carcinoma in a Chinese population | International Journal of Clinical and Experimental Medicine |  |
| **89** | C. V. Schneider, K. M. Schneider, A. Teumer, K. L. Rudolph, D. Hartmann, D. J. Rader and P. Strnad | 2022 | Association of Telomere Length With Risk of Disease and Mortality | Jama Internal Medicine | 10.1001/jamainternmed.2021.7804 |
| **90** | W. J. Seow, K. Matsuo, C. A. Hsiung, K. Shiraishi, M. S. Song, H. N. Kim, M. P. Wong, Y. C. Hong, H. D. Hosgood, Z. M. Wang, I. S. Chang, J. C. Wang, N. Chatterjee, M. Tucker, H. Wei, T. Mitsudomi, W. Zheng, J. H. Kim, B. S. Zhou, N. E. Caporaso, D. Albanes, M. H. Shin, L. P. Chung, S. J. An, P. Wang, H. Zheng, Y. Yatabe, X. C. Zhang, Y. T. Kim, X. O. Shu, Y. C. Kim, B. A. Bassig, J. Chang, J. C. M. Ho, B. T. Ji, M. Kubo, Y. Daigo, H. Ito, Y. Momozawa, K. Ashikawa, Y. Kamatani, T. Honda, H. Sakamoto, H. Kunitoh, K. Tsuta, S. I. Watanabe, H. Nokihara, Y. Miyagi, H. Nakayama, S. Matsumoto, M. Tsuboi, K. Goto, Z. H. Yin, J. X. Shi, A. Takahashi, A. Goto, Y. Minamiya, K. Shimizu, K. Tanaka, T. C. Wu, F. S. Wei, J. Y. Y. Wong, F. Matsuda, J. Su, Y. H. Kim, I. J. Oh, F. J. Song, V. H. F. Lee, W. C. Su, Y. M. Chen, G. C. Chang, K. Y. Chen, M. S. Huang, P. C. Yang, H. C. Lin, Y. B. Xiang, A. Seow, J. Y. Park, S. S. Kweon, C. J. Chen, H. X. Li, Y. T. Gao, C. Wu, B. Y. Qian, D. R. Lu, J. J. Liu, H. S. Jeon, C. F. Hsiao, J. S. Sung, Y. H. Tsai, Y. J. Jung, H. Guo, Z. B. Hu, W. C. Wang, C. C. Chung, C. Lawrence, L. Burdett, M. Yeager, K. B. Jacobs, A. Hutchinson, S. I. Berndt, X. Z. He, W. Wu, J. W. Wang, Y. Q. Li, J. E. Choi, K. H. Park, S. W. Sung, L. Liu, C. H. Kang, L. M. Hu, C. H. Chen, T. Y. Yang, J. Xu, P. Guan, W. Tan, C. L. Wang, A. D. L. Sihoe, Y. Chen, Y. Y. Choi, J. Y. Hung, J. S. Kim, H. I. Yoon, Q. Y. Cai, C. C. Lin, I. K. Park, P. Xu, J. Dong, C. Kim, Q. C. He, R. P. Perng, C. Y. Chen, R. Vermeulen, J. J. Wu, W. Y. Lim, K. C. Chen, J. K. C. Chan, M. J. Chu, Y. J. Li, J. H. Li, H. Y. Chen, C. J. Yu, L. Jin, Y. L. Lo, Y. H. Chen, J. F. Fraumeni, J. Liu, T. Yamaji, Y. Yang, B. Hicks, K. Wyatt, S. A. Li, J. C. Dai, H. X. Ma, G. F. Jin, B. Song, Z. H. Wang, S. S. Cheng, X. L. Li, Y. W. Ren, P. Cui, M. Iwasaki, T. Shimazu, S. Tsugane, J. J. Zhu, G. N. Jiang, K. Fei, G. P. Wu, L. H. Chien, H. L. Chen, Y. C. Su, F. Y. Tsai, Y. S. Chen, J. M. Yu, V. L. Stevens, I. A. Laird-Offringa, C. N. Marconett, D. X. Lin, K. X. Chen, Y. L. Wu, M. T. Landi, H. B. Shen, N. Rothman, T. Kohno, S. J. Chanock and Q. Lan | 2017 | Association between GWAS-identified lung adenocarcinoma susceptibility loci and <i>EGFR</i> mutations in never-smoking Asian women, and comparison with findings from Western populations | Human Molecular Genetics | 10.1093/hmg/ddw414 |
| **91** | J. X. Shi, K. Shiraishi, J. Y. Choi, K. Matsuo, T. Y. Chen, J. C. Dai, R. J. Hung, K. X. Chen, X. O. Shu, Y. T. Kim, M. T. Landi, D. X. Lin, W. Zheng, Z. H. Yin, B. S. Zhou, B. Song, J. C. Wang, W. J. Seow, L. Song, I. S. Chang, W. Hu, L. H. Chien, Q. Y. Cai, Y. C. Hong, H. N. Kim, Y. L. Wu, M. P. Wong, B. D. Richardson, K. M. Funderburk, S. L. Li, T. W. Zhang, C. Breeze, Z. M. Wang, B. Blechter, B. A. Bassig, J. H. Kim, D. Albanes, J. Y. Y. Wong, M. H. Shin, L. P. Chung, Y. Yang, S. J. An, H. Zheng, Y. Yatabe, X. C. Zhang, Y. C. Kim, N. E. Caporaso, J. Chang, J. C. M. Ho, M. Kubo, Y. Daigo, M. Song, Y. Momozawa, Y. Kamatani, M. Kobayashi, K. Okubo, T. Honda, D. H. Hosgood, H. Kunitoh, H. Patel, S. Watanabe, Y. Miyagi, H. Nakayama, S. Matsumoto, H. Horinouchi, M. Tsuboi, R. Hamamoto, K. Goto, Y. Ohe, A. Takahashi, A. Goto, Y. Minamiya, M. Hara, Y. Nishida, K. Takeuchi, K. Wakai, K. Matsuda, Y. Murakami, K. Shimizu, H. Suzuki, M. Saito, Y. Ohtaki, K. Tanaka, T. Wu, F. Wei, H. Dai, M. J. Machiela, J. Su, Y. H. Kim, I. J. Oh, V. H. F. Lee, G. C. Chang, Y. H. Tsai, K. Y. Chen, M. S. Huang, W. C. Su, Y. M. Chen, A. Seow, J. Y. Park, S. S. Kweon, K. C. Chen, Y. T. Gao, B. Y. Qian, C. Wu, D. R. Lu, J. J. Liu, A. G. Schwartz, R. Houlston, M. R. Spitz, I. P. Gorlov, X. F. Wu, P. Yang, S. Lam, A. Tardon, C. Chen, S. E. Bojesen, M. Johansson, A. Risch, H. Bickeböller, B. T. Ji, H. E. Wichmann, D. C. Christiani, G. Rennert, S. Arnold, P. Brennan, J. McKay, J. K. Field, S. S. Shete, L. Le Marchand, G. Liu, A. Andrew, L. A. Kiemeney, S. Zienolddiny-Narui, K. Grankvist, M. Johansson, A. Cox, F. Taylor, J. M. Yuan, P. Lazarus, M. B. Schabath, M. C. Aldrich, H. S. Jeon, S. S. Jiang, J. S. Sung, C. H. Chen, C. F. Hsiao, Y. J. Jung, H. Guo, Z. B. Hu, L. Burdett, M. Yeager, A. Hutchinson, B. Hicks, J. Liu, B. Zhu, S. I. Berndt, W. Wu, J. W. Wang, Y. Q. Li, J. E. Choi, K. H. Park, S. W. Sung, L. Liu, C. H. Kang, W. C. Wang, J. Xu, P. Guan, W. Tan, C. J. Yu, G. Yang, A. D. L. Sihoe, Y. Chen, Y. Y. Choi, J. S. Kim, H. I. Yoon, I. K. Park, P. Xu, Q. C. He, C. L. Wang, H. H. Hung, R. C. H. Vermeulen, I. Cheng, J. J. Wu, W. Y. Lim, F. Y. Tsai, J. K. C. Chan, J. H. Li, H. Y. Chen, H. C. Lin, L. Jin, J. Liu, N. Sawada, T. Yamaji, K. Wyatt, S. A. Li, H. X. Ma, M. Zhu, Z. H. Wang, S. S. Cheng, X. L. Li, Y. W. Ren, A. Chao, M. Iwasaki, J. J. Zhu, G. N. Jiang, K. Fei, G. P. Wu, C. Y. Chen, C. J. Chen, P. C. Yang, J. M. Yu, V. L. Stevens, J. F. Fraumeni, N. Chatterjee, O. Y. Gorlova, C. A. Hsiung, C. I. Amos, H. B. Shen, S. J. Chanock, N. Rothman, T. Kohno and Q. Lan | 2023 | Genome-wide association study of lung adenocarcinoma in East Asia and comparison with a European population | Nature Communications | 10.1038/s41467-023-38196-z |
| **92** | J. S. Shirvani, M. Salehi, A. R. Majd, F. Sadeghi, E. Ferdosi-Shahandashti, S. Khafri and M. Rajabnia | 2023 | Expression Assessment of the <i>Helicobacter pylori babA</i> and <i>sabA</i> Genes in Patients with Peptic Ulcer, Duodenal Ulcer and Gastric Cancer | International Journal of Molecular and Cellular Medicine | 10.22088/ijmcm.Bums.12.2.211 |
| **93** | H. Shoji, H. Isomoto, A. Yoshida, H. Ikeda, H. Minami, T. Kanda, S. Urabe, K. Matsushima, F. Takeshima, K. Nakao and H. Inoue | 2017 | MicroRNA-130a is highly expressed in the esophageal mucosa of achalasia patients | Experimental and Therapeutic Medicine | 10.3892/etm.2017.4598 |
| **94** | P. Sinha, B. H. Haughey, D. Kallogjeri and R. S. Jackson | 2019 | Long-term analysis of transorally resected p16+Oropharynx cancer: Outcomes and prognostic factors | Laryngoscope | 10.1002/lary.27472 |
| **95** | Z. Strizova, M. Snajdauf, D. Stakheev, P. Taborska, J. Vachtenheim, J. Biskup, R. Lischke, J. Bartunkova and D. Smrz | 2020 | The paratumoral immune cell signature reveals the potential for the implementation of immunotherapy in esophageal carcinoma patients | Journal of Cancer Research and Clinical Oncology | 10.1007/s00432-020-03258-y |
| **96** | M. Sumazaki, H. Shimada, M. Ito, F. Shiratori, E. Kobayashi, Y. Yoshida, A. Adachi, T. Matsutani, Y. Iwadate, S. Mine, T. Machida, I. Kamitsukasa, M. Mori, K. Sugimoto, A. Uzawa, S. Kuwabara, Y. Kobayashi, M. Ohno, E. Nishi, Y. Maezawa, M. Takemoto, K. Yokote, H. Takizawa, K. Kashiwado, H. Shin, T. Kishimoto, K. Matsushita, S. Kobayashi, R. Nakamura, N. Shinmen, H. Kuroda, X. M. Zhang, H. Wang, K. Goto and T. Hiwasa | 2020 | Serum anti-LRPAP1 is a common biomarker for digestive organ cancers and atherosclerotic diseases | Cancer Science | 10.1111/cas.14652 |
| **97** | D. S. Sun, Q. H. Li, D. D. Ding, X. C. Li, M. Xie, Y. J. Xu and X. S. Liu | 2018 | Role of Kruppel-like factor 4 in cigarette smoke-induced pulmonary vascular remodeling | American Journal of Translational Research |  |
| **98** | X. Q. Sun, R. C. Elston, J. S. Barnholtz-Sloan, G. W. Falk, W. M. Grady, A. Faulx, S. K. Mittal, M. Canto, N. J. Shaheen, J. S. Wang, P. G. Iyer, J. A. Abrams, Y. D. Tian, J. E. Willis, K. Guda, S. D. Markowitz, A. Chandar, J. M. Warfe, W. Brock and A. Chak | 2016 | Predicting Barrett's Esophagus in Families: An Esophagus Translational Research Network (BETRNet) Model Fitting Clinical Data to a Familial Paradigm | Cancer Epidemiology Biomarkers & Prevention | 10.1158/1055-9965.Epi-15-0832 |
| **99** | X. W. Sun, A. L. Xue, T. Qi, D. Chen, D. D. Shi, Y. Wu, Z. L. Zheng, J. Zeng and J. Yang | 2021 | Tumor Mutational Burden Is Polygenic and Genetically Associated with Complex Traits and Diseases | Cancer Research | 10.1158/0008-5472.Can-20-3459 |
| **100** | Y. Tan, X. Lu, Z. Z. Cheng, G. P. Pan, S. J. Liu, P. Apiziaji, H. F. Wang, J. R. Zhang and Y. Abulimiti | 2020 | miR-148a Regulates the Stem Cell-Like Side Populations Distribution by Affecting the Expression of ACVR1 in Esophageal Squamous Cell Carcinoma | Oncotargets and Therapy | 10.2147/ott.S248925 |
| **101** | W. F. Tang, J. Liu, Z. H. Zhong, H. Qiu and M. Q. Kang | 2019 | Association of metabolism-related genes polymorphisms with adenocarcinoma of the oesophagogastric junction: Evidence from 2261 subjects | Journal of Cellular Biochemistry | 10.1002/jcb.29167 |
| **102** | A. P. Thrift, H. A. Risch, L. Onstad, N. J. Shaheen, A. G. Casson, L. Bernstein, D. A. Corley, D. M. Levine, W. H. Chow, B. J. Reid, Y. Romero, L. J. Hardie, G. Liu, A. H. Wu, N. C. Bird, M. D. Gammon, W. M. Ye, D. C. Whiteman and T. L. Vaughan | 2014 | Risk of Esophageal Adenocarcinoma Decreases With Height, Based on Consortium Analysis and Confirmed by Mendelian Randomization | Clinical Gastroenterology and Hepatology | 10.1016/j.cgh.2014.01.039 |
| **103** | J. Tuzil, B. F. Pilnackova, T. Watt, J. Jiskra, M. Koudelkova, E. Novotna, K. Tuzilova, T. Dolezal and J. Bartakova | 2023 | The Impact of Subclinical Hypothyroidism on the Quality of Life During Pregnancy: Mapping 5-Level Version of EQ-5D and ThyPRO-39 | Value in Health | 10.1016/j.jval.2023.02.015 |
| **104** | Y. Usui, K. Matsuo, I. Oze, T. Ugai, Y. Koyanagi, Y. Maeda, H. Ito, A. Hishida, K. Takeuchi, T. Tamura, M. Tsukamoto, Y. Kadomatsu, M. Hara, Y. Nishida, I. Shimoshikiryo, T. Takezaki, E. Ozaki, D. Matsui, I. Watanabe, S. Suzuki, M. Watanabe, H. Nakagawa-Senda, H. Mikami, Y. Nakamura, K. Arisawa, H. Uemura, K. Kuriki, N. Takashima, A. Kadota, H. Ikezaki, M. Murata, M. Nakatochi, Y. Momozawa, M. Kubo and K. Wakai | 2021 | Impact of PSCA Polymorphisms on the Risk of Duodenal Ulcer | Journal of Epidemiology | 10.2188/jea.JE20190184 |
| **105** | V. Uttam, M. K. Rana, U. Sharma, K. Singh and A. Jain | 2024 | Circulating long non-coding RNA <i>EWSAT1</i> acts as a liquid biopsy marker for esophageal squamous cell carcinoma: A pilot study | Non-Coding Rna Research | 10.1016/j.ncrna.2023.10.009 |
| **106** | M. Venerito, C. Helmke, D. Jechorek, T. Wex, R. Rosania, K. Antweiler, J. Weigt and P. Malfertheiner | 2016 | Leukotriene receptor expression in esophageal squamous cell cancer and non-transformed esophageal epithelium: a matched case control study | Bmc Gastroenterology | 10.1186/s12876-016-0499-z |
| **107** | K. von Loga, J. Kohlhaussen, L. Burkhardt, R. Simon, S. Steurer, S. Burdak-Rothkamm, F. Jacobsen, G. Sauter and T. Krech | 2015 | <i>FGFR1</i> Amplification Is Often Homogeneous and Strongly Linked to the Squamous Cell Carcinoma Subtype in Esophageal Carcinoma | Plos One | 10.1371/journal.pone.0141867 |
| **108** | X. Y. Wang, P. Gharahkhani, D. M. Levine, R. C. Fitzgerald, I. Gockel, D. A. Corley, H. A. Risch, L. Bernstein, W. H. Chow, L. Onstad, N. J. Shaheen, J. Lagergren, L. J. Hardie, A. H. Wu, P. D. P. Pharoah, G. Liu, L. A. Anderson, P. G. Iyer, M. D. Gammon, C. Caldas, W. M. Ye, H. Barr, P. Moayyedi, R. Harrison, R. G. P. Watson, S. Attwood, L. Chegwidden, S. B. Love, D. MacDonald, J. DeCaestecker, H. Prenen, K. Ott, S. Moebus, M. Venerito, H. K. Lang, R. Mayershofer, M. Knapp, L. Veits, C. Gerges, J. Weismüller, M. Reeh, M. M. Nöthen, J. R. Izbicki, H. Manner, H. Neuhaus, T. Rösch, A. C. Böhmer, A. H. Hölscher, M. Anders, O. Pech, B. Schumacher, C. Schmidt, T. Schmidt, T. Noder, D. Lorenz, M. Vieth, A. May, T. Hess, N. Kreuser, J. Becker, C. Ell, I. Tomlinson, C. Palles, J. A. Jankowski, D. C. Whiteman, S. MacGregor, J. Schumacher, T. L. Vaughan, M. F. Buas and J. Y. Dai | 2022 | eQTL Set-Based Association Analysis Identifies Novel Susceptibility Loci for Barrett Esophagus and Esophageal Adenocarcinoma | Cancer Epidemiology Biomarkers & Prevention | 10.1158/1055-9965.Epi-22-0096 |
| **109** | Y. J. Wang, X. G. Wang, Y. Xiong, C. D. Li, Q. Xu, L. Shen, A. C. Kaushik and D. Q. Wei | 2019 | An Integrated Pan-Cancer Analysis and Structure-Based Virtual Screening of GPR15 | International Journal of Molecular Sciences | 10.3390/ijms20246226 |
| **110** | Z. M. Wang, W. J. Seow, K. Shiraishi, C. A. Hsiung, K. Matsuo, J. Liu, K. X. Chen, T. Yamji, Y. Yang, I. S. Chang, C. Wu, Y. C. Hong, L. Burdett, K. Wyatt, C. C. Chung, S. C. A. Li, M. Yeager, A. Hutchinson, W. Hu, N. Caporaso, M. T. Landi, N. Chatterjee, M. S. Song, J. F. Fraumeni, T. Kohno, J. Yokota, H. Kunitoh, K. Ashikawa, Y. Momozawa, Y. Daigo, T. Mitsudomi, Y. Yatabe, T. Hida, Z. B. Hu, J. C. Dai, H. X. Ma, G. F. Jin, B. Song, Z. H. Wang, S. S. Cheng, Z. H. Yin, X. L. Li, Y. W. Ren, P. Guan, J. Chang, W. Tan, C. J. Chen, G. C. Chang, Y. H. Tsai, W. C. Su, K. Y. Chen, M. S. Huang, Y. M. Chen, H. Zheng, H. X. Li, P. Cui, H. Guo, P. Xu, L. Liu, M. Iwasaki, T. Shimazu, S. Tsugane, J. J. Zhu, G. N. Jiang, K. Fei, J. Y. Park, Y. H. Kim, J. S. Sung, K. H. Park, Y. T. Kim, Y. J. Jung, C. H. Kang, I. K. Park, H. N. Kim, H. S. Jeon, J. E. Choi, Y. Y. Choi, J. H. Kim, I. J. Oh, Y. C. Kim, S. W. Sung, J. S. Kim, H. I. Yoon, S. S. Kweon, M. H. Shin, A. Seow, Y. Chen, W. Y. Lim, J. J. Liu, M. P. Wong, V. H. F. Lee, B. A. Bassig, M. Tucker, S. I. Berndt, W. H. Chow, B. T. Ji, J. W. Wang, J. Xu, A. D. L. Sihoe, J. C. M. Ho, J. K. C. Chan, J. C. Wang, D. R. Lu, X. Y. Zhao, Z. H. Zhao, J. J. Wu, H. Y. Chen, L. Jin, F. S. Wei, G. P. Wu, S. J. An, X. C. Zhang, J. Su, Y. L. Wu, Y. T. Gao, Y. B. Xiang, X. Z. He, J. H. Li, W. Zheng, X. O. Shu, Q. Y. Cai, R. Klein, W. Pao, C. Lawrence, H. D. Hosgood, C. F. Hsiao, L. H. Chien, Y. H. Chen, C. H. Chen, W. C. Wang, C. Y. Chen, C. L. Wang, C. J. Yu, H. L. Chen, Y. C. Su, F. Y. Tsai, Y. S. Chen, Y. J. Li, T. Y. Yang, C. C. Lin, P. C. Yang, T. C. Wu, D. X. Lin, B. S. Zhou, J. M. Yu, H. B. Shen, M. Kubo, S. J. Chanock, N. Rothman and Q. Lan | 2016 | Meta-analysis of genome-wide association studies identifies multiple lung cancer susceptibility loci in never-smoking Asian women | Human Molecular Genetics | 10.1093/hmg/ddv494 |
| **111** | Z. S. Wang, C. W. Li, X. J. Li, J. G. Shi and W. J. Wu | 2023 | Effect of vascular endothelial growth factor rs35569394 in esophageal cancer and response to chemotherapy | Biomolecules and Biomedicine | 10.17305/bjbms.2021.5891 |
| **112** | W. M. Westra, A. M. Rygiel, N. Mostafavi, G. M. J. de Wit, A. L. Roes, L. M. G. Moons, M. P. Peppelenbosch, S. Ouburg, S. A. Morré, M. Jacobs, P. D. Siersema, S. Repping, K. K. Wang and K. K. Krishnadath | 2020 | The Y-chromosome F haplogroup contributes to the development of Barrett's esophagus-associated esophageal adenocarcinoma in a white male population | Diseases of the Esophagus | 10.1093/dote/doaa011 |
| **113** | J. Y. Y. Wong, H. Zhang, C. A. Hsiung, K. Shiraishi, K. Yu, K. Matsuo, M. P. Wong, Y. C. Hong, J. C. Wang, W. J. Seow, Z. M. Wang, M. S. Song, H. N. Kim, I. S. Chang, N. Chatterjee, W. Hu, C. Wu, T. Mitsudomi, W. Zheng, J. H. Kim, A. Seow, N. E. Caporaso, M. H. Shin, L. P. Chung, S. J. An, P. Wang, Y. Yang, H. Zheng, Y. Yatabe, X. C. Zhang, Y. T. Kim, Q. Y. Cai, Z. H. Yin, Y. C. Kim, B. A. Bassig, J. Chang, J. C. N. Ho, B. T. Ji, Y. Daigo, H. Ito, Y. Momozawa, K. Ashikawa, Y. Kamatani, T. Honda, H. D. Hosgood, H. Sakamoto, H. Kunitoh, K. Tsuta, S. I. Watanabe, M. Kubo, Y. Miyagi, H. Nakayama, S. Matsumoto, M. Tsuboi, K. Goto, J. X. Shi, L. Song, X. Hua, A. Takahashi, A. Goto, Y. Minamiya, K. Shimizu, K. Tanaka, F. Wei, F. Matsuda, J. Su, Y. H. Kim, I. J. Oh, F. J. Song, W. C. Su, Y. M. Chen, G. C. Chang, K. Y. Chen, M. S. Huang, L. H. Chien, Y. B. Xiang, J. Y. Park, S. S. Kweon, C. J. Chen, K. M. Lee, B. Blechter, H. X. Li, Y. T. Gao, B. Y. Qian, D. R. Lu, J. J. Liu, H. S. Jeon, C. F. Hsiao, J. S. Sung, Y. H. Tsai, Y. J. Jung, H. Guo, Z. B. Hu, W. C. Wang, C. C. Chung, L. Burdett, M. Yeager, A. Hutchinson, S. Berndt, W. Wu, H. Pang, Y. Q. Li, J. E. Choi, K. H. Park, S. W. Sung, L. Liu, C. H. Kang, M. Zhu, C. H. Chen, T. Y. Yang, J. Xu, P. Guan, W. Tan, C. L. Wang, M. Hsin, K. Y. Sit, Y. Chen, Y. Y. Choi, J. Y. Hung, J. S. Kim, H. Il Yoon, C. C. Lin, I. K. Park, P. Xu, Y. Wang, Q. He, R. P. Perng, C. Y. Chen, R. Vermeulen, J. J. Wu, W. Y. Lim, K. C. Chen, Y. J. Li, J. H. Li, H. Y. Chen, C. J. Yu, L. Jin, T. Y. Chen, S. S. Jiang, J. Liu, T. Yamaji, B. Hicks, K. Wyatt, S. A. Li, J. C. Dai, H. X. Ma, G. F. Jin, B. Song, Z. H. Wang, S. S. Cheng, X. L. Li, Y. W. Ren, P. Cui, M. Iwasaki, T. Shimazu, S. Tsugane, J. J. Zhu, K. Y. Yang, G. N. Jiang, K. Fei, G. P. Wu, H. C. Lin, H. L. Chen, Y. H. Fang, F. Y. Tsai, W. S. Hsieh, J. M. Yu, V. L. Stevens, I. A. Laird-Offringa, C. N. Marconett, L. Rieswijk, A. Chao, P. C. Yang, X. O. Shu, T. Wu, Y. L. Wu, D. X. Lin, K. X. Chen, B. S. Zhou, Y. C. Huang, T. Kohno, H. Shen, S. J. Chanock, N. Rothman and Q. Lan | 2020 | Tuberculosis infection and lung adenocarcinoma: Mendelian randomization and pathway analysis of genome-wide association study data from never-smoking Asian women | Genomics | 10.1016/j.ygeno.2019.07.008 |
| **114** | S. Q. Wu, L. Y. Zhang, J. Q. Deng, B. B. Guo, F. Li, Y. R. Wang, R. Wu, S. H. Zhang, J. C. Lu and Y. F. Zhou | 2020 | A Novel Micropeptide Encoded by Y-Linked LINC00278 Links Cigarette Smoking and AR Signaling in Male Esophageal Squamous Cell Carcinoma | Cancer Research | 10.1158/0008-5472.Can-19-3440 |
| **115** | V. K. Yadav, J. DeGregori and S. De | 2016 | The landscape of somatic mutations in protein coding genes in apparently benign human tissues carries signatures of relaxed purifying selection | Nucleic Acids Research | 10.1093/nar/gkw086 |
| **116** | K. Yamamura, Y. Baba, S. Nakagawa, K. Mima, K. Miyake, K. Nakamura, H. Sawayama, K. Kinoshita, T. Ishimoto, M. Iwatsuki, Y. Sakamoto, Y. Yamashita, N. Yoshida, M. Watanabe and H. Baba | 2016 | Human Microbiome <i>Fusobacterium Nucleatum</i> in Esophageal Cancer Tissue Is Associated with Prognosis | Clinical Cancer Research | 10.1158/1078-0432.Ccr-16-1786 |
| **117** | K. Q. Yang, S. Y. Li, Y. C. Ding, X. D. Meng, C. H. Zhang and X. J. Sun | 2024 | Effect of smoking-related features and 731 immune cell phenotypes on esophageal cancer: a two-sample and mediated Mendelian randomized study | Frontiers in Immunology | 10.3389/fimmu.2024.1336817 |
| **118** | S. Yang, J. Lee, I. J. Choi, Y. W. Kim, K. W. Ryu, J. Sung and J. Kim | 2017 | Effects of alcohol consumption, <i>ALDH2</i> rs671 polymorphism, and <i>Helicobacter pylori</i> infection on the gastric cancer risk in a Korean population | Oncotarget | 10.18632/oncotarget.14250 |
| **119** | S. S. Yoo, H. G. Kang, J. E. Choi, S. K. Do, W. K. Lee, S. H. Choi, S. Y. Lee, S. Y. Lee, J. Lee, S. I. Cha, C. H. Kim, Y. Seok, E. Lee, M. S. Kim, J. M. Lee, H. J. Cho, I. J. Oh, Y. C. Kim, S. Cho, S. Jheon, C. Y. Jung, M. H. Kim, M. K. Lee and J. Y. Park | 2017 | Effects of polymorphism identified in genome-wide association studies of never-smoking females on the prognosis of non-small cell lung cancer | Cancer Genetics | 10.1016/j.cancergen.2017.03.003 |
| **120** | G. Q. Yu, D. J. Miller, C. T. Wu, E. P. Hoffman, C. Y. Liu, D. M. Herrington and Y. Wang | 2019 | Asymmetric independence modeling identifies novel gene-environment interactions | Scientific Reports | 10.1038/s41598-019-38983-z |
| **121** | S. Yuan, J. Chen, X. X. Ruan, Y. H. Sun, K. Zhang, X. Y. Wang, X. Li, D. Gill, S. Burgess, E. Giovannucci and S. C. Larsson | 2023 | Smoking, alcohol consumption, and 24 gastrointestinal diseases: Mendelian randomization analysis | Elife | 10.7554/eLife.84051 |
| **122** | Y. X. Yun, L. P. Dai, P. Wang, K. J. Wang, J. Y. Zhang and W. Xie | 2015 | Association of Polymorphisms in X-Ray Repair Cross Complementing 1 Gene and Risk of Esophageal Squamous Cell Carcinoma in a Chinese Population | Biomed Research International | 10.1155/2015/509215 |
| **123** | B. S. Zhang, X. M. Zhang, M. Ito, S. Yajima, K. Yoshida, M. Ohno, E. Nishi, H. Wang, S. Y. Li, M. Kubota, Y. Yoshida, T. Matsutani, S. Mine, T. Machida, M. Takemoto, H. Yamagata, A. Hayashi, K. Yokote, Y. Kobayashi, H. Takizawa, H. Kuroda, H. Shimada, Y. Iwadate and T. Hiwasa | 2024 | JMJD6 Autoantibodies as a Potential Biomarker for Inflammation-Related Diseases | International Journal of Molecular Sciences | 10.3390/ijms25094935 |
| **124** | X. N. Zhang, X. R. Yang, T. C. Zhang, X. L. Yin, J. Y. Man and M. Lu | 2023 | Association of educational attainment with esophageal cancer, Barrett's esophagus, and gastroesophageal reflux disease, and the mediating role of modifiable risk factors: A Mendelian randomization study | Frontiers in Public Health | 10.3389/fpubh.2023.1022367 |
| **125** | H. Q. Zhou, Y. Hu, R. Z. Luo, Y. Y. Zhao, H. Pan, L. Y. Ji, T. Zhou, L. J. Zhang, H. Long, J. H. Fu, Z. S. Wen, S. Y. Wang, X. Wang, P. Lin, H. X. Yang, J. Y. Wang, M. M. Song, X. Yi, L. Yang, X. F. Xia, Y. F. Guan, W. F. Fang, Y. P. Yang, S. D. Hong, Y. Huang, P. S. Li, Y. X. Zhang and N. N. Zhou | 2021 | Multi-region exome sequencing reveals the intratumoral heterogeneity of surgically resected small cell lung cancer | Nature Communications | 10.1038/s41467-021-25787-x |
| **126** | J. F. Zhu, C. Liu, X. Teng, J. Yin, L. Zheng, L. M. Wang, W. F. Tang, H. Y. Gu, B. Gu and L. Chen | 2016 | Association of the interleukin-18 receptor 1 and interleukin-18 receptor accessory protein polymorphisms with the risk of esophageal cancer | Biomedical Reports | 10.3892/br.2015.552 |
