# Supplementary material for: Gastroesophageal disease risk and inhalational exposure a systematic review and meta-analysis
Source: Sci Rep. 2025 Jul 2;15:22581. doi: 10.1038/s41598-025-06620-7 (PMC12218983; doi:10.1038/s41598-025-06620-7)
Supplement: Supplementary file 5 — Supplementary Material 5. [file 41598_2025_6620_MOESM5_ESM.docx]

| **Supplemental Table 5. Case Reports/Series Excluded (N = 51)** | | | | | | |
| --- | --- | --- | --- | --- | --- | --- |
|  | **Author(s)** | **Year** | **Title** | **Journal** | | **DOI** |
|  | **PubMed (N = 27)** | | | | | |
| **1** | Andresen, N. S., Lee, D. J., Kowalski, C. E. and Bayon, R. | 2018 | Fall With e-Cigarette in Mouth Resulting in Pharyngeal and Esophageal Burns | | JAMA Otolaryngol Head Neck Surg | 10.1001/jamaoto.2017.3265 |
| **2** | Bozzella, M. J., Magyar, M., DeBiasi, R. L. and Ferrer, K. | 2020 | Epiglottitis Associated With Intermittent E-cigarette Use: The Vagaries of Vaping Toxicity | | Pediatrics | 10.1542/peds.2019-2399 |
| **3** | Cammarano, C. A. and Villaluz, J. E. | 2021 | A Reason to Rethink Fasting Guidelines? Marijuana-Induced Gastroparesis and the Implications for Aspiration Risk in the Nil Per Os (NPO) Patient: A Case Report | | Am J Case Rep | 10.12659/ajcr.934187 |
| **4** | Changela, K. and Reddy, M. | 2017 | Smoker's melanosis: Isolated pigmented lesion in the laryngopharynx and esophagus | | Turk J Gastroenterol | 10.5152/tjg.2017.17186 |
| **5** | de-Tomás, J. and Monturiol, J. M. | 2016 | Is there any relationship between drug addiction and the development of a signet ring cell carcinoma of the stomach? | | Rev Esp Enferm Dig | 10.17235/reed.2016.4070/2015 |
| **6** | DelRosso, L. M. | 2015 | A 3-month-old infant with recurrent apparent life-threatening events in a car seat | | Chest | 10.1378/chest.14-1595 |
| **7** | Duvvuri, P. D., Bhardwaj, C. and Wattanakit, K. | 2021 | A 50-Year-Old Woman With Shortness of Breath | | Chest | 10.1016/j.chest.2020.10.098 |
| **8** | Esteban Ronda, V., Franco Serrano, J. and Briones Urtiaga, M. L. | 2016 | Pulmonary Strongyloides stercoralis infection | | Arch Bronconeumol | 10.1016/j.arbres.2016.01.010 |
| **9** | Gao, S., Li, J., Feng, X., Shi, S. and He, J. | 2016 | Characteristics and Surgical Outcomes for Primary Malignant Melanoma of the Esophagus | | Sci Rep | 10.1038/srep23804 |
| **10** | Gasmelseed, N., Abudris, D., Elhaj, A., Eltayeb, E. A., Elmadani, A., Elhassan, M. M., Mohammed, K., Elgaili, E. M., Elbalal, M., Schuz, J. and Leon, M. E. | 2015 | Patterns of Esophageal Cancer in the National Cancer Institute at the University of Gezira, in Gezira State, Sudan, in 1999-2012 | | Asian Pac J Cancer Prev | 10.7314/apjcp.2015.16.15.6481 |
| **11** | Herout, K. T., Durant, E. J. and Fong, J. | 2021 | Dysphagia as the Predominant Symptom in Posterior Circulation Stroke: A Case Report | | Am J Case Rep | 10.12659/ajcr.930502 |
| **12** | Johnson, J. A. and Landreneau, R. J. | 1991 | Esophageal obstruction and mediastinitis: a hard pill to swallow for drug smugglers | | Am Surg |  |
| **13** | Ko, E. H. and Rubin, A. D. | 2014 | Dysphagia due to inclusion body myositis: case presentation and review of the literature | | Ann Otol Rhinol Laryngol | 10.1177/0003489414525588 |
| **14** | Leceaga Gaztambide, E., Aguilar Cayuelas, A. and Fuster Martí, D. | 2018 | Empyema necessitatis 17 years after a pneumonectomy | | Med Clin (Barc) | 10.1016/j.medcli.2017.11.006 |
| **15** | Luquiens, A., Lourenco, N., Benyamina, A. and Aubin, H. J. | 2015 | Self-medication of achalasia with cannabis, complicated by a cannabis use disorder | | World J Gastroenterol | 10.3748/wjg.v21.i20.6381 |
| **16** | Nana Sede Mbakop, R., Kesiena, O., Greene, T. E. and Amakye, D. | 2023 | Cannabinoid Hyperemesis Syndrome in a 23-Year-Old Woman with Uncontrolled Type 1 Diabetes Mellitus | | Am J Case Rep | 10.12659/ajcr.938418 |
| **17** | Norii, T. and Plate, A. | 2017 | Electronic Cigarette Explosion Resulting in a C1 and C2 Fracture: A Case Report | | J Emerg Med | 10.1016/j.jemermed.2016.08.010 |
| **18** | Pasricha, T. S. and Kochar, B. | 2021 | Vaping-associated esophagitis | | BMC Gastroenterol | 10.1186/s12876-021-01695-8 |
| **19** | Rao, S. J., Kirse, D. J. and Shetty, A. K. | 2021 | Cannabis induced thermal epiglottitis in a pediatric patient | | Am J Emerg Med | 10.1016/j.ajem.2021.05.024 |
| **20** | Roa Colomo, A., García Robles, A. and Ruiz Escolano, E. | 2019 | Black esophagus, is it as bad as it seems? | | Gastroenterol Hepatol | 10.1016/j.gastrohep.2019.06.004 |
| **21** | Rodríguez-Lago, I., Calderón, Á, Cazallas, J., Camino, M. E., Barredo, I. and Cabriada, J. L. | 2017 | First case report of oesophageal actinomycosis in a patient with active eosinophilic oesophagitis | | Gastroenterol Hepatol | 10.1016/j.gastrohep.2016.04.012 |
| **22** | Ruiz-Tovar, J., Díaz, G., Alias, D., Jiménez-Fuertes, M. and Durán, M. | 2016 | Hemoperitoneum secondary to an spontaneous rupture of the spleen mimmicking a duodenal perforated ulcera: A case report | | Rev Esp Enferm Dig |  |
| **23** | Sabău, D., Dumitra, A., Sabău, A., Maniu, D., Mitachescu, A., Ilie, S., Hulpus, R. and Smarandache, G. | 2015 | Esotracheal Fistula in Esophageal Stenoses of Malignant Origin - Case Report | | Chirurgia (Bucur) |  |
| **24** | Sevinc, M. M., Kinaci, E., Bayrak, S., Yardimci, A. H., Cakar, E. and Bektaş, H. | 2015 | Extraordinary cause of acute gastric dilatation and hepatic portal venous gas: Chronic use of synthetic cannabinoid | | World J Gastroenterol | 10.3748/wjg.v21.i37.10704 |
| **25** | Singhi, A. D., Seethala, R. R., Nason, K., Foxwell, T. J., Roche, R. L., McGrath, K. M., Levy, R. M., Luketich, J. D. and Davison, J. M. | 2015 | Undifferentiated carcinoma of the esophagus: a clinicopathological study of 16 cases | | Hum Pathol | 10.1016/j.humpath.2014.11.021 |
| **26** | Watanabe, K. | 2017 | Black hairy tongue | | Acta Otorrinolaringol Esp (Engl Ed) | 10.1016/j.otorri.2016.10.002 |
| **27** | Yao, B., Guan, S., Huang, X., Su, P., Song, Q. and Cheng, Y. | 2015 | A collision tumor of esophagus | | Int J Clin Exp Pathol |  |
|  | **WoS (N = 24)** | | | | | |
| **1** | J. Al-Kassmy, M. Alsalmi, W. J. Kang and P. Huot | 2024 | Anticonvulsant Agents for Treatment of Restless Legs Syndrome | | Neurologist | 10.1097/nrl.0000000000000552 |
| **2** | M. M. Alhalabi, S. A. Alsayd and M. E. Albattah | 2022 | Advanced diffuse gastric adenocarcinoma in young Syrian woman. A case report | | Annals of Medicine and Surgery | 10.1016/j.amsu.2022.103728 |
| **3** | K. Arakawa, K. Hata, Y. Yamamoto, T. Nishikawa, T. Tanaka, T. Kiyomatsu, K. Kawai, H. Nozawa, M. Yoshida, H. Fukuhara, M. Fujishiro, T. Morikawa, T. Yamasoba, K. Koike, M. Fukayama and T. Watanabe | 2018 | Nine primary malignant neoplasms involving the esophagus, stomach, colon, rectum, prostate, and external ear canal-without microsatellite instability: a case report | | BMC Cancer | 10.1186/s12885-017-3973-2 |
| **4** | M. F. Ashraf, S. Richter, S. H. Arker and N. Parsa | 2021 | A Rare Case of Esophageal Leukoplakia: A Potential Precursor to Esophageal Malignancy | | Cureus Journal of Medical Science | 10.7759/cureus.17205 |
| **5** | C. G. Birngruber, F. Veit, J. Lang and M. A. Verhoff | 2017 | Inhaled cyanide poisoning as a vital sign in a room fire victim | | Forensic Science International | 10.1016/j.forsciint.2017.10.037 |
| **6** | O. Bruserud, D. E. Costea, S. Laakso, B. Z. Garty, E. Mathisen, A. Mäkitie, O. Mäkitie and E. S. Husebye | 2018 | Oral Tongue Malignancies in Autoimmune Polyendocrine Syndrome Type 1 | | Frontiers in Endocrinology | 10.3389/fendo.2018.00463 |
| **7** | G. Caruana, R. Cachia, S. Micallef, M. Sammut and J. Psaila | 2024 | Metastatic mediastinal hepatoid adenocarcinoma | | Bmj Case Reports | 10.1136/bcr-2022-253747 |
| **8** | B. J. Choi, S. Lee, I. J. Lee, S. W. Park and S. Lee | 2020 | Gastric and rectal cancers in workers exposed to asbestos: a case series | | Annals of Occupational and Environmental Medicine | 10.35371/aoem.2020.32.e4 |
| **9** | I. Dina, O. Ginghina, C. D. Toderescu, C. Balalau, B. Galateanu, C. Negrei and C. Iacobescu | 2017 | Zenker's diverticulum and squamous esophageal cancer: a case report | | Journal of Mind and Medical Sciences | 10.22543/7674.42.P193197 |
| **10** | A. Gonçalves, D. Simas, P. Gomes, S. Barbeiro, I. Cotrim and H. Vasconcelos | 2024 | Unveiling the complex nexus: dermatomyositis and esophageal adenocarcinoma-a case report | | Annals of Esophagus | 10.21037/aoe-23-23 |
| **11** | J. Z. Hashmi, M. Hiraj, F. Saleem, U. Malik and I. K. Mazari | 2022 | Double Peptic Ulcer Perforation due to Cumulative Effects of Post-surgery Stress and NSAIDs: A Rare Event in Surgical Practice | | Jcpsp-Journal of the College of Physicians and Surgeons Pakistan | 10.29271/jcpsp.2022.JCPSPCR.CR21 |
| **12** | M. Javeed, H. Gruhonjic, T. Kirkman, C. Pitarys and R. Akel | 2022 | A Unique Case of a Right Atrial Myxoma Infected With Escherichia coli | | Cureus Journal of Medical Science | 10.7759/cureus.25394 |
| **13** | G. Kanagalingam, Y. Achuo-Egbe, M. F. Ahmed, O. Oluaderounmu and J. Harley | 2022 | A Rare Case of Esophageal Leukoplakia in Achalasia | | Cureus Journal of Medical Science | 10.7759/cureus.23735 |
| **14** | Q. Liu, Y. Yang, X. S. Fan, X. Y. Xin, Q. Y. Pan, Y. H. Zhang, B. R. Liu and J. Wei | 2021 | Heterogeneity response to afatinib in gastric cancer patient with uncommon epidermal growth factor receptor (EGFR) mutations: a case report | | Annals of Translational Medicine | 10.21037/atm-20-7312 |
| **15** | S. H. Liu, J. Y. Qian, Q. R. Li, D. H. Liu, B. Zhang and X. X. Chen | 2024 | Case Report: foetal gastroschisis with ideal pregnancy outcomes under multidisciplinary treatment management | | Frontiers in Pediatrics | 10.3389/fped.2024.1358856 |
| **16** | T. S. Pasricha and B. Kochar | 2021 | Vaping-associated esophagitis | | Bmc Gastroenterology | 10.1186/s12876-021-01695-8 |
| **17** | B. L. Phelps, Y. M. Tiley, J. L. Skrove, A. C. Berry and K. Mohan | 2019 | Acute Dysphagia Caused by sarcomatoid Squamous Cell Carcinoma of the Esophagus | | Cureus Journal of Medical Science | 10.7759/cureus.4129 |
| **18** | M. S. Seyyedi, V. Zangouri, Z. Dehghani, A. Dehghanian and M. G. Jahromi | 2024 | A rare occurrence of breast, thyroid, and stomach tumors in a single patient: A case report | | International Journal of Surgery Case Reports | 10.1016/j.ijscr.2024.109670 |
| **19** | B. S. Shiflett, L. S. Ekanayake, A. L. Rodriguez, I. Ikramuddin and C. Myers | 2020 | Esophageal Adenocarcinoma in the Proximal Esophageal Segment: A Unique Presentation in a Male With Alcohol Abuse | | Cureus Journal of Medical Science | 10.7759/cureus.8863 |
| **20** | F. Shweikeh, G. Hong, J. Walter, M. Hoscheit, A. Lembo, M. Mouchli and J. Lane | 2024 | SMARCA4-Deficient Undifferentiated Esophageal Carcinoma: A Clinical Case Series and Literature Review | | Journal of Gastrointestinal Cancer | 10.1007/s12029-024-01060-4 |
| **21** | C. G. Solomon and R. Fass | 2022 | Gastroesophageal Reflux Disease | | New England Journal of Medicine | 10.1056/NEJMcp2114026 |
| **22** | X. Sun, L. F. Wang, Y. Feng, H. Xie, X. Y. Zheng, A. He, M. R. Karim, Z. Y. Lv and Z. D. Wu | 2016 | A case report: A rare case of infant gastrointestinal canthariasis caused by larvae of <i>Lasioderma</i> <i>serricorne</i> (<i>Fabricius</i>, 1792) (Coleoptera: Anobiidae) | | Infectious Diseases of Poverty | 10.1186/s40249-016-0129-6 |
| **23** | M. Yousef, H. Chela, H. Ertugrul, A. Albarrak, O. Basar, S. Pasha, Y. Mousa, A. Al Juboori, S. Frazier, V. Tahan and E. Daglilar | 2023 | Lymphocytic Esophagitis: A Case Series of Esophageal Disease with Increasing Frequency | | Recent Advances in Inflammation & Allergy Drug Discovery | 10.2174/2772270817666230130093341 |
| **24** | Q. Z. Zhou, Y. Q. Wei, H. H. Zhai, S. G. Li, R. Xu and P. Li | 2021 | Comorbid early esophageal cancer and <i>Gongylonema pulchrum</i> infection: a case report | | Bmc Gastroenterology | 10.1186/s12876-021-01873-8 |
