# Supplementary material for: Gastroesophageal disease risk and inhalational exposure a systematic review and meta-analysis
Source: Sci Rep. 2025 Jul 2;15:22581. doi: 10.1038/s41598-025-06620-7 (PMC12218983; doi:10.1038/s41598-025-06620-7)
Supplement: Supplementary file 6 — Supplementary Material 6. [file 41598_2025_6620_MOESM6_ESM.docx]

| **Supplemental Table 6. Pediatric Studies Excluded (N = 14)** | | | | | |
| --- | --- | --- | --- | --- | --- |
|  | **Author(s)** | **Year** | **Title** | **Journal** | **DOI** |
|  | **PubMed (N = 9)** | | | | |
| **1** | Budukh, A., Shah, S., Kulkarni, S., Pimple, S., Patil, S., Chaukar, D. and Pramesh, C. S. | 2022 | Tobacco and cancer awareness program among school children in rural areas of Ratnagiri district of Maharashtra state in India | Indian J Cancer | 10.4103/ijc.IJC_629_19 |
| **2** | Djeddi, D., Stephan-Blanchard, E., Léké, A., Ammari, M., Delanaud, S., Lemaire-Hurtel, A. S., Bach, V. and Telliez, F. | 2018 | Effects of Smoking Exposure in Infants on Gastroesophageal Reflux as a Function of the Sleep-Wakefulness State | J Pediatr | 10.1016/j.jpeds.2018.05.057 |
| **3** | Pesek, R. D., Rettiganti, M., O'Brien, E., Beckwith, S., Daniel, C., Luo, C., Scurlock, A. M., Chandler, P., Levy, R. A., Perry, T. T., Kennedy, J. L., Chervinskiy, S., Vonlanthen, M., Casteel, H., Fiedorek, S. C., Gibbons, T. and Jones, S. M. | 2017 | Effects of allergen sensitization on response to therapy in children with eosinophilic esophagitis | Ann Allergy Asthma Immunol | 10.1016/j.anai.2017.06.006 |
| **4** | Slae, M., Persad, R., Leung, A. J., Gabr, R., Brocks, D. and Huynh, H. Q. | 2015 | Role of Environmental Factors in the Development of Pediatric Eosinophilic Esophagitis | Dig Dis Sci | 10.1007/s10620-015-3740-7 |
| **5** | Song, M., Choi, J. Y., Yang, J. J., Sung, H., Lee, Y., Lee, H. W., Kong, S. H., Lee, H. J., Kim, H. H., Kim, S. G., Yang, H. K. and Kang, D. | 2015 | Obesity at adolescence and gastric cancer risk | Cancer Causes Control | 10.1007/s10552-014-0506-z |
| **6** | Suwanwongse, K. and Shabarek, N. | 2020 | Epidemiology, clinical features, and outcomes of hospitalized infants with COVID-19 in the Bronx, New York | Arch Pediatr | 10.1016/j.arcped.2020.07.009 |
| **7** | Svenningsson, A., Svensson, T., Akre, O. and Nordenskjöld, A. | 2014 | Maternal and pregnancy characteristics and risk of infantile hypertrophic pyloric stenosis | J Pediatr Surg | 10.1016/j.jpedsurg.2014.01.053 |
| **8** | Zhou, Y., Yang, S., Lin, Q., He, Q. and Cui, Y. | 2023 | Frequent presence of major dust mite allergens in human digestive tissues of children with gastritis | Allergy | 10.1111/all.15587 |
| **9** | Zwink, N., Choinitzki, V., Baudisch, F., Hölscher, A., Boemers, T. M., Turial, S., Kurz, R., Heydweiller, A., Keppler, K., Müller, A., Bagci, S., Pauly, M., Brokmeier, U., Leutner, A., Degenhardt, P., Schmiedeke, E., Märzheuser, S., Grasshoff-Derr, S., Holland-Cunz, S., Palta, M., Schäfer, M., Ure, B. M., Lacher, M., Nöthen, M. M., Schumacher, J., Jenetzky, E. and Reutter, H. | 2016 | Comparison of environmental risk factors for esophageal atresia, anorectal malformations, and the combined phenotype in 263 German families | Dis Esophagus | 10.1111/dote.12431 |
| **Web of Science (N = 5)** | | | | | |
| **1** | M. K. Almutairi, A. K. Alkharji, W. A. Alhelal, S. A. Alqahtani and M. M. Altalha | 2019 | ETIOLOGY OF APPARENT LIFE-THREATENING EVENT IN INFANTS AT NATIONAL GUARD HEALTH AFFAIRS IN RIYADH, SAUDI ARABIA | Indo American Journal of Pharmaceutical Sciences | 10.5281/zenodo.2648626 |
| **2** | D. C. Baird, D. J. Harker and A. S. Karmes | 2015 | Diagnosis and Treatment of Gastroesophageal Reflux in Infants and Children | American Family Physician |  |
| **3** | J. Berro, M. Akel, S. Hallit and S. Obeid | 2021 | Relationships between inappropriate eating habits and problematic alcohol use, cigarette and waterpipe dependence among male adolescents in Lebanon | Bmc Public Health | 10.1186/s12889-021-10184-2 |
| **4** | A. Budukh, S. H. Shah, S. Kulkarni, S. Pimple, S. Patil, D. Chaukar and C. Pramesh | 2022 | Tobacco and cancer awareness program among school children in rural areas of Ratnagiri district of Maharashtra state in India | Indian Journal of Cancer | 10.4103/ijc.IJC_629_19 |
| **5** | S. K. Sah, N. Neupane, A. Pradhan, S. Shah and A. Sharma | 2020 | Prevalence of glue-sniffing among street children | Nursing Open | 10.1002/nop2.380 |
