# Supplementary material for: Gastroesophageal disease risk and inhalational exposure a systematic review and meta-analysis
Source: Sci Rep. 2025 Jul 2;15:22581. doi: 10.1038/s41598-025-06620-7 (PMC12218983; doi:10.1038/s41598-025-06620-7)
Supplement: Supplementary file 7 — Supplementary Material 7. [file 41598_2025_6620_MOESM7_ESM.docx]

| **Supplemental Table 7A. Risk of Bias Assessment for Cohort Studies (N = 39)** | | | | | | | | | | | |
| --- | --- | --- | --- | --- | --- | --- | --- | --- | --- | --- | --- |
|  | **Study** | **Selection** | | | | **Comparability** | **Outcome** | | | **Total Score** | **Paper Quality** |
|  |  | **SL1** | **SL2** | **SL3** | **SL4** | **CP** | **OC1** | **OC2** | **OC3** |  |  |
|  | **PubMed (N = 20)** | | | | | | | | | | |
| 1 | **Almadi, 2014*** | - | ★ | - | ★ | - | - | N/A | ★ | 3/8 | Poor |
| 2 | **Begovic, 2015** | ★ | ★ | ★ | - | ★ | ★ | N/A | ★ | 6/8 | Good |
| 3 | **Crews, 2016** | ★ | ★ | ★ | - | ★★ | ★ | N/A | ★ | 7/8 | Good |
| 4 | **Dore, 2016*** | - | ★ | ★ | ★ | ★ | ★ | N/A | ★ | 6/9 | Good |
| 5 | **Etemadi, 2017** | ★ | ★ | ★ | - | ★★ | - | ★ | ★ | 7/9 | Good |
| 6 | **Jayalekshmi, 2015** | ★ | ★ | ★ | ★ | ★ | ★ | ★ | ★ | 8/9 | Good |
| 7 | **Jayalekshmi, 2021** | ★ | ★ | ★ | ★ | ★★ | ★ | ★ | ★ | 9/9 | Good |
| 8 | **Kim, 2019*** | ★ | ★ | - | ★ | ★★ | ★ | ★ | ★ | 8/9 | Good |
| 9 | **Kim, 2018** | - | ★ | - | - | ★★ | ★ | N/A | ★ | 5/8 | Poor |
| 10 | **Le, 2022** | ★ | ★ | ★ | ★ | ★ | ★ | ★ | ★ | 9/9 | Good |
| 11 | **Levenstein, 2017** | ★ | ★ | ★ | ★ | ★★ | ★ | ★ | - | 8/9 | Good |
| 12 | **Minami, 2018** | ★ | ★ | - | ★ | ★★ | ★ | ★ | ★ | 8/9 | Good |
| 13 | **Moses, 2017** | ★ | ★ | ★ | - | - | ★ | N/A | ★ | 5/8 | Poor |
| 14 | **Park, Kim, Jung, 2022** | ★ | ★ | - | ★ | ★★ | ★ | ★ | ★ | 8/9 | Good |
| 15 | **Park, Kim, Oh, 2022** | ★ | ★ | - | ★ | ★★ | ★ | ★ | ★ | 8/9 | Good |
| 16 | **Spreafico, 2017** | ★ | ★ | - | - | ★★ | ★ | ★ | ★ | 7/9 | Fair |
| 17 | **Sun, 2023*** | ★ | ★ | ★ | ★ | ★★ | ★ | ★ | ★ | 9/9 | Good |
| 18 | **Wang, 2021*** | ★ | ★ | ★ | - | ★★ | ★ | ★ | ★ | 8/9 | Good |
| 19 | **Yates, 2014** | ★ | ★ | ★ | ★ | ★★ | ★ | ★ | ★ | 9/9 | Good |
| 20 | **Zacharakis, 2023** | - | ★ | - | - | - | ★ | ★ | ★ | 4/9 | Poor |
|  | **Web of Science (N = 19)** | | | | | | | | | | |
| 1 | **Alcala, 2023** | ★ | ★ | ★ | ★ | ★★ | ★ | ★ | ★ | 9/9 | Good |
| 2 | **Arroyo-Martinez, 2016** | ★ | ★ | ★ | ★ | ★ | ★ | ★ | ★ | 8/9 | Good |
| 3 | **Chen, Li, 2023** | ★ | ★ | ★ | ★ | ★★ | ★ | ★ | ★ | 9/9 | Good |
| 4 | **Chen, Peto, 2015** | ★ | ★ | ★ | - | ★★ | ★ | ★ | ★ | 8/9 | Good |
| 5 | **Dighe, 2021** | ★ | ★ | ★ | ★ | ★★ | ★ | ★ | ★ | 9/9 | Good |
| 6 | **Etemadi, 2024** | ★ | ★ | ★ | - | ★★ | ★ | ★ | ★ | 8/9 | Good |
| 7 | **Kang, 2016** | ★ | ★ | ★ | ★ | ★★ | ★ | ★ | ★ | 9/9 | Good |
| 8 | **Kim, Gong, 2014** | ★ | ★ | ★ | ★ | ★★ | ★ | ★ | - | 8/9 | Good |
| 9 | **Kim, Lee, 2014** | ★ | ★ | ★ | - | ★★ | ★ | ★ | ★ | 9/9 | Good |
| 10 | **Kumar, 2020** | ★ | ★ | ★ | ★ | ★★ | ★ | ★ | ★ | 9/9 | Good |
| 11 | **Kunzmann, 2018** | ★ | ★ | ★ | ★ | ★★ | ★ | ★ | ★ | 9/9 | Good |
| 12 | **Laaksonen, 2023** | ★ | ★ | ★ | ★ | ★★ | ★ | ★ | ★ | 9/9 | Good |
| 13 | **Li, Xu, 2020** | ★ | ★ | ★ | ★ | ★★ | ★ | ★ | ★ | 9/9 | Good |
| 14 | **Li, He, 2024** | ★ | ★ | ★ | - | ★★ | ★ | ★ | ★ | 8/9 | Good |
| 15 | **Lim, 2021** | ★ | ★ | ★ | ★ | ★★ | ★ | ★ | ★ | 9/9 | Good |
| 16 | **Rabiee, 2016** | ★ | ★ | ★ | - | ★★ | ★ | - | - | 6/9 | Poor |
| 17 | **Sheikh, 2019** | ★ | ★ | ★ | - | ★★ | ★ | ★ | ★ | 8/9 | Good |
| 18 | **Soroush, 2023** | ★ | ★ | ★ | ★ | ★★ | ★ | ★ | ★ | 9/9 | Good |
| 19 | **Wang, Kendall, 2022** | ★ | ★ | ★ | ★ | ★★ | ★ | ★ | ★ | 9/9 | Good |
| **Abbreviations:** **SL** Selection; **CP** Comparability; **OC** Outcome. **N/A*** Not applicable. **Max** Maximum  **SL1**: Representativeness of the exposed cohort (truly representative of the average ★, somewhat representative ★, selected group of users, no description of derivation of cohort) Maximum: ★  **SL2**: Selection of the non-exposed cohort (drawn from the same community ★, drawn from different source, no description of derivation of cohort) Max: ★  **SL3**: Ascertainment of exposure (secure record ★, structured interview ★, written self-report, no description) Maximum: ★  **SL4**: Demonstration that outcome of interest was not present at start of study (Yes ★, No) Maximum: ★  **CP**: Comparability of cohorts on the basis of the design or analysis (study controls for basic confounders: age, sex, marital status, etc. ★; controls for other study-specific confounders ★) Maximum: ★★  **OC1**: Assessment of outcome (Independent blind assessment ★, record linkage ★, self-report, no description) Maximum: ★  **OC2**: Adequate follow up length (Yes ★, No) Maximum: ★  **OC3**: Adequacy of follow up (All subjects accounted for ★, Subjects lost <20% or description of those lost suggested no different from those followed ★, Follow up rate <80% and no description of those lost) Maximum: ★  Converting NOS to AHRQ Standards (Good, fair, and poor quality)  Good: 3-4★ in selection AND 1-2★ in comparability AND 2-3★ in outcome;  Fair: 2★ in selection AND 1-2 in comparability AND 2-3 outcome; Poor: 0-1★ in selection OR 0★ in comparability OR 0-1★ in outcome  (*) Found in both PubMed and Web of Science | | | | | | | | | | | |

| **Supplemental Table 7B. Risk of Bias Assessment for Case-Control Studies (N = 39)** | | | | | | | | | | | |
| --- | --- | --- | --- | --- | --- | --- | --- | --- | --- | --- | --- |
|  | **Study** | **Selection** | | | | **Comparability** | **Exposure** | | | **Total Score** | **Paper Quality** |
|  |  | **SL1** | **SL2** | **SL3** | **SL4** | **CP** | **EP1** | **EP2** | **EP3** |  |  |
|  | **PubMed (N = 23)** | | | | | | | | | | |
| 1 | **Baroudi, 2014*** | ★ | ★ | - | ★ | - | - | ★ | ★ | 5/9 | Poor |
| 2 | **Filiberti, 2015** | ★ | ★ | ★ | ★ | ★★ | ★ | ★ | ★ | 9/9 | Good |
| 3 | **Kayamba, 2015** | ★ | ★ | - | ★ | ★★ | - | ★ | ★ | 7/9 | Good |
| 4 | **Koutlas, 2018** | - | ★ | ★ | - | ★★ | ★ | ★ | ★ | 7/9 | Fair |
| 5 | **Koca, 2015** | ★ | ★ | ★ | ★ | ★★ | - | ★ | ★ | 8/9 | Good |
| 6 | **Lai, 2016** | ★ | ★ | - | ★ | ★★ | - | ★ | ★ | 7/9 | Good |
| 7 | **Lee, 2016** | ★ | ★ | - | ★ | ★★ | - | ★ | ★ | 7/9 | Good |
| 8 | **Lin, 2020** | ★ | ★ | ★ | ★ | ★★ | - | ★ | ★ | 8/9 | Good |
| 9 | **Matsuzaki, 2015** | ★ | ★ | - | ★ | ★ | - | ★ | ★ | 6/9 | Good |
| 10 | **Miftahussurur, 2018** | ★ | ★ | - | ★ | ★ | - | ★ | ★ | 6/9 | Good |
| 11 | **Mlombe, 2015** | ★ | ★ | ★ | ★ | ★★ | - | ★ | ★ | 8/9 | Good |
| 12 | **Nguyen, 2022** | ★ | ★ | - | - | ★★ | - | ★ | ★ | 6/9 | Fair |
| 13 | **Okello, 2016** | ★ | ★ | - | - | ★ | ★ | ★ | ★ | 6/9 | Fair |
| 14 | **Rafiq, 2020*** | ★ | ★ | - | - | ★★ | - | ★ | ★ | 6/9 | Fair |
| 15 | **Ramos, 2018** | ★ | ★ | - | ★ | ★★ | - | ★ | ★ | 7/9 | Good |
| 16 | **Schmidt, 2020** | ★ | ★ | ★ | ★ | ★ | - | ★ | ★ | 7/9 | Good |
| 17 | **Sewram, 2016** | ★ | ★ | - | - | ★★ | - | ★ | ★ | 6/9 | Fair |
| 18 | **Simba, 2023** | ★ | ★ | - | ★ | ★★ | - | ★ | ★ | 7/9 | Good |
| 19 | **Thrift, 2022*** | ★ | ★ | ★ | ★ | ★★ | - | ★ | ★ | 8/9 | Good |
| 20 | **Wei, 2021** | ★ | ★ | - | ★ | ★★ | - | ★ | ★ | 7/9 | Good |
| 21 | **Yang, 2020** | ★ | ★ | - | ★ | ★★ | - | ★ | ★ | 7/9 | Good |
| 22 | **Yang, 2017*** | ★ | ★ | ★ | - | ★★ | - | ★ | - | 6/9 | Poor |
| 23 | **Zhou, 2017*** | ★ | ★ | ★ | ★ | ★★ | - | ★ | - | 7/9 | Poor |
|  | **Web of Science (N = 16)** | | | | | | | | | | |
| 1 | **Asombang, 2016** | ★ | ★ | ★ | - | ★★ | ★ | ★ | - | 7/9 | Good |
| 2 | **Fang, 2015** | ★ | ★ | - | - | ★★ | ★ | ★ | ★ | 7/9 | Fair |
| 3 | **Flores-Luna, 2020** | ★ | ★ | - | - | ★★ | ★ | ★ | ★ | 7/9 | Fair |
| 4 | **Gado, 2015** | ★ | ★ | - | ★ | ★★ | - | ★ | ★ | 7/9 | Good |
| 5 | **Ghanadi, 2018** | - | - | - | - | ★★ | ★ | ★ | ★ | 5/9 | Poor |
| 6 | **Ghosh, 2021** | - | ★ | - | - | - | ★ | ★ | ★ | 4/9 | Poor |
| 7 | **Hazarika, 2016** | - | - | - | - | - | - | ★ | ★ | 2/9 | Poor |
| 8 | **Jideh, 2017** | - | ★ | - | - | - | - | ★ | ★ | 3/9 | Poor |
| 9 | **Kaimila, 2023** | ★ | ★ | - | - | ★★ | ★ | ★ | ★ | 7/9 | Fair |
| 10 | **Liu, 2022** | ★ | ★ | - | ★ | ★★ | ★ | ★ | ★ | 8/9 | Good |
| 11 | **Meyers, 2017** | ★ | ★ | ★ | ★ | ★★ | ★ | ★ | ★ | 9/9 | Good |
| 12 | **Pan, 2022** | ★ | ★ | ★ | - | ★★ | ★ | ★ | ★ | 8/9 | Good |
| 13 | **Poosari, 2021** | ★ | ★ | - | ★ | ★★ | ★ | ★ | ★ | 8/9 | Good |
| 14 | **Pournaghi, 2019** | ★ | ★ | - | - | - | ★ | ★ | ★ | 5/9 | Poor |
| 15 | **Wang, Shih, 2022** | - | - | - | - | ★★ | ★ | - | ★ | 4/9 | Poor |
| 16 | **Zhang, 2021** | ★ | ★ | ★ | - | ★★ | ★ | ★ | ★ | 8/9 | Good |
| **Abbreviations:** **SL** Selection; **CP** Comparability; **EP** Exposure. **N/A*** Not applicable; **Max** maximum  **SL1**: Adequacy of case definitions (Yes with independent validation ★, yes with record linkage or self-reports, no description) Max: ★  **SL2**: Representativeness of the cases (consecutive or obviously represented series of cases ★, potential for selection biases or not stated) Max: ★  **SL3**: Selection of controls (community controls ★, hospital controls, no description) Max: ★  **SL4**: Definition of controls (If cases are first occurrence of outcome, then controls must have no history of outcome, if cases have new, not first, occurrence of outcome, then controls with previous occurrences should not be excluded ★) Max: ★  **CP**: Comparability of cases and controls on the basis of the design or analysis (study controls for basic confounders: age, sex, marital status, etc. ★; controls for other study-specific confounders ★) Max: ★★  **EP1**: Exposure ascertainment (Secure record ★, structured interview blinded ★, interview not blinded, written self-report or medical record only, no description) Max: ★  **EP2**: Same method of ascertainment for cases and controls (Yes ★, No) Maximum: ★  **EP3**: Non-response rate (Same rate for both groups ★, non-respondents described, rate different and no designation) Maximum: ★  Converting NOS to AHRQ Standards (Good, fair, and poor quality)  Good: 3-4★ in selection AND 1-2★ in comparability AND 2-3★ in outcome;  Fair: 2★ in selection AND 1-2 in comparability AND 2-3 outcome; Poor: 0-1★ in selection OR 0★ in comparability OR 0-1★ in outcome | | | | | | | | | | | |

| **Supplemental Table 7C. Risk of bias assessment for cross-sectional studies (N = 29)** | | | | | | | | | | |
| --- | --- | --- | --- | --- | --- | --- | --- | --- | --- | --- |
|  | **Study** | **Selection** | | | | **Comparability** | **Outcome** | | **Total Score** | **Paper Quality** |
|  |  | **SL1** | **SL2** | **SL3** | **SL4** | **CP** | **OC1** | **OC2** |  |  |
|  | **PubMed (N = 18)** | | | | | | | | | |
| 1 | **Chuang, 2019*** | ★ | ★ | - | ★★ | ★★ | ★★ | ★ | 9/10 | Good |
| 2 | **Ghoshal, 2021** | ★ | - | - | ★★ | ★★ | ★ | ★ | 8/10 | Good |
| 3 | **Kim, Jung, 2019** | ★ | ★ | - | ★ | ★★ | ★★ | ★ | 8/10 | Good |
| 4 | **Li, 2022** | ★ | ★ | N/A | ★★ | ★★ | ★★ | ★ | 9/9 | Good |
| 5 | **Li, 2021** | ★ | ★ | N/A | ★★ | ★★ | ★★ | ★ | 9/9 | Good |
| 6 | **Li, 2024** | ★ | ★ | N/A | ★★ | ★★ | ★★ | ★ | 9/9 | Good |
| 7 | **Lin, 2022** | ★ | ★ | N/A | ★★ | ★★ | ★★ | ★ | 9/9 | Good |
| 8 | **Martinucci, 2018** | ★ | ★ | - | ★ | ★★ | ★ | ★ | 7/10 | Good |
| 9 | **Navab, 2015** | ★ | ★ | N/A | - | - | ★★ | - | 4/9 | Unsatisfactory |
| 10 | **Okamoto, 2023** | ★ | ★ | N/A | ★ | ★★ | ★★ | ★ | 8/9 | Good |
| 11 | **Pan, 2019*** | ★ | ★ | N/A | ★ | ★★ | ★★ | ★ | 9/9 | Good |
| 12 | **Rao, 2022** | ★ | ★ | N/A | ★ | - | ★★ | ★ | 6/9 | Satisfactory |
| 13 | **Sadafi, 2024*** | ★ | ★ | N/A | ★ | ★ | ★★ | ★ | 7/9 | Good |
| 14 | **Seo, 2020** | ★ | ★ | N/A | ★★ | - | ★★ | ★ | 7/9 | Good |
| 15 | **Song, 2024** | ★ | ★ | N/A | - | - | ★★ | ★ | 5/9 | Satisfactory |
| 16 | **Wang, 2016** | ★ | ★ | N/A | - | - | ★ | ★ | 4/9 | Unsatisfactory |
| 17 | **Wong, 2016** | ★ | ★ | N/A | ★★ | ★ | ★★ | ★ | 8/9 | Good |
| 18 | **Yu, 2021** | ★ | ★ | N/A | ★ | ★★ | ★★ | ★ | 8/9 | Good |
|  | **Web of Science (N = 11)** | | | | | | | | | |
| 1 | **Ahmed, 2020** | - | - | N/A | ★★ | - | ★★ | ★ | 5/9 | Satisfactory |
| 2 | **Al-Towairqi, 2020** | ★ | - | N/A | ★★ | - | ★★ | ★ | 6/9 | Satisfactory |
| 3 | **Alrashed, 2019** | ★ | ★ | N/A | ★★ | ★ | ★★ | ★ | 8/9 | Good |
| 4 | **Chen, Zheng, 2023** | ★ | ★ | N/A | ★★ | ★★ | ★★ | ★ | 9/9 | Good |
| 5 | **Fan, 2023** | ★ | ★ | N/A | ★★ | ★★ | ★★ | ★ | 9/9 | Good |
| 6 | **Guo, 2019** | ★ | ★ | N/A | ★★ | ★★ | ★★ | ★ | 9/9 | Good |
| 7 | **Huang, 2017** | ★ | ★ | N/A | ★★ | ★★ | ★★ | ★ | 9/9 | Good |
| 8 | **Lu, 2020** | ★ | ★ | N/A | ★★ | ★★ | ★★ | ★ | 9/9 | Good |
| 9 | **Ness-Jensen, 2023** | ★ | ★ | N/A | ★★ | ★★ | ★★ | ★ | 9/9 | Good |
| 10 | **Ohashi, 2021** | ★ | ★ | N/A | ★★ | ★★ | ★★ | ★ | 9/9 | Good |
| 11 | **Wang, Zhang, 2019** | ★ | ★ | N/A | ★★ | ★★ | ★★ | ★ | 9/9 | Good |
| **Abbreviations:** **SL** Selection; **CP** Comparability; **EP** Exposure. **N/A*** Not applicable.  **SL1**: Representativeness of the exposed cohort (truly representative of the average ★, somewhat representative ★, selected group of users, no description of derivation of cohort) Maximum: ★  **SL2**: Sample size (justified and satisfactory ★, not justified, no information provided) Maximum: ★  **SL3**: Non-respondents. (comparability between respondent and non-respondent characteristics is established, and the response rate is satisfactory ★) Max: ★  **SL4**: Ascertainment of the exposure (risk factor): (Validated measurement tool ★★, non-validated measurement tool, but the tool is available or described★, no description of measurement tool) Max: ★★  **CP**: Comparability of cases and controls on the basis of the design or analysis (study controls for basic confounders: age, sex, marital status, etc. ★; controls for other study-specific confounders ★) Max: ★★  **OC1**: Assessment of outcome (Independent blind assessment ★★, record linkage ★★, self-report ★, no description) Max: ★★  **OC2**: Statistical Test (statistical test used is clearly described and appropriate, and measurement of association is presented, including confidence intervals and p-value ★) Max: ★  Good: 7-8★; Satisfactory: 5-6★; Unsatisfactory: 0-4★ | | | | | | | | | | |
