# Supplementary material for: Gastroesophageal disease risk and inhalational exposure a systematic review and meta-analysis
Source: Sci Rep. 2025 Jul 2;15:22581. doi: 10.1038/s41598-025-06620-7 (PMC12218983; doi:10.1038/s41598-025-06620-7)
Supplement: Supplementary file 8 — Supplementary Material 8. [file 41598_2025_6620_MOESM8_ESM.docx]

| **Supplemental Table 8A. Raw Data Set of Odds Ratios for Esophageal Diseases** | | | | | | | | | | |
| --- | --- | --- | --- | --- | --- | --- | --- | --- | --- | --- |
|  |  | **Esophagitis** | | | **BE** | | | **ECa** | | |
|  |  | **Mean** | **Lower Limit** | **Upper Limit** | **Mean** | **Lower Limit** | **Upper Limit** | **Mean** | **Lower Limit** | **Upper Limit** |
| **1** | Chuang, 2019 | 1.26 | 1.09 | 1.46 |  |  |  |  |  |  |
| **2** | Crews, 2016 | 0.9 | 0.5 | 1.9 |  |  |  |  |  |  |
| **3** | Filiberti, 2015 | 1.31 | 0.8 | 2.17 |  |  |  |  |  |  |
| **4** | Gado, 2015 | 1.99 | 1.3 | 3.1 |  |  |  |  |  |  |
| **5** | Kim, Jung, 2019 | 1.67 | 1.3 | 2.16 |  |  |  |  |  |  |
| **6** | Kim, Lee, 2014 | 1.015 | 1.004 | 1.025 |  |  |  |  |  |  |
| **7** | Koutlas, 2018 | 0.36 | 0.17 | 0.76 |  |  |  |  |  |  |
| **8** | Lee, 2016 | 1.366 | 1.068 | 1.748 |  |  |  |  |  |  |
| **9** | Liu, 2022 | 2.41 | 2.41 | 4.06 |  |  |  |  |  |  |
| **10** | Matsuzaki, 2015 | 1.79 | 1.23 | 2.6 |  |  |  |  |  |  |
| **11** | Ohashi, 2021 | 1.94 | 1.56 | 2.42 |  |  |  |  |  |  |
| **12** | Okamoto, 2023 | 2.4 | 1.5 | 3.9 |  |  |  |  |  |  |
| **13** | Wang, Zhang, 2019 | 1.41 | 1.01 | 1.98 |  |  |  |  |  |  |
| **14** | Chuang, 2019 | 1.26 | 1.09 | 1.46 |  |  |  |  |  |  |
|  |  | | | | | | | | | |
| **1** | Chuang, 2019 |  |  |  | 1.470 | 1.080 | 2.000 |  |  |  |
| **2** | Crews, 2016 |  |  |  | 0.900 | 0.500 | 1.900 |  |  |  |
| **3** | Dore, 2016 |  |  |  | 0.447 | 0.199 | 1.002 |  |  |  |
| **4** | Filiberti, 2015 |  |  |  | 1.860 | 0.980 | 3.160 |  |  |  |
| **5** | Matsuzaki, 2015 |  |  |  | 1.370 | 0.830 | 2.260 |  |  |  |
|  |  | | | | | | | | | |
| **1** | Asombang, 2016 |  |  |  |  |  |  | 11.24 | 1.37 | 92.4 |
| **2** | Chen, Li, 2021 |  |  |  |  |  |  | 1.58 | 1.14 | 2.18 |
| **3** | Chen, Peto, 2015 |  |  |  |  |  |  | 1.47 | 1.25 | 1.73 |
| **4** | Chuang, 2019 |  |  |  |  |  |  | 1.4 | 0.57 | 3.43 |
| **5** | Guo, 2019" |  |  |  |  |  |  | 1.35 | 0.92 | 1.98 |
| **6** | Kayamba, 2015 |  |  |  |  |  |  | 8 | 2.8 | 22.7 |
| **7** | Kim, Gong, 2014 |  |  |  |  |  |  | 8.317 | 0.94 | 73.583 |
| **8** | Kunzmann, 2018 |  |  |  |  |  |  | 3.83 | 5.66 | 2.59 |
| **9** | Lu, 2020 |  |  |  |  |  |  | 1.26 | 1 | 1.57 |
| **10** | Meyers, 2017 |  |  |  |  |  |  | 1.46 | 1.24 | 1.73 |
| **11** | Mlombe, 2015 |  |  |  |  |  |  | 5.4 | 2 | 15.2 |
| **12** | Moses, 2017 |  |  |  |  |  |  | 2.02 | N/A | N/A |
| **13** | Okello, 2016 |  |  |  |  |  |  | 1.38 | 0.41 | 4.67 |
| **14** | Pan, 2019 |  |  |  |  |  |  | 1.75 | 1.09 | 2.8 |
| **15** | Pan, 2022 |  |  |  |  |  |  | 3.11 | 1 | 9.63 |
| **16** | Poosari, 2021 |  |  |  |  |  |  | 3.5 | 9.56 | 1.28 |
| **17** | Pournaghi, 2019 |  |  |  |  |  |  | 1.1 | 0.6 | 2.04 |
| **18** | Rafiq, 2020 |  |  |  |  |  |  | 1.49 | 1.08 | 2.04 |
| **19** | Rafiq, 2020 |  |  |  |  |  |  | 1.25 | 0.66 | 2.38 |
| **20** | Sewram, 2016 |  |  |  |  |  |  | 4.11 | 2.55 | 6.65 |
| **21** | Simba, 2023 |  |  |  |  |  |  | 2.15 | 1.72 | 2.68 |
| **22** | Wang, Shih, 2022 |  |  |  |  |  |  | 4.7 | 1.6 | 13.5 |
| **23** | Wei, 2021 |  |  |  |  |  |  | 3.11 | 1.63 | 6.05 |
| **24** | Yang, 2020 |  |  |  |  |  |  | 2.24 | 1.2 | 4.19 |
| **25** | Yang, 2017 |  |  |  |  |  |  | 1.12 | 0.88 | 1.44 |
| **26** | Zhou, 2017 |  |  |  |  |  |  | 1.68 | 1.5 | 1.87 |

| **Supplemental Table 8B. Raw Data Set of Odds Ratios for Gastric Diseases** | | | | | | | | | | |
| --- | --- | --- | --- | --- | --- | --- | --- | --- | --- | --- |
|  |  | **GERD** | | | **PUD** | | | **GCa** | | |
|  |  | **Mean** | **Lower Limit** | **Upper Limit** | **Mean** | **Lower Limit** | **Upper Limit** | **Mean** | **Lower Limit** | **Upper Limit** |
| **1** | Ahmed, 2020 | 6.25 | 4.4 | 8.91 |  |  |  |  |  |  |
| **2** | Almadi, 2014 | 1.34 | 0.95 | 1.87 |  |  |  |  |  |  |
| **3** | Dore, 2016 | 1.392 | 1.085 | 1.787 |  |  |  |  |  |  |
| **4** | Etemadi, 2017 | 1.2 | 1.02 | 1.4 |  |  |  |  |  |  |
| **5** | Etemadi, 2017 | 1.3 | 1.08 | 1.56 |  |  |  |  |  |  |
| **6** | Ghoshal, 2017 | 1.36 | 0.99 | 1.88 |  |  |  |  |  |  |
| **7** | Kim, 2018 | 2.31 | 0.94 | 5.66 |  |  |  |  |  |  |
| **8** | Kim, Jung, 2019 | 1.48 | 0.85 | 2.57 |  |  |  |  |  |  |
| **9** | Martinucci, 2018 | 1.6 | 1.25 | 2.05 |  |  |  |  |  |  |
| **10** | Miftahussurur, 2018 | 3.6 | 1.298 | 9.955 |  |  |  |  |  |  |
| **11** | Ness-Jensen, 2023 | 1.14 | 1.01 | 2.29 |  |  |  |  |  |  |
| **12** | Rabiee, 2016 | 3.53 | 2.17 | 5.74 |  |  |  |  |  |  |
| **13** | Sadafi, 2024 | 1.23 | 1.02 | 1.55 |  |  |  |  |  |  |
| **14** | Wang, 2016 | 0.7 | 0.4 | 1.2 |  |  |  |  |  |  |
| **15** | Wang, Kendall, 2022 | 1.13 | 0.93 | 1.38 |  |  |  |  |  |  |
|  |  | | | | | | | | | |
| **1** | Chuang, 2019 |  |  |  | 1.790 | 1.520 | 2.100 |  |  |  |
| **2** | Ghanadi, 2018 |  |  |  | 4.750 | 1.61 | 8.200 |  |  |  |
| **3** | Quan, 2015 |  |  |  | 0.750 | 0.610 | 0.900 |  |  |  |
| **4** | Quan, 2015 |  |  |  | 0.870 | 0.750 | 1.000 |  |  |  |
| **5** | Tsai, 2019 |  |  |  | 1.140 | 1.090 | 1.180 |  |  |  |
| **6** | Tsai, 2019 |  |  |  | 1.050 | 1.010 | 1.080 |  |  |  |
| **7** | Yu, 2021 |  |  |  | 1.178 | 1.118 | 1.242 |  |  |  |
|  |  | | | | | | | | | |
| **1** | Baroudi, 2014 |  |  |  |  |  |  | 3.66 | 1.82 | 7.78 |
| **2** | Chen, Zheng, 2023 |  |  |  |  |  |  | 1.26 | 1.07 | 1.43 |
| **3** | Chen, Peto, 2015 |  |  |  |  |  |  | 1.34 | 1.16 | 1.55 |
| **4** | Chuang, 2019 |  |  |  |  |  |  | 1.24 | 0.53 | 2.91 |
| **5** | Fang, 2015 |  |  |  |  |  |  | 1.47 | 1.01 | 2.14 |
| **6** | Flores-Luna, 2020 |  |  |  |  |  |  | 1.3 | 0.9 | 2 |
| **7** | Ghosh, 2021 |  |  |  |  |  |  | 3.14 | N/A | N/A |
| **8** | Guo, 2019 |  |  |  |  |  |  | 0.55 | 0.23 | 1.34 |
| **9** | Lai, 2016 |  |  |  |  |  |  | 1.1 | 0.8 | 1.4 |
| **10** | Lai, 2016 |  |  |  |  |  |  | 1.8 | 1.3 | 2.4 |
| **11** | Lim, 2021 |  |  |  |  |  |  | 0.667 | 0.25 | 1.72 |
| **12** | Lin, 2020 |  |  |  |  |  |  | 1.83 | 1.19 | 2.8 |
| **13** | Nguyen, 2022 |  |  |  |  |  |  | 3.26 | 1.24 | 8.55 |
| **14** | Nguyen, 2022 |  |  |  |  |  |  | 2.9 | 1.05 | 7.97 |
| **15** | Ramos, 2018 |  |  |  |  |  |  | 2.67 | 1.72 | 4.13 |
| **16** | Song, 2024 |  |  |  |  |  |  | 0.971 | 0.694 | 1.359 |
| **17** | Thrift, 2022 |  |  |  |  |  |  | 2.05 | 1.47 | 2.85 |
| **18** | Zacharakis, 2023 |  |  |  |  |  |  | 4 | 2.05 | 7.81 |
| **19** | Zhao, 2017 |  |  |  |  |  |  | 1.61 | 1.43 | 1.81 |
| **20** | Zhang, 2021 |  |  |  |  |  |  | 3.06 | 1.7 | 5.54 |

| **Supplemental Table 8C. Raw Data Set of Risk Ratios** | | | | | | | | | | | | | |
| --- | --- | --- | --- | --- | --- | --- | --- | --- | --- | --- | --- | --- | --- |
|  |  | **ECa** | | | **PUD** | | | **GCa** | | | **Esophagitis** | | |
|  |  | **Mean** | **Lower Limit** | **Upper Limit** | **Mean** | **Lower Limit** | **Upper Limit** | **Mean** | **Lower Limit** | **Upper Limit** | **Mean** | **Lower Limit** | **Upper Limit** |
| **1** | Ethan, 2020 |  |  |  |  |  |  | 1.0003 | 1.0001 | 1.002 |  |  |  |
| **2** | Fan, 2023 |  |  |  |  |  |  | 1.028 | 1.011 | 1.046 |  |  |  |
| **3** | Ghosh, 2021 |  |  |  |  |  |  | 1.18 | N/A | N/A |  |  |  |
| **4** | Jayalekshmi, 2015 |  |  |  |  |  |  | 0.8 | 0.5 | 1.2 |  |  |  |
| **5** | Jayalekshmi, 2021 | 1.3 | 0.9 | 1.8 |  |  |  |  |  |  |  |  |  |
| **6** | Kang, 2016 |  |  |  |  |  |  |  |  |  | 2.7 | 2.26 | 3.23 |
| **7** | Wu, 2021 |  |  |  | 2.4 | 1.36 | 4.24 |  |  |  |  |  |  |
| **8** | Wu, 2021 |  |  |  | 1.65 | 0.98 | 2.76 |  |  |  |  |  |  |

| **Supplemental Table 8D. Raw Data Set of Correlation Coefficients** | | | | | | | |
| --- | --- | --- | --- | --- | --- | --- | --- |
|  |  | **BE** | | | **ECa** | | |
|  |  | **Mean** | **Lower Limit** | **Upper Limit** | **Mean** | **Lower Limit** | **Upper Limit** |
| 1 | Huang, 2017 |  |  |  | 0.51 |  |  |
| 2 | Lin, 2022 |  |  |  | -0.17 | -0.22 | -0.12 |
| 3 | Navab, 2015 | 0.73 | 0.5 | 1.06 |  |  |  |
| 4 | Rao, 2022 |  |  |  | -0.365 | N/A | N/A |

| **Supplemental Table 8E. Raw Data Set of Hazard Ratios** | | | | | | | | | | | | | |
| --- | --- | --- | --- | --- | --- | --- | --- | --- | --- | --- | --- | --- | --- |
|  |  | **BE/GERD** | | | **ECa** | | | **PUD** | | | **GCa** | | |
|  |  | **Mean** | **Lower Limit** | **Upper Limit** | **Mean** | **Lower Limit** | **Upper Limit** | **Mean** | **Lower Limit** | **Upper Limit** | **Mean** | **Lower Limit** | **Upper Limit** |
| 1 | **Soroush, 2023** | 0.26 | 0.83 | 0.08 |  |  |  |  |  |  |  |  |  |
| 2 | **Yates, 2014** | 1.570 | 0.830 | 2.960 |  |  |  |  |  |  |  |  |  |
|  |  | | | | | | | | | | | | |
| 1 | **Dighe, 2021** |  |  |  | 2.54 | 1.42 | 4.53 |  |  |  |  |  |  |
| 2 | **Kaimila, 2023** |  |  |  | 1.06 | 0.81 | 1.38 |  |  |  |  |  |  |
| 3 | **Laaksonen, 2023** |  |  |  | 3.27 | 1.84 | 5.8 |  |  |  |  |  |  |
| 4 | **Sheikh, 2019** |  |  |  | 1.35 | 0.09 | 2.02 |  |  |  |  |  |  |
| 5 | **Spreafico, 2017** |  |  |  | 1.22 | 1.15 | 1.43 |  |  |  |  |  |  |
| 6 | **Sun, 2023** |  |  |  | 1.18 | 1.04 | 1.35 |  |  |  |  |  |  |
| 7 | **Sun, 2023** |  |  |  | 1.32 | 1.01 | 1.72 |  |  |  |  |  |  |
| 8 | **Wang, 2021** |  |  |  | 3.16 | 2.54 | 3.92 |  |  |  |  |  |  |
| 9 | **Yates, 2014** |  |  |  | 1.82 | 0.81 | 4.09 |  |  |  |  |  |  |
|  |  | | | | | | | | | | | | |
| 1 | **Levenstein, 2017** |  |  |  |  |  |  | 1.800 | 1.100 | 2.800 |  |  |  |
| 2 | **Park, Kim, Jung, 2022** |  |  |  |  |  |  | 1.379 | 1.256 | 1.513 |  |  |  |
| 3 | **Wong, 2016** |  |  |  |  |  |  | 1.590 | 1.370 | 1.840 |  |  |  |
| 4 | **Wong, 2016** |  |  |  |  |  |  | 1.180 | 1.020 | 1.360 |  |  |  |
|  |  | | | | | | | | | | | | |
| 1 | **Kim, Chang, 2019** |  |  |  |  |  |  |  |  |  | 1.51 | 1.41 | 1.61 |
| 2 | **Kumar, 2020** |  |  |  |  |  |  |  |  |  | 1.38 | 1.25 | 1.52 |
| 3 | **Laaksonen, 2023** |  |  |  |  |  |  |  |  |  | 1.96 | 1.07 | 3.59 |
| 4 | **Le, 2022** |  |  |  |  |  |  |  |  |  | 1.9 | 0.88 | 4.07 |
| 5 | **Le, 2022** |  |  |  |  |  |  |  |  |  | 3.22 | 1.67 | 6.21 |
| 6 | **Li, Xu, 2020** |  |  |  |  |  |  |  |  |  | 2.07 | 1.14 | 3.74 |
| 7 | **Minami, 2018** |  |  |  |  |  |  |  |  |  | 1.25 | 0.92 | 1.69 |
| 8 | **Park, Kim, Oh, 2022** |  |  |  |  |  |  |  |  |  | 1.589 | 1.355 | 1.864 |
| 9 | **Wang, 2021** |  |  |  |  |  |  |  |  |  | 3.16 | 2.42 | 4.13 |
