# Supplementary material for: Gastroesophageal disease risk and inhalational exposure a systematic review and meta-analysis
Source: Sci Rep. 2025 Jul 2;15:22581. doi: 10.1038/s41598-025-06620-7 (PMC12218983; doi:10.1038/s41598-025-06620-7)
Supplement: Supplementary file 9 — Supplementary Material 9. [file 41598_2025_6620_MOESM9_ESM.pptx]

## Slide 1
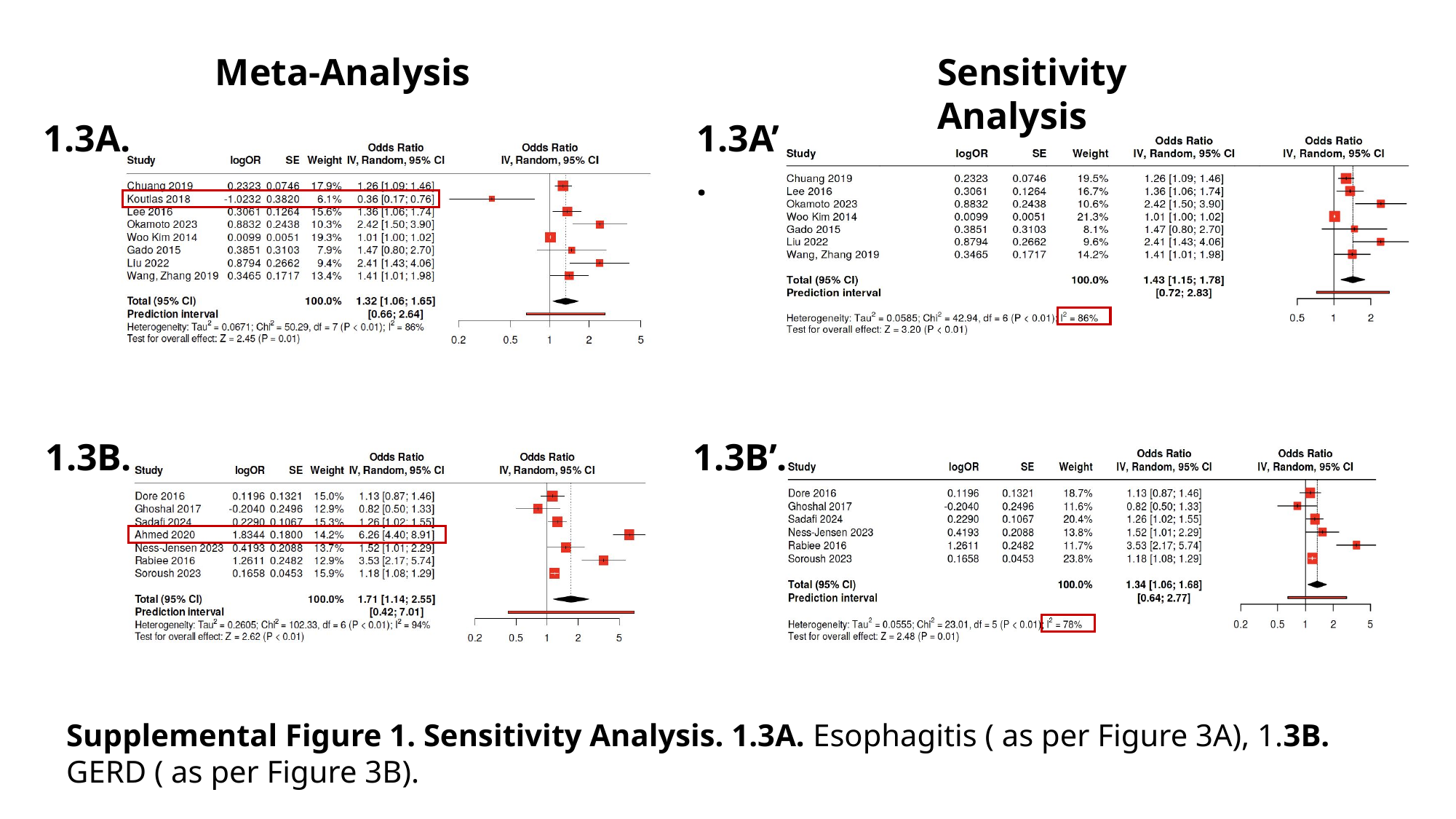

Meta-Analysis
Sensitivity Analysis
1.3A.
1.3A’.
1.3B.
1.3B’.
Supplemental Figure 1. Sensitivity Analysis. 1.3A. Esophagitis ( as per Figure 3A), 1.3B. GERD ( as per Figure 3B).
